# Supplementary material for: A practical framework RNMF for exploring the association between mutational signatures and genes using gene cumulative contribution abundance
Source: Cancer Med. 2022 May 16;11(21):4053–69. doi: 10.1002/cam4.4717 (PMC9636515; doi:10.1002/cam4.4717)
Supplement: Supplementary file 1 — Figure S1 [file CAM4-11-4053-s001.pdf]

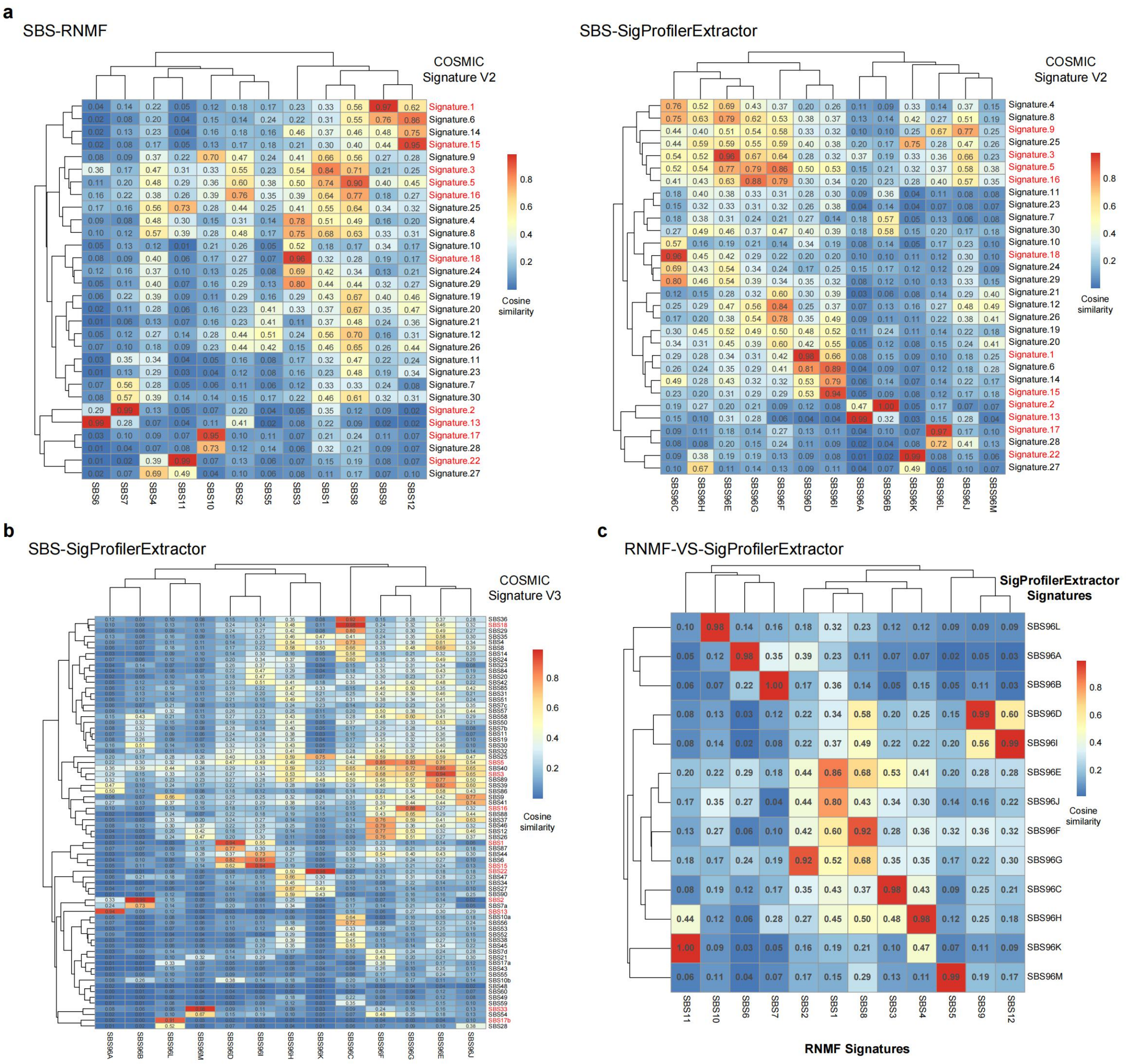

Supplementary Figure 1. Heatmap of the cosine similarity between mutational signatures and COSMIC signatures. (a) The cosine similarity between SBS signatures and COSMIC signatures with version 2 is evaluated, and the extraction methods of mutational signatures include *RNMF* and SigProfilerExtractor. (b) The cosine similarity between SBS signatures which deciphered by SigProfilerExtractor and COSMIC signatures with version 3. (c) Cosine similarity comparison of SBS signatures between *RNMF* and SigProfilerExtractor. The red mark represents the signature with the highest similarity.
