# Supplementary material for: A practical framework RNMF for exploring the association between mutational signatures and genes using gene cumulative contribution abundance
Source: Cancer Med. 2022 May 16;11(21):4053–69. doi: 10.1002/cam4.4717 (PMC9636515; doi:10.1002/cam4.4717)
Supplement: Supplementary file 2 — Figure S2 [file CAM4-11-4053-s007.pdf]

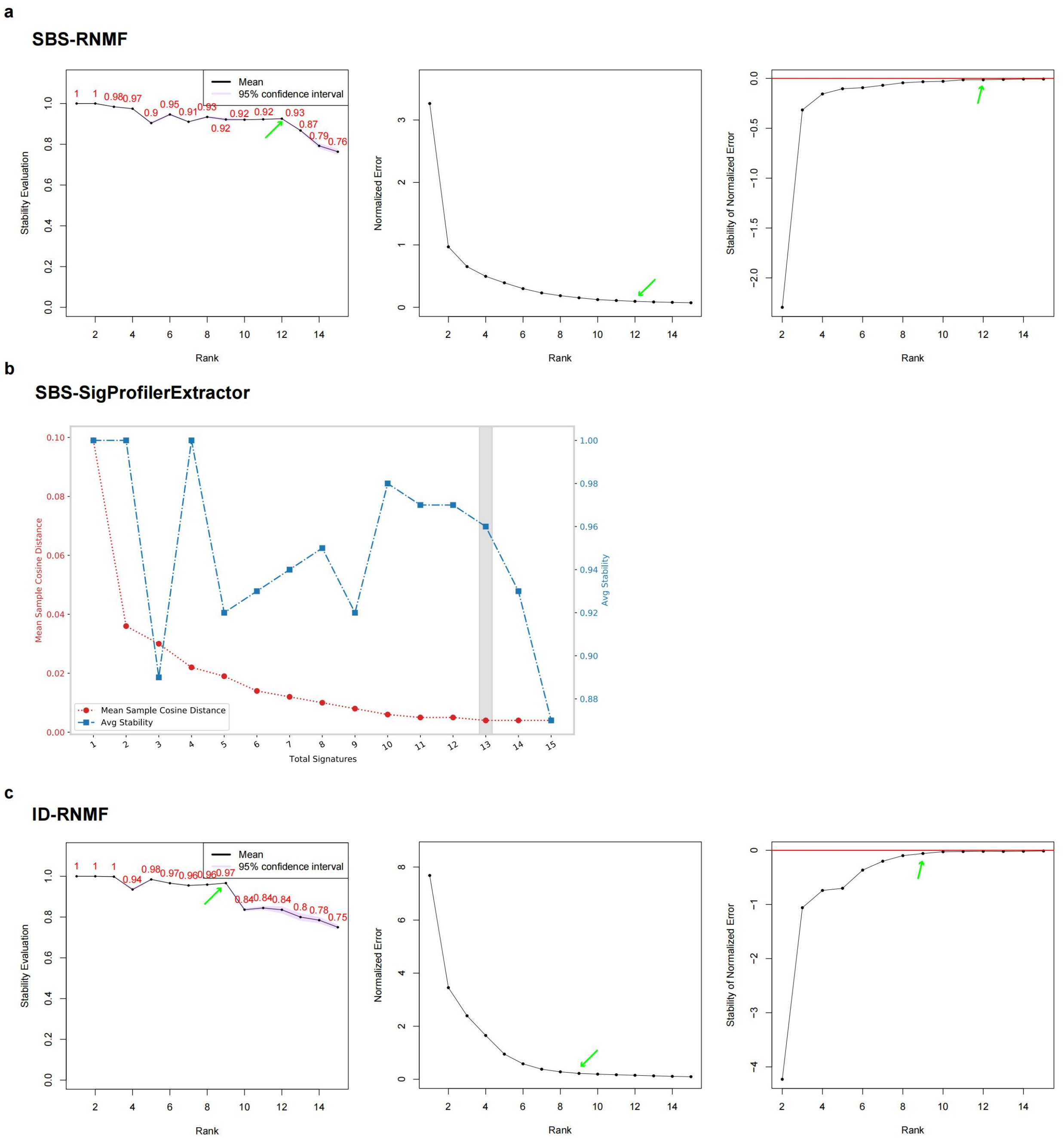

Supplementary Figure 2. Deciphering mutational signatures from a set of mutational catalogs from 508 ESCC. (a) Identifying the number of SBS signatures in a set of 508 ESCC genomes based on strong stability, low error rate and more stable gradient of error. We choose the classification with stability not less than 0.9 as the best number of SBS signatures for final decomposition, in which the green clipper on each graph indicates the current classification position to be selected. The light purple band represents the confidence region of stability change under the current classification number. (b) The classification selection is adopted by SigProfilerExtractor method, and the gray strip represents the current optimal number of selected signatures. (c) Identifying the number of ID signatures in a set of 508 ESCC genomes based on strong stability, low error rate and more stable gradient of error. We choose the classification with stability not less than 0.9 as the best number of ID signatures for final decomposition, in which the green clipper on each graph indicates the current classification position to be selected. The light purple band represents the confidence region of stability change under the current classification number.
