# Supplementary material for: A practical framework RNMF for exploring the association between mutational signatures and genes using gene cumulative contribution abundance
Source: Cancer Med. 2022 May 16;11(21):4053–69. doi: 10.1002/cam4.4717 (PMC9636515; doi:10.1002/cam4.4717)
Supplement: Supplementary file 3 — Figure S3 [file CAM4-11-4053-s013.pdf]

a

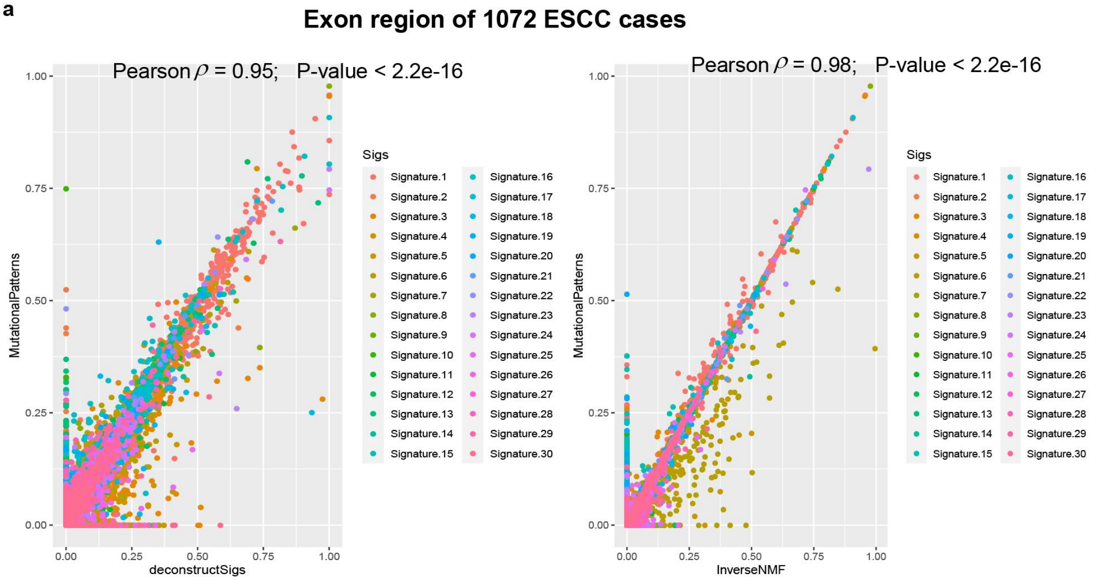

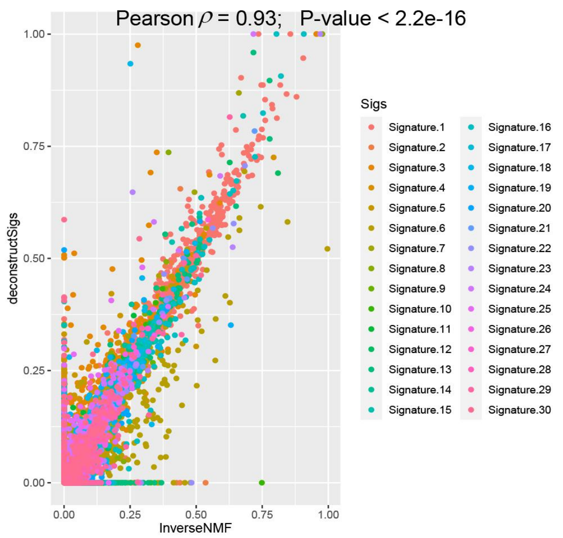

b

	InverseNMF	MutationalPatterns	deconstructSigs
Runtime (1072 samples)	44. 9 s	22. 8 s	9 min 5. 5 s

Supplementary Figure 3. **Comparison of signature contributions identified with deconstructSigs, MutationalPatterns and RNMF framework.** (a) Scatterplots represent the relationship between the weighted proportions calculated using three methods on a set of exon region of 1072 ESCC samples. Each point plotted represents the weights assigned by both methods to one signature detected in a individual. (b) The runtime (elapsed time) in seconds to find the optimal linear combination of mutational signatures from 1072 samples for both packages.
