# Supplementary material for: A practical framework RNMF for exploring the association between mutational signatures and genes using gene cumulative contribution abundance
Source: Cancer Med. 2022 May 16;11(21):4053–69. doi: 10.1002/cam4.4717 (PMC9636515; doi:10.1002/cam4.4717)
Supplement: Supplementary file 4 — Figure S4 [file CAM4-11-4053-s010.pdf]

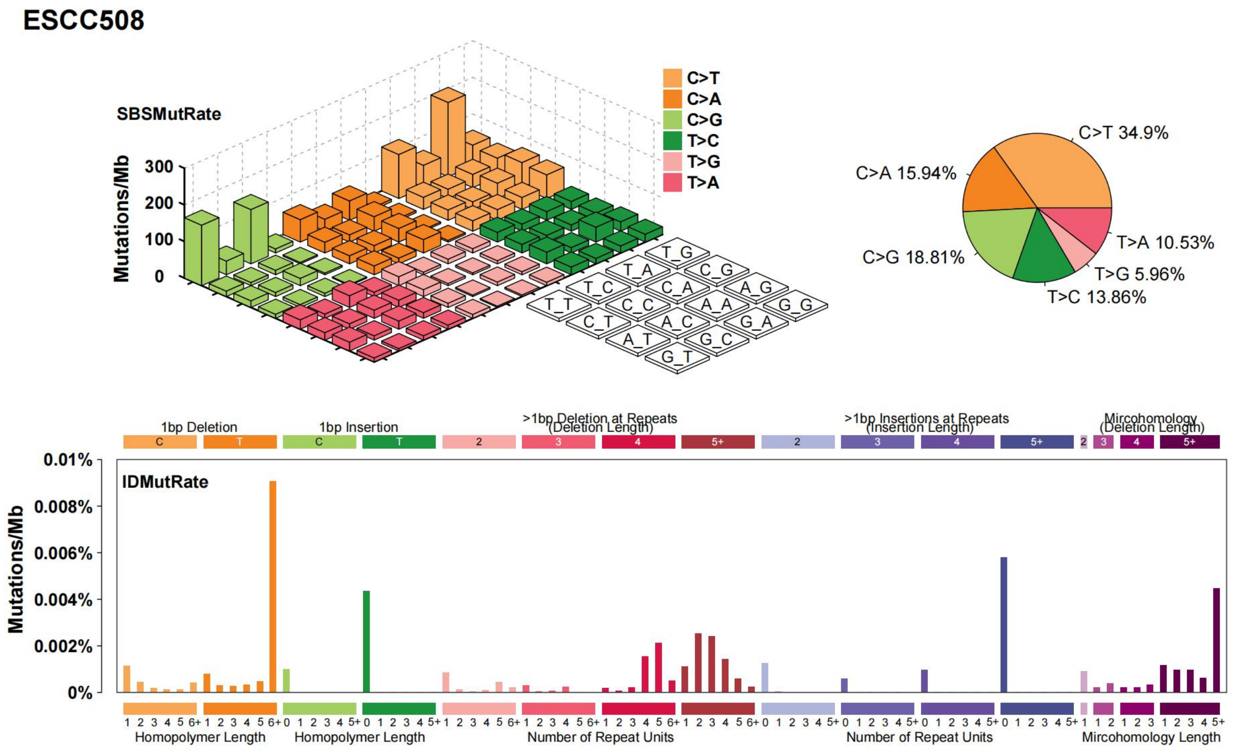

Supplementary Figure 4. Mutation rate of 508 ESCC. Lego plot and bar plot representation of mutation patterns in 508 ESCC with whole genome sequencing. Single-nucleotide substitutions are divided into six categories with 16 surrounding flanking bases. Inset pie chart shows the proportion of six categories of mutation patterns. And 83 mutation types were used to display the small insert and deletion. Each column represents the mutation proportion of this mutation type.
