# Supplementary material for: A practical framework RNMF for exploring the association between mutational signatures and genes using gene cumulative contribution abundance
Source: Cancer Med. 2022 May 16;11(21):4053–69. doi: 10.1002/cam4.4717 (PMC9636515; doi:10.1002/cam4.4717)
Supplement: Supplementary file 5 — Figure S5 [file CAM4-11-4053-s015.pdf]

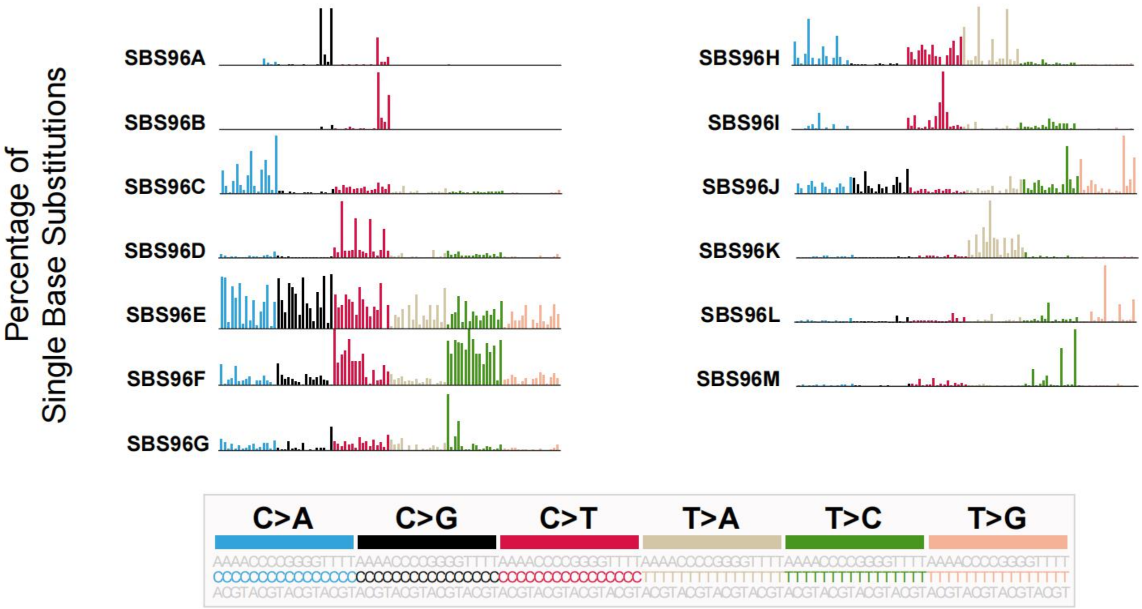

Supplementary Figure 5. SBS mutational signatures extracted from 508 WGS cases of Chinese ESCC. The classifications of each mutation type (SBS, 96 classes) are shown in the picture, separately. Each color is used to illustrate the positions of each mutation subtype on each plot.
