# Supplementary material for: A practical framework RNMF for exploring the association between mutational signatures and genes using gene cumulative contribution abundance
Source: Cancer Med. 2022 May 16;11(21):4053–69. doi: 10.1002/cam4.4717 (PMC9636515; doi:10.1002/cam4.4717)
Supplement: Supplementary file 6 — Figure S6 [file CAM4-11-4053-s016.pdf]

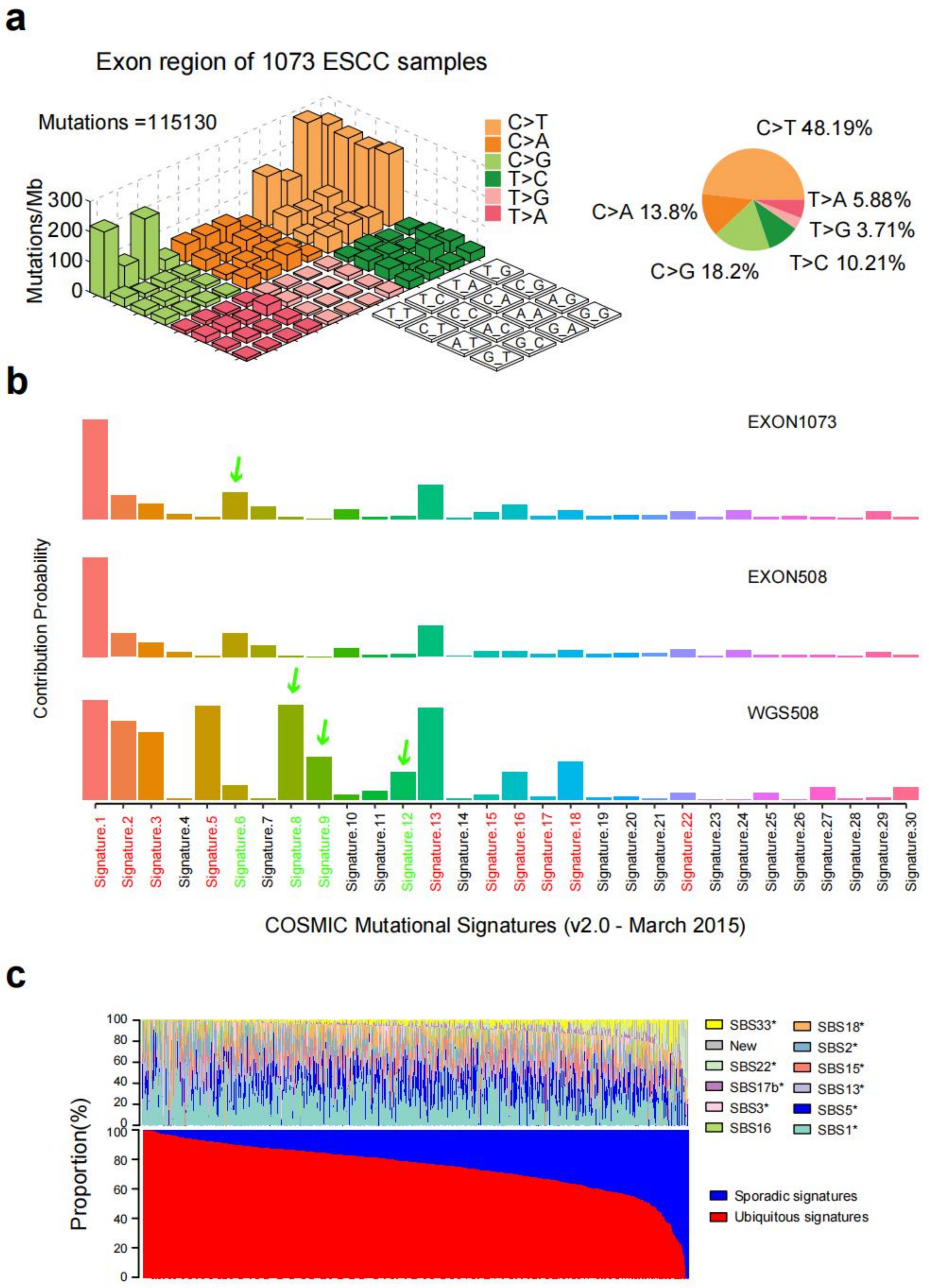

Supplementary Figure 6. (a) Lego plot representation of mutation patterns in exon region of 1073 ESCC cases. Single-nucleotide substitutions are divided into six categories with 16 surrounding flanking bases. Inset pie chart shows the proportion of six categories of mutation patterns. (b) Based on the background mutation contribution probability of COSMIC Mutational Signatures (v2.0 - March 2015), each color represents a mutational signature, the length of each column represents the contribution proportion of mutation to the signature, the red mark represents the signature most similar to the 12 mutational signature, and the green arrow and green font indicate that this signature is very similar to the 12 mutational signature. (c) The proportion of 1073 ESCC samples in 12 mutational signatures, each color represents a feature, in which red represents the proportion of ubiquitous signatures.
