# Supplementary material for: A practical framework RNMF for exploring the association between mutational signatures and genes using gene cumulative contribution abundance
Source: Cancer Med. 2022 May 16;11(21):4053–69. doi: 10.1002/cam4.4717 (PMC9636515; doi:10.1002/cam4.4717)
Supplement: Supplementary file 7 — Figure S7 [file CAM4-11-4053-s008.pdf]

a

Survival analysis

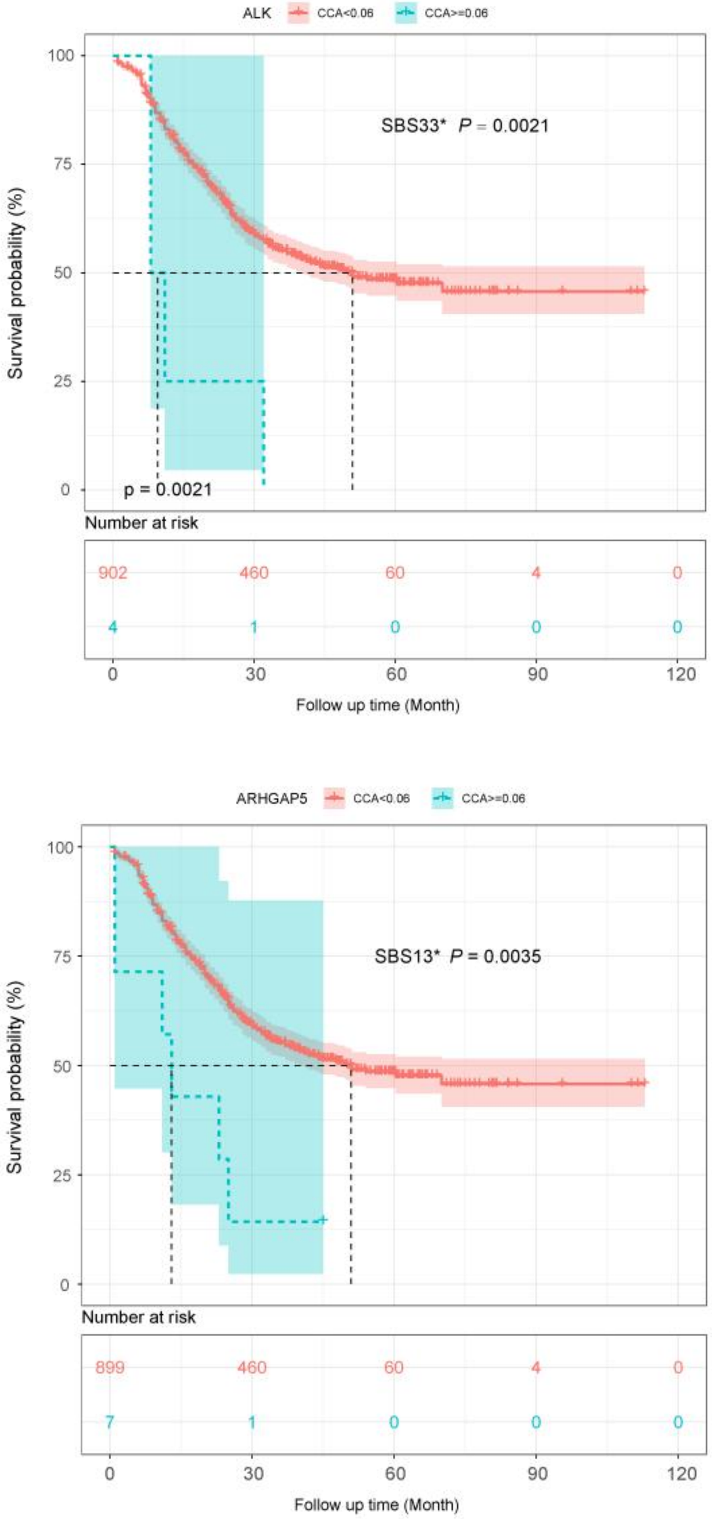

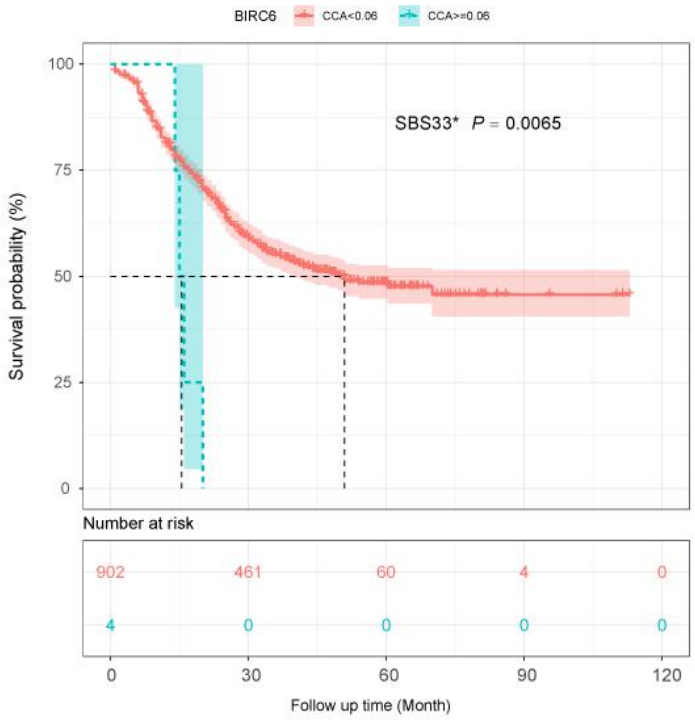

BIRC6
CCA<0.06
CCA>=0.06
SBS33* P = 0.0065
Survival probability (%)
100
75
50
25
0
Number at risk
902
461
60
4
0
4
0
0
0
0
0
30
60
90
120
Follow up time (Month)

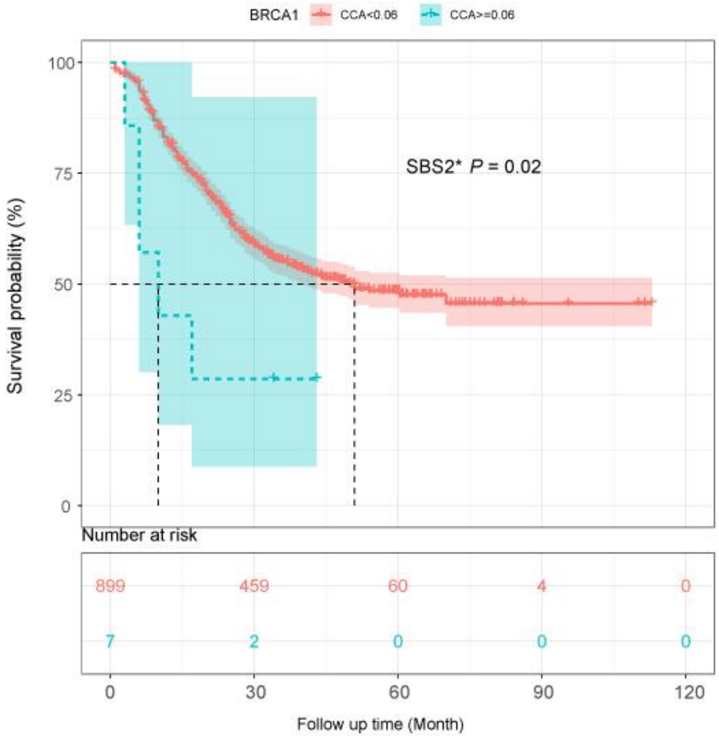

BRCA1
CCA<0.06
CCA>=0.06
SBS2* P = 0.02
Survival probability (%)
100
75
50
25
0
Number at risk
899
459
60
4
0
7
2
0
0
0
0
30
60
90
120
Follow up time (Month)

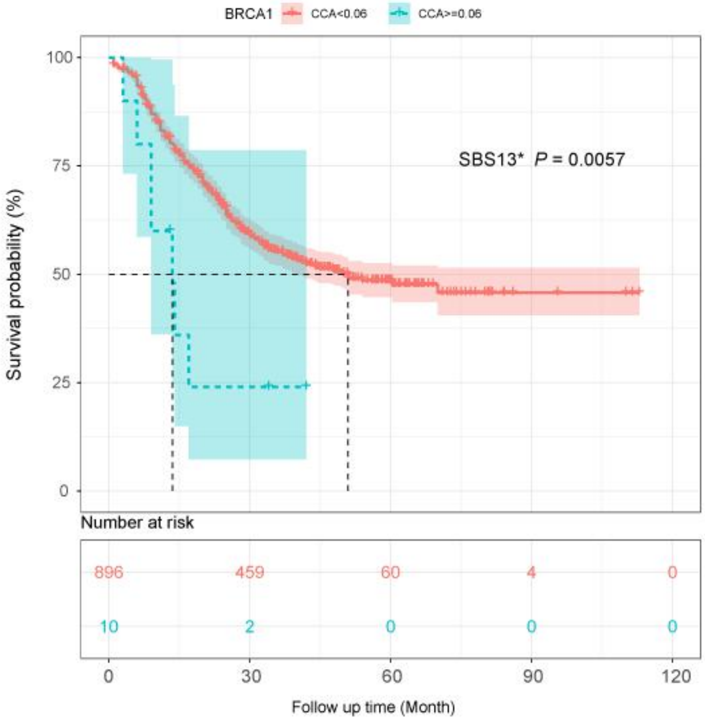
BRCA1
CCA<0.06
CCA>=0.06
SBS13* P = 0.0057
Survival probability (%)
100
75
50
25
0
Number at risk
896
459
60
4
0
10
2
0
0
0
0
30
60
90
120
Follow up time (Month)

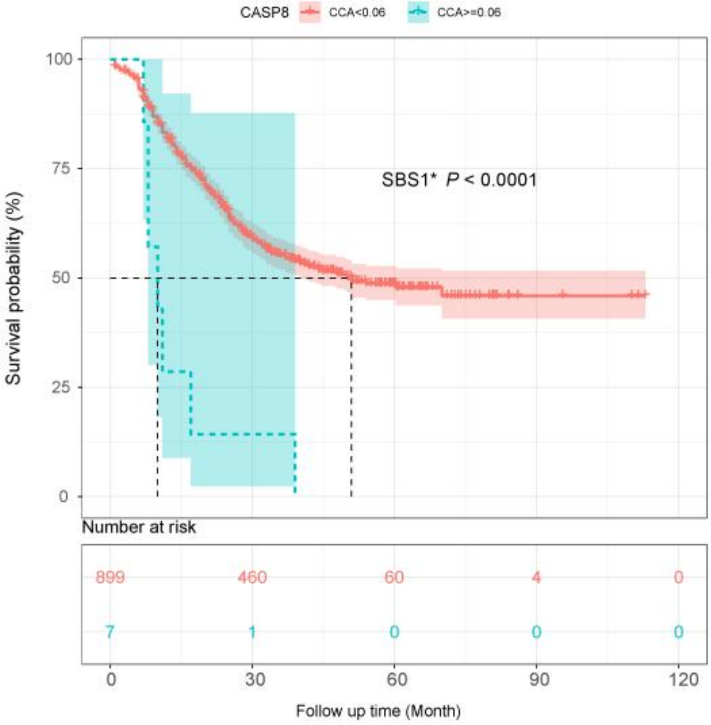
CASP8
CCA<0.06
CCA>=0.06
SBS1* P < 0.0001
Survival probability (%)
100
75
50
25
0
Number at risk
899
460
60
4
0
7
1
0
0
0
0
30
60
90
120
Follow up time (Month)

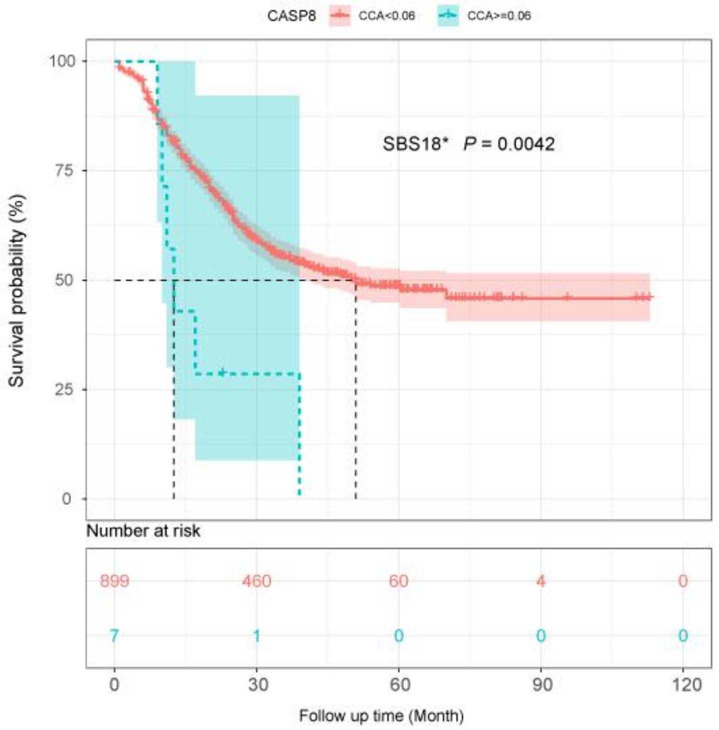

CASP8
CCA<0.06
CCA>=0.06
SBS18* P = 0.0042
Survival probability (%)
100
75
50
25
0
Number at risk
899
460
60
4
0
7
1
0
0
0
0
30
60
90
120
Follow up time (Month)

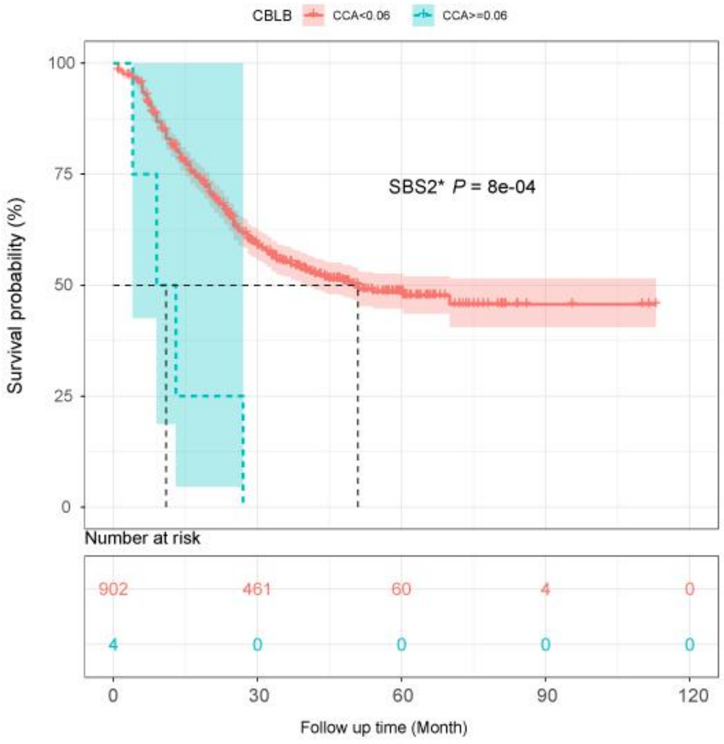

CBLB
CCA<0.06
CCA>=0.06
SBS2* P = 8e-04
Survival probability (%)
100
75
50
25
0
Number at risk
902
461
60
4
0
4
0
0
0
0
0
30
60
90
120
Follow up time (Month)

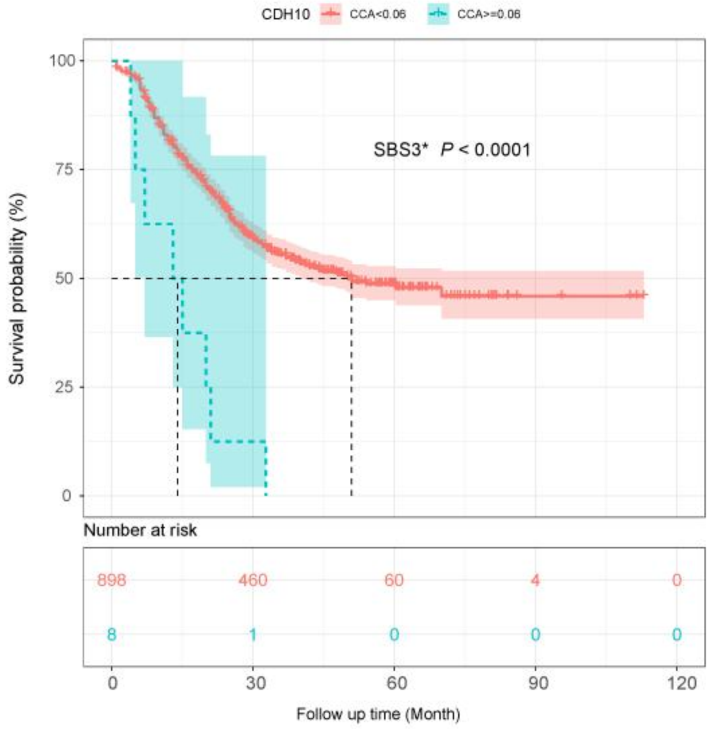

CDH10
CCA<0.06
CCA>=0.06
SBS3* P < 0.0001
Survival probability (%)
100
75
50
25
0
Number at risk
898
460
60
4
0
8
1
0
0
0
0
30
60
90
120
Follow up time (Month)

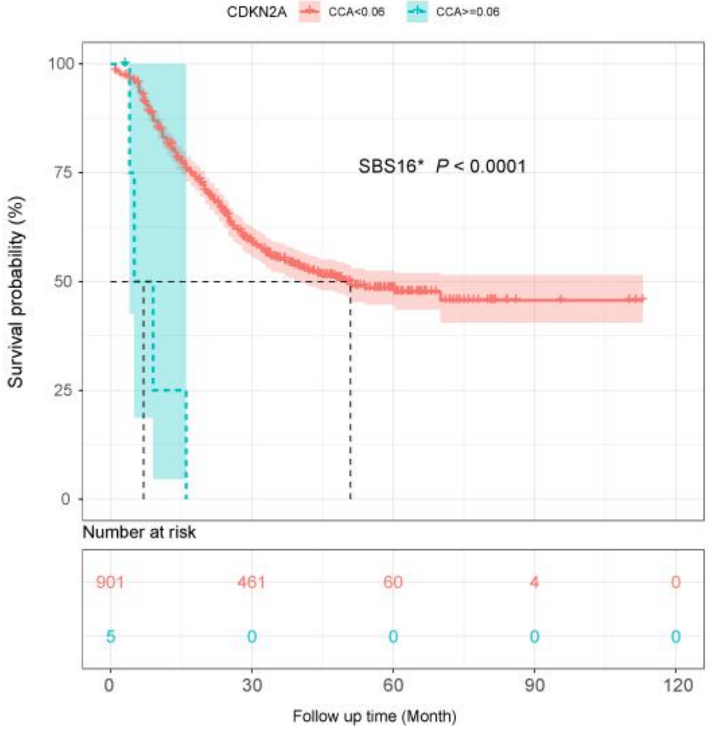

CDKN2A
CCA<0.06
CCA>=0.06
SBS16* P < 0.0001
Survival probability (%)
100
75
50
25
0
Number at risk
901
461
60
4
0
5
0
0
0
0
0
30
60
90
120
Follow up time (Month)

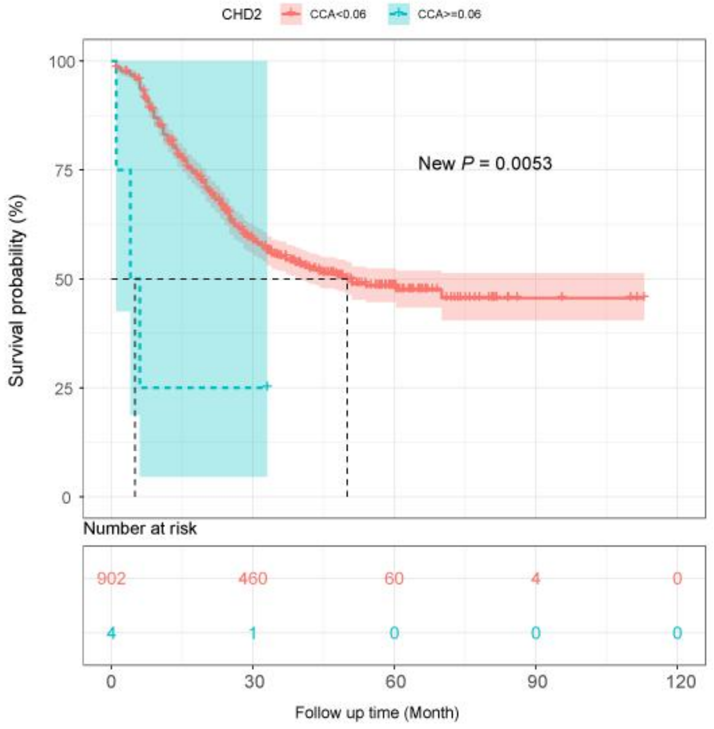
CHD2
CCA<0.06
CCA>=0.06
New P = 0.0053
Survival probability (%)
100
75
50
25
0
Number at risk
902
460
60
4
0
4
1
0
0
0
0
30
60
90
120
Follow up time (Month)

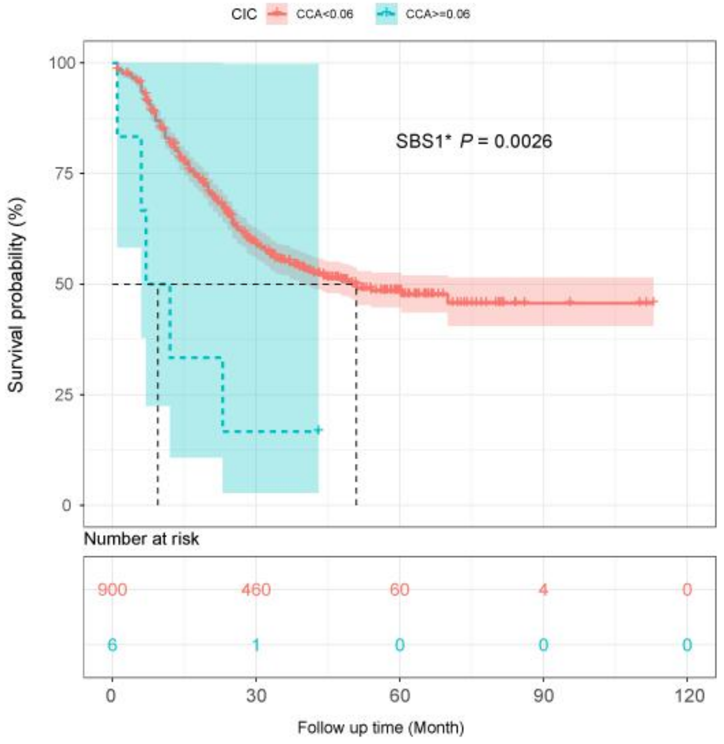
CIC
CCA<0.06
CCA>=0.06
SBS1* P = 0.0026
Survival probability (%)
100
75
50
25
0
Number at risk
900
460
60
4
0
6
1
0
0
0
0
30
60
90
120
Follow up time (Month)

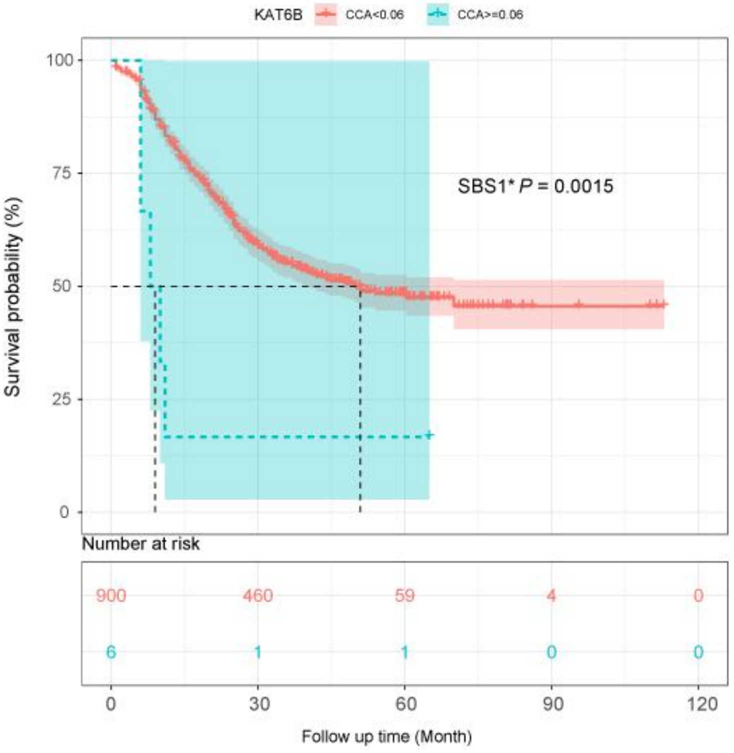
KAT6B
CCA<0.06
CCA>=0.06
SBS1* P = 0.0015
Survival probability (%)
Number at risk
900 460 59 4 0
6 1 1 0 0
Follow up time (Month)

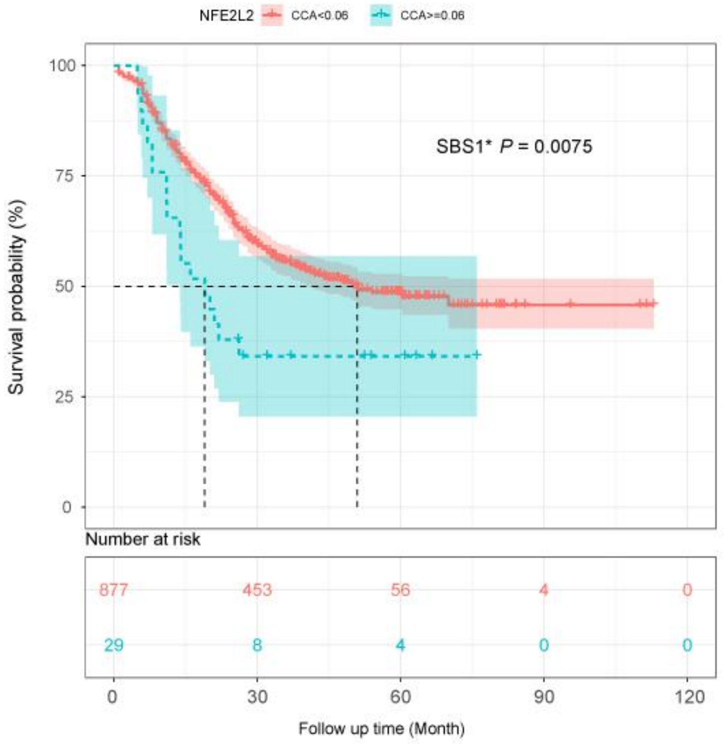
NFE2L2
CCA<0.06
CCA>=0.06
SBS1* P = 0.0075
Survival probability (%)
Number at risk
877 453 56 4 0
29 8 4 0 0
Follow up time (Month)

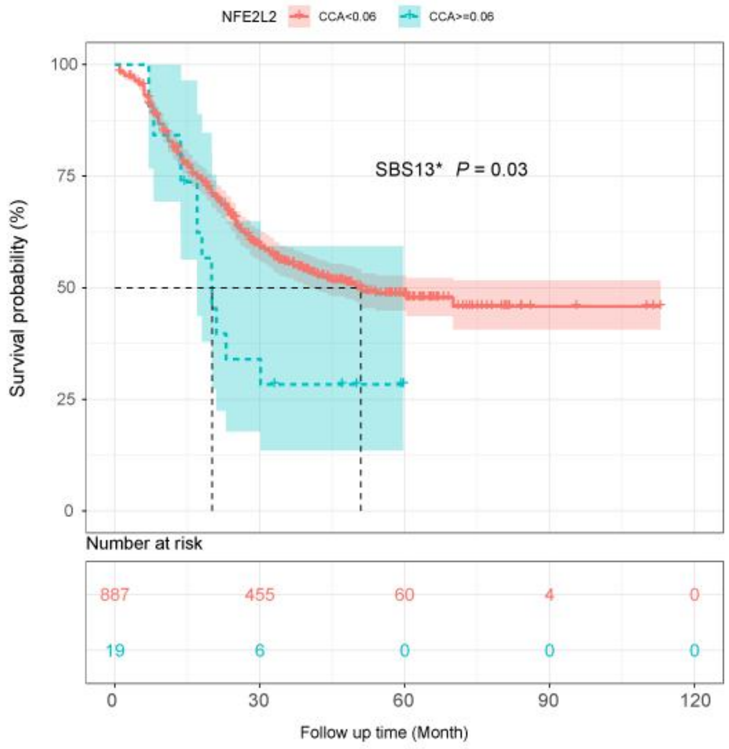
NFE2L2
CCA<0.06
CCA>=0.06
SBS13* P = 0.03
Survival probability (%)
100
75
50
25
0
Number at risk
887
455
60
4
0
19
6
0
0
0
0
30
60
90
120
Follow up time (Month)

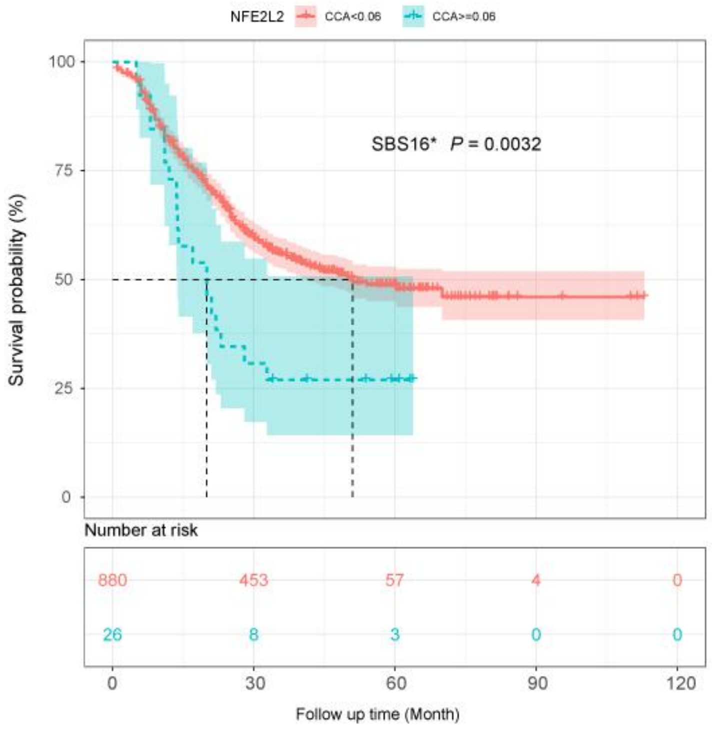
NFE2L2
CCA<0.06
CCA>=0.06
SBS16* P = 0.0032
Survival probability (%)
100
75
50
25
0
Number at risk
880
453
57
4
0
26
8
3
0
0
0
30
60
90
120
Follow up time (Month)

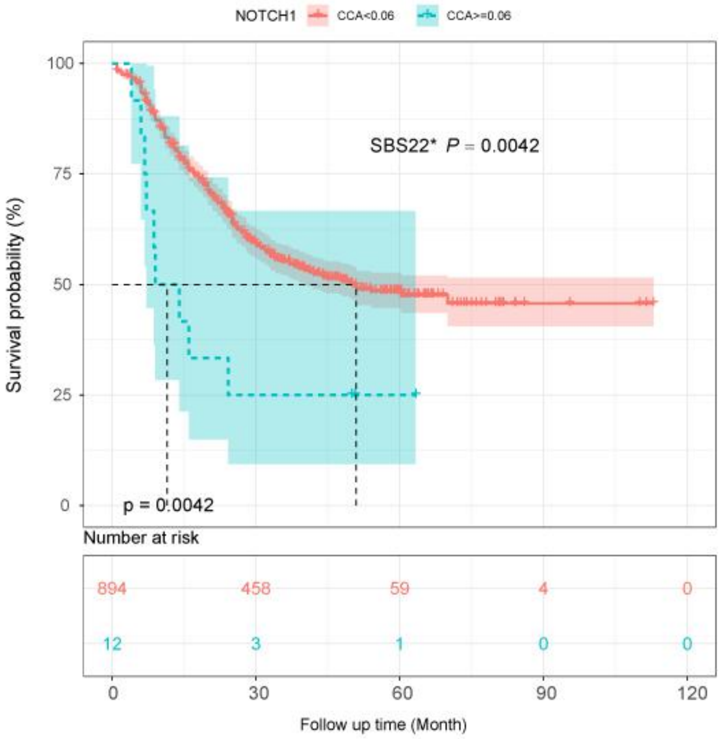
NOTCH1
CCA<0.06
CCA>=0.06
SBS22* P = 0.0042
p = 0.0042
Survival probability (%)
100
75
50
25
0
Number at risk
894
458
59
4
0
12
3
1
0
0
0
30
60
90
120
Follow up time (Month)

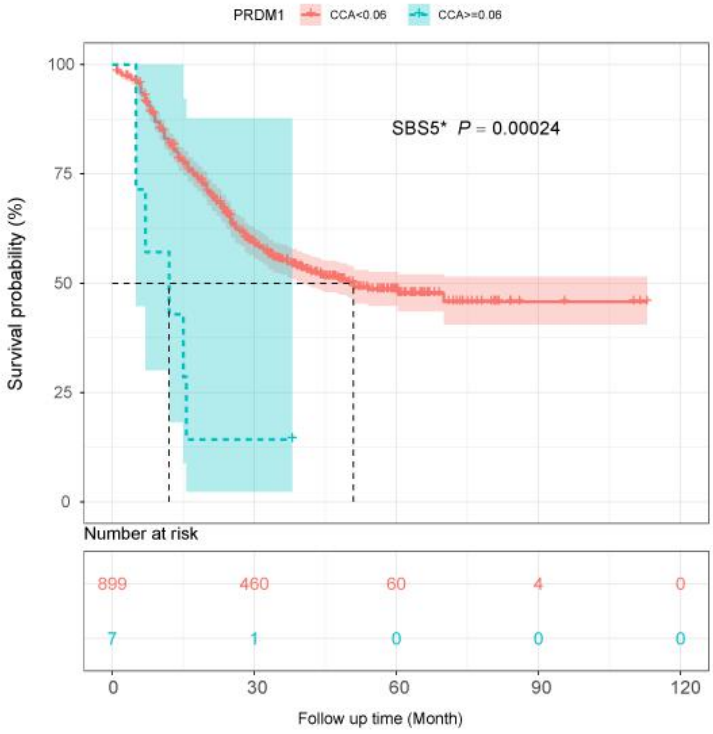
PRDM1
CCA<0.06
CCA>=0.06
SBS5* P = 0.00024
Survival probability (%)
100
75
50
25
0
Number at risk
899
460
60
4
0
7
1
0
0
0
0
30
60
90
120
Follow up time (Month)

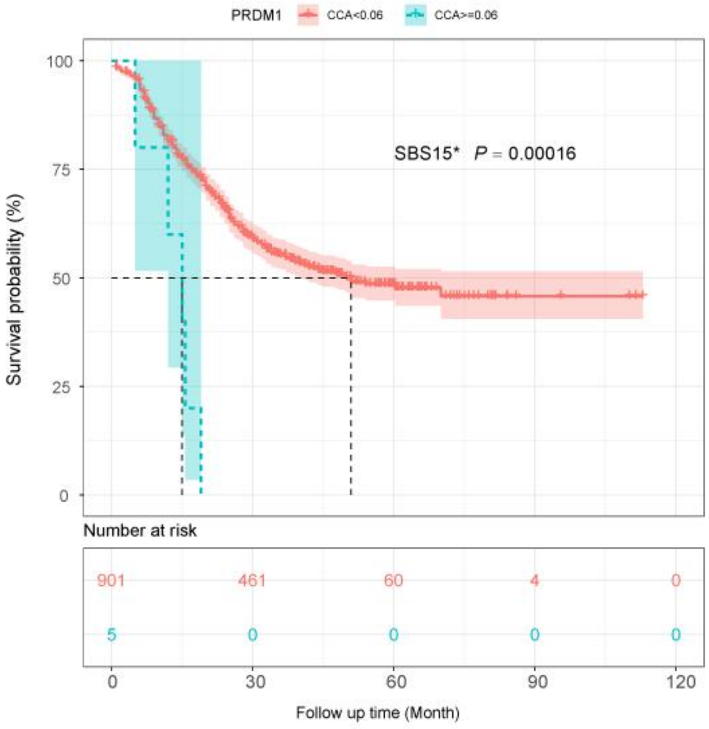
PRDM1
CCA<0.06
CCA>=0.06
SBS15* P = 0.00016
Survival probability (%)
100
75
50
25
0
Number at risk
901
461
60
4
0
5
0
0
0
0
0
30
60
90
120
Follow up time (Month)

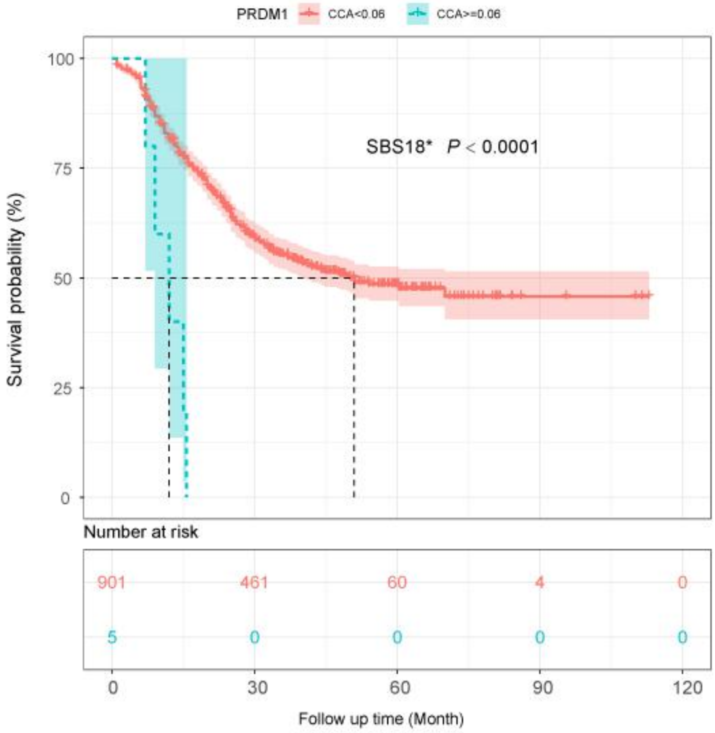
PRDM1
CCA<0.06
CCA>=0.06
SBS18* P < 0.0001
Survival probability (%)
100
75
50
25
0
Number at risk
901
461
60
4
0
5
0
0
0
0
0
30
60
90
120
Follow up time (Month)

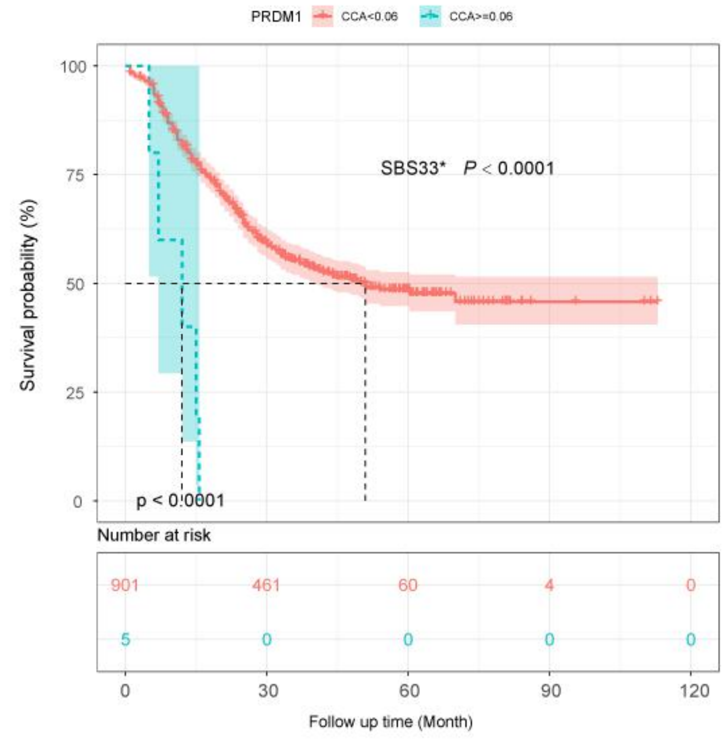
PRDM1
CCA<0.06
CCA>=0.06
100
75
50
25
0
Survival probability (%)
SBS33* $P < 0.0001$
p < 0.0001
Number at risk
901
461
60
4
0
5
0
0
0
0
0
30
60
90
120
Follow up time (Month)

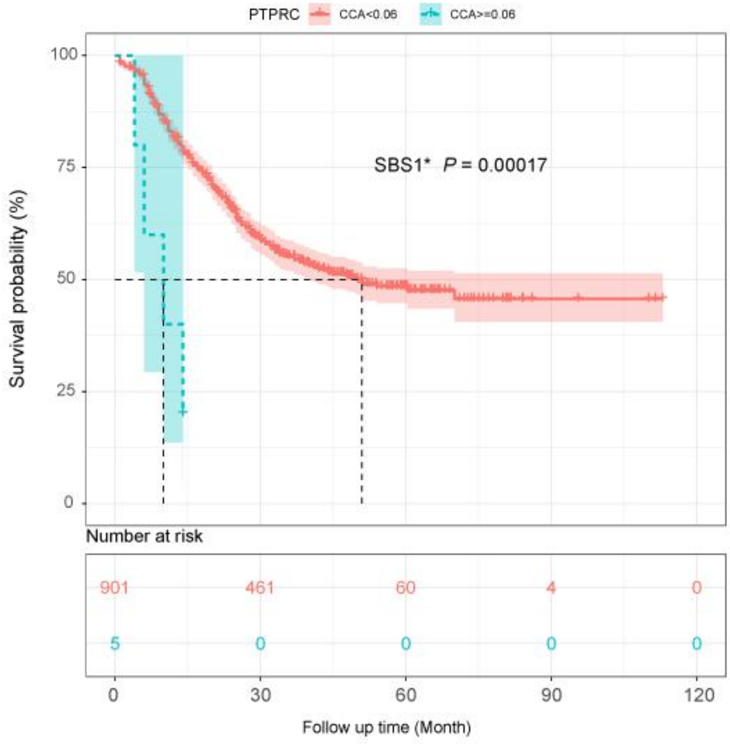
PTPRC
CCA<0.06
CCA>=0.06
100
75
50
25
0
Survival probability (%)
SBS1* $P = 0.00017$
Number at risk
901
461
60
4
0
5
0
0
0
0
0
30
60
90
120
Follow up time (Month)

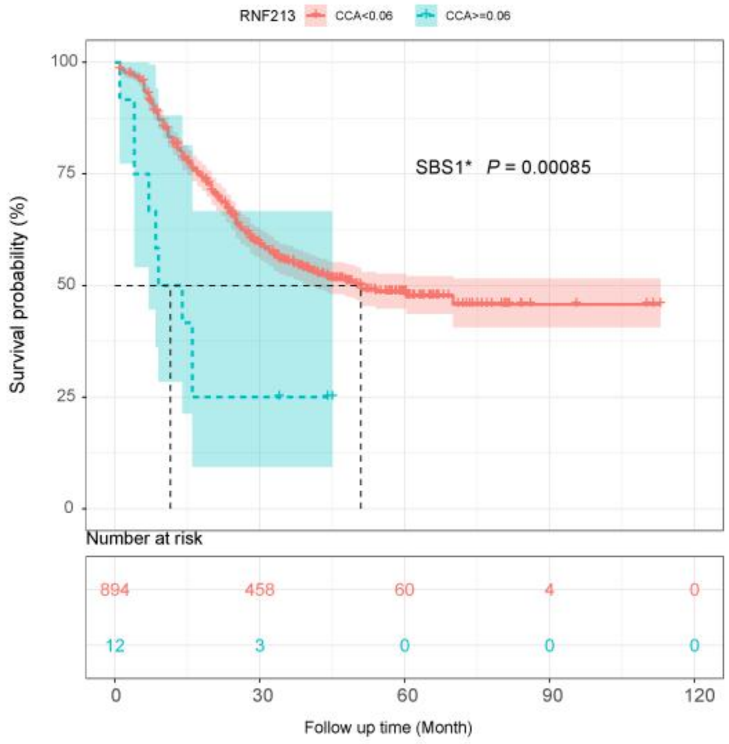

RNF213
CCA<0.06
CCA>=0.06
SBS1* P = 0.00085
Survival probability (%)
100
75
50
25
0
Number at risk
894
458
60
4
0
12
3
0
0
0
0
30
60
90
120
Follow up time (Month)

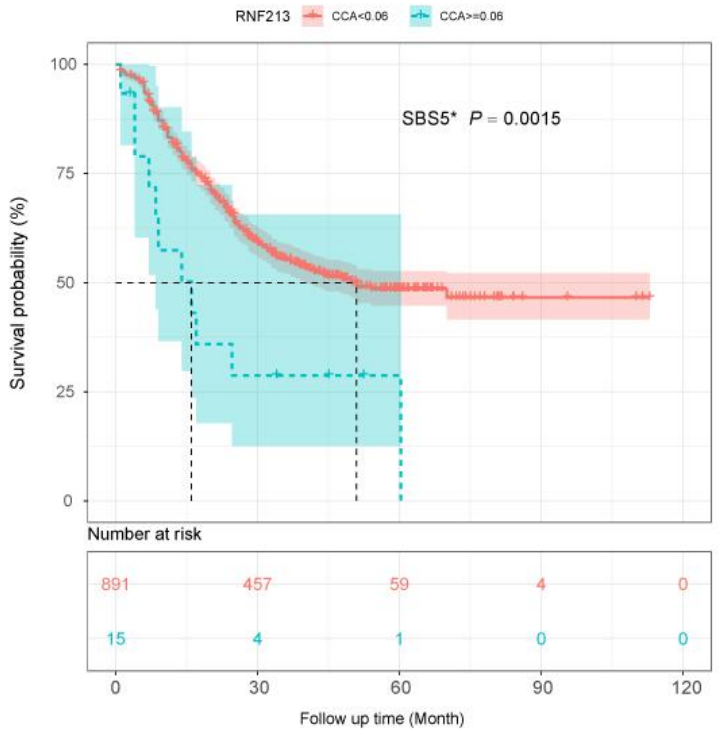

RNF213
CCA<0.06
CCA>=0.06
SBS5* P = 0.0015
Survival probability (%)
100
75
50
25
0
Number at risk
891
457
59
4
0
15
4
1
0
0
0
30
60
90
120
Follow up time (Month)

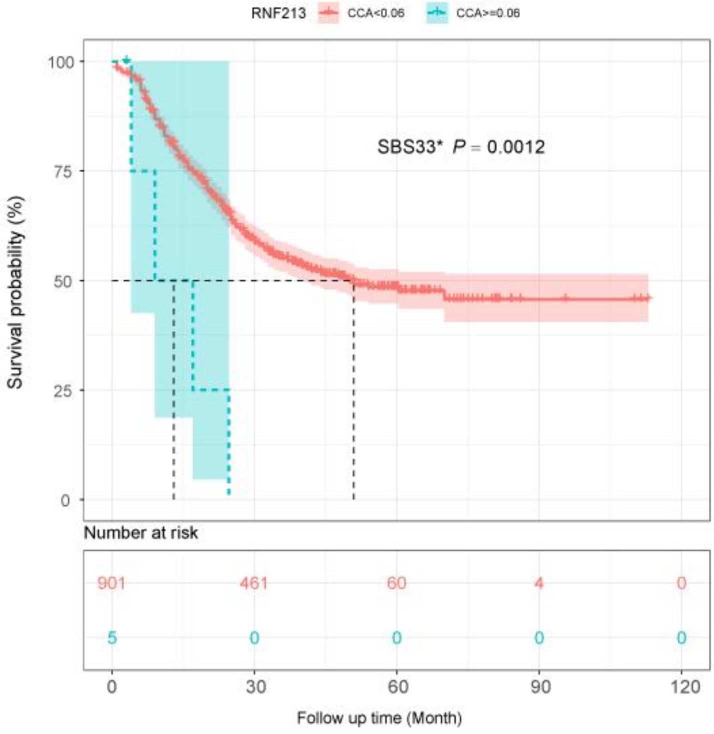
RNF213
CCA<0.06
CCA>=0.06
SBS33* P = 0.0012
Survival probability (%)
100
75
50
25
0
Number at risk
901
461
60
4
0
5
0
0
0
0
0
30
60
90
120
Follow up time (Month)

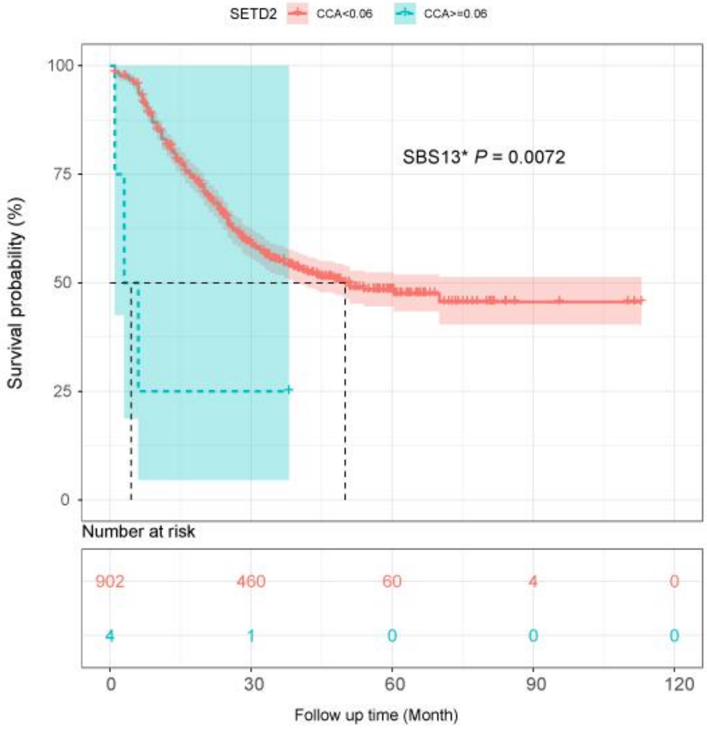
SETD2
CCA<0.06
CCA>=0.06
SBS13* P = 0.0072
Survival probability (%)
100
75
50
25
0
Number at risk
902
460
60
4
0
4
1
0
0
0
0
30
60
90
120
Follow up time (Month)

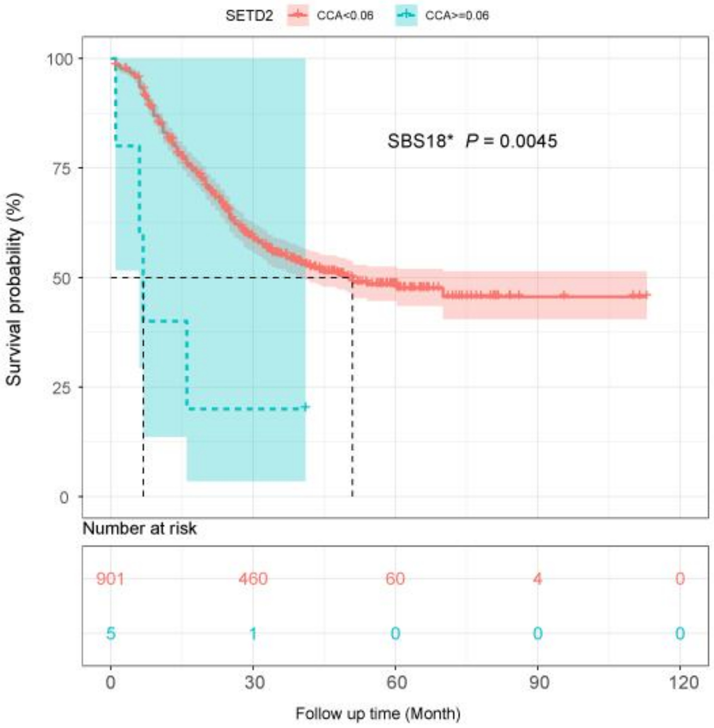

SETD2
CCA<0.06
CCA>=0.06
SBS18* P = 0.0045
Survival probability (%)
100
75
50
25
0
Number at risk
901
460
60
4
0
5
1
0
0
0
0
30
60
90
120
Follow up time (Month)

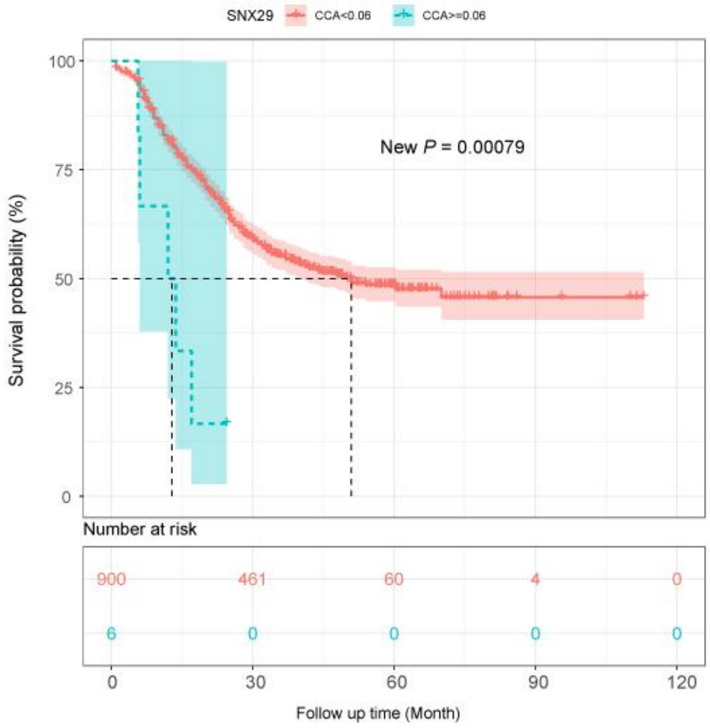

SNX29
CCA<0.06
CCA>=0.06
New P = 0.00079
Survival probability (%)
100
75
50
25
0
Number at risk
900
461
60
4
0
6
0
0
0
0
0
30
60
90
120
Follow up time (Month)

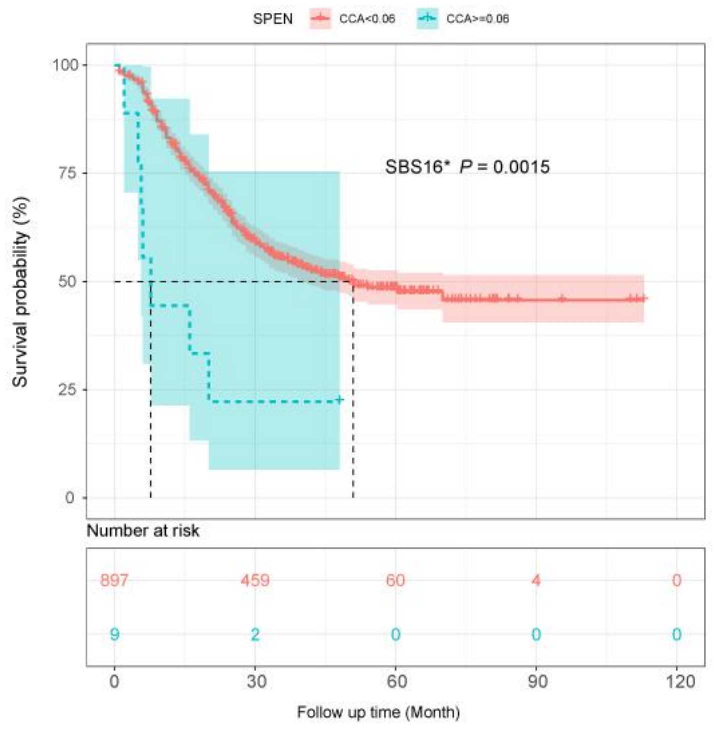
SPEN
CCA<0.06
CCA>=0.06
SBS16* P = 0.0015
Survival probability (%)
100
75
50
25
0
Number at risk
897
459
60
4
0
9
2
0
0
0
0
30
60
90
120
Follow up time (Month)

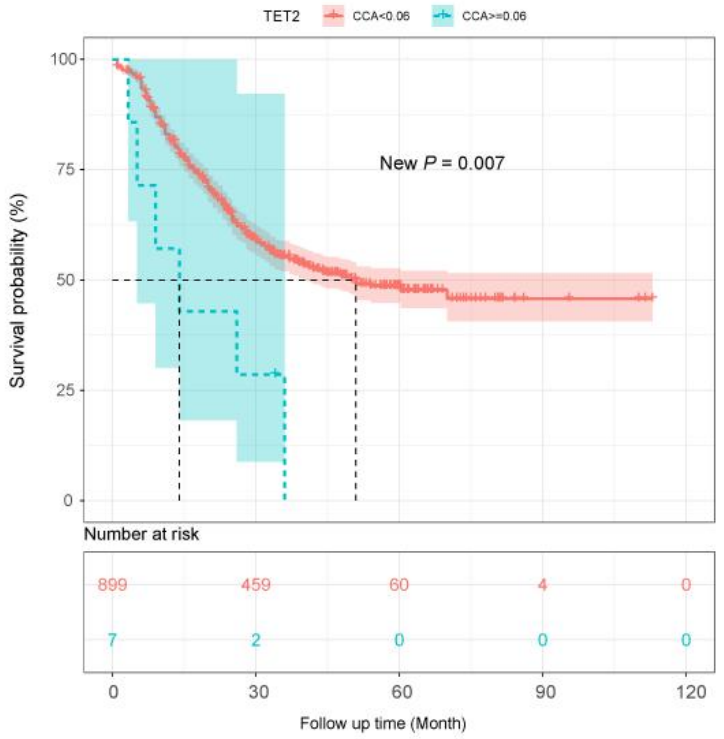
TET2
CCA<0.06
CCA>=0.06
New P = 0.007
Survival probability (%)
100
75
50
25
0
Number at risk
899
459
60
4
0
7
2
0
0
0
0
30
60
90
120
Follow up time (Month)

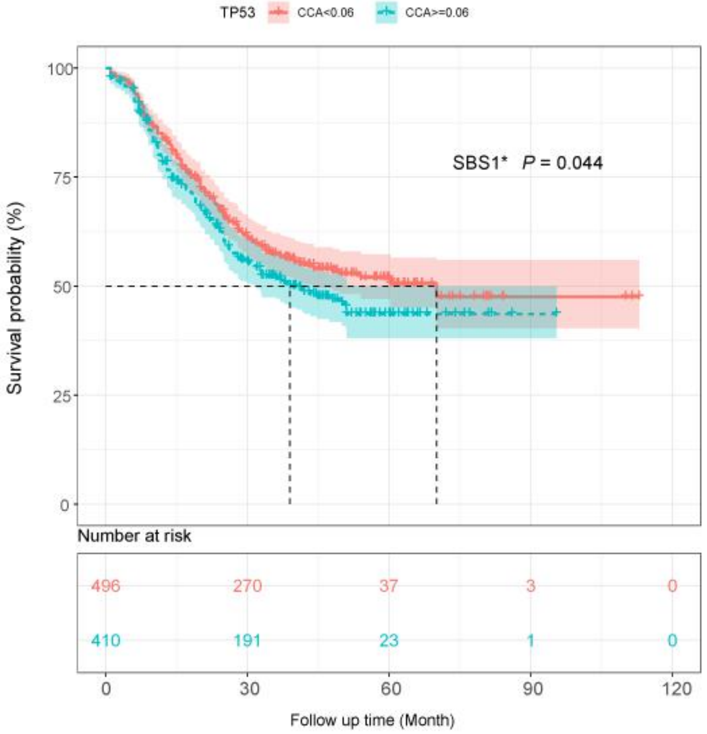
TP53
CCA<0.06
CCA>=0.06
SBS1* P = 0.044
Survival probability (%)
100
75
50
25
0
Number at risk
496 270 37 3 0
410 191 23 1 0
0 30 60 90 120
Follow up time (Month)

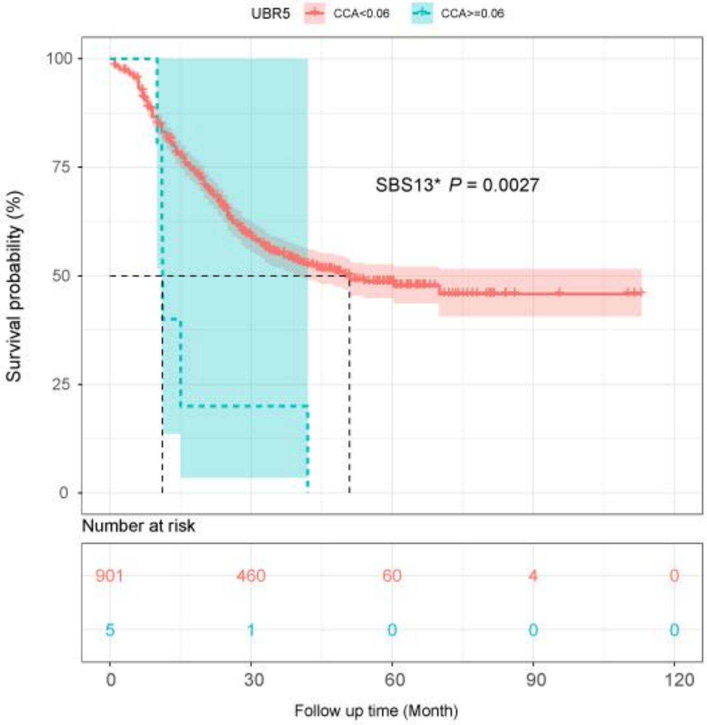
UBR5
CCA<0.06
CCA>=0.06
SBS13* P = 0.0027
Survival probability (%)
100
75
50
25
0
Number at risk
901 460 60 4 0
5 1 0 0 0
0 30 60 90 120
Follow up time (Month)

b

Multivariate Cox Analysis

SBS1* Signature

Variable	HR (95% CI)	P−value
Age	1.01 (0.997 ~ 1.03)	0.119
Gender(Male vs. Female)	1.08 (0.85 ~ 1.38)	0.517
Stage (I,II,III,IV)	2.37 (1.91 ~ 2.95)	5.23e−15
CASP8-SBS1*(CCA : >=0.06 vs. <0.06)	3.1 (1.36 ~ 7.07)	0.00727
PTPRC-SBS1*(CCA : >=0.06 vs. <0.06)	2.25 (0.662 ~ 7.65)	0.194
RNF213-SBS1*(CCA : >=0.06 vs. <0.06)	3.86 (1.91 ~ 7.82)	0.000177
KAT6B-SBS1*(CCA : >=0.06 vs. <0.06)	1.46 (0.497 ~ 4.31)	0.49
CIC-SBS1*(CCA : >=0.06 vs. <0.06)	3.08 (1.25 ~ 7.56)	0.0144
NFE2L2-SBS1*(CCA : >=0.06 vs. <0.06)	3.53 (2.01 ~ 6.18)	1.05e−05
TP53-SBS1*(CCA : >=0.06 vs. <0.06)	1.01 (0.818 ~ 1.25)	0.915

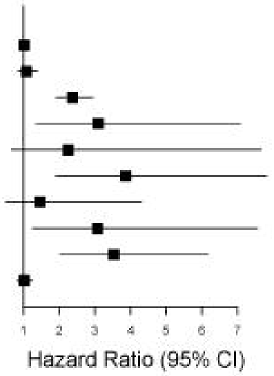

SBS2* Signature

Variable	HR (95% CI)	P−value
Age	1.01 (0.999 ~ 1.03)	0.0719
Gender(Male vs. Female)	1.07 (0.841 ~ 1.36)	0.579
Stage (I,II,III,IV)	2.39 (1.93 ~ 2.97)	2.54e−15
CBL-SBS2*(CCA : >=0.06 vs. <0.06)	3.08 (1.14 ~ 8.31)	0.0264
PIK3CA-SBS2*(CCA : >=0.06 vs. <0.06)	0.958 (0.608 ~ 1.51)	0.852

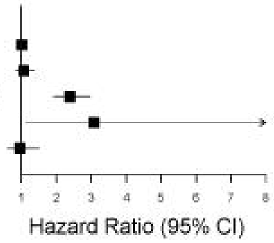

SBS3* Signature

Variable	HR (95% CI)	P−value
Age	1.01 (0.998 ~ 1.03)	0.098
Gender(Male vs. Female)	1.06 (0.832 ~ 1.35)	0.642
Stage (I,II,III,IV)	2.39 (1.93 ~ 2.97)	2.09e−15
CDH10-SBS3*(CCA : >=0.06 vs. <0.06)	4.16 (1.96 ~ 8.87)	0.000217

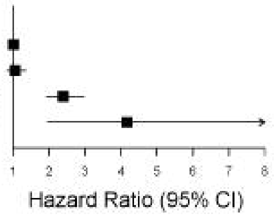

New Signature

Variable	HR (95% CI)	P−value
Age	1.01 (0.998 ~ 1.03)	0.092
Gender(Male vs. Female)	1.09 (0.853 ~ 1.39)	0.5
Stage (I,II,III,IV)	2.42 (1.95 ~ 3.01)	9.67e−16
SNX29-New(CCA : >=0.06 vs. <0.06)	5.16 (1.63 ~ 16.4)	0.00529
CHD2-New(CCA : >=0.06 vs. <0.06)	5.18 (1.64 ~ 16.3)	0.00502
TET2-New(CCA : >=0.06 vs. <0.06)	1.46 (0.544 ~ 3.94)	0.45

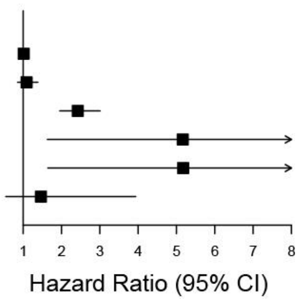

SBS5* Signature

Variable	HR (95% CI)	P−value
Age	1.01 (0.998 ~ 1.03)	0.0909
Gender(Male vs. Female)	1.07 (0.844 ~ 1.37)	0.561
Stage (I,II,III,IV)	2.4 (1.94 ~ 2.98)	1.49e−15
PRDM1-SBS5*(CCA : >=0.06 vs. <0.06)	3.92 (1.74 ~ 8.85)	0.000984
RNF213-SBS5*(CCA : >=0.06 vs. <0.06)	4.68 (2.31 ~ 9.5)	1.87e−05

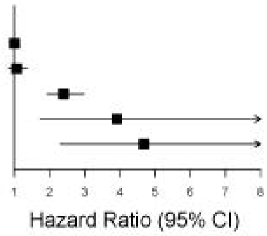

SBS13* Signature

Variable	HR (95% CI)	P−value
Age	1.01 (0.996 ~ 1.02)	0.178
Gender(Male vs. Female)	1.06 (0.835 ~ 1.36)	0.613
Stage (I,II,III,IV)	2.46 (1.98 ~ 3.06)	4.79e−16
UBR5-SBS13*(CCA : >=0.06 vs. <0.06)	2.57 (0.951 ~ 6.92)	0.0627
ARHGAP5-SBS13*(CCA : >=0.06 vs. <0.06)	2.33 (1.03 ~ 5.27)	0.042
BRCA1-SBS13*(CCA : >=0.06 vs. <0.06)	3.13 (1.39 ~ 7.05)	0.00603
SETD2-SBS13*(CCA : >=0.06 vs. <0.06)	5.67 (1.77 ~ 18.2)	0.00345
PIK3CA-SBS13*(CCA : >=0.06 vs. <0.06)	0.806 (0.479 ~ 1.35)	0.415

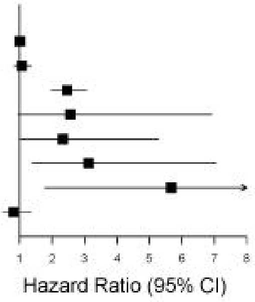

SBS15* Signature

Variable	HR (95% CI)	P−value
Age	1.01 (0.998 ~ 1.03)	0.108
Gender(Male vs. Female)	1.06 (0.833 ~ 1.35)	0.635
Stage (I,II,III,IV)	2.42 (1.95 ~ 3)	8.77e−16
PRDM1-SBS15*(CCA : >=0.06 vs. <0.06)	4.14 (1.69 ~ 10.1)	0.00182

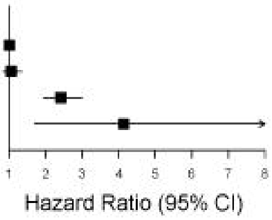

SBS16* Signature

Variable	HR (95% CI)	P−value
Age	1.01 (1 ~ 1.03)	0.0536
Gender(Male vs. Female)	1.06 (0.836 ~ 1.35)	0.614
Stage (I,II,III,IV)	2.39 (1.93 ~ 2.97)	2.3e−15
CDKN2A-SBS16*(CCA : >=0.06 vs. <0.06)	4.63 (1.68 ~ 12.7)	0.00299
SPEN-SBS16*(CCA : >=0.06 vs. <0.06)	2.35 (0.961 ~ 5.76)	0.0611
NFE2L2-SBS16*(CCA : >=0.06 vs. <0.06)	3.18 (1.82 ~ 5.56)	5.08e−05

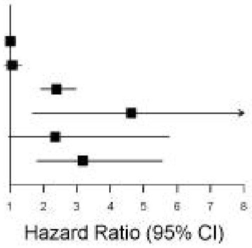

SBS18* Signature

Variable	HR (95% CI)	P−value
Age	1.01 (0.998 ~ 1.03)	0.101
Gender(Male vs. Female)	1.07 (0.843 ~ 1.36)	0.571
Stage (I,II,III,IV)	2.38 (1.92 ~ 2.95)	3.51e−15
PRDM1-SBS18*(CCA : >=0.06 vs. <0.06)	3.42 (1.35 ~ 8.67)	0.00942
CASP8-SBS18*(CCA : >=0.06 vs. <0.06)	2.1 (0.834 ~ 5.26)	0.115
SETD2-SBS18*(CCA : >=0.06 vs. <0.06)	2.39 (0.763 ~ 7.45)	0.135

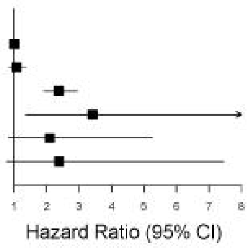

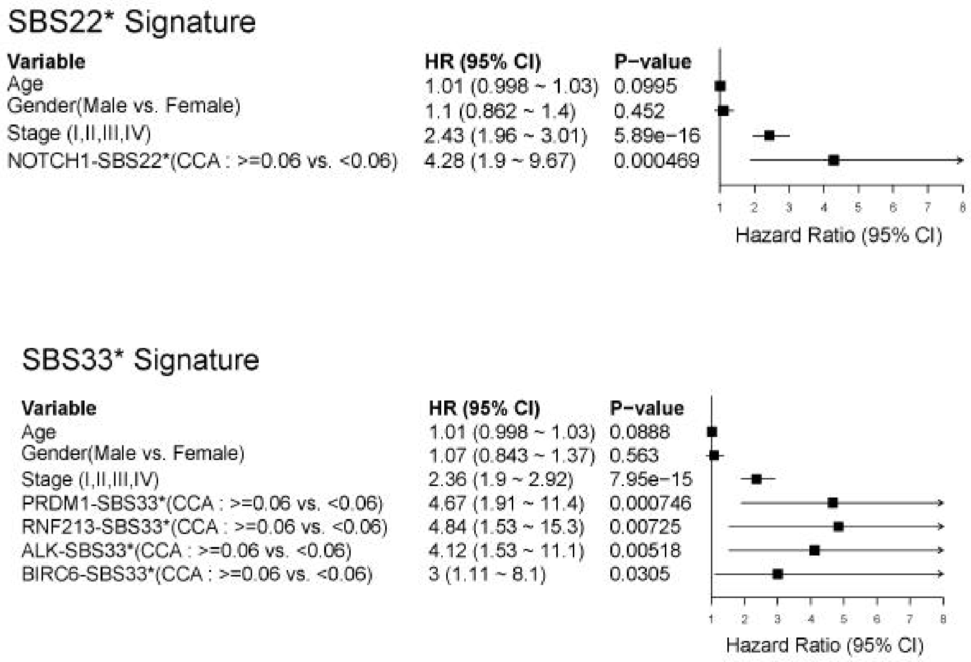

Supplementary Figure 7. (a) Association of CCA of cancer-related genes from the COSMIC census assigned to SBS signatures with prognosis. Kaplan-Meier survival analysis classified by the status that CCA of genes assigned to SBS signatures with a threshold 6%. (b) Multivariate Cox regression analysis of CCA of genes assigned to SBS signatures with age, gender, stage and CCA of genes assigned to SBS signatures. Red is marked with significant items.
