# Supplementary material for: A practical framework RNMF for exploring the association between mutational signatures and genes using gene cumulative contribution abundance
Source: Cancer Med. 2022 May 16;11(21):4053–69. doi: 10.1002/cam4.4717 (PMC9636515; doi:10.1002/cam4.4717)
Supplement: Supplementary file 8 — Figure S8 [file CAM4-11-4053-s012.pdf]

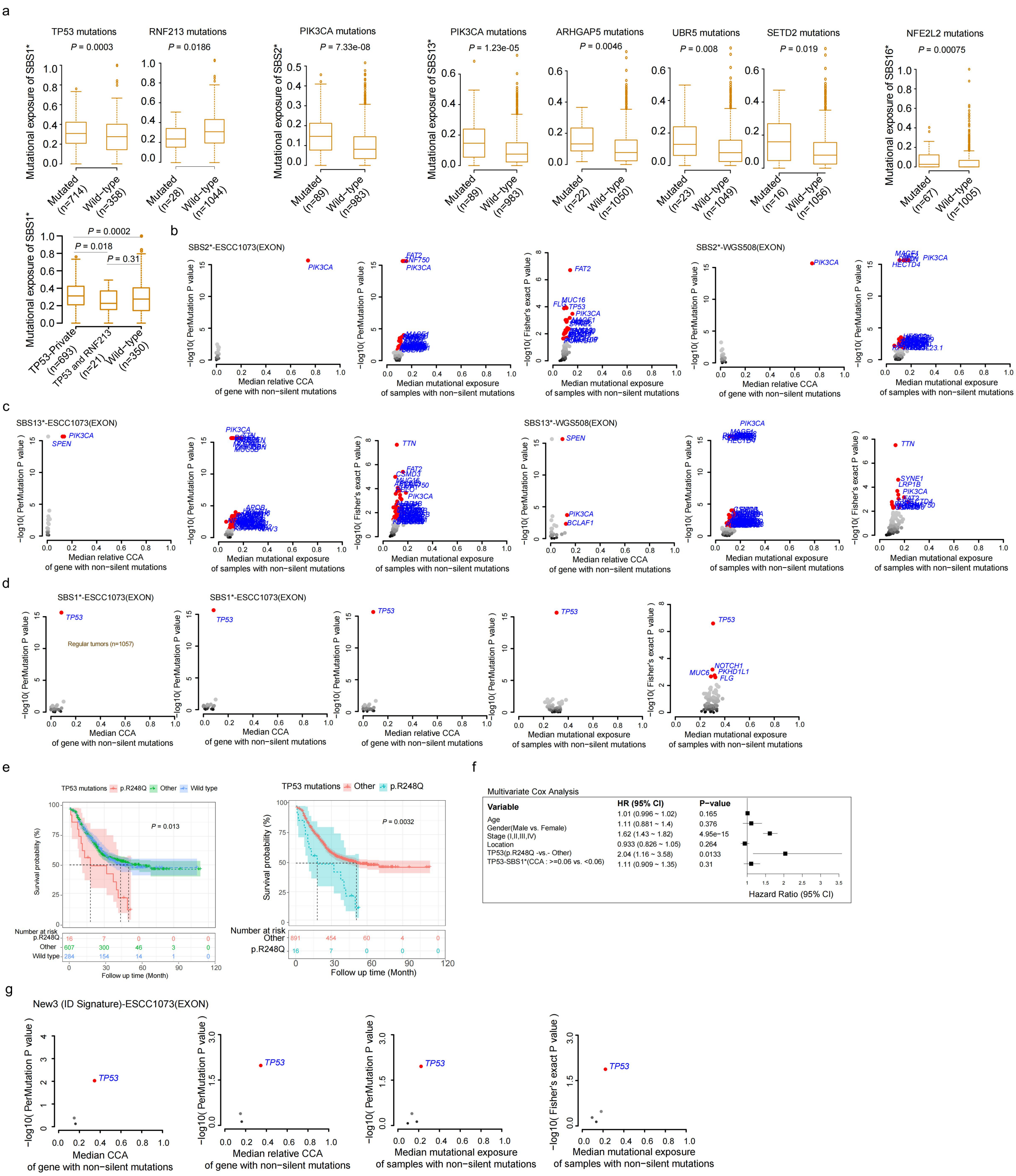

Supplementary Figure 8. (a) Box plot showing that the SBS signatures were associated with cancer-related genes mutations (only SNV), where n represents the number of samples. (b-d) CCA enrichment analysis and mutational signature enrichment analyses identify an association between somatic mutations and activity of signatures in ESCC. Here, we use two datasets: exon regions of 1073 and 508 ESCC cases. First, the median CCA of each gene in the current mutational signature is calculated, and then the contribution importance of each gene is calculated by PERMUTATION test or Fisher's test to study the association between gene and feature. The regular tumors in the figure represent the samples with non hypermutated. For genes mutated in >5% of samples, the CCA of genes attributed to SBS or ID signatures was compared in tumors with wild-type versus mutated copies of the gene. Genes with FDR q < 0.1 are highlighted in red. (e) Association of *TP53* mutations with prognosis. Kaplan-Meier survival analysis classified by the status that *TP53* hotspot mutation (p.R248Q). (f) Multivariate Cox regression analysis of *TP53* CCA assigned to SBS1 with age, gender, stage, Location, *TP53* hotspot mutation p.R248Q status and CCA of *TP53* assigned to SBS1*. (g) CCA enrichment analysis and mutational signature enrichment analyses identify an association between somatic mutations and activity of signatures in ESCC. First, the median CCA of each gene in the current mutational signature is calculated, and then the contribution importance of each gene is calculated by PERMUTATION test or Fisher's test to study the association between gene and feature. The regular tumors in the figure represent the samples with non hypermutated. For genes mutated in >5% of samples, the CCA of genes attributed to SBS or ID signatures was compared in tumors with wild-type versus mutated copies of the gene. Genes with FDR q < 0.1 are highlighted in red.
