# Supplementary material for: A practical framework RNMF for exploring the association between mutational signatures and genes using gene cumulative contribution abundance
Source: Cancer Med. 2022 May 16;11(21):4053–69. doi: 10.1002/cam4.4717 (PMC9636515; doi:10.1002/cam4.4717)
Supplement: Supplementary file 9 — Figure S9 [file CAM4-11-4053-s003.pdf]

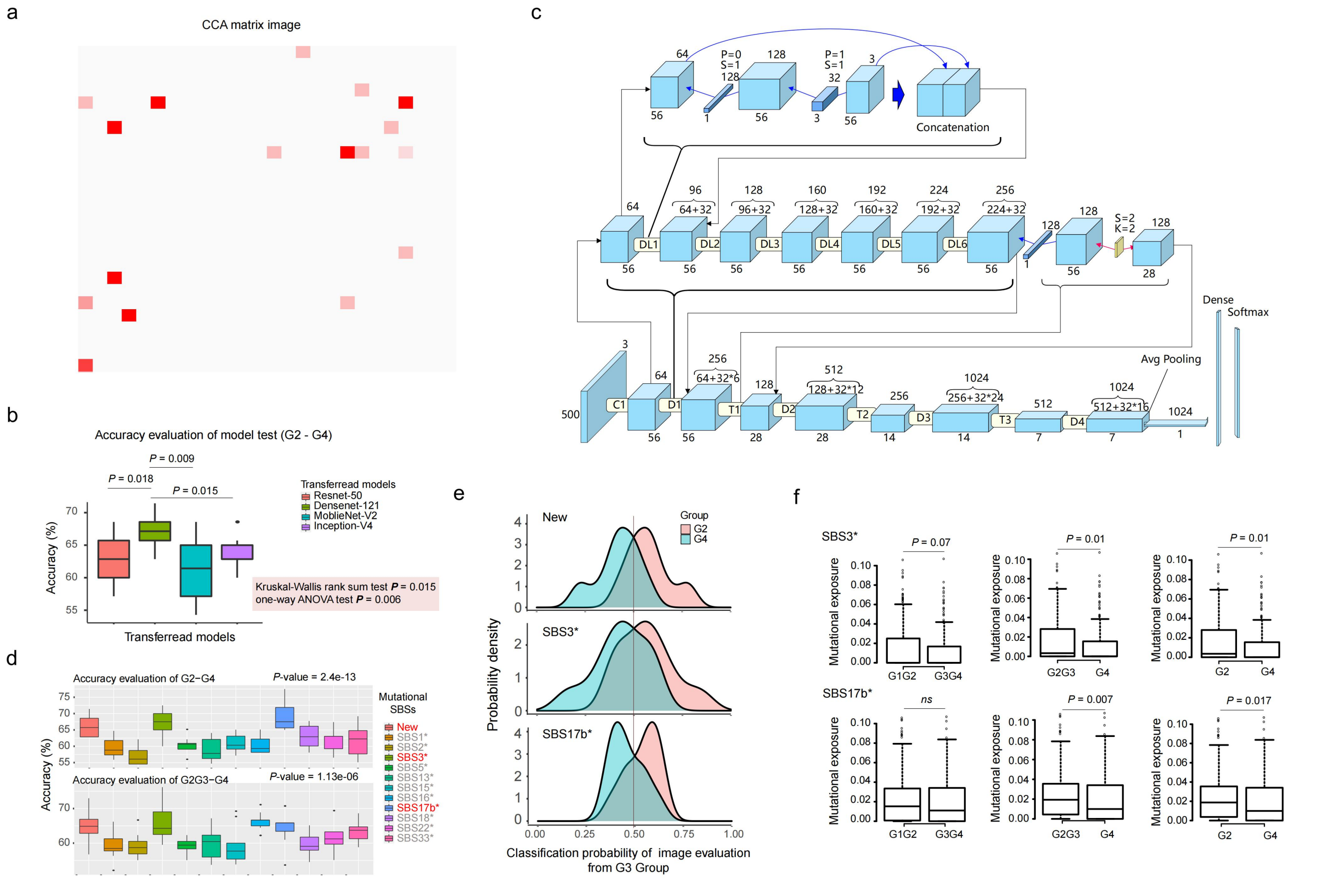

Supplementary Figure 9. The application and association of CCA matrix of genes. (a) The example of CCA matrix image of genes. (b) Accuracy evaluation of model test between G2 group and G4 group. Wilcoxon rank sum test with two-sided was used for statistical significance. (c) Full schematic representation of DenseNet-121. (d) The model DenseNet-121 was trained 10 times for each sub-feature data of G2-G4 and G2G3-G4. For each result, we randomly select 90% of the samples as training, and the remaining 10% as test data set for analysis. Each box represents a mutational signature and is marked in a different color. One way ANOVA test was used for statistical significance. The higher accuracy level of mutational signature is marked in red. (e) The distribution of G3 groups in the parameter model obtained by G2-G4. A color represents a group, a curve represents a density curve. (f) The difference of sample contribution in different groups was compared. Wilcoxon rank sum test with two-sided was used for statistical significance.
