# Supplementary material for: A practical framework RNMF for exploring the association between mutational signatures and genes using gene cumulative contribution abundance
Source: Cancer Med. 2022 May 16;11(21):4053–69. doi: 10.1002/cam4.4717 (PMC9636515; doi:10.1002/cam4.4717)
Supplement: Supplementary file 10 — Table S1 [file CAM4-11-4053-s005.pdf]

Table S1. The paper catalogue of data acquisition.

Code	Title
1	Whole-genome sequencing of 508 patients identifies key molecular features associated with poor prognosis in esophageal squamous cell carcinoma
2	Genomic Analyses Reveal Mutational Signatures and Frequently Altered Genes in Esophageal Squamous Cell Carcinoma
3	Genomic Landscape of Esophageal Squamous Cell Carcinoma in a Japanese Population
4	Identification of genomic alterations in oesophageal squamous cell cancer
5	Genomic comparison of esophageal squamous cell carcinoma and its precursor lesions by multi-region whole-exome sequencing
6	Genomic and molecular characterization of esophageal squamous cell carcinoma
