# Supplementary material for: A practical framework RNMF for exploring the association between mutational signatures and genes using gene cumulative contribution abundance
Source: Cancer Med. 2022 May 16;11(21):4053–69. doi: 10.1002/cam4.4717 (PMC9636515; doi:10.1002/cam4.4717)
Supplement: Supplementary file 11 — Table S2 [file CAM4-11-4053-s011.pdf]

Table S2a. Mutational signature matrix of single-base substitutions (SBS).

Subtype	SBS1	SBS2	SBS3	SBS4	SBS5	SBS6	SBS7	SBS8	SBS9	SBS10	SBS11	SBS12
A[C>A]A	0.021667264	0.019713876	0.051430071	0.032599851	0.001293938	2.79E-25	0.002643495	0.016933534	0.010197277	2.14E-18	0.000435758	3.11E-79
A[C>A]C	0.018081045	0.009998501	0.020031468	0.006386546	0.006791404	1.41E-33	0.002970577	0.009257624	0.006881389	1.04E-25	0.002161167	3.76E-09
A[C>A]G	0.001479892	0.003638271	0.00313254	0.002484592	0.00219721	0.000105866	3.29E-17	0.004932795	0.001126625	8.17E-197	6.69E-05	2.65E-262
A[C>A]T	0.018241197	0.009927579	0.028889582	0.014902568	0.002639348	0.000383521	0.001404816	0.007644189	0.002490705	0.005518446	0.001585796	0.001316673
C[C>A]A	0.014103313	1.55E-10	0.066442136	0.073815625	0.003201661	0.00164765	1.83E-40	0.014136583	0.00057093	0.010109458	0.000805903	0.00712092
C[C>A]C	0.016846712	0.003562849	0.020203291	0.00783044	0.007801125	0.001330017	0.00163308	0.008050985	0.001192643	0.001172429	0.004241609	0.013048146
C[C>A]G	0.001341281	0.00338041	0.007023917	0.001700051	0.001488761	0.000555092	4.79E-12	0.003652801	0.000642337	0.000863136	0.000473935	0.002635609
C[C>A]T	0.013777105	1.85E-28	0.040591415	0.007250467	0.01147083	0.000480387	4.24E-30	0.008754027	9.00E-33	0.012064825	0.003299378	0.049148284
G[C>A]A	8.11E-17	0.01145155	0.094605496	0.023201849	2.90E-68	3.61E-17	0.000338915	0.008329425	0.003953093	0.015487023	0.001752988	3.76E-08
G[C>A]C	0.00896147	0.010157165	0.019048576	0.011857452	0.004868166	3.52E-08	0.001443008	0.006888262	0.001452751	0.002508583	0.000975531	0.003485091
G[C>A]G	0.000219854	0.002048153	0.005310877	0.001913496	0.001102302	3.73E-45	0.000234193	0.004508666	0.002864049	8.17E-112	1.31E-32	0.000408653
G[C>A]T	0.005028693	0.008989957	0.051560727	0.008067769	0.001879907	5.33E-18	4.86E-33	0.004755793	0.001090172	0.012071094	0.001397022	0.016382079
T[C>A]A	0.006848708	0.030422354	0.070847784	0.045200183	0.002805571	0.035933938	0.003427943	0.007964107	0.005478658	0.006099967	2.74E-12	7.25E-81
T[C>A]C	0.015282923	0.017829305	0.041221388	0.008570337	0.002477226	0.015318333	0.001721516	0.006659283	0.011084646	0.00131702	0.002288403	4.74E-30
T[C>A]G	1.06E-13	0.005422732	0.007107237	0.001268841	0.001049209	0.003950966	0.00093091	0.002580894	0.002594987	0.001210459	0.000102468	1.17E-153
T[C>A]T	0.009719261	0.044084505	0.118517759	0.000183889	0.001631977	0.014717386	0.004549736	3.50E-30	0.024619538	0.027625018	3.41E-05	0.013933203
A[C>G]A	0.025145114	1.51E-06	0.008397879	0.002858894	0.006006956	0.006318161	5.85E-32	0.013360707	0.006767798	0.0002756	0.000917262	8.73E-106
A[C>G]C	0.015200836	1.37E-28	0.008297762	0.000346076	0.003452539	0.00182942	4.74E-36	0.005067845	0.00532148	0.0004742	0.000250107	3.60E-41
A[C>G]G	0.006276748	1.89E-17	0.001473325	0.001060716	0.001534928	0.000649431	2.24E-14	0.004222037	2.87E-47	6.67E-05	0.000605687	4.71E-231
A[C>G]T	0.026473864	0.020348599	0.006388556	0.000231622	0.001354953	0.004532916	1.16E-41	0.006493352	0.001396619	0.00187745	6.47E-125	0.000541709
C[C>G]A	0.016376302	0.002156711	0.004053149	0.000156734	0.002426386	0.007308081	1.72E-17	0.004948959	0.000907537	4.57E-26	0.001625004	4.91E-111
C[C>G]C	0.013233782	0.003302962	0.003456521	2.51E-161	0.002509723	0.001651522	0.000716548	0.004365509	4.07E-08	3.88E-42	0.001829918	7.56E-60
C[C>G]G	0.004037153	0.001798716	0.001489576	0.000730881	0.00139676	0.000819833	5.03E-05	0.003342269	1.77E-24	1.71E-96	0.000213916	7.84E-165
C[C>G]T	0.019553488	0.017527493	0.004034975	1.73E-305	0.002396805	0.007191087	1.30E-71	0.003381178	1.74E-42	5.38E-44	0.000741658	3.65E-54
G[C>G]A	0.011208565	2.38E-49	0.003536792	0.002557424	0.003372876	0.003165099	9.20E-30	0.006810372	0.000866769	1.47E-06	0.000526257	8.26E-114
G[C>G]C	0.010319151	9.55E-15	0.003263474	0.001448563	0.003495571	0.001338244	4.37E-31	0.005614721	0.00154013	0.002377207	0.000577159	0.00032494
G[C>G]G	0.000909642	6.82E-89	1.30E-139	1.30E-100	0.002389711	0.000352647	2.70E-93	0.005995709	0.000158118	2.8705214023	0.000434688	1.5563067843

										3764e-321		9993e-321
G[C>G]T	0.01607021	0.004441245	0.002993544	0.001325848	0.001511548	0.004728667	7.35E-33	0.006077036	1.81E-17	5.92E-57	0.000473886	2.69E-34
T[C>G]A	1.18E-07	2.89E-10	9.38E-12	0.00486997	9.20E-12	0.3432284	5.44E-08	0.010739767	0.000793169	0.034742273	0.010114036	0.002949368
T[C>G]C	0.021012813	0.000367644	0.004814489	0.001486881	0.00233673	0.063478362	1.82E-17	0.007261952	9.27E-11	1.06E-14	0.003048111	4.22E-18
T[C>G]G	0.001279932	4.28E-08	0.000841013	0.001087226	0.00096945	0.021722456	1.21E-14	0.003026003	2.49E-10	0.001201998	0.000565309	6.77E-40
T[C>G]T	0.014966178	0.114155448	0.000762311	1.14E-09	4.71E-11	0.329906216	0.020197221	1.63E-11	5.81E-09	1.18E-08	5.54E-10	6.99E-41
A[C>T]A	0.009313375	3.60E-05	0.016077094	0.02618651	0.018853145	5.96E-05	0.010920543	0.050873827	0.019128881	9.64E-06	0.001208402	0.030455815
A[C>T]C	0.007063443	0.00557337	0.009129335	0.018847632	0.007062033	8.23E-35	0.00337966	0.019368313	0.014098409	0.002777965	0.000550334	0.008277434
A[C>T]G	0.014871708	3.84E-07	0.01253424	2.34E-05	0.006686715	3.97E-08	2.25E-16	0.027278654	0.198996917	0.007320626	3.63E-06	0.042733416
A[C>T]T	0.013485432	0.00957885	0.014375431	0.020383832	0.004008578	1.41E-68	0.005622923	0.025978902	0.01832632	0.004043448	5.27E-06	0.002782227
C[C>T]A	0.011434923	2.77E-10	0.016433264	0.029215398	0.010195266	0.000984699	0.020883391	0.03301713	0.01724703	0.001150585	0.00649469	1.95E-27
C[C>T]C	0.006977567	0.002201638	0.01164419	0.021619771	0.005499208	3.56E-53	0.005645006	0.0251742	0.02076467	0.003385068	0.004895424	0.005524769
C[C>T]G	0.001643533	0.001518059	0.005472073	0.016414512	0.019922311	0.001490086	0.002447738	0.028426242	0.132589999	0.008389369	0.001764295	0.025816127
C[C>T]T	0.012222878	0.017850137	0.015465951	0.027138554	0.002169009	6.18E-23	0.01142964	0.027662989	0.019615496	0.002483487	0.002391874	0.002345758
G[C>T]A	0.013222148	0.004465364	0.017665696	0.011104291	0.015233542	0.001852473	0.008086984	0.022618087	0.010597455	0.003714913	0.001475001	0.045380326
G[C>T]C	0.007326738	0.008568648	0.008838907	0.012206248	0.015162304	0.000814012	0.002911167	0.016293249	5.65E-11	0.002842605	0.001883057	0.096626154
G[C>T]G	0.010860786	0.007409812	1.10E-08	7.44E-16	0.021336114	4.24E-07	1.53E-05	0.008637447	0.111454378	0.008128044	0.001938255	0.218195485
G[C>T]T	0.008361273	0.015830882	0.008980413	0.013855404	0.007255113	5.60E-26	0.005184742	0.017874559	0.00779939	0.004471174	4.76E-43	0.063033171
T[C>T]A	0.03352399	2.83E-06	1.58E-07	0.000195922	0.013180225	0.079680067	0.444433453	2.84E-07	2.02E-07	0.014777593	0.014107528	7.84E-21
T[C>T]C	0.021119334	0.003060052	0.016096653	0.026908861	0.004879509	0.006361144	0.090426162	0.013650897	0.02110676	0.006738886	0.003863308	0.006124027
T[C>T]G	4.73E-15	0.012291637	1.21E-17	0.016796116	0.003080963	0.010708436	0.054620451	0.018127129	0.096989478	1.41E-11	9.02E-05	0.007979367
T[C>T]T	0.019174178	0.051302528	0.001790565	0.033701301	6.28E-20	1.12E-13	0.264501525	0.014045328	0.019069021	9.32E-08	2.40E-13	5.07E-10
A[T>A]A	0.001572866	0.022978422	0.001378843	0.061602354	0.005199795	4.03E-18	0.000398924	0.010198137	0.002594505	1.34E-09	0.052688628	0.009538214
A[T>A]C	0.00564561	0.010790942	0.004944985	0.006778233	0.00283238	1.18E-07	0.000554893	0.005838639	0.001784373	0.001585016	0.009022296	0.016032288
A[T>A]G	0.002194626	0.012829357	0.00318797	0.006496259	0.001779314	0.000318909	0.000467144	0.008759963	0.006826154	8.20E-19	0.071034016	3.12E-95
A[T>A]T	0.011093643	0.021449185	0.018135649	0.010322465	0.006870147	1.25E-93	0.001425601	0.005973841	0.010182013	0.009018314	0.015211788	0.025312947
C[T>A]A	1.22E-21	4.60E-09	0.00075414	0.091373234	0.008800359	0.000673062	5.26E-41	0.005308111	0.000878282	5.69E-13	0.093673373	0.00037675
C[T>A]C	0.008085644	0.007310963	0.006222358	0.005056578	0.003202008	0.000352015	0.001376544	0.00613961	0.001418881	0.007151429	0.047770177	0.003583119
C[T>A]G	2.11E-05	1.07E-10	2.38E-07	5.12E-11	0.004654339	0.001015502	0.000671272	0.004413862	0.005905554	0.004273119	0.174672995	2.32E-41

C[T>A]T	0.015539743	0.00818214	0.007451018	0.005881878	2.00E-88	9.31E-20	0.002934498	0.006494104	0.003741665	0.023966926	0.059717211	4.16E-84
G[T>A]A	0.001222701	9.77E-59	2.72E-63	0.043848933	0.001750861	0.000272366	6.39E-16	0.002295304	2.56E-120	9.55E-27	0.054513025	0.001175778
G[T>A]C	0.004964915	0.000875017	0.002169546	0.004875177	0.001672121	0.000243686	0.000438289	0.003786059	1.99E-20	0.00179635	0.009549985	0.006685434
G[T>A]G	0.000787686	3.80E-05	0.001409503	0.011526655	0.00427357	0.000821842	3.56E-19	0.00778737	0.001072604	0.000461773	0.061005308	0.000639161
G[T>A]T	0.010915195	0.001992928	0.006071633	0.008753284	6.19E-61	0.000184067	0.001482038	0.004787553	3.22E-19	0.004871823	0.014299163	0.001963147
T[T>A]A	0.015017359	0.016141538	1.41E-06	0.08993686	7.19E-33	1.95E-15	0.001432082	1.33E-68	0.022778067	0.0127769	0.058832316	0.014644435
T[T>A]C	0.010429255	0.004204244	0.006565751	0.006989728	0.001516283	2.23E-21	0.001694813	0.004449801	0.000123094	0.002023331	0.023612938	0.002911347
T[T>A]G	0.003009567	0.003309342	0.001977669	0.006216552	0.002940064	7.68E-15	0.001072488	0.003809828	0.002458677	0.001535084	0.070913116	3.70E-32
T[T>A]T	0.022107277	0.011153675	0.01151704	0.022612817	0.001095395	8.51E-34	0.002816025	0.006866136	0.012060169	0.013255932	0.031303167	0.006846787
A[T>C]A	7.48E-12	0.144908925	1.40E-12	0.001675912	0.010475369	1.03E-74	5.40E-60	0.035147517	0.00015907	0.004146269	0.01227851	0.022865782
A[T>C]C	0.009840215	0.006711506	0.003028327	0.005018248	0.006635395	0.001155071	1.95E-09	0.013976301	0.007497097	0.005182453	0.000197048	0.007753005
A[T>C]G	0.002764099	0.0322825	0.002774096	0.004936131	0.07547384	0.00185114	2.98E-23	0.029652087	9.69E-05	0.004004898	0.003174733	0.007954664
A[T>C]T	0.019000303	0.06764419	0.006807849	0.001118522	0.002116609	0.00044354	0.001281634	0.024011726	0.010643775	0.009791258	3.89E-53	0.007053751
C[T>C]A	0.01217058	0.012207796	0.00125267	0.005030234	0.013326921	0.000739133	2.10E-19	0.016777972	0.002749286	0.008330355	0.005309837	0.010273481
C[T>C]C	0.015825228	1.17E-13	0.002700417	0.000618293	0.025334614	0.002056312	1.32E-08	0.015543269	0.007707052	0.023329612	0.002255375	0.004115047
C[T>C]G	0.003727429	2.41E-22	5.74E-52	0.012804023	0.049677927	0.0016859	6.89E-52	0.028029661	0.000188763	0.011716501	0.002222182	0.011433288
C[T>C]T	0.011658064	0.016418922	0.003291022	0.005556724	0.010629559	0.000850252	4.36E-21	0.019381332	0.001409506	0.076175789	0.002356864	0.006163044
G[T>C]A	0.014619579	0.008138509	0.003688263	0.000757692	0.007585075	0.00040214	4.98E-23	0.012176052	0.00684656	0.004766679	0.002931189	0.03686015
G[T>C]C	0.009738607	7.04E-09	0.001835832	0.00357344	0.008310084	0.000715364	1.17E-32	0.011644628	0.006083485	0.010964409	0.000469346	0.026609547
G[T>C]G	0.000235449	0.007707171	0.003974213	0.002586659	0.165766178	0.000708171	0.000746454	0.011966988	0.003904397	0.004957805	7.16E-19	0.010976232
G[T>C]T	0.01639872	0.006354349	0.005893543	0.002601462	0.006041306	0.001100761	3.18E-32	0.016695837	0.011762941	0.013362764	0.001323863	0.018388451
T[T>C]A	0.034008105	0.008904116	0.002303626	0.003293674	0.006511238	0.000357114	0.000471203	0.010005043	0.005824995	0.017283306	0.007317576	0.021099792
T[T>C]C	0.019288768	0.002109765	0.00320813	0.002077512	0.009778872	0.000823415	1.06E-06	0.014426951	0.008994505	0.013246987	0.000391056	0.020300009
T[T>C]G	0.000284801	0.008751417	0.009571802	1.07E-06	0.244253639	4.91E-25	0.00139918	2.50E-08	7.49E-06	0.007449286	0.000932675	0.000853416
T[T>C]T	0.023971357	0.010050029	0.005687101	0.004327415	0.006126566	0.001855395	4.83E-19	0.018639604	0.014907968	0.018781447	0.001873785	0.018509242
A[T>G]A	0.01774965	0.007600982	2.59E-119	0.001902046	0.004052708	2.40E-114	0.000567495	0.003707897	0.001206526	0.008086091	0.002086991	7.27E-26
A[T>G]C	0.003362789	0.001533967	0.000463598	0.001838683	0.001891704	0.000350219	0.00021165	0.003951877	0.001523624	0.003009312	0.00016437	4.92E-155
A[T>G]G	0.008708453	0.00543387	0.002208013	0.001512733	0.003796471	4.58E-19	0.001044882	0.008014549	0.000985531	0.001284542	0.002370925	4.59E-121
A[T>G]T	0.008899973	0.007983621	0.002814363	0.001182769	0.002159272	0.000538724	2.11E-05	0.004630904	0.003873596	0.041026923	6.20E-05	1.23E-17

C[T>G]A	0.007159933	0.004435473	3.08E-08	0.000692024	0.004542442	6.41E-05	4.24E-26	0.001299979	0.002386605	0.004614886	0.002990561	8.72E-05
C[T>G]C	0.004657077	0.001108314	0.001215664	0.000856212	0.003585765	0.000714664	5.19E-05	0.003116512	0.000755683	0.015758648	0.001786731	0.001069594
C[T>G]G	0.008621335	0.004047587	0.001402483	7.67E-65	0.004957801	0.000783533	0.000922033	0.006385227	0.000997684	0.007652613	0.004059241	0.001342244
C[T>G]T	3.18E-12	6.27E-08	5.88E-08	4.04E-94	0.006400857	0.001728603	2.19E-13	4.97E-31	2.19E-22	0.203746193	0.002625103	9.92E-51
G[T>G]A	0.004652249	0.000940692	0.000466199	0.001830746	0.001387082	0.000316907	0.000106246	0.00406236	0.001772508	0.001427577	0.001784384	9.45E-140
G[T>G]C	0.002382987	0.000334481	2.24E-79	0.002217856	0.002149047	0.000321893	7.11E-45	0.003794301	0.000914715	0.005148961	0.000203699	0.000608247
G[T>G]G	0.005375774	0.001892649	3.73E-115	6.65E-242	0.014939338	0.000487291	0.001287056	0.014599489	0.002362551	7.95E-70	0.002024059	1.14E-202
G[T>G]T	0.003957504	1.48E-09	3.79E-22	0.001177971	0.004288612	0.000996111	2.65E-37	0.005640137	0.001414265	0.064200466	0.000688049	0.006387646
T[T>G]A	0.030870782	0.004387176	6.75E-136	0.003533614	9.97E-25	1.95E-130	1.92E-23	3.39E-71	1.72E-17	0.018212847	0.001902318	0.000974684
T[T>G]C	0.007728637	0.001231713	0.002525163	0.000979102	0.001894382	0.000273789	0.000599829	0.005414129	0.00241928	0.0059437	0.001105965	3.52E-115
T[T>G]G	0.01371476	0.006088728	0.002155829	0.000610837	0.003294366	0.000204233	0.001114696	0.009258813	0.003691885	0.00741614	0.0030769	4.61E-121
T[T>G]T	0.021755726	0.015117936	0.004672337	0.003424823	0.003652721	2.99E-38	0.00063799	0.006356897	0.013311874	0.088967427	6.87E-59	0.002043306

Table S2b. Abundance fractions matrix of single-base substitutions (SBS).

SampleID	SBS1	SBS2	SBS3	SBS4	SBS5	SBS6	SBS7	SBS8	SBS9	SBS10	SBS11	SBS12
FP1705100059DN01	0.111834603	0.041643542	0.195937281	0.108927187	0.020726011	0.011337477	0.050678369	0.297348361	0.085719811	0.007571764	0.035110628	0.033164964
FP1705100061DN01	0.062394486	0.023780204	0.02715238	0.015817312	0.011079285	0.422559466	0.15338804	0.093016922	0.12612133	0.011024266	0.014029844	0.039636465
FP1705100065DN01	0.057597527	0.205015649	0.097143085	0.016731253	0.0013437	0.124577978	0.155903843	0.168118715	0.056563933	0.053081966	0.017560923	0.04636143
FP1705100067DN01	0.075246467	0.055147309	0.147977189	0.049290915	0.045497689	0.013519601	0.03497452	0.299879452	0.177098229	0.007139833	0.018798146	0.075430649
FP1705100071DN01	0.173354093	0.014345799	0.06049055	0.016490971	0.017924892	0.250745758	0.17913338	0.167663949	0.044026408	0.027233632	0.029309084	0.019281484
FP1705100073DN01	0.287502138	0.06053893	0.048301094	0.021543229	0.067583365	0.104039114	0.044579654	0.234090531	0.009425811	0.06214063	0.038247923	0.022007582
FP1705100077DN01	0.036594038	0.018574533	0.221411685	0.064544599	0.026039613	0.029232455	0.109240713	0.236296922	0.122167261	0.012624234	0.050983463	0.072290484
FP1705100079DN01	0.23819212	0.055456168	0.097557283	0.010578173	0.018862106	0.15357725	0.117614081	0.188945259	0.030771606	0.010452255	0.03985112	0.03814258
FP1705100081DN01	0.158031004	0.063257567	0.142602523	0.040484375	0.027537463	0.132065463	0.104846131	0.129042385	0.099884231	0.008058071	0.029373092	0.064817694
FP1705100089DN01	0.070369742	0.185258099	0.073487948	0.042724678	0.012421831	0.039422837	0.083988543	0.282964013	0.057377089	0.103084615	0.018398428	0.030502179
FP1705100091DN01	0.215632888	0.092088875	0.148010598	0.028565538	0.022729832	0.093021886	0.04899362	0.151332672	0.105275775	0.025652655	0.013770207	0.054925456
FP1705100093DN01	0.177486586	0.064058253	0.162261594	0.05080188	0.053379639	0.084184643	0.067054061	0.154039524	0.09630701	0.022352244	0.030764213	0.037310353
FP1705100095DN01	0.061050912	0.033511047	0.141737397	0.082835065	8.547335673 05357e-321	0.031062555	0.091701897	0.417551899	0.08684699	0.004385891	0.014897663	0.034418685
FP1705100097DN01	0.025970102	0.029458725	0.1547729	0.189202973	0.027511816	0.047833237	0.117786275	0.208866207	0.127750307	0.017235273	0.009169175	0.044443009
FP1705100099DN01	0.309207738	0.083595958	0.046720817	0.01643971	0.019774522	0.231710436	0.029012538	0.190555439	0.016170995	0.027500942	0.024427424	0.004883482
FP1705100101DN01	0.141251711	0.052175618	0.126928088	0.158658546	0.037223644	0.023107606	0.035902951	0.169699524	0.108992207	0.029197004	0.036664059	0.080199041
FP1705100103DN01	0.033482089	0.054343307	0.329844469	0.055714865	0.01357291	0.040203617	0.082812869	0.159407358	0.141116789	0.017092598	0.01558496	0.05682417
FP1705100109DN01	0.10344284	0.087342481	0.065246978	0.061489103	0.016854174	0.361227244	0.159080075	0.011204375	0.095763519	0.007400812	0.007536464	0.023411934
FP1705100111DN01	0.136049707	0.060307156	0.169496698	0.062623414	0.018307149	0.020913649	0.04801561	0.244963038	0.117418192	0.025423709	0.028423188	0.068058491
FP1705100113DN01	0.110268895	0.056889107	0.11380091	0.025915354	0.020145812	0.055006979	0.085065176	0.157884071	0.222999393	0.030249239	0.016584657	0.105190407
FP1705100115DN01	0.165794206	0.07737752	0.145384644	0.035249783	0.03802574	0.016708336	0.020097006	0.282782309	0.099298811	0.024597971	0.067164213	0.027519462
FP1705100117DN01	0.09340528	0.063704628	0.077028998	0.056457525	0.015067073	0.11257105	0.236017633	0.141073458	0.122279238	0.028343045	0.010698572	0.0433535
FP1705100119DN01	0.089668895	0.040884209	0.044805606	0.010289235	0.010288236	0.346286948	0.183411873	0.125761611	0.056740214	0.040709998	0.01160216	0.039551016
FP1705100121DN01	0.162245703	0.064167791	0.199233424	0.036804987	0.104926324	0.034949065	0.037980821	0.103652969	0.164996236	0.027554682	0.010201487	0.053286512
FP1705100123DN01	0.042419687	0.051586275	0.080644606	0.106056581	0.026194062	0.011308611	0.047786213	0.469241327	0.158414822	0.006347815	5.23E-103	2.929809279 83859e-321

FP1705100125DN01	0.16941281	0.057191836	0.124642806	0.050146106	0.012185863	0.013542323	0.033415041	0.323583817	0.136729154	0.015796235	0.029832975	0.033521035
FP1705100127LD02	0.160217467	0.063827454	0.198899719	0.027528094	0.015701021	0.053656403	0.041926781	0.238678792	0.103811137	0.032655822	0.02815812	0.034939189
FP1705100129DN01	0.210601575	0.09412405	0.101240802	0.0411834	0.016943522	0.032051676	0.022851563	0.223710497	0.155568204	0.022932553	0.024018665	0.054773494
FP1705100131DN01	0.156653486	0.065460982	0.088635198	0.002881947	0.099027849	0.023443659	0.030949696	0.210192806	0.220913099	0.023491113	0.018462442	0.059887721
FP1705100133DN01	0.038913602	0.090173877	0.040257669	0.050117737	0.009491911	0.012424332	0.017098198	0.078856885	0.062052684	0.010779853	0.576216094	0.013617158
FP1705100135DN01	0.170251148	0.082242685	0.140287631	0.043100586	0.012785107	0.032711729	0.047704864	0.178927787	0.15125264	0.048049925	0.025398942	0.067286956
FP1705100137DN01	0.066780765	0.123692108	0.093488884	0.027728222	0.009135703	0.216240083	0.218442538	0.072085072	0.094047642	0.011436205	0.021952148	0.044970629
FP1705100139LD02	0.095677596	0.082600518	0.163310892	0.057557763	0.019387754	0.044376921	0.067478241	0.194909182	0.151044031	0.018150893	0.01554614	0.089960068
FP1705100141DN01	0.109980175	0.043941602	0.107890857	0.038434706	0.017962027	0.065527921	0.065335715	0.300445798	0.164350225	0.017552964	0.0167006	0.051877411
FP1705100143DN01	0.176216455	0.06569143	0.090670155	0.038823854	0.028405631	0.057111709	0.05040113	0.216016248	0.128876272	0.038547608	0.024870465	0.084369042
FP1705100145DN01	0.038230077	0.072491321	0.144154896	0.032974542	0.004813032	0.196086033	0.217640036	0.097167339	0.115136246	0.024760785	0.009414804	0.047130889
FP1705100149DN01	0.141380615	0.09485062	0.106951551	0.034976417	0.017326527	0.119909016	0.12181705	0.192373802	0.077802819	0.006573859	0.041459008	0.044578717
FP1705100153DN01	0.222376893	0.034186592	0.056601974	0.017026788	0.017849315	0.281766121	0.08501394	0.187628967	0.028318759	0.021567448	0.02695607	0.020707134
FP1705100155DN01	0.091792343	0.086859047	0.085521217	0.010574255	0.007172565	0.110385982	0.103222403	0.141248664	0.05464824	0.016549268	0.26594096	0.026085057
FP1705100159DN01	0.086180253	0.219625018	0.115942349	0.001968209	0.01976036	0.08492409	0.066686691	0.217556946	0.076756259	0.029656336	0.031942173	0.049001315
FP1705100161LD02	0.070207985	0.051305775	0.063492913	0.031789708	0.019125786	0.192323994	0.13714732	0.165127507	0.164913249	0.02610213	0.00543834	0.073025294
FP1705100163DN01	0.149788527	0.081850384	0.054410379	0.036185016	0.020925441	0.208078794	0.129223659	0.104838297	0.148902382	0.010756233	0.01285	0.042190886
FP1705220177LD01	0.098888545	0.065932674	0.201840967	0.11615149	0.064818578	0.027759681	0.058310519	0.115144635	0.148314998	0.019931741	0.02519991	0.05770626
FP1705220179LD01	0.162290902	0.235242153	0.127021249	0.028883348	0.015727206	0.016232302	0.022263108	0.221710295	0.059585606	0.029445692	0.026415165	0.055182974
FP1705220181LD01	0.223119702	0.069315119	0.060622779	0.011368347	0.025473409	0.128904982	0.092908276	0.176996508	0.033253313	0.101185268	0.027338185	0.049514111
FP1705220182LD01	0.12668066	0.062280056	0.200812005	0.026180137	0.032555392	0.033301924	0.081767324	0.199653901	0.1411737	0.007239526	0.015521182	0.072834193
FP1705220184LD01	0.037610299	0.01003104	0.039871119	0.009079061	0.01344092	0.137130934	0.608077971	0.039337887	0.060653292	0.009633679	0.009114677	0.026019121
FP1705220185LD01	0.059463439	0.068249767	0.143379517	0.038427647	0.012071399	0.077418774	0.173915238	0.193063049	0.128279746	0.019361203	0.012769713	0.073600507
FP1705220186LD01	0.15137348	0.088884538	0.043205772	0.376070286	0.03563266	0.012578176	0.045288241	4.93E-13	0.13361317	0.020229661	0.04728042	0.045843595
FP1705220187LD01	0.136773151	0.156628825	0.126990529	0.126375845	0.014126396	0.024786125	0.044894373	0.201300753	0.041622641	0.061149252	0.025900532	0.039451581
FP1705220188LD01	0.148296816	0.061847635	0.10847707	0.045468572	0.076283849	0.092359127	0.102810631	0.133027873	0.105926017	0.045775476	0.026529559	0.053197376
FP1705220189LD01	0.137851806	0.027226543	0.066695291	0.017447172	0.006828859	0.469477642	0.148457828	0.079665491	0.005369412	0.004402071	0.024872702	0.011705183
FP1705220190LD01	0.151586834	0.078765498	0.094043912	0.018969196	0.016833469	0.142981546	0.103621441	0.19898158	0.062575185	0.067329896	0.030471722	0.033839721
FP1705220191LD01	0.064013114	0.065954609	0.121145691	0.019334406	0.336795896	0.053901096	0.083491875	0.016520106	0.191908112	0.003239526	0.02195266	0.021742909

FP1705220192LD01	0.111226754	0.069513476	0.083918941	0.030870137	0.011476191	0.190461886	0.132595609	0.180647772	0.098180446	0.027042445	0.015186009	0.048880333
FP1705220195LD01	0.125202791	0.095869844	0.098556959	0.028106593	0.024479493	0.093633987	0.096233543	0.21089645	0.087253274	0.070079335	0.019070708	0.050617023
FP1705220196LD01	0.052537858	0.04514483	0.078511104	0.022163181	0.033385547	0.170057731	0.324061809	0.099897562	0.091740576	0.024781913	0.0105171	0.047200791
FP1705220197LD01	0.147147071	0.052011565	0.130556219	0.029432975	0.033822158	0.150250653	0.089151616	0.179686107	0.070125459	0.033009052	0.02085182	0.063955306
FP1705220198LD01	0.328798114	0.071919507	0.144385862	0.016415919	0.031001503	0.077653448	0.01453514	0.16045332	0.043621347	0.035157627	0.019141328	0.056916886
FP1705220199LD01	0.183357294	0.061882893	0.089997453	0.018704405	0.020280324	0.158116558	0.113356313	0.13146526	0.054537784	0.102584724	0.037593091	0.0281239
FP1705220200LD01	5.62E-134	0.013096275	0.017376922	0.006211145	0.068144094	2.084957025<br>45006e-321	0.012824746	0.286968852	0.218855254	0.026487004	0.014874109	0.335161598
FP1705220201LD01	0.043318595	0.037895671	0.144899868	0.040053348	0.017214683	0.067593663	0.093620944	0.263482549	0.186565681	0.020548902	0.015565591	0.069240506
FP1705220202LD01	0.195641164	0.063034148	0.089455842	0.017830933	0.105709371	0.141878662	0.07882264	0.144693157	0.072302032	0.041718682	0.023870689	0.02504268
FP1705220203LD01	0.038602427	0.091016169	0.225823146	0.04521847	2.26E-115	0.07864908	0.167288599	0.115054498	0.091997295	0.081358401	0.013186303	0.05180561
FP1705220204LD01	0.181062217	0.103038257	0.18001629	0.030321998	0.059230665	0.072983206	0.051491635	0.121338501	0.120324816	0.017607095	0.022242271	0.040343047
FP1705220205LD01	0.2960731	0.101350698	0.106655765	0.0452127	0.021510125	0.087113074	0.02694416	0.13668065	0.093082043	0.042767932	0.015053044	0.027556708
FP1705220206LD01	0.146206793	0.107580746	0.097549082	0.040020606	0.016415552	0.153702843	0.114183407	0.142747213	0.090924697	0.025314721	0.012187638	0.053166703
FP1705220208LD01	0.06204872	0.063159504	0.290174519	0.052656559	0.044910941	0.06455441	0.083048511	0.114328475	0.107671203	0.025638462	0.01881703	0.072991668
FP1705220209LD01	0.149546521	0.061746997	0.21164015	0.060767064	0.037230236	0.041922506	0.056489946	0.211847151	0.088054074	0.01842829	0.029694206	0.032632859
FP1705220210LD01	0.388302207	0.069378035	0.11186024	0.005687389	0.029606542	0.025651223	1.51E-69	0.24718331	1.71E-128	0.036703255	0.055056002	0.030571797
FP1705220211LD01	0.144903559	0.046640443	0.033822539	0.02435073	0.018681494	0.193039985	0.106486028	0.253448181	0.075476751	0.045177442	0.008480033	0.049492814
FP1705220212LD01	0.28688993	0.062300446	0.099913595	0.040289414	0.037797643	0.021435248	0.013689917	0.284023763	0.058307159	0.027929928	0.021917438	0.04550552
FP1705220213LD01	0.120329754	0.082432418	0.114365883	0.035698092	0.020833828	0.088580945	0.081112422	0.142463869	0.200834397	0.035026543	0.016260035	0.062061813
FP1705220214LD01	0.109449909	0.060629749	0.164372618	0.050754957	0.11777919	0.075158148	0.083006766	0.138258846	0.135597124	0.014657446	0.021072372	0.029262875
FP1705220215LD01	0.066770061	0.074479807	0.196139012	0.040957082	0.028033515	0.018918486	0.04387879	0.221133576	0.204829463	0.019047544	0.014485192	0.071327472
FP1705220216LD01	0.061451643	0.097096257	0.133891018	0.020642351	0.011605279	0.055474374	0.137670738	0.200507152	0.165183806	0.01759911	0.017783968	0.081094303
FP1706060153LD01	0.598119258	0.126164504	0.014811029	0.039293865	0.029453707	0.06222157	0.000555919	6.33E-90	6.59E-200	0.092286408	0.037093742	7.82E-95
FP1706060155LD01	7.87E-58	0.049147783	0.034754249	0.118109378	0.05339052	0.007384643	0.043139012	0.556277141	0.130138872	1.78E-97	0.007658402	1.34E-201
FP1706060156LD01	0.007750287	5.06E-41	0.006223188	2.84E-103	0.080156455	1.269748709<br>812e-321	0.022531905	0.220801128	0.087115606	6.35E-34	6.605657684<br>89747e-321	0.575421431
FP1706060159LD01	0.323556753	0.090770213	0.099420389	0.039637292	0.185501831	0.083518182	0.021016627	0.044675217	0.052175944	0.030575551	0.026763909	0.002388092
FP1706060160LD01	0.195477271	0.111615388	0.113845053	0.033176623	0.013364057	0.083528407	0.072465355	0.178332322	0.139401292	0.01103693	0.020805448	0.026951853

FP1706060161LD01	0.140114598	0.061006877	0.156424058	0.059045871	0.054141062	0.057074737	0.074682204	0.121121212	0.119537594	0.086987005	0.018381124	0.051483659
FP1706060162LD01	0.076395989	0.038157185	0.082759493	0.011268985	0.006443129	0.291497468	0.173473233	0.135825718	0.091241175	0.027754882	0.029688266	0.035494476
FP1706060163LD01	0.018929162	0.058560655	0.063521407	0.042487959	0.033673605	0.043966549	0.112853448	0.438327413	0.117338128	0.012825905	0.013291597	0.044224171
FP1706060165LD01	0.051287941	0.090461764	0.083131011	0.032774965	0.011137747	0.165268573	0.1822349	0.089052996	0.059258819	0.017720088	0.186699668	0.030971528
FP1706060167LD01	0.137744375	0.06615452	0.123049555	0.034271388	0.028442477	0.111268269	0.107859328	0.22070587	0.098561917	0.017599401	0.013227403	0.041115496
FP1706060169LD01	0.171365227	0.045863871	0.060383424	0.081531849	0.069728721	0.118591991	0.065225774	0.149186601	0.111027599	0.053523119	0.015404439	0.058167385
FP1706060170LD01	0.083535784	0.0850376	0.071308603	0.085491871	0.015143339	0.092652171	0.105073947	0.096894045	0.090529579	0.004926044	0.237827357	0.03157966
FP1706060171LD01	0.126779587	0.128591172	0.060330679	0.023471755	0.010730879	0.204160637	0.115372901	0.165409508	0.078468962	0.023402748	0.01659761	0.046683561
FP1706060172LD01	0.099081145	0.054902668	0.109439392	0.04242551	0.0147778	0.098341061	0.196026773	0.098633644	0.17437781	0.022665346	0.010847612	0.078481239
FP1706060175LD01	0.201339942	0.07748287	0.159986678	0.043300635	0.022381125	0.037936873	0.044246267	0.172402279	0.109643991	0.046114444	0.028847477	0.056317418
FP1706060176LD01	0.089152331	0.050184637	0.096875663	0.061571339	0.019081554	0.094598838	0.123848464	0.184693771	0.169506999	0.020839971	0.007654338	0.081992096
FP1706060177LD01	0.124618185	0.07564075	0.172364701	0.063482066	0.028423739	0.033819025	0.082333521	0.177162234	0.107920834	0.01786924	0.034617076	0.081748629
FP1706060178LD01	0.135908342	0.049168507	0.207291352	0.025135485	0.00636542	0.128673893	0.100094017	0.169633862	0.095152891	0.017025596	0.014720601	0.050830034
FP1706060179LD01	0.061027805	0.065389319	0.037124768	0.27205407	0.02887291	0.003031994	0.059906969	0.307279042	0.099562058	0.015889343	0.016951248	0.032910474
FP1706060180LD01	0.16701638	0.058175565	0.151110622	0.052532279	0.017284278	0.021891183	0.028091258	0.144466833	0.186918473	0.047305946	0.032231348	0.092975835
FP1706060184LD01	0.091616943	0.048747502	0.194734566	0.030928249	0.023202871	0.063522873	0.103429424	0.138403602	0.151708664	0.033266879	0.024038751	0.096399675
FP1706060185LD01	0.129244175	0.067015545	0.095333402	0.059135185	0.024292933	0.144562404	0.17425717	0.091107443	0.098096335	0.073301585	0.004310295	0.039343528
FP1706060188LD01	0.102576499	0.078562242	0.195396556	0.037006897	0.008328663	0.059798751	0.109110162	0.176133573	0.120395056	0.037269678	0.006399137	0.069022786
FP1706060191LD01	0.066425302	0.111086418	0.180121866	0.051352045	0.007178373	0.082921454	0.178522575	0.133854195	0.102690042	0.02030715	0.01440105	0.05113953
FP1706060192LD01	0.106481502	0.056150237	0.080114075	0.011931522	0.48983457	0.023469127	0.022037036	7.66E-30	0.205146747	0.003436098	1.57E-42	0.001399087
FP1706060193LD01	0.078869535	0.07233405	0.124355656	0.037928452	0.019646198	0.101959465	0.148490303	0.207035989	0.097758762	0.037340149	0.020506595	0.053774845
FP1706060194LD01	0.005636551	0.002568087	0.034977509	0.01146314	0.013983273	0.289421756	0.341755163	0.151738929	0.035888439	0.076678312	0.009899148	0.025989694
FP1706060195LD01	0.117026705	0.047440812	0.203562458	0.023050984	0.02318157	0.032556414	0.048472063	0.22587065	0.178214198	0.016588337	0.01726454	0.06677127
FP1706060196LD01	0.105376552	0.095204382	0.135369877	0.027120132	0.020940297	0.106645898	0.154588131	0.134247857	0.147061892	0.014632561	0.016646764	0.042165657
FP1706060197LD01	0.019108358	9.92E-138	0.126438789	0.013324791	0.060683014	0.041612822	0.056181878	0.326781325	0.037123605	0.035823851	0.005148661	0.277772906
FP1706060198LD01	0.135851806	0.127302917	0.175396563	0.014182156	0.005919093	0.077356559	0.081639738	0.184085044	0.127097404	0.01521705	0.016952926	0.038998744
FP1706060199LD01	0.113756402	0.042924087	0.071869543	0.011201515	0.042792883	0.019338796	0.042218166	0.455892193	0.111372907	0.012804336	0.01519661	0.060632562
FP1706060200LD01	0.196018883	0.078446947	0.121779544	0.022992212	0.022497586	0.038994846	0.055317225	0.264495482	0.10927903	0.021229616	0.017485717	0.05146291
FP1706060201LD01	0.058401947	0.051686811	0.288258623	0.02302198	0.019580921	0.032695306	0.085214489	0.13513909	0.223486196	0.01050144	0.002792192	0.069221007

FP1706060202LD01	0.022284764	0.029157731	0.08890251	0.003734636	0.006954224	0.118742398	0.310960615	0.084510256	0.104193179	0.019161788	0.183094683	0.028303216
FP1706060203LD01	0.063497095	0.068791828	0.181581494	0.033515041	0.01263379	0.024207998	0.08980647	0.234486551	0.205854514	0.011337836	1.17E-22	0.074287383
FP1706060204LD01	0.172269204	0.113091146	0.073040953	0.032558511	0.010467972	0.186869849	0.102373132	0.166866452	0.032077416	0.081810034	0.015023462	0.013551869
FP1706060205LD01	0.150803617	0.047072384	0.143882347	0.024341653	0.063014862	0.171911315	0.110486044	0.102447407	0.109277539	0.019852478	0.02366708	0.033243273
FP1706060206LD01	0.209448428	0.08913305	0.071341594	0.035048	0.015316759	0.19358703	0.146096871	0.07686813	0.086381328	0.028752503	0.019317255	0.028709052
FP1706060207LD01	0.05732325	0.073406997	0.171210965	0.018464711	0.015953535	0.020806419	0.158822134	0.299090735	0.124054554	0.012619341	0.019879625	0.028367736
FP1706060208LD01	0.235474381	0.096010213	0.080640937	0.024715708	0.007081889	0.094160638	0.065801247	0.208358437	0.048184005	0.052513679	0.041128221	0.045930646
FP1706060209LD01	0.050337847	0.021537612	0.18938745	0.019055563	0.023539007	0.182873581	0.149722165	0.109715905	0.158943037	0.018843901	0.013632225	0.062411705
FP1706060210LD01	0.076610584	0.04512952	0.245456414	0.041371176	0.041582773	0.099994308	0.10151151	0.199150093	0.082851026	0.017519352	0.018110187	0.030713056
FP1706060212LD01	0.099790116	0.069812801	0.167799482	0.025045984	0.021886454	0.033867689	0.084902299	0.184313677	0.191359535	0.027599789	0.012141797	0.081480377
FP1706060213LD01	0.113465205	0.127462239	0.094137379	0.024330728	0.007855886	0.38523942	0.182385235	1.20E-43	0.044574616	7.72E-78	0.007481967	0.013067324
FP1706060214LD01	0.105413563	0.037581123	0.136485409	0.027852336	0.014813906	0.151633922	0.134055718	0.134597453	0.121328685	0.058935295	0.008209818	0.069092771
FP1706060215LD01	0.110225508	0.056920436	0.164718266	0.007833071	0.116820699	0.054839349	0.069712935	0.101240401	0.25082168	0.013322004	0.019084371	0.034461281
FP1706060216LD01	0.129272847	0.078426433	0.139786743	0.021448472	0.026758909	0.065872661	0.083666313	0.227010341	0.107777099	0.037238805	0.01758699	0.065154389
FP1706060217LD01	0.197896074	0.013436326	0.049458645	3.04E-19	0.047410514	0.293701018	0.170209746	0.1417386	0.008532811	0.034694481	0.028530282	0.014391502
FP1706060218LD01	0.131808653	0.055359767	0.121131881	0.043452182	0.015207001	0.038841112	0.100868382	0.129911146	0.137066262	0.099796198	0.021778779	0.104778637
FP1706060221LD01	0.011819361	6.73E-75	0.022134027	0.026252205	0.026435458	0.109646108	0.136052445	0.291502411	0.061095363	0.004183786	0.011676467	0.299202368
FP1706060222LD01	0.10412498	0.074123254	0.104840658	0.035189575	0.012170145	0.158156691	0.228324386	0.077626256	0.147672773	0.019138712	0.007101524	0.031531047
FP1706060223LD01	0.108500034	0.057072112	0.060926478	0.01578242	0.013845503	0.263444682	0.168553448	0.140889881	0.073543044	0.038476572	0.013775877	0.045189948
FP1706060224LD01	0.147491866	0.074639095	0.161345203	0.023603438	0.028373305	0.033758273	0.06537765	0.185258381	0.185403133	0.01631501	0.014102305	0.064332342
FP1706060225LD01	0.191309184	0.07814745	0.121962859	0.011318781	0.023595637	0.035729054	0.033501242	0.145398227	0.242270619	0.028372476	0.013450076	0.074944397
FP1706060226LD01	0.138409616	0.051621484	0.159029999	0.019990653	0.011104618	0.08100879	0.102730562	0.124018144	0.221451698	0.017707422	0.013559407	0.059367605
FP1706060227LD01	0.218516944	0.136933386	0.195196232	0.014383549	0.014070132	0.050139642	0.029788688	0.162991499	0.082317167	0.023856643	0.034421924	0.037384194
FP1706060228LD01	0.176972566	0.090800827	0.113259026	0.014076714	0.023889969	0.017548054	0.027917206	0.243444116	0.087718616	0.068744342	0.028372932	0.107255631
FP1706060229LD01	0.080366021	0.18077887	0.135215375	0.019006264	0.006963781	0.038674373	0.082680455	0.235010963	0.104115539	0.02697261	0.018020978	0.07219477
FP1706060230LD01	0.212790749	0.092797116	0.116627754	0.016671477	0.013940098	0.055230636	0.065274037	0.235001553	0.043388183	0.085379112	0.024529331	0.038369954
FP1706060231LD01	0.07268686	0.096827503	0.276732751	0.003484008	0.017544579	0.025210902	0.045312773	0.147174491	0.197638301	0.024090977	0.01752629	0.075770564
FP1706060232LD01	0.155931534	0.056080325	0.183375212	0.028265284	0.031547827	0.031776685	0.050613128	0.167943383	0.194498653	0.023347529	0.014065998	0.062554441
FP1706060233LD01	0.096934554	0.076826592	0.119718301	3.12E-05	0.008965249	0.218272589	0.180360913	0.114530314	0.102984812	0.016270449	0.015921711	0.049183362

FP1706060234LD01	0.047287255	0.084971783	0.070871068	0.012242172	0.007054403	0.37453295	0.28170826	0.00948998	0.081380104	0.002419755	3.11E-83	0.02804227
FP1706060235LD01	0.021238702	0.043337403	0.155651737	0.014320714	0.057682509	0.107031779	0.283682536	0.116036771	0.102007555	0.016128412	0.018328343	0.064553539
FP1707040176LD01	0.175119385	0.050280471	0.238269668	0.091834599	0.039063844	0.066912011	0.067228008	0.106625282	0.072652475	0.040391778	0.033621236	0.018001242
FP1707040177LD01	0.063925696	0.042579531	0.262741499	0.119227237	0.027778327	0.085630677	0.086652513	0.058472786	0.15490633	0.030214813	0.012858541	0.05501205
FP1707040178LD01	0.105742125	0.082606388	0.074393087	0.069075099	0.007373795	0.187597688	0.221325901	0.089626509	0.09772338	0.026175868	0.016427933	0.021932226
FP1707040179LD01	0.185420833	0.131359673	0.108233942	0.05937597	0.006533578	0.12456641	0.089979893	0.15202527	0.055314093	0.033541342	0.029235577	0.024413418
FP1707180231LD01	0.154346899	0.094232915	0.118451159	0.161471713	0.025210801	0.047876345	0.071503696	0.153340797	0.066500124	0.026497587	0.027925173	0.052642792
FP1707180232LD01	0.076959628	0.098517537	0.146144106	0.2844497	0.021438729	0.032900843	0.085640734	0.038400763	0.110124544	0.00884183	0.038930061	0.057651526
TI1706140111LD02	0.074306076	0.068336094	0.18978281	0.027191798	0.018249894	0.091939418	0.13346155	0.184418623	0.097955978	0.032881924	0.028732727	0.052743109
TI1706140113LD03	0.065112035	0.075109522	0.032396244	0.005640019	0.00688109	0.379454808	0.201685145	0.127707125	0.046872526	0.014624217	0.014442349	0.03007492
TI1706140115LD02	0.277868401	0.060258013	0.089477633	0.011784712	0.02161093	0.151315682	0.0505788	0.212353745	0.03443354	0.011292372	0.057912908	0.021113263
TI1706140117LD01	0.091243005	0.067998693	0.089653166	0.006030715	0.015278849	0.096072311	0.117910208	0.240184155	0.142717527	0.024263583	0.040643374	0.068004415
TI1706140119LD01	0.165804307	0.058630357	0.183808489	0.022656449	0.009078225	0.105297349	0.128734532	0.146743247	0.086930304	0.019826783	0.033721319	0.03876864
TI1706140121LD01	0.175473629	0.072775932	0.078096314	0.012464588	0.019810006	0.041328359	0.049052941	0.238832292	0.22269138	0.021892117	0.020315946	0.047266496
TI1706140123LD01	0.063708609	0.068048037	0.12396697	0.013015362	0.346323746	0.054927824	0.08544471	1.58E-28	0.210291296	7.70E-47	0.014544905	0.019728542
TI1706140125LD01	0.20046272	0.073638379	0.041449662	0.036543546	0.016292754	0.008085919	0.031447114	0.273918119	0.084316309	0.157546713	0.013509816	0.06278895
TI1706140127LD01	0.222448136	0.059970798	0.098966107	0.001656221	0.022393775	0.126710572	0.060086542	0.266039128	0.052846611	0.011069008	0.046115469	0.031697635
TI1706140129LD02	0.275455101	0.086417243	0.030369165	0.013156446	0.023937194	0.222573775	0.0696724	0.147543271	0.056127985	0.035922712	0.022087949	0.016736759
TI1706140131LD01	0.140985973	0.049276817	0.087709656	0.013602598	0.032742551	0.317708049	0.131577824	0.103801125	0.044760797	0.049349481	0.012509745	0.015975383
TI1706140133LD01	0.004617847	0.002911354	0.029422565	2.45E-282	0.00098748	0.568438808	0.352314188	1.62E-141	0.019192899	4.69E-60	0.001634226	0.020480634
TI1706140135LD01	0.126615655	0.058527847	0.178161496	0.025781073	0.046589559	0.094324779	0.118908463	0.123636873	0.117259326	0.030114445	0.034723487	0.045356998
TI1706140137LD02	0.066952315	0.073491037	0.181294093	0.022955253	0.013354943	0.132797716	0.210727092	0.068644942	0.11717254	0.015744126	0.04899626	0.047869682
TI1706140139LD01	0.121870157	0.062612094	0.107984077	0.013523259	0.005345331	0.346689006	0.241579035	0.044678122	0.016385312	1.78E-85	0.0247179	0.014615707
TI1706140141LD01	0.11527364	0.039706073	0.134062984	0.035255995	0.008541335	0.132041364	0.149063088	0.1911825	0.100249177	0.021372511	0.021077207	0.052174126
TI1706140143LD01	0.116459886	0.035614559	0.230307631	0.019050017	0.014653903	0.048204251	0.078651171	0.203548364	0.152194827	0.015746259	0.013954596	0.071614535
TI1706140145LD01	0.250086223	0.065808911	0.133583246	1.67E-12	0.021052251	0.067681991	0.031260626	0.223469819	0.101310993	0.013459681	0.066879476	0.025406783
TI1706140147LD01	2.07E-60	0.073256051	0.048122559	0.065670486	0.004202992	0.016894433	0.055916582	1.11E-38	0.05289355	0.012668697	0.655792525	0.014582125
TI1706140149LD01	0.101654077	0.103901653	0.121786491	0.012851988	0.016056143	0.220324276	0.142129519	0.128185835	0.077907597	0.014119428	0.023775216	0.037307779
TI1706140151LD01	0.134979157	0.110285995	0.10481423	0.034952733	0.032958053	0.057756912	0.045825657	0.220473644	0.16108621	0.026740429	0.005711924	0.064415056

TI1706140153LD01	0.000925667	1.63E-43	0.042992768	8.84E-30	0.011300605	0.524683297	0.288079942	0.050407252	0.046756894	0.005609681	0.011769644	0.01747425
TI1706140155LD01	0.210610856	0.085077987	0.044504206	0.040278578	0.020705736	0.059799868	0.0306906	0.273254854	0.098830731	0.034596799	0.067453682	0.034196103
TI1706140157LD01	0.059914844	0.042683826	0.108421114	0.013693203	0.247769953	0.158428741	0.13548843	0.047725484	0.158239224	0.015405562	7.19E-69	0.012229619
TI1706140159LD01	0.230203019	0.061188875	0.129245772	0.014900495	0.00850484	0.148390098	0.083012659	0.205452354	0.038804896	0.026626914	0.027972007	0.025698072
TI1706140161LD01	0.016812893	5.34E-17	0.083701629	0.011035771	0.013945596	0.429899475	0.306989586	0.041805206	0.046623782	0.018244852	0.008986675	0.021954534
TI1706140163LD01	0.142728057	0.061568663	0.086205522	0.023782264	0.01240952	0.165910208	0.16245677	0.154455842	0.055880552	0.059083345	0.027444279	0.048074977
TI1706140165LD01	0.077385903	0.074682165	0.067836721	0.008632588	0.012331332	0.181121545	0.237984313	0.145396677	0.131993515	0.013695825	2.02E-103	0.048939416
TI1706140167LD01	0.188115519	0.02544014	0.059715611	0.00387092	0.021794873	0.255817277	0.1418085	0.132087432	0.029182357	0.089249583	0.027789134	0.025128653
TI1706140171LD01	0.06877673	0.022920099	0.052489716	0.007557017	0.010473706	0.179919712	0.473182108	0.068320772	0.059170363	0.023679452	0.008054908	0.025455417
TI1706140173LD01	0.131454124	0.039670264	0.12085201	0.002852034	0.041049423	0.278808762	0.168732048	0.094022371	0.042898301	0.025068176	0.030441104	0.024151383
TI1706140175LD01	0.060753984	0.054189458	0.055730239	0.00729695	0.019519723	0.127794906	0.292368881	0.136070599	0.103443634	0.045299506	0.061060529	0.036471592
TI1706140177LD01	0.045587552	0.023792778	0.213670517	3.85E-49	0.004211453	0.230422153	0.196942563	0.128896935	0.074367921	0.026986573	0.016752581	0.038368973
TI1706140179LD01	0.171413826	0.061630343	0.131725208	0.015286786	0.019044275	0.223691726	0.155833499	0.098767732	0.047262439	0.02398811	0.016532674	0.034823383
TI1706140181LD01	0.1786852	0.045756506	0.04256964	0.012346408	0.016505645	0.118880639	0.057656383	0.094097638	0.184488793	0.025306109	0.014301599	0.20940544
TI1706140183LD01	0.146799495	0.105861884	0.099207576	0.03376313	0.016796321	0.155208093	0.112744916	0.153763497	0.086549065	0.024563207	0.010210772	0.054532043
TI1706140185LD01	0.171475803	0.067157253	0.209049468	0.012627982	0.024055439	0.053195615	0.049483353	0.210107004	0.112913989	0.022004076	0.025984496	0.041945522
TI1706140187LD01	0.130569593	0.070723132	0.131940564	0.039898504	0.014585267	0.095795642	0.091181066	0.26066692	0.062891388	0.033709263	0.013610691	0.054427971
TI1706140189LD01	0.143858198	0.150927168	0.179761822	0.020286894	0.02292144	0.054494368	0.065678991	0.212324448	0.062167574	0.012815584	0.03101466	0.043748852
TI1706140191LD01	0.355009191	0.047677889	0.111846359	0.019350531	0.022292806	0.021721506	0.005304165	0.309836296	0.000879855	0.017162724	0.051558364	0.037360313
TI1706140193LD01	0.164825259	0.089870737	0.126636051	0.011874408	0.025134078	0.021721462	0.031484761	0.28748607	0.132486618	0.018705342	0.036224532	0.053550683
TI1706140195LD01	2.87E-14	0.104379091	1.61E-46	2.67E-303	0.087167086	0.001631143	0.04099088	0.653609665	0.039439977	0.00781018	7.75E-214	0.064971979
TI1706140197LD01	0.179348673	0.107503273	0.122461741	0.025119955	0.035808853	0.018063906	0.024028003	0.382754272	0.035468002	0.011446089	0.026004309	0.031992925
TI1706140199LD01	0.384723546	0.059948696	0.028434194	0.011824791	0.01518633	0.153853986	0.043745608	0.139488429	0.005245055	0.107753419	0.044657335	0.005138611
WGC106522DB	0.136131255	0.06963119	0.146616374	0.020872804	0.021664084	0.053220604	0.050603942	0.206456335	0.197460142	0.024284977	0.015277151	0.057781143
WGC106524D	0.072498261	0.031430031	0.062825043	0.02634923	0.008236015	0.273346338	0.245141861	0.130849816	0.072516039	0.027810116	0.010833931	0.038163319
WGC106526D	0.210795712	0.065920622	0.068572325	0.083133098	0.010487439	0.153954785	0.118000429	0.160836488	0.029668026	0.035069866	0.024184572	0.039376636
WGC106528D	0.083991015	0.056050456	0.226080213	0.023129136	0.020777543	0.044252039	0.058568969	0.174189187	0.194218917	0.016296204	0.024607164	0.077839157
WGC106530D	0.128245969	0.122043441	0.123424298	0.060861629	0.010729389	0.042129039	0.107144708	0.163739569	0.155600072	0.023841443	0.020378335	0.041862108
WGC106534D	0.353008447	0.064942359	0.110602857	0.032105403	0.020516571	0.06367798	0.021588746	0.181271331	0.017032473	0.044318997	0.032515255	0.05841958

WGC106536D	0.267466509	0.123215446	0.013013318	8.99E-33	0.041389087	0.060483937	0.000922704	0.26856549	0.078593518	0.048349851	0.039954575	0.058045564
WGC106538D	0.169154093	0.071105262	0.180121655	0.029669302	0.014300137	0.131630181	0.129714578	0.119722453	0.078775897	0.024193809	0.017295695	0.034316937
WGC106540D	0.075429905	0.062546097	0.135910513	0.024470976	0.012390832	0.163601163	0.148813761	0.082494028	0.172546537	0.009548148	0.048760133	0.063487908
WGC106542D	0.14977505	0.08367471	0.140432013	0.043872624	0.020286442	0.038723823	0.050411481	0.298479744	0.079718324	0.007014654	0.044253457	0.04335768
WGC106544D	0.184779353	0.086981951	0.060356736	0.026492024	0.010639134	0.189117804	0.113837425	0.196805049	0.04519855	0.029147372	0.020411485	0.036233117
WGC106550D	0.100479059	0.032771294	0.059620418	0.314875974	0.032972447	0.080501678	0.097068005	0.142207235	0.058827843	0.015303418	0.039857453	0.025515177
WGC106552D	0.043186726	0.066127088	0.159558574	0.070800698	0.022923843	0.113426169	0.227236295	0.019796356	0.148021939	0.005770105	0.08354078	0.039611428
WGC106554D	0.142668845	0.103262826	0.076418851	0.024251014	0.013323262	0.17742692	0.140273335	0.154960575	0.089864445	0.012976037	0.017768005	0.046805885
WGC106556D	0.152108153	0.052268849	0.043339133	0.036679754	0.013554907	0.1019721	0.069441727	0.165684691	0.108300237	0.190025865	0.027427927	0.039196656
WGC106558D	0.120838413	0.065676611	0.113432259	0.046957759	0.016896772	0.151432342	0.136306887	0.20227677	0.093230816	0.01049986	0.016862984	0.025588526
WGC106560D	0.028121389	0.025945644	0.032620774	0.02219413	0.006709597	0.362185388	0.275037618	0.130514394	0.053471846	0.029949017	0.007391983	0.025858218
WGC106562D	0.00501688	0.036466191	0.024743611	0.018702829	0.004806095	0.389085643	0.327495509	0.035257292	0.025069898	0.097311034	0.002828721	0.033216297
WGC106564D	0.126963638	0.062841083	0.1991318	0.107459516	0.02044822	0.05173576	0.066472759	0.175189481	0.074312824	0.032697939	0.046402048	0.036344931
WGC106566D	0.222691402	0.06969313	0.125620167	0.011928586	0.028491956	0.108612366	0.070570945	0.182974459	0.058899695	0.037829934	0.031340484	0.051346877
WGC106568D	0.117465231	0.06457011	0.135296218	0.015121233	0.011752324	0.084866644	0.081353125	0.178478436	0.188179784	0.023345604	0.024023909	0.075547381
WGC106570D	0.036088046	0.075660711	0.230164163	0.030802608	0.009393595	0.102975883	0.143679209	0.078138106	0.184193441	0.014501691	0.02147978	0.072922767
WGC106572D	0.131456159	0.071185039	0.108673296	0.021098055	0.054627392	0.108834793	0.106965985	0.090225765	0.129310045	0.014263126	0.123935561	0.039424784
WGC106574D	0.128273709	0.076408201	0.114317772	0.03229195	0.007085003	0.207725621	0.218721247	0.098307931	0.045884818	0.028843498	0.017580341	0.024559912
WGC106576DB	0.062443355	0.063386842	0.041417265	0.026970021	0.038980331	0.131411047	0.153534199	0.310806456	0.067656121	0.031956417	0.021326754	0.05011119
WGC106578D	0.214947669	0.104848114	0.082445713	0.036729476	0.024284862	0.075422994	0.047149647	0.270040339	0.039508252	0.024263035	0.047558647	0.032801252
WGC106580D	5.06E-36	0.082362385	0.049951228	0.100078989	0.011281799	0.458852959	0.229645891	7.81E-127	0.054686958	9.59E-37	0.001203208	0.011936583
WGC106582D	0.08454948	0.056760586	0.210815891	0.01486574	0.014870257	0.110300009	0.075882345	0.190809872	0.128482067	0.029131222	0.025121147	0.058411385
WGC106584D	0.147592902	0.121723931	0.106842909	0.06433232	0.013785514	0.098524136	0.06795595	0.180514458	0.092529664	0.022696152	0.026792836	0.056709228
WGC106586D	0.090952044	0.035941882	0.122482565	0.027503789	0.034569633	0.070583868	0.082825189	0.365981373	0.057534285	0.019490773	0.054761836	0.037372762
WGC106588D	0.087504998	0.048631087	0.088524264	0.026277778	0.23412284	0.08563077	0.103385899	0.090480297	0.154126892	0.021715932	0.021527651	0.038071591
WGC106592D	0.042496479	0.026826898	0.123996772	0.035038021	0.029127949	0.137842187	0.221917287	0.116659982	0.094170617	0.104509925	0.013363467	0.054050416
WGC106594D	0.049259443	0.064586546	0.170701295	0.035233945	0.013519399	0.012692086	0.119922122	0.182597358	0.133668249	0.026878175	0.12106875	0.069872633
WGC106596D	0.106773801	0.068052086	0.049062855	0.04366825	0.011319186	0.010607009	0.034489484	0.243381009	0.072774896	0.292466716	0.014844399	0.052560309
WGC106598D	0.283511362	0.068464767	0.098818878	0.020161358	0.032761606	0.075059425	0.024994212	0.239693206	0.048229431	0.022591073	0.041074525	0.044640155

WGC106600D	0.106740816	0.05866263	0.004985396	0.174854339	0.020516409	0.105465067	0.107455973	0.128668392	0.023642096	0.236072025	0.023035825	0.009901032
WGC106602D	0.171788973	0.066772768	0.178617829	0.034403153	0.007178419	0.10851326	0.128460701	0.137327035	0.078070282	0.021718119	0.033575122	0.033574339
WGC106606D	0.066431039	0.077410958	0.042503967	0.029275816	0.007262427	0.396460666	0.198638625	0.061981115	0.083708716	0.010708866	0.003221902	0.022395904
WGC106608D	0.194762116	0.12580317	0.061986815	0.023501067	0.017076794	0.117181304	0.059004613	0.270078029	0.028932453	0.032661787	0.038310531	0.030701322
WGC106610D	0.048593477	0.056111734	0.117932511	0.209917051	0.020944261	0.08835617	0.16738566	0.110476296	0.095434868	0.013364528	0.024096532	0.047386911
WGC106612D	0.06032151	0.054371131	0.102844458	0.192918114	0.023671596	0.057459395	0.146006185	0.107120709	0.103054408	0.016657198	0.072781398	0.062793899
WGC106614D	0.13875653	0.059173656	0.122620524	0.031072208	0.037394901	0.10710576	0.127718816	0.209593789	0.09293336	0.024250977	0.016364255	0.033015223
WGC106616D	0.167375252	0.088538186	0.023347291	0.064247646	0.010881277	0.29748048	0.177015871	0.050241749	0.080267396	0.013968376	0.010449014	0.01618746
WGC106618DB	0.080400849	0.073511396	0.110915947	0.045005355	0.009140208	0.156642688	0.236258799	0.096816032	0.106953198	0.012826422	0.018186466	0.05334264
WGC107774D	0.109201317	0.076643846	0.173478943	0.019015075	0.013580988	0.122339547	0.167744642	0.152992001	0.070027431	0.029180861	0.030446796	0.035348552
WGC107782D	0.107171083	0.061005515	0.160357198	0.026544799	0.019393224	0.064670179	0.075498391	0.193375658	0.15987502	0.016836118	0.048563692	0.066709124
WGC107786D	0.142789601	0.058833496	0.200392393	0.065418735	0.022170049	0.027665463	0.040738269	0.108457035	0.145536845	0.010431139	0.126642892	0.050924083
WGC107788D	0.031157593	0.058093131	0.15135661	0.197745745	0.022999999	0.033335763	0.078800406	0.228908749	0.125601919	0.013449944	0.017397035	0.041153106
WGC107790D	0.144424696	0.055090184	0.095996618	0.31201867	0.022499081	0.022017249	0.065228698	0.110921252	0.094653156	0.011966051	0.033683556	0.031500789
WGC107794D	0.052729853	0.064226236	0.041138665	0.046525811	0.005627156	0.224222184	0.167770244	0.075850626	0.073749834	0.114072241	0.114233879	0.01985327
WGC107798D	0.188385576	0.137159976	0.109411486	0.059381421	0.022689369	0.020030486	0.014694378	0.195860981	0.10733681	0.054865887	0.024353075	0.065830555
WGC107800D	0.106249074	0.061343628	0.179628477	0.050322273	0.021708962	0.056547075	0.071460526	0.21636552	0.083709418	0.063264622	0.028455728	0.060944697
WGC107802D	0.185698947	0.117661167	0.197972155	0.03654983	0.008752347	0.051536099	0.054315741	0.212132779	0.047843352	0.019987559	0.014066167	0.053483856
WGC107806D	0.048867396	0.042814014	0.273677177	0.005277305	0.050364394	0.024697104	0.04233282	0.141458922	0.245103059	0.015865661	0.008514472	0.101027676
WGC107808D	0.17042929	0.16439423	0.150401739	0.055108267	0.025534162	0.004211566	0.026257481	0.230888049	0.053987456	0.039008638	0.038169127	0.041609994
WGC107812D	0.167234513	0.059413666	0.175715921	0.030138303	0.014830477	0.073635396	0.061942529	0.156630611	0.147933997	0.016434319	0.014555692	0.081534577
WGC107814D	0.042739292	0.033573268	0.111776798	0.279959416	0.012078426	0.094992535	0.21093024	0.073317945	0.075855142	0.000686734	0.025708979	0.038381225
WGC107816D	0.192170747	0.064580097	0.083523102	0.045871732	0.020837465	0.134651813	0.087683213	0.17196755	0.101469506	0.037546252	0.01502454	0.044673983
WGC107820D	0.382883733	0.052481472	0.060820756	1.15E-118	0.027335017	0.068804848	0.013788106	0.253376303	0.034751431	0.048437922	0.044005314	0.013315098
WGC107822D	0.210883938	0.047785493	0.18417591	0.043439521	0.050183254	0.025313635	0.028820837	0.266299208	0.067747474	0.008900643	0.032789497	0.033660591
WGC107824D	0.050735426	0.080128851	0.132895875	0.035333208	0.046317069	0.263376623	0.173492404	0.027505113	0.136692852	0.014022844	6.31E-185	0.039499735
WGC107826D	0.100651992	0.060734299	0.231202226	0.077238944	0.012668946	0.011048275	0.038818724	0.187399781	0.171302971	0.019278964	0.017774631	0.071880247
WGC107828D	0.115043268	1.24E-155	0.018593861	1.72E-32	0.008496627	0.525678222	0.160566831	0.115033805	0.006405314	0.012784657	0.021620026	0.015777391
WGC107830D	0.109907703	0.075269459	0.338801286	0.031954602	0.014588192	0.047347503	0.052302897	0.118696925	0.080540483	0.027355877	0.044496658	0.058738416

WGC107832D	0.122157772	0.052925047	0.175438644	0.035352491	0.072830916	0.01194516	0.022355986	0.158994338	0.203423329	0.022013316	0.056148348	0.066414652
WGC107834D	0.114520179	0.047380242	0.088214742	0.038134273	0.015422229	0.046913366	0.097339238	0.198153818	0.20673626	0.013478868	0.030056451	0.103650334
WGC107836D	0.026330695	0.012153699	0.034230195	5.56E-07	0.004425345	0.305876982	0.438764655	0.079007982	0.030893997	0.023664005	0.019394352	0.025257537
WGC107838D	0.149053447	0.076181276	0.197203543	0.030442735	0.055268287	0.125001298	0.102618351	0.106826035	0.066638131	0.0211427	0.028774407	0.04084979
WGC107840D	0.020411777	0.022770603	0.12278878	0.005751032	0.011417196	0.170158419	0.410619838	0.091201946	0.082829976	0.018775137	0.004249208	0.039026089
WGC107842D	0.131590579	0.202051711	0.043102085	0.046398373	0.017686084	0.015434056	0.035314801	0.329602964	0.068835894	0.025024776	0.030856429	0.054102248
WGC107844D	0.055616477	0.051050122	0.036310317	0.044492061	0.010784534	0.199127756	0.203440233	0.142540945	0.167491454	0.025502444	0.02088741	0.042756246
WGC107846D	0.062659263	0.078885211	0.107122501	0.01457163	0.005505239	0.459707806	0.19218694	1.38E-21	0.045374574	0.004727123	0.010968543	0.018291172
WGC107848D	0.125415838	0.069653676	0.122749977	0.044633081	0.014862347	0.090511063	0.099974384	0.230321292	0.115219511	0.017012894	0.019032964	0.050612972
WGC107850D	0.008605819	1.02E-64	0.118524565	0.149354021	0.035776192	0.011579264	0.086045787	0.457300418	0.041460876	4.362599652<br>77821e-321	0.043957776	0.047395282
WGC107852D	0.167970632	0.092654326	0.123427764	0.037578985	0.009243454	0.068357039	0.065318599	0.200558036	0.14058732	0.021817684	0.01800225	0.054483911
WGC107854D	0.177236723	0.045212115	0.098137709	0.03723435	0.014984623	0.146538205	0.110937325	0.153712191	0.06628839	0.086912676	0.020204759	0.042600935
WGC107856D	0.066009196	0.042605709	0.149890678	0.220092265	0.02067716	0.027334674	0.096533554	0.132235325	0.093770119	0.048984367	0.05111318	0.050753774
WGC107858D	0.119661645	0.131842348	0.159766964	0.045144175	0.017564013	0.017569485	0.048980117	0.241676155	0.112214393	0.021469614	0.0297761	0.054334991
WGC107860D	0.237673809	0.073404058	0.080798709	0.004784245	0.033255884	0.109814359	0.038228961	0.199422296	0.11162561	0.03172785	0.027873589	0.05139063
WGC107862D	0.08580393	0.051710329	0.195809877	0.14155812	0.025445442	0.042503251	0.083410969	0.107460985	0.14323375	0.02223356	0.039450989	0.0613788
WGC107864D	0.121210705	0.053065455	0.08009045	0.115235871	0.02023433	0.153744381	0.089869177	0.173483992	0.078011377	0.025326496	0.04027365	0.049454114
WGC107866D	0.083292619	0.026184393	0.054842491	0.488090388	0.033225517	5.390256196<br>128e-321	0.084144219	0.117995606	0.055823714	0.00115106	0.029493718	0.025756274
WGC107868D	0.111452363	0.069773553	0.115386307	0.063705177	0.015994548	0.135191331	0.179187183	0.091952998	0.129162938	0.010317249	0.020561439	0.057314914
WGC107870D	0.13633777	0.06028028	0.125649046	0.036945616	0.018515984	0.137063859	0.083874229	0.192611349	0.112593268	0.020782057	0.022926668	0.052419875
WGC107872D	0.015979072	0.049299153	0.22067637	0.101243735	0.014901093	0.117425101	0.129226886	0.158347105	0.12336029	0.01115396	0.012833014	0.04555422
WGC107874D	0.20269902	0.086918003	0.194927195	0.051010886	0.017782413	0.068204626	0.054341032	0.071341525	0.073879123	0.100020092	0.036038872	0.042837213
WGC108520D	0.064646418	0.041908865	0.077984641	0.393689152	0.021398753	0.101541154	0.157943207	5.01E-33	0.060460546	0.012263747	0.036468678	0.031694839
WGC108522D	0.053286031	0.105381589	0.066098149	0.18675826	0.028894841	0.00648921	0.061145446	0.369607888	0.068539262	0.011864217	0.023147572	0.018787535
WGC108526D	0.073543864	0.067385445	0.03056783	0.136408963	0.045739486	0.00392675	0.031533292	0.472496249	0.051848194	0.017395175	0.008106867	0.061047886
WGC108528D	0.087163403	0.056277986	0.283730542	0.019779784	0.011020929	0.034983603	0.062665662	0.144887981	0.142296746	0.03776853	0.059599304	0.05982553
WGC108530D	0.118100251	0.040022724	0.12251457	0.01981084	0.011471311	0.096094405	0.095766937	0.150698574	0.100868264	0.021419081	0.143514888	0.079718156

WGC108532D	0.152920553	0.051241133	0.256977598	0.07286749	0.018273082	0.065816017	0.080426027	0.110218931	0.073140737	0.030989544	0.033019881	0.054109007
WGC108536D	0.085670101	0.066272925	0.159655549	0.070979845	0.007774534	0.024807439	0.08145306	0.254267783	0.137025425	0.013165074	0.036942294	0.061985971
WGC108538D	0.228420764	0.071503342	0.126920965	0.040022257	0.018044486	0.071754496	0.049650551	0.142888472	0.098585955	0.056536785	0.02298836	0.072683567
WGC108542D	0.116408171	0.087738082	0.072636764	0.098750586	0.032114204	0.032211894	0.054519673	0.211786017	0.150204673	0.043750397	0.040986443	0.058893096
WGC108544D	0.155268146	0.089873179	0.076296228	0.017421849	0.016739492	0.196020348	0.087355409	0.176347748	0.064681774	0.039136143	0.053828363	0.02703132
WGC108546D	0.214115667	0.071590283	0.151306943	0.028846913	0.006440284	0.058610691	0.052246263	0.195878563	0.11593381	0.014708457	0.041002953	0.049319173
WGC108548D	0.070401663	0.064536875	0.052263822	0.128013578	0.020810133	0.227738749	0.27385078	0.041602719	0.067458412	0.015080757	0.015644333	0.02259818
WGC108550D	0.13913035	0.068753089	0.087430756	0.054143771	0.067678035	0.088442321	0.134741975	0.036015383	0.209152062	0.01634363	0.066005893	0.032162736
WGC108552D	0.149376778	0.038934541	0.086105836	0.164310371	0.041313789	0.098041942	0.094555882	0.175380674	0.086461551	0.017083693	0.022835628	0.025599315
WGC108554D	0.198729665	0.072000785	0.115221971	0.011035376	0.015217712	0.109037423	0.052466632	0.162799782	0.067645612	0.039026803	0.050366194	0.106452045
WGC108556D	0.063193173	0.040881307	0.241094709	0.114437278	0.016033612	0.016814963	0.077399274	0.172324756	0.147363345	0.01846386	0.016629339	0.075364384
WGC108558D	0.095783267	0.06246987	0.109123883	0.062633787	0.049363623	0.135514767	0.138501212	0.181802136	0.085314517	0.008043735	0.041881266	0.029567937
WGC108560D	0.058014467	0.08196576	0.043369388	0.04418684	0.006101916	0.443327205	0.230748589	0.030372156	0.035487684	0.00767081	0.003691704	0.015063482
WGC108564D	0.072339165	0.167306007	0.193632228	0.030204235	0.015672415	0.036480577	0.048820108	0.267059508	0.08019822	0.01798473	0.027862626	0.042440181
WGC108566D	0.079904839	0.075338593	0.119635876	0.054503063	0.012259502	0.133204089	0.216507321	0.12909781	0.112231822	0.013178354	0.020445687	0.033693043
WGC108568D	0.211784169	0.066130991	0.062816962	0.00170943	0.025791986	0.041248335	0.035118815	0.311382368	0.083644473	0.098938399	0.029248067	0.032186003
WGC108570D	0.022330522	0.003771162	0.065470436	7.54E-64	0.006246274	0.388431383	0.426645252	0.019202875	0.025991164	0.004270416	0.005313168	0.032327349
WGC108572D	0.21682023	0.100815676	0.063146136	0.013459859	0.014885708	0.117258226	0.041927146	0.281299795	0.044331368	0.035298379	0.037187357	0.033570118
WGC108574D	0.202009433	0.073767451	0.190914409	0.047637531	0.029094053	0.025520072	0.022472052	0.11465412	0.129916232	0.035810184	0.023642301	0.104562163
WGC108576D	9.62E-31	0.060577029	0.084351612	0.332961286	0.048478021	0.016058899	0.073245495	0.205525702	0.113837021	0.011157561	0.011178835	0.042628539
WGC108578D	0.024149524	0.069942015	0.035815011	0.146667684	0.053545631	0.003277538	0.042833176	0.523626666	0.049726411	0.017558157	0.016569494	0.016288692
WGC108580D	0.103585685	0.053576434	0.188744512	0.019855623	0.005962652	0.214831151	0.160104721	0.083683245	0.092546736	0.034190498	0.006907573	0.036011169
WGC108582D	0.163635876	0.064291083	0.078833013	0.041635466	0.019009972	0.195764252	0.084217285	0.158722561	0.10260319	0.02690363	0.023543091	0.040840581
WGC108584D	0.313341654	0.064124359	0.074109412	0.072698908	0.022668984	0.133362579	0.041650335	0.183774312	0.019189498	0.020544149	0.047640607	0.006895202
WGC108586D	0.013351528	0.085047891	0.030921766	0.047068897	0.068853961	0.002101276	0.031219527	0.614070915	0.009194627	0.027287381	8.20E-199	0.070882232
WGC108590D	0.062851434	0.079777752	0.124717154	0.029446481	0.015628305	0.230245691	0.151656119	0.113568514	0.106005801	0.01281605	0.009304189	0.063982507
WGC108592D	0.116097842	0.046812613	0.119122554	0.152591112	0.021518668	0.00912934	0.044034024	0.316643197	0.059313299	0.030214386	0.030628264	0.053894702
WGC108596D	0.030627608	2.82E-149	0.112504457	0.476558563	0.006706126	0.027446145	0.124296783	0.116620289	0.019113708	5.958431688<br>84543e-321	0.066902695	0.019223626

WGC108600D	0.036384301	0.033171248	0.050415216	0.046571173	0.013385499	0.305903826	0.225021217	0.140925093	0.097306169	0.02149789	0.006741446	0.022676922
WGC108602D	0.058877621	0.037116747	0.074125378	0.010332433	0.008193998	0.30983538	0.289703273	0.056820852	0.101914511	0.011446411	0.006354072	0.035279324
WGC108604D	1.86E-05	2.50E-20	0.005534051	0.007096551	0.002539664	0.500801291	0.269716555	0.103967638	0.04783622	0.008148702	0.018247609	0.036093102
WGC108606D	0.131078653	0.066957461	0.161613402	0.061450198	0.016638524	0.128153903	0.107359633	0.135208546	0.095283626	0.019362347	0.026965811	0.049927896
WGC108608D	0.246534672	0.065182795	0.090845215	0.006647561	0.028020001	0.056080208	0.03693227	0.187389673	0.174396787	0.028817659	0.022903354	0.056249806
WGC108614D	0.093854994	0.067182489	0.122849398	0.075608244	0.020856935	0.164280308	0.111107451	0.165859931	0.095860718	0.019051408	0.018144239	0.045343884
WGC108620D	0.219007338	0.073530972	0.151534769	0.086700753	0.015693276	0.058235083	0.030654072	0.161979287	0.075178438	0.036778494	0.070822106	0.019885413
WGC108624D	0.186582332	0.049220616	0.112107442	0.089051871	0.015829196	0.023209325	0.038170593	0.210077247	0.13517372	0.023385355	0.074872073	0.042320232
WGC108815D	0.11065838	0.066748789	0.12741612	0.030667369	0.014522297	0.215051956	0.112891626	0.11525889	0.122319068	0.026095821	0.013608895	0.044760788
WGC108817D	0.15177256	0.043018205	0.073461274	0.005422242	0.017735569	0.231349902	0.199517896	0.171032334	0.019546064	0.009255508	0.05280664	0.025081805
WGC108819D	0.033337352	0.040567941	0.178506367	1.52E-07	0.031734015	0.022130084	0.069037287	0.365967772	0.180558025	0.019663475	0.008757571	0.049739958
WGC108825D	0.087519951	0.04944716	0.082093804	0.028088093	0.01768115	0.26705726	0.168258746	0.151697507	0.065237198	0.029170158	0.010482847	0.043266127
WGC108827D	0.108750058	0.075569404	0.235050152	0.087637006	0.027073551	0.022040689	0.042046728	0.168806252	0.114441125	0.027854672	0.035803375	0.054926988
WGC108831D	0.112185346	0.02803612	0.060244378	0.128758144	0.043249777	0.02474846	0.025070909	0.314254733	0.068395411	0.060890031	0.103066795	0.031099897
WGC108833D	0.147407022	0.074681795	0.128624412	0.065689004	0.037204726	0.07441136	0.07784702	0.213137919	0.077553433	0.020594968	0.041994501	0.04085384
WGC108835D	0.081012277	0.0561179	0.135281696	0.024413195	0.020394959	0.143863453	0.104587274	0.130394932	0.212844507	0.017746265	0.010757334	0.062586209
WGC108837D	0.13208913	0.081317772	0.144555197	0.106971701	0.177542958	0.022544198	0.04296242	0.07776417	0.142845346	0.011919522	0.023248751	0.036238836
WGC108841D	0.106623241	0.04850783	0.089312387	0.069983449	0.013847182	0.08133063	0.116071492	0.150925393	0.053808618	0.22432454	0.013140548	0.03212469
WGC108845D	0.12297645	0.055299953	0.172566402	0.132243709	0.073566832	0.017787579	0.050195277	0.109893615	0.093962211	0.094766463	0.0127148	0.06402671
WGC108847D	0.088727865	0.074253638	0.135459376	0.081034199	0.008470944	0.043829553	0.055527263	0.084895211	0.109409581	0.007395085	0.268314055	0.04268323
WGC108849D	0.058404604	0.035079301	0.157033447	0.046328129	0.009485332	0.183269952	0.212569426	0.097795516	0.08152488	0.059510587	0.008590009	0.050408817
WGC108851D	0.108533635	0.066336594	0.153230486	0.059505981	0.023949087	0.096236571	0.101059405	0.172557238	0.125676084	0.019708747	0.010558261	0.062647911
WGC108853D	0.044102683	0.049268692	0.136612714	0.37702627	0.016111612	0.075462234	0.118183521	0.083579819	0.037389715	0.004661221	0.031089453	0.026512064
WGC108855D	0.085002902	0.026661041	0.078463992	0.039061441	0.015108915	0.294975516	0.1729097	0.155452768	0.046384888	0.024776518	0.020226826	0.040975493
WGC108857D	0.060814256	0.05042548	0.155711418	0.061221121	0.038580417	0.157328147	0.229390097	0.05006229	0.126887448	0.014419037	0.012383056	0.042777232
WGC108859D	0.235887128	0.099404093	0.096730096	0.097385807	0.025388837	0.067115472	0.024463794	0.106328999	0.125899359	0.067219895	0.01120961	0.04296691
WGC108861D	0.048658371	0.055996697	0.035830537	0.04166565	0.054468207	0.001735598	0.035909527	0.581796926	0.076292731	0.012255788	0.016598626	0.038791341
WGC108863D	0.082007977	0.080996923	0.120377494	0.189644623	0.020334928	0.099591939	0.097901513	0.039808254	0.161789654	0.019204539	0.019720013	0.068622144
WGC108865D	0.119425266	0.059195539	0.162808351	0.074338216	0.035763976	0.09638941	0.098224045	0.165905495	0.053254387	0.025890607	0.06693735	0.041867356

WGC108867D	0.147519341	0.070955726	0.152671015	0.058840769	0.066067281	0.159755146	0.098121748	0.060011773	0.113027715	0.024138596	0.014682331	0.03420856
WGC108869D	6.57E-121	0.01188179	0.139141065	0.421705233	0.024672627	0.02049841	0.114952101	0.167888721	0.048033467	0.003677625	0.017906489	0.029642471
WGC108871D	0.056962849	0.106152433	0.138554211	0.086251329	0.010502231	0.103641096	0.172757738	0.135056083	0.09520016	0.028982877	0.015783816	0.050155177
WGC108873D	0.020599089	0.041044549	0.299874165	0.101040471	0.010114097	0.095563426	0.137882743	0.105859334	0.108223026	0.013167877	0.020821541	0.04580968
WGC108877D	0.178892401	0.079569506	0.099808006	0.060976062	0.011412742	0.10934092	0.069226114	0.180196415	0.113674483	0.031751089	0.026937046	0.038215218
WGC108879D	0.103459884	0.056192452	0.1265608	0.072698386	0.007956938	0.117159141	0.243259253	0.117648718	0.083996621	0.026597479	0.003378939	0.04109139
WGC108881D	0.145883144	0.055718154	0.199837893	0.100386809	0.025063081	0.026544773	0.04341905	0.256248977	0.066727663	0.014091761	0.025500644	0.040578052
WGC108883D	0.097503599	0.05989632	0.153641351	0.032731848	0.090003806	0.021313723	0.055605625	0.153191009	0.167985841	0.110474753	0.020005482	0.037646643
WGC108885D	0.36866475	0.04754975	0.046657819	0.022062103	0.046814945	0.073863584	0.005508775	0.244560837	0.04755576	0.028689588	0.032448329	0.03562376
WGC108889D	0.087327791	0.065725973	0.117774755	0.038642611	0.136213464	0.15840884	0.148807024	0.058116328	0.106302231	0.022807371	0.012786202	0.047087411
WGC108891D	0.1056159	0.096662063	0.161832681	0.057262929	0.027628838	0.052543983	0.082486935	0.085173859	0.158995768	0.014521728	0.076374881	0.080900435
WGC108893D	0.099162978	0.051853262	0.22738244	0.082923624	0.021968419	0.027925628	0.047320605	0.185748559	0.162027844	0.021305693	0.026619398	0.04576155
WGC108895D	0.266474348	0.082202702	0.158531407	0.021546317	0.022385828	0.037373433	0.018864772	0.18686472	0.085543548	0.050190176	0.032319862	0.037702887
WGC108897D	0.063549283	0.061434018	0.104033983	0.047830476	0.024641282	0.110173671	0.082139487	0.320772894	0.114979624	0.01833835	0.008966953	0.04313998
WGC108899D	0.175279314	0.083723486	0.114168956	0.059115349	0.014775208	0.056125519	0.090887166	0.213290536	0.081021782	0.045139331	0.028981804	0.03749155
WGC108901D	0.09397718	0.022956416	0.1139332	0.102865714	0.009834145	0.247669331	0.190258023	0.070235535	0.062706189	0.01280258	0.031887073	0.040874615
WGC108903D	0.154720555	0.073957369	0.108418549	0.018933004	0.043534317	0.152906112	0.101405997	0.154568558	0.110739144	0.032531242	0.013808688	0.034476465
WGC108905D	0.079987532	0.04870304	0.067752851	0.072074035	0.006520562	0.082748879	0.225901104	0.139820759	0.106450755	0.106533762	0.005735755	0.057770966
WGC108907D	0.197301916	0.064456753	0.198966904	0.05218793	0.008438959	0.091401236	0.084436923	0.118659223	0.085562282	0.021386467	0.029767073	0.047434333
WGC108909D	0.070492375	0.074069869	0.120611134	0.040626539	0.007626389	0.236085359	0.233152094	0.070215031	0.082656645	0.017738928	0.015594731	0.031130905
WGC108911	0.106342813	0.043572877	0.212494595	0.097353695	0.020556132	0.0257831	0.064173399	0.207969229	0.123067062	0.01562269	0.037371172	0.045693237
WGC108913	0.097336766	0.080026539	0.131172228	0.084921159	0.014219946	0.164664782	0.177418957	0.095858488	0.084688458	0.009838367	0.019847856	0.040006453
WGC108915	0.085057759	0.036461057	0.122972029	0.133666907	0.029319641	0.043030954	0.06878081	0.263403078	0.119291053	0.010664021	0.038262533	0.049090159
WGC108917	0.13894357	0.080550824	0.26684213	0.047638344	0.036985905	0.052358662	0.053311616	0.150598885	0.074200611	0.026865521	0.029582695	0.042121238
WGC108919	0.087612463	0.049280256	0.07797121	0.11111371	0.02713165	0.207375408	0.157693546	0.10789656	0.102777815	0.020759239	0.0133717	0.037016444
WGC108921	0.210977176	0.039039532	0.201252543	0.032307824	0.017163015	0.164113127	0.102059087	0.138249492	0.020591732	0.003773424	0.038984816	0.031488234
WGC109742	0.177355055	0.089719434	0.06208709	0.057795148	0.022679846	0.120688686	0.089939935	0.089196721	0.114473613	0.091999083	0.019745569	0.06431982
WGC109744	0.343835939	0.066092297	0.004949886	0.063373216	0.029286736	0.112687588	0.055392396	0.088642489	0.036315408	0.141908578	0.016212775	0.041302692
WGC109746	0.196547459	0.068665075	0.13164614	0.036627475	0.020008901	0.051198859	0.061510961	0.112840206	0.019082597	0.03780685	0.243013114	0.021052363

WGC109748	0.126522334	0.074173207	0.191727803	0.076889313	0.014317627	0.060885527	0.046169565	0.048933889	0.145455226	0.009587036	0.147453774	0.057884699
WGC109750	0.096149897	0.073533444	0.181470289	0.076993947	0.032060663	0.022006736	0.053793484	0.151604028	0.168993933	0.000803603	0.065429224	0.077160752
WGC109752	0.114733292	0.03602825	0.052832078	0.030775901	0.008908964	0.313677646	0.152392806	0.173451534	0.046550875	0.019025253	0.018272503	0.033350898
WGC109754	0.098455872	0.052118644	0.152874877	0.101740103	0.042019735	0.058720594	0.083939452	0.124034094	0.126565154	0.080878183	0.013838929	0.064814363
WGC109756	0.042220682	0.068152558	0.114906024	0.077823244	0.024191153	0.080080875	0.13919743	0.185664449	0.144560568	0.013630235	0.04898874	0.060584042
WGC109758	0.088373037	0.029459047	0.006532158	0.007899501	0.074710831	0.007792633	0.02956532	0.645215698	0.04825998	0.00959676	4.44E-55	0.052595035
WGC109760	1.10E-137	0.022894333	1.26E-57	0.015522489	1.953925600<br>29229e-312	0.539117649	0.383188899	4.10E-166	0.031743719	6.62E-230	1.016668523<br>38628e-318	0.007532911
WGC109762	0.165761486	0.092044993	0.118878361	0.063815381	0.00970826	0.123814579	0.064708291	0.156046726	0.106874252	0.021667846	0.025362002	0.051317822
WGC109764	0.142426855	0.057034871	0.140207463	0.268794338	0.015050355	0.027266642	0.105739852	0.080293626	0.07678226	0.013583794	0.034086266	0.038733677
WGC109770	0.057974085	0.131627925	0.078081564	0.014839073	0.004203857	0.245541991	0.258470183	0.088241497	0.066912469	0.005901767	0.017963297	0.030242292
WGC109774	0.276917583	0.079710439	0.079620239	0.088677709	0.026531869	0.034948454	0.02502125	0.191445022	0.085546018	0.031129782	0.029124048	0.051327587
WGC109776	0.162709455	0.078788157	0.128802326	0.052328664	0.131933161	0.054280637	0.040511467	0.139462138	0.114023479	0.010163025	0.042757462	0.044240029
WGC109778	0.010186092	0.014368349	0.089717334	0.229592205	0.024247824	0.006770757	0.060572465	0.383525631	0.138687483	3.819127442<br>35284e-321	0.012497445	0.029834415
WGC109780	0.058475076	0.061420819	0.152448505	0.085972997	0.023607589	0.069792187	0.109091023	0.144451578	0.149519055	0.01290334	0.073501833	0.058815996
WGC109782	0.093976602	0.060014642	0.161924086	0.080551319	0.015669975	0.14951753	0.148340423	0.058447693	0.099562942	0.063258781	0.01793748	0.050798527
WGC109784	0.191423966	0.032192968	0.135008794	0.041835553	0.012167384	0.046351496	0.047357621	0.303155784	0.070539525	0.026425689	0.017075846	0.076465374
WGC109786	0.280638121	0.064661827	0.118354406	0.083893479	0.025370192	0.042660174	0.023676579	0.170299779	0.05633399	0.065867262	0.02993267	0.038311522
WGC109788	0.074892793	0.102940366	0.083225873	0.034117598	0.010716926	0.314453512	0.143352706	0.059357602	0.071826757	0.019376682	0.033975939	0.051763247
WGC109790	0.142945308	0.060157932	0.172739139	0.043897009	0.009800724	0.091944846	0.125124118	0.157425744	0.103215592	0.021787801	0.018353738	0.052608047
WGC109792	0.06154287	0.071342544	0.07463899	0.014779286	0.010247337	0.359544136	0.284659737	0.006111614	0.075231725	0.006843256	0.005236489	0.029822016
WGC109794	0.11569114	0.06431139	0.303630941	0.069403459	0.008717092	0.025495546	0.04556193	0.164243055	0.103901977	0.022871543	0.014334218	0.061837708
WGC109796	0.071948103	0.033749763	0.047381066	0.047092366	0.014514187	0.323298722	0.180652739	0.105441171	0.090951065	0.031729829	0.007209964	0.046031026
WGC109798	0.180767333	0.095566351	0.090834138	0.083395563	0.010474918	0.167796849	0.13024084	0.088078427	0.078395572	0.030209043	0.003747688	0.040493279
WGC109800	0.089158996	0.053923869	0.147168201	0.270252276	0.049007337	0.008921414	0.074546123	0.143986594	0.075876208	0.008533101	0.027957103	0.050668776
WGC109802	0.087818675	0.076486206	0.135837321	0.310750964	0.022285781	0.070915725	0.119779369	0.006619619	0.075759813	0.012102601	0.04732089	0.034323035
WGC109804	0.08665638	0.061047951	0.118491166	0.153206462	0.02550071	0.072832176	0.076658651	0.077742434	0.093329908	0.036806239	0.143115773	0.05461215
WGC109806	0.077214197	0.052121426	0.257046952	0.106618801	0.015364119	0.066471747	0.08295802	0.110122173	0.144923283	0.015326291	0.008581315	0.063251675

WGC109808	0.132439154	0.029900887	0.072315199	0.053286991	0.007858453	0.214335452	0.174152485	0.097330379	0.071223798	0.041251635	0.067270524	0.038635043
WGC109810	0.051569368	0.054431621	0.08159405	0.023929581	0.00390367	0.275722585	0.367125203	0.055637399	0.020139698	0.0204977	0.016154212	0.029294911
WGC109812	0.097425533	0.052080802	0.130242292	0.044143142	0.015250819	0.159212888	0.216470847	0.105784131	0.098363028	0.018259848	0.012818169	0.0499485
WGC109814	0.172559101	0.051682173	0.092701602	0.109077966	0.021705976	0.113889404	0.078053319	0.170614721	0.088092602	0.033784064	0.022921175	0.044917897
WGC109816	0.069531493	0.082855772	0.094005628	0.032085662	0.007459031	0.329001249	0.291156622	2.30E-58	0.064417474	0.002601536	0.005934323	0.02095121
WGC109818	0.05592325	0.009621396	0.065091132	0.028163253	0.008130967	0.328801847	0.367383295	0.023599872	0.049343572	0.021333265	0.015016652	0.0275915
WGC109820	0.075780987	0.042291773	0.095353572	0.146339625	0.00689402	0.208643576	0.183140361	0.102983742	0.044707694	0.045946061	0.016324526	0.031594064
WGC109824	0.135616609	0.023076264	0.124993527	0.06989635	0.009682474	0.142096694	0.133653779	0.206895689	0.038922483	0.02310533	0.074792428	0.017268372
WGC109828	0.21085796	0.081045443	0.130730073	0.039338182	0.02205676	0.026399136	0.032868321	0.232534734	0.103470243	0.030934969	0.031817074	0.057947106
WGC109830	0.069764516	0.064654927	0.010081535	0.058079964	0.013781314	0.25430997	0.195691595	0.129887629	0.073858031	0.052705407	0.043383763	0.03380135
WGC109832	0.406219905	0.086795534	0.064946459	0.025299423	0.021113202	0.113824297	0.018445175	0.13496328	0.046871285	0.038429429	0.020632686	0.022459326
WGC109836	0.116275764	0.067861031	0.164154425	0.102658478	0.038395829	0.091821913	0.100876717	0.112973677	0.114098741	0.021453007	0.018381481	0.051048939
WGC109840	0.139832595	0.085667443	0.104145959	0.048876774	0.013012638	0.12961208	0.102797134	0.149421484	0.131387791	0.027468706	0.015044444	0.052732952
WGC109842	0.136755091	0.090923197	0.114505995	0.053430642	0.01293265	0.051287952	0.061240892	0.201759292	0.108383085	0.033750113	0.024438997	0.110592093
WGC109844	0.339312011	0.076310572	0.081790473	0.057347792	0.026563572	0.064388254	0.007847873	0.198604197	0.052459117	0.020254595	0.043144888	0.031976656
WGC109846	0.135955527	0.057925295	0.058120969	0.11948038	0.034253439	0.020671983	0.028986983	0.188586099	0.196354263	0.042327843	0.016676002	0.100661217
WGC109848	0.117322484	0.067311781	0.096117285	0.071088452	0.014164408	0.130583735	0.16243699	0.144010073	0.091107743	0.047615559	0.021571247	0.036670243
WGC109850	0.13605813	0.077594008	0.095036555	0.123963013	0.022623121	0.077178705	0.090157736	0.197257235	0.115822532	0.015757246	0.01575258	0.032799138
WGC109852	0.049515201	0.076162923	0.148700749	0.051278386	0.010758735	0.133853692	0.151058649	0.129848617	0.147093048	0.020756636	0.012008432	0.068964932
WGC109854	4.25E-29	0.025647462	0.066036739	0.054195718	0.00489162	0.017830488	0.105598826	0.029286113	0.06282617	0.002105642	0.614922186	0.016659036
WGC109856	0.121113065	0.072243224	0.212910877	0.037278632	0.083922326	0.048045446	0.069823065	0.193575354	0.090593702	0.013396185	0.020287534	0.03681059
WGC109858	0.097155762	0.011753212	0.040792516	0.024840322	0.00471961	0.347549479	0.339130472	0.081751103	0.002363123	0.017291121	0.014821835	0.017831444
WGC110899	0.129731794	0.05091639	0.070048189	0.12983765	0.036701696	0.128049367	0.095193741	0.188884247	0.070582345	0.018632508	0.034879383	0.04654269
WGC110903	0.128337077	0.121622668	0.120609789	0.052968099	0.013011999	0.030723211	0.040211719	0.234829203	0.120214873	0.032671606	0.020552264	0.084247491
WGC110907	0.117849234	0.050786632	0.075318581	0.026886416	0.014041153	0.16782213	0.113457889	0.196219565	0.089068043	0.061042614	0.019142774	0.06836497
WGC110909	0.217301126	0.080056018	0.104808022	0.023858767	0.005823515	0.072720766	0.053408015	0.109515393	0.040549121	0.248108147	0.02565613	0.018194978
WGC110911	0.089520268	0.066905132	0.115182642	0.15583489	0.050517471	0.00861064	0.039815251	0.331097854	0.088232576	0.011787141	0.029382757	0.013113377
WGC110915	0.075159766	0.04369508	0.104440249	0.023832828	0.010695517	0.192421024	0.183721704	0.127499144	0.137626863	0.013477665	0.026915358	0.060514801
WGC110917	1.22E-270	2.56E-70	4.856665298	0.02620985	1.73E-26	0.165239127	0.117480763	5.50E-112	1.88E-53	0.023499233	2.88E-64	0.667571026

			61945e-321									
WGC110919	0.000168968	0.034848612	0.138775064	0.032122417	0.004699533	0.134757303	0.38090803	0.061845222	0.130910828	0.019196127	0.006397184	0.055370712
WGC110921D	0.150872103	0.102676427	0.082287895	0.022057354	0.012960974	0.142886925	0.102656814	0.194463504	0.075634581	0.038111421	0.034238214	0.041153789
WGC110923D	0.046167328	0.05063332	0.053768991	0.007804826	0.006374615	0.49347518	0.270329539	1.22E-28	0.045928946	0.002590379	0.002308075	0.020618801
WGC110925D	0.309848008	0.085023224	0.069628394	0.033826446	0.019273796	0.107902309	0.068463388	0.091978906	0.065169225	0.098514381	0.012730952	0.037640971
WGC110927D	0.03835413	0.057513056	0.11516967	0.025684893	0.042618654	0.037013871	0.29146281	0.14139774	0.143235986	0.026343453	0.01800531	0.063200428
WGC110929D	0.09381437	0.066076962	0.16098871	6.50E-17	0.015023582	0.09509042	0.119690969	0.228142716	0.114644577	0.011397525	0.017426217	0.077703951
WGC110933D	0.251990019	0.086029195	0.139560056	0.02403684	0.021772778	0.032205269	0.00769441	0.234883258	0.082778273	0.046382764	0.029413568	0.043253571
WGC110935D	0.071935367	0.078362469	0.085365403	0.018077176	0.005573174	0.15657043	0.265626491	0.122560812	0.089715865	0.009794454	0.061855853	0.034562507
WGC110937D	0.087089564	0.042202969	0.220147796	0.054387302	0.185927253	0.02850562	0.070544337	0.088619559	0.159214302	0.013631206	0.005059244	0.044670848
WGC110939D	0.104498898	0.038037792	0.225346483	0.048339209	0.020749535	0.076535129	0.129485021	0.1816926	0.104187016	0.027291438	0.022917365	0.020919515
WGC110941D	0.102600612	0.056533113	0.171050606	0.02337333	0.007475771	0.080528718	0.109379415	0.112225784	0.197687688	0.027316999	0.021695822	0.090132141
WGC110943D	0.117631396	0.101923962	0.130168127	0.063638102	0.246252917	0.038763235	0.039631569	0.083182882	0.123199436	0.002821508	0.015998675	0.036788191
WGC110945D	0.012975634	0.099974567	0.038985432	0.030000316	0.006048667	0.254541748	0.351176887	9.00E-244	0.150039122	0.012899853	0.009610777	0.033746997
WGC110947D	0.064099636	0.012741158	0.085019246	0.036736145	0.024583572	0.192621921	0.335877619	0.088408342	0.021375297	0.022464963	0.078341956	0.037730146
WGC110949D	0.102574377	0.041158769	0.179515257	0.184674133	0.008102408	0.061840067	0.138581396	0.102879742	0.07878194	0.036441756	0.03280266	0.032647495
WGC110951D	0.01718275	0.030342118	2.03E-96	0.009133796	0.041375904	0.06532661	0.061052217	0.078106863	0.144057747	0.029114341	0.002611279	0.521696376
WGC110953D	0.093175857	0.058899901	0.175730322	0.049128464	0.026132259	0.021142364	0.04180786	0.319762206	0.125114294	0.014897945	0.025440322	0.048768206
WGC110955D	0.093102527	0.089009102	0.067454663	0.006882902	0.010706639	0.309105703	0.24170319	0.10260893	0.017348809	0.021913576	0.016214517	0.023949441
WGC110957D	0.119595627	0.059280717	0.065273279	0.025810193	0.032720704	0.24415384	0.150761941	0.119495498	0.095375927	0.012966365	0.022065045	0.052500864
WGC110961D	0.204032251	0.076436787	0.15586915	0.043815189	0.074186684	0.057155696	0.053279906	0.174701386	0.07748934	0.020882775	0.023887083	0.038263753
WGC110963D	0.200392574	0.071548637	0.226518695	0.022898381	0.049734381	0.04187091	0.043734893	0.11067447	0.135035895	0.027104682	0.025436313	0.045050169
WGC110965D	0.06184709	0.067057683	0.22126661	0.033141847	0.014654929	0.076561818	0.116395834	0.125062136	0.184193784	0.021052105	0.013962296	0.064803868
WGC110967D	0.100749098	0.074213842	0.278997414	0.016544886	0.007405327	0.027956454	0.057630995	0.115228151	0.185933745	0.054687365	0.017497361	0.063155362
WGC110969D	0.087967342	0.043689548	0.007101408	0.011460974	0.072245166	1.59E-141	0.019518199	0.631071786	0.063254429	0.01345789	0.009305872	0.040927387
WGC110973D	0.151366163	0.071848062	0.139499092	0.052920662	0.029800995	0.055888747	0.067788337	0.223952396	0.088308564	0.014615211	0.046554544	0.057457227
WGC110975D	0.049004081	0.044988338	0.068761632	0.072250235	0.020255588	0.116881682	0.114962318	0.163501183	0.225358398	0.045900923	0.033918856	0.044216767
WGC110977D	0.027425946	0.071264952	0.087350371	0.037384058	0.008592557	0.254855965	0.259841635	0.04919294	0.154175999	0.005181001	1.74E-149	0.044734575
WGC110979D	0.107492297	0.075175988	0.182204018	0.06768491	0.099959793	0.052714499	0.075626309	0.103194977	0.140917253	0.028003082	0.009439126	0.057587749

WGC110981D	0.042631861	0.079100179	0.040024675	0.025192823	0.006283796	0.206480884	0.138542265	0.316200073	0.028257836	0.059561741	0.032546445	0.025177422
WGC110983D	0.085076244	0.055366585	0.147744292	0.010840657	0.013202379	0.224040487	0.204069213	0.07036932	0.114759646	0.01526918	0.010858295	0.048403702
WGC110985D	0.069481758	0.064809249	0.107660819	0.058375478	0.017360682	0.154586949	0.204413124	0.120557324	0.101552769	0.047642303	0.009569026	0.043990517
WGC110987D	0.234786292	0.071108043	0.05639784	0.046860155	0.021717294	0.23471372	0.106365625	0.099818017	0.066113385	0.027782944	0.014164381	0.020172303
WGC110989D	0.241018856	0.055657853	0.057745571	0.049015929	0.024142181	0.170204236	0.087374806	0.124212054	0.063233975	0.057207774	0.035040859	0.035145906
WGC110991D	0.079480631	0.07107246	0.282253867	0.059125068	0.011289134	0.086346331	0.100216769	0.076203774	0.139800345	0.035777176	0.016597322	0.041837124
WGC110997D	0.116736667	0.055015201	0.182485543	0.057800112	0.01919091	0.088355698	0.057463478	0.234816092	0.093996521	0.013745173	0.027232392	0.053162216
WGC111001D	0.050089254	0.074875679	0.127278048	0.046297995	0.007094758	0.200583221	0.313825572	0.037104856	0.095965265	0.011873812	0.002246565	0.032764976
WGC111003D	1.10E-137	0.009253056	0.005402618	1.16E-102	3.79E-23	0.434778294	0.304586315	8.26E-133	0.006068276	0.232342277	0.002484239	0.005084924
WGC111005D	0.179710151	0.079034942	0.109583228	0.042502199	0.023073005	0.080674162	0.048872559	0.157382694	0.198088075	0.015674133	0.036401488	0.029003364
WGC111007D	0.144973127	0.03847883	0.056481621	0.137702276	0.036089462	0.17255075	0.106903421	0.177409566	0.037165649	0.016221272	0.037290369	0.038733655
WGC111009D	0.119515709	0.042791451	0.090594005	0.299128004	0.072478461	0.043929204	0.075560643	0.094820084	0.079995678	0.006881889	0.037948177	0.036356695
WGC111011D	0.14332584	0.060250187	0.047129053	0.09984424	0.038381583	0.1814695	0.065831679	0.18178613	0.109463716	0.020688409	0.017282689	0.034546974
WGC111013D	0.171494537	0.064653231	0.140707237	0.08716302	0.023573823	0.017229315	0.029232762	0.16845763	0.115592511	0.073685514	0.014162188	0.094048233
WGC111015D	0.099068757	5.12E-36	1.976262583 36499e-321	3.29E-229	0.157795189	0.002940974	0.032929332	0.665829613	2.53E-37	0.027629058	0.007758508	0.00604857
WGC111825D	0.10174826	0.056977423	0.152446194	0.193049736	0.024104245	0.015297218	0.061963276	0.24553718	0.09258742	0.009250988	0.015103259	0.0319348
WGC111827D	0.047877883	0.028084301	0.064452029	0.08739231	0.012106903	0.218425817	0.234964827	0.151531244	0.10932331	0.014748864	0.006616903	0.024475608
WGC111833D	0.068703398	0.053354975	0.239541044	0.081223632	0.013777108	0.101417442	0.083408749	0.147427705	0.118762689	0.021303847	0.012329417	0.058749993
WGC111835D	0.296893536	0.0607456	0.048411751	0.01158813	0.017378479	0.077654429	0.018695879	0.189309516	0.007487872	0.195577471	0.066870222	0.009387115
WGC111839D	0.051713384	0.11022042	0.124428401	0.053934661	0.017871866	0.093386112	0.115640121	0.241503193	0.100290289	0.019430787	0.01724801	0.054332756
WGC111841D	0.155930163	0.088505248	0.081071413	0.062743966	0.010119929	0.242005007	0.167091245	0.024385591	0.11431409	0.0171817	0.013763187	0.02288846
WGC111843D	0.111948277	0.097049815	0.108273033	0.078878297	0.015517292	0.117581396	0.095956904	0.205955933	0.071608033	0.015877679	0.027166466	0.054186876
WGC111845D	0.270575757	0.096779386	0.117255537	0.034552943	0.023635635	0.142264753	0.045851962	0.129068148	0.036288784	0.037674829	0.042284248	0.023768017
WGC111853D	0.113636906	0.048457581	0.093396797	0.118693312	0.017408258	0.111583529	0.147023413	0.19978323	0.087239283	0.020767852	0.019516412	0.022493427
WGC111857D	1.40E-158	0.078087353	0.555712173	0.045823439	0.005781979	0.038029352	0.067084455	2.43E-67	0.125932422	0.022594213	0.005079084	0.05587553
WGC111859D	0.0703379	0.048183441	0.172674286	0.073418219	0.020924874	0.065965809	0.141125547	0.128724344	0.165349801	0.012124098	0.041534548	0.059637133
WGC111861D	0.293148187	0.068441941	0.107413082	0.023873493	0.058308261	0.037268287	0.009792309	0.181480967	0.102213613	0.028696736	0.032677491	0.056685634
WGC111867D	0.139418865	0.077763595	0.194324736	0.051080394	0.028576995	0.037421118	0.046826846	0.197961136	0.141945965	0.020712631	0.02333501	0.040632709

WGC111869D	0.160143188	0.059153794	0.180021748	0.042692209	0.01339468	0.134614257	0.097626928	0.111699606	0.073900851	0.043578307	0.037669594	0.045504839
WGC111871D	0.139620048	0.08427344	0.12396992	0.055440854	0.019125212	0.04449892	0.09490976	0.127341334	0.114772176	0.049363817	0.04461353	0.102070989
WGC111873D	0.30127335	0.103272132	0.068600763	0.017392182	0.030463374	0.023458901	0.002578619	0.25486749	0.017693342	0.11209408	0.044865734	0.023440034
WGC111881D	0.025983608	0.040044408	0.044441572	0.107088248	0.04773738	0.000493127	0.057484815	0.508046589	0.123459763	0.013771033	0.005296887	0.026152572
WGC111883D	0.034422188	0.035917321	0.179632151	0.12772009	0.026596913	0.024719226	0.081429588	0.224689664	0.178877677	0.013543841	0.013153859	0.059297483
WGC111885D	5.19E-151	0.042490773	0.069817551	0.23350402	0.041603067	0.00743671	0.068840734	0.390463025	0.081744571	3.03E-05	0.019454842	0.044614408
WGC111887D	0.109486701	0.141595062	0.148565056	0.043743201	0.015300449	0.053245895	0.080949789	0.245793268	0.05842998	0.035946466	0.032305871	0.034638263
WGC111889D	0.123857006	0.079922509	0.005157931	0.01803229	0.014640289	0.292477523	0.132987636	0.137097292	0.139343928	0.024976921	9.01E-05	0.031416589
WGC111891D	0.09889165	0.075172686	0.086919633	0.099942777	0.011020556	0.170415091	0.193522294	0.129011493	0.085151781	0.013878144	0.005382958	0.030690936
WGC111895D	0.076240091	0.060111283	0.251706673	0.046610444	0.00871479	0.086750636	0.128709264	0.11488502	0.127443774	0.038326087	0.013828762	0.046673176
WGC111897D	0.080290268	0.041446133	0.103820856	0.031141011	0.02286977	0.158142307	0.206401519	0.161178576	0.092682407	0.028522824	0.011705885	0.061798443
WGC111899D	0.086984461	0.019686287	0.045663653	4.55E-71	0.019071177	0.250000467	0.246846234	0.159305402	0.068006932	0.012617682	0.053499115	0.038318591
WGC111901D	0.123475048	0.128055907	0.146512055	0.033912016	0.011439798	0.020250208	0.045050166	0.255634257	0.041836105	0.061323812	0.038473681	0.094036946
WGC111909D	0.030097956	1.04E-298	0.039795725	1.46E-31	0.005349163	0.516878077	0.276440737	0.053573745	0.030461987	0.010144217	0.013716108	0.023542285
WGC111911D	0.135965691	0.17324239	0.131517092	0.027956575	0.022417746	0.077464508	0.078572938	0.165361799	0.077328014	0.031353196	0.018246279	0.060573772
WGC111917D	0.296230552	0.050104293	0.10062806	0.013117879	0.016683676	0.192711346	0.098108481	0.134238144	0.015339873	0.014782002	0.041187916	0.026867778
WGC111919D	0.032915771	0.053676181	0.17710379	0.059457792	0.007454762	0.094575483	0.214218683	0.080864017	0.148418734	0.020833808	0.008516706	0.101964271
WGC111921D	0.153893791	0.082882955	0.132758425	0.114044938	0.09033236	0.066583039	0.063619113	0.130891108	0.076501037	0.024788713	0.023617339	0.04008718
WGC111923D	0.21272972	0.079107855	0.11683794	0.067459932	0.020276205	0.177474398	0.098333424	0.092419398	0.053598759	0.014585506	0.038286942	0.02888992
WGC111925D	0.182701918	0.080467241	0.097334068	0.019290795	0.074558422	0.230288211	0.122073373	0.063334448	0.077530168	0.013109369	0.016198847	0.02311314
WGC111927D	0.099621691	0.062491358	0.136650139	0.042610803	0.014871725	0.061720554	0.060017507	0.186518489	0.225709602	0.017771449	0.021860866	0.070155818
WGC111929D	0.134017254	0.06670747	0.20470212	0.042313116	0.01077909	0.050207821	0.059859786	0.182519964	0.125448349	0.039566512	0.016163046	0.067715473
WGC111931D	0.113981013	0.141523043	0.209345189	0.029254003	0.007626894	0.02965976	0.043466208	0.185015007	0.124236282	0.037608714	0.028371075	0.049912812
WGC111933D	0.23405081	0.093825298	0.073152507	0.032499301	0.025869855	0.072717586	0.044194888	0.22031103	0.102762008	0.024491083	0.026555646	0.049569989
WGC111935D	0.014969821	2.045431773<br>78276e-321	0.011395307	0.0129944	0.022353243	0.298579702	0.321149427	0.087035257	0.042902189	0.017516672	0.145752966	0.025351014
WGC111937D	0.139622534	0.129224693	0.187957141	0.048133908	0.014106642	0.01441136	0.035015468	0.251189756	0.075199037	0.014463093	0.027940368	0.062736
WGC111939D	0.051901436	0.049333726	0.138896873	0.053613253	0.018988762	0.045208455	0.103315262	0.292657853	0.124057957	0.015195518	0.022745695	0.084085209
WGC111941D	0.14593443	0.095296572	0.190009635	0.039528795	0.018605611	0.057899116	0.042164263	0.129924506	0.149484072	0.041040193	0.019156967	0.070955841

Table S2c. Mutational signature matrix of small insertion and deletion (ID).

Subtype	ID1	ID2	ID3	ID4	ID5	ID6	ID7	ID8	ID9
1:Del:C:0	1.51E-05	0.030497537	0.019600601	0.049085393	0.134634337	0.006730657	0.010819049	0.011687035	0.011476891
1:Del:C:1	3.47E-89	0.019396443	0.006292635	0.016718875	0.052796176	0.000139762	0.006937974	0.012508942	0.002775514
1:Del:C:2	0.001508885	0.012546849	0.004640473	7.03E-143	0.023411134	0.000110497	0.001859944	0.005846193	0.001248763
1:Del:C:3	0.000986461	0.009715889	0.001698796	2.76E-174	0.012085605	0.002823855	0.000153679	0.004301532	4.49E-252
1:Del:C:4	0.000349416	0.005127862	0.00093254	6.27463370218383e-322	0.006701937	0.006151351	1.57E-153	0.001266239	1.97570973971245e-312
1:Del:C:5	0.000392463	0.002303054	0.000602705	2.91498731046335e-322	0.005912868	0.02552296	2.02566914794911e-322	0.010554447	1.87744945419674e-322
1:Del:T:0	0.010649461	0.028576803	0.012799544	0.03421238	0.064933499	0.001629113	0.007318076	0.017497878	0.008519513
1:Del:T:1	0.003324811	0.028476158	0.003054652	0.00983576	0.02934033	0.000577155	0.000617301	0.007424384	0.003290224
1:Del:T:2	0.00367894	0.02726431	0.007184795	0.000281413	0.029764957	0.00346696	1.25E-69	0.010577053	0.000788897
1:Del:T:3	0.00163919	0.021675399	0.007140608	1.67982319586024e-322	0.031769036	0.009816546	3.63E-18	0.010135252	1.02E-260
1:Del:T:4	0.001935995	0.014905912	0.005435676	5.95E-296	0.020680421	0.02441428	2.21E-17	0.012025921	2.34E-143
1:Del:T:5	0.000149363	1.92E-22	0.010862176	3.74E-12	6.64E-15	0.652215855	1.64E-29	0.199508456	0.003419128
1:Ins:C:0	0.010113845	0.604694261	0.015540865	0.015626299	1.48E-11	0.014126525	8.46E-08	2.15E-09	2.93E-09
1:Ins:C:1	0	0	0	0	0	0	0	0	0
1:Ins:C:2	0	0	0	0	0	0	0	0	0
1:Ins:C:3	0	0	0	0	0	0	0	0	0
1:Ins:C:4	0	0	0	0	0	0	0	0	0
1:Ins:C:5	0	0	0	0	0	0	0	0	0
1:Ins:T:0	0.023644483	1.47E-12	0.02598936	1.11E-05	0.015731828	0.085828089	1.59E-09	0.576660124	0.057745679
1:Ins:T:1	0	0	0	0	0	0	0	0	0
1:Ins:T:2	0	0	0	0	0	0	0	0	0
1:Ins:T:3	0	0	0	0	0	0	0	0	0
1:Ins:T:4	0	0	0	0	0	0	0	0	0
1:Ins:T:5	0	0	0	0	0	0	0	0	0

2:Del:R:0	7.73E-210	3.22E-126	0.003851561	0.083992478	0.045858438	1.15E-21	7.41E-143	1.93E-213	0.026866542
2:Del:R:1	0.001889012	0.001607572	0.000655578	0.006970884	0.010844388	0.000772195	0.002237426	0.001288709	0.00193262
2:Del:R:2	0.001591781	0.004466154	0.000263364	0.000522992	0.001335752	0.000514997	7.34E-130	0.000654154	0.00074002
2:Del:R:3	0.002300233	0.001805024	6.47E-299	2.63E-127	0.000981325	0.004987868	0.000403437	9.09080788347894e-322	7.79E-05
2:Del:R:4	2.46E-141	4.10074486048235e-322	2.07507571253324e-322	1.38338380835549e-322	1.13635098543487e-322	0.031868008	0.001537146	5.43472210425371e-323	9.88131291682493e-323
2:Del:R:5	0.000422141	9.68368665848843e-322	4.32E-164	2.13E-157	3.26083326255223e-322	0.011603436	0.004345155	1.97626258336499e-322	1.14E-173
3:Del:R:0	0.002816772	1.76E-08	0.005448644	0.023016773	0.013640475	0.000733598	2.14E-89	0.001028638	0.008032755
3:Del:R:1	0.002888157	0.002252874	0.000231933	0.002326982	0.000612969	8.20E-06	5.99E-30	0.000356267	0.000856108
3:Del:R:2	0.001771151	0.000647675	0.000413785	9.80E-228	0.000618169	0.003771033	0.00090333	2.76676761671098e-322	5.59E-100
3:Del:R:3	0.00185565	7.11454530011395e-322	3.55727265005698e-322	1.48219693752374e-322	2.56914135837448e-322	0.014725202	0.000359913	9.38724727098368e-323	1.63041663127611e-322
3:Del:R:4	9.80E-05	0.001316771	4.09086354756552e-321	2.44562494691417e-321	3.98216910548045e-321	0.001024254	0.000419768	0.000222972	1.44761234231485e-321
3:Del:R:5	0.000102263	0.00018297	3.24601129317699e-321	2.83E-132	0.000187816	0.000199109	0.000297878	0.000314053	0.000168096
4:Del:R:0	0.002770468	3.30E-114	0.004413079	0.010770631	0.009689124	0.000311561	2.58E-143	0.001366806	0.006703885
4:Del:R:1	0.001616622	0.003022664	0.000302108	0.002319158	0.004641547	0.000800997	0.001170361	0.001126721	0.000629757
4:Del:R:2	0.008959862	8.48E-92	0.001269091	0.000732768	9.45E-17	0.006468301	0.006319563	2.66795448754273e-322	0.001584164
4:Del:R:3	0.134738554	6.62E-89	0.004727146	0.003896767	9.38E-20	0.005287743	0.018467183	0.003017457	0.007485021
4:Del:R:4	0.203089221	0.010753764	0.011741905	1.83E-05	0.029147939	0.002228117	1.67E-16	2.10E-10	0.008351897
4:Del:R:5	0.042790276	2.45E-32	1.50E-17	3.66E-112	0.002797263	0.002976909	0.002228638	0.009392117	1.76E-58
5:Del:R:0	0.010981573	1.16E-14	0.072387644	0.052197446	0.060068985	5.10E-05	0.01049668	0.001286849	0.023157813
5:Del:R:1	3.29E-15	1.49E-56	0.007508751	0.037629412	0.037766323	0.004567474	0.272764595	9.09E-20	0.01204669
5:Del:R:2	0.076472317	1.57E-09	0.007024193	0.004086637	3.36E-39	0.007925261	0.208710266	0.001783392	1.68E-07

5:Del:R:3	0.075407547	1.12E-37	1.14E-24	1.57E-95	1.97E-83	0.002508443	0.092137612	2.91E-96	1.96E-64
5:Del:R:4	0.031303312	5.11E-81	0.00137806	5.07E-110	1.50E-93	0.003747956	0.032142439	0.001649983	4.86E-141
5:Del:R:5	0.015489824	0.00565871	0.001470965	9.67E-251	6.88E-13	0.002209914	0.009272965	0.002687476	3.37E-100
2:Ins:R:0	0.031300083	0.056082289	0.005276984	3.65E-59	0.048899922	0.01567011	1.17E-43	0.052307825	0.048630508
2:Ins:R:1	0.001498665	9.79E-88	0.000991445	0.000239337	0.000939159	8.24E-37	0.001131702	0.00059497	0.002318737
2:Ins:R:2	2.71E-115	2.23910550695253e-320	5.24697715883404e-321	5.70151755300799e-321	0.000147438	6.6204796542727e-321	0.000234213	5.12346074737373e-321	8.79E-05
2:Ins:R:3	5.76E-06	5.03270088823269e-319	0	0	1.15E-05	8.54E-06	1.88140197936347e-320	2.53E-25	2.47E-06
2:Ins:R:4	5.00192059849678e-320	1.39E-74	1.95E-06	1.84350714432744e-319	1.31381936542104e-319	2.64E-06	3.45E-06	4.05054779086488e-319	7.14E-06
2:Ins:R:5	2.35E-160	1.57E-180	7.46533190866124e-321	4.58986984986518e-321	0.000163752	1.68426978667281e-320	4.53E-05	2.27517229909894e-320	7.055257422613e-321
3:Ins:R:0	0.010684621	0.020995249	0.005880451	0.007724283	0.008226804	0.00117629	0.00533464	0.003947799	0.047645896
3:Ins:R:1	0.000434701	7.65307685408091e-321	0.000291595	1.97132192690657e-321	0.000616256	3.98E-05	0.000341519	0.000409334	0.000532299
3:Ins:R:2	2.64522746783403e-320	2.80E-05	0	3.00589538929814e-320	1.21357344587985e-319	4.38878513200779e-320	1.50E-05	3.35668199784543e-320	1.15621242439769e-319
3:Ins:R:3	0	0	0	0	0	0	0	0	0
3:Ins:R:4	1.96E-06	0	0	0	1.45383756945245e-319	0	2.29E-06	1.21382047870277e-319	0
3:Ins:R:5	0	0	0	0	0	0	0	0	0
4:Ins:R:0	0.040296478	0.030944302	0.006799617	1.98E-189	0.025108019	0.005647637	1.50E-15	0.01598647	0.053277996
4:Ins:R:1	0.00019049	0.000846202	4.30331177527726e-321	5.12346074737373e-321	0.000343538	3.05826634775732e-321	5.66E-223	2.4159810081637e-321	0.000189816
4:Ins:R:2	0	1.03E-05	2.03E-254	2.49063432725031e-319	0	3.23E-06	1.42740505739995e-319	1.58530843781081e-319	0
4:Ins:R:3	0	0	0	0	0	0	0	0	0
4:Ins:R:4	0	0	0	0	0	0	0	0	0

4:Ins:R:5	3.92337529362534e-320	4.7625457930867e-319	0	8.83092935376644e-320	1.25591487172845e-319	7.04E-07	5.92E-06	1.05151991404393e-319	0
5:Ins:R:0	0.10400824	2.00E-25	0.058098168	0.024754602	1.01E-07	0.009196277	0.093743163	4.32E-14	0.57122504
5:Ins:R:1	0.001160533	1.30E-65	7.19E-110	1.54E-150	0.00064736	1.78E-121	0.000413492	2.51E-60	0.000641631
5:Ins:R:2	0	0	0	4.14619889989974e-320	4.06E-06	0	2.62793517022959e-320	1.45E-05	4.98E-06
5:Ins:R:3	6.19E-07	2.46454766114989e-319	0	5.34084963154388e-320	6.80723646840069e-320	2.15644832440329e-319	3.53849815551501e-320	5.91E-06	7.29E-06
5:Ins:R:4	0	0	1.10E-276	9.01E-06	0	0	0	0	0
5:Ins:R:5	0	0	0	0	0	0	0	0	0
2:Del:M:1	0.008864498	4.22E-147	0.000286856	0.071160901	0.0596133	0.004339377	0.012586449	0.001493853	0.011468658
3:Del:M:1	1.10E-122	1.22034214522788e-321	0.003796809	0.021183176	0.010563195	0.000110871	0.002482134	1.01E-165	0.003897592
3:Del:M:2	0.005084995	0.010323664	0.004641599	0.021415058	0.021303936	0.003653703	0.004324594	0.006709155	0.003336655
4:Del:M:1	0.006792265	7.09E-75	0.004454356	0.013754712	0.00862151	0.00138711	3.29E-29	0.003254685	0.002247039
4:Del:M:2	0.002595913	0.008209412	0.005515985	0.01207857	0.015017674	0.001331743	0.000895866	0.002804779	0.00176495
4:Del:M:3	0.0083928	1.61E-55	0.004442942	0.013432482	0.015132685	0.003500353	0.003133956	0.00568164	0.004367182
5:Del:M:1	0.017314173	0.010763664	0.118454903	0.021934866	0.065243375	0.002115537	0.016812906	7.03E-06	0.007766776
5:Del:M:2	0.008036999	0.008614792	0.196538899	3.51E-20	0.036282308	0.001014705	0.000129622	0.000613018	0.004100462
5:Del:M:3	0.009177026	0.016287403	0.158934324	0.019419285	0.023770011	0.001747196	0.006798951	5.06E-39	0.003646232
5:Del:M:4	7.25E-05	3.72E-194	0.088767775	0.03542827	0.013391487	0.000749164	4.33E-34	3.16E-96	0.004998073
5:Del:M:5	0.066343568	2.90E-17	0.091962104	0.383212982	2.53E-26	0.005459843	0.159644354	5.08E-19	0.03993655

Table S2d. Abundance fractions matrix of small insertion and deletion (ID).

SampleID	ID1	ID2	ID3	ID4	ID5	ID6	ID7	ID8	ID9
FP1705100059DN01	0.257269709	0.00153828	0.020517281	0.143250148	0.103497626	1.01777523043 297e-321	0.341643624	0.01736993	0.114913402
FP1705100061DN01	0.316780337	0.010885222	0.021154149	0.075398515	0.037193476	0.137943363	0.207233021	0.050821001	0.142590917
FP1705100065DN01	0.197439546	0.024620688	0.041027537	0.131167628	0.069618189	0.092909592	0.08887174	0.215310859	0.139034219
FP1705100067DN01	0.26319297	0.008186209	0.008368983	0.192560939	0.037350927	0.004946604	0.322356526	0.029683379	0.133353462
FP1705100071DN01	0.19913226	0.006347362	0.216021708	0.122650865	0.095399935	0.012593888	0.180957249	0.017606024	0.149290708
FP1705100073DN01	0.190898334	0.006445698	0.228060256	0.129947423	0.093911185	0.002995956	0.207048727	0.038277733	0.102414687
FP1705100077DN01	0.25741525	0.008813001	0.013698917	0.158698966	0.041325409	0.039041897	0.180094559	0.121893936	0.179018065
FP1705100079DN01	0.181380638	0.010412234	0.237808756	0.065680791	0.158933226	0.025995634	0.183325912	0.039690369	0.096772439
FP1705100081DN01	0.18641258	0.014303994	0.029996215	0.148807906	0.035059207	0.07236117	0.300701177	0.115316671	0.09704108
FP1705100089DN01	0.15741683	0.029503829	0.035560625	0.188980539	0.111480119	0.018964322	0.115566373	0.117242669	0.225284695
FP1705100091DN01	0.293829896	0.015417427	0.041988555	0.089240695	0.041688282	0.042212125	0.261443834	0.107045467	0.107133719
FP1705100093DN01	0.19523655	0.024237722	0.021740484	0.098926853	0.071983559	0.046324737	0.238078174	0.083577272	0.219894649
FP1705100095DN01	0.13934677	0.024533611	0.053037407	0.116956703	0.106727846	1.26974870981 2e-321	0.327166757	0.067189743	0.165041163
FP1705100097DN01	0.105827555	0.010344481	2.53E-59	0.207796984	0.111530588	0.005399991	0.278032257	0.002087311	0.278980834
FP1705100099DN01	0.127962215	1.83792420252 944e-321	0.553438307	0.06658105	0.029492405	0.025726305	0.125412235	0.000660398	0.070727085
FP1705100101DN01	0.13644128	0.012108272	2.71E-210	0.207128855	0.130901019	0.080288157	0.23239625	0.053479462	0.147256703
FP1705100103DN01	0.279091422	0.008881484	0.034100378	0.145065708	0.056499548	0.02360155	0.228086968	0.101762994	0.122909948
FP1705100109DN01	0.104411277	0.02544417	0.03609827	0.175513896	0.121725555	0.080008155	0.19753142	0.055224024	0.204043233
FP1705100111DN01	0.206265294	0.011545102	1.37E-190	0.114506289	0.103764674	0.135877679	0.14067145	0.066706461	0.220663051
FP1705100113DN01	0.356363426	0.009135147	0.018177451	0.134941097	0.068746443	0.125015974	0.100588929	0.085386254	0.101645277
FP1705100115DN01	0.337913363	0.007525986	0.009830067	0.136195778	0.084278828	0.00505133	0.240905034	0.059863016	0.118436599
FP1705100117DN01	0.295286732	0.017144944	0.052354975	0.100595473	0.110454776	0.072482914	0.1533135	0.067697481	0.130669205
FP1705100119DN01	0.312640959	0.013174365	0.009259613	0.070063962	0.05338261	0.245691518	0.160880043	0.082738788	0.052168141
FP1705100121DN01	0.042156272	0.022534853	0.037222728	0.103994677	0.162297279	0.264896092	0.050069434	0.194074737	0.122753928

FP1705100123DN01	0.405658198	0.017029349	0.019538113	0.160549804	0.002705672	1.12152901605963e-321	0.172307562	0.007260762	0.214950539
FP1705100125DN01	0.127070183	0.037896355	7.00E-120	0.243755265	0.229945385	1.46E-102	0.117152364	0.057376528	0.18680392
FP1705100127LD02	0.39390477	0.01651559	0.021478681	0.049486351	0.123277068	0.118944882	0.144459029	0.104783343	0.027150286
FP1705100129DN01	0.058424433	0.023629119	0.209497256	0.136205338	0.18186334	0.031666729	0.130057138	0.102995303	0.125661345
FP1705100131DN01	0.305704801	0.020716947	0.03202276	0.100155433	0.110713483	0.103204467	0.144580769	0.060558487	0.122342854
FP1705100133DN01	0.143232311	0.020229981	0.012904669	0.191950934	0.148822859	0.00766048	0.242454172	0.022258676	0.210485918
FP1705100135DN01	0.183648264	0.007339865	0.015223047	0.101076497	0.201874497	0.12899313	0.144286382	0.1197199	0.097838418
FP1705100137DN01	0.239786328	0.021892488	0.030992217	0.072751931	0.083582913	0.159505651	0.071279954	0.178378671	0.141829847
FP1705100139LD02	0.283000748	0.025706634	0.044304251	0.055093245	0.080141977	0.016733321	0.296766804	0.148237515	0.050015504
FP1705100141DN01	0.324119143	0.042919254	0.067621871	0.150148422	0.109391213	0.021190206	0.069996002	0.047907545	0.166706343
FP1705100143DN01	0.129105689	0.019652986	0.040551323	0.100048428	0.132399818	0.252995654	0.024961443	0.175205152	0.125079508
FP1705100145DN01	0.261263471	0.023090949	0.02086315	0.137489347	0.117477993	0.094231137	0.100820012	0.114084644	0.130679299
FP1705100149DN01	0.310643261	0.027317377	0.00154876	0.094695726	0.147808963	0.001597968	0.114044838	0.187751297	0.11459181
FP1705100153DN01	0.211076156	0.01731212	0.329688596	0.079256166	0.14317908	0.02869689	0.052158806	0.064988486	0.0736437
FP1705100155DN01	0.404763266	0.025717161	0.02083395	0.168902683	0.140214215	0.035700399	0.070488231	0.058316317	0.075063777
FP1705100159DN01	0.167319952	0.023987806	0.040582284	0.149747393	0.15979797	0.032402854	0.106065748	0.189673346	0.130422646
FP1705100161LD02	0.347633771	0.010899089	0.03177013	0.112496601	0.078991619	0.076283933	0.290300637	0.051624221	1.57E-279
FP1705100163DN01	0.265638335	0.012479846	0.022186995	0.168527425	0.013145544	0.122956946	0.251952547	0.087801945	0.055310417
FP1705220177LD01	0.044811875	0.011709674	0.068312378	0.165258772	0.078444493	0.137119576	0.199646019	0.118348144	0.176349068
FP1705220179LD01	0.242062928	0.025559228	0.046504194	0.075249171	0.074187462	0.059997946	0.220983578	0.199555946	0.055899547
FP1705220181LD01	0.130405126	0.024320718	0.033334416	0.066421614	0.086978052	0.342996112	0.070749756	0.19659687	0.048197336
FP1705220182LD01	0.288215891	0.018875257	0.033559172	0.086896665	0.082261357	0.030651937	0.238888283	0.175575491	0.045075949
FP1705220184LD01	0.179393094	0.022051556	0.039026196	0.136218496	0.094900264	0.02135563	0.297892415	0.121406827	0.087755522
FP1705220185LD01	0.311510905	0.011005132	0.02987215	0.05865245	0.041610705	0.12024024	0.158717852	0.23210228	0.036288285
FP1705220186LD01	0.013607797	0.009013668	0.093885365	0.351457571	0.083462072	0.009321551	0.212893	0.014015096	0.212343882
FP1705220187LD01	0.151251123	0.010230404	0.07973744	0.186440478	0.024361053	0.090855901	0.279347034	0.12238171	0.055394857
FP1705220188LD01	0.202812019	0.027337964	0.04427365	0.072608031	0.069570892	0.141142474	0.222853318	0.189273526	0.030128127
FP1705220189LD01	0.230328816	0.012722254	0.285665067	0.115757463	0.081824955	0.043997727	0.214109982	0.012948989	0.002644749

FP1705220190LD01	0.499140914	0.01750009	0.016817963	0.115896475	0.095596091	0.00897783	0.186413707	0.05965693	5.56E-19
FP1705220191LD01	0.340047183	0.025369601	0.028020059	0.0998469	0.050802734	0.070974717	0.179133517	0.176677422	0.029127867
FP1705220192LD01	0.305936054	0.022212811	0.02124294	0.097540978	0.111320276	0.148545817	0.082751586	0.16091798	0.049531558
FP1705220195LD01	0.403271083	0.019454129	0.009583859	0.087715727	0.079529268	0.206504123	0.057619566	0.135455254	0.000866991
FP1705220196LD01	0.23579265	0.016990951	0.021734893	0.126121463	0.036418068	0.244910291	0.077383309	0.150249035	0.090399339
FP1705220197LD01	0.278381745	0.024171408	0.021381248	0.116232922	0.040645793	0.158675625	0.069189774	0.26563498	0.025686504
FP1705220198LD01	0.380282896	0.055296515	0.09755872	0.175765399	0.051428167	2.06E-304	0.076468574	0.076836972	0.086362757
FP1705220199LD01	0.160262289	0.101889225	0.040824964	0.098505762	0.154397105	0.088125002	0.116924544	0.138519221	0.100551889
FP1705220200LD01	6.71929278344095e-322	4.60E-141	1.5464254714831e-321	2.47032822920623e-321	1.73911107336119e-321	1	8.54733567305357e-322	7.90505033345994e-323	6.66988621885683e-322
FP1705220201LD01	0.469871034	0.008289015	0.030633604	0.165668879	0.02483259	0.00722454	0.219950062	0.016721114	0.056809162
FP1705220202LD01	0.242964054	0.033398425	0.027632538	0.071150038	0.132868023	0.015872776	0.151575751	0.049570925	0.274967469
FP1705220203LD01	0.272740477	0.02961197	0.070302596	0.073441086	0.060697096	0.086731304	0.093169508	0.149933296	0.163372667
FP1705220204LD01	0.312201366	0.019562737	0.049203365	0.130818557	0.083132297	0.073847821	0.11728349	0.131159211	0.082791156
FP1705220205LD01	0.173264717	0.021061775	0.056057168	0.091991657	0.156302479	0.239261408	0.10146995	0.088625202	0.071965644
FP1705220206LD01	0.267938002	0.019752378	0.0308591	0.027322876	0.097916445	0.289047115	0.074871508	0.146028283	0.046264295
FP1705220208LD01	0.02193125	0.034436685	0.049579289	0.103012813	0.119753914	0.02425826	0.141132343	0.168620028	0.337275417
FP1705220209LD01	0.027320374	0.028061453	0.098687303	0.137183262	0.091444245	0.048664497	0.134379586	0.090845011	0.343414269
FP1705220210LD01	0.12969866	0.000845486	0.666272312	0.008862988	1.63E-250	0.028196792	0.107813766	0.058309995	5.18768928133309e-322
FP1705220211LD01	0.548293951	0.010370449	0.033109302	0.022556011	0.05411022	0.00680788	0.246794691	0.01374706	0.064210436
FP1705220212LD01	0.355682517	0.024071001	0.019321381	0.038462079	0.095899824	3.81E-53	0.35399864	0.08446738	0.028097177
FP1705220213LD01	0.168032859	0.009342635	0.035510886	0.094516233	0.054936664	0.301679913	0.193690966	0.063863377	0.078426468
FP1705220214LD01	0.23010395	0.025129592	0.020727371	0.040279609	0.068584345	0.100603039	0.234899523	0.158143185	0.121529386
FP1705220215LD01	0.267275008	0.024980717	0.030564259	0.077760985	0.039464267	0.047351076	0.260616659	0.126436627	0.125550402
FP1705220216LD01	0.393409101	0.016080741	0.037240594	0.086937309	0.036134365	0.02750683	0.205890692	0.175223083	0.021577284
FP1706060153LD01	0.110318558	0.011826055	0.36814327	0.079227873	0.089872964	0.040352494	0.143912071	0.065202223	0.091144492
FP1706060155LD01	0.472287133	0.000632777	3.32E-69	0.169504799	1.15611361126852e-321	0.003875026	0.289321391	3.90311860214585e-322	0.064378874

FP1706060156LD01	0.062020856	0.018022051	1.00937611445367e-320	0.003361434	4.72820823070073e-321	0.669123715	0.034922693	0.202500727	0.010048525
FP1706060159LD01	0.181153708	0.085514501	0.051253227	0.119842244	0.089901895	0.080062086	0.163490892	0.167970399	0.060811048
FP1706060160LD01	0.498794037	0.005465712	0.03199793	0.135422449	0.09866877	0.011177177	0.14950164	0.059931019	0.009041266
FP1706060161LD01	0.271943327	0.018995168	0.038893618	0.087364528	0.03783217	0.153169091	0.193408123	0.142668878	0.055725097
FP1706060162LD01	0.441208254	0.005151648	0.026793969	0.083403663	0.086343002	0.040041554	0.231077541	0.042573622	0.043406747
FP1706060163LD01	0.628458467	0.005453743	0.018660191	0.071454298	0.036741527	0.003653288	0.210749324	5.97E-157	0.024829163
FP1706060165LD01	0.249222063	0.026091798	0.037788801	0.088590269	0.103711203	0.063113666	0.097624404	0.269867359	0.063990437
FP1706060167LD01	0.524206051	0.027765415	0.066123613	0.093264258	0.087473459	1.81E-182	0.145691517	0.044337	0.011138688
FP1706060169LD01	0.153433618	0.01984955	0.057158976	0.179447636	0.021199552	0.209193848	0.150468466	0.079707451	0.129540904
FP1706060170LD01	0.34722874	0.01499257	0.034495729	0.19754338	0.085950747	0.024714044	0.206302764	0.031252249	0.057519777
FP1706060171LD01	0.326267275	0.015236487	0.071040629	0.08503262	0.059971803	0.160796273	0.10577091	0.113074611	0.062809393
FP1706060172LD01	0.339503338	0.0201764	0.01918581	0.100806205	0.055209052	0.223603849	0.090735	0.129455497	0.021324849
FP1706060175LD01	0.262459895	0.031796434	0.046223742	0.164098027	0.029378619	0.159087003	0.090977775	0.168688839	0.047289666
FP1706060176LD01	0.358467971	0.015780235	0.044172555	0.176616095	0.048262305	0.035449715	0.190370583	0.101012578	0.029867963
FP1706060177LD01	0.314049058	0.017187547	0.053234435	0.100394752	0.053497309	0.081599833	0.137539026	0.200994709	0.041503331
FP1706060178LD01	0.413808466	0.013308827	0.021770283	0.10183032	0.106094074	0.041786307	0.093608451	0.100654422	0.10713885
FP1706060179LD01	0.283475245	0.001139156	0.02647457	0.166152399	0.048439509	1.06718179501709e-321	0.303470335	0.011457717	0.159391069
FP1706060180LD01	0.244194092	0.010745214	0.042739853	0.089425573	0.052931143	0.201003713	0.11251868	0.153125054	0.093316678
FP1706060184LD01	0.192399676	0.029511555	0.021709457	0.1109576	0.070258067	0.190635667	0.113474364	0.176333946	0.094719669
FP1706060185LD01	0.280774668	0.017498993	0.034422644	0.129443804	0.043522067	0.18286484	0.152409828	0.099799422	0.059263734
FP1706060188LD01	0.344876446	0.024462	0.054206896	0.069383955	0.07242375	0.167213072	0.053340582	0.177068689	0.037024609
FP1706060191LD01	0.23044179	0.029228339	0.027061126	0.117993341	0.08312577	0.054740198	0.094586114	0.196421744	0.166401577
FP1706060192LD01	0.400729857	0.020807477	0.016406015	0.155040226	0.023897035	0.046150684	0.08183515	0.165113318	0.090020237
FP1706060193LD01	0.421168401	0.009231143	0.028426189	0.128248209	0.013834272	0.071947828	0.111695759	0.109737311	0.105710887
FP1706060194LD01	0.604757868	0.004028433	0.016373651	0.146885625	2.46538757274782e-321	0.003467088	0.149017452	0.027660391	0.047809493
FP1706060195LD01	0.483625181	0.02680256	0.022936188	0.107270284	0.076793805	0.057091326	0.085661132	0.117404097	0.022415427

FP1706060196LD01	0.419010745	0.020804152	0.019765265	0.100618428	0.064368064	0.047142979	0.144294126	0.175720772	0.00827547
FP1706060197LD01	6.66988621885683e-322	2.28752394024497e-321	6.66E-155	1.21441335747778e-320	3.27E-247	0.985206841	0.00908734	0.005705819	1.66994188294341e-321
FP1706060198LD01	0.383174742	0.02457734	0.053599053	0.069166914	0.160264275	0.030788873	0.052292035	0.15915507	0.066981698
FP1706060199LD01	0.713985124	0.011252816	0.018843366	0.01757771	0.096671107	0.010237567	0.093156342	0.000546713	0.037729255
FP1706060200LD01	0.521098592	0.011323289	0.02568624	0.080848728	0.136197194	0.034589145	0.062288786	0.114651164	0.013316862
FP1706060201LD01	0.426185182	0.007372041	0.041120618	0.125442879	0.063549541	0.063156645	0.082063351	0.17070633	0.020403412
FP1706060202LD01	0.312980434	0.022717504	0.025335678	0.175670044	0.093777292	0.072399219	0.070003366	0.064807247	0.162309216
FP1706060203LD01	0.53396327	0.018092535	0.039136259	0.096843063	0.067201213	0.044744948	0.09984098	0.084352708	0.015825024
FP1706060204LD01	0.238392106	0.026476402	0.018685297	0.088584645	0.217498112	0.076423873	0.173284037	0.0920993	0.06855623
FP1706060205LD01	0.314435132	0.030187336	0.046213157	0.084042491	0.169170099	0.058225086	0.095898432	0.109777315	0.092050952
FP1706060206LD01	0.321994336	0.025548389	0.016620287	0.090459126	0.156633903	0.142071725	0.063150603	0.127224501	0.05629713
FP1706060207LD01	0.66136178	0.004258631	1.58E-66	0.031682568	0.082734804	0.012388902	0.116740446	0.026013812	0.064819058
FP1706060208LD01	0.520026813	0.024901892	0.037478704	1.34E-13	0.158298332	0.044168675	0.093554721	0.064918283	0.056652581
FP1706060209LD01	0.414128486	0.03064424	0.03351953	0.116631282	0.053607101	0.079481657	0.054538134	0.131751079	0.085698491
FP1706060210LD01	0.372968795	0.020334597	0.083737695	0.144623881	0.043693889	0.012253019	0.106366634	0.083494582	0.132526908
FP1706060212LD01	0.329035092	0.020198047	0.014818855	0.035039632	0.091885493	0.173032894	0.065649965	0.193043107	0.077296917
FP1706060213LD01	0.402237025	0.013019957	0.056426515	1.35E-36	0.190586491	0.13260403	0.12521289	0.074213835	0.005699256
FP1706060214LD01	0.223782027	0.025858701	0.024979018	0.051708243	0.045310536	0.408389557	0.045573056	0.153247602	0.021151259
FP1706060215LD01	0.512502764	0.020881516	0.011228684	0.08009587	0.152795338	0.067565638	0.052698672	0.080123121	0.022108397
FP1706060216LD01	0.386922878	0.014583414	0.024162145	0.005196858	0.09190553	0.187061792	0.051252945	0.228344438	0.010569999
FP1706060217LD01	0.30899869	0.034013441	0.112108071	0.016572536	0.260237914	0.144312156	0.008791881	0.100556242	0.014409068
FP1706060218LD01	0.16729898	0.008881234	0.018152402	0.009058849	0.102344424	0.551036296	0.023887716	0.099426391	0.019913708
FP1706060221LD01	0.42998629	0.032033147	1.32903658731295e-321	3.28E-156	0.147480051	0.225479245	0.131065758	0.031462522	0.002492987
FP1706060222LD01	0.317637471	0.02873493	0.049404842	0.0214628	0.182785588	0.11499805	0.1512433	0.080636656	0.053096364
FP1706060223LD01	0.323880159	0.012702312	0.016210556	0.024914142	0.111844528	0.350478094	0.041130715	0.10900551	0.009833985
FP1706060224LD01	0.38536132	0.025944622	0.013543272	8.99199475431069e-322	0.117327501	0.056052088	0.050605634	0.324170898	0.026994666

FP1706060225LD01	0.377661508	0.016106317	3.65E-59	6.76869934802 508e-322	0.111559343	0.279820402	0.052359517	0.128483451	0.034009462
FP1706060226LD01	0.334396238	0.016662369	0.021325361	0.017877603	0.137416979	0.175533393	0.063771519	0.196227266	0.036789273
FP1706060227LD01	0.364946056	0.025095823	1.34E-227	0.011478388	0.172861718	0.126838575	0.050054623	0.227732741	0.020992077
FP1706060228LD01	0.512237203	0.022863004	0.039338759	0.014380315	0.1353903	0.081809977	0.073775918	0.120204525	7.86E-135
FP1706060229LD01	0.414447974	0.040525604	0.051245455	0.01570795	0.102427047	0.004710976	0.095368276	0.25023453	0.025332188
FP1706060230LD01	0.416534205	0.021067297	0.027285589	0.042282265	0.175753501	0.107442305	0.017546049	0.163798088	0.028290701
FP1706060231LD01	0.351142453	0.025406474	0.048339141	0.024586782	0.153154365	0.115758748	0.030189079	0.216638171	0.034784787
FP1706060232LD01	0.429195452	0.035225502	0.041104967	0.044228468	0.162360712	0.083771949	0.029244521	0.159856994	0.015011434
FP1706060233LD01	0.46943146	0.026518822	0.014630275	0.00464229	0.137393846	0.147862691	0.039001319	0.115347384	0.045171914
FP1706060234LD01	0.433665594	0.015719253	4.98E-07	0.02169834	0.205108522	0.134119268	0.043583818	0.138407803	0.007696905
FP1706060235LD01	0.366738085	0.026534028	0.04327951	0.058345599	0.101197303	0.118371843	0.042556302	0.217729336	0.025247993
FP1707040176LD01	0.212475001	0.016324006	0.019089833	0.082002731	0.097635267	0.102130531	0.249173932	0.109168375	0.112000326
FP1707040177LD01	0.192327735	0.019209048	0.040430987	0.05137089	0.051069876	0.097048563	0.246425778	0.135775044	0.166342081
FP1707040178LD01	0.206005994	0.02123665	0.038358636	0.05561194	0.114533434	0.074414207	0.329331788	0.070175883	0.090331467
FP1707040179LD01	0.11840259	0.032865304	0.080589137	0.150101448	0.151625743	0.042284738	0.266357149	0.072733789	0.085040101
FP1707180231LD01	0.042015272	0.024884167	0.069766613	0.150655094	0.041456293	0.025422524	0.172115203	0.070448291	0.403236543
FP1707180232LD01	1.64E-185	0.0099403	0.093238901	0.266440589	0.104450283	0.003962871	0.134571238	0.041272724	0.346123094
TI1706140111LD02	0.258511134	0.047878696	0.013596329	0.037374646	0.103257592	0.192976322	0.002676905	0.27742932	0.066299055
TI1706140113LD03	0.286679946	0.030307024	0.052970275	0.11591797	0.121799255	0.090195741	0.043742347	0.165933424	0.092454018
TI1706140115LD02	0.142829352	0.00540509	0.602186968	0.026647879	0.125884003	0.033467182	0.008088046	0.036696209	0.01879527
TI1706140117LD01	0.367927637	0.040821521	0.009199131	0.078801651	0.158688283	0.051495095	0.006944746	0.197446974	0.088674962
TI1706140119LD01	0.078468885	0.015192782	0.578739304	0.018592182	0.093236525	0.055012354	0.003824971	0.094718944	0.062214054
TI1706140121LD01	0.326130753	0.052484579	5.50E-38	0.080516482	0.173354654	0.070847869	8.695555366 80594e-322	0.078423954	0.218241708
TI1706140123LD01	0.284666651	0.026351623	0.008488885	0.028713971	0.12826993	0.132126718	0.032698162	0.241820163	0.116863897
TI1706140125LD01	0.142746865	0.03450137	0.025928077	0.041854035	0.104119833	0.446825755	1.39E-179	0.126636927	0.07738714
TI1706140127LD01	0.253571489	0.01219107	0.362419924	0.04568073	0.196037516	0.008837106	1.180816893 56058e-321	0.014714004	0.106548162

TI1706140129LD02	0.264563969	0.095011792	0.01394272	0.096412796	0.13341893	0.17169542	8.42E-209	0.115784188	0.109170184
TI1706140131LD01	0.227628596	0.035830742	0.0951143	0.03118815	0.144044375	0.195330563	0.034601007	0.129113015	0.107149252
TI1706140133LD01	0.157548343	0.049875223	1.85E-249	0.044331281	0.279268038	0.123994527	0.014151024	0.240163777	0.090667785
TI1706140135LD01	0.163333739	0.044896501	0.011216136	0.030820889	0.157464367	0.212016934	0.001629702	0.282314135	0.096307598
TI1706140137LD02	0.163792601	0.027896501	0.026831806	0.02204015	0.205096711	0.229161695	0.003943146	0.2109614	0.11027599
TI1706140139LD01	0.050296718	0.029988167	0.124200153	0.024793805	0.463794999	0.064347615	0.009788576	0.073199437	0.159590529
TI1706140141LD01	0.188245023	0.050339465	0.025098634	0.082514962	0.272573968	0.13434254	0.010164399	0.139631332	0.097089677
TI1706140143LD01	0.255070173	0.05049552	0.001999114	0.095606581	0.205530553	0.087195213	9.10E-93	0.189115944	0.114986902
TI1706140145LD01	0.166486922	0.022550327	0.263940516	9.05E-05	0.3209795	0.034075353	0.002487975	0.044103045	0.145285849
TI1706140147LD01	0.177420309	0.043468475	3.78E-120	0.020448658	0.161001444	0.132905679	0.024980316	0.332030503	0.107744616
TI1706140149LD01	0.216013363	0.042903142	0.054868895	0.05309315	0.138913862	0.094898519	0.026218239	0.254101194	0.118989636
TI1706140151LD01	0.248816514	0.041101546	0.006445713	0.043998135	0.136181445	0.27786741	0.013961507	0.152618763	0.079008967
TI1706140153LD01	0.282295256	0.03863792	0.014925686	0.067785826	0.191821941	0.099132737	3.88E-50	0.163601142	0.141799493
TI1706140155LD01	0.264777747	0.050855486	0.051972944	0.070384045	0.256094673	0.080346662	0.011634951	0.055159818	0.158773674
TI1706140157LD01	0.249494103	0.035349756	0.011149226	0.10836285	0.11633209	0.142065288	0.017048129	0.215774013	0.104424546
TI1706140159LD01	0.129408548	0.030476856	0.321308594	0.012276893	0.14347808	0.144427206	0.020500039	0.134704609	0.063419175
TI1706140161LD01	0.10720239	0.041990419	0.009900262	0.03862113	0.127599942	0.324329048	0.010965181	0.214337109	0.125054519
TI1706140163LD01	0.17504981	0.031932395	0.039641112	0.037146002	0.213006047	0.239887581	0.023627532	0.153952622	0.085756898
TI1706140165LD01	0.326452965	0.033898984	0.078601126	0.070989498	0.193215012	0.125602674	8.12E-138	0.028252976	0.142986765
TI1706140167LD01	0.075518596	0.038357006	0.549103112	0.032687465	0.040574928	0.106978846	8.61E-96	0.095449634	0.061330413
TI1706140171LD01	0.125749105	0.02565413	0.035865594	0.04789139	0.12434854	0.378047329	1.57E-161	0.193695184	0.068748727
TI1706140173LD01	0.087769166	0.035334145	0.167075331	0.074606899	0.118319879	0.188930051	2.68E-90	0.201862547	0.126101983
TI1706140175LD01	0.185906886	0.049203999	0.039298153	0.054683175	0.209776542	0.131380684	0.017000722	0.208123322	0.104626518
TI1706140177LD01	0.101162016	0.031037869	0.076186353	0.025981035	0.273110257	0.260131503	0.043259088	0.114592319	0.07453956
TI1706140179LD01	0.103843757	0.063777359	0.006962537	0.018697012	0.098787149	0.26213175	0.006750781	0.377209331	0.061840324
TI1706140181LD01	0.283702624	0.040867275	0.027866702	0.036051859	0.160466839	0.135703225	1.378443151 89708e-321	0.193759322	0.121582155
TI1706140183LD01	0.111699301	0.045950392	0.035605553	0.035527846	0.140683638	0.356860422	0.017381995	0.178458598	0.077832254
TI1706140185LD01	0.2329733	0.018195755	0.080995987	0.011870472	0.167950377	0.147769273	0.004343502	0.18707386	0.148827474

TI1706140187LD01	0.250053947	0.061343033	0.017491714	0.041430456	0.173326466	0.129166983	0.022802928	0.224945792	0.079438681
TI1706140189LD01	0.303893109	0.039919039	0.018969784	0.093355201	0.178126073	0.020362792	0.011207779	0.210843	0.123323222
TI1706140191LD01	0.042230329	1.66E-280	0.927248686	6.18E-88	0.030520985	8.59674223763 769e-322	2.99E-248	8.102676591796 44e-322	1.89109973701 155e-315
TI1706140193LD01	0.261262905	0.042873463	0.045221478	0.04366329	0.221781492	0.07298642	0.017899752	0.149508939	0.144802261
TI1706140195LD01	0.554239044	0.017492677	1.58E-91	0.138589606	0.108692506	0.003817161	0.00125035	2.36E-302	0.175918655
TI1706140197LD01	0.388019056	0.033622349	0.025434819	0.100798778	0.151466872	2.91498731046 335e-322	1.50E-217	0.04455049	0.256107637
TI1706140199LD01	0.11465802	0.245950914	0.406146105	0.041300058	0.059171995	0.023413932	0.005561087	0.036317731	0.067480159
WGC106522DB	0.343586054	0.023427526	0.009693317	0.009964202	0.092074451	0.106067849	0.164936528	0.189424171	0.060825902
WGC106524D	0.271801423	0.029870758	0.014088295	0.048513821	0.033684903	0.084153827	0.233301839	0.116739946	0.167845189
WGC106526D	0.045890891	0.032032058	0.119739674	0.19653956	0.077043518	0.026752307	0.1352948	0.069735076	0.296972116
WGC106528D	0.227369092	0.012273734	0.042113344	0.047961728	0.049616348	0.087696619	0.20688017	0.062322489	0.263766476
WGC106530D	0.145775317	0.028371975	0.055910222	0.071037594	0.103676856	0.08273568	0.186318471	0.079678507	0.246495378
WGC106534D	0.159033643	0.042516994	0.024058027	0.019940436	0.011589472	0.059932016	0.12583495	0.111641488	0.445452973
WGC106536D	0.42236703	0.024113741	0.012589725	0.035862814	0.07954431	0.136062125	0.16507127	0.062276995	0.062111988
WGC106538D	0.258704122	0.01480631	0.027472469	0.021953963	0.071239734	0.182301161	0.107468678	0.12530487	0.190748693
WGC106540D	0.22707483	0.014785185	0.019737955	9.79E-07	0.038826126	0.116953542	0.181757112	0.078368167	0.322496104
WGC106542D	0.332268347	0.02405427	0.036052452	0.089304375	0.052076449	1.00E-14	0.203274619	0.025796188	0.2371733
WGC106544D	0.242490846	0.029599648	0.04211471	0.144504405	0.062140275	0.080165414	0.200451555	0.0773197	0.121213447
WGC106550D	7.97E-155	1.66994188294 341e-321	0.19642768	0.440741998	8.12E-137	5.43472210425 371e-322	0.017877831	1.378443151897 08e-321	0.344952491
WGC106552D	0.056769827	0.014077281	0.055987167	0.157149432	0.054782095	0.054138486	0.199551705	0.100776977	0.30676703
WGC106554D	0.456344541	0.011587897	0.026588841	0.071734256	0.105762018	0.021014765	0.113560328	0.061954759	0.131452596
WGC106556D	0.180710168	0.021954065	0.045953426	0.082406658	0.043857787	0.068033898	0.291368337	0.07945536	0.186260301
WGC106558D	0.304543943	0.024822878	0.034509724	0.075996081	0.040144302	0.00880866	0.206805586	0.026210737	0.278158089
WGC106560D	0.396465237	0.009094059	0.014596264	0.139927193	0.079235018	0.015001792	0.195893421	0.013052947	0.136734069
WGC106562D	0.070813338	0.012649757	0.061895985	0.095401682	0.085404818	0.076774061	0.216250169	0.055628234	0.325181956
WGC106564D	0.028586045	0.014898775	0.114046406	0.292402623	0.038574528	0.027171975	0.130030878	0.055002968	0.299285801

WGC106566D	0.269652104	0.016806508	0.04194688	0.008647094	0.125876666	0.199621617	0.119407057	0.110717637	0.107324437
WGC106568D	0.36818678	0.012960658	0.031242865	0.039184555	0.060952245	0.163512626	0.134294238	0.111531658	0.078134374
WGC106570D	0.218655287	0.022196284	0.02713612	0.068374932	0.052591151	0.169668492	0.112014471	0.194337703	0.13502556
WGC106572D	0.271300429	0.011314575	0.138678912	0.011489822	0.111965489	0.059968827	0.102144897	0.189065512	0.104071536
WGC106574D	0.233163275	0.023759769	0.009365038	0.024644901	0.079646229	0.176623556	0.124361552	0.064408278	0.264027401
WGC106576DB	0.453291642	0.007034533	4.49E-63	0.081653188	0.019257386	0.033944391	0.133587722	2.84E-281	0.271231139
WGC106578D	0.307511824	0.019792685	0.018286253	0.015446763	0.050482156	0.06813273	0.104137586	0.041128493	0.375081509
WGC106580D	0.013819	0.011082554	0.103695016	0.288065905	0.04103386	0.004063366	0.085487263	0.006507802	0.446245234
WGC106582D	0.221503797	0.032185303	0.028927406	0.031117911	0.120657226	0.072269063	0.135095986	0.128688239	0.22955507
WGC106584D	0.15437274	0.021696006	0.026237351	0.070140612	0.033484367	0.047037228	0.222930168	0.063485177	0.36061635
WGC106586D	0.357691919	0.021138509	0.00911734	0.052072096	7.80E-19	1.05730048210 027e-321	0.186243551	9.337840706399 56e-322	0.373736584
WGC106588D	0.199465698	0.018684641	0.010780872	5.47E-49	0.033872483	0.054532573	0.132073562	0.069780131	0.48081004
WGC106592D	0.261433913	0.026655722	0.01695242	0.024095955	0.032042899	0.119773223	0.197412158	0.097664371	0.223969339
WGC106594D	0.296767604	0.016611323	0.019673202	0.043322912	0.035516955	0.107430087	0.180408638	0.079120406	0.221148872
WGC106596D	0.229458423	0.022017921	0.034469279	0.035307199	0.073966019	0.008612788	0.2517359	0.064123132	0.28030934
WGC106598D	0.091854538	0.02170492	0.198661038	0.097448199	0.083376618	0.133625115	0.206731028	0.058802525	0.107796019
WGC106600D	2.17882949815 99e-321	0.003867479	0.255724846	0.472529927	1.49E-216	0.005007456	0.048078444	0.012457003	0.202334844
WGC106602D	0.227863864	0.011217108	0.391498878	0.041479839	0.053621412	0.048911859	0.100462408	0.064619451	0.060325181
WGC106606D	0.299879859	0.014002351	0.010014527	0.100797234	0.060690467	0.2362805	0.180812753	0.035681956	0.061840353
WGC106608D	0.176338113	0.019635373	0.082510581	0.151203451	0.107258554	0.000714324	0.234805902	0.093695401	0.1338383
WGC106610D	3.90E-34	0.000245539	0.183106498	0.41763524	1.46E-30	0.002124558	0.073984125	0.023202129	0.29970191
WGC106612D	0.000105001	2.36163378712 116e-321	0.175370213	0.34660979	4.70E-20	2.05531308669 959e-321	0.08576015	0.000878716	0.391276129
WGC106614D	0.153475324	0.03570453	0.053569179	0.062527945	0.122242108	0.066333453	0.145147006	0.16681525	0.194185204
WGC106616D	0.1689277	0.017837095	0.053974699	0.140073887	0.16167406	0.045301023	0.149752111	0.058592838	0.203866586
WGC106618DB	0.263062604	0.021914892	0.014895593	0.043764665	0.066105482	0.057385918	0.186845521	0.128096274	0.217929052
WGC107774D	0.250797922	0.025151667	0.048841124	0.057639579	0.122859605	0.06430391	0.106788867	0.100181322	0.223436003

WGC107782D	0.242017226	0.013518864	0.023875922	0.123214656	0.016544143	0.107484516	0.141786867	0.13800709	0.193550716
WGC107786D	0.200896468	0.019525378	0.036948312	0.076578327	0.112903497	0.025163586	0.22868226	0.054714591	0.24458758
WGC107788D	0.204233236	0.015036593	0.060791569	0.150352418	0.027316802	0.005978805	0.298609331	0.005211904	0.232469341
WGC107790D	0.010042737	0.001027633	0.180055762	0.386936192	0.060733523	0.00409708	0.077242546	9.05E-54	0.279864528
WGC107794D	0.151479964	0.029703971	0.033299327	0.153480396	0.10665683	0.061668923	0.247075595	0.067535448	0.149099546
WGC107798D	0.208104533	0.023236196	0.027946343	0.028062988	0.09759551	0.139731531	0.148009617	0.086471625	0.240841657
WGC107800D	0.187997114	0.040450914	0.059322647	0.125193938	0.096055618	0.040105768	0.235142958	0.054255718	0.161475324
WGC107802D	0.315166919	0.023041685	0.111067742	1.19069820647 74e-321	0.10898197	1.35454816100 161e-317	0.158769717	0.249351802	0.033620165
WGC107806D	0.22852971	0.041674088	0.03598678	0.048462739	0.145855367	0.107222501	0.143221262	0.204414159	0.044633394
WGC107808D	0.214676984	0.020184141	0.08538639	0.110716231	0.040543679	0.031971484	0.215701501	0.145112237	0.135707353
WGC107812D	0.143606177	0.015873937	0.04973086	0.089418634	0.123843668	0.114017408	0.200305106	0.167817956	0.095386254
WGC107814D	0.031924712	7.72E-63	0.189612477	0.438106696	0.051266184	0.005508385	0.070266945	0.013316938	0.199997664
WGC107816D	0.152826332	0.024263	0.060011408	0.134300556	0.100984046	0.076156472	0.241778983	0.062633259	0.147045944
WGC107820D	0.229374044	0.014047293	0.270191556	5.38531553966 959e-322	0.192326719	0.102439289	0.111318189	0.074165133	0.006137777
WGC107822D	0.408301988	0.018267375	0.048064686	0.142396696	0.112813935	1.95E-189	0.054672614	0.079023116	0.136459591
WGC107824D	0.291775124	0.038224033	0.018447296	0.030265243	0.081915726	0.146284285	0.057713611	0.282903718	0.052470966
WGC107826D	0.184808216	0.018087331	0.061867648	0.108550091	0.077778264	0.030670652	0.262421391	0.141414212	0.114402195
WGC107828D	0.241191308	0.028025549	0.192987887	1.30E-53	0.136234451	0.172218275	0.074478359	0.0213647	0.133499472
WGC107830D	0.251235087	0.033337138	0.025294492	0.02263482	0.120955728	0.087698764	0.058278752	0.35933027	0.041234948
WGC107832D	0.32176117	0.022547087	0.015715071	0.070035794	0.038560819	0.047797058	0.126458906	0.183817262	0.173306833
WGC107834D	0.205560326	0.016600674	0.055669715	0.071069375	0.081348343	0.030966303	0.234110957	0.067303354	0.237370952
WGC107836D	0.305658283	0.023941163	0.019273948	0.055398047	0.084839339	0.11089007	0.137759428	0.085015773	0.177223949
WGC107838D	0.21292481	0.028062368	0.10019534	0.061658153	0.019049101	0.141137467	0.11827518	0.160959306	0.157738274
WGC107840D	0.414940761	0.015977014	0.011205384	0.013062584	0.137241833	0.095589656	0.117575437	0.136350772	0.058056559
WGC107842D	0.295243172	0.012286456	0.045055655	0.157676716	0.010941614	0.011177543	0.262520835	0.05730588	0.14779213
WGC107844D	0.238628915	0.008583891	0.069080075	0.126091152	0.122147891	0.050696574	0.239589753	0.014796215	0.130385534
WGC107846D	0.205325157	0.019271085	0.040995209	0.109857672	0.10052919	0.10841902	0.185022893	0.069916243	0.160663531

WGC107848D	0.272642623	0.010703356	0.034143387	0.133710825	0.091864071	0.015610691	0.225609865	0.020664744	0.195050438
WGC107850D	0.268624233	0.005462913	0.054257354	0.163558788	0.059287767	1.72428910398595e-321	0.278400648	0.007853952	0.162554345
WGC107852D	0.244715561	0.015855448	0.037336617	0.046413583	0.077346898	0.046135448	0.155749626	0.082503755	0.293943064
WGC107854D	0.078736619	0.025810271	0.077480099	0.14999959	0.105046379	0.133229784	0.165995887	0.0987754	0.16492597
WGC107856D	6.49E-86	0.001834814	0.18377553	0.399953615	0.08417115	0.015461194	0.078695724	0.024340035	0.211767937
WGC107858D	0.123089941	0.032022799	0.048554155	0.159705325	0.089068014	0.034215316	0.200150309	0.108396087	0.204798054
WGC107860D	0.350404999	0.029049728	0.065999768	0.055477315	0.086418711	0.122571671	0.091399572	0.129029748	0.069648488
WGC107862D	0.03894426	0.011089408	0.135998567	0.278535235	0.05686426	0.017825537	0.155256999	0.037451308	0.268034425
WGC107864D	0.046631278	0.010161262	0.106564377	0.284704482	0.04530891	0.094506156	0.114555059	0.022956276	0.274612201
WGC107866D	0.026294067	0.001828906	0.154229991	0.418622693	0.037194185	1.13141032897645e-321	0.113855139	0.004766048	0.243208972
WGC107868D	0.116897193	0.00604475	0.336440849	0.142989762	1.45E-211	0.005683671	0.178813074	0.117660808	0.095469893
WGC107870D	0.228163793	0.023445517	0.039224337	0.087201774	0.087105898	0.06405269	0.253351752	0.092891388	0.124562852
WGC107872D	0.201621217	0.013729759	0.054313328	0.15383683	0.083260598	1.09E-261	0.299753552	0.05805517	0.135429545
WGC107874D	0.056120439	0.186432808	0.045771232	0.10429578	0.002874598	0.129483186	0.112381056	0.246602777	0.116038124
WGC108520D	2.40609969524687e-321	2.30234590962021e-321	0.198698988	0.471444144	0.107958234	2.93E-139	0.053329739	0.018990503	0.149578392
WGC108522D	0.410422662	0.005934061	0.042163626	0.120796367	7.82E-233	2.17E-180	0.30728197	2.06E-188	0.113401314
WGC108526D	0.405527852	0.009450289	0.025583148	0.092471961	0.011663059	0.003125506	0.341020478	0.00449491	0.106662796
WGC108528D	0.353613295	0.025820518	0.037237271	0.093188763	0.090661866	0.11237155	0.148893061	0.051798785	0.086414892
WGC108530D	0.390885318	0.047322378	0.019322013	1.11658835960122e-321	0.135016682	0.06273394	0.235262667	0.104107534	0.005349467
WGC108532D	0.151002633	0.016203022	0.05020071	0.180522409	0.096449472	0.03579275	0.228865551	0.089753396	0.151210057
WGC108536D	0.230247269	0.015602075	0.06740124	0.128317662	0.054014328	0.008993245	0.284224625	0.038059391	0.173140164
WGC108538D	0.109094602	0.04245122	0.049145216	0.061715447	0.098560983	0.2386224	0.142809065	0.131466476	0.126134591
WGC108542D	0.254875267	0.020191232	0.079098834	0.110886181	0.045897843	0.130040699	0.121944393	0.085477482	0.151588068
WGC108544D	0.137751884	0.016858279	0.090339347	0.114298333	0.091695909	0.112632571	0.127659236	0.096780194	0.211984246
WGC108546D	0.425975305	0.015111293	0.047211519	0.115184168	0.047426628	0.015780229	0.194719142	0.050160089	0.088431626

WGC108548D	0.009056625	0.012832097	0.171366601	0.470740634	0.054269805	0.003654373	0.078567458	0.044253085	0.155259322
WGC108550D	0.285650804	0.011986783	0.006471743	0.060202009	0.059843285	0.199449594	0.201669975	0.105296293	0.069429515
WGC108552D	0.012180752	0.015889993	0.124351301	0.348462588	0.098236105	0.005756448	0.113396367	0.024188884	0.257537561
WGC108554D	0.290582084	0.035262024	0.008304832	0.03022381	0.063197659	0.222235063	0.07907954	0.239244511	0.031870477
WGC108556D	0.126553097	0.015876529	0.054510849	0.109510105	0.035885199	0.077457131	0.220703373	0.200504686	0.15899903
WGC108558D	0.154935228	0.018901628	0.065133597	0.119259893	0.135009028	5.09E-126	0.230769704	0.050376188	0.225614734
WGC108560D	0.086257324	0.014045811	0.086858243	0.160108983	0.130717803	0.011648481	0.266509917	0.079756924	0.164096514
WGC108564D	0.264588026	0.02294391	0.084525826	0.133096003	0.033596766	0.014958959	0.127409591	0.153680478	0.16520044
WGC108566D	0.176748987	0.018819107	0.05052699	0.093781045	0.124263929	0.036833933	0.215998932	0.059681151	0.223345926
WGC108568D	0.282633792	0.04152713	0.008186504	0.097904757	0.199449798	0.046385122	0.064964006	0.171564874	0.087384017
WGC108570D	0.165687634	0.018713841	0.037117764	0.100650954	0.13589756	0.016538387	0.129932959	0.189521437	0.205939465
WGC108572D	0.119270459	0.016827857	0.204022746	0.109452757	0.127588647	0.050069483	0.1009223	0.068670777	0.203174974
WGC108574D	0.196166876	0.03882205	0.043335411	0.091421921	0.015448071	0.095938122	0.160933881	0.199986194	0.157947474
WGC108576D	0.171978011	0.014614799	0.06127437	0.192436299	0.06826105	0.00335248	0.376172711	0.010466825	0.101443457
WGC108578D	0.428628278	0.011964702	0.024417945	0.140535688	2.97921584442272e-321	1.92685601878086e-321	0.254679965	0.001151436	0.138621986
WGC108580D	0.252453938	0.010569444	0.015208085	0.040858676	0.101127459	0.308391872	0.108512772	0.101678469	0.061199285
WGC108582D	0.20850238	0.045660558	0.044688061	0.110502846	0.078899785	0.104930159	0.178250855	0.097742738	0.130822618
WGC108584D	0.039647895	0.074321397	0.217676253	0.193142668	0.132010222	0.000859069	0.195309683	0.028361205	0.118671608
WGC108586D	0.536539814	0.004750702	0.014204921	0.167563615	1.74899238627801e-321	1.49207825044056e-321	0.259520997	0.000924184	0.016495768
WGC108590D	0.30955816	0.019591028	0.02730998	0.105540363	0.020141438	0.104659845	0.231580884	0.137051684	0.044566617
WGC108592D	0.312202173	0.010298805	0.043936376	0.144215625	0.025154539	0.00064807	0.271085134	0.01197762	0.18048166
WGC108596D	5.13828271674896e-322	5.88602540905047e-311	0.137332085	0.333041339	0.215148494	5.82997462092671e-322	7.46039125220282e-322	1.31421461793772e-321	0.314478083
WGC108600D	0.246573612	0.015587391	0.055399898	0.120310655	0.055640905	0.080121643	0.323504372	0.035939126	0.066922398
WGC108602D	0.25973185	0.020291693	0.067919219	0.094418924	0.126667844	0.064892932	0.189870223	0.100618094	0.075589221
WGC108604D	0.478739067	0.016143042	0.011698226	0.13002781	0.044472886	0.072234313	0.084186052	0.113223458	0.049275146
WGC108606D	0.180656764	0.016454225	0.066052003	0.103435562	0.080023695	0.095701621	0.221703577	0.131995632	0.103976921

WGC108608D	0.391360906	0.019268826	0.012683098	2.76676761671 098e-322	0.147841405	0.070045122	0.225731324	0.124147707	0.008921612
WGC108614D	0.138664135	0.008679769	0.067118503	0.185485278	0.06374546	0.043638518	0.249001425	0.074199798	0.169467115
WGC108620D	0.125409995	0.023104154	0.05080777	0.099354673	0.089537832	0.066990707	0.213867794	0.032940901	0.297986175
WGC108624D	0.180121994	0.008021022	0.051586046	0.105507308	0.10177248	0.013823102	0.276857063	0.05556897	0.206742015
WGC108815D	0.295546861	0.017063121	0.015678651	0.088378322	0.070997071	0.175679294	0.168404672	0.076628234	0.091623774
WGC108817D	0.307634473	0.011905509	0.186011571	0.111781077	0.080602603	0.045363612	0.095292872	0.096707059	0.064701223
WGC108819D	0.62106513	0.00969885	0.007886578	0.032556607	0.149317885	0.009056373	0.089643487	0.046520715	0.034254375
WGC108825D	0.259743901	0.021561017	0.034334144	0.057556212	0.03387178	0.234584439	0.050156909	0.276439791	0.031751807
WGC108827D	0.218378232	0.016123995	0.025406895	0.110051621	0.074435794	0.009298479	0.243245792	0.161918249	0.141140943
WGC108831D	0.080820256	0.013595125	0.241168442	0.172092716	0.105950979	0.017426595	0.16093653	0.041757135	0.166252223
WGC108833D	0.150901365	0.024466677	0.053845646	0.100879911	0.119489956	0.055689883	0.195639949	0.107221017	0.191865595
WGC108835D	0.326388292	0.011771639	0.046616772	0.104104578	0.062389452	0.082056415	0.214719185	0.100664014	0.051289653
WGC108837D	0.169080097	0.024258334	0.043677055	0.158785515	0.033081375	7.26276499386 632e-322	0.27371727	0.151323074	0.14607728
WGC108841D	0.237541155	0.026091307	0.056144417	0.101055032	0.101696065	0.014968183	0.27431633	0.072094267	0.116093243
WGC108845D	0.08685685	0.014559108	0.079646094	0.138267408	0.100781235	0.003980397	0.299358351	0.14294822	0.133602339
WGC108847D	0.238228534	0.010545663	0.028409316	0.08705461	0.156086144	0.037773152	0.335849079	0.050691955	0.055361546
WGC108849D	0.182786099	0.012444785	0.055388376	0.101842645	0.033425967	0.102487801	0.199837204	0.196423571	0.115363553
WGC108851D	0.172613796	0.021525878	0.092581281	0.136558711	0.02932791	0.105504666	0.106495188	0.21375267	0.121639898
WGC108853D	0.008736727	0.003287954	0.169982922	0.35806869	0.11987015	1.60571334898 405e-321	0.099760838	0.007287421	0.233005298
WGC108855D	0.093145573	0.020148217	0.076044345	0.205593332	0.061223114	0.016219289	0.252588075	0.123751167	0.151286888
WGC108857D	0.184477831	0.014242211	0.037068065	0.105820178	0.074648703	0.073991122	0.297263902	0.100043149	0.112444839
WGC108859D	0.143503436	0.020663707	0.074230472	0.056315419	0.093469644	0.20147317	0.177958405	0.07471092	0.157674829
WGC108861D	0.487069155	0.003401549	0.013295062	0.063212817	0.051632756	0.003530935	0.200835882	4.39E-175	0.177021844
WGC108863D	0.089285554	0.015146342	0.08567795	0.170978648	0.061604205	0.050166337	0.253195917	0.064047182	0.209897865
WGC108865D	0.100525933	0.029031165	0.034092336	0.173493194	0.087369908	0.01982636	0.224232462	0.07464783	0.256780813
WGC108867D	0.062898287	0.022486032	0.228774944	0.108418868	0.066662192	0.096726485	0.128225216	0.081247705	0.204560271

WGC108869D	3.98E-114	0.003671669	0.150979497	0.318986871	0.078714496	1.18081689356 058e-321	0.16750907	9.436653835567 81e-322	0.280138396
WGC108871D	0.127522859	0.028688726	0.05744801	0.145407218	0.035726773	0.063675401	0.219132937	0.145939287	0.176458788
WGC108873D	0.152621743	0.018231759	0.031186729	0.101952094	1.92E-08	0.039249022	0.201969712	0.085829741	0.368959181
WGC108877D	0.094999624	0.025185465	0.063799771	0.157150446	0.150206383	0.053450648	0.240341208	0.062452809	0.152413647
WGC108879D	0.136034841	0.014091698	0.035450035	0.061604426	0.109996075	0.17406957	0.217638386	0.101853332	0.149261637
WGC108881D	0.225860128	0.020796855	0.040476038	0.10608578	0.052358982	4.10074486048 235e-322	0.249854091	0.090414782	0.214153343
WGC108883D	0.181733502	0.027605228	0.043382724	0.067264642	0.052267767	0.14072716	0.227198791	0.117577882	0.142242305
WGC108885D	0.178960807	0.004051723	0.422060065	0.036274907	0.03267665	0.054811399	0.108500905	0.054012782	0.108650762
WGC108889D	0.1634441	0.030713482	0.026756157	0.056487508	0.055878743	0.079283717	0.175245772	0.207104414	0.205086107
WGC108891D	0.299228482	0.012875362	0.026955199	0.033553558	0.051208508	0.138831002	0.17410538	0.152742896	0.110499613
WGC108893D	0.168474829	0.010098219	0.0360863	0.135626735	0.073665311	0.032034529	0.305549175	0.063440956	0.175023946
WGC108895D	0.28060073	0.02066738	0.042966383	0.059832081	0.105883949	0.076974614	0.175228263	0.143311945	0.094534654
WGC108897D	0.456953583	0.011141209	0.00699936	0.108771623	0.071202901	0.004036856	0.248942653	0.019630865	0.072320951
WGC108899D	0.136456814	0.012717065	0.106124226	0.129048194	0.101206761	0.026053824	0.275017983	0.084423996	0.128951137
WGC108901D	0.199551824	0.011566578	0.05719355	0.176204839	0.044114416	0.035630187	0.241201417	0.099062639	0.13547455
WGC108903D	0.336152938	0.023370825	0.007586967	0.023511142	0.121029633	0.193667884	0.097495863	0.123222281	0.073962467
WGC108905D	0.190606273	0.010675189	0.018193783	0.037317492	0.050112771	0.494828875	0.105844398	0.029212287	0.063208932
WGC108907D	0.182629548	0.029913284	0.042768879	0.027633916	0.106482057	0.133290502	0.180138582	0.084381638	0.212761594
WGC108909D	0.166254393	0.016006032	0.043622603	0.104751463	0.065703379	0.034541842	0.234207277	0.12423256	0.210680451
WGC108911	0.20260715	0.022683199	0.04496586	0.04866071	0.094137723	0.004944985	0.319795092	0.054309158	0.207896122
WGC108913	0.133400624	0.018728599	0.038704795	0.100622058	0.093145238	0.051180367	0.276505697	0.063291718	0.224420904
WGC108915	0.308448084	0.011690803	0.03452608	0.096855001	0.024096448	3.07E-30	0.338734335	0.006138305	0.179510945
WGC108917	0.128400577	0.02747419	0.048948117	0.095195456	0.043094579	0.014998944	0.189038857	0.17566913	0.277180149
WGC108919	0.131564096	0.015471326	0.03974924	0.114520592	0.084694371	0.044176332	0.366348924	0.029916127	0.173558991
WGC108921	0.15040595	0.016346857	0.21512157	0.047127211	0.078470004	0.025890599	0.250143124	0.098015162	0.118479521
WGC109742	0.14753809	0.033651989	0.032080181	0.087617792	0.021627802	0.21043234	0.215624364	0.0480589	0.203368542
WGC109744	0.165284951	0.049699905	0.03935048	0.073238303	0.032365608	0.032677144	0.240313459	0.075476523	0.291593626

WGC109746	0.295752562	0.039703485	6.26E-291	0.044578369	0.174952143	0.106089577	0.102702736	0.15885954	0.077361587
WGC109748	0.155621392	0.018204924	0.049433686	0.104365266	0.029711877	0.120520941	0.291780927	0.119887175	0.110473813
WGC109750	0.237853105	0.014455648	0.009099304	0.042399016	0.152555636	0.012205542	0.280851415	0.047881069	0.202699264
WGC109752	0.110857395	0.02240211	0.032577084	0.160843165	0.105439835	0.040370981	0.286776159	0.050799691	0.189933581
WGC109754	0.148936719	0.027809739	0.036002146	0.121066741	0.087919552	0.042098028	0.244305303	0.091258184	0.200603588
WGC109756	0.121472585	0.022574648	0.043320185	0.133633728	0.052043813	0.036142823	0.310716529	0.043721781	0.236373907
WGC109758	0.590951798	0.012122838	0.016062545	0.118083425	0.01103816	0.002917606	0.185245898	1.73E-44	0.06357773
WGC109760	0.229634042	0.014829599	0.014357947	0.093183343	0.072387209	0.021260644	0.280731943	0.025257337	0.248357937
WGC109762	0.192157383	0.017066284	0.053188576	0.063007248	0.092189238	0.072477864	0.253198434	0.04057884	0.216136133
WGC109764	8.99E-11	0.006663592	0.160233147	0.353368225	0.098292186	0.005281927	0.074232204	0.037167483	0.264761237
WGC109770	0.183431915	0.014134698	0.054377903	0.102164231	0.121462264	0.095353236	0.222683057	0.121649275	0.084743421
WGC109774	0.066714606	0.030672701	0.08343408	0.15969389	0.089264708	0.071711923	0.173247924	0.124951156	0.200309012
WGC109776	0.230979755	0.025101327	0.028512719	0.085888702	0.073245198	0.000243323	0.188644833	0.090402274	0.276981869
WGC109778	0.243211891	0.009850259	0.033428592	0.089026955	0.083555836	0.000589769	0.428366017	0.011520253	0.100450427
WGC109780	0.208116161	0.01417494	0.037497629	0.102445464	0.041325899	0.085791972	0.231108838	0.100352018	0.179187078
WGC109782	0.08463363	0.012615307	0.04197746	0.094331136	0.064871343	0.41907406	0.156661218	0.03306869	0.092767156
WGC109784	0.231889767	0.019762704	0.048755527	0.072602501	0.086461405	0.046588639	0.229273395	0.116493734	0.148172327
WGC109786	0.214487269	0.027441175	0.055613349	0.093006369	0.029029753	0.09374381	0.225963861	0.067551592	0.193162823
WGC109788	0.20098013	0.028506059	0.018429999	0.047759859	0.029781983	0.164875882	0.154790942	0.159704878	0.195170267
WGC109790	0.334967015	0.016121324	0.041409381	0.151016894	0.037162963	0.036454695	0.188555698	0.075607766	0.118704263
WGC109792	0.333503683	0.027213405	0.033051231	0.077429406	0.078777225	0.1207459	0.129492453	0.169643971	0.030142726
WGC109794	0.281220224	0.016812637	0.021903382	0.090048137	0.072571662	0.080734322	0.153450009	0.178980483	0.104279143
WGC109796	0.316936335	0.011417532	0.014222937	0.055740652	0.061616309	0.268523351	0.143994561	0.091932037	0.035616286
WGC109798	0.334828369	0.010674569	0.024192735	0.03687208	0.016595693	0.092606512	0.270131323	0.031821924	0.182276796
WGC109800	4.51E-201	0.00455537	0.17681988	0.330762907	0.075579701	1.06E-66	0.06908405	0.031035477	0.312162615
WGC109802	1.82310223315 42e-321	0.001791058	0.146059484	0.359672719	0.107234233	0.01577586	0.082676741	0.01018418	0.276605726
WGC109804	0.101483579	0.017271097	0.044032432	0.157323209	0.086034177	0.041883126	0.289036794	0.089140424	0.173795161
WGC109806	0.078686607	0.020113011	0.047905036	0.130126074	0.062599409	0.126052105	0.252964821	0.099410704	0.182142233

WGC109808	0.141193112	0.030696901	0.04305502	0.095330543	0.046625874	0.036795688	0.27571344	0.072352355	0.258237068
WGC109810	0.070582735	0.022930821	0.071094928	0.195219184	0.071969168	0.016147593	0.274173703	0.098666062	0.179215806
WGC109812	0.232644925	0.011997355	0.067482134	0.132710288	0.011142023	0.066830409	0.237794966	0.114492143	0.124905758
WGC109814	0.185975111	0.009103803	0.074364793	0.169516957	0.042810853	0.026594715	0.299905691	0.030515576	0.1612125
WGC109816	0.11762523	0.024127044	0.045361366	0.11036459	0.103690841	0.11104424	0.225827264	0.111431524	0.150527901
WGC109818	0.139185405	0.021339304	0.044493688	0.095254347	0.040265538	0.084396839	0.232869688	0.11332018	0.228875011
WGC109820	0.050952318	0.006242963	0.062511595	0.207844892	0.123570536	0.016237894	0.326683104	0.032662368	0.17329433
WGC109824	0.095593406	0.019945016	0.065898777	0.166125156	0.137154342	0.007639321	0.226044354	0.017266457	0.26433317
WGC109828	0.225777107	0.036936597	0.053350174	0.065680221	0.017790314	0.029725149	0.242940734	0.162722312	0.165077392
WGC109830	0.17491991	0.015627793	0.039029327	0.106567281	0.060332187	0.117616033	0.237246634	0.035647595	0.213013239
WGC109832	0.213904916	0.101290434	0.038893068	0.073031309	0.073148179	0.031080306	0.206010018	0.081844311	0.180797461
WGC109836	0.069354812	0.011479714	0.072550342	0.211745905	0.113442256	0.065272218	0.209544807	0.105864849	0.140745098
WGC109840	0.151678177	0.022793944	0.059983563	0.071607405	0.062951703	0.114223298	0.27373469	0.161029383	0.081997837
WGC109842	0.201691315	0.023942925	0.051428847	0.128715015	0.049177908	0.0134084	0.320735196	0.086349331	0.124551063
WGC109844	0.125200829	0.00911502	0.339765665	0.048582009	0.021803403	0.100880259	0.168148433	0.032225894	0.154278488
WGC109846	0.274987582	0.002943008	0.046492441	0.125167511	0.048073001	0.061389771	0.316183493	0.024865472	0.099897721
WGC109848	0.154725981	0.023037455	0.061081943	0.092065386	0.093088051	0.049882607	0.277463472	0.06599686	0.182658245
WGC109850	0.340175929	0.007388374	0.034981946	0.146731405	0.042989289	0.007363969	0.361966095	0.009445496	0.048957496
WGC109852	0.299747938	0.024897455	0.02077266	0.012933438	0.090550219	0.059269082	0.239627227	0.143231655	0.108970324
WGC109854	0.240172599	0.016495834	0.020097677	0.078777004	0.108712959	0.068189479	0.224893474	0.094777861	0.147883113
WGC109856	0.221549079	0.016769814	0.042286348	0.101379068	0.105263526	0.045746076	0.227927365	0.129303284	0.10977544
WGC109858	0.062585754	0.026373933	0.119853369	0.128563271	0.159900344	0.051198126	0.230718668	0.099337346	0.121469189
WGC110899	0.247872453	0.0133914	0.034035297	0.108566636	0.034720221	1.03457346239<br>157e-320	0.334519966	0.010198664	0.216695363
WGC110903	0.169380519	0.018333838	0.056150531	0.139034067	0.050249016	0.040873241	0.204282592	0.151679328	0.170016867
WGC110907	0.174861266	0.023570539	0.040294864	0.06214228	0.082726666	0.191341296	0.152770507	0.112533681	0.1597589
WGC110909	0.250981844	0.006904016	0.218982334	0.032858052	0.117373957	0.029802445	0.165145403	0.071456056	0.106495894
WGC110911	0.37856297	0.009036611	0.034568895	0.095529081	0.030889142	4.69E-40	0.327337939	0.012396331	0.111679032
WGC110915	0.274841321	0.026023748	0.048387389	0.067917308	0.078216858	0.053301139	0.238901133	0.103618372	0.108792734

WGC110917	0.017869994	0.018986387	8.76E-242	1.55E-52	0.083456196	0.720373739	0.139586042	0.004561634	0.015166007
WGC110919	0.264232195	0.022070225	0.026583804	0.061059847	0.015780069	0.147477474	0.175677127	0.177077162	0.110042097
WGC110921D	0.29252939	0.016539182	0.056801627	0.086014754	0.059576738	0.046698298	0.232757298	0.131039906	0.078042807
WGC110923D	0.223888166	0.025777592	0.021613595	0.062016669	0.070870706	0.212733203	0.175921369	0.122553623	0.084625077
WGC110925D	0.281250719	0.201613289	0.018813988	0.051216427	2.7371236779 6051e-321	0.164695042	0.073304777	0.189542079	0.01956368
WGC110927D	0.435880106	0.010172972	0.019498976	0.059647923	0.037862465	0.111037927	0.101986858	0.189402226	0.034510548
WGC110929D	0.610290524	0.016676567	0.023661126	0.042046854	0.115353426	0.009995836	0.079662782	0.102312885	1.08E-167
WGC110933D	0.404422711	0.016298317	0.013573177	0.039885376	0.118696089	0.142734318	0.148185009	0.083923849	0.032281154
WGC110935D	0.424229293	0.006708001	0.032540912	0.132040475	0.05699664	0.038288149	0.226768253	0.053620613	0.028807664
WGC110937D	0.261602872	0.020929585	0.037656896	0.090374975	0.028447281	0.068836765	0.192137599	0.181660964	0.118353062
WGC110939D	0.273662254	0.014549813	0.028487418	0.104117022	0.116983931	0.007469373	0.231396285	0.085108277	0.138225625
WGC110941D	0.275007134	0.012895308	0.031774862	0.059518571	0.06775051	0.186220991	0.150178565	0.160945475	0.055708584
WGC110943D	0.223486364	0.02190392	0.028657976	0.048410542	0.028745719	0.077124576	0.156620286	0.183448235	0.231602382
WGC110945D	0.225924041	0.008179261	0.035089485	0.050327815	0.067876888	0.243591961	0.180875937	0.097362823	0.090771789
WGC110947D	0.043110128	0.020518449	0.103129036	0.210409868	0.09682094	0.034111738	0.209193498	0.081658629	0.201047714
WGC110949D	0.095922032	0.014818995	0.061153345	0.186536715	0.050095919	0.018577769	0.13492382	0.039548955	0.398422449
WGC110951D	1.38832446481 39e-321	4.56022591111 471e-321	2.43080297753 893e-321	1.21046083231 105e-321	6.3635655184 3526e-321	0.996302931	0.003697069	5.97E-158	3.45845952088 873e-322
WGC110953D	0.523493756	2.28E-278	0.012607299	0.070291096	0.056551043	0.001191492	0.273501196	0.020646436	0.041717681
WGC110955D	0.184983345	0.007760784	0.413191851	7.52E-16	0.058662511	0.121518143	0.105383046	0.054519164	0.053981157
WGC110957D	0.450673985	0.027316453	0.013466127	0.067035879	0.073497083	0.029288083	0.255319008	0.083403381	3.58E-198
WGC110961D	0.175896873	0.025763712	0.042363515	0.108819732	0.109094097	0.079344144	0.262581533	0.108684617	0.087451779
WGC110963D	0.34133625	0.029491603	0.126748131	0.090173793	0.046136532	0.022168452	0.181021852	0.099216027	0.063707362
WGC110965D	0.301990923	0.014620778	0.014668723	0.03677754	0.03375752	0.07608273	0.240136693	0.110346998	0.171618095
WGC110967D	0.164522283	0.033742347	0.026027529	0.033478403	0.109100259	0.124773141	0.209032898	0.173231389	0.126091751
WGC110969D	0.670126409	3.19166407213 445e-321	0.003330219	0.096003474	0.041753409	2.18377015461 831e-321	0.186317978	0.00246851	8.59E-266
WGC110973D	0.054974739	0.00841347	0.277277179	0.172937681	0.069365952	9.37E-130	0.123549275	0.076799466	0.216682238

WGC110975D	0.243135725	0.023459212	0.025903526	0.067612591	0.087563778	0.051415897	0.257028731	0.06831483	0.17556571
WGC110977D	0.318687866	0.01896461	0.032764182	0.051852817	0.051756731	0.034835078	0.320932033	0.097137158	0.073069525
WGC110979D	0.25268818	0.017482282	0.053141391	0.065725612	0.048731392	0.092206556	0.223943595	0.147729769	0.098351222
WGC110981D	0.425708635	0.014566307	0.023062598	0.091010496	0.049314269	3.17E-124	0.286340976	0.019727071	0.090269647
WGC110983D	0.215989589	0.019813114	0.040036271	0.074404629	0.052753685	0.098661886	0.285357149	0.112574918	0.10040876
WGC110985D	0.258393319	0.013257545	0.052154176	0.107046828	0.033760418	0.096225136	0.228903962	0.105297942	0.104960674
WGC110987D	0.219572264	0.062682126	0.046312789	0.085655909	0.047978636	0.063088717	0.278600915	0.086238901	0.109869745
WGC110989D	0.062692721	0.063467251	0.071855934	0.105035731	0.082173542	0.140786638	0.198539631	0.131298181	0.144150371
WGC110991D	0.18724849	0.01546409	0.039289071	0.13862856	0.031902709	0.135864897	0.216700044	0.083486784	0.151415355
WGC110997D	0.214345555	0.019401266	0.044672595	0.122468773	0.090393461	0.00971799	0.27671823	0.03623649	0.186045639
WGC111001D	0.167545178	0.014674061	0.051798603	0.078504521	0.054370221	0.120822818	0.267027704	0.104663457	0.140593439
WGC111003D	0.257140505	0.037843137	0.017619927	0.076042087	0.057630196	0.020066602	0.163609621	0.093938929	0.276108998
WGC111005D	0.27885116	0.016481179	0.013429881	0.016705403	0.044161509	0.068155561	0.266312066	0.10375558	0.192147661
WGC111007D	2.14424490295 101e-321	0.006198246	0.239249096	0.353334273	0.062941423	0.004229671	0.049914392	0.026191081	0.257941818
WGC111009D	1.53E-159	0.001268035	0.169830438	0.374774293	0.039479074	1.81816157669 579e-321	0.13936886	0.014583908	0.260695392
WGC111011D	0.014326896	0.01401136	0.128342581	0.269010741	0.117548701	0.037525438	0.110598526	0.036943825	0.271691932
WGC111013D	0.286616537	0.016184288	0.056571864	0.114778872	0.010894427	0.066235812	0.271360774	0.096210328	0.081147099
WGC111015D	0.009266799	0.106929013	0.03679177	0.186992697	0.12026491	4.54540394173 947e-322	0.020406411	0.076198227	0.443150173
WGC111825D	0.304494853	0.009757811	0.033001482	0.143152168	0.021280512	0.002903259	0.331148179	0.00046161	0.153800127
WGC111827D	0.143805549	0.016215404	0.06240491	0.193133938	0.05223531	0.013550497	0.28493208	0.028479903	0.205242408
WGC111833D	0.188356947	0.024655451	0.046762404	0.10519198	0.084982115	0.047934045	0.301938374	0.074770426	0.125408258
WGC111835D	0.222442458	0.01259404	0.347939407	0.076524175	0.036332991	0.026573619	0.176227681	0.035606075	0.065759554
WGC111839D	0.258375786	0.023858546	0.035467291	0.025869926	0.065532296	0.03141652	0.212525211	0.120534084	0.226420341
WGC111841D	0.142249643	0.016311246	0.037819365	0.0820722	0.121572446	0.081032249	0.352925741	0.021596057	0.144421053
WGC111843D	0.294421682	0.029679409	0.040391211	0.097497258	0.004396855	0.00266916	0.268683141	0.139722015	0.122539269
WGC111845D	0.153333355	0.049140765	0.082476103	0.088728468	0.041794922	0.275389412	0.128811428	0.102116853	0.078208694

WGC111853D	0.264045227	0.021652019	0.055723693	0.121936122	0.074140745	0.00956761	0.326249618	0.005605832	0.121079133
WGC111857D	0.238830444	0.0163318	0.046676255	0.100296663	0.080611073	0.101310987	0.207591051	0.136168624	0.072183103
WGC111859D	0.219288037	0.008590419	0.028708213	0.088552679	0.040195853	0.136233187	0.263494362	0.16354042	0.05139683
WGC111861D	0.246961713	0.025025402	0.050159409	0.050024022	0.066030757	0.088601359	0.145604623	0.268797956	0.058794759
WGC111867D	0.292420985	0.022015745	0.035364664	0.036884089	0.032611438	0.029903251	0.233674397	0.145411282	0.171714148
WGC111869D	0.268034158	0.016009628	0.044563995	0.0670703	0.08004342	0.113313319	0.230346594	0.090753726	0.08986486
WGC111871D	0.26248817	0.014034776	0.033443178	0.043474144	0.111693648	0.138258436	0.198761494	0.144605729	0.053240425
WGC111873D	0.282008803	0.016208188	0.01893741	0.04139948	0.097628191	0.257115773	0.214594032	0.031617529	0.040490595
WGC111881D	0.437954223	0.002269543	0.01970305	0.160521337	3.10E-38	0.001830361	0.290687208	2.376455756496<br>4e-321	0.087034278
WGC111883D	0.075762115	0.007660415	0.087686259	0.258566206	0.025934487	0.001233605	0.227672635	0.042038767	0.273445511
WGC111885D	0.316036952	0.006519679	0.042544629	0.152066755	1.03E-22	2.24E-290	0.38133266	0.006431243	0.095068081
WGC111887D	0.33240829	0.01824167	0.032781496	0.078523978	0.052264352	0.020879549	0.186272801	0.078918392	0.199709472
WGC111889D	0.338729252	0.018209836	0.02018317	0.054545948	0.094350559	0.208180844	0.179308928	0.03391615	0.052575313
WGC111891D	0.21420434	0.016069698	0.020194958	0.101857825	0.081962803	0.104648959	0.292756189	0.070993323	0.097311904
WGC111895D	0.210511735	0.01728855	0.029040808	0.062739388	0.078470825	0.130694787	0.27378557	0.148587315	0.048881022
WGC111897D	0.34067992	0.022995448	0.027085388	0.03283642	0.036194227	0.135867553	0.181253676	0.180178125	0.042909243
WGC111899D	0.441910151	0.006374514	0.02534641	0.09814232	0.049505775	0.070814573	0.179141271	0.097471595	0.031293391
WGC111901D	0.266100722	0.011994042	0.029453516	0.050978437	0.027010515	0.194951441	0.136767883	0.142306524	0.140436919
WGC111909D	0.280926199	0.02543193	0.090639716	0.089614109	0.043340644	0.090195823	0.143897164	0.11576126	0.120193155
WGC111911D	0.365117743	0.008383276	0.036949765	0.162405985	5.58E-227	0.056319781	0.190740667	0.12554914	0.054533644
WGC111917D	0.146335109	0.039731617	0.509854065	0.065812882	7.43E-74	0.053775772	0.062921667	0.09658857	0.02498032
WGC111919D	0.178594205	0.015475319	0.047759565	0.08640352	0.070831634	0.084551011	0.277519265	0.188194994	0.050670487
WGC111921D	0.002380059	0.015414489	0.125952156	0.216354123	0.057498254	0.065005007	0.139961979	0.117107482	0.260326451
WGC111923D	0.022333554	0.029199493	0.190743118	0.282685076	0.094622327	0.015348317	0.113765712	0.072103918	0.179198486
WGC111925D	0.2134552	0.02703212	0.029644637	0.017488411	0.088944286	0.135647843	0.191580996	0.129467213	0.166739294
WGC111927D	0.40514974	0.015847725	0.023959913	0.056088303	0.060330638	0.017953918	0.262662438	0.043098873	0.114908451
WGC111929D	0.301245761	0.020887019	0.046853716	0.065592924	0.090732714	0.038278872	0.235410292	0.090629993	0.110368709
WGC111931D	0.384534791	0.013793714	0.027746036	0.065281367	0.083351309	3.30E-12	0.262411835	0.106670397	0.05621055

WGC111933D	0.352511505	0.019655067	0.031636824	0.115520594	0.062788702	0.032150658	0.266343403	0.065344373	0.054048874
WGC111935D	0.376449323	0.017791772	0.023053361	0.030418365	0.134274114	0.041776749	0.291211274	0.055317174	0.029707869
WGC111937D	0.370042916	0.02775965	0.031342429	0.086192896	0.047095055	0.023432102	0.22059165	0.133504233	0.060039069
WGC111939D	0.30784659	0.010194399	0.043260611	0.047286287	0.07574336	0.022907734	0.376321063	0.086189238	0.030250718
WGC111941D	0.304903778	0.015963373	0.038887406	0.021517021	0.041012692	0.164158832	0.230436315	0.121499433	0.061621151
