# Supplementary material for: A practical framework RNMF for exploring the association between mutational signatures and genes using gene cumulative contribution abundance
Source: Cancer Med. 2022 May 16;11(21):4053–69. doi: 10.1002/cam4.4717 (PMC9636515; doi:10.1002/cam4.4717)
Supplement: Supplementary file 12 — Table S3 [file CAM4-11-4053-s006.pdf]

Table S3. Mutation count of 1073 ESCC tumors.

SampleID	Mutation Count	HyperMutated	SampleID	MutationCount	HyperMutated
WGC110951D	1248	Yes	WGC108841D	101	No
TI1706140133LD01	1216	Yes	WGC108881D	101	No
WGC111003D	941	Yes	WGC110957D	101	No
FP1705220200LD01	772	Yes	WGC111939D	101	No
WGC108596D	734	Yes	AJHG2015-3N64-VS-3T64	100	No
FP1706060197LD01	711	Yes	FP1706060212LD01	100	No
WGC109858	645	Yes	FP1706060228LD01	100	No
FP1706060217LD01	628	Yes	Gastroenterology2016-ESCC_17	100	No
Gastroenterology2016-ESCC_143	594	Yes	Gastroenterology2016-ESCC_28	100	No
WGC109760	586	Yes	Gastroenterology2016-ESCC_77	100	No
WGC110917	571	Yes	Nature2014-ESCC-158T	100	No
WGC111909D	565	Yes	WGC109852	100	No
Gastroenterology2016-ESCC_11	541	Yes	WGC110929D	100	No
TI1706140139LD01	511	Yes	AJHG2015-1N51-VS-1T51	99	No
FP1705220189LD01	510	Yes	AJHG2015-3N39-VS-3T39	99	No
WGC110923D	500	Yes	nc2017-ESCC_171	99	No
WGC107828D	435	No	nc2017-ESCC_199	99	No
FP1705220210LD01	420	No	TI1706140187LD01	99	No
TI1706140161LD01	419	No	WGC109790	99	No
FP1706060213LD01	418	No	WGC111933D	99	No
Gastroenterology2016-ESCC_55	411	No	AJHG2015-3N43-VS-3T43	98	No
TI1706140171LD01	404	No	FP1705100101DN01	98	No
TI1706140153LD01	402	No	Gastroenterology2016-ESCC_154	98	No
Gastroenterology2016-ESCC_170	395	No	Nature2014-ESCC-210T	98	No
Gastroenterology2016-ESCC_158	387	No	nc2017-ESCC_234	98	No
WGC109818	372	No	nc2017-ESCC_64	98	No
TI1706140147LD01	369	No	FP1705100131DN01	97	No
Gastroenterology2016-ESCC_25	362	No	FP1706060200LD01	97	No
WGC110955D	354	No	Nature2014-ESCC-214T	97	No
FP1706060156LD01	353	No	nc2017-ESCC_179	97	No
WGC106606D	348	No	WGC111859D	97	No
WGC111845D	341	No	AJHG2015-1N52-VS-1T52	96	No
Gastroenterology2016-ESCC_13	337	No	AJHG2015-3N32-VS-3T32	96	No
WGC111935D	328	No	Nature2014-ESCC-246T	96	No
TI1706140179LD01	327	No	WGC108624D	96	No
WGC108580D	322	No	Gastroenterology2016-ESCC_132	95	No
FP1705100079DN01	320	No	Gastroenterology2016-ESCC_41	95	No
TI1706140137LD02	318	No	Gastroenterology2016-ESCC_52	95	No
WGC106614D	316	No	WGC106550D	95	No
WGC111917D	310	No	WGC107856D	95	No

WGC107836D	308	No	WGC110903	95	No
Gastroenterology2016-ESCC_109	301	No	WGC110909	95	No
Gastroenterology2016-ESCC_157	300	No	FP1706060162LD01	94	No
WGC107820D	299	No	Gastroenterology2016-ESCC_27	94	No
Gastroenterology2016-ESCC_10	296	No	Gastroenterology2016-ESCC_43	94	No
TI1706140167LD01	295	No	Gastroenterology2016-ESCC_87	94	No
TI1706140159LD01	286	No	nc2017-ESCC_E25	94	No
FP1706060153LD01	285	No	WGC106530D	94	No
WGC111925D	283	No	WGC107798D	94	No
WGC109810	282	No	Gastroenterology2016-ESCC_30	93	No
FP1705100073DN01	276	No	Gastroenterology2016-ESCC_71	93	No
Gastroenterology2016-ESCC_4	276	No	Nature2014-ESCC-216T	93	No
WGC108560D	276	No	nc2017-ESCC_125	93	No
WGC108570D	275	No	WGC111929D	93	No
WGC109746	275	No	AJHG2015-3N31-VS-3T31	92	No
FP1705100119DN01	272	No	FP1705220209LD01	92	No
WGC106538D	272	No	Gastroenterology2016-ESCC_137	92	No
WGC111007D	270	No	Gastroenterology2016-ESCC_167	92	No
FP1706060234LD01	269	No	Nature2014-ESCC-112T	92	No
WGC109788	268	No	FP1705220215LD01	91	No
FP1705220196LD01	267	No	Gastroenterology2016-ESCC_95	91	No
Gastroenterology2016-ESCC_68	265	No	Nature2014-ESCC-059T	91	No
FP1705100071DN01	264	No	TI1706140155LD01	91	No
TI1706140173LD01	260	No	WGC107852D	91	No
WGC108604D	260	No	WGC108564D	91	No
WGC106574D	256	No	WGC108827D	91	No
WGC109792	256	No	WGC111937D	91	No
TI1706140135LD01	254	No	AJHG2015-1N03-VS-1T03	90	No
WGC109816	254	No	FP1705100095DN01	90	No
TI1706140177LD01	253	No	nc2017-ESCC_23	90	No
WGC106562D	253	No	TI1706140165LD01	90	No
WGC108921	246	No	TI1706140193LD01	90	No
Gastroenterology2016-ESCC_76	244	No	WGC108572D	90	No
WGC109844	243	No	AJHG2015-3N05-VS-3T05	89	No
AJHG2015-1N31-VS-1T31	242	No	Gastroenterology2016-ESCC_111	89	No
FP1705100061DN01	242	No	Gastroenterology2016-ESCC_136	89	No
Gastroenterology2016-ESCC_164	240	No	Nature2014-ESCC-248T	89	No
WGC106534D	240	No	WGC106522DB	89	No
WGC110949D	236	No	WGC106556D	89	No
Nature2014-ESCC-184T	235	No	WGC109800	89	No
FP1706060165LD01	234	No	WGC111867D	89	No
WGC110919	233	No	FP1705220182LD01	88	No
WGC111835D	233	No	FP1706060178LD01	88	No

FP1705100099DN01	232	No	WGC108853D	88	No
WGC109770	232	No	WGC108877D	88	No
FP1705100065DN01	231	No	WGC109814	88	No
Gastroenterology2016-ESCC_129	231	No	Gastroenterology2016-ESCC_78	87	No
Gastroenterology2016-ESCC_160	230	No	Nature2014-ESCC-152T	87	No
WGC109796	229	No	nc2017-ESCC_26	87	No
AJHG2015-3N50-VS-3T50	226	No	WGC107808D	87	No
TI1706140175LD01	226	No	WGC108837D	87	No
nc2017-ESCC_E3	225	No	AJHG2015-1N38-VS-1T38	86	No
TI1706140115LD02	225	No	AJHG2015-3N63-VS-3T63	86	No
WGC107846D	225	No	FP1705100135DN01	86	No
Gastroenterology2016-ESCC_62	224	No	Gastroenterology2016-ESCC_128	86	No
Nature2014-ESCC-148T	224	No	Nature2014-ESCC-134T	86	No
TI1706140199LD01	224	No	Nature2014-ESCC-225T	86	No
FP1705100109DN01	222	No	nc2017-ESCC_19	86	No
WGC109854	221	No	WGC107834D	86	No
WGC111869D	220	No	AJHG2015-1N36-VS-1T36	85	No
WGC111861D	219	No	AJHG2015-3N36-VS-3T36	85	No
Gastroenterology2016-ESCC_44	218	No	Nature2014-ESCC-129T	85	No
TI1706140131LD01	218	No	nc2017-ESCC_16	85	No
WGC108889D	218	No	WGC107814D	85	No
WGC107824D	217	No	WGC108919	85	No
FP1706060159LD01	216	No	AJHG2015-1N25-VS-1T25	84	No
FP1705220199LD01	214	No	FP1705220202LD01	84	No
TI1706140113LD03	214	No	Nature2014-ESCC-205T	84	No
TI1706140163LD01	214	No	ng2014-Genomicandmolecular-ESCC-D4	84	No
WGC106602D	213	No	WGC108536D	84	No
WGC110945D	213	No	WGC108568D	84	No
Gastroenterology2016-ESCC_5	212	No	WGC111883D	84	No
WGC106580D	212	No	AJHG2015-1N27-VS-1T27	83	No
WGC110947D	211	No	Gastroenterology2016-ESCC_124	83	No
Gastroenterology2016-ESCC_142	210	No	Nature2014-ESCC-123T	83	No
WGC106524D	210	No	WGC107870D	83	No
FP1705100153DN01	209	No	AJHG2015-1N53-VS-1T53	82	No
FP1706060214LD01	209	No	AJHG2015-3N23-VS-3T23	82	No
Gastroenterology2016-ESCC_103	207	No	AJHG2015-3N28-VS-3T28	82	No
WGC109774	207	No	ng2014-Genomicandmolecular-ESCC-D21	82	No
Gastroenterology2016-ESCC_31	206	No	WGC106612D	82	No
WGC108903D	206	No	WGC107842D	82	No
WGC109744	205	No	AJHG2015-1N49-VS-1T49	81	No
FP1707040179LD01	204	No	FP1705100089DN01	81	No

Gastroenterology2016-ESCC_118	203	No	Nature2014-ESCC-116T	81	No
Gastroenterology2016-ESCC_153	203	No	nc2017-ESCC_182	81	No
WGC110925D	202	No	WGC108542D	81	No
FP1706060206LD01	201	No	FP1705100141DN01	80	No
FP1706060233LD01	200	No	FP1705220208LD01	80	No
Gastroenterology2016-ESCC_57	200	No	Gastroenterology2016-ESCC_110	80	No
Gastroenterology2016-ESCC_133	199	No	Gastroenterology2016-ESCC_56	80	No
FP1706060229LD01	198	No	Nature2014-ESCC-183T	80	No
WGC106566D	198	No	WGC107800D	80	No
WGC108554D	198	No	AJHG2015-1N21-VS-1T21	79	No
TI1706140119LD01	197	No	FP1705100097DN01	79	No
WGC108905D	197	No	Gastroenterology2016-ESCC_6	79	No
Gastroenterology2016-ESCC_152	196	No	Nature2014-ESCC-127T	79	No
Gastroenterology2016-ESCC_32	195	No	Nature2014-ESCC-235T	79	No
FP1706060223LD01	194	No	nc2017-ESCC_24	79	No
Gastroenterology2016-ESCC_161	194	No	WGC106526D	79	No
WGC108855D	194	No	WGC106528D	79	No
AJHG2015-1N45-VS-1T45	193	No	WGC110935D	79	No
WGC108909D	192	No	Gastroenterology2016-ESCC_114	78	No
WGC111873D	192	No	WGC108911	78	No
WGC111857D	191	No	WGC111927D	78	No
FP1706060202LD01	190	No	FP1705100139LD02	77	No
FP1705100137DN01	189	No	FP1705100159DN01	77	No
Gastroenterology2016-ESCC_54	188	No	WGC108851D	77	No
FP1705220184LD01	187	No	WGC111911D	77	No
Gastroenterology2016-ESCC_122	187	No	AJHG2015-1N37-VS-1T37	76	No
Nature2014-ESCC-015T	187	No	Nature2014-ESCC-002T	76	No
WGC107830D	187	No	Nature2014-ESCC-010T	76	No
WGC109812	185	No	nc2017-ESCC_239	76	No
WGC111001D	185	No	WGC108566D	76	No
FP1707180231LD01	184	No	AJHG2015-1N55-VS-1T55	75	No
FP1705220204LD01	183	No	AJHG2015-3N27-VS-3T27	75	No
FP1707180232LD01	183	No	AJHG2015-3N35-VS-3T35	75	No
Gastroenterology2016-ESCC_45	183	No	AJHG2015-3N53-VS-3T53	75	No
WGC107874D	183	No	FP1705100127LD02	75	No
FP1705220205LD01	182	No	Gastroenterology2016-ESCC_144	75	No
FP1705100081DN01	181	No	Gastroenterology2016-ESCC_64	75	No
Gastroenterology2016-ESCC_82	181	No	Nature2014-ESCC-003T	75	No
nc2017-ESCC_191	181	No	Nature2014-ESCC-083T	75	No
Gastroenterology2016-ESCC_134	180	No	ng2014-Genomicandmolecular-ESCC-D8	75	No
Gastroenterology2016-ESCC_159	180	No	WGC108835D	75	No
TI1706140149LD01	180	No	WGC110963D	75	No

WGC111889D	180	No	AJHG2015-1N23-VS-1T23	74	No
FP1705220192LD01	179	No	FP1705100113DN01	74	No
Gastroenterology2016-ESCC_169	179	No	FP1705100143DN01	74	No
WGC108857D	179	No	Nature2014-ESCC-055T	74	No
WGC109806	179	No	WGC111853D	74	No
AJHG2015-3N24-VS-3T24	178	No	AJHG2015-3N56-VS-3T56	73	No
TI1706140157LD01	178	No	Gastroenterology2016-ESCC_135	73	No
AJHG2015-3N44-VS-3T44	177	No	Gastroenterology2016-ESCC_66	73	No
FP1706060218LD01	177	No	Nature2014-ESCC-219T	73	No
Gastroenterology2016-ESCC_91	177	No	nc2017-ESCC_150	73	No
Nature2014-ESCC-190T	177	No	nc2017-ESCC_E45	73	No
WGC108849D	177	No	FP1705100115DN01	72	No
WGC109762	177	No	FP1705220212LD01	72	No
WGC111901D	177	No	Gastroenterology2016-ESCC_121	72	No
TI1706140143LD01	176	No	Nature2014-ESCC-175T	72	No
WGC108602D	176	No	nc2017-ESCC_169	72	No
WGC111895D	176	No	ng2014-Genomicandmolecular-ESCC-D14	72	No
WGC106552D	174	No	WGC106560D	72	No
WGC108917	173	No	AJHG2015-1N29-VS-1T29	71	No
Gastroenterology2016-ESCC_151	172	No	AJHG2015-1N50-VS-1T50	71	No
Nature2014-ESCC-153T	172	No	AJHG2015-1N59-VS-1T59	71	No
WGC107868D	172	No	AJHG2015-3N62-VS-3T62	71	No
FP1705220177LD01	171	No	FP1705220179LD01	71	No
WGC109830	171	No	Gastroenterology2016-ESCC_116	71	No
WGC109832	171	No	Nature2014-ESCC-070T	71	No
AJHG2015-1N30-VS-1T30	170	No	AJHG2015-1N32-VS-1T32	70	No
AJHG2015-1N40-VS-1T40	170	No	FP1706060160LD01	70	No
FP1706060209LD01	169	No	Nature2014-ESCC-180T	70	No
Gastroenterology2016-ESCC_73	169	No	Nature2014-ESCC-191T	70	No
WGC108833D	169	No	nc2017-ESCC_35	70	No
FP1705220181LD01	168	No	nc2017-ESCC_65	70	No
FP1706060230LD01	168	No	ng2014-Genomicandmolecular-ESCC-D2	70	No
FP1707040178LD01	167	No	WGC106596D	70	No
Gastroenterology2016-ESCC_156	167	No	WGC110997D	70	No
Gastroenterology2016-ESCC_162	167	No	AJHG2015-3N29-VS-3T29	69	No
Gastroenterology2016-ESCC_74	166	No	FP1705100067DN01	69	No
WGC106584D	166	No	Gastroenterology2016-ESCC_101	69	No
nc2017-ESCC_208	165	No	Nature2014-ESCC-147T	69	No
WGC108871D	165	No	nc2017-ESCC_173	69	No
FP1706060226LD01	164	No	AJHG2015-1N54-VS-1T54	68	No
FP1707040176LD01	163	No	AJHG2015-3N03-VS-3T03	68	No

WGC106618DB	163	No	AJHG2015-3N33-VS-3T33	68	No
WGC109840	163	No	AJHG2015-3N55-VS-3T55	68	No
Gastroenterology2016-ESCC_46	162	No	Gastroenterology2016-ESCC_100	68	No
Nature2014-ESCC-243T	162	No	Gastroenterology2016-ESCC_99	68	No
WGC108859D	162	No	Nature2014-ESCC-141T	68	No
WGC107794D	161	No	AJHG2015-3N34-VS-3T34	67	No
WGC110915	161	No	FP1705220186LD01	67	No
WGC111005D	161	No	Gastroenterology2016-ESCC_120	67	No
WGC111897D	161	No	Nature2014-ESCC-005T	67	No
Nature2014-ESCC-098T	160	No	WGC109824	67	No
WGC108899D	160	No	AJHG2015-3N42-VS-3T42	66	No
AJHG2015-1N46-VS-1T46	159	No	WGC106558D	66	No
FP1705100133DN01	159	No	WGC108893D	66	No
WGC108520D	159	No	AJHG2015-1N33-VS-1T33	65	No
WGC111009D	159	No	AJHG2015-3N30-VS-3T30	65	No
WGC108863D	158	No	FP1706060194LD01	65	No
WGC108879D	158	No	FP1706060203LD01	65	No
FP1705100163DN01	157	No	Nature2014-ESCC-185T	65	No
FP1706060191LD01	157	No	nc2017-ESCC_185	65	No
TI1706140151LD01	157	No	WGC106564D	65	No
WGC111923D	157	No	nc2017-ESCC_132	64	No
Gastroenterology2016-ESCC_96	156	No	AJHG2015-3N07-VS-3T07	63	No
TI1706140181LD01	156	No	AJHG2015-3N60-VS-3T60	63	No
WGC106570D	156	No	FP1706060170LD01	63	No
WGC108544D	156	No	Gastroenterology2016-ESCC_102	63	No
WGC110907	156	No	nc2017-ESCC_152	63	No
FP1706060172LD01	155	No	nc2017-ESCC_243	63	No
FP1706060177LD01	155	No	nc2017-ESCC_E79	63	No
Gastroenterology2016-ESCC_127	155	No	WGC107844D	63	No
Gastroenterology2016-ESCC_72	155	No	WGC107866D	63	No
WGC106572D	155	No	Nature2014-ESCC-006T	62	No
WGC110921D	155	No	nc2017-ESCC_10	62	No
WGC110989D	155	No	AJHG2015-1N64-VS-1T64	61	No
Gastroenterology2016-ESCC_51	154	No	FP1706060167LD01	61	No
WGC108574D	154	No	nc2017-ESCC_240	61	No
WGC108606D	154	No	nc2017-ESCC_27	61	No
WGC108825D	154	No	nc2017-ESCC_57	61	No
WGC108867D	154	No	ng2014-Genomicandmolecular-ESCC-D18	61	No
WGC110941D	154	No	ng2014-Genomicandmolecular-ESCC-D7	61	No
WGC111841D	154	No	AJHG2015-1N41-VS-1T41	60	No
WGC106578D	153	No	Nature2014-ESCC-057T	60	No

WGC107816D	153	No	AJHG2015-1N63-VS-1T63	59	No
WGC110987D	153	No	Nature2014-ESCC-128T	59	No
FP1706060188LD01	152	No	ng2014-Genomicandmolecular-ESCC-D13	59	No
Gastroenterology2016-ESCC_12	152	No	AJHG2015-1N04-VS-1T04	58	No
Gastroenterology2016-ESCC_35	151	No	AJHG2015-3N58-VS-3T58	58	No
WGC108530D	151	No	FP1705100129DN01	58	No
FP1705100149DN01	150	No	Nature2014-ESCC-240T	58	No
FP1705220214LD01	150	No	nc2017-ESCC_161	58	No
Gastroenterology2016-ESCC_126	150	No	ng2014-Genomicandmolecular-ESCC-D10	58	No
TI1706140185LD01	149	No	ng2014-Genomicandmolecular-ESCC-D15	58	No
AJHG2015-3N48-VS-3T48	148	No	AJHG2015-3N21-VS-3T21	57	No
FP1705100077DN01	148	No	AJHG2015-3N47-VS-3T47	57	No
FP1705220216LD01	148	No	Nature2014-ESCC-212T	57	No
FP1706060235LD01	148	No	AJHG2015-1N44-VS-1T44	56	No
FP1707040177LD01	148	No	ng2014-Genomicandmolecular-ESCC-F120	56	No
Gastroenterology2016-ESCC_107	148	No	AJHG2015-1N65-VS-1T65	55	No
TI1706140129LD02	148	No	Nature2014-ESCC-201T	55	No
WGC106588D	148	No	nc2017-ESCC_36	55	No
WGC107838D	148	No	ng2014-Genomicandmolecular-ESCC-D16	55	No
WGC110983D	148	No	AJHG2015-3N49-VS-3T49	54	No
Gastroenterology2016-ESCC_40	147	No	FP1705220211LD01	54	No
Nature2014-ESCC-250T	147	No	Nature2014-ESCC-100T	54	No
WGC107860D	147	No	ng2014-Genomicandmolecular-ESCC-D5	54	No
WGC108550D	147	No	WGC107822D	54	No
WGC108582D	147	No	AJHG2015-1N02-VS-1T02	53	No
WGC109782	147	No	AJHG2015-3N45-VS-3T45	53	No
FP1706060175LD01	146	No	Nature2014-ESCC-011T	53	No
Gastroenterology2016-ESCC_29	146	No	Nature2014-ESCC-014T	53	No
Gastroenterology2016-ESCC_38	146	No	nc2017-ESCC_245	53	No
WGC106598D	146	No	AJHG2015-1N66-VS-1T66	52	No
WGC107854D	146	No	AJHG2015-3N04-VS-3T04	52	No
AJHG2015-1N34-VS-1T34	145	No	Nature2014-ESCC-008T	52	No
WGC108873D	145	No	ng2014-Genomicandmolecular-ESCC-F93	52	No
WGC110975D	145	No	AJHG2015-1N24-VS-1T24	51	No
AJHG2015-3N57-VS-3T57	144	No	FP1706060176LD01	51	No
FP1705100093DN01	144	No	Nature2014-ESCC-012T	51	No

WGC108608D	144	No	nc2017-ESCC_224	51	No
WGC108817D	144	No	TI1706140121LD01	51	No
WGC108847D	144	No	WGC106542D	51	No
WGC109794	144	No	WGC110973D	51	No
WGC108600D	143	No	FP1705220190LD01	50	No
WGC108901D	143	No	nc2017-ESCC_134	50	No
WGC109776	143	No	nc2017-ESCC_178	50	No
AJHG2015-3N26-VS-3T26	142	No	nc2017-ESCC_62	50	No
FP1706060225LD01	142	No	Gastroenterology2016-ESCC_39	49	No
ng2014-Genomicandmolecular-ESCC-D20	142	No	nc2017-ESCC_140	49	No
WGC108895D	142	No	nc2017-ESCC_175	49	No
WGC109748	142	No	WGC107848D	49	No
FP1706060196LD01	141	No	nc2017-ESCC_12	48	No
FP1706060231LD01	141	No	nc2017-ESCC_54	48	No
Gastroenterology2016-ESCC_112	141	No	nc2017-ESCC_158	47	No
Nature2014-ESCC-139T	141	No	nc2017-ESCC_223	47	No
WGC108538D	141	No	WGC106586D	47	No
AJHG2015-3N09-VS-3T09	140	No	WGC109846	47	No
WGC106540D	140	No	Gastroenterology2016-ESCC_113	46	No
WGC107782D	140	No	Gastroenterology2016-ESCC_22	46	No
WGC108885D	140	No	AJHG2015-3N54-VS-3T54	45	No
Gastroenterology2016-ESCC_63	139	No	FP1706060221LD01	45	No
WGC109808	139	No	nc2017-ESCC_138	45	No
TI1706140125LD01	138	No	nc2017-ESCC_210	45	No
TI1706140191LD01	138	No	nc2017-ESCC_E47	45	No
WGC106582D	138	No	nc2017-ESCC_42	44	No
WGC109804	138	No	FP1706060193LD01	43	No
WGC110991D	138	No	Gastroenterology2016-ESCC_104	43	No
AJHG2015-1N57-VS-1T57	137	No	Nature2014-ESCC-249T	43	No
Gastroenterology2016-ESCC_117	137	No	nc2017-ESCC_61	43	No
WGC109754	137	No	ng2014-Genomicandmolecular-ESCC-F75	43	No
WGC110979D	137	No	WGC107872D	43	No
WGC111941D	137	No	FP1705220201LD01	42	No
AJHG2015-1N01-VS-1T01	136	No	WGC108869D	42	No
FP1705220198LD01	136	No	WGC110899	42	No
Gastroenterology2016-ESCC_149	136	No	WGC111015D	42	No
Gastroenterology2016-ESCC_58	136	No	AJHG2015-1N61-VS-1T61	41	No
WGC111899D	136	No	ng2014-Genomicandmolecular-ESCC-D17	41	No
AJHG2015-3N02-VS-3T02	135	No	AJHG2015-3N61-VS-3T61	40	No
FP1705100155DN01	135	No	Nature2014-ESCC-039T	40	No

FP1706060171LD01	135	No	WGC108819D	40	No
WGC107864D	135	No	WGC108831D	40	No
WGC109798	135	No	AJHG2015-1N42-VS-1T42	39	No
WGC110965D	135	No	nc2017-ESCC_201	39	No
FP1705100111DN01	134	No	ng2014-Genomicandmolecular-ESCC-F86	39	No
Gastroenterology2016-ESCC_131	134	No	AJHG2015-1N39-VS-1T39	38	No
Nature2014-ESCC-009T	134	No	AJHG2015-1N43-VS-1T43	38	No
TI1706140145LD01	134	No	Nature2014-ESCC-018T	38	No
WGC108556D	134	No	nc2017-ESCC_50	38	No
WGC111871D	134	No	ng2014-Genomicandmolecular-ESCC-D9	38	No
FP1706060224LD01	133	No	WGC108915	38	No
FP1706060227LD01	133	No	Nature2014-ESCC-209T	37	No
Gastroenterology2016-ESCC_3	133	No	ng2014-Genomicandmolecular-ESCC-D1	37	No
Gastroenterology2016-ESCC_53	133	No	ng2014-Genomicandmolecular-ESCC-F87	37	No
WGC108815D	133	No	WGC107788D	37	No
WGC109752	133	No	WGC108592D	37	No
WGC110933D	133	No	Nature2014-ESCC-154T	36	No
AJHG2015-1N56-VS-1T56	132	No	Nature2014-ESCC-231T	36	No
Gastroenterology2016-ESCC_14	132	No	nc2017-ESCC_145	36	No
TI1706140183LD01	132	No	WGC109850	36	No
WGC106594D	132	No	AJHG2015-3N06-VS-3T06	35	No
WGC108532D	132	No	WGC108546D	35	No
WGC109848	132	No	WGC108897D	35	No
AJHG2015-1N28-VS-1T28	131	No	WGC109750	35	No
AJHG2015-1N47-VS-1T47	131	No	AJHG2015-1N35-VS-1T35	34	No
FP1705220191LD01	131	No	Nature2014-ESCC-204T	34	No
FP1705220206LD01	131	No	nc2017-ESCC_131	34	No
FP1706060204LD01	131	No	nc2017-ESCC_198	34	No
Gastroenterology2016-ESCC_140	131	No	WGC110981D	34	No
Gastroenterology2016-ESCC_168	131	No	nc2017-ESCC_162	33	No
Gastroenterology2016-ESCC_92	131	No	ng2014-Genomicandmolecular-ESCC-D3	33	No
nc2017-ESCC_130	131	No	ng2014-Genomicandmolecular-ESCC-F71	33	No
WGC106616D	131	No	Nature2014-ESCC-017T	32	No
WGC108548D	131	No	ng2014-Genomicandmolecular-ESCC-F1	32	No
WGC108620D	131	No	AJHG2015-1N58-VS-1T58	31	No
WGC108913	131	No	nc2017-ESCC_170	31	No

WGC109742	131	No	ng2014-Genomicandmolecular-ESCC-D11	31	No
WGC109856	131	No	ng2014-Genomicandmolecular-ESCC-F58	31	No
FP1705220185LD01	130	No	AJHG2015-3N37-VS-3T37	30	No
Gastroenterology2016-ESCC_108	130	No	nc2017-ESCC_220	30	No
Gastroenterology2016-ESCC_33	130	No	TI1706140197LD01	30	No
nc2017-ESCC_55	130	No	WGC106576DB	30	No
WGC106544D	130	No	Nature2014-ESCC-004T	29	No
WGC106592D	130	No	nc2017-ESCC_E74	29	No
FP1706060161LD01	129	No	ng2014-Genomicandmolecular-ESCC-F104	29	No
FP1706060184LD01	129	No	ng2014-Genomicandmolecular-ESCC-F81	29	No
FP1706060185LD01	129	No	FP1706060179LD01	28	No
Gastroenterology2016-ESCC_8	129	No	Nature2014-ESCC-133T	28	No
Nature2014-ESCC-162T	129	No	ng2014-Genomicandmolecular-ESCC-F103	28	No
nc2017-ESCC_142	129	No	ng2014-Genomicandmolecular-ESCC-F40	28	No
WGC107806D	129	No	WGC110953D	28	No
WGC108907D	129	No	AJHG2015-1N48-VS-1T48	27	No
WGC111887D	129	No	FP1705100059DN01	27	No
AJHG2015-3N38-VS-3T38	128	No	WGC108576D	27	No
FP1705100103DN01	128	No	ng2014-Genomicandmolecular-ESCC-F85	26	No
WGC109802	128	No	Nature2014-ESCC-107T	25	No
TI1706140123LD01	127	No	ng2014-Genomicandmolecular-ESCC-F32	25	No
FP1705100145DN01	126	No	ng2014-Genomicandmolecular-ESCC-F76	25	No
Nature2014-ESCC-159T	126	No	WGC108578D	25	No
nc2017-ESCC_144	126	No	FP1705100125DN01	24	No
WGC107774D	126	No	FP1706060199LD01	24	No
WGC107790D	126	No	nc2017-ESCC_E30	24	No
WGC109780	126	No	WGC108861D	24	No
WGC110961D	126	No	WGC111825D	24	No
AJHG2015-3N40-VS-3T40	125	No	ng2014-Genomicandmolecular-ESCC-F91	23	No
AJHG2015-3N52-VS-3T52	125	No	ng2014-Genomicandmolecular-ESCC-F113	22	No
FP1705220197LD01	125	No	ng2014-Genomicandmolecular-ESCC-F66	22	No

Nature2014-ESCC-178T	125	No	WGC111881D	22	No
TI1706140111LD02	125	No	ng2014-Genomicandmolecular-ESCC-F25	21	No
TI1706140141LD01	125	No	ng2014-Genomicandmolecular-ESCC-F41	21	No
WGC110937D	125	No	ng2014-Genomicandmolecular-ESCC-F57	21	No
FP1705220195LD01	124	No	ng2014-Genomicandmolecular-ESCC-F80	21	No
FP1706060198LD01	124	No	TI1706140195LD01	21	No
Gastroenterology2016-ESCC_47	124	No	ng2014-Genomicandmolecular-ESCC-F10	20	No
Nature2014-ESCC-060T	124	No	ng2014-Genomicandmolecular-ESCC-F51	20	No
Nature2014-ESCC-211T	124	No	ng2014-Genomicandmolecular-ESCC-F83	20	No
WGC109786	124	No	WGC109758	20	No
WGC111931D	124	No	FP1706060155LD01	19	No
AJHG2015-1N05-VS-1T05	123	No	Nature2014-ESCC-001T	19	No
AJHG2015-3N25-VS-3T25	123	No	nc2017-ESCC_206	19	No
FP1705220213LD01	123	No	nc2017-ESCC_222	19	No
FP1706060222LD01	123	No	nc2017-ESCC_58	19	No
Gastroenterology2016-ESCC_18	123	No	ng2014-Genomicandmolecular-ESCC-F55	19	No
Gastroenterology2016-ESCC_9	123	No	ng2014-Genomicandmolecular-ESCC-F60	19	No
WGC108584D	123	No	WGC109778	19	No
WGC109828	123	No	WGC110911	19	No
AJHG2015-3N51-VS-3T51	122	No	ng2014-Genomicandmolecular-ESCC-F27	18	No
Gastroenterology2016-ESCC_146	122	No	ng2014-Genomicandmolecular-ESCC-F45	18	No
Nature2014-ESCC-237T	122	No	ng2014-Genomicandmolecular-ESCC-F59	18	No
WGC108590D	122	No	ng2014-Genomicandmolecular-ESCC-F74	18	No
Gastroenterology2016-ESCC_123	121	No	nc2017-ESCC_E34	17	No
WGC107826D	121	No	ng2014-Genomicandmolecular-ESCC-F111	17	No
WGC111839D	121	No	ng2014-Genomicandmolecular-ESCC-F14	17	No
AJHG2015-3N59-VS-3T59	120	No	ng2014-Genomicandmolecular-ESCC-F2	17	No

FP1706060169LD01	120	No	ng2014-Genomicandmolecular-ESCC-F34	17	No
Gastroenterology2016-ESCC_80	120	No	ng2014-Genomicandmolecular-ESCC-F36	17	No
Gastroenterology2016-ESCC_93	120	No	ng2014-Genomicandmolecular-ESCC-F38	17	No
WGC109756	120	No	ng2014-Genomicandmolecular-ESCC-F42	17	No
AJHG2015-3N41-VS-3T41	119	No	ng2014-Genomicandmolecular-ESCC-F50	17	No
Gastroenterology2016-ESCC_1	119	No	ng2014-Genomicandmolecular-ESCC-F52	17	No
Gastroenterology2016-ESCC_16	119	No	ng2014-Genomicandmolecular-ESCC-F84	17	No
Gastroenterology2016-ESCC_34	119	No	WGC107850D	17	No
Gastroenterology2016-ESCC_42	119	No	WGC108526D	17	No
Gastroenterology2016-ESCC_79	119	No	WGC111885D	17	No
Nature2014-ESCC-110T	119	No	nc2017-ESCC_225	16	No
nc2017-ESCC_168	119	No	ng2014-Genomicandmolecular-ESCC-F18	16	No
WGC106554D	119	No	ng2014-Genomicandmolecular-ESCC-F35	16	No
WGC110977D	119	No	ng2014-Genomicandmolecular-ESCC-F97	16	No
FP1705100117DN01	118	No	FP1705100123DN01	15	No
FP1706060195LD01	118	No	FP1706060163LD01	15	No
Gastroenterology2016-ESCC_139	118	No	FP1706060207LD01	15	No
WGC111011D	118	No	ng2014-Genomicandmolecular-ESCC-F109	15	No
WGC111843D	118	No	ng2014-Genomicandmolecular-ESCC-F114	15	No
AJHG2015-3N46-VS-3T46	117	No	ng2014-Genomicandmolecular-ESCC-F20	15	No
Gastroenterology2016-ESCC_165	117	No	ng2014-Genomicandmolecular-ESCC-F72	15	No
Gastroenterology2016-ESCC_61	117	No	nc2017-ESCC_149	14	No
Gastroenterology2016-ESCC_94	117	No	nc2017-ESCC_E11	14	No
Nature2014-ESCC-207T	117	No	ng2014-Genomicandmolecular-ESCC-F118	14	No
WGC107862D	117	No	ng2014-Genomicandmolecular-ESCC-F19	14	No
Gastroenterology2016-ESCC_130	116	No	ng2014-Genomicandmolecular-ESCC-F73	14	No

FP1705100161LD02	115	No	nc2017-ESCC_E78	13	No
Gastroenterology2016-ESCC_65	115	No	ng2014-Genomicandmolecular-ESCC-F100	13	No
Nature2014-ESCC-038T	115	No	ng2014-Genomicandmolecular-ESCC-F101	13	No
nc2017-ESCC_196	115	No	ng2014-Genomicandmolecular-ESCC-F11	13	No
WGC106568D	115	No	ng2014-Genomicandmolecular-ESCC-F12	13	No
WGC107840D	115	No	ng2014-Genomicandmolecular-ESCC-F21	13	No
WGC108558D	115	No	ng2014-Genomicandmolecular-ESCC-F46	13	No
WGC109784	115	No	ng2014-Genomicandmolecular-ESCC-F78	13	No
FP1705220187LD01	114	No	ng2014-Genomicandmolecular-ESCC-F90	13	No
FP1706060232LD01	114	No	ng2014-Genomicandmolecular-ESCC-F106	12	No
Gastroenterology2016-ESCC_21	114	No	ng2014-Genomicandmolecular-ESCC-F54	12	No
Gastroenterology2016-ESCC_37	114	No	ng2014-Genomicandmolecular-ESCC-F67	12	No
nc2017-ESCC_213	114	No	ng2014-Genomicandmolecular-ESCC-F69	12	No
nc2017-ESCC_215	114	No	ng2014-Genomicandmolecular-ESCC-F94	12	No
WGC108528D	114	No	WGC110969D	12	No
WGC111013D	114	No	nc2017-ESCC_E75	11	No
AJHG2015-1N60-VS-1T60	113	No	ng2014-Genomicandmolecular-ESCC-F102	11	No
Gastroenterology2016-ESCC_50	113	No	ng2014-Genomicandmolecular-ESCC-F13	11	No
WGC106600D	113	No	ng2014-Genomicandmolecular-ESCC-F39	11	No
WGC108614D	113	No	ng2014-Genomicandmolecular-ESCC-F48	11	No
AJHG2015-1N26-VS-1T26	112	No	ng2014-Genomicandmolecular-ESCC-F5	11	No
FP1705100091DN01	112	No	ng2014-Genomicandmolecular-ESCC-F63	11	No
FP1705100121DN01	112	No	ng2014-Genomicandmolecular-ESCC-F68	11	No

FP1705220188LD01	112	No	ng2014-Genomicandmolecular-ESCC-F79	11	No
FP1706060208LD01	112	No	ng2014-Genomicandmolecular-ESCC-F117	10	No
FP1706060216LD01	112	No	ng2014-Genomicandmolecular-ESCC-F15	10	No
WGC107802D	112	No	ng2014-Genomicandmolecular-ESCC-F17	10	No
FP1706060201LD01	111	No	ng2014-Genomicandmolecular-ESCC-F47	10	No
Gastroenterology2016-ESCC_150	111	No	ng2014-Genomicandmolecular-ESCC-F77	10	No
Gastroenterology2016-ESCC_163	111	No	ng2014-Genomicandmolecular-ESCC-F96	10	No
nc2017-ESCC_172	111	No	ng2014-Genomicandmolecular-ESCC-F98	10	No
nc2017-ESCC_39	111	No	ng2014-Genomicandmolecular-ESCC-F108	9	No
TI1706140189LD01	111	No	ng2014-Genomicandmolecular-ESCC-F3	9	No
WGC107812D	111	No	ng2014-Genomicandmolecular-ESCC-F30	9	No
WGC107858D	111	No	ng2014-Genomicandmolecular-ESCC-F43	9	No
WGC108552D	111	No	ng2014-Genomicandmolecular-ESCC-F53	9	No
WGC110927D	111	No	ng2014-Genomicandmolecular-ESCC-F56	9	No
WGC111919D	111	No	Nature2014-ESCC-013T	8	No
FP1706060210LD01	110	No	nc2017-ESCC_E26	8	No
Nature2014-ESCC-078T	110	No	ng2014-Genomicandmolecular-ESCC-F16	8	No
WGC106536D	110	No	ng2014-Genomicandmolecular-ESCC-F26	8	No
WGC106608D	110	No	ng2014-Genomicandmolecular-ESCC-F37	8	No
WGC111891D	110	No	ng2014-Genomicandmolecular-ESCC-F4	8	No
AJHG2015-1N62-VS-1T62	109	No	ng2014-Genomicandmolecular-ESCC-F44	8	No
FP1706060192LD01	109	No	ng2014-Genomicandmolecular-ESCC-F6	8	No
FP1706060205LD01	109	No	ng2014-Genomicandmolecular-ES	8	No

			CC-F62		
FP1706060215LD01	109	No	ng2014-Genomicandmolecular-ESCC-F92	8	No
Gastroenterology2016-ESCC_36	109	No	ng2014-Genomicandmolecular-ESCC-F95	8	No
Gastroenterology2016-ESCC_60	109	No	Nature2014-ESCC-144T	7	No
WGC108891D	109	No	nc2017-ESCC_E50	7	No
WGC109764	109	No	ng2014-Genomicandmolecular-ESCC-D12	7	No
WGC110985D	109	No	ng2014-Genomicandmolecular-ESCC-F110	7	No
WGC111833D	109	No	ng2014-Genomicandmolecular-ESCC-F23	7	No
Gastroenterology2016-ESCC_75	108	No	ng2014-Genomicandmolecular-ESCC-F24	7	No
Gastroenterology2016-ESCC_84	108	No	ng2014-Genomicandmolecular-ESCC-F31	7	No
nc2017-ESCC_156	108	No	ng2014-Genomicandmolecular-ESCC-F64	7	No
TI1706140127LD01	108	No	ng2014-Genomicandmolecular-ESCC-F7	7	No
WGC109836	108	No	ng2014-Genomicandmolecular-ESCC-F70	7	No
nc2017-ESCC_143	107	No	ng2014-Genomicandmolecular-ESCC-F9	7	No
WGC108865D	107	No	ng2014-Genomicandmolecular-ESCC-F99	7	No
Gastroenterology2016-ESCC_115	106	No	Nature2014-ESCC-229T	6	No
Gastroenterology2016-ESCC_125	106	No	nc2017-ESCC_21	6	No
Gastroenterology2016-ESCC_141	106	No	ng2014-Genomicandmolecular-ESCC-F107	6	No
Gastroenterology2016-ESCC_15	106	No	ng2014-Genomicandmolecular-ESCC-F28	6	No
Gastroenterology2016-ESCC_81	106	No	WGC108522D	6	No
AJHG2015-3N01-VS-3T01	105	No	WGC108586D	6	No
AJHG2015-3N08-VS-3T08	105	No	Nature2014-ESCC-092T	5	No
Gastroenterology2016-ESCC_86	105	No	ng2014-Genomicandmolecular-ESCC-D19	5	No
WGC107786D	105	No	ng2014-Genomicandmolecular-ESCC-F119	5	No
WGC107832D	105	No	ng2014-Genomicandmolecular-ESCC-F22	5	No
WGC110967D	105	No	ng2014-Genomicandmolecular-ES	5	No

			CC-F8		
AJHG2015-1N22-VS-1T22	104	No	ng2014-Genomicandmolecular-ESCC-F82	5	No
Nature2014-ESCC-233T	104	No	Nature2014-ESCC-164T	4	No
nc2017-ESCC_3	104	No	Nature2014-ESCC-171T	4	No
WGC106610D	104	No	Nature2014-ESCC-193T	4	No
WGC109820	104	No	nc2017-ESCC_60	4	No
WGC110943D	104	No	ng2014-Genomicandmolecular-ESCC-F105	4	No
WGC111921D	104	No	ng2014-Genomicandmolecular-ESCC-F115	4	No
AJHG2015-3N22-VS-3T22	103	No	ng2014-Genomicandmolecular-ESCC-F116	4	No
Gastroenterology2016-ESCC_83	103	No	ng2014-Genomicandmolecular-ESCC-F29	4	No
nc2017-ESCC_48	103	No	ng2014-Genomicandmolecular-ESCC-F49	3	No
WGC108845D	103	No	ng2014-Genomicandmolecular-ESCC-F89	3	No
WGC111827D	103	No	Nature2014-ESCC-016T	2	No
FP1706060180LD01	102	No	Nature2014-ESCC-155T	2	No
Gastroenterology2016-ESCC_24	102	No	Nature2014-ESCC-230T	2	No
nc2017-ESCC_249	102	No	nc2017-ESCC_246	2	No
TI1706140117LD01	102	No	Nature2014-ESCC-242T	1	No
WGC108883D	102	No	nc2017-ESCC_235	1	No
WGC109842	102	No	nc2017-ESCC_E33	1	No
WGC110939D	102	No	nc2017-ESCC_E71	1	No
FP1705220203LD01	101	No	ng2014-Genomicandmolecular-ESCC-F33	1	No
Gastroenterology2016-ESCC_26	101	No	ng2014-Genomicandmolecular-ESCC-F61	1	No
Nature2014-ESCC-069T	101	No			
