# Supplementary material for: A practical framework RNMF for exploring the association between mutational signatures and genes using gene cumulative contribution abundance
Source: Cancer Med. 2022 May 16;11(21):4053–69. doi: 10.1002/cam4.4717 (PMC9636515; doi:10.1002/cam4.4717)
Supplement: Supplementary file 13 — Table S4 [file CAM4-11-4053-s009.pdf]

Table S4. Abundance fractions matrix of single-base substitutions (SBS) in exon region of 1072 ESCC samples.

SampleNames	COSMIC SBS3*	COSMIC SBS16*	COSMIC SBS18*	New	COSMIC SBS33*	COSMIC SBS13*	COSMIC SBS2*	COSMIC SBS5*	COSMIC SBS1*	COSMIC SBS17b*	COSMIC SBS22*	COSMIC SBS15*
FP1705100059DN01	0.269278535	0	0.278484977	0.237042814	0	0	0	0.046392358	0	0.059862225	0	0.108939091
FP1705100061DN01	0	0	2.94E-165	2.01E-83	0.025521188	0.422184629	0.117994067	0.014841536	0.326915129	1.19E-33	0	0.09254345
FP1705100065DN01	0	0.192274997	1.23E-118	0	0.018320787	0.130643641	0.109021268	0.153594909	2.89E-01	0.00150484	0.054346135	0.051710551
FP1705100067DN01	0.12327879	0.086870737	0.105845305	0	0	0.001709162	0	0.245847904	0.323854832	0	0.025576738	0.087016533
FP1705100071DN01	0.020528556	0	0.047354524	0.016374403	3.99E-03	0.276975705	0.183830471	0.255256459	0.177081706	0.015196211	4.23E-14	0.003407111
FP1705100073DN01	0.276972889	0.058391132	2.03E-02	0	0.060274909	0.105973009	0.025973803	0.230001275	0.053858147	0.00962258	0.052705085	0.10594798
FP1705100077DN01	0	0	0.090590544	0.062720985	2.93E-17	2.50E-02	0.135610602	0.320640757	0.307808323	0.006337563	0.014312057	0.037026056
FP1705100079DN01	0.172823251	0	0.063710818	0	1.36E-02	1.58E-01	0.165817282	0.272165736	0.148652794	1.62E-90	2.21E-07	0.005642126
FP1705100081DN01	0.003591912	0.033662552	0.019566896	0.060481202	0.029889488	0.113580439	0.077271517	0.256359193	0.337129295	0.027031602	0	0.041435903
FP1705100089DN01	0.088449302	0.123290037	1.32E-34	0	0.025551449	0.030423203	0.093762858	0.351512729	0.266709599	0.013291085	3.14E-133	0.007009739
FP1705100091DN01	1.56E-26	0.048429543	4.54E-02	0	0.084270531	0.062332702	0.082746909	7.19E-49	0.489544354	0	0	0.187255111
FP1705100093DN01	0.067085109	0.086860122	0.180126544	3.05E-68	2.32E-48	0.080377809	0.030022736	8.26E-54	0.48284198	6.84E-74	0.010166729	0.062518971
FP1705100095DN01	0	0	0.16470396	0.030580155	0	0.070111486	0.133540748	0.052710322	0.429556174	0	0	0.118797155
FP1705100097DN01	0	7.82E-03	0.132541736	0.124081977	0	0	0.211918714	0.129982285	0.230208359	0.079323885	0	0.084127434
FP1705100099DN01	0.284331126	0	0.105689989	0	0.006789532	0.171299209	0.051983592	0.209439475	0.159542196	0	0.01092488	0
FP1705100101DN01	0	0	0.093463208	0.074364484	0.087632351	1.368561838 98025e-321	0	0.157984862	0.382689703	0	0.031758339	0.172107052
FP1705100103DN01	0	0	0.020381137	1.52E-73	0.012279258	0.035330038	0.08344392	0.160657193	0.472110484	0	0.005433417	0.210364551
FP1705100109DN01	0.059187203	0.030305539	3.62E-07	0.055772845	0.03046769	0.36516541	0.135092046	5.27E-32	0.310822576	0	0.004631373	0.008554957
FP1705100111DN01	0.030148877	0	1.32E-01	0	0	0.007319562	0.068197761	0.365821704	0.337848198	0.00709595	0.009264532	0.041956645
FP1705100113DN01	0	0	0.004807107	0	0.016492362	0.008239904	0.12735894	0.347356551	0.495745135	4.93E-49	0	6.14E-73
FP1705100115DN01	3.89E-15	0	3.45E-42	0	0.014863057	0.001316875	0	0.535907442	3.72E-01	0	0.013259349	0.062554183
FP1705100117DN01	3.21E-111	0.028669308	2.33E-94	0.057296046	0.029629253	0.108487302	0.166331293	0.023493746	0.449935555	1.56E-38	0	0.136157498
FP1705100119DN01	0.01063149	0.095060668	0.01310934	1.63E-22	0	0.385329404	0.163532832	0	0.320802191	0	0	0.011534076
FP1705100121DN01	0.031227628	0.030296625	0.152265478	0.046355879	0.002054945	0.015893849	0.077099821	0.193112022	0.451693754	0	0	1.34E-110
FP1705100123DN01	8.68E-72	0	0	8.10E-39	0.166039828	0	0	0.69760914	0.073061397	0.063289636	0	0

FP1705100125DN01	0	3.31E-20	0.00E+00	0.148450338	0	0	0.001743357	0.440768747	0.399901353	1.27E-09	0	0.009136203
FP1705100127LD02	0.013862016	0	0.061941295	0.022972465	0.001323646	7.46039125220282e-322	0.060090458	0.429119048	0.343835592	0	0.033721592	0.033133888
FP1705100129DN01	0.291957581	0	0.01537043	0	0.046314578	0.007212634	0	0.058163415	0.478909484	0	2.07E-164	0.102071878
FP1705100131DN01	0.005165002	0	0.026197771	0	0.13356384	0.06469679	0.06700178	0.090244956	0.613129861	0	1.27E-201	0
FP1705100133DN01	3.83E-10	0	0.052881597	0	0.015743904	0.039295108	0	7.50E-12	0.190366307	0.010796121	0.69091696	2.28E-09
FP1705100135DN01	1.92E-58	0.042092693	0.051958426	0	0.07457445	0.012656875	0.047542245	0.048759196	0.489445566	0	0	0.232970549
FP1705100137DN01	1.81E-45	0.1389261	1.74E-114	1.50E-19	0.020487862	0.114881054	0.225505204	0.059756007	0.420321074	1.65E-11	0.0201227	2.07E-23
FP1705100139LD02	0.06986923	0	1.13E-35	2.59E-51	0	0.012551016	0.028188627	0.264277742	0.541459599	0	0	0.083653786
FP1705100141DN01	0.110525567	0	0.028865934	0.015652162	0.027006735	0.027186831	0.032236838	0.26762629	0.39641465	0	0.012062344	0.082422648
FP1705100143DN01	2.79E-33	0	0.1209544	0.013480529	0.029024699	0.116732339	0.020145857	1.01E-07	0.494679235	2.23E-64	0.042647002	0.162335838
FP1705100145DN01	0	0.022448144	7.17E-116	0	0.026881692	0.220763807	0.253436124	3.47E-08	0.401158162	1.67E-09	0	0.075312034
FP1705100149DN01	0.087605014	0	0.008347807	0	0.027513137	0.137141297	0.23613976	0.101002989	0.260855692	0.011785803	0.057681356	0.071927145
FP1705100153DN01	0.110450616	0	0.078846859	0	0.027744535	0.313116944	0.062834593	0.214174959	0.082630284	0.021563106	0.037966574	0.050671531
FP1705100155DN01	0	0	0.039234059	0	0.011410393	0.115215522	0.090456945	0.088939803	0.24050262	3.90E-22	0.369168395	0.045072262
FP1705100159DN01	0	0.167345378	1.20E-50	0.001137883	0	3.14E-57	0.05392172	0.491864138	0.104688436	0.0210387	0	0.160003745
FP1705100161LD02	0	0.045236861	0.003538694	0	0.006577386	0.093102262	0.145934492	0	0.490941088	0.006086232	0	0.208582986
FP1705100163DN01	0	0	0.060832904	0.002291095	0.056600795	0.182613054	0.118867234	0.083212894	0.35259992	0.001308595	0	0.141673509
FP1705220177LD01	0	0.031387462	0.122817809	0.098150486	0.037531897	1.46E-100	0.063075192	0.116838491	0.294817393	0.043666532	0	0.191714738
FP1705220179LD01	0.253105809	0.070440034	0.063394951	0	0.029763476	0.007927037	0.032998559	0.086728879	0.263390713	0	0.035316416	0.156934126
FP1705220181LD01	0.079574469	0.113829565	1.25E-20	0	0.060359061	0.117533102	0.171581787	0.05112982	0.11711438	0.076284089	0	0.212593728
FP1705220182LD01	7.66E-58	0.04054621	0.285270033	0	0.055181942	0	0.108768571	1.39E-21	0.482733443	0.027499802	2.34E-281	0
FP1705220184LD01	0	0	0.029548513	0	0	0.204114607	0.455635245	2.05E-14	0.212239155	9.88E-19	0	0.09846248
FP1705220185LD01	0	0.032221871	0.068516257	0	0.042728805	0.063861376	0.122175958	0.137502633	0.490296257	0.008920953	0	0.03377589
FP1705220186LD01	0	0	5.74E-20	0.253660789	0.048866154	0	0.005764069	0.162115432	0.422073074	0.015404961	0.074493981	0.01762154
FP1705220187LD01	0.000576395	0.139066841	0.104534985	0.056764153	0.004818576	0.055117089	0	0.227241891	0.142931462	0.088548969	0.012613826	0.167785812
FP1705220188LD01	8.70E-56	0	0.001150682	0.067536979	0.047592876	0.083505685	0.143207391	0.11121644	0.381887027	0.041281803	0.023834768	0.098786349
FP1705220189LD01	0.183016655	0	0.095765583	0.024800091	0.003515717	0.485396759	0.058451537	0.070109817	0.069680201	0	0	0.009263641
FP1705220190LD01	6.42E-11	5.73E-135	7.69E-96	0.040330233	0	0.258511525	0.054261095	0.113153064	0.350110676	0.016384316	0.00109762	0.16615147

FP1705220191LD01	2.15E-39	0.003063949	4.46E-06	0	0.327339871	0.033467276	0.031949841	0	0.578936423	0	0.020609592	0.004628589
FP1705220192LD01	3.28E-65	0	0.039466552	0.009490521	0.012314275	0.205260046	0.147770015	0.192081131	0.39361746	8.59E-30	0	0
FP1705220195LD01	0	1.26E-07	0	8.65E-12	0.038646114	0.106892717	0.159182339	0.257979199	0.226913428	0.046194239	0.002500234	0.161691602
FP1705220196LD01	0	0.033582787	0.056377594	0	0.036878281	0.133267318	0.244366854	7.83E-09	0.412037501	9.82E-25	6.02E-135	0.083489657
FP1705220197LD01	0	0	0.045593467	0.066225379	0.033756094	0.110580349	0.101479966	0.221928788	0.396591645	0.004779142	0	0.019065169
FP1705220198LD01	1.14E-54	0	0.187642009	0	0.019940897	0.019629793	0.045497007	0.345282279	0.203339398	0.033997302	0	0.144671315
FP1705220199LD01	2.93E-23	0.026326577	0.039309537	0.03888161	0.033555654	0.10271944	0.119226616	0.12787933	0.302008421	0.067410221	0.015446429	0.127236164
FP1705220200LD01	0	0	8.16E-166	1.20E-162	0.05364437	0.000257546	1.03E-61	1.18E-60	0.490200626	1.14E-125	1.29E-191	0.455897457
FP1705220201LD01	0	0	0.02150412	0	0.030858477	0.064876134	0.140914951	0.132136726	0.36799143	0.003045344	0	0.238672818
FP1705220202LD01	0.143760256	0.068354513	0.06938216	0	0.130105038	0.138567439	0.089189797	0.115458363	0.197734755	0.02621441	0.003054323	0.018178946
FP1705220203LD01	0	0	0.138477485	0	0	0.075931706	0.158951675	0.18631321	0.339994493	3.86E-26	0	0.100331431
FP1705220204LD01	0.002996691	0.022951384	0.057140024	0.030298527	0.021139299	0.108605188	0.024198257	0.328791594	0.342531745	0	5.09E-149	0.06134729
FP1705220205LD01	2.54E-38	0	0.108672696	0	0.008948767	0.075916881	0.048784118	0.137115938	0.526299963	0.017296848	0.039579515	0.037385275
FP1705220206LD01	0	0	4.15E-57	0.061199799	0.044919226	0.145262757	0.076791569	0.040760285	0.372400827	1.92E-58	0	0.258665536
FP1705220208LD01	0	0.172281343	0.170184453	0	0.014601409	0.060550974	0.081573655	0.239255769	0.261552397	0	6.73E-124	1.36E-20
FP1705220209LD01	0	0	0.187966018	0.086272874	0.008788655	0.022433281	0.074825387	0.372026163	0.161770731	4.72E-119	0	0.085916891
FP1705220210LD01	0.299948327	0	0.054359063	0	0.026183088	0.053174746	0.020196925	0.41965985	0.029901343	0.019193001	0.017033837	0.06034982
FP1705220211LD01	0.13292815	1.96E-13	0	0.101251701	0	0.140057818	0.161124908	4.05E-01	0.059860238	1.01E-90	0	0
FP1705220212LD01	0.285141689	0	0.054145991	0.051563149	0.020492582	0.019788745	0.01570503	0.311999384	0.128192682	0	1.19E-29	0.112970748
FP1705220213LD01	5.08E-51	0.023499065	1.65E-67	0	0	0.079617115	0.100520804	0.014240114	0.606212254	0.05708998	5.87E-25	0.118820667
FP1705220214LD01	0	0	0.063992782	0.072595764	0.044957046	0.053866011	0.023968886	0.176902366	0.538152781	0	0	0.025564363
FP1705220215LD01	9.07E-55	3.84E-50	0.107786434	0	0.03058853	0.021868272	0.063476668	0.063276056	0.532944299	0	0	0.180059742
FP1705220216LD01	0	0.003323291	0.012645504	0	0	0.041827788	0.098337461	0.219646839	0.465649774	0.015296849	1.85E-115	0.143272495
FP1706060153LD01	0.339210607	0	0.103552558	0	0.044084316	0.134640163	0.024853091	0.237281593	0.026521539	0.038239474	0.017144858	0.0344718
FP1706060155LD01	0	0.034656453	2.61E-105	0	0.211762368	0.104988507	0	0.159853475	0.452631103	0	0.036108095	0
FP1706060156LD01	0	0	0	0	0.05173505	1.852746171<br>90467e-321	1.20E-149	0	0.287659859	0	0	0.660605091
FP1706060159LD01	0.106990193	0.012463941	0.149178077	0.053221212	0.067000399	0.049428832	0.08430223	0.149568043	0.218350569	0.009700988	0.023468022	0.076327494
FP1706060160LD01	0.216468781	0.078891842	0.043742456	0.019640709	0	0.042817759	0.066869795	0.316216296	0.20729548	0	1.13E-61	0.008056882

FP1706060161LD01	0	0.094647958	0.034048083	0	0.038771499	0.045372536	0.064518536	0.182253712	0.401656151	0	0.02474802	0.113983506
FP1706060162LD01	3.31E-09	0.050648261	0	4.11E-36	0.01754605	0.29606773	0.170322313	0.168597917	0.296817725	0	0	1.30E-23
FP1706060163LD01	0	0	0	0	0.031293819	0.087067865	0.137756972	0.346382222	0.303656572	0.066696861	0.027145689	0
FP1706060165LD01	1.88E-76	0.00758696	0.045456059	0	0	0.130218821	0.248209596	0.034318297	0.326867927	0.006511678	0.133653518	0.067177144
FP1706060167LD01	0.083779432	0	0.050498173	0.071270915	0.06246681	0.085782551	0.049385974	0.250157855	0.244913822	5.63E-21	0	0.101744468
FP1706060169LD01	0	2.42E-43	0.1042716	5.37E-57	0.018774265	0.064949621	0.092802568	0.134950946	0.575655962	7.13E-40	0.008595039	3.24E-48
FP1706060170LD01	0	0	0.02468401	9.68E-48	0.006496118	0.048785783	0.237615337	0.044611938	0.205006265	0.045185333	0.368731402	0.018883815
FP1706060171LD01	0	0.123416486	0.082579997	0.036543522	0.026585475	0.148421753	0.134218308	0.187430166	0.248479857	0	0.012324437	0
FP1706060172LD01	0	0	0.031435541	1.14E-08	0.044636972	0.125602646	0.107307575	0.096160843	0.52215813	0	1.86E-152	0.072698281
FP1706060175LD01	0	0	0.068890017	0	0.035222324	0.008336326	0.025445743	0.094037441	0.622660105	0.034446601	7.31E-63	0.110961443
FP1706060176LD01	0	0	0	0.114788687	0.043566223	0.051897305	0.025733523	0.04600093	0.430655151	0.00297527	0	0.28438291
FP1706060177LD01	2.04E-31	0.06341652	0.190755212	0	0.029417105	0.045621172	0.072811863	1.10E-11	0.467264278	0	3.91E-07	0.130713458
FP1706060178LD01	0	0.032198781	0.051855661	0	0	0.066594743	0.0985934	0.359133741	0.252200743	0	0.015641074	0.123781858
FP1706060179LD01	1.11E-14	0	0.393522204	0	1.51E-19	2.86E-59	0	0	0.277056137	0.023901738	0.305519921	0
FP1706060180LD01	0	0.049570342	0.047654213	0	0.081614971	0.00117898	0.014298128	0.216148894	0.584279537	1.81E-06	2.87E-24	0.005253127
FP1706060184LD01	9.89E-67	5.73E-35	0.11974858	1.26E-10	0.007012953	0.049979834	7.37E-02	0.019486358	0.422460068	0	0	0.307657062
FP1706060185LD01	0.025109922	0.101903668	0.18077583	0	0.018400701	0.161280167	0.104892839	7.27E-51	0.342022763	0	0	0.065614111
FP1706060188LD01	2.29E-73	0.132405669	0.086437604	0	0	0.02983838	0.066302268	0.101319505	0.49078428	0	0.019532301	0.073379994
FP1706060191LD01	0	0.124809516	2.47E-92	2.09E-64	0.019546689	0.052199932	0.182033631	0.11686606	4.21E-01	0.031167112	1.59E-112	0.052309078
FP1706060192LD01	8.86E-86	0	0.09562333	5.03E-114	0.304925008	0.002176151	1.12E-54	2.78E-19	0.395939808	0	0	0.201335702
FP1706060193LD01	0	0	0	0	0.02514595	0.065523748	0.02917623	0.306689974	0.490103601	0	0	0.083360497
FP1706060194LD01	2.63E-31	0.124338368	0.023701804	0.029106606	0	0.298837547	0.235678731	0	0.091500912	0.067206604	7.22E-61	0.129629428
FP1706060195LD01	5.08E-74	0	0.069298847	0	0.043303124	0.000751277	0.052319506	0.08757948	0.526660728	0	0.013388548	0.206698489
FP1706060196LD01	0	0.002587769	0.080993547	0.014631382	0.005615746	0.067058063	0.226988714	0.165196556	0.414488964	0	0	0.022439259
FP1706060197LD01	0	0	0.07012165	7.24E-82	0.034434047	0.035037321	0.026501068	0.117614434	0.36712623	2.86E-25	1.45E-196	0.34916525
FP1706060198LD01	0	0	0.04661768	4.86E-32	0.002837339	0.085639348	0.110822078	0.2368803	0.206578749	0.007928973	0.043907058	0.258788474
FP1706060199LD01	0	0	0	0	0.139828335	0.111443535	0.040147886	0.562952898	0.114711319	0.030916027	3.49E-193	0
FP1706060200LD01	0.1266488	0.10829138	0.026226835	0	0.030147562	0.014308194	0	0.099215139	0.48082251	0.055973247	0.028354986	0.030011348
FP1706060201LD01	0	0.013571772	0.336345657	0	0	0.060320497	0.076976921	0.002405656	0.330489446	0	0	0.179890051

FP1706060202LD01	0	0	0.069799496	0	0.03313914	0.091627043	0.211443712	0.15310642	0.311946565	0	0.128937623	0
FP1706060203LD01	0	0	0.049668737	0	0.023857711	0.002725176	0.017304008	0.309170348	0.358535985	0.014734183	0	0.224003853
FP1706060204LD01	0.038629878	0.134821578	0.150030288	0.033537526	0	0.14708732	0.110218473	0.131854088	0.178509973	0.050963831	0.006826659	0.017520385
FP1706060205LD01	0	0	0.081368695	0	0.039402742	0.096508455	0.234332063	0.021076073	0.512541354	0.014770618	4.22E-99	0
FP1706060206LD01	0.06407379	0	0.048084876	0.005431379	0.028002768	0.204847751	0.130708216	0.038349344	0.316815306	0	2.00E-92	0.16368657
FP1706060207LD01	0	0	0.106383882	0.011119169	0	0.08786293	0	0.118973682	0.665860378	0	0.00979996	4.75E-195
FP1706060208LD01	0.088669377	0.019319898	0.014096651	0	0	0.078189079	0.054536524	0.309297396	0.422621639	0	0.000195443	0.013073992
FP1706060209LD01	0	1.58E-49	0.150807385	0	0.02946412	0.192284521	0.159905388	0.040108128	0.354625983	3.99E-17	5.84E-44	0.072804474
FP1706060210LD01	0	0.007925971	0.068589372	0	0.028028672	0.062301272	0.048805832	0.237025114	0.547323767	0	3.61E-98	0
FP1706060212LD01	1.13E-157	0.015256996	0.055505781	0.021669007	0.017658343	0.026903023	0.016174309	0.137889734	0.659901272	1.03E-218	0	0.049041536
FP1706060213LD01	0.034309931	7.93E-18	0.061251108	0.002934445	0.001934893	0.446564274	0.208563746	0.00453466	0.153267199	1.13E-29	0.002292961	0.084346783
FP1706060214LD01	0	0	0.136081791	0.019361936	0.016837895	0.167169473	0.139216235	0.06485721	0.299384816	0	0	0.157090645
FP1706060215LD01	1.84E-26	0	8.65E-165	2.61E-113	0.03360084	0.045019665	0.12155133	7.09E-24	0.478520078	0	6.31E-148	0.321308087
FP1706060216LD01	0.00043005	0.121610801	0.060891637	1.50E-11	0	0.093033628	0.043019613	0.078603544	0.350809016	0.017296754	0	0.234304957
FP1706060217LD01	0.123299859	0	0.037655046	0	0.015787919	0.348591958	0.171428855	0.174441135	0.083101469	3.30E-13	0.000707266	0.044986493
FP1706060218LD01	3.44E-71	0	0.046971771	0	0.033886438	0.035736424	0.065160687	4.60E-26	0.469208373	0.042967679	0.023487961	0.282580666
FP1706060221LD01	0.048926364	0	6.17E-208	0	0.001005511	0.131058377	4.28E-44	0.182377869	0.260179272	0	0	0.376452607
FP1706060222LD01	0	0	0.007352808	1.30E-14	0.043705396	0.149709861	0.09937597	0.226098413	0.443750527	4.68E-06	0	0.030002346
FP1706060223LD01	0.034638225	0	0.043575534	0	0.04137926	0.273345777	0.14544407	5.26E-12	0.377253325	0	5.60E-162	0.08436381
FP1706060224LD01	0	0.052818506	0.19979565	0	0.0382254	0.035147253	0.085733913	0	0.555953184	0	0.028545013	0.003781081
FP1706060225LD01	3.91E-148	9.96E-113	6.68E-209	2.11E-120	0.010981696	0.064232922	0.013234309	5.52E-54	0.441171019	0.002930428	0	0.467449627
FP1706060226LD01	1.11E-91	0.018313289	0.057437199	4.89E-22	0.022671159	0.070635267	0.094143522	0.05057293	0.64416133	0.008532488	0	0.033532815
FP1706060227LD01	0.036978354	0.073465979	0.093838229	0.008128117	0.011766161	0.038582077	0.05961354	0.308345016	0.34924129	0.020041237	0	0
FP1706060228LD01	2.75E-56	0	1.93E-01	0	0.0104803	0.00306032	0.024849446	0.083651873	0.188159976	0.042040623	0.069926126	0.384442131
FP1706060229LD01	0	0.121849368	0.013376031	0	0.017631594	0.011944643	0.101923586	0.306670602	0.336240822	3.60E-27	0.014355745	0.076007609
FP1706060230LD01	0	0.057669994	0.049470433	0	0.012720491	0.058125704	0.058481531	0.337313413	0.290585639	0	4.76E-20	0.135632795
FP1706060231LD01	0	0.070214234	0.033480657	0.005802938	0.038891613	0.036968078	0.021780165	0.093762705	0.42098577	0.013636982	0.007309497	0.257167361
FP1706060232LD01	0	0	0.157167605	0	0.008108328	3.39E-25	0.133495223	0.061915066	0.590592254	0	0.048721523	0
FP1706060233LD01	5.64E-14	0.066925243	0	3.83E-02	0.004343993	0.245547934	0.16709276	0.020148011	0.401589789	0	2.65E-43	0.056073294

FP1706060234LD01	0.007463859	0	0.012558911	2.77E-37	1.44E-19	0.346420191	0.286614628	0	0.259140793	0	0.000856185	0.086945433
FP1706060235LD01	0	0	0.049490521	0	0.072479117	0.081545578	0.218507138	0.050006438	0.492753139	0	0.010184658	0.025033412
FP1707040176LD01	0.00986559	0	0.191697668	0	0.001748685	0.017550629	0.10184833	2.23E-01	0.373245438	0.016968914	0	0.064546579
FP1707040177LD01	0	0	0.102575921	0.064177684	0.035612073	0.0619865	0.086963395	0.114183468	0.495372507	0	0	0.039128452
FP1707040178LD01	0	0.065147952	4.79E-08	0.056136045	0.00468815	0.206210729	0.198461847	0.141427219	0.298219578	0.016842536	0.000936647	0.011929249
FP1707040179LD01	0.020447932	0.06995142	0.107303604	0	0	0.140197591	0.086894479	0.151079677	0.349245692	0.016193775	0.058685832	2.27E-101
FP1707180231LD01	0	0.124329436	0.08179429	0.010216716	0.004765839	0.081704852	0.033163395	0.362686266	0.256559919	0.01044976	0.034329527	0
FP1707180232LD01	0	0.119219811	0.052790493	0.095518547	0	0.009858434	0.122649753	0.351492419	0.155479491	5.24E-49	0.001508471	0.091482582
TI1706140111LD02	7.72E-31	0	0.137806507	0.039012639	0.003107809	0.062799117	0.127387922	0.043633002	0.517840896	1.30E-61	0.003425722	0.064986386
TI1706140113LD03	0	5.20E-69	0.05499905	4.29E-48	0.004734593	0.417674397	0.201283835	0.015774754	0.263938332	0.028049319	9.15E-101	0.013545719
TI1706140115LD02	0.175691651	0	0.038782914	0	0.052134441	0.174742729	0.069934237	0.323396712	0.036468366	8.97E-41	0.097962799	0.03088615
TI1706140117LD01	0	0.013471726	3.46E-110	0	0.01521601	0.087589962	0.093107601	0.216046769	0.448600831	3.25E-48	0.009077958	0.116889144
TI1706140119LD01	3.29E-36	0	0.096899892	0	0	0.1002009	0.073493372	0.181231339	0.476964668	0	0.047549341	0.023660487
TI1706140121LD01	0.049519943	0.024437038	0	1.15E-45	0	0.01556863	0	0.480311315	0.430163074	0	0	0
TI1706140123LD01	1.34E-91	0	1.10E-75	0	0.292972206	0.046478357	0.043186571	0	0.615460416	0	0.001897107	5.34E-06
TI1706140125LD01	0.003147098	0	0	0	0	0.00506009	0.089811155	0.434848507	0.364521825	0.035251497	2.74E-113	0.067359828
TI1706140127LD01	0.17607497	0	0.046010972	0	0.0221115	0.204631777	0.04363073	0.281765498	0.201158504	0	0	0.02461605
TI1706140129LD02	0.115171821	0	0	0	0.017834003	0.208299179	0.157471601	0.027241672	0.287949133	0	0	0.186032591
TI1706140131LD01	0.038178426	0.09024081	0.071589965	0.019694348	0.02219409	0.280284556	0.126878828	0.098151603	0.207261834	0.006232473	8.23E-76	0.039293067
TI1706140133LD01	0.005293144	6.14E-11	8.71E-06	0	1.06E-77	0.483523012	0.353762405	0.00E+00	0.15741273	0	0	3.28E-159
TI1706140135LD01	1.13E-19	0.010613741	0.109648657	0.004117375	0.058418145	0.083015	0.154235535	0	0.416275252	0	1.13E-91	0.163676294
TI1706140137LD02	0	0.044456462	6.83E-02	0	0.008610431	0.070267912	0.176440177	0.017018657	0.477657068	0	0.024214182	0.1130846
TI1706140139LD01	0.051424677	0	0.070206732	0	0.003283512	0.309097417	0.303265607	0.14285	0.099334764	0	0.009914043	0.01062325
TI1706140141LD01	0	0	1.26E-34	0.027386137	0.002380793	0.135769225	0.109335919	0.189061456	0.392694945	5.21E-50	4.22E-133	0.143371526
TI1706140143LD01	8.51E-82	4.49E-302	0.051957644	6.29E-71	0.009221645	0.027454358	0.0675257	0.211288445	0.557630759	4.44E-125	9.44E-68	0.074921448
TI1706140145LD01	0	0	0.100762635	0.013664438	0.008794726	0.090070109	0.009847605	0.610666674	0.092254613	0	0.069471484	0.004467716
TI1706140147LD01	0	0	0.024595307	0	0.014787292	0.005149221	0.072057522	0.044854625	0.138468273	0	0.699625381	4.62E-04
TI1706140149LD01	0.053705286	0.107671781	0.058817148	0.032281705	0.048705497	1.59E-01	0.101785115	0.049586043	0.276669236	1.43E-22	1.11E-30	0.111342391
TI1706140151LD01	2.13E-22	0.046214842	2.00E-33	0.000222157	0.031685598	0.047420997	0.052845941	0.126843917	0.444947363	0	0	0.249819184

TI1706140153LD01	0.000685867	0.001944333	0.028007679	0.016187937	0.023209073	0.466829994	0.254097492	0.014148927	0.17454801	0	9.25E-30	0.020340688
TI1706140155LD01	0	0.022911939	0.05830382	0	0.012296738	0.023475355	0.003195653	0.390191879	0.340773462	0	0.077945997	0.070905157
TI1706140157LD01	0	0.019081183	0.074535501	1.13E-05	0.156186489	0.115953018	0.067752264	0.09053024	0.475945819	0	0	4.19E-06
TI1706140159LD01	0.160852806	0.003021712	0.056493623	0.026448821	0	0.137855705	0.083947739	0.143911328	0.177941979	0.025916482	1.56E-36	0.183609807
TI1706140161LD01	0.00114227	0	0.070107726	0	0.006882072	0.364592977	0.334530462	3.09E-15	0.21228602	0	0.002649306	0.007809167
TI1706140163LD01	0.04735068	0.025203292	0.015492259	0	0.017809584	0.157213222	0.198313449	0.157356841	0.305828467	0.002523787	0.005630901	0.067277518
TI1706140165LD01	0.045077837	0	0.057535474	0	0	0.094867177	0.201269195	0	0.540545443	0	8.448522543 88532e-322	0.060704874
TI1706140167LD01	0.004624066	0	0.101518231	0	0	0.271405784	0.111102947	0.183289455	0.226725137	0.031607044	0.018769525	0.050957812
TI1706140171LD01	0	0.002367573	0.034930883	0.021003736	0.013333329	0.149851171	0.354252809	0.146257565	0.263257849	0.008497089	0	0.006247995
TI1706140173LD01	0.00767177	0	0.056612034	1.08E-60	0.048411297	0.332059962	0.186680462	0.158261948	0.206507166	0.00208251	0	0.001712853
TI1706140175LD01	0	4.21E-26	2.20E-122	0.044766407	0.011199885	0.108547974	0.208569355	0.113378714	0.467179879	0.018121758	0.028236027	8.80E-111
TI1706140177LD01	1.77E-62	0	0.017608659	1.41E-08	0.035332395	0.194095272	0.172022304	0.035787774	0.413733211	6.09E-09	0	0.131420366
TI1706140179LD01	0	0	0.11178203	0	0	0.28180684	0.183467781	0.114742242	0.272086129	0	0.005544319	0.03057066
TI1706140181LD01	1.72E-72	2.05E-14	0	4.61E-38	0.024730798	0.105213949	0.060340103	0.001420949	0.430639667	0.001168008	6.62E-92	0.376486527
TI1706140183LD01	3.23E-86	0	0.029460854	0.061046414	0.045543653	0.141466775	0.069387239	0.029375363	0.365906258	0	0	0.257813443
TI1706140185LD01	0	0	0.152258264	0.016209625	0.010105934	0.063327983	0.054082966	0.211001041	0.362086812	0	0	0.130927375
TI1706140187LD01	0	0	0.05796963	0	0	0.168681103	0.142240683	0.313764642	0.311755141	0.000249003	3.41E-66	0.005339797
TI1706140189LD01	0.113334468	0.120222175	0.075971858	6.65E-58	0.005759417	0.041146268	0.086239892	0.206874975	0.211324814	0.019762082	1.46E-42	0.119364052
TI1706140191LD01	0.041396586	0	0.161843619	0.063120215	0.067923809	0.020605706	0.000492666	0.429332648	0.08744937	0	0	0.127835382
TI1706140193LD01	4.25E-95	3.06E-09	0.027016479	0	0.093797445	0	0.045796333	0.230897242	0.410546347	2.43E-80	1.34E-58	0.191946151
TI1706140195LD01	0	0	0	1.27E-157	0.036911937	0	6.89E-05	0.391933333	0.016631647	2.07E-28	0	0.554454142
TI1706140197LD01	0.210765016	0	0	2.14E-67	0.003701071	0	0.081749281	0.33462278	0.205363207	0.028692541	0	0.135106104
TI1706140199LD01	0.111181113	0.0571066	0.014553018	0.010399858	0.017251348	0.16850696	0.065494849	0.280202366	0.114972935	0.063572281	0.096758672	0
WGC106522DB	0.063647138	0.108599904	0.044231314	0	0	0.032286464	0.01859695	0	0.5750206	0	0	0.15761763
WGC106524D	0	0.007757712	0	3.32E-02	0.014226497	0.333639804	0.274253655	0.050896382	0.213331235	0	0	0.072691338
WGC106526D	0.030959003	0	0.034422057	0.038473363	0	0.131913238	0.129595789	0.561924865	0.072711686	0	0	0
WGC106528D	0	0.090649871	0.025657666	0.022662139	0.037851426	0.0037904	0.114306964	6.74E-23	0.4949451	0	0.074090022	0.136046411
WGC106530D	0.009614986	0.014556308	0.088286175	0.070347909	0	0.030942137	0.125883365	0.17049699	0.485169801	0.00470233	3.37E-39	0

WGC106534D	0.278875783	0	0.022750484	0	0.02427465	0.100824887	0	0.212204147	0.125147099	0.017170631	0.088451879	0.130300439
WGC106536D	0.012166271	0.171283502	0	0.063912054	0.034668915	0.029466093	0	0.01669708	0.559566874	0	0.009596283	0.102642928
WGC106538D	0.004943652	0.04265523	0.116978311	2.81E-13	0	0.132128275	0.14041868	0.171420537	0.302018291	4.21E-42	3.30E-133	0.089437025
WGC106540D	1.79E-07	0.011017633	0.021399981	1.13E-42	0.038792823	0.107711041	0.142240136	5.55E-45	0.427206228	0	1.99E-91	0.251631979
WGC106542D	0	0	9.32E-42	0.032180684	0.063512323	0.141933819	0	0.316691907	0.291003446	0	0.018274006	0.136403815
WGC106544D	0	0.117034673	3.06E-22	2.73E-12	0	0.138188549	0.102454967	0.249765668	0.237512881	0.015549916	0	0.139493345
WGC106550D	0.127090025	0	0.173832133	0.2365174	0.039975602	0.0647492	0.082382834	5.93E-19	0.074323443	0	0.129170296	0.071959066
WGC106552D	0	9.17E-09	0.135093138	0	0.00611697	0.083616002	0.168754462	0.008123005	0.461210103	0	0.137086312	0
WGC106554D	0.032444089	0	0	0	0.027282081	0.195345597	0.16002088	0.28840536	0.224735946	0.028075127	0.039776125	0.003914794
WGC106556D	0	0	7.80E-45	0.008210947	0.05814336	0.159119816	0.129334661	0.239174401	0.306418686	0.060635235	0.037087625	0.001875268
WGC106558D	3.74E-11	0	0.127892555	0	0.03909903	0.135413796	0.12409836	0.171564317	0.395788091	0.006143852	0	4.19E-80
WGC106560D	9.43E-51	0	0.041078842	0.010103372	0.015047967	0.17772345	0.276256999	0.078916581	0.282147637	0.056657514	0	0.062067638
WGC106562D	5.38E-05	0.013389541	0.016592908	5.48E-25	0.014084602	0.402190108	0.433734182	1.14E-28	0.085029951	3.38E-41	0.008577658	0.026347256
WGC106564D	3.28E-76	0	0.353142771	0	4.19E-25	0	0.106006537	0.148495002	0.385316806	0	0.007038885	0
WGC106566D	0.038272615	0	0.080669032	0	0.014720778	0.178625935	0.087101478	0.110270591	0.370437692	0	5.99E-21	0.11990188
WGC106568D	0	0	0.06126633	0.042079459	0	0.068880499	0.068663504	0.211286376	0.362535416	0	0	0.185288416
WGC106570D	1.14E-128	6.81E-107	0.031048125	0.031204901	0.01380086	0.040564839	0.073767165	4.82E-70	0.543737388	0.025156413	8.64E-114	0.24072031
WGC106572D	2.41E-16	0.069500028	0.073923289	0	0.026105907	0.089995576	0.15122161	0	0.397677234	0	0.058855712	0.132720644
WGC106574D	0.092978108	0	0.078925071	0	0.038959763	0.242570537	0.105914726	0.024582122	0.316489786	0	0.015785916	0.083793972
WGC106576DB	0.311612268	0.005728703	0	0.007357336	2.14E-278	0.044089018	0.149881367	0	0.475367613	0.005963694	0	0
WGC106578D	0	0.173885785	0.067458217	0.024033782	0.131706066	0.081578312	0.051400726	0.273413144	0.196523966	0	6.01E-43	8.00E-86
WGC106580D	0	0.05528372	0	0.068068302	0.00676978	0.484312301	0.2106112	0.05703679	0.104122545	0	4.85E-85	0.013795361
WGC106582D	0	0.011565852	0.112840113	0	0.014106478	0.150682689	0.090522549	0.035000989	0.41986052	9.32E-17	0	0.16542081
WGC106584D	0.079353674	0	0.031590227	0	0.008690871	0.097014224	0.028031954	0.299339362	0.349676783	0	0	0.106302904
WGC106586D	0	0.098051695	0	0.00E+00	0.041434988	0.008826669	0.010492715	0.53311404	0.190550803	0.020943799	0.096585292	3.29E-121
WGC106588D	0	0.027834383	0.042107486	0.017226934	0.108620002	0.139859757	0.075456615	3.08E-22	0.486041366	0	0	0.102853458
WGC106592D	0	0	0.06534189	1.67E-51	0.010770879	0.171178222	0.236513959	0.198558418	0.271417944	0.046214791	3.90E-06	1.86E-55
WGC106594D	1.62E-34	0	0.133414558	0.016005107	0.042263516	0.004706724	0.078261365	0.087390237	0.453154604	0.042763741	0.045431136	0.096609012
WGC106596D	0.012142161	0	0.188678205	0.073291611	0	0.059615454	0.023971405	0.178620409	0.448054989	0.012036782	0.003588984	0

WGC106598D	0.162503744	0.045644332	0.015121122	0	0.041566001	0.087195397	0.027680923	0.284735944	0.209218438	0	0.03415552	0.092178579
WGC106600D	7.18E-46	0.156513922	0.021543664	0.171498201	0.029283984	0.179552466	0.167985047	0.011802428	0.172230932	0.089589355	1.19E-138	0
WGC106602D	0.000112651	0.013585578	0.104576641	0	0.005355059	0.118898958	0.07476003	0.206011356	0.393349441	1.07E-104	0.040227403	0.043122883
WGC106606D	0	0.062213861	0.013273928	0.022437656	0.009918036	0.398663925	0.188530997	0.025615153	0.279346444	0	1.95E-111	1.28E-73
WGC106608D	0.009225144	0.100603762	0.016487454	0	0	0.10193038	0.029758482	0.205584484	0.234825407	0.019613722	0.064084487	0.217886678
WGC106610D	2.62E-110	0.004933956	1.86E-74	0.045393031	0.006595723	0.126125384	0.209338967	0.288945907	0.318598073	0	0	6.90E-05
WGC106612D	0.033587247	0	3.76E-29	0.012579053	0.028003834	0.088166851	0.148075041	0.244846486	0.370776699	0	0.030954426	0.043010363
WGC106614D	3.46E-68	0	0.052773632	0.008711303	0.02894483	0.043937804	0.146642508	0.172747848	0.3642479	1.20E-59	0	0.181994175
WGC106616D	0.010968049	0.073773756	3.12E-10	3.46E-64	0.025583295	0.234615324	0.241803643	0.12058988	0.246899344	0.001499653	0	0.044267055
WGC106618DB	2.19E-45	5.34E-100	0.042468402	3.71E-05	0	0.11293732	0.238363038	0.111439757	0.351167182	0.008155358	0	0.135431853
WGC107774D	0.011048782	0	0.098176418	0.047324588	0	0.06884636	0.126903527	0.118837713	0.459412176	1.65E-24	1.52E-54	0.069450435
WGC107782D	1.56E-88	0.020549425	0.122085801	7.16E-15	0	0.088369928	0.034673514	4.56E-48	0.574805702	0.000103607	0	0.159412023
WGC107786D	0	0	0.080969767	0	0.026355537	0.028770489	0.109620693	0.047402335	0.528435686	0.01062172	0.12807906	0.039744713
WGC107788D	0	0.284868036	3.51E-138	0	0.014866884	0.001985679	1.80E-39	0.217328246	0.437813386	4.33E-45	0	0.043137768
WGC107790D	0	0	0.013455793	0.043675462	0.022041628	0.01320175	0.157882951	0.54180203	0.159985078	0	0.047955308	0
WGC107794D	0	0	8.35E-78	0.005388656	0.022552158	0.192412597	0.135171069	0.220409873	0.16743584	0.05170242	0.137877962	0.067049426
WGC107798D	3.96E-73	0.0877685	0.160379746	0.022661683	0	0	0.005909473	0.093519553	0.25870169	0.000331216	0.090267623	0.280460516
WGC107800D	1.82E-37	0.065088519	0.171268997	0	0.076558633	0.026618349	0.091770804	0.213685544	0.157291609	0.031603598	0.07835715	0.087756797
WGC107802D	0.014942996	0.054181056	0.035643074	0	0.055350746	0.118938836	0.037802468	0.430159423	0.207263389	0	0	0.045718011
WGC107806D	0	0	0.01943419	0	0.026352591	0.000801134	0.001554148	2.56E-61	0.558359838	0.000538696	0.046940392	0.346019011
WGC107808D	0	0.157947332	0.066551088	0.150861138	0	0.018117125	0.024234662	0.457113517	0.076505277	0.026857932	0	0.021811929
WGC107812D	2.66E-52	0	0.087722612	0.016756315	0.025043041	0.040520318	0.07522403	0.224379263	0.419999894	0	2.12E-89	0.110354527
WGC107814D	0	0	0.035351573	0.156230297	0.02517063	0.246662597	0.196524803	0.085230785	0.254829315	0	0	1.96E-64
WGC107816D	0	0	0.016871723	0	0.057686075	0.170577061	0.087773245	0.27163027	0.34124149	1.00E-10	0.001246752	0.052973384
WGC107820D	0.186616096	0	0.025843355	0	0.014040303	0.062108484	0.02524513	0.37198384	0.12843609	0.069213325	0.052055574	0.064457803
WGC107822D	0.079165246	0.11411526	0	0.018907532	0.126940623	0.033286578	0.01482702	0.319906774	0.025654185	0	0.079435746	0.187761034
WGC107824D	0	0.040718048	1.33E-30	1.02E-58	0.017005821	0.182952109	0.129193673	0.097398125	0.532300166	0.000432057	2.88E-75	5.64E-54
WGC107826D	8.81E-26	0.095601775	0.069788468	1.39E-51	0	0.015152082	0.044876951	0.053852577	0.655120837	0	0.011119873	0.054487437
WGC107828D	0.091729117	0.021287213	0.005584023	0	0.023407445	0.494990184	0.17961279	0.069510792	0.113878437	0	5.08E-16	4.40E-62

WGC107830D	4.47E-120	0.011986474	0.227187764	0.033528925	0.01412604	0.034070365	0.039642229	0.180090448	0.309376733	0	0	0.149991021
WGC107832D	4.80E-49	1.37E-52	0.043999344	0	0.065036409	0.026633587	0.008579098	0.129596452	0.475874623	0.010165037	0.051196452	0.188918999
WGC107834D	1.00E-100	0	9.98E-16	0	0	0.008752143	0.083535038	0.412728297	0.471693439	0.002327022	0.020964061	3.72E-41
WGC107836D	0.011576399	0	0	0	0.012999315	0.286388097	0.458763656	0.049635126	0.176660327	0	0.003977081	1.68E-45
WGC107838D	0.008723072	0	0.140463406	2.32E-11	0.038528034	0.148169706	0.038759573	0.144038224	0.432374363	0	6.37E-193	0.048943622
WGC107840D	0	7.73E-05	0.078127675	0.019350499	0.038369072	0.123560519	0.33625899	0.057303813	0.28292201	0	0	0.064030078
WGC107842D	0	0.127891859	0	0	0.025209124	0	0.023632998	0.478723583	0.259190547	0.04244129	0.031266201	0.011644397
WGC107844D	1.22E-25	0	0.136270092	0.010250865	0.03847527	0.032248437	0.314455587	0	0.439453012	0.007829223	0	0.021017514
WGC107846D	0.028499892	0.074200365	0.074708237	0	3.44E-93	0.462139682	0.247648282	0	0.080411088	0	3.29E-72	0.032392454
WGC107848D	0.020440681	0	0.017140778	0.03673471	0.005667036	0.060776956	0.219126606	0.466625176	0.173483468	0	0	4.59E-06
WGC107850D	0.210473744	0	0.158878643	0	9.62E-240	0.011351801	0.147684872	0	0.216436707	0	0.255174235	0
WGC107852D	1.07E-175	0	0.029044305	0	0	0.086138558	0.01102061	0.407523323	0.423494932	0.036623862	0	0.006154409
WGC107854D	0	0	0.069334088	0.023506069	0	0.136199251	0.142239795	0.037612961	0.498370528	0.031220235	0	0.061517073
WGC107856D	0	0	0.247091775	0.125979033	3.28E-02	0.02228508	0.111107289	0.080432959	0.327345612	0.02873386	0	0.024254588
WGC107858D	0	0	0.01093858	0	0.014371394	0.02332359	0.114849008	0.316120233	0.285428505	0.010429112	0.02947432	0.195065258
WGC107860D	0.207852769	0.038941481	0	0.005789983	0.113935661	0.08991491	0.01520725	0.057211431	0.386504707	0.016157607	0.00780691	0.060677291
WGC107862D	0	0	0.237305051	0.0036395	0.067513638	0.097674462	0.065169941	0	0.452791069	9.34E-31	0.0068537	0.06905264
WGC107864D	2.05E-44	1.26E-121	0.077362629	0	0.018093618	0.15483298	0.148025242	0.131168607	0.320487551	5.03E-49	0.027110724	0.122918649
WGC107866D	0	7.76E-61	0.00E+00	0.195064169	0.232280068	0	1.27E-83	0.287064594	0.285591168	0	0	0
WGC107868D	0	0	0.050961755	0.034035849	0.080892553	0.128569379	0.174427361	0.109952517	0.421160586	6.36E-35	0	6.97E-99
WGC107870D	0	0	0.07952138	0	0	0.144219654	0.07843114	0.453922501	0.232820517	1.38E-13	0.008852129	0.002232679
WGC107872D	0.00074029	1.93E-27	0.145626023	0	0.063035784	0.076766634	0.164893503	0.434945716	0	0	0.002490855	0.111501194
WGC107874D	0.061618947	0	0.180933053	0.049397977	0.006795222	0.086093382	0.081813267	0.073302976	0.337889105	0.028662581	4.67E-09	0.093493486
WGC108520D	0	0	0.128212345	0.204540188	0.012187062	0.072655386	0.12692022	0.068237834	0.195768029	0.000166496	0.074228797	0.117083642
WGC108522D	0.551046846	0	0	0	0	0	0.057150097	7.50E-19	0.257822672	0	0.133980384	0
WGC108526D	0	3.56E-22	0	3.85E-18	0	0	0	0.881061946	0	0.104097109	0	0.014840945
WGC108528D	0	0.011725755	0.002802906	0	0	0.044300556	0.049517646	0.002401135	0.691798112	0	0.084104221	0.113349668
WGC108530D	7.29E-69	0	0.045472253	0	0	0.106442267	0.060376947	0.217693925	0.314680913	0	0.140501525	0.11483217
WGC108532D	6.78E-08	0.010353328	0.15111432	0.166180989	0.010608625	0.082694865	0.064082242	0.093122119	0.229977666	0	0.051480116	0.140385663

WGC108536D	3.17E-36	0.015892687	0.119909319	0	0	0.00266275	0.046455812	0.219388845	0.514570424	0.003032115	0.078088048	3.02E-63
WGC108538D	1.13E-40	0	0.056666173	0	0.050493839	0.102843711	0.05526876	4.24E-07	5.50E-01	0.037766674	0.025389029	0.121222457
WGC108542D	1.77E-113	2.26E-55	0.048994069	1.86E-23	0.02390719	0.047434453	0.018492297	0.341182607	0.370409221	2.24E-121	0.09877433	0.050805833
WGC108544D	0.126337432	0.024912175	0.12116754	0	0.059876628	0.184402615	0.087522798	0.184888336	0.152763812	0.02882778	0.029300884	0
WGC108546D	0	0.171075985	0.145375098	0	0.023847365	0.008729686	0.033048833	0.09642803	0.422297242	0	0.099197762	0
WGC108548D	0.001862131	2.15E-40	0.014968178	0	0.00225603	0.217766734	0.38120015	0.016228084	0.305833876	0.044803309	0.015081508	6.14E-20
WGC108550D	5.31E-105	0.003978464	1.27E-96	0	0.032096382	0.021075998	0.070215139	0.019332986	0.732646218	0	0.040772932	0.079881882
WGC108552D	0	0.018716357	0.233661953	0.013189261	0.020787216	0.095893042	0.144806786	0.148565515	0.203901416	0.001911449	0.047541187	0.071025819
WGC108554D	2.41E-55	0	0.030337199	0	0	0.176655921	0.053349342	5.58E-37	0.296266411	0.047377702	0.027469862	0.368543563
WGC108556D	2.27E-61	0.059673167	0.118319028	0.070171714	0.012449887	0.05736362	0.056070322	0.2160919	0.39518439	4.64E-41	0.014675971	0
WGC108558D	0.094997752	0	0.077766426	0	0.03384629	0.061995219	0.211407621	0.15601462	0.282847971	0.007747681	0.072312608	0.001063812
WGC108560D	0.013141094	0.13185409	0.019666312	0.005250241	4.88E-44	0.42639235	0.190872274	0.000537036	0.210182021	0	1.94E-28	0.002104581
WGC108564D	6.72E-35	0.035956808	0.043606652	0.004647843	0.032126959	0.008738299	0.035945497	0.407236908	0.305594221	0	0	0.126146814
WGC108566D	0.081223752	0	0.005067847	3.43E-12	0.04372539	0.073593734	0.399136771	0.253394419	0.130669505	0	0.013188581	8.83E-82
WGC108568D	0.120613375	0.050055574	0	0	0	0.067876226	0.057074365	0.294053579	0.084870282	0.077585849	0	0.24787075
WGC108570D	0	0.113180369	0.024813744	0.007780871	3.02E-10	0.27581864	0.420958745	0	0.088757211	0	0	0.06869042
WGC108572D	0.090169138	0.163818641	0.063822484	4.09E-81	0.006329783	0.194901619	0.012997915	0.247463943	0.140089768	0	0.005708997	0.074697713
WGC108574D	0.062613147	0	0.143150259	0	0.103167655	0.021911202	0.046987415	0.010712437	0.375530856	0.018441419	1.39E-76	0.217485608
WGC108576D	3.23E-37	0	0.097022585	0.104396327	1.82E-01	1.78E-95	0.082726871	0.450111035	0.083339663	0	0	0
WGC108578D	0	1.55E-33	0	0.217600437	0.006029749	0	0.046491042	0.71133969	0	0	0	0.018539082
WGC108580D	2.84E-33	1.65E-69	0.10827149	0.000577265	0.030912044	0.176798705	0.113164586	0.011654064	0.388094088	7.98E-48	0	0.170527759
WGC108582D	0.018719138	0	0.078000607	0	0.046051837	0.137860626	0.085159986	0.142752399	0.308357559	0	0.043519449	0.139578399
WGC108584D	0.258005458	0.0027113	0.044775391	0.079842175	0.021589685	0.281557796	0.054167688	0.022631927	0.123700234	0.005213969	0.049972648	0.055831729
WGC108586D	0	0	8.84E-66	0.14677943	0	8.695555366 80594e-322	0.038329961	0.597139381	0.217751227	0	0	0
WGC108590D	0	8.92E-07	0.00E+00	3.43E-38	0.039770236	0.247773874	0.168435373	6.53E-19	0.394974066	0	0	0.149045559
WGC108592D	0	0	0.119793632	0	0	0.033712794	0.068323668	0.264839498	0.223711234	0	0.060104756	0.229514418
WGC108596D	0	0	0.166127047	0.22876075	4.34E-55	0.036984138	0.122375005	0.275811396	0.051320135	0	0.062822942	0.055798588
WGC108600D	0	0	0.000339048	0.031795811	0.027220952	0.201574338	0.203574799	0.106288819	0.40935746	0.007135907	6.55E-39	0.012712866

WGC108602D	0	0	0.088103319	0	0.014892092	0.179152648	0.187588317	8.29E-37	0.36992578	2.21E-15	0.024654893	0.13568295
WGC108604D	0	0.060827182	0	4.00E-22	0	0.453345525	0.278857741	3.76E-29	0.154716201	0	0.00781928	0.044434071
WGC108606D	0	0.098932415	0.085401262	0	0	0.087116666	0.082193059	0.167373229	0.47659523	0.002388139	0	1.90E-72
WGC108608D	0.067761949	0	0.018489146	2.45E-84	0.037311038	0.036604838	0.009378977	0.068238341	0.73300667	0	1.39E-112	0.029209042
WGC108614D	1.33E-07	0.086438485	2.98E-31	0.067857374	0.00347276	0.150046016	0.154489314	0.205338551	0.322814164	0	0.009543203	0
WGC108620D	1.16E-51	0.030636629	0.126083443	0.051894853	0.046566314	0.062042266	0.077212649	0.149847931	0.314211215	0.004635135	0.027749665	0.1091199
WGC108624D	0.174161333	0	4.16E-69	0	0.024040099	0.053090006	0.082544596	3.08E-58	0.224345903	0.050636448	0.139495006	0.251686609
WGC108815D	5.72E-42	0	0.091183656	0	0.022889591	0.255643205	0.074298766	0.126286755	0.31390001	0.001018353	0.006236381	0.108543282
WGC108817D	0	0	0.073338742	0.011416445	0	0.275918152	0.226226281	0.244965949	0.14387992	0.024254512	5.53E-59	0
WGC108819D	0.098851588	0	0.04684715	0.006562436	0.146935683	0.034076432	0	1.15E-11	0.666726711	4.38E-52	0	0
WGC108825D	0.037762483	0.096450935	0.04143336	0.019827194	0.044417053	0.327828911	0.175082305	0.02326261	0.161727866	0	0	0.072207282
WGC108827D	0	0	0.123944599	2.05E-33	0.005014724	0.010929416	0.025182227	0.382189526	0.374014162	0.035388708	6.85E-56	0.043336638
WGC108831D	0	0	0.059393471	0.054003666	0.15581701	0	0.036797214	0.221479766	0.327831906	0	0.144676966	9.66E-133
WGC108833D	0	0	0.060364016	0.0383579	0.053309712	0.060811022	0.144281777	0.145192253	0.367147163	0	2.07E-14	0.130536157
WGC108835D	7.87E-93	7.33E-168	5.19E-171	3.21E-188	0.016621847	0.038624547	0.105199252	9.89E-80	0.485240223	0	0	0.35431413
WGC108837D	0	0.013108438	0.065438344	0	0.06337281	0	0.071629671	0.206002409	0.369913862	1.04E-14	0.02256005	0.187974416
WGC108841D	2.74E-79	3.89E-37	0.040573609	0	0.025944005	0.103271335	0.157760775	0.220850636	0.141654735	0.095898487	0.008065868	0.20598055
WGC108845D	0.153781815	0	0.145540137	0.098896291	0.023449263	0.012742894	0.079729493	0.033557797	0.219356937	0.065722807	0.029200238	0.138022328
WGC108847D	1.01E-13	0	0.056537567	0	0.027312192	0.065902104	0.054012481	0.176714471	0.386060438	0	0.233460746	0
WGC108849D	0	0.021843928	0.040203969	1.76E-17	0	0.146553273	0.218480321	0.222821515	0.219922037	2.36E-32	0	0.130174955
WGC108851D	1.47E-23	0.118153795	0.07570901	0.04157573	6.91E-14	0.087484426	0.045155581	6.81E-56	0.468154449	0	0.00141851	0.162348499
WGC108853D	0	5.70E-283	0.085263858	0.416727261	0.033546876	0.139541473	0.056715704	0.04821509	0.202902096	0.002329015	0.014758627	1.33E-21
WGC108855D	0.052115788	0	0.047981729	0	0.01270049	0.250160782	0.263817325	0.05572666	0.1620691	0.01763634	0.040442066	0.097349719
WGC108857D	0	0	0.071925976	0.010142448	0.021410298	0.162283668	0.246933099	2.09E-60	0.311969827	0	1.86E-15	0.175334684
WGC108859D	2.81E-31	0.043462883	0.004800041	3.47E-40	0.006876558	0.079616314	0	0.01008426	0.42652657	0.037036487	0.053255713	0.338341174
WGC108861D	7.23E-71	0	0	0	0	0	0.020099529	0.071167705	0.461647904	0.126339353	0.28359029	0.037155218
WGC108863D	1.62E-46	0.1039408	9.16E-17	0.050770737	0.002598041	0.028918073	0.160938699	1.26E-25	0.497237479	4.43E-66	0	0.155596171
WGC108865D	4.19E-33	0	0.084865032	0	0	0.08019542	0.170939534	0.214343006	0.222859958	0	0.120841754	0.105955297
WGC108867D	0	0	0.083739969	0.070354287	0.049618702	0.176893431	0.119473369	0.18336451	0.189295003	0	0	0.127260731

WGC108869D	4.38E-71	0.000906221	0.138265739	0.428875725	0.078073149	0	0.011652854	0.021296813	0.082311056	0.067851397	0.043901508	0.126865537
WGC108871D	0	0.042428321	0.052306237	0.061799927	0.006232134	0.134525929	0.120655913	0.029692279	0.518076348	0.000714017	0	0.033568894
WGC108873D	0	0	0.167244882	0.002148155	0	0.123611297	0.096200874	0.088801046	0.337950383	0.017608111	5.97E-87	0.166435251
WGC108877D	0.10620104	0	1.51E-25	0	0.014066456	0.094595993	0.155272457	0.168059127	0.329860793	0	0.004331506	0.127612627
WGC108879D	2.96E-39	0	1.04E-13	0.060583045	0.023653842	0.16705065	0.185711153	0.091854059	0.368233501	0	0	0.102913751
WGC108881D	0	0.111983514	0.213282601	0	6.52E-02	0.036046329	0.073478445	0.017658755	0.183945468	0	0.071323676	0.227095882
WGC108883D	0.004108585	0.137481849	1.41E-02	2.18E-30	0.067347432	0.06129291	0.036017819	0.114482492	0.467801294	0	0.016943445	0.080431032
WGC108885D	0.129714619	0	0.079309853	0.068741973	0.058363674	0.112347501	0	0.19840996	0.24168129	0	0.018356855	0.093074274
WGC108889D	1.48E-80	0	0.094470514	0.020297553	0.065368847	0.121584044	0.157109694	0.125850025	0.37315795	0.026448189	4.36E-48	0.015713183
WGC108891D	0.016838735	0.149900188	0.080429614	0.029830291	0	0.013264743	0.093975509	0.047491701	0.340534074	0	0.005712272	0.222022873
WGC108893D	2.71E-13	0.003884616	0.20897803	0	0.061264458	0	0.038963813	0.120412256	0.450361756	0	0.116135072	0
WGC108895D	0.121805154	0	0.120005716	0	0.023783898	0.085034921	0	0.18062102	0.38997979	0.010587101	0.008149671	0.060032729
WGC108897D	0.181738385	0.033030147	0.021986079	0	0	0	0.065800384	0.056780581	0.548805215	0.06371484	0	0.028144368
WGC108899D	0.094689713	0.012980922	0.069670324	7.38E-49	0	0.061380681	0.034525815	0.20941924	0.3782615	0.052106612	4.28E-111	0.086965193
WGC108901D	0	0.001741352	1.23E-33	0.092188964	0	0.254695344	0.26077655	0.058986106	0.18151071	0	0.023603668	0.126497305
WGC108903D	0.026027742	0	0.09695607	2.38E-61	0.013342364	0.164181637	0.104742759	0.131753624	0.401576349	0.016411919	0	0.045007537
WGC108905D	0	0	0.040875725	0.001164003	0.01454137	0.144963919	0.16938536	1.98E-22	0.501968855	0.019200393	0.010395048	0.097505327
WGC108907D	1.59E-66	0.042004997	0.101762415	0	0.024550066	0.131848094	0.117705675	8.52E-48	0.350317522	0	1.37E-46	0.231811231
WGC108909D	6.43E-50	0.004698324	0.037700061	0	0	0.229715314	0.231827061	0.081778061	0.389417981	0	0.024863198	4.73E-148
WGC108911	0	0.097921116	0.066243101	0.00E+00	0	0.029782397	1.39E-228	2.29E-27	0.370669872	0	0.133787602	0.301595913
WGC108913	4.70E-07	0.011898906	0.040810236	0	0.027012064	0.084961325	0.119098085	0.050956244	0.489354289	0	0.016956004	0.158952376
WGC108915	0	0.001807719	1.58E-01	0.097366781	0.125471783	0.087450108	0.008415473	0.249418443	0.237692761	0.034054564	0	0
WGC108917	0	0	0.188718608	0.096320633	0.004050517	0.065268758	0.084516406	0.170049679	0.382491248	0	0	0.00858415
WGC108919	0.022214795	0	0.033629693	0	0	0.159259045	0.208749974	0	0.267382462	0.093066303	0.107249096	0.108448631
WGC108921	0.177976936	0	0.198293877	0	0.022003374	0.106583199	0.097056139	0.139361102	0.177869752	0	0.01628118	0.06457444
WGC109742	0.000235557	0	3.69E-62	0.062149177	0.042528657	0.069451395	0.020915496	0.116991051	0.639900368	0.000228525	0	0.047599774
WGC109744	0.179893832	0	0	0.005265528	5.68E-02	0.148828078	0.033883436	0.199051974	0.210375907	0.03200373	0.01188621	0.122033672
WGC109746	2.18E-23	2.04E-19	0.093440965	0	0.028830119	0.041886056	0.047678941	0.149904029	0.218815136	0	0.247074578	0.172370175
WGC109748	0.149561448	0	0.05766684	0	0	0.033715745	0.023493183	0.008107954	0.452987625	0	0.1840392	0.090428004

WGC109750	0.125534747	0	0.083492765	0	0.069983732	0.04447811	0.115221511	0.218630503	0.314641931	0.028016701	0	0
WGC109752	0.000124637	0.089386981	0.054370051	0.031676388	0	0.338712468	0.161019728	0.161299536	0.163410211	0	0	2.02E-24
WGC109754	4.52E-73	0.042377496	0.032956887	0.066599234	0	0.043045818	0.100751121	0.163053399	0.454686885	0.0354191	0	0.06111006
WGC109756	0	0	0.136473793	0	0	0.099380913	0.107586015	0.239671935	0.383328327	0.01825613	8.32E-38	0.015302888
WGC109758	0.009237399	0	0	0	0.152611478	0	0	0.539329794	0.25512261	0	0	0.043698719
WGC109760	0	0.031703165	8.78E-19	0.036132365	0	0.408045687	0.379872754	2.77E-29	0.144246029	7.91E-80	0	6.17E-103
WGC109762	7.81E-38	0	0.06025211	0.050013396	0.031635342	0.095396573	0.056062327	0.090819492	0.526526282	0	0.003342072	0.085952407
WGC109764	0.135745736	0.08852816	0.027093911	0.339662769	1.42E-41	0.040273813	0.09137537	0.05083945	0.139945223	0.013357802	0	0.073177767
WGC109770	0.03265023	0.11510658	0.005214842	0	0.009497374	0.222033316	0.274459067	0.028102701	0.239931953	0.008002273	0.024373766	0.040627898
WGC109774	0.061855291	0.042608609	0.08894296	1.36E-28	0.025112329	0.089718678	0.05120454	0.127825534	0.406928389	0	0.023482716	0.082320955
WGC109776	7.46E-50	0	0.150745429	0.021639138	0.066699221	0.068640806	0.094174834	5.12E-15	0.438633696	5.48E-09	0.056439507	0.103027364
WGC109778	0	9.35E-65	0	9.18E-20	0	0	0.112597922	0.4219365	0.465465578	0	0	0
WGC109780	0	0	1.99E-07	0.064206018	0.022534146	0.100540209	0.137294743	0.030582588	0.543174761	1.02E-53	0.036647344	0.065019992
WGC109782	0	0	0.126001505	0.02456383	0.008037208	0.22026704	0.154142454	0.107696256	0.252408213	0.004994075	0.001913686	0.099975734
WGC109784	0.231514217	0	0.058687808	0.006786854	0.033622489	0.060255131	0.006778893	0.196141044	0.247452637	0	0.009972978	0.148787949
WGC109786	0.130477578	0	0.070171	0.063638495	0.05167805	0.069896393	0.072629121	0.037146429	0.238759702	0	0.032240979	0.233362251
WGC109788	0	0.022951456	3.17E-121	0	0.005583708	0.298996741	0.102163909	0.224873839	0.286394165	0	0.0256013	0.033434882
WGC109790	6.72E-29	0.024384121	0.172248244	0	0.001578218	0.102150908	0.10095889	0.138127491	0.349871946	0.012016346	0.034733652	0.063930185
WGC109792	0	0.084801058	0.012878156	0	0.000312908	0.302936771	0.291016947	7.09E-78	0.273102428	0	9.98E-97	0.034951733
WGC109794	0	0	1.56E-01	1.58E-48	0.012531166	0.014436234	0.079482689	1.98E-08	0.542695388	0	2.10E-169	0.195270708
WGC109796	1.77E-50	0.005240042	0.005467117	0.000673691	0.018765348	0.28560193	0.197266143	0.018670399	0.387002519	0	1.79E-44	0.081312811
WGC109798	0	0	0.021763395	0.037368533	0.002752488	0.169437505	0.131540478	0.341342181	0.276333434	0	0.019461986	4.78E-37
WGC109800	0	0.044792162	0.179837347	0.331456727	0.013253944	0.010749276	0.026085685	0.204440989	0.098401963	0.008878413	0	0.082103493
WGC109802	0	0.065125211	0.105906826	0.055609166	0	0.107129856	0.144321778	0.141945239	0.349841915	0.030120009	0	3.73E-152
WGC109804	1.43E-74	0.102563345	0.046681122	0.094747535	0	0.028417947	0.10766176	0.024138606	0.258083231	0	0.123944203	0.213762251
WGC109806	0	0	0.19968876	5.15E-48	0.028275436	0.054394037	0.074737599	0.175370954	0.422498608	0.008942594	9.09E-26	0.036092012
WGC109808	0.054467187	0.02926812	3.54E-34	0.093430224	0.018680611	0.170050081	0.164260034	0.144606448	0.288204712	0	0.037032584	1.46E-32
WGC109810	0	1.40E-73	0.107968567	0	0.016244384	0.30229934	0.302914213	0.08989757	0.156769444	0	0.023906482	0
WGC109812	2.79E-36	0.001736468	0.002782463	2.01E-74	0.0159722	0.138024376	0.253838272	0.097708849	0.424732216	5.99E-138	0	0.065205157

WGC109814	0.189508964	0	0.071324811	0.009132568	0	0.073018248	0.072654424	0.132007868	0.256723415	0	0.011995966	0.183633736
WGC109816	2.82E-51	0.092558377	0.070063002	0.022960645	0	0.210932271	0.31619144	1.81E-47	0.28729422	0	4.49E-08	1.24E-131
WGC109818	0	0	1.43E-32	0.013364034	0.012878824	0.297817709	0.353606023	0.079669974	0.235072106	0	0.00759133	4.05E-157
WGC109820	2.81E-21	0.167160088	0.039522759	0	0	0.105673968	0.210038314	0.182862724	0.210535949	0	0.084206197	0
WGC109824	0	0	5.92E-147	0	0.028425463	0.174668394	0.119677265	0.372340345	8.36E-02	0	0.11506183	0.106219358
WGC109828	0.155806543	0	0.037983704	0	0.036630563	0.005345228	0.067127411	0.19793057	0.485019998	0.0059829	0.008173083	0
WGC109830	4.05E-20	0.069446613	0.036413237	0	0	0.248823296	0.235774059	0.099289939	0.142670571	0.064489153	0.045443342	0.05764979
WGC109832	0.079347144	0.026491701	0.10688031	0	0.034830124	0.106695491	0.089465378	0.136129113	0.268468302	3.02E-36	0.000188648	0.151503789
WGC109836	0.013350533	0	0.168376253	0.02510788	0.023493376	0.075230103	0.102185403	0.149487038	0.417051169	0	0.025718245	0
WGC109840	0.004265993	0	0.006980175	0.003176399	0.012497235	0.101861232	0.15920579	0.015900103	0.419470955	0.020172207	0.01868533	0.237784582
WGC109842	0	0.114522796	0.028199911	0	0.035810201	0.005324377	0.08795375	0.203892933	0.142167273	0	0.088892171	0.293236588
WGC109844	0.238841575	0	0.088048472	0	0.013391141	0.098157257	0	0.191978059	0.342846453	0.026737042	7.12E-19	0
WGC109846	0	0.069934459	0.075319344	0	0.011822596	0	0.001055941	0.097884562	0.606811143	1.92E-21	0	0.137171955
WGC109848	0.036093974	0	0.081787963	0	0	0.144016274	0.222884636	0.178728978	0.329497078	0	0.006991096	6.05E-11
WGC109850	0.010410677	0	0.043125514	0.083976509	0.018672376	0.102422667	0.312108094	0.337550982	0	0	0.024965124	0.066768057
WGC109852	0	0	1.25E-148	0	0	0.039991871	0.166613263	0.178988396	0.500598373	0	0	0.113808098
WGC109854	0	1.91E-11	0.050162277	0	0.005681424	0.014935864	0.086528748	0.024320045	0.223813502	0	0.577206756	0.017351384
WGC109856	0.036421059	0.000634709	0.197667172	0.040975278	0.013800133	0.061656168	0.130022185	0.20497841	0.277694933	6.61E-14	0	0.036149953
WGC109858	0.054915379	0.002013382	0.038526379	0.01709125	1.68E-28	0.365940787	0.337171446	0.040683469	0.12872427	8.58E-36	3.19E-78	0.014933639
WGC110899	5.36E-10	0	0.151361593	0	0.015180729	0.094177439	0.313097936	0.145224278	0.26328159	0	0	0.017676435
WGC110903	0	0.079629676	0.031568831	0	0.028702298	0.054920584	0.038252235	0.306725082	0.308914559	0	0.005625511	0.145661224
WGC110907	0	0	4.24E-132	0	0.031687932	0.127703335	0.189414628	0.238269042	0.270832079	0.057435128	0	0.084657858
WGC110909	1.48E-94	0	1.50E-15	0.07234828	0	0.060925069	0.118959251	0.244403122	0.162319239	0.188206176	0	0.152838863
WGC110911	0	0	1.59E-106	0.193252419	0.030230199	0	0	0.324923753	0	0.171415163	0	0.280178466
WGC110915	0	0.054645583	2.09E-104	0.046784271	0	0.184842187	0.073164995	0.141408602	0.404646432	0	0.019072835	0.075435097
WGC110917	0	6.31E-212	0	3.95252516672997e-322	2.15E-172	0.070613601	0.074830712	0	0.104440288	3.34E-189	0	0.750115399
WGC110919	0	0	1.44E-84	1.03E-15	0	0.166905585	0.363970323	0.027811039	0.346653057	0.003393172	0	0.091266823
WGC110921D	0.017044283	0.070813405	0	0.004703825	0.011793491	0.139610856	0.068237329	0.081408728	0.291888741	0.038420523	0.060300576	0.215778243

WGC110923D	8.22E-05	0.023066837	0.00508308	0	0.009121201	0.433604215	0.282206531	0	0.206968693	5.42E-84	2.68E-16	0.039867208
WGC110925D	0.058859336	0.030128216	0.108575248	0.010907555	0.012170128	0.116381662	0.094432611	1.42E-16	0.329840269	0.062625844	8.96E-05	0.175989525
WGC110927D	4.74E-121	8.27E-114	1.89E-240	3.86E-31	0.150494087	0.050920615	0.166138586	1.15E-71	0.401388927	0	0	0.231057785
WGC110929D	0	0.12526484	4.33E-41	0.020829272	0.014448435	0.044218955	0.11188458	0.251320889	0.332156806	0	0	0.099876224
WGC110933D	0	0.059198972	0.042586263	0	0.006812932	0.04676482	0.00088104	0.393058355	0.346104661	0	0.072206903	0.032386054
WGC110935D	1.43E-40	0.075110066	0.062590451	0.025368005	0.024928919	0.162440354	0.204612262	1.90E-06	0.199265573	9.78E-87	5.88E-148	0.245682471
WGC110937D	0	0	0.093267052	0.03083302	0.032572113	0	0.086604645	0.426214137	0.330509032	0	0	2.27E-21
WGC110939D	0	0	0.1404331	0.093827543	0	0.086325552	0	0.265508845	0.293728518	0.086671502	1.91E-36	0.03350494
WGC110941D	5.38E-25	0.011892672	0.070075243	3.27E-05	0	0.061355487	0.140594357	0.061201685	0.348204275	0.014569009	0	0.292074604
WGC110943D	2.03E-88	0.096055723	0.027956587	0.017331032	0.137463354	0.001394435	0.039413738	0.093810909	0.320466935	0.014786319	0	0.251320968
WGC110945D	0	0.02840268	4.26E-164	0.024238712	0	0.202518536	0.271275207	0.02380052	0.449764345	0	0	2.94E-308
WGC110947D	0	0	0.045238327	0.053387468	0.017569931	0.214088475	0.429450663	0.024752698	0.083774312	0.046681201	0.019815426	0.065241499
WGC110949D	1.40E-79	0	0.057050683	0.0898358	0.002682881	0.034676789	0.045917628	0.290877584	0.317754399	0.009156528	0.065671153	0.086376555
WGC110951D	0	0	0	3.804305472<br>9776e-322	0.006480217	0.023722818	0.020041866	0	0.32621552	1.82E-232	0	0.623539578
WGC110953D	2.74E-22	0.105358737	0.040477817	0	0.085643177	0.048290756	0.094749245	0.146409776	0.479070492	0	1.75E-29	0
WGC110955D	0.023810209	0.003059524	0.071687776	0.001539063	0.024615278	0.325744061	0.211944019	0.162555204	0.116233492	0.002825803	0.005129773	0.050855798
WGC110957D	0	0.061288397	0.114063907	9.27E-20	0.040337026	0.266419365	0.120772604	0.010487803	0.350525789	0	0	0.036105109
WGC110961D	0.219954272	0	0.04850057	0	0.00442562	0.107141392	0.0295574	0.040415886	0.427269985	1.31E-13	0.028272978	0.094461896
WGC110963D	0.037071062	1.38E-71	0.273441915	3.97E-169	0	0.083785414	0.156197252	0.035563317	0.365161601	0	0.04877944	7.22E-71
WGC110965D	7.71E-46	0	0.074102932	0.041038508	0.004261818	0.033067168	0.052791989	0.056664923	0.413578307	0	1.05E-128	0.324494356
WGC110967D	1.54E-105	0	1.79E-60	0	0.003397321	0.116985349	0.110318021	1.93E-66	0.619812104	2.54E-41	1.08E-283	0.149487205
WGC110969D	0	0	0.436020823	0	0	0.135919272	0	0.237766121	0	0.022080976	0.067176206	0.101036603
WGC110973D	0	0	0.232846011	0.081493008	0.014064498	0.026241871	0.05697373	0.304832324	0.222150588	0	0	0.061397971
WGC110975D	0	0	0.021201495	0	0.024180001	0.141818765	0.093928991	0.224421265	0.476456651	0.017992832	6.71E-18	4.58E-278
WGC110977D	0	0	0.07623997	9.09E-34	0	0.16494007	0.237828432	0.164557251	0.356434278	0	0	0
WGC110979D	0.033529194	0	0.096460553	0	0.054603897	0.05353534	0.062047997	0.103681333	0.420341354	0.01213518	0.018175645	0.145489506
WGC110981D	3.84E-80	0	0.039576715	0	0.047271243	0.049385231	0.070682585	0.608099095	0.114034802	0	0	0.07095033
WGC110983D	1.81E-27	0	0.035196776	0.090788631	0.034097402	0.200825575	0.170980262	0.085935624	0.34671127	0	3.24E-162	0.035464459

WGC110985D	0	0	0	0.016737132	0.004538033	0.21086511	0.196957389	0.11658876	0.454313575	0	0	6.37E-20
WGC110987D	0.126755756	4.57E-14	0	0	0.028946102	0.269847566	0.122032994	0.010499387	0.362249786	0.010579881	1.62E-24	0.069088528
WGC110989D	0.013063288	2.48E-39	5.43E-61	0.001215343	9.69E-18	0.208894129	0.089422882	0.308214943	0.207569573	0.057127116	0.056850967	0.057641758
WGC110991D	0	0	0.169417553	0	0.003934651	0.076073788	0.15227537	6.61E-09	0.564784521	0.033514111	2.42E-68	3.38E-121
WGC110997D	0	0	0.129081013	0.074057863	0.123369272	0.07380693	0.063936593	0.18920432	0.306241029	1.77E-67	0.030362868	0.009940113
WGC111001D	0	0.019505419	0.045955966	0.064073629	0	0.159486326	0.269405052	0.072752848	0.239223184	0	0	0.129597575
WGC111003D	0	0	0.056722539	0.007579983	0.000537751	0.446406978	0.349677846	3.80E-15	0.087399706	0.051663834	1.14E-05	0
WGC111005D	3.44E-25	0.014829894	0.024706026	0.044277702	0.019617057	0.091744819	0.070814727	0.178294841	0.51940425	9.47E-32	0	0.036310684
WGC111007D	0.119270054	0	0.110056571	0.069974616	0.031666208	0.163708367	0.123954673	0.171948201	0.09313474	0	0.034929049	0.081357522
WGC111009D	3.33E-08	0	0	0.136075837	0	0.085751656	0.13043283	0.25791879	0.258456551	0.024540891	0.055007632	0.051815779
WGC111011D	2.37E-19	0.164766723	0.010681346	0.069931463	0	0.084463112	0.187672041	0.077421274	0.368275667	0.030562912	0	0.006225462
WGC111013D	3.22E-51	0	0.161147493	0	0.028854514	0.033880817	0.007223328	0.115482145	0.511704407	0.018635312	0.001505948	0.121566036
WGC111015D	0	0.016499554	0.190744747	0.199138478	3.58E-14	0.063876179	2.33E-190	0.362145166	0.167595875	0	0	0
WGC111825D	0.093602957	0	0.148789483	0.065091169	0	0.008467892	0.011284838	0.119184792	0.395057235	0	0	0.158521634
WGC111827D	0	0.016838461	0.123867421	0.013367326	0	0.182974081	0.23932239	0.182289467	0.231056911	2.45E-49	0.010283944	0
WGC111833D	5.76E-52	0	0.177699356	0.090784325	0	0.134657593	0.035809725	0.075269522	0.468534506	4.40E-120	0	0.017244972
WGC111835D	0.197309115	0	0.035014448	0.007865096	0.02599183	0.166586568	0.027545802	0.258677883	0.068270915	0.056538828	0.056020592	0.100178925
WGC111839D	0	0.072201536	0.069754523	0.120566784	0.013687585	0.0501481	0.077356701	0.224714707	0.247545718	5.31E-53	0.059358115	0.064666232
WGC111841D	2.35E-54	0	0.035410731	0.048991554	0.03757956	0.137305633	0.192641856	0.099512615	0.447983207	0.000574845	0	1.13E-148
WGC111843D	1.72E-78	0	0	0.039568395	0.006015483	0.173471113	0.077290997	0.399130874	0.155382246	0	0.049154756	0.099986135
WGC111845D	0.077251103	0	0.068445938	1.01E-28	0.016785983	0.155181768	0.076597776	0.336472535	0.192043337	0.046958387	0.00015279	0.030110382
WGC111853D	0.087514811	0.036240887	2.84E-35	0.001122612	0.076461651	0.031625448	0.110365864	0.233049788	0.35278046	0.070838479	0	1.16E-115
WGC111857D	0	0	0.361237639	0.001153129	0.018292945	0.045087083	0.105080775	0.041395233	0.299362915	0.004776169	0	0.123614112
WGC111859D	0.029502607	0	2.89E-23	0	0.009659951	0.02204101	0.180500145	1.25E-07	0.421097165	0.006689966	0	0.33050903
WGC111861D	0.001461255	0.008317136	0.082748059	0	0.037424346	0.041427044	0.04223094	0.110232754	0.50020799	3.89E-22	4.34E-59	0.175950476
WGC111867D	0.101232789	0	9.62E-47	1.64E-23	4.67E-69	0.048167938	0.032257828	0.198015389	0.620326055	0	5.05E-84	8.25E-138
WGC111869D	0.130420168	0.091122893	0.091151203	0.048339555	0.008422526	0.155081418	0.090412038	0.078928434	0.175881323	0	7.23E-82	0.13024044
WGC111871D	0	0.044968175	0.090821188	0	0.020914375	0.080419746	0.078740746	0.159643246	0.437975589	0	0.062638219	0.023878717
WGC111873D	0.152067796	0	0.113813282	0	0.07608434	0.042722906	0	0.163272971	0.36269032	0.042226538	1.07E-116	0.047121847

WGC111881D	0	0	0	0.09568738	0.087454151	0	0.00269828	0.651965219	0.111550008	0.023533823	0	0.027111139
WGC111883D	0	0	0.184782571	0.049462794	0.033474475	0.007476405	0.109172241	9.92E-29	0.288451393	0	0.037446377	0.289733743
WGC111885D	0	0	0.240191692	0.16809302	0.219658956	0	0.057501698	0.314554634	0	0	0	0
WGC111887D	0	0.133640945	0.010216786	0	0.043454405	0.160275601	0.066814466	0.114848835	0.336049979	0	0.040498755	0.094200229
WGC111889D	0	0.021921512	0	0.005336553	0	0.371846924	0.145236317	0.144036198	0.274592001	0.010960877	0	0.026069618
WGC111891D	0	0	0.05166795	0	0.00204162	0.196338236	0.177897607	0.203085462	0.34636018	0	0.021887699	0.000721246
WGC111895D	1.25E-56	0	0.227657588	0.005242036	0	0.084428068	0.077688993	0.084178144	0.350602212	0.033769057	0.005436865	0.130997038
WGC111897D	2.64E-53	0	0.041925136	0.016845311	0	0.092754487	0.145046831	0.091711707	0.47169571	0	0	0.140020818
WGC111899D	0	0	0.011351121	0	0.039396401	0.170265613	0.315914937	0.038362806	0.252047123	0	0.075634812	0.097027189
WGC111901D	0.147180582	0.004375461	0.092848292	0	0	0.016045195	0.042982403	0.07688924	0.229206314	0.0219107	0.049778922	0.31878289
WGC111909D	7.28E-07	1.68E-06	0.037321358	0	0.010222784	0.471680978	0.339882742	0	0.116838703	0.007407868	0	0.016643164
WGC111911D	0	0.221000868	0	2.11E-24	0.041610317	6.51E-103	0.065395614	0.234926296	0.247478941	0.059024422	0	0.130563542
WGC111917D	0.272978662	0.021669604	0.075774535	0	0	0.174992883	0.101064796	0.171056921	0.114610656	0	0.024144081	0.043707863
WGC111919D	0	0.062627802	0.014299552	1.66E-38	0.021744387	0.055055827	0.119323493	4.71E-17	0.598198898	0.019348595	0	0.109401446
WGC111921D	0	0.059742809	0.090424343	0	0.034674991	0.006465562	0.046178821	0.234609478	0.37916011	0	0.091448498	0.057295389
WGC111923D	0.277238384	3.13E-21	0.126197495	0.093951869	0	0.092793566	0.094385586	0.175393537	0.096662912	0.043376651	0	0
WGC111925D	3.58E-30	0.000745697	0.018563352	1.16E-23	0.10265509	0.176247414	0.192522877	0.151460267	0.33384702	0	0	0.023958283
WGC111927D	1.98E-50	9.33E-64	0.044987718	0	0	0.049457189	0.076757884	0.069977461	0.56435504	0	3.58E-142	0.194464709
WGC111929D	0.077793456	0.050837329	0.034663396	0.003768087	1.63E-08	0.075129481	0.051172936	0.174765853	0.337753958	0	0.016988142	0.177127348
WGC111931D	0.124004164	0.041040198	0.058056271	0	0.009771573	0.016549765	0.083767706	0.193384509	0.388342736	0	1.99E-111	0.085083078
WGC111933D	0.053849513	0.04092828	0.065101337	0.034487054	0.060895603	0.093421681	0.054221177	0.09087666	0.48732075	0	0	0.018897944
WGC111935D	2.88E-66	0	7.61E-61	0.015389302	0.042602022	0.366136608	0.267774217	0.055183064	0.213805412	0.015458207	0.023650794	3.74E-07
WGC111937D	0.033133223	0	0.217923909	0	0.050953768	0.020258707	0.013061681	0.454463235	0.109067003	0	0.02834377	0.072794706
WGC111939D	8.51E-09	0	0.057403916	0.075589023	0.105162734	0.013126603	0.104474158	0.158080363	0.217075324	0	0	0.269087872
WGC111941D	1.61E-82	0	0.119586103	0.00732316	0	0.008747588	0.086835829	0.167302451	0.397927306	0.02192418	0	0.190353383
AJHG2015-1N01-VS-1T01	0	0	0.087125681	0.022767236	0.021916173	0.182982017	0.198752502	0.088126671	0.394004995	0.004324727	0	0
AJHG2015-1N02-VS-1T02	0.067052496	3.23E-66	0.082997893	0	0.039016947	0.156949682	0.185234758	1.79E-45	0.468748224	0	0	1.22E-155

AJHG2015-1N03-VS-1T03	0.065901328	0.102960175	0.02694791	0	0.031352733	0.081218459	0.106273208	0.246766337	0.328393678	0	0.010186173	0
AJHG2015-1N04-VS-1T04	2.33E-130	1.65E-14	0.239240726	0	0	0.047895402	0	9.18E-87	0.459045071	0	6.89E-45	0.253818801
AJHG2015-1N05-VS-1T05	5.87E-96	0.009428797	0.122152742	0.026667726	0.018411314	0.11642032	0.181468955	0.075745422	0.332116723	0	4.83E-82	0.117588002
AJHG2015-1N21-VS-1T21	0	0.033880844	0.098793294	0.062268969	0.041896842	0.057792552	0.068204986	0.277617119	0.270174252	0	1.77E-50	0.089371143
AJHG2015-1N22-VS-1T22	0	0	0.110518319	0.00798979	0.064665002	0.049243483	0.180310004	0.021528276	0.43219509	1.59E-60	0	0.133550035
AJHG2015-1N23-VS-1T23	0	3.07E-129	0.209000855	0	0.018338417	0.07369028	0.189070204	5.71E-123	0.30801432	0.029212733	2.29E-269	0.17267319
AJHG2015-1N24-VS-1T24	9.01E-05	0.059416025	1.08E-40	0.104107051	0	0.242704997	0.102710462	0.116372917	0.211817423	0	4.40E-84	0.162780992
AJHG2015-1N25-VS-1T25	0	0	0.108175537	0	0.055896914	0.01527676	0.052120226	0.134267477	0.38058286	0.027075936	0.037474987	0.189129303
AJHG2015-1N26-VS-1T26	0.012043747	0.196573626	0.08311659	0	0.014112254	0.091212709	0.063368599	0.199257807	0.164422671	0	0.093426113	0.082465884
AJHG2015-1N27-VS-1T27	0	0	0.003697559	8.37E-44	0.018325303	0.08444258	0.133301285	0	0.418553348	0	0.02745486	0.314225065
AJHG2015-1N28-VS-1T28	0	0	0.056317365	0	0.002384573	0.212911791	0.089920333	0.05819199	0.561422445	0.018851503	0	0
AJHG2015-1N29-VS-1T29	1.47E-28	0.054280866	0.306203691	0.017333422	0.023724063	0.032081383	0.210779544	0.112814214	0.171239166	0.023985989	0.013643144	0.033914519
AJHG2015-1N30-VS-1T30	0	0	0.049770068	3.17E-71	0	0.196130526	0.172473686	0.193498118	0.381837239	0	0.006290362	5.70E-58
AJHG2015-1N31-VS-1T31	0	0	2.55E-59	0.02960656	0.00334684	0.264763281	0.283236188	0.049453394	0.296211879	0.039199999	0	0.03418186
AJHG2015-1N32-VS-1T32	5.16E-70	0.027600758	0.063354238	0	0	0.047702476	0.202990184	0.035429278	0.537141463	0	0.00841725	0.077364353

AJHG2015-1N33-VS-1T33	0	0	1.35E-53	0	0.058455348	0.122057266	0.060793218	0.197341329	0.370824965	0.006714799	0.064069959	0.119743117
AJHG2015-1N34-VS-1T34	0.121824382	0	0.08515108	0	0.014719209	0.120530837	0.101711305	0.283948702	0.163382769	0	0.035920209	0.072811507
AJHG2015-1N35-VS-1T35	0	0.084445549	0.407402967	0	0	0.020450471	0.093088808	2.12E-102	0.241669564	0.000714274	0	0.152228368
AJHG2015-1N36-VS-1T36	9.26E-25	0	0.07879755	0.015924836	0.02429638	0.165607188	0.064336737	0.039142503	0.529180352	0	0	0.082714454
AJHG2015-1N37-VS-1T37	0.292746849	0.070989098	0.012658419	0	0.011111733	0.182402172	0.086086457	0.056969113	0.162982199	0.024088721	0	0.09996524
AJHG2015-1N38-VS-1T38	0.185770223	0	0.100127041	0	0.019502269	0.1588971	0.086519628	0.083968192	0.264432265	0.004687482	0.035175282	0.060920518
AJHG2015-1N39-VS-1T39	1.54E-49	0	0.119851277	0	0	0.094749991	0.055221654	0.236019716	0.490611621	0.003545741	0	2.19E-130
AJHG2015-1N40-VS-1T40	0	0.018716672	0.034649684	0	0	0.294997391	0.256395195	0.089324509	0.245108802	0.013894972	0.011239066	0.035673709
AJHG2015-1N41-VS-1T41	6.87E-77	0	0.076292238	0.017742528	0.077796469	0.098579641	0.07821915	0.082226076	0.415806489	0	0	0.153337409
AJHG2015-1N42-VS-1T42	0	0.015875742	0	3.89E-56	0.041739054	0.065206507	0.065610032	0.473433452	0.301458005	0	0	0.036677208
AJHG2015-1N43-VS-1T43	0.021904055	0.029040512	0.031454728	0	0	0.093908343	0.118067623	0.423780663	0.220739184	0	0.061104892	3.27E-148
AJHG2015-1N44-VS-1T44	0.094934902	0.075178462	0.008926976	0.042935655	0	0	0.022222204	0.290630107	0.449545022	0	0	0.015626672
AJHG2015-1N45-VS-1T45	0.021184166	0	0.037235421	0.054026411	0	0.239391321	0.237577754	0.144651022	0.265933905	0	0	6.07E-100
AJHG2015-1N46-VS-1T46	1.01E-46	0.036742917	0.208852021	0.004978627	0	0.164376235	0.130640512	4.30E-40	0.287160319	8.66E-46	0.014805754	0.152443615
AJHG2015-1N47-VS-1T47	0	0	1.51E-17	0.00491021	0.007360876	0.071615685	0.133403494	0.155932454	0.543411064	0	0	0.083366216

AJHG2015-1N48-VS-1T48	0	0	0.094040418	0.079973355	0	0.184339918	0.142401922	0.123449523	0.24453135	4.46E-19	0	0.131263515
AJHG2015-1N49-VS-1T49	6.90E-65	0.127081373	0.002972808	0.00075965	0	0.044803043	0.120913356	1.78E-44	0.433443841	0.005572464	0	0.264453465
AJHG2015-1N50-VS-1T50	6.60E-79	0.043358236	0	0.077157645	0.015561294	0.006391449	0.045594792	0.143410997	0.543929089	0.011816367	0.033050047	0.079730084
AJHG2015-1N51-VS-1T51	0.016906362	0.005924941	0.208680982	0	0.007931987	0.042683127	0.053616853	0.007414628	0.3139074	0.066376722	0.056591332	0.219965667
AJHG2015-1N52-VS-1T52	0	0	0.189102561	0	0.022352672	0.030288084	0.072013554	0.109127646	0.446831209	0	0.018690743	0.111593532
AJHG2015-1N53-VS-1T53	2.74E-09	0.080363892	0.111628612	0.078201648	7.17E-09	0.148504082	0.079045251	0.026604662	0.43537404	0	0	0.040277803
AJHG2015-1N54-VS-1T54	0	0.091302494	0.022436025	0.085767129	0.037938273	0.055089898	0.027264544	0.235949323	0.40613354	0	8.54E-82	0.038118775
AJHG2015-1N55-VS-1T55	0	1.83E-05	0.098929072	0	0	0.116561354	0.184476066	0.239980158	0.334137	0.025898054	0	9.28E-182
AJHG2015-1N56-VS-1T56	0.08284278	0	0.056046515	0	0.024652428	0.082158225	0.146172498	0.129717444	0.259501216	0	0.113478373	0.10543052
AJHG2015-1N57-VS-1T57	7.87E-69	0.043591666	0.050471625	0.125993247	0.021088871	0.102213745	0.078259516	0.035359226	0.371036539	0.01012385	1.65E-290	0.161861716
AJHG2015-1N58-VS-1T58	0	0	0.316612599	0	0	0.030424795	0.123261474	0.155016263	0.250745205	0.095393072	2.14E-64	0.028546592
AJHG2015-1N59-VS-1T59	0	0	0	1.89E-25	0	0.300188033	0.149959992	0.208797887	0.224580358	0.104960757	0.011512975	1.66E-252
AJHG2015-1N60-VS-1T60	2.03E-28	0.100665781	0.050257456	0	0	0.037190252	0.150756008	0.110360721	0.460644305	0.000493199	0.043256706	0.046375573
AJHG2015-1N61-VS-1T61	0	2.36E-28	0.285402929	0.011536763	0.024879294	0	0.042752293	0.064250155	0.571177881	0	0	6.85E-07
AJHG2015-1N62-VS-1T62	0	0	0.037285529	0.051498086	0.003827335	0.298993282	0.188016308	0.063200708	0.3020585	0	0.02184438	0.033275872

AJHG2015-1N63-VS-1T63	0	0	1.06E-65	0	0.028218238	0.049553264	0.327834455	0.009448097	0.352249277	0	6.46E-298	0.232696669
AJHG2015-1N64-VS-1T64	0.093265422	0.026803207	0.123910287	0	0.065997258	0.063696743	0.102011812	1.74E-32	0.394965385	0.018752039	0	0.110597848
AJHG2015-1N65-VS-1T65	0	0	0.137356796	0	0.033880915	0.081502253	0.075621771	0.133815327	0.340467249	0.031443235	0.003137007	0.162775446
AJHG2015-1N66-VS-1T66	0	0	0.070401615	8.51E-07	0.024905497	0.107887918	0.212705324	9.55E-07	0.466279181	0	0	0.117818659
AJHG2015-3N01-VS-3T01	0.127863829	0	0.072235678	0	0	0.108284534	0.05328646	0.210287193	0.393225476	0.031109684	0.003707145	1.89E-24
AJHG2015-3N02-VS-3T02	8.31E-12	0.015167592	0.155602164	0	0.026188409	0.113208997	0.054086021	0.183879491	0.430838726	0.0210286	1.40E-76	4.83E-125
AJHG2015-3N03-VS-3T03	2.61E-51	0.049468311	0.088066926	0	0	0.05925632	0.07488388	0.122764742	0.460596676	0	0.023662848	0.121300297
AJHG2015-3N04-VS-3T04	0.142339625	0.004647152	0	0	0.004374842	0.047337956	0.153439691	0.101841932	0.439216699	0	0.004532329	0.102269773
AJHG2015-3N05-VS-3T05	3.09E-46	0	0.161482442	0.040607836	0.068901377	0.106581576	0.09997875	0.067099179	0.45534884	9.31E-74	8.57E-65	5.61E-134
AJHG2015-3N06-VS-3T06	8.62E-05	0.054928164	0.14829824	0	0	0.050282763	0.036293879	0.236398357	0.068981761	0	0.273927182	0.130803502
AJHG2015-3N07-VS-3T07	0	0.111071953	0.12072226	1.35E-63	0.041859124	0.039780598	0.007022652	0.038544477	0.626945634	0	0.014043172	1.01E-05
AJHG2015-3N08-VS-3T08	0	0.021382702	0	0	0.045355425	0.218288282	0.117266747	0.235667264	0.297594714	0	0.004528488	0.059916378
AJHG2015-3N09-VS-3T09	1.27E-92	0	0.045053531	0	0.034923619	0.23378123	0.139450439	8.97E-05	0.371876466	0.036226787	1.85E-20	0.13859822
AJHG2015-3N21-VS-3T21	0	0	6.04E-20	0	0.039300016	0.143174134	0.029469033	0.35086947	0.330534151	0.021356182	0.014566542	0.070730472
AJHG2015-3N22-VS-3T22	0	0.02793826	0.184226177	0.050391746	0.010124251	0.046329464	0.062353512	0.350807322	0.234227962	0	0	0.033601305

AJHG2015-3N23-VS-3T23	0.010520973	0	0.091993411	0	0.05655846	0.017933114	0.092689785	0.290233013	0.295993443	0	0.06680556	0.077272241
AJHG2015-3N24-VS-3T24	0	0.081063802	0.069108328	0.044048681	0.020572619	0.269085351	0.21578152	0.116991758	0.183347941	9.78E-39	8.69E-172	3.12E-175
AJHG2015-3N25-VS-3T25	0	0.175812235	0.002040175	0.003623566	0	0.123741029	0.177045113	0.171942792	0.33638426	0	0	0.00941083
AJHG2015-3N26-VS-3T26	6.31E-07	0	0.104601421	0	0.000638585	0.105657122	0.107113495	0.227464459	0.397578556	0	0.04596394	0.010981791
AJHG2015-3N27-VS-3T27	0	0	0.150710349	0	0	0.076060661	0.119915065	0.102265643	0.551048282	0	0	0
AJHG2015-3N28-VS-3T28	0	0	0.099584593	3.39E-31	0.078123073	9.80E-102	0.042694962	0.065567291	0.690230163	0.007669289	0	0.01613063
AJHG2015-3N29-VS-3T29	0.10718261	0.142755375	4.65E-25	0.051568373	0	0.031021436	0.039337362	0.285871558	0.279866697	0.002230867	0	0.060165722
AJHG2015-3N30-VS-3T30	0.085684589	0.002474647	0	0.003644261	0	0.013513034	0.053871669	0	0.71151956	0	0	0.129292239
AJHG2015-3N31-VS-3T31	5.28E-30	0.069859354	0.117495687	0	0.109775478	0.067187861	0.045307815	6.36E-07	0.486906952	0	0.09282711	0.010639107
AJHG2015-3N32-VS-3T32	0	0	0.038279601	9.40E-17	0	0.129705617	0.144573317	0.191985599	0.416567734	0	0	0.078888131
AJHG2015-3N33-VS-3T33	1.85E-75	4.93E-57	0.004794737	0.104111165	0.006992936	0.085671027	0.128971482	0.110680734	0.490465558	0.014031383	6.78E-103	0.054280978
AJHG2015-3N34-VS-3T34	0.00552407	0.04346177	0.042336185	0	0.001509754	0.043899695	0.007298632	0.353041907	0.14685817	0.01803429	0.021134088	0.316901439
AJHG2015-3N35-VS-3T35	0.066391524	0.050829028	0.006978818	0.0541858	0	0.033571368	0.059773846	0.162221114	0.437470014	0.060233529	0	0.068344957
AJHG2015-3N36-VS-3T36	0	0.027660322	0.179722537	0.00429439	0.019223342	0.034557097	0.174494172	0.167629628	0.392418512	0	0	0
AJHG2015-3N37-VS-3T37	0.032017949	0	0.051524456	0	0	0.102661929	0.101932611	0.368633147	0.26159618	0.004733677	0	0.076900051

AJHG2015-3N38-VS-3T38	0	0	3.61E-58	0	1.07E-21	0.385990778	0.365333455	1.22E-59	0.235048963	0.013626804	0	0
AJHG2015-3N39-VS-3T39	1.12E-35	0.077716254	0.064636098	0.009680977	0	0.01399507	0.069129047	1.88E-60	0.673410892	0	0	0.091431662
AJHG2015-3N40-VS-3T40	0	0	0.0499513	0.006507347	0.020580892	0.121299981	0.223601591	0.050925725	0.334699402	6.95E-31	0	0.192433761
AJHG2015-3N41-VS-3T41	0.064276031	0.024126234	0.031384549	0.09140616	0.057318269	0.071807387	0.099443771	1.17E-21	0.40577863	0	0.022392361	0.132066609
AJHG2015-3N42-VS-3T42	0	0	0.041463286	0.15871424	0	0.182355144	0.160950036	0.056287732	0.368610639	0	0	0.031618923
AJHG2015-3N43-VS-3T43	0	0.062915224	0.006478112	0.095097604	0	0.058161716	0.126591596	0.18679622	0.422572517	0.001570509	0	0.039816502
AJHG2015-3N44-VS-3T44	0	4.58E-24	0.003525708	0.005871235	0.006627008	0.404064415	0.255329093	0.244208185	0.080366723	0	7.63E-06	0
AJHG2015-3N45-VS-3T45	0	0.090044894	0.014554838	0.026940118	0.025735969	0.026629715	0.175551945	0.078379653	0.506149139	0	0	0.056013728
AJHG2015-3N46-VS-3T46	0.049704976	0.195654051	0	0	0.08591359	0.292177003	0.181623118	0	0.194927262	0	0	0
AJHG2015-3N47-VS-3T47	0	0.195037757	0.136250959	0	0	0	0.164999808	0.287147234	0.148545105	0	0	0.068019138
AJHG2015-3N48-VS-3T48	2.14E-126	0.122023821	1.90E-120	7.09E-43	0	0.229228319	0.160952376	1.40E-38	0.487795484	9.23E-150	0	2.89E-68
AJHG2015-3N49-VS-3T49	0.032327632	0	0.149312676	0.018311097	0.026785566	0.117933842	0.140906052	0.055145083	0.092600367	0.017185874	0	0.34949181
AJHG2015-3N50-VS-3T50	0	0.065881832	2.86E-76	0	1.57E-09	0.362467654	0.206136026	0	0.365514487	0	0	1.55E-122
AJHG2015-3N51-VS-3T51	0.023665214	0	0.110221326	0	0	0.279551842	0.166349388	0.171782303	0.196251208	0.002331226	0.002288837	0.047558655
AJHG2015-3N52-VS-3T52	1.55E-16	1.16E-100	0.074194736	4.51E-129	0.009235671	0.38495481	0.226074571	0.023545528	0.251927381	4.33E-62	2.00E-29	0.030067302

AJHG2015-3N53-VS-3T53	0.064759876	0.060469329	0.026105575	0	0.0027347	0.005198882	0.149545231	0.147757433	0.15180266	0.037879295	0.12402904	0.229717977
AJHG2015-3N54-VS-3T54	0.218569063	0	0.058996567	0	0.054397103	0.056653043	7.30E-66	0.058555477	0.52209191	0	0.030736838	0
AJHG2015-3N55-VS-3T55	0.042840471	0.108856607	0.257304109	0	0.009198401	0.055704201	6.76E-83	0.037431435	0.48717217	0.001492606	0	0
AJHG2015-3N56-VS-3T56	0	0	0.066701074	0	0.248739478	0.018644987	0	4.67E-32	0.446504208	0.015329565	0.059972659	0.144108029
AJHG2015-3N57-VS-3T57	0	0.014774471	0.185179805	0	0	0.159641225	0.161975416	0.052145493	0.279974583	0.004652268	0.083788407	0.057868332
AJHG2015-3N58-VS-3T58	0	2.47E-17	0.137465468	0.058259738	0.00841813	0.032482443	0.13966538	0.13980001	0.356126595	0	0	0.127782236
AJHG2015-3N59-VS-3T59	0	3.38E-17	0.162869524	0	0	0.157632924	0.235263307	7.55E-67	0.330294484	0.004115886	0	0.109823875
AJHG2015-3N60-VS-3T60	0.078860442	0.098071885	0.018270968	0	0.002836405	0.031874133	0.124031264	1.79E-45	0.460205377	0	1.24E-52	0.185849527
AJHG2015-3N61-VS-3T61	1.48E-136	0	0.194700113	0.022317256	0	0.101437071	0.044978319	0.0232578	0.200110795	0.330560357	0	0.082638289
AJHG2015-3N62-VS-3T62	0	0.155835721	0.163162611	0	0	0.067140197	0	0.403921706	0.092637983	0.009383957	0.107917824	0
AJHG2015-3N63-VS-3T63	7.32E-58	0.124724301	0	5.44E-65	0.005289406	0.115980904	0.098089259	0.375881109	0.157567378	0.077789414	0	0.044678229
AJHG2015-3N64-VS-3T64	0.139073822	0.036445841	0.048385641	0	0.035205138	0.048506896	0.012569633	0.260861519	0.111573142	0.02609599	0.038774554	0.242507825
Gastroenterology 2016-ESCC_1	0	0	0.197676121	0	0.021265745	0.031369985	0.045411658	0.298124641	0.267047198	0	0	0.139104652
Gastroenterology 2016-ESCC_3	0	0.040950996	0.030532661	0	0.030086605	0.116000109	0.090181486	0.26727939	0.211462589	0.051458213	0.069020278	0.093027671
Gastroenterology 2016-ESCC_4	1.39E-07	0.031149814	0.026249952	0	0.018574004	0.290854863	0.281360735	8.04E-65	0.299632645	0	0	0.052177848

Gastroenterology 2016-ESCC_5	0	0.049916076	0.093437445	0	0.018683279	0.052379315	0.082068057	0.335908844	0.249713028	0.026851883	0.031224388	0.059817684
Gastroenterology 2016-ESCC_6	8.16E-47	0	0.138267215	0	0.033840501	0.115458471	0.069004418	1.35E-05	0.499557544	0	0	0.143858305
Gastroenterology 2016-ESCC_8	0.027801368	0.07977383	0.025362874	0	0.030116202	0	0.058651524	0.35960306	0.138399982	0.048486872	0.047321312	0.184482977
Gastroenterology 2016-ESCC_9	0.157999421	0.080783295	0.161010652	0	0.004657178	0	4.57E-23	0.277128411	0.22964501	0.027835181	0.034362813	0.026578038
Gastroenterology 2016-ESCC_10	0.147972255	0.01523297	0.076945688	0	0.008204191	0.16382291	0.235190648	0.119959778	0.127286858	0.023454485	0.008041661	0.073888556
Gastroenterology 2016-ESCC_11	0.001371833	0.000896217	7.42E-05	0	0.046150772	0.036863852	0	0.372471243	0.444642554	0.07128102	0.026248356	0
Gastroenterology 2016-ESCC_12	0	0.214310586	0.13538453	0	0.022378716	0.047136772	0.053950307	0.231578857	0.070003456	0.07021379	0.045587099	0.109455888
Gastroenterology 2016-ESCC_13	0	0	0.022084183	0.041976034	0	0.272720661	0.323789388	0.118778712	0.220651022	0	0	0
Gastroenterology 2016-ESCC_14	7.76E-45	0.120478754	0.031336743	6.83E-18	0.003952072	0	0.04993796	0.583038977	0.149303751	0.061951744	0	0
Gastroenterology 2016-ESCC_15	0	0.031552897	0.075387246	0.027054333	0.013867305	0.073796387	0.107948144	0.414758021	0.255635665	0	0	9.53E-10
Gastroenterology 2016-ESCC_16	0.098257542	0.286526359	0	0	0.009535904	0.052198842	0.055131972	0.359454877	0.085576528	8.95E-09	0	0.053317966
Gastroenterology 2016-ESCC_17	0	0.160079667	3.27E-100	0	0	0.02299286	0.013921946	0.457189731	0.337709557	3.65E-23	0.008106238	1.24E-49
Gastroenterology 2016-ESCC_18	0.003576913	0.07484058	0.05406411	0	0.022999339	0.084443717	0.132852542	0.275894765	0.173899598	0.013850708	0.003839124	0.159738602
Gastroenterology 2016-ESCC_21	0.001093428	0.1746971	0.153412191	0	0.024001061	0.043300887	0.089925002	0.289151175	0.153817516	0.065645626	0.004956013	0
Gastroenterology 2016-ESCC_22	0	0.326162438	1.88E-261	0	0	0.016665856	0.048925771	0.024174995	0.560631389	1.27E-197	0	0.023439551

Gastroenterology 2016-ESCC_24	0.196274122	0.029823931	0.028258959	0.049289071	0	0.049428838	0.026125469	0.05799511	0.393518395	0	0	0.169286104
Gastroenterology 2016-ESCC_25	0	0.10280475	2.13E-95	3.52E-11	0.015633486	0.289775852	0.276192157	0.148489557	0.158890443	1.63E-87	0	0.008213755
Gastroenterology 2016-ESCC_26	0	0.095036057	0.139708871	0.157290011	0	0.011404619	0.147897368	0.22071812	0.097799057	0	0.018078471	0.112067427
Gastroenterology 2016-ESCC_27	0	0.255264055	0.063596281	0	0	2.70E-146	0.095588646	0.313598898	0.170597864	0.011953251	0.033398209	0.056002796
Gastroenterology 2016-ESCC_28	0.176722808	0.042672788	0.155983448	1.77E-17	0.006103774	6.75E-169	0.069501037	0.34893403	0.100894143	0	0.007313576	0.091874395
Gastroenterology 2016-ESCC_29	0.045618914	0.119557956	0.023660346	6.93E-30	0.047957039	0.102226754	0.279271814	0.066517304	0.200983627	0.041451104	0.003500786	0.069254355
Gastroenterology 2016-ESCC_30	0.020891054	0.100991509	0.030122708	0.041016935	0	0.042489221	0.079327465	0.019898464	0.428473277	0.012340511	0	0.224448856
Gastroenterology 2016-ESCC_31	7.45E-123	0.311203364	1.29E-257	1.33E-14	2.06E-151	0.306635459	0.165944447	3.94E-73	0.216216729	9.98E-99	2.12E-119	2.20E-145
Gastroenterology 2016-ESCC_32	0	0	0.046524825	0.008407706	0	0.178714046	0.334793905	0.033360852	0.324512768	0.002226062	0	0.071459835
Gastroenterology 2016-ESCC_33	0.036738531	0.12033571	0.080259942	0.079343443	1.11E-201	0.007463609	0.127245104	0.339170776	0.209442885	4.98E-117	3.19E-244	0
Gastroenterology 2016-ESCC_34	0.394939241	0	0.180746242	0	0.033659696	0.021663133	0.079991141	0.097798461	0.091233147	0.027133135	0.072835804	0
Gastroenterology 2016-ESCC_35	0	0	0.028173453	0.008015595	0.035697308	0.183271901	0.225968456	0.16339097	0.308190633	0	0	0.047291684
Gastroenterology 2016-ESCC_36	0.000314828	1.06E-24	0.077180127	0	0.115942018	0.025188622	0.037319704	0.121960399	0.400952081	0.00721913	0.011833629	0.202089461
Gastroenterology 2016-ESCC_37	6.26E-28	0.09607065	0.03486327	0	0.02067485	0.001325016	0.086669446	0.278407173	0.237778221	1.46E-09	0.077580301	0.166631071
Gastroenterology 2016-ESCC_38	8.45E-45	0.119493104	0.012274029	0	0.017679494	0.125791518	0.034239923	0.388754725	0.127682298	0.05023929	0.067900765	0.055944854

Gastroenterology 2016-ESCC_39	0	0.251754791	0.061247744	0	0	0.005421039	0.075248324	0.121720866	0.240626572	0.091629346	0.10062326	0.051728059
Gastroenterology 2016-ESCC_40	0.176397169	0.155415217	0.008297337	0.074595087	0.038527082	0.019408713	0.049817161	0.18607467	0.123538524	7.50E-105	0.057766078	0.110162962
Gastroenterology 2016-ESCC_41	0	0.301698886	0.127998339	0	0	0.020699273	0.049764315	0.227396624	0.256680967	0.00734617	0.008415425	4.13E-100
Gastroenterology 2016-ESCC_42	0.012958858	0.146770313	0	6.77E-21	0.000864297	0.099247397	0.129936497	0.295511385	0.181739723	0.034101456	0.064867829	0.034002246
Gastroenterology 2016-ESCC_43	0	0.200525016	0.05233815	0	0	0	0.162623981	0.239228147	0.042037882	0.04273439	0.003382457	0.257129976
Gastroenterology 2016-ESCC_44	0.013075168	0.022964196	0.069410023	0	0.036917509	0.113210704	0.083045188	0.35434442	0.134235197	0.037023827	0.034337536	0.101436231
Gastroenterology 2016-ESCC_45	0.119021943	0	0.008173374	0	0.066924768	0.233856774	0.109129972	0.06135793	0.303426374	0.03460161	0.062959542	0.000547714
Gastroenterology 2016-ESCC_46	0.250081316	0	0.113227329	0	0.001449414	0.104819927	0	0.421458202	0.100929432	0.008034381	2.94E-40	0
Gastroenterology 2016-ESCC_47	0	0.249944256	0.080851299	0	0.010119208	0.018481023	0.035349241	0.252662332	0.270872427	0	9.84E-53	0.081720213
Gastroenterology 2016-ESCC_50	0	0	0.028123997	0	0.045637849	0.030539631	0.041097017	0.357468828	0.394260803	0	0.015123984	0.08774789
Gastroenterology 2016-ESCC_51	0.021268499	0.032251889	0.075063421	0	0.013251878	0.045994264	0.096470257	0.128117327	0.350118294	0.020247978	0	0.217216194
Gastroenterology 2016-ESCC_52	0	0.068985539	0.15904598	0	0	0.012665338	0.087151285	0.195041423	0.271967425	0	0.003248105	0.201894904
Gastroenterology 2016-ESCC_53	0.040850215	0.150988501	0.012379206	2.65E-30	0.202573065	0.015965349	0.079013557	0.022111669	0.303214809	0.01301554	0	0.15988809
Gastroenterology 2016-ESCC_54	0	0.108383559	0.114141814	0	0	0.149669729	0.086689611	0.231142702	0.151157633	0.025490822	0.042346485	0.090977644
Gastroenterology 2016-ESCC_55	0	0	0.008148077	0	0.002261332	0.353947311	0.294024805	0.201542906	0.140075569	1.27E-25	0	0

Gastroenterology 2016-ESCC_56	0	0	0.149406446	0	0	0.012806048	0.037050281	0.141984141	0.612371511	0	3.30E-183	0.046381572
Gastroenterology 2016-ESCC_57	1.79E-23	0.088115929	0.034165312	8.21E-52	0	0.196335857	0.102869915	0.379672944	0.197728653	0.00111139	5.82E-160	0
Gastroenterology 2016-ESCC_58	1.03E-62	8.54E-68	0.03515311	9.37E-57	0	0.037898266	0.078652383	0.519731519	0.229419703	2.49E-161	0.012603184	0.086541836
Gastroenterology 2016-ESCC_60	0.096940201	0.376031746	0.023124401	0.062828779	0.013026134	0	0.029838715	0.166781427	0.162439111	0	0.010006108	0.058983378
Gastroenterology 2016-ESCC_61	5.49E-06	0.250145253	0	0.007877104	0	0	0.032353107	0.44116462	0.060886123	0.048499475	0.087846591	0.071222238
Gastroenterology 2016-ESCC_62	1.19E-45	0.037956737	0.069139034	0	0	0.294195356	0.185507136	0.241839523	0.146759434	0.017779784	0.006822997	0
Gastroenterology 2016-ESCC_63	0	0.035399906	0.274301298	0	0.010157925	0.008865877	0.019501223	0.157464444	0.184987765	0.000194254	0.0464663	0.262661007
Gastroenterology 2016-ESCC_64	0	0.078331155	0.106159688	1.44E-22	0.060480746	0.027397639	0.016622421	0.000170211	0.467599221	0	0.025474827	0.217764093
Gastroenterology 2016-ESCC_65	0	0.193489875	0	0	0.005246214	0.011111616	0.046943428	0.239877403	0.19531226	0.075136044	0.050860387	0.182022774
Gastroenterology 2016-ESCC_66	0.094350158	0.364903993	0	1.19E-58	0	0	0	0.341394874	0.167334026	0.011749855	0	0.020267094
Gastroenterology 2016-ESCC_68	0	0	0.016576915	0.004896361	0.007303119	0.345933476	0.18959944	0.179571458	0.228750348	0.027368883	0	1.62E-24
Gastroenterology 2016-ESCC_71	0	0.03043469	0.297480477	0	0.026396126	0.003531814	0.069208242	0.034181547	0.358441145	0.073905575	0.0228535	0.083566884
Gastroenterology 2016-ESCC_72	0.001817056	0.111306	0.135951489	0	0.041482022	0.056009644	0.031837302	0.347911412	0.231639046	0	0.042046028	0
Gastroenterology 2016-ESCC_73	4.12E-27	0.115548545	0.18919002	0.043077334	0	0.123239906	0.110270377	0.159032032	0.214655258	0.041280181	7.79E-71	0.003706347
Gastroenterology 2016-ESCC_74	2.78E-41	0	0.101486714	0.032820197	0.012766249	0.065737217	0.115008121	0.019902803	0.311318443	0	0.012374873	0.328585384

Gastroenterology 2016-ESCC_75	0	0.2081588	0.092890145	0	0.024463317	0.078935748	0.13935213	0.131432088	0.213770621	0.032167062	0.018875316	0.059954773
Gastroenterology 2016-ESCC_76	0.147623726	0	0.142183102	0	0	0.134618457	0.136556861	0.177894646	0.231631943	0.00942165	0.01326976	0.006799856
Gastroenterology 2016-ESCC_77	0	0.129095989	0.220521524	0	0.063882244	0.061396345	0.027163456	0	0.365061313	0.054274065	0.013940652	0.064664412
Gastroenterology 2016-ESCC_78	0.141927124	0.193614461	0.105819851	0	0	0	0.025939319	0.310212618	0.181799612	0.040687014	8.65E-109	7.19E-26
Gastroenterology 2016-ESCC_79	5.98E-48	0.011907281	0.023759852	0.050684231	0	0.091094236	0.067296038	0.199352211	0.403108003	1.94E-112	0	0.152798148
Gastroenterology 2016-ESCC_80	6.20E-54	0.008771266	0.029646914	0	0.0064604	0.033447136	0.061648882	0.39856961	0.176823688	0.083742213	0.004280367	0.196609524
Gastroenterology 2016-ESCC_81	1.10E-128	0.143474235	9.18E-83	0	0.029245769	0.045100196	0.071402124	0.211101082	0.343920593	0.020962414	0.024902084	0.109891503
Gastroenterology 2016-ESCC_82	0.102212379	0.086474874	0.005626379	0	0.04527761	0.220271813	0.089905437	0.038124885	0.396286891	0.014099453	0	0.00172028
Gastroenterology 2016-ESCC_83	0	0.403484202	0	0	0	0.081332279	0.138555386	0.120360788	0.176066282	1.63E-62	0	0.080201063
Gastroenterology 2016-ESCC_84	0.228361571	0.028818724	0	0	0.00349529	0.138245925	0.031401655	0.087715172	0.063061521	0.074190391	0	0.34470975
Gastroenterology 2016-ESCC_86	0.066683859	0.086638481	6.18E-80	0	0.089279715	0.098347628	0.077968059	0.256167834	0.246941478	0.030734981	0.047237965	3.30E-19
Gastroenterology 2016-ESCC_87	0.049651081	0.106750603	0.028475671	0	0.085239502	0.011221504	2.38E-69	0.370295261	0.177536411	0	0.09976268	0.071067287
Gastroenterology 2016-ESCC_91	0	0.041041825	3.73E-52	0	0.049389556	0.1110842	0.167640547	0.14132987	0.48145431	0.008057694	2.70E-77	2.00E-06
Gastroenterology 2016-ESCC_92	0	0	0.176478463	0	0.022196868	0.042703733	0.129146892	0.03616888	0.586550828	0.006754336	1.84E-142	0
Gastroenterology 2016-ESCC_93	1.03E-36	0.004980638	0.175995335	0	0.024637381	0.040688197	0.032293977	0.171736745	0.380741561	0.009970794	0.005596506	0.153358867

Gastroenterology 2016-ESCC_94	0	0.18412716	0	0.038778412	0.021312034	3.359646391 72048e-322	0.105830703	0.321111144	0.236817664	2.53E-38	0.043391379	0.048631504
Gastroenterology 2016-ESCC_95	1.74E-36	0	0.023993526	0.040854532	0.036770893	0.170305672	0.062745859	0.069079852	0.342634152	0.031462012	8.14E-90	0.222153501
Gastroenterology 2016-ESCC_96	0.118938519	0	0.089027774	0	0.012363755	0.037455454	0.02713992	0.39445952	0.235544957	0.030197897	2.33E-40	0.054872204
Gastroenterology 2016-ESCC_99	0.016574303	0.032392494	0.211179345	0	0	0.06669052	0.052511361	0.227583322	0.171839033	1.89E-08	0.016643686	0.204585916
Gastroenterology 2016-ESCC_100	0	0.174878239	0.070214491	0.142780984	0.038351552	0.120871793	0.100797791	0.164435671	0.187669479	0	0	1.35E-47
Gastroenterology 2016-ESCC_101	0	0.044031733	0.16531039	0.043161219	0	0	0.037913836	0.281005069	0.264488465	0.04109436	0.041353897	0.081641032
Gastroenterology 2016-ESCC_102	0	0.124344748	0.127454931	0	0	0	0.025119438	0.264897663	0.248616089	0.0889093	0.016869814	0.103788017
Gastroenterology 2016-ESCC_103	0.206394896	0	0.231707971	0	0.009485811	0.062159807	0.087364879	0.005969681	0.085770002	0.025531712	0.056427722	0.22918752
Gastroenterology 2016-ESCC_104	0.026340975	0	0.035699685	0	0.00483967	0.133396276	0.034397129	0.411854133	0.073841734	0.013363607	0.189118604	0.077148187
Gastroenterology 2016-ESCC_107	0	0	0.099218363	0	0.028701645	0.155669841	0.099058612	0.071361318	0.291740513	4.75E-34	0	0.254249707
Gastroenterology 2016-ESCC_108	2.84E-67	0.037223673	3.24E-133	0	0.060907838	0.165256567	0.124651756	1.22E-24	0.284338914	0.028305936	0	0.299315317
Gastroenterology 2016-ESCC_109	0.068670255	0.053223228	5.82E-92	0	0.000246269	0.285187802	0.350342886	1.03E-28	0.188229661	0	6.14E-32	0.054099899
Gastroenterology 2016-ESCC_110	0.037053917	0.03779331	0.209197331	0.045200587	0.092639266	0.008754828	0.009261581	0.244227724	0.256140909	0	0	0.059730548
Gastroenterology 2016-ESCC_111	4.72E-09	0.175058939	0.006480354	0	0.06261839	0.07776363	0.051876479	3.74E-25	0.266832752	0.06663754	0.052427323	0.240304589
Gastroenterology 2016-ESCC_112	0.07276584	0.050197364	0.108152074	0	0	0.015852004	0.025899566	0.229414464	0.384630524	0.00788703	0	0.105201135

Gastroenterology 2016-ESCC_113	0	0.218715928	0.067964401	0.023596322	0.015806568	0	0.037843018	0.212552155	0.142956528	0	0	0.28056508
Gastroenterology 2016-ESCC_114	0	0.056071256	0.11563772	0	0.046193322	0.104664336	0.049604968	0.291828788	0.272250665	0.053044235	1.27E-265	0.010704711
Gastroenterology 2016-ESCC_115	0	0.037710476	0.105540915	0	0	0.064260669	0.111479046	0.063565821	0.493142885	1.69E-05	1.02E-238	0.124283291
Gastroenterology 2016-ESCC_116	0.005546279	0	0.144580547	0.01292468	0.038813792	0.024724088	0.128028123	0.159738241	0.252102832	0	0.052203084	0.181338334
Gastroenterology 2016-ESCC_117	0.064206681	0.014456657	0.038478073	0	0.007539571	0.044055617	0.034846048	0.370354538	0.208718529	0.003731467	0.06855128	0.14506154
Gastroenterology 2016-ESCC_118	0	0.02043848	0.13710023	0	5.99E-18	0.163146743	0.162705744	0.117905462	0.301552409	0.003721294	0.002551186	0.090878452
Gastroenterology 2016-ESCC_120	0	0.212685714	0.215011618	0	0.017928539	0.007210012	0.036345163	0.047390042	0.233465337	0.019325573	1.35E-82	0.210638002
Gastroenterology 2016-ESCC_121	1.37E-65	0.033829004	0.039003325	0	0.08769107	0.019300682	0.025999534	0.209974493	0.387192086	0.029859612	0.024880818	0.142269375
Gastroenterology 2016-ESCC_122	5.70E-62	0.108670819	0.052592	2.09E-32	0	0.213078006	0.21860057	0.052072768	0.220571413	0.018374446	0.000176532	0.115863447
Gastroenterology 2016-ESCC_123	5.37E-40	0.23119845	3.68E-127	1.63E-15	0.034928469	0.050977276	0.106911472	0.018034293	0.352400921	0.023896711	0.015870068	0.16578234
Gastroenterology 2016-ESCC_124	7.59E-25	0.120611217	0.248260996	0	0.00074285	0.066431407	0.063570124	2.20E-55	0.303467819	0	1.96E-37	0.196915588
Gastroenterology 2016-ESCC_125	7.84E-91	0	2.45E-42	0	0	0.019838403	0.090420963	1.23E-29	0.649746383	0.028625442	0.003521676	0.207847133
Gastroenterology 2016-ESCC_126	0	0.053385106	0.116579961	0.014926529	0.01116574	0.099312546	0.082838879	0.196433646	0.242145562	0.032632616	0.057229146	0.093350269
Gastroenterology 2016-ESCC_127	3.35E-05	0.247384437	0.067259164	0	4.99E-164	0.006447625	0.020192511	0.330407726	0.257914156	7.92E-104	3.00E-38	0.070360907
Gastroenterology 2016-ESCC_128	0	0.269529549	1.97E-37	3.55E-12	0	0.026027445	0.052489668	0.241843367	0.353753022	0.007946332	0	0.048410617

Gastroenterology 2016-ESCC_129	0.277579161	0	0.297980782	0.049934808	0	0.032952249	0.032729174	0.23367985	0.026278856	0	0.002810122	0.046054999
Gastroenterology 2016-ESCC_130	0	0	0.01560826	0.037827613	0.037328006	0.046020539	0.047232099	0.218471654	0.29648924	0	0.000364965	0.300657624
Gastroenterology 2016-ESCC_131	0	0.015063382	3.68E-09	0	0.056440697	0.185635686	0.152982912	0.24650784	0.204051924	0.035623304	0.072576194	0.031118059
Gastroenterology 2016-ESCC_132	2.34E-21	0	0.248641935	0	0.005474607	0.005061239	0.038767277	0.258501308	0.294640194	0	0.055668348	0.093245092
Gastroenterology 2016-ESCC_133	0.017269998	0.118161952	0.030621439	0.017699879	0.004549404	0.137051541	0.092789614	0.242785471	0.242440108	0.028803103	0.005560289	0.062267202
Gastroenterology 2016-ESCC_134	2.79E-57	4.13E-44	0.017408058	0.009751509	0.012322802	0.307058302	0.148065352	0.184485311	0.245404169	0.006508479	0.025850157	0.04314586
Gastroenterology 2016-ESCC_135	8.45E-39	0.029410141	0.105660632	0.011537741	0.002957501	0.114852658	0.07040853	0.170496486	0.235317698	0.107689125	0.000920589	0.150748898
Gastroenterology 2016-ESCC_136	1.33E-06	0	0.217451919	0.009154313	0.021724629	0.091841367	0.037496139	0.225527411	0.249501018	0	0	0.147301877
Gastroenterology 2016-ESCC_137	0	0.205015743	0.053586972	0	0.051886809	0.04885636	0.02326909	0.17326654	0.208909178	0.014088966	0	0.221120343
Gastroenterology 2016-ESCC_139	0.001404686	0.018207908	0.053541435	0	0.025951784	0.027075765	0.12871884	2.22E-31	0.519197132	0	0	0.22590245
Gastroenterology 2016-ESCC_140	1.35E-57	8.99E-43	0.084283738	2.64E-08	0	0.081373406	0.188136629	1.34E-45	0.422856004	0.021377936	0.030499249	0.171473012
Gastroenterology 2016-ESCC_141	0.199245369	0.08174309	0.097169382	0	0.010144871	0.077030677	0.026165671	0.063868105	0.285110998	0	0.062701407	0.096820431
Gastroenterology 2016-ESCC_142	0	0.071949335	2.50E-36	2.34E-11	0.005520305	0.163030282	0.186225531	0.139262366	0.399201889	0.004813976	1.01E-98	0.029996316
Gastroenterology 2016-ESCC_143	0	3.63E-11	0.002650258	0	0.060460089	2.09E-109	0.003039862	0.057361808	0.045432091	0.065903522	2.01E-135	0.76515237
Gastroenterology 2016-ESCC_144	0	0.132508553	0.001009708	0	0	0.023661754	0.054858946	0.283487971	0.39816376	0.015244665	0	0.091064642

Gastroenterology 2016-ESCC_146	1.07E-37	0.086842349	0.324955141	0	0.035537403	0	0.090793384	0.103445239	0.273748121	0.000518183	0.03211695	0.05204323
Gastroenterology 2016-ESCC_149	2.02E-36	0.312365272	0.038382103	0	8.17E-51	0.028171571	0.00154629	0.293105856	0.194119677	0.004778436	0.021237583	0.106293212
Gastroenterology 2016-ESCC_150	0	0.13153218	0.159612293	0	0	0.062202988	0.019425077	0.166211567	0.275142742	0.078392109	0.07761875	0.029862293
Gastroenterology 2016-ESCC_151	0.015460682	0.006887276	0.085505992	0	0.007400846	0.00526214	0.034185334	0.563208653	0.173558467	0.06947317	0.021111643	0.017945797
Gastroenterology 2016-ESCC_152	0.039796094	0.039596514	0.055869428	0	0.066045573	0.097980608	0.093264167	0.058917702	0.392506842	0.048995823	1.39E-07	0.107027108
Gastroenterology 2016-ESCC_153	0.050519409	0.143965661	0.000836632	0	0.003515811	0.184160402	0.123133114	0.296140668	0.179077185	0.016593112	5.77E-53	0.002058007
Gastroenterology 2016-ESCC_154	1.04E-70	0.055200171	0.026841255	0	0.029086367	1.73E-11	0.10275286	0.00721081	0.614969872	0	0.040937117	0.123001548
Gastroenterology 2016-ESCC_156	2.81E-56	0	0	0	0.006043469	0.178779258	0.130849931	0.142232634	0.408855126	0	0.010302531	0.122937051
Gastroenterology 2016-ESCC_157	0	0.000205409	0	9.31E-23	0	0.288031524	0.337473631	0.156580061	0.180421612	0.024461991	0.012825773	0
Gastroenterology 2016-ESCC_158	0	0.030085123	0	0.009022484	0.032290339	0.305488383	0.186456264	2.93E-46	0.349935292	2.71E-112	0	0.086722115
Gastroenterology 2016-ESCC_159	0.005195562	3.38E-35	0.151160816	0	0.057141577	0.080547859	0.153793317	0.052947918	0.437123707	1.48E-26	0.014409758	0.047679486
Gastroenterology 2016-ESCC_160	0	0	0.107849564	0.042561854	0	0.327838579	0.174763278	0.07112855	0.256113558	0	3.00E-22	0.019744617
Gastroenterology 2016-ESCC_161	1.41E-61	5.08E-58	0.026524571	0.017478381	0	0.293244165	0.176844396	0.026731639	0.273176106	0.065315434	0	0.120685309
Gastroenterology 2016-ESCC_162	0.071359452	0	0	0	0.029228898	0.3877955	0.118359173	8.09E-73	0.104469533	0	0.009649654	0.279137791
Gastroenterology 2016-ESCC_163	0	8.73E-34	0.229820398	0.026034362	0	0.042863944	0.031987221	0.384663689	0.276613415	0.008016971	0	0

Gastroenterology 2016-ESCC_164	0	0.168719753	0.063858605	1.52E-19	0	0.152624154	0.212846591	0.169494134	0.222273969	8.62E-48	0	0.010182794
Gastroenterology 2016-ESCC_165	0	0.132963805	1.07E-25	0.138437619	0.010935957	0.044674986	0.016695778	0.138007689	0.433351714	8.46E-127	3.37E-81	0.084932452
Gastroenterology 2016-ESCC_167	0.008575566	0.131213881	0	0.052490046	0	0.085018048	0.042952461	0.166439962	0.477778187	0	0	0.035531849
Gastroenterology 2016-ESCC_168	0	0.070416632	0	0	0	0.161566039	0.032422752	0.321329779	0.245346728	0.056431452	0.012701634	0.099784986
Gastroenterology 2016-ESCC_169	0	0	2.93E-231	0	0.008285158	0.22140482	0.213836274	1.28E-18	0.464663872	1.02E-27	0	0.091809875
Gastroenterology 2016-ESCC_170	0	0.116512788	0.112427916	1.40E-06	0	0.456457133	0.168295819	0.025579578	0.120725368	0	0	2.23E-24
Nature2014-ESCC-001T	0	0	2.42E-21	0	0	0.233922798	0.223060578	0.034285394	0.50873123	6.57E-106	0	0
Nature2014-ESCC-002T	0.060167723	0.137645264	0.119550258	0	0	0.212489546	0.09342517	0	0.257978196	0.049772123	0	0.068971721
Nature2014-ESCC-003T	0	8.66E-26	0.192343903	3.24E-44	0	0.112627933	0.153549362	3.69E-127	0.47250851	4.86E-93	0	0.068970292
Nature2014-ESCC-004T	0.253467238	0	0.035299184	0.001489061	0.074472593	0.047112438	0	0.046856728	0.300722085	0	0.080960447	0.159620226
Nature2014-ESCC-005T	0.015387077	0.010086737	0	6.65E-32	0.023958609	0.168417933	0.045348449	0.152484739	0.273701236	0.18509594	1.14E-89	0.12551928
Nature2014-ESCC-006T	7.66E-162	1.28E-108	1.46E-25	0.062251525	0.012086654	0.064794923	0.028330841	0.502237347	0.326187739	0.004110972	0	2.95E-79
Nature2014-ESCC-008T	0	0	0.209877075	0	0.072823675	0.073235161	0.002539721	7.82E-06	0.222785443	0	0.067965159	0.350765947
Nature2014-ESCC-009T	0.068402325	0	0.145816622	0	0.022457577	0.135821684	0.179108568	0.111468634	0.161325186	0.020305819	0.031133881	0.124159704
Nature2014-ESCC-010T	0.083260812	0	0.077170259	0	0.063944983	0.277778381	0.079991199	0.137321841	0.170058426	0	0.110474098	0

Nature2014-ESCC-011T	0	0.034389268	0.057090932	5.88E-05	0.141332191	0.038006198	0.10298449	2.74E-16	0.626138083	0	0	2.55E-154
Nature2014-ESCC-012T	0	0.081128308	0.192470607	0.015354011	0.039134777	0.012298297	0.07071338	0.045243008	0.477669823	0	1.10E-134	0.06598779
Nature2014-ESCC-013T	0.016022725	0	0	1.69E-110	4.04E-214	0	0	0	0.523418298	0	0	0.460558977
Nature2014-ESCC-014T	0	0.264337868	7.00E-112	0	0.020963161	0.055575191	2.27E-66	0.489154936	0.070928716	0	0.062633377	0.036406752
Nature2014-ESCC-015T	0	0.012677777	0.131663919	0	0.041199992	0.103691605	0.192494376	0.109554793	0.369009943	0	0.039707595	0
Nature2014-ESCC-016T	0	0	0.207663974	0	0	0	0	0.224146042	0	0	0.568189984	0
Nature2014-ESCC-017T	0	0.093594376	0	3.60E-144	0	0.129731512	0.23655266	0	0.489326127	1.47E-141	7.93E-37	0.050795325
Nature2014-ESCC-018T	0	0.053851747	0.144589168	0	0	0.177660836	0.049803391	2.99E-130	0.423622392	0	0.006085856	0.14438661
Nature2014-ESCC-038T	4.43E-07	0	0.09970652	0	0.020869205	0.12130953	0.006242436	0.197721376	0.49384644	0	0.039342561	0.020961489
Nature2014-ESCC-039T	0	0	0.021587965	0	0	0.039317832	0.023549962	0.419199706	0.39896885	0.011396803	0	0.085978882
Nature2014-ESCC-055T	0	0.0752704	0.149964085	0	0.055471623	0	0.057410512	0.336165931	0.305349915	0.001247569	0.019119965	0
Nature2014-ESCC-057T	0	0	0.046177253	1.18E-133	0	0.171689564	0.087260334	0.123799572	0.516254576	0	2.10E-11	0.054818701
Nature2014-ESCC-059T	6.07E-117	0.120874445	0.064926713	0	0.00507005	0.084725486	0.018035565	0.481991042	0.024464676	0.072604155	0.0091529	0.118154968
Nature2014-ESCC-060T	2.68E-46	4.39E-70	0.138702931	0	0	0.056378671	0.029383847	0.256461862	0.237082719	1.52E-75	0.071512305	0.210477664
Nature2014-ESCC-069T	2.66E-189	3.55E-86	0.240864006	0	0.014326984	0.026513281	0.045359624	0	0.572806406	5.62E-105	0	0.100129698

Nature2014-ESCC-070T	0	0.11128948	5.83E-77	0	0.033951766	0	0.016252146	0.324416911	0.308186402	0.03609553	0.064498422	0.105309342
Nature2014-ESCC-078T	8.38E-10	0	0.09190139	0	0.000573621	0.249628434	0.1650072	0.165262725	0.230809241	0.063273809	0.033543579	0
Nature2014-ESCC-083T	4.92E-85	0	0.208901574	0	0.029776496	0.054510328	0.078763095	0.015887538	0.434957973	0.019853696	0.033603356	0.123745945
Nature2014-ESCC-092T	0.007421042	0.019194136	0.187156946	2.25E-17	0	0	0.41308917	0	0.373138706	0	0	0
Nature2014-ESCC-098T	0	0.034083288	0.122683165	0.021894218	0	0.077364112	0.116769067	0.193002191	0.42585596	0.008348	1.50E-83	0
Nature2014-ESCC-100T	0.075326109	0	2.16E-158	0	0	0.057301475	0.009163791	0.150557115	0.5927838	0	0	0.11486771
Nature2014-ESCC-107T	0	0	0	0	0.066306569	8.04E-243	3.28E-70	4.45E-97	0.233690196	0	0.043186707	0.656816528
Nature2014-ESCC-110T	0.12329589	1.03E-61	0.188098375	7.06E-59	0.01605246	0.00600711	0.067107991	0.15222004	0.351128843	0	0.041141235	0.054948055
Nature2014-ESCC-112T	0	0.014785176	0.166965051	0	0	0.213021484	0.153408756	1.88E-34	0.35022398	0	0.101595552	3.84E-141
Nature2014-ESCC-116T	4.90E-51	0	7.91E-43	0.023191513	0	0.0835249	0.093505164	0.122608841	0.585475853	0	0	0.091693728
Nature2014-ESCC-123T	0.005992567	0	0.069462892	3.55E-11	0.022506281	0.09874478	0.154922454	0.150220135	0.462822462	0.000554383	0	0.034774046
Nature2014-ESCC-127T	0.030252902	0.059942523	0.055044859	0	0.025304694	0.014085776	0.071239895	0.471298031	0.08016553	0	0.045286219	0.147379571
Nature2014-ESCC-128T	0	0	0.09695776	0.000579225	0.051372152	3.30E-91	0.098819029	0.471581678	0.153614021	0	0	0.127076136
Nature2014-ESCC-129T	8.95E-142	0.152743419	0.122542803	0	0.045537955	0.115465362	0.093545058	0.031911986	0.370280538	0	0.06797288	2.62E-81
Nature2014-ESCC-133T	0	0.110585183	0.180989686	0	0.037713478	0.024675231	1.7028430979454e-310	0.017586034	0.248388516	0	0.193287575	0.186774295

Nature2014-ESCC-134T	0.167508707	0	0.095129266	0.024373492	0	0.067657455	0.053408848	0.333997547	0.227812446	0	0.03011224	6.06E-66
Nature2014-ESCC-139T	0.046451917	0	0.063357968	0	0.039612228	0.101202012	0.153756712	0.142911714	0.344397063	0.033645494	0.012297447	0.062367444
Nature2014-ESCC-141T	0	0	0.119276318	0	0.035051354	0.082189968	0.131316634	0.141341469	0.480044798	0.010779449	9.62E-09	5.84E-12
Nature2014-ESCC-144T	0.273622765	0	0	0	0	0	0	0	0.64198053	0	0	0.084396706
Nature2014-ESCC-147T	0.012085851	0.0197668	0.076418652	0	0	0.113139446	0.056130623	0.018522464	0.691498612	0	0.012437554	1.21E-82
Nature2014-ESCC-148T	0	0	0.146200014	0	0	0.190878723	0.190111472	0.056659857	0.321871002	0.012384958	0.021265096	0.060628877
Nature2014-ESCC-152T	1.48E-69	3.38E-50	0.109052375	0	0.012522807	0.185356289	0.103878687	0.121902047	0.448654081	0	0.018633714	0
Nature2014-ESCC-153T	1.28E-20	1.14E-16	3.24E-188	2.75E-78	0	0.281655689	0.3490596	1.11E-65	0.286558414	2.23E-53	0	0.082726296
Nature2014-ESCC-154T	0.014652945	0	0.052794335	5.27E-71	0.1698971	0.040597835	0	0.017595667	0.515923889	0.021489754	2.87E-69	0.167048475
Nature2014-ESCC-155T	0	0.999992246	0	0	0	0	0	7.75E-06	0	0	4.59481050632359e-322	7.68E-234
Nature2014-ESCC-158T	0	0.026561403	0.0906693	0	0	0.086110261	0.123359561	0.130306503	0.270167007	0	0.235153337	0.037672629
Nature2014-ESCC-159T	0	0	0.040843898	0.026152954	0	0.206675884	0.220423912	5.60E-08	0.505903295	0	0	7.05E-54
Nature2014-ESCC-162T	2.50E-52	0.050519385	0.007988528	0.071718937	0.047525331	0.130807152	0.148699547	0.127748554	0.35026213	0.002690562	0	0.062039873
Nature2014-ESCC-164T	6.77E-40	0.436980872	0	1.87E-103	0	0.285641217	0	0	0.277377911	0	0	0
Nature2014-ESCC-171T	0.074870483	0	0.613258308	0.135369083	0	0	0.142695467	0.025727221	0.001454738	0.0066247	0	0

Nature2014-ESCC-175T	0	1.70E-41	0.080727375	0	0.006497416	0.080312532	0.091946458	0.185878956	0.180945651	0.028139107	0.027315225	0.31823728
Nature2014-ESCC-178T	0	0	0.127242335	0	0.0166817	0.004594253	0.098233954	0.287580628	0.326986382	0	0.015068114	0.123612634
Nature2014-ESCC-180T	0.041345492	0.170461167	0.055549018	0	0.040727413	0.149814339	0.070914319	0.219873762	0.057907573	0	0.049714366	0.143692552
Nature2014-ESCC-183T	1.43E-107	0.073505139	0.068677364	0.038276424	0	0	0.107176078	0.004835261	0.656083709	0.051446026	5.80E-42	3.32E-168
Nature2014-ESCC-184T	0.06697858	0.006620352	0.007674488	1.73E-16	0.005322654	0.356947671	0.256601123	1.35E-33	0.198920325	0	0	0.100934807
Nature2014-ESCC-185T	0	0.02666934	0.132890519	0	0	0.010133771	0.006219211	0.267247988	0.253514789	0.026288182	0.138453925	0.138582274
Nature2014-ESCC-190T	0.189458841	0.176193858	0.059635227	0	0.013617044	0.02291292	0.012507451	0.184443843	0.163377835	0.041876315	0.135976666	0
Nature2014-ESCC-191T	1.11E-47	0	0.345204754	0.075661804	0	0.026256622	0.096682778	4.64E-05	0.396202089	0	2.84E-109	0.059945526
Nature2014-ESCC-193T	0.259061676	0	0	0.048902275	0	0	0	0.620766899	0	0	0	0.07126915
Nature2014-ESCC-201T	0	0.110131909	0.131328469	0.018581132	0	0.025934501	0.046109268	0.218990136	0.280594954	1.28E-68	0	0.168329631
Nature2014-ESCC-204T	0	1.35E-24	0.016218222	0	0.063879817	0.063369304	0.110830146	4.26E-15	0.278771638	0	0.349653419	0.117277454
Nature2014-ESCC-205T	0.03732829	0.054844133	0.025284298	0.053185263	0	0.100879022	0.174964211	0.176453588	0.366357785	0	5.82E-86	0.01070341
Nature2014-ESCC-207T	0	0	0.00256496	0	0.03523692	0.242185474	0.172217905	0.002220135	0.470427219	0	0.075147387	0
Nature2014-ESCC-209T	0	0.042171285	0	0	0.037723964	0.093349497	0.233190649	0	0.447871738	0	0	0.145692867
Nature2014-ESCC-210T	0.014293158	0.103716507	0.089919437	0	0	0.034980623	0.038154452	0.038454112	0.67601533	0.00446638	0	3.15E-16

Nature2014-ESCC-211T	1.09E-24	0	0.097452065	1.66E-112	0.019294418	0.037139543	0.103529487	0.033389488	0.665260544	0	0	0.043934455
Nature2014-ESCC-212T	2.83E-39	0	0.08743861	2.67E-38	0.091852488	0.050358141	0.044857873	6.83E-20	0.644986804	0	0	0.080506085
Nature2014-ESCC-214T	0	0	0.058361232	7.00E-43	0.024473863	0.104256441	0.10130052	0.003833809	0.443684948	0.073067775	5.78E-32	0.191021412
Nature2014-ESCC-216T	0	0.134214563	0	0	0	0.262357908	0.130061739	0.249309978	0.112518047	2.17E-32	0.083868129	0.027669637
Nature2014-ESCC-219T	0	0	0.042118391	0	0.023951557	0.099211463	0.081042704	0.228310554	0.435346919	0.008655244	0	0.081363168
Nature2014-ESCC-225T	7.78E-57	0	0.085247088	0.013135714	0.036457303	0.131120657	0.035038836	0.108650405	0.53882535	0	0	0.051524647
Nature2014-ESCC-229T	2.14E-140	0.066749558	0.146126061	0	1.95E-205	0.210992964	0	0	0.20557987	0	0	0.370551547
Nature2014-ESCC-230T	0.901981928	0	0	0	9.14021444806306e-322	0.098018072	0	2.80E-240	0	0	0	0
Nature2014-ESCC-231T	0.131832921	0	0.279818129	2.51E-75	0.060265772	0.033208202	0.02149317	0	0.251038637	0.003797918	0.009943537	0.208601714
Nature2014-ESCC-233T	5.78E-79	0.0185613	0.130717843	4.51E-48	1.60E-84	0.079312926	0.064606226	0	0.472331765	2.04E-46	0	0.23446994
Nature2014-ESCC-235T	3.31E-119	1.27E-05	0.125814497	0	0.012462415	0.031911375	0.069686653	0	0.496103916	0	0	0.264008465
Nature2014-ESCC-237T	0	0.199752331	0.025840674	0.012354314	0	0.241343331	0.145841436	0.015158367	0.222894629	5.17E-29	0	0.136814918
Nature2014-ESCC-240T	0.190978238	0.05369748	0.017519914	0	0	0.090972409	0.006168056	0.0608907	0.526095507	0	0.051074193	0.002603503
Nature2014-ESCC-242T	0	0	2.96E-216	0.098755315	0	0	0	0.901244685	0	0	0	0
Nature2014-ESCC-243T	0.22565433	0.059446475	2.42E-09	0	0.0377052	0.092850117	0.019472748	0.324004832	0.208970167	0	0.012217431	0.019678696

Nature2014-ESCC-246T	7.67E-190	2.89E-78	4.04E-80	6.39E-75	0	0.055702107	0.09461935	1.81E-109	0.60934645	0	0	0.240332093
Nature2014-ESCC-248T	0.082633463	0.002753561	7.32E-08	0	0	0.057210433	0.014188695	0.309926346	0.433866559	0	0.020752425	0.078668444
Nature2014-ESCC-249T	0.007699271	3.30E-133	2.61E-199	0.113924245	0.04519183	0	0.140987488	0	0.677805096	0	0.014392071	0
Nature2014-ESCC-250T	0	0.134293157	1.22E-120	0	0.026757342	0.280934514	0.17370492	0.056627251	0.202890122	0.103178387	0	0.021614305
nc2017-ESCC_179	0.042720626	0.081855252	0.169563084	0.031501202	0	0.134014926	0.103033986	0.175646078	0.146796935	0.020101801	0	0.094766109
nc2017-ESCC_185	0	0.076726352	0.117000259	0	0	0.06055666	0.06257946	0.184756807	0.300810173	0.009796748	0	0.187773541
nc2017-ESCC_144	0	0.012726512	0.107664347	0.015378141	0	0.197326463	0.111392831	0	0.379515953	4.13E-142	0	0.175995752
nc2017-ESCC_245	0	0	2.43E-44	2.23E-12	0	0	0.055524382	0.384105153	0.302548235	0	0.136532906	0.121289323
nc2017-ESCC_169	0.320142684	0	0.004621037	0	0.086572718	0.032963953	0.023906256	0.199347217	0.223409177	0	0.011443576	0.097593381
nc2017-ESCC_175	0	0.250434138	0.016781006	0.042601925	0	0.034917622	0.063240068	0.447664687	0.017564638	0.007496441	0	0.119299476
nc2017-ESCC_61	0.08620369	0.102283314	0	0.289690945	0.016208022	0.019741173	0.013059341	0.012452619	0.196666542	0.013120375	0	0.250573978
nc2017-ESCC_23	3.40E-43	0.063233359	0.165608328	3.47E-33	0.075212771	0.035296977	0.102040767	0.01314896	0.326485628	0.053523346	2.72E-78	0.165449864
nc2017-ESCC_170	0	0.416429033	0.205185466	0	0.042076376	0	6.77E-107	0.080584738	0.186525815	0	2.37E-126	0.069198572
nc2017-ESCC_E3	0.170167846	0.035361544	0.075222497	0	0.021528117	0.112770837	0.05778708	0.223196481	0.122484953	0.021371833	0.088654165	0.071454648
nc2017-ESCC_134	0	0.091315043	0.030327268	0.095806824	0.003082465	0.030316963	0.01681457	0.348119677	0.279589961	0.015513112	0.089114119	1.58E-231
nc2017-ESCC_161	0	0	0.054215031	1.18E-32	0.020873676	0.166328083	0.133884715	0.03377966	0.393935433	0	0	0.196983401
nc2017-ESCC_10	7.45E-47	0.0673971	1.29E-32	0.094756173	0.005634141	0.120815662	0.033284293	0.320026012	0.278215778	0.0391824	0	0.04068844
nc2017-ESCC_12	0	0.006901415	0.180035499	0.018879875	0.021386121	0.005610833	0.023605759	1.10E-72	0.472432161	0	0	0.271148337
nc2017-ESCC_168	0.040048059	0	0.1909603	0	0	0.005243702	0.162475694	0.097670084	0.300173785	0	0.044850506	0.15857787
nc2017-ESCC_208	0	0	0.017896353	0.001411583	0.010595031	0.282642632	0.275021318	0.09846795	0.313952889	1.22E-05	0	1.86E-27
nc2017-ESCC_150	2.27E-11	0.318397187	1.06E-77	0.000844949	0	0.068808731	0	0.268879814	0.130092277	0.049371239	0.025347675	0.138258128
nc2017-ESCC_196	0	0	0.019969114	1.82E-75	0.008017449	0.109977629	0.325185179	2.07E-16	0.484479903	0	3.70E-259	0.052370725
nc2017-ESCC_243	0	0.207418193	0.198398197	0.04949226	0.003812333	0.008189228	0.009725518	0.228570747	0.198504572	0.011649611	9.05E-61	0.084239339
nc2017-ESCC_57	0	0.153922796	0.159358483	0	0.044744416	2.52E-26	0.109473205	0.293537824	0.147104506	0	0.0191486	0.072710169
nc2017-ESCC_213	2.21E-15	0.175829148	0.026495126	0	0	0.014842269	0.081739083	0.2174477	0.208573092	0.106633502	0.016570651	0.151869429

nc2017-ESCC_130	0	0.146718172	0.039201475	0.022313344	0.03321635	0.193901399	0.115461044	8.49E-10	0.397672021	0	0	0.051516194
nc2017-ESCC_E45	0	0.016589105	0.117990362	0	0.077565786	0	0.092923184	0.230557597	0.349134996	0.02375803	0.044074403	0.047406537
nc2017-ESCC_191	0	0.017729044	0	0.027345306	0.010245004	0.308071155	0.297245825	0.06663039	0.272733275	0	0	0
nc2017-ESCC_55	1.52E-63	0.070936336	0.101774362	0.003929379	0.009252662	0.097990338	0.119321896	0.006038534	0.387321231	0	0	0.203435263
nc2017-ESCC_39	0	0.154649483	1.22E-12	0.010278291	0.075608158	0.139909857	0.094549731	0.213018632	0.16029283	0.002089995	0.058588769	0.091014254
nc2017-ESCC_240	1.59E-92	0.072090374	0.05055504	0	0	0.083406398	0.135577652	0.109547454	0.237993676	0.000315871	0.000689755	0.30982378
nc2017-ESCC_E25	0	0	0	0	0.057744659	0.016629028	0.006952595	4.90E-31	0.459001187	0.006812984	0	0.452859547
nc2017-ESCC_E26	0.3843025	0.345095481	1.37E-09	0.09016615	0.025985942	0.076814219	0.077635706	0	1.697928577<br>29804e-316	0	0	0
nc2017-ESCC_239	0	0.054725228	0.164549006	0	0	0.029732897	0.034293081	0.235499542	0.290820447	6.36E-19	0	0.1903798
nc2017-ESCC_171	0	4.66E-15	0.037319948	0	0.035989346	0.080374013	0.108108743	0.303521821	0.21804706	0.005439915	0.016617341	0.194581812
nc2017-ESCC_158	0	0.038153147	0.007786879	0.064626594	0.025143789	0.055901128	0.039540755	0.20068803	0.353675786	0	0.000770887	0.213713004
nc2017-ESCC_143	0	0	0.045443512	0	0.003638023	0.211775438	0.309845826	1.44E-34	0.429297201	9.97E-71	0	0
nc2017-ESCC_222	0	0.062850586	0.319357275	0	0.055601765	0.173320096	0.167856108	0.108034773	0.034244688	0	0.026021381	0.052713328
nc2017-ESCC_152	0.143007334	0.096367671	0.126735955	0.046248676	0.034883072	0	0.033013829	0.083996138	0.393010432	0	0.02391326	0.018823632
nc2017-ESCC_142	0.059713209	0.050977126	0.038191353	0	0	0.12370169	0.193143001	0.103265172	0.284765984	0.034071544	0.048192368	0.063978551
nc2017-ESCC_224	0	0.090951003	9.41E-192	0.024000945	0	0.102426302	0.258727815	1.07E-36	0.365208697	0.035043687	0	0.123641551
nc2017-ESCC_3	0.044331377	0.168655047	0.064640877	0	0.064995308	0.148591261	0.149300741	3.30E-47	0.336096336	5.02E-94	0.004380033	0.019009018
nc2017-ESCC_E79	4.84E-63	0.013052329	0.055982745	0.050926891	0.026329369	0.03289422	0.054343876	0.073086329	0.448082336	0	0	0.245301905
nc2017-ESCC_54	2.64E-33	0.099072259	0.272872183	7.77E-31	0.038842888	0	0.179835983	0.039092934	0.158202474	5.71E-44	0.008159307	0.203921972
nc2017-ESCC_62	0.017435815	0	0.126277943	0	0.021566675	0.0351509	0.019324047	0.21545037	0.438556193	0	0.004983011	0.121255045
nc2017-ESCC_65	1.42E-89	0.267997799	0	0.01768706	0.011931663	0.053767877	0.024554658	1.15E-39	0.348561344	0	0.011226489	0.264273109
nc2017-ESCC_156	0.194202634	0.041720638	0.043633935	0	0.05592676	0.106158851	0.103338232	0.310193901	0.10985179	0	0.011915005	0.023058254
nc2017-ESCC_64	0.052695902	0	0.126075629	0.021824421	0	0.028402072	0.04376208	0.292424669	0.181703009	0.031598242	0.004168259	0.217345717
nc2017-ESCC_215	3.95E-19	0.033852738	0.096950521	0	0.008284837	0.097954282	0.098022511	0.331631147	0.186547942	0.071561368	0.013685669	0.061508985
nc2017-ESCC_140	0	0.073849105	0.107996758	0.12652698	0	0.10916021	0.048396772	3.44E-36	0.414399023	0	4.33E-21	0.119671152
nc2017-ESCC_26	0	0	4.13E-93	0	0	0.0952237	0.144104356	0.341984575	0.357682775	0	0.002001053	0.05900354
nc2017-ESCC_172	0	2.14E-97	1.68E-24	0	0.053771739	0.151602892	0.067059926	0.213151792	0.295722444	1.01E-19	0.017305264	0.201385943
nc2017-ESCC_198	0	0.10653209	0.173873246	0.082903155	0	0	0.010335426	0.194310493	0.201469431	0.003972032	0.066772733	0.159831395

nc2017-ESCC_125	0	0.522360505	0	0	0	0.007313561	0.013637899	5.94E-24	0.280921889	0	0.028979724	0.146786422
nc2017-ESCC_131	0	0	1.07E-112	0	0.046133494	0.006463635	0.076204302	0.076195244	0.795003325	0	0	0
nc2017-ESCC_48	3.24E-40	0.2613522	4.41E-54	0	0.012002509	0.02606806	0.067939359	0.261172679	0.211643178	0.049834465	0.045943894	0.064043655
nc2017-ESCC_42	0.069851313	0.108988951	0.115066464	0	2.17E-09	0.159087978	0.084480833	0.006221233	0.243819126	0	0.057580006	0.154904094
nc2017-ESCC_19	0	0	0.288958527	0	0.010131134	0.048366589	0.147366883	0.066701603	0.415615964	0.017461212	0	0.005398088
nc2017-ESCC_35	0.123845682	0.098512234	1.71E-59	0	0.03127232	0.092663834	0.034512115	0.11848558	0.24176755	0	0.178311663	0.080629023
nc2017-ESCC_50	0	0.207585115	0.067629691	0	0	2.82E-171	0.098552495	0.031872549	0.572210444	0	0.022149707	1.84E-13
nc2017-ESCC_234	0	0.108841943	0.107180093	0.013148564	0	0.036877365	0.062613887	0.370374135	0.280734352	6.14E-57	0.020229296	3.66E-07
nc2017-ESCC_138	0	0	2.66E-258	2.29E-138	0.034631732	9.98E-62	0.05566469	0	0.48775541	1.09E-212	0	0.421948168
nc2017-ESCC_162	0	0	0.134223472	0.062758361	0	0	0.014542235	0.020641231	0.68413248	0	0	0.083702221
nc2017-ESCC_173	4.91E-96	0.022469266	4.545403941<br>73947e-322	0	2.55E-121	0.121622981	0.147550801	0	0.708356952	0	0	8.80E-80
nc2017-ESCC_178	0	0.228488834	0.004496075	0	0.07427112	0.050945831	0.105663132	0.261582517	0.133319781	0	0.10304678	0.038185928
nc2017-ESCC_199	1.94E-44	0.014941324	0.199590732	0	0.02585783	0.018696625	0.037513866	0.085879012	0.26730082	0.022136182	0.252801901	0.075281707
nc2017-ESCC_225	0	0.285011921	0.223837351	0.018389965	0.052620478	0	0	0.026079662	0.137628595	0	0.002144219	0.25428781
nc2017-ESCC_249	8.22E-85	0.067157299	0.023274036	0	0.011617369	0.021984004	0.147611594	1.05E-61	0.457097028	1.01E-36	0.27125867	4.39E-36
nc2017-ESCC_210	0	0.094722885	6.64E-51	0	0	0.023585471	0.078462375	0.003107414	0.264824109	0.040453336	0.090318355	0.404526055
nc2017-ESCC_132	0.362685427	0.094948849	0.017070395	0	0.008621637	0.059728634	0.024442387	0.148119132	0.179714543	0	3.81E-160	0.104668996
nc2017-ESCC_58	1.28E-17	5.29E-112	1.90E-182	2.81E-40	0	0	0.050580369	6.00E-80	0.395944716	0	0	0.553474914
nc2017-ESCC_16	0	0	0.00190018	0.139678144	0.018116832	0.067105388	0.254117766	0.265340374	0.241470834	0	0	0.012270481
nc2017-ESCC_24	7.58E-135	0.220234022	0.01919913	0	0.01236345	0	0.09954572	0.308094686	0.249081002	0.029237425	0.025673719	0.036570847
nc2017-ESCC_182	0.075521337	0.296456042	1.96E-117	0	0	0	0.032814975	0.279920702	0.278983745	0	1.69E-181	0.036303199
nc2017-ESCC_21	0.445827809	0.187662272	0.117526256	0	0.248983661	0	0	2.11E-45	0	0	0	2.12E-09
nc2017-ESCC_E34	0	0.054822067	0.058078138	0	5.434722104<br>25371e-322	0.07422121	0.210001812	0	0.498245691	0	0.104631082	0
nc2017-ESCC_220	0	0.03947832	0	0.077275477	0	0.092071845	0.151777964	0.101497089	0.371318901	0.157925643	0	0.008654762
nc2017-ESCC_201	0.249782258	0	0.090121702	0	0	0.14926579	0.176006782	0	0.07838229	0.021609708	0.122658566	0.112172904
nc2017-ESCC_E11	0.102785592	0	1.04E-145	0	0	2.95E-114	0.459118505	0.114784468	0.063282718	0	0.051894136	0.208134581
nc2017-ESCC_27	0	0	2.95E-93	0	0	0.231840737	0.122874752	0.355683924	0.18804918	5.48E-65	4.30E-87	0.101551407

nc2017-ESCC_E47	0	0.054877957	0.028810656	0.014688036	0.091924737	0	0.009178549	0.077280872	0.429174108	0.070388722	0.085951545	0.137724819
nc2017-ESCC_145	0	2.96E-08	0	0.147602798	0.009974975	0.023057298	0.003334296	0.064920529	0.31770354	0.027065746	0.375277468	0.03106332
nc2017-ESCC_E75	7.66E-107	0	0	1.39E-63	0	0.016317547	0.286855237	1.34E-49	0.381471045	0	0	0.315356172
nc2017-ESCC_E30	0	0.179411019	5.13E-224	0	0	0	0.057657481	0.470401398	0.071785217	0.086169924	0.13457496	0
nc2017-ESCC_223	0.106602859	0.217614843	0	0.016895662	0.034596459	0.039834809	0.105213675	0.136405	0.246661546	0	0.058670433	0.037504714
nc2017-ESCC_36	6.98E-33	1.84E-101	0.110327688	0.071386955	0	0.046455543	0.076558919	0.177785286	0.330316801	0	0	0.187168807
nc2017-ESCC_E74	0	0	0.245723436	0.021080006	0	1.27E-307	0.081112197	0	0.220059827	0	0.018351469	0.413673065
nc2017-ESCC_149	0.072902159	0.546137979	0	9.70E-29	0	0.005103516	0.107768115	0.111040943	0	0	0	0.157047287
nc2017-ESCC_206	3.63E-145	3.71E-11	0.096318014	0.049217487	0	0.166517633	0	0	0.426412075	0	0.029584354	0.231950438
nc2017-ESCC_E50	0	0	0.418372178	0	0	7.26276499386632e-322	0	0.417533446	0.164094376	0	0	0
nc2017-ESCC_E78	0	0	5.12E-244	0	0	2.85E-20	0.005660272	7.23E-32	0.994339728	0	0	0
nc2017-ESCC_E71	0.809571481	0	0	0	0.007835235	0	0	0.092096459	0	0.014198494	0	0.076298332
nc2017-ESCC_246	0	0	0	0	0.028402021	0.016194768	0.515704837	5.62E-212	0.439698373	5.24E-35	2.10E-137	0
nc2017-ESCC_60	0	0	0	0.631364119	0	0	0	0.207998532	0	0	0.115526656	0.045110693
nc2017-ESCC_235	0.249827869	0	0.033548927	0	0	0	0	1.53E-93	0	0	0.716623204	0
ng2014-Genomicandmolecular-ESCC-D1	0.124490222	0.206365028	0.187278891	0.03585157	5.25E-95	0.16674348	0.068394384	5.07E-06	0.155552486	0	0.012556503	0.042762368
ng2014-Genomicandmolecular-ESCC-D2	0.136336379	0.054400567	0.041837114	0.019129047	0.062990875	0.13476649	0.100396784	0.025916631	0.396738781	0.019120994	0	0.00836634
ng2014-Genomicandmolecular-ESCC-D3	0.199947623	0.195392214	0.040564888	0.001850625	0	1.66E-60	0	0.220617702	0.303877171	0	0	0.037749778
ng2014-Genomicandmolecular-ESCC-D4	0.171003113	0.020448988	0.063873148	0	0	0.267716988	0.196902777	0.126710686	0.1533443	0	0	6.94E-36
ng2014-Genomican	3.69E-27	0	0.076012086	0	0.015697688	0.048483394	0.044119046	0.004602627	0.468268711	0.118875491	0.001507089	0.222433868

dmolecular-ESCC-D5												
ng2014-Genomicandmolecular-ESCC-D7	0.05530049	0	0.178428539	0	0	0.174655589	0.170919691	0	0.235032285	0	0.078381049	0.107282357
ng2014-Genomicandmolecular-ESCC-D8	0.163395866	0	0.100559015	0.033732151	0	0.057451957	0.047770624	0.073013106	0.44228373	0	0.011101555	0.070691996
ng2014-Genomicandmolecular-ESCC-D9	0.126916324	0.208014676	0.011848493	0	0	0.13237339	0.053625561	8.71E-05	0.422782676	0.017039213	0.027312608	5.77E-19
ng2014-Genomicandmolecular-ESCC-D10	0	0.054021274	0.042786575	1.78E-32	0	0.092772159	0.195658446	0.060676357	0.396918907	0.056986183	0.036915968	0.06326413
ng2014-Genomicandmolecular-ESCC-D11	0.092319961	0.122824394	0.015888649	0	0.010991757	0	0.006750762	0.346241252	0.404983226	1.53E-107	0	7.52E-69
ng2014-Genomicandmolecular-ESCC-D12	0	3.57E-59	3.18E-251	0	0.311655024	0	0	0.346958033	0.341386943	0	0	0
ng2014-Genomicandmolecular-ESCC-D13	0	1.50E-134	0	0	0.021591042	0.390944758	0.141612341	0.239845424	0.150907428	0.013725155	0.041373852	0
ng2014-Genomicandmolecular-ESCC-D14	2.07E-34	0.091974447	0.099513737	0.056852082	0	0.152485937	0.050058429	3.51E-13	0.415848952	0	0	0.133266416
ng2014-Genomicandmolecular-ESCC-D15	0.099644527	0.007905262	0.037145899	0.008256347	0.030941444	0.217842407	0.055556851	0.194448197	0.262838942	0.047503411	0.037916711	2.54E-97
ng2014-Genomican	0.03160876	0	0.03393884	0.079910289	0.03922835	0.220758751	0.148515568	1.77E-29	0.328417328	0	0	0.117622114

dmolecular-ESCC-D16												
ng2014-Genomicandmolecular-ESCC-D17	4.81E-19	0.019751238	0.054086983	0.035684514	0.07632301	0.075817271	0	0.182736591	0.279937686	0	0.048493306	0.227169401
ng2014-Genomicandmolecular-ESCC-D18	0.047613258	0.002850234	0.088220914	0	0	0.016982766	0.125963758	0.145326377	0.361959757	0.099167836	0.068855206	0.043059895
ng2014-Genomicandmolecular-ESCC-D19	0.912119067	0	0	0	0.003910319	0	0	0.062790295	0	0	0.021180319	0
ng2014-Genomicandmolecular-ESCC-D20	0.221547704	0.073619253	0.138020449	0.018277627	0.029150833	0.268652038	0.109594872	0.027428235	0.106118031	0.007590957	0	0
ng2014-Genomicandmolecular-ESCC-D21	1.89E-21	0	0.033838313	0	0	0.266177916	0.316780344	0.097690815	0.243834048	0	0.010528636	0.031149928
ng2014-Genomicandmolecular-ESCC-F1	0.148219732	0	0.053147835	0	0.069739578	0.191294795	0.108280424	0.051680377	0.213128354	0	0.164508906	0
ng2014-Genomicandmolecular-ESCC-F2	0	0	0.176785703	0.058154997	0	0.214936597	0.080702015	0.03566275	0.346671402	0	0.037842115	0.04924442
ng2014-Genomicandmolecular-ESCC-F3	0.5685719	0.065651611	2.76E-192	0	2.09E-246	0.123651763	0.139133906	0	4.94065645841247e-322	0.066678259	0.036312561	0
ng2014-Genomicandmolecular-ESCC-F4	0.166146078	0	0.231395058	0.010810319	0.295009608	0.044514721	0.156198886	0	0.09592533	0	0	0
ng2014-Genomican	0	0.285489261	0.108633487	0	0.021893296	0	0	0.331509639	0.223012172	0	0.029462145	0

dmolecular-ESCC-F5												
ng2014-Genomicandmolecular-ESCC-F6	0.13710521	0	0	0	4.88E-18	0.471311941	0.391582849	6.8181059126092e-322	0	7.25E-32	1.57E-18	0
ng2014-Genomicandmolecular-ESCC-F7	0.261034781	0	0	0	0	0.285434658	0.234026935	5.21E-91	0	0.013147961	0.166218237	0.040137428
ng2014-Genomicandmolecular-ESCC-F8	0	0.432507822	0.230021821	0	0	0	0	0.268428499	0	9.30E-199	0.069041858	0
ng2014-Genomicandmolecular-ESCC-F9	2.80E-15	0.029119743	0.386578569	0	0	0	0	0.413117857	0.091220641	0	0	0.079963189
ng2014-Genomicandmolecular-ESCC-F10	2.48E-154	0	0.164272996	2.78E-74	0	0.315975722	0.015058349	0.273900892	0.053779562	0.030433684	0.015050337	0.131528458
ng2014-Genomicandmolecular-ESCC-F11	6.53E-181	0	0.40216488	2.46E-153	0	0.005950188	0.02877591	4.27E-282	0.21842503	4.19E-136	0	0.344683992
ng2014-Genomicandmolecular-ESCC-F12	0	0	0.074907893	0.028457357	0	0.127941129	0.098586474	0.555698882	0	0	0.08467047	0.029737794
ng2014-Genomicandmolecular-ESCC-F13	0.176958134	0.023275015	0.045975374	0	0.139842256	0.12073043	0.120611656	0	0	0	0.040772635	0.3318345
ng2014-Genomicandmolecular-ESCC-F14	0.07816194	0.200053063	0.090172539	0	0	0.230766865	2.22329540628561e-322	0.035137498	0.292895443	0.072812652	1.58E-229	1.41E-178
ng2014-Genomican	0	0	0	0	0	0.511392412	0.079517115	0.0099955	0.395360928	0.003734044	0	0

dmolecular-ESCC-F15												
ng2014-Genomicandmolecular-ESCC-F16	0	0.024391971	0.45440172	0	0	0.111418306	0.162331345	9.44E-56	0.237282402	6.63E-35	0	0.010174258
ng2014-Genomicandmolecular-ESCC-F17	2.16E-21	0.302089916	0	0.074237026	0.007432762	0.052627907	0.16522691	0.02279686	0	0	0	0.37558862
ng2014-Genomicandmolecular-ESCC-F18	0	0.100070559	1.88E-11	0	0	0	0	1.67E-12	0.643603271	0	0.102760231	0.153565939
ng2014-Genomicandmolecular-ESCC-F19	0.193735138	0.203611695	0.110940815	0	0	0.26051233	0	0.005088946	0.226111077	0	2.25E-22	0
ng2014-Genomicandmolecular-ESCC-F20	0.254144668	0.072527209	0.096049528	0	0	0.068668615	0	0	0.347463615	0.012302444	0.148843922	1.81E-123
ng2014-Genomicandmolecular-ESCC-F21	0.195310721	0.468366116	0.087192672	4.15015142506647e-322	0	0.201876152	0.04725434	5.88E-104	4.00E-66	0	0	0
ng2014-Genomicandmolecular-ESCC-F22	0	0	0.493255278	0	0	0.012213534	0.218305688	0.14371321	0	0	0	0.13251229
ng2014-Genomicandmolecular-ESCC-F23	0	0	0	0.016430862	0	0	0.070896429	0.637572975	0.275099734	0	0	0
ng2014-Genomicandmolecular-ESCC-F24	1.76E-86	0	0	0.143787701	0	0	0	0.544807496	0	0	0.311404802	0
ng2014-Genomican	0	0.576321286	0.005748189	0	0	0	0.001847047	0.416083478	0	0	1.20E-28	0

dmolecular-ESCC-F25												
ng2014-Genomicandmolecular-ESCC-F26	0	0.084944414	0.234579436	0	0	0	0.072760985	0	3.05E-06	0.090989825	0.01726846	0.499453827
ng2014-Genomicandmolecular-ESCC-F27	0	0	0	0	0	0.683409028	0.069122546	0.09113321	0.055011615	4.34E-14	0.101323601	0
ng2014-Genomicandmolecular-ESCC-F28	0.065599195	1.18E-163	0.247507343	1.58E-07	0	0.067894768	0	0	0.618998535	0	0	0
ng2014-Genomicandmolecular-ESCC-F29	0.062551404	0	0	0	0	0.350900981	0	0.217723795	0	0.10992351	0.208494876	0.050405433
ng2014-Genomicandmolecular-ESCC-F30	0	0	0	0	0.05480586	8.88E-14	0.218285051	0.448433107	0.278475981	0	0	0
ng2014-Genomicandmolecular-ESCC-F31	1.86E-100	0.323693424	0	1.86E-55	0	0.054440364	0.161483865	0	0.334256885	0	0.005338693	0.120786769
ng2014-Genomicandmolecular-ESCC-F32	0.15114177	0	0.116506307	0	9.98E-28	0.162093823	0	0.270344948	0.183585396	0	0	0.116327755
ng2014-Genomicandmolecular-ESCC-F33	0.65591174	0	0.34408826	2.27E-34	0	0	0	0	0	0	0	0
ng2014-Genomicandmolecular-ESCC-F34	0.003980707	0.222613858	0	0	6.85E-53	0.208927406	0.307482777	7.52E-61	0.15755024	6.39E-112	0.012658955	0.086786056
ng2014-Genomican	1.90E-59	6.17E-05	0.289265824	0.035419385	0	0.102608906	0.142753025	0	0.315147052	0.01594999	0	0.09879415

dmolecular-ESCC-F35												
ng2014-Genomicandmolecular-ESCC-F36	0	0.017533576	0.360762313	0	0	0.100780319	0.075694602	0	0.286813579	0	0	0.158415611
ng2014-Genomicandmolecular-ESCC-F37	4.58E-21	0.360860851	0.053313578	0.339891569	0	0.063390041	0.182543961	0	3.95252516672997e-322	0	1.48E-261	0
ng2014-Genomicandmolecular-ESCC-F38	0.252351877	0.161011554	0.06807457	0.115266664	0.011667374	0.287714763	0	1.70E-17	0.093487235	0.010425964	0	1.07E-61
ng2014-Genomicandmolecular-ESCC-F39	1.95E-106	0.878511117	3.86E-55	0	0	0	0.090632152	2.92E-22	0	0.030856731	5.78056805634258e-322	0
ng2014-Genomicandmolecular-ESCC-F40	1.69E-13	0	0.245531036	0.01447369	5.73E-12	0.367981247	0.232938369	7.21E-59	0.038674011	0.100401646	0	2.77E-17
ng2014-Genomicandmolecular-ESCC-F41	0.050592087	0.260181194	0.063689949	0.023407883	0	0.250549109	0.113746901	0	0.155828085	0	6.16E-100	0.082004792
ng2014-Genomicandmolecular-ESCC-F42	2.53E-38	0.195345641	9.75E-20	0.04556	0	0.207639849	0.043536118	0	0.203097934	0	0.245969761	0.058850698
ng2014-Genomicandmolecular-ESCC-F43	1.15E-104	0.629519177	0.072979846	0	0	0.287929499	0	4.38E-55	0	0	0	0.009571479
ng2014-Genomicandmolecular-ESCC-F44	1.04E-130	0.322601374	0.088927329	9.64910206327955e-321	0	0.537612975	0.050858322	0	0	0	0	0
ng2014-Genomican	0	0.014309593	0.226183884	0.09545087	0	0.050503881	0.032534955	0.392504139	0.188512678	0	0	1.95E-104

dmolecular-ESCC-F45												
ng2014-Genomicandmolecular-ESCC-F46	1.22E-183	0	0.213935742	1.70E-12	0.000906527	0.252716219	0.082940554	1.96E-36	0.300233488	0	0.047584398	0.101683073
ng2014-Genomicandmolecular-ESCC-F47	0.072513197	0.14256445	0.331260667	0	0.068574558	0	0	0.062736223	0.142453228	0.179897678	0	0
ng2014-Genomicandmolecular-ESCC-F48	0	0.026366486	0.33398639	0	0	1.99E-96	0.203951431	1.59E-262	0.249507761	0	0.046236285	0.139951648
ng2014-Genomicandmolecular-ESCC-F49	3.2114266979681e-322	0.088594233	0.899852296	0	0	0	0	0	4.09E-132	0.011553471	0	0
ng2014-Genomicandmolecular-ESCC-F50	0	0.198681302	0.007645469	0.040058267	0	0.463980259	0.115687876	8.07E-52	0.153546949	0	0	0.020399878
ng2014-Genomicandmolecular-ESCC-F51	1.48E-75	0	1.84E-89	0	0.096045791	0.406056054	0.014852724	0.47352539	0	0	0	0.00952004
ng2014-Genomicandmolecular-ESCC-F52	0	0.523490652	0	0.081747417	0	0.201072444	0.033501123	0	0.160188364	0	0	0
ng2014-Genomicandmolecular-ESCC-F53	0	0	5.48E-34	8.25E-14	0	0	0.009345539	0.569251042	0.42140342	5.36E-226	0	0
ng2014-Genomicandmolecular-ESCC-F54	0.183536862	0	0	0	0	0	0	0	0.540238701	0	0	0.276224437
ng2014-Genomican	0	0	0.25923643	0.127470824	0	0.152164527	0.14284687	0.056594412	0	0.066796856	0.012675804	0.182214277

dmolecular-ESCC-F55												
ng2014-Genomicandmolecular-ESCC-F56	0	0	0	0	0.151876524	0	3.29E-263	0.469003606	0.252174325	0	0	0.126945545
ng2014-Genomicandmolecular-ESCC-F57	1.56E-72	0.048636781	0.135694766	0.007533582	0	0	0.034884932	0.013764239	0.7594857	0	0	0
ng2014-Genomicandmolecular-ESCC-F58	0.05023217	0.117324007	0.025538302	0	0.012877153	0.341255321	0.260419316	0.046292755	0.146060976	0	0	0
ng2014-Genomicandmolecular-ESCC-F59	0	0	0	0	0	0.133723073	0.022125601	0.529808048	0.314343278	0	0	0
ng2014-Genomicandmolecular-ESCC-F60	2.01E-09	0	0.076259658	0.0894739	0.026704898	0.00567364	0	0.316204158	0.231103932	0.030226055	0.011425269	0.212928488
ng2014-Genomicandmolecular-ESCC-F61	0.248937642	0	0	0	0	0	0	0.345410168	0	0.082248136	0	0.323404054
ng2014-Genomicandmolecular-ESCC-F62	0	0	0.168277292	0	0.246170888	6.05E-110	0.166897981	0.418653838	0	0	7.85564376887582e-322	3.66E-42
ng2014-Genomicandmolecular-ESCC-F63	0.068180686	0	0	0	0.056572738	0.280097907	0	0.19665366	0	0	0	0.398495009
ng2014-Genomicandmolecular-ESCC-F64	2.22E-42	6.46E-236	0	0	0.266701354	0	0.454600452	0	0.194210559	0	0.084487634	0
ng2014-Genomican	6.98E-82	0.614098867	0	1.69E-10	0.014453827	0.017217825	0	0.198409718	0.155819763	7.58E-15	0	0

dmolecular-ESCC-F66												
ng2014-Genomicandmolecular-ESCC-F67	0.361188457	0	0.201710126	0.054712276	0	9.69E-122	0.270174269	4.68E-96	0.006109969	0.099703105	0	0.006401798
ng2014-Genomicandmolecular-ESCC-F68	2.27E-125	0.023389556	0	0	0	0	0.062488092	0	0.842792143	0.071330209	7.32E-91	0
ng2014-Genomicandmolecular-ESCC-F69	0	0	0.336367128	0.174912961	0	0.103467335	0	0.383904774	0	0.001347802	0	0
ng2014-Genomicandmolecular-ESCC-F70	0	6.68E-142	0.242388394	0.303925955	3.68E-283	0	0.36735971	0	0	0	0.086325941	0
ng2014-Genomicandmolecular-ESCC-F71	8.98E-47	0.322519384	0.072060008	0.017065249	0	0.294793265	0.18143129	0	0.112130804	0	0	0
ng2014-Genomicandmolecular-ESCC-F72	0	0.326690665	0.138040864	0.065821558	0	0.175956773	0	0.146497236	0.146992904	0	0	1.96E-120
ng2014-Genomicandmolecular-ESCC-F73	6.31E-71	0.157326792	0.193338504	2.80E-25	0	0	0.304959138	0.150980159	0	0.193395408	0	0
ng2014-Genomicandmolecular-ESCC-F74	1.62E-27	0.496504104	0.273076543	0	0	0.168558905	0	0	0.061860448	0	4.15E-39	1.07E-199
ng2014-Genomicandmolecular-ESCC-F75	0.000376716	0	0.081018145	6.64E-101	1.22E-43	0.332768599	0.191471523	0	0.074646734	0.072510509	0.077137573	0.170070201
ng2014-Genomican	1.05E-59	0	5.72E-59	0.092516239	0	0.318637467	0.248962592	0.158385575	0.181498127	9.96E-19	0	3.03E-22

dmolecular-ESCC-F76												
ng2014-Genomicandmolecular-ESCC-F77	0	0	0	0	0	0.72222297	0.097604771	4.19E-71	0.173868733	0.006303526	0	0
ng2014-Genomicandmolecular-ESCC-F78	0.137768451	0.286600476	0.192154954	0	0	0.134587381	0.074315628	0	0.133519823	0.041053287	0	0
ng2014-Genomicandmolecular-ESCC-F79	2.15E-85	0	0.116879987	0.078715956	0.006781625	0.004696753	0	0.385337199	0.397007293	0.010581187	0	3.20E-101
ng2014-Genomicandmolecular-ESCC-F80	5.30E-28	0	0	0	0	0.362590179	0.080091221	0.041492667	0.515825933	0	0	0
ng2014-Genomicandmolecular-ESCC-F81	0.047027022	0	0	0	0	0.602366413	0.032477329	1.01E-52	0.110439173	0.038419074	0.012279421	0.156991568
ng2014-Genomicandmolecular-ESCC-F82	0	0.010410055	0	2.60E-194	0	0	0.124183458	0	0.281207211	0	0	0.584199276
ng2014-Genomicandmolecular-ESCC-F83	7.39E-12	0.022173472	0.335925473	0	7.09E-210	0.196536814	0.049116122	0	0.39624812	0	0	4.30E-13
ng2014-Genomicandmolecular-ESCC-F84	0	0.005413584	0.104668949	0	2.40E-99	0.23162063	0.409355126	0	0.226945035	0	0.021996676	0
ng2014-Genomicandmolecular-ESCC-F85	0	0.01962917	0.09250373	0.147175366	0	0.297661636	0.094812119	0.160975463	0.187242516	0	0	0
ng2014-Genomican	0.029412831	0.06007349	0.073917495	0	0	0.336439769	0.347813267	8.41E-17	0.132880926	0	0.019462223	0

dmolecular-ESCC-F86												
ng2014-Genomicandmolecular-ESCC-F87	0.003172661	0	0.035256491	0.010953835	0	0.498349619	0.235312115	0.02818788	0.188767399	0	0	8.34E-242
ng2014-Genomicandmolecular-ESCC-F89	3.22E-261	0	0.085284475	0	0	0	0	7.70E-282	0.446485005	0	0.46823052	1.26E-114
ng2014-Genomicandmolecular-ESCC-F90	0	0	0.183837133	0.083397732	0	0.225853005	0.002510656	0.093738447	0.276707619	3.04E-156	0.133955408	2.81617418129511e-322
ng2014-Genomicandmolecular-ESCC-F91	0.151358603	0.001027401	0.146118612	0	6.66988621885683e-322	0.217958103	0.327013703	0	0.156523578	0	0	0
ng2014-Genomicandmolecular-ESCC-F92	0	0	0	0	0	0.28003155	0.476540012	7.38E-42	0.212675773	0.030752665	1.26E-126	6.94E-142
ng2014-Genomicandmolecular-ESCC-F93	7.43E-21	0	0.152560967	0.044322764	0	0.273885848	0.198880037	0.161160777	0	0.08947063	0.005141548	0.07457743
ng2014-Genomicandmolecular-ESCC-F94	0.373294242	0	0	0	0.011080482	0	0	0.284554175	0.3310711	0	0	0
ng2014-Genomicandmolecular-ESCC-F95	1.09E-80	0.067105669	0.34967065	0	0	0.132597599	0	0	0.404695209	0.045930873	0	0
ng2014-Genomicandmolecular-ESCC-F96	0	0.143873752	0	0.12766874	0	0.079835684	0	0.300337216	0.052714936	0	0	0.295569671
ng2014-Genomican	2.16E-29	0	0.128405203	0.01881964	3.41E-105	0.219878228	0	0.161688604	0.471208325	0	0	0

dmolecular-ESCC-F97												
ng2014-Genomicandmolecular-ESCC-F98	1.08E-300	0	2.84E-276	0	0	0.176706281	0	3.22E-18	0.823293719	0	0	0
ng2014-Genomicandmolecular-ESCC-F99	0	0	0.42268903	0.185053285	0	0.038490407	0.22318575	6.90E-65	0	0	0.020686795	0.109894732
ng2014-Genomicandmolecular-ESCC-F100	0.294549741	0	0.23921766	0	0	4.42E-232	0	0	0.424288133	0.041944466	7.85564376887582e-322	3.07E-101
ng2014-Genomicandmolecular-ESCC-F101	0	0.180875165	0.325937915	0	0	0.14654806	0.332000114	0	0.014638746	0	8.07E-256	0
ng2014-Genomicandmolecular-ESCC-F102	0.027782371	0.274234676	0.002977203	0	0.056798012	0.223913149	0	0.032042834	0.382251755	0	0	1.14E-187
ng2014-Genomicandmolecular-ESCC-F103	0.347088639	0.125437958	2.87E-10	0	0	0.153781947	0.125068972	0.20826961	0	0	0.040352873	0
ng2014-Genomicandmolecular-ESCC-F104	0	0	0.110048213	0	2.62E-161	0.389037599	0.275134743	0	0.161011504	0	1.08E-189	0.064767941
ng2014-Genomicandmolecular-ESCC-F105	0	0	0.148002502	0.139427201	0	0.3498986	0	0.362671697	0	7.44E-60	0	0
ng2014-Genomicandmolecular-ESCC-F106	0	0	0.122089554	0.163731467	0.118233824	0	0.075439784	1.04E-43	0.308492026	0.212013344	0	0
ng2014-Genomican	0	0	0.230488663	0	2.36E-294	0.014289196	0.40488304	0	1.18E-165	8.60E-08	4.41E-242	0.350339016

dmolecular-ESCC-F107												
ng2014-Genomicandmolecular-ESCC-F108	0	7.05E-26	0.214073916	0	0.121812726	7.90E-112	0.013181189	0.185324852	0.465607317	0	0	0
ng2014-Genomicandmolecular-ESCC-F109	4.12E-256	6.92E-22	0.059552985	2.11E-129	8.78E-173	0.181413827	0	0	0.683220874	0	0	0.075812314
ng2014-Genomicandmolecular-ESCC-F110	0	2.07E-63	0.166491344	0	0.051236511	0.176731136	0	0.528956493	0.068312164	0.008272352	0	0
ng2014-Genomicandmolecular-ESCC-F111	0.031835632	0	0.229953082	0	0	0.071164596	0.118524086	9.11E-29	0.225996475	0.021644656	0.14314457	0.157736903
ng2014-Genomicandmolecular-ESCC-F113	0.062895059	0	0.100105925	0	0	0.461476843	0.04213789	6.73E-28	0.123700812	0.052121299	0.097755004	0.059807168
ng2014-Genomicandmolecular-ESCC-F114	0	0.164794595	0.032622053	0.076062178	0	0.126331018	0.043280853	1.99E-21	0.324088945	0	0	0.232820359
ng2014-Genomicandmolecular-ESCC-F115	3.78E-32	0.114116502	0.389428935	0	0	0	0.026867177	2.38E-27	0.469587386	0	0	0
ng2014-Genomicandmolecular-ESCC-F116	0	0.139354375	0.494405383	0	0.116780037	0	0	0.249460204	0	0	1.54E-50	1.10E-47
ng2014-Genomicandmolecular-ESCC-F117	0.420768315	0.000260689	0	0	0.024896943	0	0	0.291653281	0	0	0.016775726	0.245645046
ng2014-Genomican	0	0	1.30E-161	0	9.79E-88	0.224523882	0.296346707	1.35E-109	0.479129412	0	1.60E-267	0

dmolecular-ESCC-F118												
ng2014-Genomicandmolecular-ESCC-F119	9.97E-171	0	0	0	0	8.22E-10	0	0	0.999999999	0	0	0
ng2014-Genomicandmolecular-ESCC-F120	0	0	0	0	0.011093585	0.058352497	0.000155886	0.115228248	0.179980733	0	0.079329913	0.555859138
