# Supplementary material for: A practical framework RNMF for exploring the association between mutational signatures and genes using gene cumulative contribution abundance
Source: Cancer Med. 2022 May 16;11(21):4053–69. doi: 10.1002/cam4.4717 (PMC9636515; doi:10.1002/cam4.4717)
Supplement: Supplementary file 14 — Table S5 [file CAM4-11-4053-s004.pdf]

Table S5. Cancer-related genes from the COSMIC census.

Hugo_Symbol	Chromosome	Length	Hugo_Symbol	Chromosome	Length	Hugo_Symbol	Chromosome	Length
KDSR	chr18	39784	LMO2	chr11	33713	HNF1A	chr12	22520
ATIC	chr2	37946	LYL1	chr19	3834	PTCH1	chr9	65681
AKAP9	chr7	169806	LCK	chr1	34926	IKZF1	chr7	100432
ACSL3	chr2	83705	KDM5A	chr12	109325	ID3	chr1	1876
ACSL6	chr5	61689	KMT2A	chr11	90342	IGF2BP2	chr3	181037
AFF1	chr4	206052	KAT7	chr17	46475	KAT6B	chr10	205997
ALDH2	chr12	50607	MAML2	chr11	366582	KEAP1	chr19	17621
ASPSCR1	chr17	39854	MTCP1	chrX	7192	KLF6	chr10	9279
BCL7A	chr12	42622	MDM2	chr12	42510	LARP4B	chr10	124791
BCR	chr22	137827	MDM4	chr1	41737	LATS1	chr6	60103
CANT1	chr17	18100	MECOM	chr3	62806	LATS2	chr13	88515
CLIP1	chr12	151135	MTOR	chr1	155972	LRP1B	chr2	1900278
CNTRL	chr9	102596	MITF	chr3	228901	LEPROTL1	chr8	42868
CEP89	chr19	96038	MET	chr7	123937	LRIG3	chr12	48373
C15orf65	chr15	10164	MACC1	chr7	82735	LZTR1	chr22	17025
WDCP	chr2	18013	MAPK1	chr22	113129	KDM5C	chrX	33270
CLP1	chr11	4710	MAP2K1	chr15	105495	KMT2C	chr7	301080
CHCHD7	chr8	6859	MAP2K2	chr19	33808	MED12	chrX	23724
COL3A1	chr2	38426	MN1	chr22	53221	MEN1	chr11	7770
COL1A1	chr17	18343	MUC16	chr19	132498	EXT1	chr8	317363
COL2A1	chr12	31521	MUC4	chr3	65512	EXT2	chr11	149232
CRTC3	chr15	112745	MALT1	chr18	83091	MSH2	chr2	80259
CHIC2	chr4	54901	MSI2	chr17	428151	MSH6	chr2	27019
COX6C	chr8	16196	MYD88	chr3	4541	MUTYH	chr1	11146
DNAJB1	chr19	3650	MLLT10	chr10	209287	MAX	chr14	26926
DUX4L1	chr4	1274	AFDN	chr6	142597	MLF1	chr3	23624
DCTN1	chr2	19201	MPL	chr1	14923	MYH9	chr22	106790
EML4	chr2	163198	MYOD1	chr11	2563	N4BP2	chr4	101426
ELN	chr7	42118	NRAS	chr1	12425	NRG1	chr8	215772
ERC1	chr12	468177	NTRK3	chr15	379974	NF1	chr17	282750
EIF4A2	chr3	6353	NFATC2	chr20	155762	NF2	chr22	95040
EZR	chr6	53664	NCOA2	chr8	294043	NAB2	chr12	6582
FAM131B	chr7	9352	NR4A3	chr9	45036	NBN	chr8	51380
FGFR1OP	chr6	53531	NPM1	chr5	23489	NDRG1	chr8	60410
FIP1L1	chr4	82023	NUP98	chr11	122782	NTHL1	chr16	8051
FNBP1	chr9	155975	NUTM1	chr15	14413	NFKBIE	chr6	7622
GPHN	chr14	674396	OLIG2	chr21	3261	NCOA4	chr10	24195
GOPC	chr6	42095	PAX3	chr2	99093	NCOR1	chr17	186392
GOLGA5	chr14	45732	PSIP1	chr9	46953	NCOR2	chr12	170837
GAS7	chr17	284701	PREX2	chr8	284912	MGMT	chr10	300823
GMPS	chr3	73490	PIK3CB	chr3	106660	PHOX2B	chr4	4888

HSP90AA1	chr14	58637	PIK3CA	chr3	91570	PALB2	chr16	38140
HSP90AB1	chr6	6017	PLCG1	chr20	38761	PRF1	chr10	5426
HMGN2P46	chr15	45594	PIM1	chr6	5223	PER1	chr17	11963
HIST1H4I	chr6	1293	PDGFB	chr22	21392	PPARG	chr3	82884
HERPUD1	chr16	12815	PDGFRB	chr5	42023	PHF6	chrX	55477
HOOK3	chr8	133607	PDGFRA	chr4	69150	PTEN	chr10	108817
ITK	chr5	74365	PLAG1	chr8	50375	PIK3R1	chr5	86101
IL2	chr4	5255	KCNJ5	chr11	29679	PMS2	chr7	35886
IL21R	chr16	49880	POU2AF1	chr11	27440	PBRM1	chr3	137995
JAZF1	chr7	350170	POU5F1	chr6	6356	POLE	chr12	63597
KLK2	chr19	7318	PRDM16	chr1	369410	PRDM1	chr6	23618
KIAA1549	chr7	143793	PBX1	chr1	292196	PRDM2	chr1	120224
SHTN1	chr10	122200	PDCD1LG2	chr9	60684	PML	chr15	52054
KTN1	chr14	104262	PRKACA	chr19	26056	POT1	chr7	107595
KIF5B	chr10	47421	PPM1D	chr17	66097	PPP2R1A	chr19	39494
LMNA	chr1	25382	PTPN11	chr12	85652	PPP6C	chr9	43365
LIFR	chr5	120441	P2RY8	chrX	74535	PTPN13	chr4	220856
LASP1	chr17	51911	RAP1GDS1	chr4	180816	PTPN6	chr12	10045
LHFPL6	chr13	260279	RAC1	chr7	27981	PTPRD	chr9	1719544
LSM14A	chr19	56949	RET	chr10	53324	PTPRT	chr20	1117217
LCP1	chr13	85428	RARA	chr17	47602	PTPRB	chr12	121574
HLA-A	chr6	4624	ARHGAP5	chr14	82439	PTPRC	chr1	118469
MSN	chrX	74261	RSPO3	chr6	78692	PTPRK	chr6	551499
MNX1	chr7	5798	STIL	chr1	63998	RAD17	chr5	44995
MUC1	chr1	7089	SRSF2	chr17	3218	RAD51B	chr14	791786
MDS2	chr1	59073	SRSF3	chr6	11232	RANBP2	chr2	66330
MLLT1	chr19	69569	SGK1	chr6	148812	RHOH	chr4	50060
MLLT11	chr1	10739	SETBP1	chr18	387612	RMI2	chr16	6331
MLLT3	chr9	280878	SETDB1	chr1	38396	RB1	chr13	178164
MLLT6	chr17	24261	SET	chr9	12745	ARHGAP26	chr5	458634
MYO5A	chr15	221767	SH3GL1	chr19	40177	ARHGEF12	chr11	152858
MYH11	chr16	153839	STAT3	chr17	75244	ARHGEF10	chr8	134626
NACA	chr12	12866	STAT6	chr12	16025	ARHGEF10L	chr1	158034
NCKIPSD	chr3	12083	SIX1	chr14	6047	RPL10	chrX	4109
NIN	chr14	105281	SIX2	chr2	4269	RPL22	chr1	14592
NONO	chrX	17585	SKI	chr1	81424	RPL5	chr1	9941
NFIB	chr9	226098	SMO	chr7	24673	RFWD3	chr16	45487
NUMA1	chr11	77823	SALL4	chr20	20189	RNF43	chr17	63857
NSD1	chr5	166290	SYK	chr9	96762	RBM10	chrX	41365
NCOA1	chr2	278770	SF3B1	chr2	45307	ROBO2	chr3	1740815
NUP214	chr9	109109	SRC	chr20	59896	RSPO2	chr8	184331
NUTM2D	chr10	9527	SOX2	chr3	2512	SFRP4	chr7	10983
NUTM2B	chr10	9530	SND1	chr7	440427	STK11	chr19	21971

OMD	chr9	10216	SSX1	chrX	12127	SETD1B	chr12	26059
PAX7	chr1	105132	SSX2	chrX	10265	SETD2	chr3	147278
PAX8	chr2	62924	SSX4	chrX	9820	SH2B3	chr12	45675
PRRX1	chr1	75513	TAF15	chr17	37787	SBDS	chr7	7924
PRCC	chr1	33317	TAL1	chr1	13479	SIRPA	chr20	46084
PCM1	chr8	105128	TAL2	chr9	655	SMAD2	chr18	121602
PICALM	chr11	110115	TCL1A	chr14	4228	SMAD3	chr15	129350
PDE4DIP	chr1	143553	TLX1	chr10	7284	SMAD4	chr18	54826
PAFAH1B2	chr11	26781	TLX3	chr5	2850	SLC34A2	chr4	22904
PPFIBP1	chr12	171358	TEC	chr4	134081	SPOP	chr17	79226
PWWP2A	chr5	28084	TNC	chr9	97723	SPEN	chr1	92596
RABEP1	chr17	103512	TSHR	chr14	191259	SFPQ	chr1	10216
RALGDS	chr9	23456	TCF7L2	chr10	217086	SOX21	chr13	2777
RPN1	chr3	30879	TFE3	chrX	14755	STAG1	chr3	416135
RNF213	chr17	137918	TFEB	chr6	52281	STAG2	chrX	140979
RBM15	chr1	8171	TRRAP	chr7	134753	SMC1A	chrX	48579
SNX29	chr16	597551	TRIM27	chr6	20987	SDHAF2	chr11	17456
S100A7	chr1	2957	TNFRSF17	chr16	2961	SDHA	chr5	38459
SEPT5	chr22	8855	U2AF1	chr21	14631	SDHB	chr1	35448
SEPT6	chrX	75731	UBR5	chr8	159829	SDHC	chr1	61083
SEPT9	chr17	219182	USP6	chr17	58553	SDHD	chr11	9021
SRGAP3	chr3	268788	USP8	chr15	90037	SOCS1	chr16	1783
SLC45A3	chr1	22608	ABL1	chr9	171737	SUFU	chr10	129548
SPECC1	chr17	227733	AKT1	chr14	24775	SMARCA4	chr19	101457
STRN	chr2	122832	AKT2	chr19	55078	SMARCB1	chr22	47542
SDC4	chr20	23136	AKT3	chr1	343562	SMARCD1	chr12	15740
SS18L1	chr20	38689	KDR	chr4	47336	SMARCE1	chr17	23902
SS18	chr18	74372	ERBB2	chr17	28582	TET2	chr4	133523
TFPT	chr19	8662	ERG	chr21	281667	TGFBR2	chr3	87541
THRAP3	chr1	80941	HRAS	chr11	3307	TMEM127	chr2	17478
TRIP11	chr14	74068	KRAS	chr12	41372	TRIM33	chr1	118383
TOP1	chr20	95669	KIT	chr4	82797	TPM3	chr1	24746
TCEA1	chr8	55973	MAF	chr16	6876	TSC1	chr9	53285
TCF12	chr15	369891	MAFB	chr20	3392	TSC2	chr16	41255
TFRC	chr3	32905	MYB	chr6	37852	TNFRSF14	chr1	7463
TPR	chr1	63673	MYCL	chr1	6587	TNFAIP3	chr6	16123
TMPRSS2	chr21	43512	MYC	chr8	5365	TRAF7	chr16	22364
TFG	chr3	39541	MYCN	chr2	6443	YWHAE	chr17	56106
TPM4	chr19	35405	BRAF	chr7	194263	USP44	chr12	34930
VAV1	chr19	84381	RAF1	chr3	80625	VHL	chr3	11212
VTI1A	chr10	371486	REL	chr2	50089	WRN	chr8	139968
ZMYM2	chr13	133159	ROS1	chr6	137555	WNK2	chr9	133341
ZNF384	chr12	22862	WAS	chrX	7648	WIF1	chr12	70940

ZCCHC8	chr12	28174	NSD2	chr4	89362	XPA	chr9	22448
NT5C2	chr10	105288	NSD3	chr8	112575	XPC	chr3	33636
ATF1	chr12	56830	WWTR1	chr3	186038	ZNRF3	chr22	173895
ACVR1	chr2	138667	ZEB1	chr10	210641	ZRSR2	chrX	32788
AFF3	chr2	555007	ZNF521	chr18	290226	ZBTB16	chr11	191083
AFF4	chr5	88255	EP300	chr22	88293	ZFHX3	chr16	1108986
ALK	chr2	728792	ABI1	chr10	114342	ZMYM3	chrX	14516
AR	chrX	186583	ACVR2A	chr2	86307	PATZ1	chr22	20428
A1CF	chr10	86254	ASXL1	chr20	80989	ZNF331	chr19	59245
ARAF	chrX	10820	ASXL2	chr2	38384	ANK1	chr8	243537
ACKR3	chr2	12717	APC	chr5	108351	APOBEC3B	chr22	10375
BIRC6	chr2	261871	ATRX	chrX	281391	ARNT	chr1	67063
BCL11A	chr2	97962	AMER1	chrX	20627	ATP1A1	chr1	30905
BCL2	chr18	196782	ARID1A	chr1	86077	BIRC3	chr11	20239
BCL3	chr19	11497	ARID1B	chr6	433405	BCL11B	chr14	101941
BCL6	chr3	24350	ARID2	chr12	178375	BCL9L	chr11	17029
BCL9	chr1	84804	ATM	chr11	146267	BCLAF1	chr6	32988
BCL2L12	chr19	8773	ATP2B3	chrX	46807	BCORL1	chrX	52895
BRD3	chr9	37712	ATR	chr3	129589	BMP5	chr6	121919
BRD4	chr19	44932	AXIN1	chr16	65219	BMPR1A	chr10	176188
ABL2	chr1	130274	AXIN2	chr17	33084	BTK	chrX	41349
CDH17	chr8	81416	BCL10	chr1	10842	CIC	chr19	11132
CACNA1D	chr3	318684	BTG1	chr12	5619	CBL	chr11	101873
CALR	chr19	5882	BAX	chr19	6869	CBLC	chr19	22765
CREB1	chr2	73539	BCOR	chrX	46155	CTNND1	chr11	57381
CREB3L2	chr7	127078	B2M	chr15	7400	CD209	chr19	7540
CHST11	chr12	305041	BLM	chr15	98838	CREBBP	chr16	155672
CARD11	chr7	137804	BAP1	chr3	9337	CRNKL1	chr20	21678
CTNNB1	chr3	19337	BARD1	chr2	84058	CUX1	chr7	442222
CTNNA2	chr2	997137	BRIP1	chr17	182255	CNBD1	chr8	516414
CTNND2	chr5	932203	BAZ1A	chr14	122910	CDKN1A	chr6	8627
CCR4	chr3	4775	BUB1B	chr15	60110	CYP2C8	chr10	32724
CCR7	chr17	11703	CDH1	chr16	98323	DDB2	chr11	24298
CD28	chr2	32364	CDH10	chr5	157878	DCC	chr18	1195727
CD74	chr5	10599	CDH11	chr16	178445	DCAF12L2	chrX	2598
CD79A	chr19	4252	CAMTA1	chr1	984382	DAXX	chr6	4456
CD79B	chr17	3596	CREB3L1	chr11	43761	POLQ	chr3	114575
CHD4	chr12	37388	KNL1	chr15	70322	ELF4	chrX	45567
CSF1R	chr5	60081	CBLB	chr3	213582	EPAS1	chr2	89301
CSF3R	chr1	17235	CASP3	chr4	21779	EZH2	chr7	76893
CRTC1	chr19	98655	CASP8	chr2	29675	EPHA3	chr3	374610
CXCR4	chr2	1894	CASP9	chr1	32025	EPHA7	chr6	179506
CCND1	chr11	13387	CDX2	chr13	9002	ECT2L	chr6	107959

CCND2	chr12	31578	CEBPA	chr19	2630	ERBB4	chr2	1163124
CCND3	chr6	6915	CTCF	chr16	76709	ESR1	chr6	412778
CCNE1	chr19	12410	CNBP	chr3	13808	EIF1AX	chrX	17326
CDK4	chr12	4794	CNOT3	chr19	14284	FAM135B	chr8	366799
CDK6	chr7	228996	CD274	chr9	20022	FAM47C	chrX	3269
CYSLTR2	chr13	4671	CDC73	chr1	131884	FAT3	chr11	543929
CRLF2	chrX	22260	CHEK2	chr22	54075	FES	chr15	11364
DDX5	chr17	6905	CHD2	chr15	127685	FLNA	chrX	26112
DDX6	chr11	43182	CIITA	chr16	47785	FKBP9	chr7	49526
DEK	chr6	40955	CLTC	chr17	77267	FOXL2	chr3	2916
DGCR8	chr22	31639	CLTCL1	chr22	112241	FOXO1	chr13	110930
DDR2	chr1	154935	CCDC6	chr10	117893	FOXO3	chr6	123902
DDIT3	chr12	3928	CNTNAP2	chr7	2304639	FOXO4	chrX	7338
ELK4	chr1	24019	CBFB	chr16	71777	GATA1	chrX	7754
EGFR	chr7	192527	CBFA2T3	chr16	102346	GATA3	chr10	20550
ERBB3	chr12	23644	CSMD3	chr8	1214171	GPC3	chrX	449892
ETV1	chr7	98718	CUL3	chr2	115198	HOXA11	chr7	3701
ETV4	chr17	18588	CCNB1IP1	chr14	21944	HOXA9	chr7	3095
ETV5	chr3	62804	CCNC	chr6	26593	IRS4	chrX	3927
EWSR1	chr22	32027	CDK12	chr17	73107	ITGAV	chr2	90836
XPO1	chr2	60434	CDKN1B	chr12	5247	IRF4	chr6	19708
FUBP1	chr1	30681	CDKN2A	chr9	6810	ISX	chr22	21251
FCGR2B	chr1	15493	CDKN2C	chr1	13888	JAK1	chr1	133275
FCRL4	chr1	24331	CARS	chr11	56515	KLF4	chr9	4794
FEV	chr2	4570	CPEB3	chr10	194637	LEF1	chr4	120877
FGFR1	chr8	56707	DDX10	chr11	275905	KDM6A	chrX	239091
FGFR2	chr10	118546	DDX3X	chrX	18384	KMT2D	chr12	36349
FGFR3	chr4	15560	DICER1	chr14	71782	MB21D2	chr3	121346
FGFR4	chr5	11258	DNMT3A	chr2	114735	MRTFA	chr22	122245
FLT3	chr13	97318	POLD1	chr19	33670	MALAT1	chr11	8707
FLT4	chr5	48118	POLG	chr15	18542	MAP2K4	chr17	122996
FSTL3	chr19	7034	DROSHA	chr5	131564	MAP3K1	chr5	80578
FOXA1	chr14	5050	DNM2	chr19	113772	MAP3K13	chr3	125977
FOXP1	chr3	629274	MLH1	chr3	57586	NBEA	chr13	730448
FOXR1	chr11	9584	ELF3	chr1	8064	NTRK1	chr1	20748
FLI1	chr11	119497	EBF1	chr5	403841	NKX2-1	chr14	3836
GATA2	chr3	13758	ELL	chr19	79464	NOTCH1	chr9	51353
GLI1	chr12	12124	EED	chr11	33590	NOTCH2	chr1	158073
GRM3	chr7	220970	EPS15	chr1	165065	NFE2L2	chr2	34826
GNA11	chr19	29594	ETNK1	chr12	65590	NFKB2	chr10	7927
GNAS	chr20	58478	ETV6	chr12	245548	PAX5	chr9	200910
GNAQ	chr9	315371	EIF3E	chr8	47022	PMS1	chr2	93248
H3F3A	chr1	8024	ERCC2	chr19	20750	PABPC1	chr8	19796

H3F3B	chr17	3345	ERCC3	chr2	36886	PCBP1	chr2	1749
HEY1	chr8	3844	ERCC4	chr16	32188	PRPF40B	chr12	21229
HLF	chr17	60053	ERCC5	chr13	31157	PRKCB	chr16	384628
HNRNPA2B1	chr7	10819	BRCA1	chr17	81188	PRKAR1A	chr17	21044
HMGA1	chr6	9357	BRCA2	chr13	84792	PTK6	chr20	9353
HMGA2	chr12	90987	CYLD	chr16	59175	QKI	chr6	163953
HIST1H3B	chr6	410	TENT5C	chr1	22438	RAD21	chr8	28931
HOXA13	chr7	5742	FANCA	chr16	79097	RGPD3	chr2	63386
HOXC11	chr12	4517	FANCC	chr9	218648	RHOA	chr3	53057
HOXC13	chr12	7779	FANCD2	chr3	73246	RECQL4	chr8	6535
HOXD11	chr2	2302	FANCE	chr6	14742	RGS7	chr1	581715
HOXD13	chr2	3047	FANCF	chr11	4268	RUNX1	chr21	261543
HIP1	chr7	205647	FANCG	chr9	6178	RUNX1T1	chr8	140687
HIF1A	chr14	50637	FAS	chr10	25128	STAT5B	chr17	77238
IKBKB	chr8	60306	FAT1	chr4	136072	SUZ12	chr17	64027
IL6ST	chr5	59898	FAT4	chr4	176533	TBX3	chr12	13335
IL7R	chr5	22754	FBXW7	chr4	215519	TERT	chr5	41880
IDH1	chr2	18057	FBXO11	chr2	98873	TET1	chr10	133826
IDH2	chr15	19459	FBLN2	chr3	89291	TCF3	chr19	43039
JAK2	chr9	142938	FEN1	chr11	4607	TBL1XR1	chr3	177123
JAK3	chr19	23246	FLCN	chr17	24927	TRIM24	chr7	129659
JUN	chr1	3539	FHIT	chr3	1500052	TP53	chr17	19136
KAT6A	chr8	122508	FH	chr1	22158	TP63	chr3	265852
KNSTRN	chr15	11524	FUS	chr16	14761	WT1	chr11	47855
LPP	chr3	677738	GRIN2A	chr16	429350	ZNF429	chr19	32514
LMO1	chr11	39554	GPC5	chr13	1468630	ZNF479	chr7	20250
