# Supplementary material for: A practical framework RNMF for exploring the association between mutational signatures and genes using gene cumulative contribution abundance
Source: Cancer Med. 2022 May 16;11(21):4053–69. doi: 10.1002/cam4.4717 (PMC9636515; doi:10.1002/cam4.4717)
Supplement: Supplementary file 15 — Table S6 [file CAM4-11-4053-s014.pdf]

Table S6a. Association between CCA of genes undering age signature and prognosis.

GeneName	CCA>=0.06	CCA<0.06	pvalue	qvalue(fdr)
**CASP8**	**7**	**1064**	**1.51E-05**	**0.00053058**
**PTPRC**	**5**	**1066**	**0.000165698**	**0.005477774**
**RNF213**	**14**	**1057**	**0.000845889**	**0.018284226**
**KAT6B**	**6**	**1065**	**0.001488093**	**0.029868145**
**CIC**	**6**	**1065**	**0.002614567**	**0.04630824**
NFE2L2	35	1036	0.007506364	0.111015172
TP53	467	604	0.043610352	0.350128822
IDH1	1	1070	1.29E-14	1.81E-12
AKT2	1	1070	1.29E-14	1.81E-12
FANCE	1	1070	1.29E-14	1.81E-12
FANCG	1	1070	1.29E-14	1.81E-12
STRN	1	1070	3.34E-10	3.13E-08
TCF12	1	1070	3.34E-10	3.13E-08
C15orf65	1	1070	2.07E-09	1.66E-07
ATIC	1	1070	1.13E-07	5.27E-06
MALT1	1	1070	1.13E-07	5.27E-06
TAL1	1	1070	1.13E-07	5.27E-06
TFEB	1	1070	1.13E-07	5.27E-06
FOXO1	1	1070	1.13E-07	5.27E-06
TNFRSF14	1	1070	3.07E-07	1.33E-05
MLLT10	3	1068	6.66E-07	2.68E-05
ROS1	4	1067	8.55E-07	3.20E-05
PRDM1	4	1067	0.00019349	0.006041177
DCTN1	3	1068	0.000276383	0.008175123
ZNF384	2	1069	0.000333525	0.008520057
STK11	1	1070	0.000333525	0.008520057
TRAF7	1	1070	0.000333525	0.008520057
PRPF40B	1	1070	0.000382315	0.009341773
ACSL6	4	1067	0.000452828	0.01060372
PMS2	1	1070	0.000495919	0.011148252
FGFR1OP	2	1069	0.001115801	0.023225195
IDH2	1	1070	0.002636768	0.04630824
CDH1	2	1069	0.002636768	0.04630824
PTK6	1	1070	0.002636768	0.04630824
HIST1H3B	2	1069	0.003131954	0.053338128
SRSF3	2	1069	0.003467473	0.057315284
SMARCE1	2	1069	0.004315683	0.069297543
FGFR3	3	1068	0.005724765	0.089369941
FEN1	2	1069	0.00705645	0.10718175
JAZF1	1	1070	0.009835352	0.128545767
CSF3R	1	1070	0.009835352	0.128545767

MPL	1	1070	0.009835352	0.128545767
OLIG2	2	1069	0.009413082	0.128545767
TLX1	1	1070	0.009835352	0.128545767
CHST11	2	1069	0.010343917	0.132120033
PAX3	4	1067	0.011289166	0.140989141
ZCCHC8	2	1069	0.012294021	0.150200863
CSMD3	58	1013	0.012979484	0.155201494
BRIP1	5	1066	0.0133666	0.156500612
FNBP1	3	1068	0.014341656	0.164490015
GNA11	2	1069	0.015348536	0.172517548
AKAP9	15	1056	0.016784942	0.184963478
NUMA1	8	1063	0.022203236	0.227533323
MSH2	3	1068	0.022267496	0.227533323
TPM3	1	1070	0.021319051	0.227533323
VHL	1	1070	0.022122331	0.227533323
STIL	4	1067	0.023152081	0.231109312
SRC	2	1069	0.023439912	0.231109312
SNX29	7	1064	0.024152824	0.234032538
NOTCH2	17	1054	0.025970117	0.247376364
NIN	4	1067	0.027131382	0.254130611
ERG	1	1070	0.035853933	0.303185378
CCDC6	1	1070	0.035853933	0.303185378
CBFA2T3	2	1069	0.036144876	0.303185378
CCNC	1	1070	0.035853933	0.303185378
KDM5C	5	1066	0.033663796	0.303185378
SPEN	13	1058	0.035961596	0.303185378
DDB2	1	1070	0.035853933	0.303185378
H3F3B	3	1068	0.038495024	0.318150052
PRDM16	3	1068	0.042636313	0.347269678
KDM6A	12	1059	0.047158421	0.373282148
KDR	6	1065	0.049634381	0.387423916
SDHC	3	1068	0.050346403	0.387598335
BIRC6	8	1063	0.056331484	0.411146673
SH2B3	1	1070	0.055627619	0.411146673
GATA1	1	1070	0.055627619	0.411146673
STAT5B	1	1070	0.055627619	0.411146673
CAMTA1	8	1063	0.060183121	0.433627101
FLT3	2	1069	0.065236638	0.46408849
GRIN2A	12	1059	0.069925322	0.487841943
SETD1B	5	1066	0.070311739	0.487841943
COL2A1	6	1065	0.076088924	0.488989559
MNX1	1	1070	0.07656776	0.488989559
MAP2K1	1	1070	0.07656776	0.488989559

AMER1	3	1068	0.074885219	0.488989559
BAZ1A	6	1065	0.076229105	0.488989559
SMARCA4	6	1065	0.073568628	0.488989559
BTK	1	1070	0.07656776	0.488989559
ACSL3	6	1065	0.078906641	0.49180442
PWWP2A	2	1069	0.078023764	0.49180442
EXT2	1	1070	0.079633812	0.49180442
PRDM2	10	1061	0.081450926	0.497558919
CHD2	9	1062	0.08681022	0.524595091
NRAS	1	1070	0.093904564	0.549732971
HNF1A	3	1068	0.092862778	0.549732971
LRIG3	4	1067	0.0931402	0.549732971
PAX7	4	1067	0.097691079	0.56022843
JUN	1	1070	0.097251898	0.56022843
PDE4DIP	27	1044	0.100863059	0.566850391
CLTCL1	4	1067	0.1007959	0.566850391
TFG	2	1069	0.105303911	0.583773596
PDGFRA	5	1066	0.105951791	0.583773596
ATP2B3	5	1066	0.107973097	0.589134761
BCL3	1	1070	0.125277811	0.664208774
LCK	3	1068	0.123471372	0.664208774
BARD1	5	1066	0.124283268	0.664208774
DNAJB1	3	1068	0.133876858	0.677084848
FGFR4	5	1066	0.14019561	0.677084848
PSIP1	1	1070	0.140401482	0.677084848
RET	4	1067	0.138021721	0.677084848
ABL1	4	1067	0.134130912	0.677084848
FH	4	1067	0.136656791	0.677084848
ROBO2	4	1067	0.131959665	0.677084848
SPOP	1	1070	0.140401482	0.677084848
CYP2C8	2	1069	0.141674121	0.677084848
FAM135B	28	1043	0.133994555	0.677084848
KMT2D	49	1022	0.136517439	0.677084848
NOTCH1	95	976	0.142163723	0.677084848
TSC1	5	1066	0.143712429	0.678382273
WNK2	7	1064	0.144850307	0.678382273
MSH6	4	1067	0.149669836	0.695160728
A1CF	6	1065	0.152429575	0.702175581
ITGAV	3	1068	0.155449734	0.710266265
HNRNPA2B1	3	1068	0.157810102	0.715236109
DDR2	7	1064	0.160936771	0.715879867
UBR5	15	1056	0.159275511	0.715879867
PER1	3	1068	0.163047372	0.715879867

RBM10	3	1068	0.162938243	0.715879867
CLIP1	4	1067	0.166383052	0.719157583
NUP214	3	1068	0.170958859	0.719157583
ZMYM2	3	1068	0.172029892	0.719157583
FANCC	3	1068	0.172358671	0.719157583
PRF1	7	1064	0.174031017	0.719157583
POT1	1	1070	0.170285157	0.719157583
TSC2	4	1067	0.168894677	0.719157583
ELF4	3	1068	0.172978065	0.719157583
ABL2	5	1066	0.181726293	0.740073745
SUFU	3	1068	0.180568423	0.740073745
FCRL4	3	1068	0.186793859	0.750648837
MEN1	1	1070	0.187129562	0.750648837
EPHA3	8	1063	0.188330046	0.750648837
PHOX2B	5	1066	0.190294734	0.753138312
PLCG1	3	1068	0.192451315	0.756347127
ERC1	4	1067	0.234074313	0.77575719
EIF4A2	2	1069	0.234900111	0.77575719
GOLGA5	2	1069	0.265807737	0.77575719
LSM14A	2	1069	0.264227822	0.77575719
NFIB	3	1068	0.239884432	0.77575719
PRCC	2	1069	0.221234376	0.77575719
SLC45A3	2	1069	0.251274364	0.77575719
TOP1	2	1069	0.257253975	0.77575719
CTNNA2	13	1058	0.273912129	0.77575719
ETV1	5	1066	0.200711151	0.77575719
FLT4	6	1065	0.233763829	0.77575719
FOXA1	3	1068	0.220415231	0.77575719
FOXP1	3	1068	0.269837564	0.77575719
FOXR1	1	1070	0.263918441	0.77575719
GNAS	12	1059	0.274689823	0.77575719
H3F3A	2	1069	0.253808418	0.77575719
MITF	4	1067	0.204532415	0.77575719
POU2AF1	3	1068	0.211470957	0.77575719
RAC1	2	1069	0.234981788	0.77575719
SETBP1	11	1060	0.215226562	0.77575719
STAT3	5	1066	0.273507723	0.77575719
SMO	3	1068	0.221354829	0.77575719
USP8	2	1069	0.237468848	0.77575719
AKT3	2	1069	0.234981788	0.77575719
KIT	4	1067	0.228009898	0.77575719
ACVR2A	2	1069	0.241529589	0.77575719
ASXL2	6	1065	0.272753667	0.77575719

ARID1B	6	1065	0.238114342	0.77575719
BUB1B	2	1069	0.241108389	0.77575719
CDH10	18	1053	0.265694999	0.77575719
CDC73	1	1070	0.231603482	0.77575719
CIITA	8	1063	0.261007095	0.77575719
CDKN2A	33	1038	0.223107567	0.77575719
DNM2	2	1069	0.244176796	0.77575719
ETV6	2	1069	0.257544622	0.77575719
ERCC2	3	1068	0.205421322	0.77575719
ERCC5	7	1064	0.203678139	0.77575719
BRCA2	8	1063	0.211205847	0.77575719
FUS	2	1069	0.247578126	0.77575719
ID3	2	1069	0.27156427	0.77575719
IGF2BP2	2	1069	0.256482613	0.77575719
LATS2	2	1069	0.260246365	0.77575719
EXT1	2	1069	0.258816028	0.77575719
NAB2	2	1069	0.254352404	0.77575719
PIK3R1	2	1069	0.223500184	0.77575719
PBRM1	5	1066	0.266752102	0.77575719
ARHGAP26	2	1069	0.236861429	0.77575719
ARHGEF12	5	1066	0.240911451	0.77575719
SDHB	2	1069	0.23403774	0.77575719
SMARCD1	2	1069	0.249032412	0.77575719
USP44	2	1069	0.263068786	0.77575719
CD209	2	1069	0.257544622	0.77575719
FAT3	38	1033	0.22793376	0.77575719
FKBP9	1	1070	0.231603482	0.77575719
JAK1	2	1069	0.266314034	0.77575719
TCF3	2	1069	0.247918536	0.77575719
AFF1	3	1068	0.408981776	0.776695289
ALDH2	1	1070	0.337248505	0.776695289
BCR	11	1060	0.313401463	0.776695289
CRTC3	1	1070	0.385922639	0.776695289
EML4	3	1068	0.364284318	0.776695289
EZR	1	1070	0.402894233	0.776695289
FAM131B	3	1068	0.372867137	0.776695289
FIP1L1	1	1070	0.419287106	0.776695289
GOPC	1	1070	0.395300839	0.776695289
HERPUD1	2	1069	0.311461884	0.776695289
HOOK3	1	1070	0.395300839	0.776695289
ITK	1	1070	0.391950155	0.776695289
KLK2	1	1070	0.391950155	0.776695289
LMNA	2	1069	0.299920249	0.776695289

LCP1	2	1069	0.389360932	0.776695289
MLLT6	5	1066	0.293058412	0.776695289
PAX8	1	1070	0.407619244	0.776695289
PCM1	4	1067	0.393604131	0.776695289
PAFAH1B2	1	1070	0.337248505	0.776695289
TPM4	1	1070	0.395300839	0.776695289
NT5C2	3	1068	0.305693664	0.776695289
AFF3	13	1058	0.330832054	0.776695289
AFF4	5	1066	0.348413294	0.776695289
BCL2L12	1	1070	0.407619244	0.776695289
BRD3	1	1070	0.385922639	0.776695289
CREB3L2	1	1070	0.440780193	0.776695289
CARD11	6	1065	0.295169469	0.776695289
CCR7	2	1069	0.413530834	0.776695289
CD28	1	1070	0.440780193	0.776695289
CSF1R	3	1068	0.43356585	0.776695289
CDK4	2	1069	0.394938992	0.776695289
DEK	1	1070	0.417847639	0.776695289
ELK4	1	1070	0.433111469	0.776695289
EGFR	7	1064	0.309324746	0.776695289
ETV5	3	1068	0.373622612	0.776695289
XPO1	5	1066	0.368569102	0.776695289
FGFR2	1	1070	0.391950155	0.776695289
FLI1	1	1070	0.385922639	0.776695289
IKBKB	1	1070	0.41690198	0.776695289
IL6ST	1	1070	0.419287106	0.776695289
KDM5A	7	1064	0.399693331	0.776695289
KMT2A	11	1060	0.443279478	0.776695289
MAML2	6	1065	0.384282893	0.776695289
MTCP1	1	1070	0.453302589	0.776695289
MUC4	6	1065	0.295305154	0.776695289
MSI2	1	1070	0.439748565	0.776695289
NUP98	3	1068	0.342432375	0.776695289
PIK3CB	7	1064	0.364127398	0.776695289
PTPN11	2	1069	0.427152138	0.776695289
SRSF2	1	1070	0.411623741	0.776695289
SETDB1	3	1068	0.351025341	0.776695289
SET	2	1069	0.351845493	0.776695289
SIX1	1	1070	0.411623741	0.776695289
SIX2	1	1070	0.438674524	0.776695289
SALL4	1	1070	0.314749166	0.776695289
SSX1	1	1070	0.430792583	0.776695289
TAF15	1	1070	0.445739552	0.776695289

TAL2	1	1070	0.427152138	0.776695289
TRRAP	10	1061	0.453235368	0.776695289
USP6	15	1056	0.451732751	0.776695289
MAFB	1	1070	0.375455671	0.776695289
MYCL	1	1070	0.411623741	0.776695289
MYCN	1	1070	0.453302589	0.776695289
RAF1	1	1070	0.453302589	0.776695289
ZNF521	13	1058	0.330293926	0.776695289
EP300	40	1031	0.448186101	0.776695289
ARID2	7	1064	0.431145418	0.776695289
ATM	8	1063	0.348774571	0.776695289
BAX	2	1069	0.385922639	0.776695289
BCOR	2	1069	0.414260415	0.776695289
CD274	1	1070	0.395300839	0.776695289
CCNB1IP1	1	1070	0.407619244	0.776695289
CPEB3	2	1069	0.419287106	0.776695289
DICER1	7	1064	0.312027226	0.776695289
DNMT3A	2	1069	0.385922639	0.776695289
MLH1	4	1067	0.342903019	0.776695289
EIF3E	1	1070	0.411623741	0.776695289
BRCA1	5	1066	0.386747134	0.776695289
FANCD2	7	1064	0.370814445	0.776695289
FAT4	29	1042	0.305709296	0.776695289
FLCN	1	1070	0.411623741	0.776695289
KEAP1	16	1055	0.327156194	0.776695289
N4BP2	2	1069	0.302675747	0.776695289
NDRG1	1	1070	0.407619244	0.776695289
NCOA4	2	1069	0.282577042	0.776695289
PALB2	5	1066	0.328340495	0.776695289
PPARG	3	1068	0.33679834	0.776695289
PTPRT	8	1063	0.316360803	0.776695289
RAD51B	1	1070	0.453302589	0.776695289
RPL10	1	1070	0.445739552	0.776695289
RPL5	1	1070	0.385922639	0.776695289
RFWD3	1	1070	0.427152138	0.776695289
SBDS	1	1070	0.417847639	0.776695289
SIRPA	1	1070	0.419287106	0.776695289
SMAD3	1	1070	0.385922639	0.776695289
STAG1	4	1067	0.302710106	0.776695289
STAG2	3	1068	0.298589479	0.776695289
SDHA	1	1070	0.430792583	0.776695289
TRIM33	2	1069	0.337248505	0.776695289
YWHAE	1	1070	0.41162043	0.776695289

WRN	4	1067	0.354948888	0.776695289
XPC	1	1070	0.437589284	0.776695289
ZFHX3	17	1054	0.280941105	0.776695289
PATZ1	1	1070	0.41162043	0.776695289
ZNF331	6	1065	0.449056328	0.776695289
BIRC3	2	1069	0.440780193	0.776695289
BCLAF1	24	1047	0.301748677	0.776695289
BCORL1	3	1068	0.396574793	0.776695289
BMP5	4	1067	0.390837555	0.776695289
CBL	1	1070	0.407619244	0.776695289
CTNND1	1	1070	0.413219358	0.776695289
CRNKL1	1	1070	0.370271862	0.776695289
DCC	15	1056	0.319161203	0.776695289
DCAF12L2	8	1063	0.414748617	0.776695289
POLQ	10	1061	0.383045088	0.776695289
EPHA7	6	1065	0.316779473	0.776695289
ECT2L	1	1070	0.411623741	0.776695289
ERBB4	12	1059	0.396194458	0.776695289
FOXO3	1	1070	0.417847639	0.776695289
IRF4	1	1070	0.287800974	0.776695289
LEF1	3	1068	0.322418144	0.776695289
MB21D2	4	1067	0.378681064	0.776695289
MAP2K4	1	1070	0.395300839	0.776695289
NKX2-1	4	1067	0.424627964	0.776695289
NFKB2	1	1070	0.407619244	0.776695289
RUNX1	1	1070	0.395300839	0.776695289
RUNX1T1	15	1056	0.372724067	0.776695289
TBX3	5	1066	0.298842559	0.776695289
TRIM24	4	1067	0.396168467	0.776695289
ACKR3	1	1070	0.456719497	0.777642679
RB1	22	1049	0.459390337	0.777642679
ARHGEF10	7	1064	0.458404972	0.777642679
SUZ12	1	1070	0.458875868	0.777642679
BRD4	4	1067	0.462545919	0.780633052
SPECC1	3	1068	0.468457625	0.78084583
CDH17	1	1070	0.469858578	0.78084583
CTNNB1	4	1067	0.465667833	0.78084583
NUTM1	5	1066	0.472500876	0.78084583
HRAS	1	1070	0.473470043	0.78084583
AXIN2	5	1066	0.473787238	0.78084583
LZTR1	4	1067	0.469358178	0.78084583
PML	2	1069	0.472569172	0.78084583
SEPT5	4	1067	0.481672313	0.782369479

CNOT3	2	1069	0.480353008	0.782369479
FANCA	3	1068	0.480325767	0.782369479
PTCH1	10	1061	0.479951612	0.782369479
EZH2	5	1066	0.477260107	0.782369479
ELF3	1	1070	0.483425555	0.782954357
PCBP1	1	1070	0.48639064	0.78549293
CHD4	7	1064	0.490507124	0.787614295
ERBB3	5	1066	0.490369314	0.787614295
AR	5	1066	0.496565477	0.791915815
AKT1	1	1070	0.496937394	0.791915815
FAM47C	6	1065	0.497413315	0.791915815
ARID1A	3	1068	0.504828689	0.801451196
SKI	3	1068	0.510861391	0.807101481
SMAD4	3	1068	0.511260013	0.807101481
GMPS	7	1064	0.51611777	0.811181594
NTRK3	6	1065	0.516731336	0.811181594
MAP3K1	3	1068	0.520689709	0.815118709
FBXW7	29	1042	0.533324506	0.832578812
PTEN	13	1058	0.534927534	0.832768072
BLM	4	1067	0.537138227	0.833899678
PABPC1	15	1056	0.541347304	0.835816443
PRKCB	2	1069	0.541013301	0.835816443
COL3A1	12	1059	0.549685968	0.837191095
BCL11A	7	1064	0.549572721	0.837191095
REL	3	1068	0.548248256	0.837191095
DROSHA	7	1064	0.547071299	0.837191095
ERCC4	7	1064	0.54503779	0.837191095
GPHN	2	1069	0.553530825	0.840075566
CTNND2	17	1054	0.557045684	0.840075566
MECOM	4	1067	0.554819166	0.840075566
TSHR	2	1069	0.55755905	0.840075566
RGS7	11	1060	0.56085253	0.842778401
NCOA2	8	1063	0.563464616	0.844445637
PTPRD	22	1049	0.568937834	0.849069727
RANBP2	7	1064	0.56972841	0.849069727
RNF43	4	1067	0.571082486	0.849069727
NONO	1	1070	0.587849196	0.849283414
FUBP1	2	1069	0.587849196	0.849283414
TNC	5	1066	0.581854924	0.849283414
BAP1	7	1064	0.574062481	0.849283414
KMT2C	45	1026	0.581903373	0.849283414
MGMT	3	1068	0.57851185	0.849283414
PTPRB	14	1057	0.585711116	0.849283414

ANK1	4	1067	0.586737447	0.849283414
ARNT	3	1068	0.587070765	0.849283414
FLNA	7	1064	0.575319607	0.849283414
NTRK1	3	1068	0.586289784	0.849283414
MSN	1	1070	0.594507495	0.850160845
MET	5	1066	0.590991519	0.850160845
TET2	5	1066	0.593831471	0.850160845
CNBD1	6	1065	0.592506166	0.850160845
SF3B1	4	1067	0.598372284	0.853084342
CREB3L1	4	1067	0.599587749	0.853084342
MYH11	14	1057	0.610043393	0.857110967
JAK3	3	1068	0.605872857	0.857110967
CUL3	14	1057	0.608019251	0.857110967
NCOR1	10	1061	0.605016625	0.857110967
SLC34A2	1	1070	0.609717786	0.857110967
S100A7	1	1070	0.617980156	0.860502636
MYH9	8	1063	0.618581966	0.860502636
RSPO2	5	1066	0.615815358	0.860502636
ATP1A1	3	1068	0.615651094	0.860502636
NR4A3	2	1069	0.622367313	0.862065639
PREX2	9	1062	0.624010949	0.862065639
PTPRK	4	1067	0.624307322	0.862065639
NBEA	11	1060	0.625846973	0.862073526
ASXL1	3	1068	0.62757484	0.86234
PIM1	1	1070	0.631164581	0.863052298
KLF4	1	1070	0.631164581	0.863052298
NCOA1	6	1065	0.648517899	0.86571748
PPFIBP1	1	1070	0.636889808	0.86571748
TFPT	1	1070	0.647750187	0.86571748
TCEA1	2	1069	0.639105527	0.86571748
CYSLTR2	1	1070	0.646658489	0.86571748
IL7R	3	1068	0.643813754	0.86571748
STAT6	1	1070	0.639282373	0.86571748
CBFB	2	1069	0.648313941	0.86571748
FAS	1	1070	0.639282373	0.86571748
TP63	8	1063	0.644260002	0.86571748
CASP3	1	1070	0.652057651	0.868380095
GPC5	3	1068	0.659498711	0.874146876
EPAS1	6	1065	0.659059995	0.874146876
ALK	7	1064	0.661071013	0.874169199
RABEP1	2	1069	0.670075541	0.878273655
DGCR8	3	1068	0.670425975	0.878273655
TFE3	1	1070	0.666106344	0.878273655

CDKN1A	3	1068	0.669819361	0.878273655
JAK2	6	1065	0.674326564	0.879502944
MTOR	8	1063	0.677624154	0.879502944
CDH11	12	1059	0.6770476	0.879502944
CBLB	6	1065	0.677298366	0.879502944
MAP2K2	2	1069	0.679981148	0.880528583
FCGR2B	1	1070	0.683259216	0.880714861
FANCF	1	1070	0.683259216	0.880714861
HLA-A	2	1069	0.688862885	0.884379377
MACC1	2	1069	0.689249407	0.884379377
TRIP11	5	1066	0.691040331	0.884657553
GRM3	14	1057	0.693047584	0.885210777
LATS1	6	1065	0.699378662	0.891271673
CLP1	3	1068	0.706086696	0.895100633
GAS7	5	1066	0.706232362	0.895100633
NFATC2	3	1068	0.707161354	0.895100633
TET1	5	1066	0.709289745	0.895777161
CLTC	3	1068	0.713469749	0.897024606
SFPQ	2	1069	0.712529485	0.897024606
SH3GL1	2	1069	0.718470329	0.899288029
SMARCB1	5	1066	0.717616775	0.899288029
ATR	9	1062	0.721170166	0.900661408
LPP	8	1063	0.72837968	0.904842873
MYC	1	1070	0.729348437	0.904842873
PRKAR1A	1	1070	0.727984809	0.904842873
BCL9	5	1066	0.733031974	0.905415317
DDX10	3	1068	0.732242658	0.905415317
KIAA1549	10	1061	0.737682788	0.906957844
ZEB1	9	1062	0.738229094	0.906957844
APC	4	1067	0.739122229	0.906957844
HSP90AB1	3	1068	0.749793807	0.907858144
HIF1A	3	1068	0.75281249	0.907858144
PIK3CA	16	1055	0.755910259	0.907858144
PDGFRB	5	1066	0.753772533	0.907858144
PLAG1	5	1066	0.757625391	0.907858144
SND1	4	1067	0.747676309	0.907858144
AXIN1	5	1066	0.753237717	0.907858144
IKZF1	5	1066	0.755318631	0.907858144
NF2	4	1067	0.752654943	0.907858144
PMS1	3	1068	0.747690047	0.907858144
RAD21	3	1068	0.757515265	0.907858144
MYO5A	5	1066	0.760222006	0.90903142
PICALM	1	1070	0.764573816	0.912294023

MAPK1	3	1068	0.766935832	0.913173596
LARP4B	2	1069	0.772082745	0.913495795
LRP1B	59	1012	0.769719535	0.913495795
PTPN13	4	1067	0.77063687	0.913495795
MN1	5	1066	0.776121712	0.916345382
NCKIPSD	3	1068	0.788409876	0.916431376
RARA	2	1069	0.782140719	0.916431376
RSPO3	2	1069	0.785120776	0.916431376
CYLD	4	1067	0.789708586	0.916431376
NF1	8	1063	0.785173923	0.916431376
ZBTB16	6	1065	0.78582391	0.916431376
ESR1	2	1069	0.790870493	0.916431376
EIF1AX	2	1069	0.780300791	0.916431376
FES	2	1069	0.787161576	0.916431376
CACNA1D	6	1065	0.795391657	0.919773891
FAT1	50	1021	0.797041175	0.919788789
TGFBR2	12	1059	0.799656589	0.92091599
RALGDS	4	1067	0.806277332	0.926641842
TPR	10	1061	0.816690494	0.932886296
TLX3	2	1069	0.815228737	0.932886296
ARHGEF10L	3	1068	0.815228737	0.932886296
ERCC3	2	1069	0.820207244	0.935002983
KIF5B	4	1067	0.823078687	0.936376967
NBN	2	1069	0.828823383	0.941007559
RGPD3	8	1063	0.833321699	0.944207248
KRAS	5	1066	0.839130443	0.948875873
TMPRSS2	2	1069	0.841461511	0.949106842
NCOR2	7	1064	0.842712303	0.949106842
SRGAP3	2	1069	0.852689655	0.952862128
VAV1	4	1067	0.851132288	0.952862128
ZMYM3	2	1069	0.852574437	0.952862128
APOBEC3B	2	1069	0.854524044	0.952862128
ZNF429	6	1065	0.853410329	0.952862128
CEP89	4	1067	0.867502271	0.954082733
NSD1	7	1064	0.860429626	0.954082733
RPN1	2	1069	0.862245028	0.954082733
ARHGAP5	9	1062	0.858835575	0.954082733
TNFRSF17	2	1069	0.866473972	0.954082733
WWTR1	2	1069	0.865694024	0.954082733
CDK12	8	1063	0.862792901	0.954082733
FBLN2	6	1065	0.870767392	0.95580327
ABI1	2	1069	0.872999342	0.956385244
CCR4	2	1069	0.880638223	0.957101084

CXCR4	3	1068	0.876966031	0.957101084
GLI1	4	1067	0.878033875	0.957101084
CTCF	5	1066	0.885363796	0.957101084
EPS15	2	1069	0.879137449	0.957101084
MAX	3	1068	0.885573957	0.957101084
ZNRF3	3	1068	0.882768983	0.957101084
CREBBP	19	1052	0.888310347	0.958215768
CNTRL	5	1066	0.894445876	0.958958078
LIFR	4	1067	0.905463011	0.958958078
SEPT9	2	1069	0.903876302	0.958958078
DDX5	3	1068	0.903354952	0.958958078
HOXD13	3	1068	0.902221259	0.958958078
WAS	3	1068	0.899232203	0.958958078
MED12	4	1067	0.904143715	0.958958078
NRG1	2	1069	0.903424176	0.958958078
PTPN6	1	1070	0.896764362	0.958958078
WT1	4	1067	0.906061814	0.958958078
NACA	2	1069	0.910370899	0.961707603
CRTC1	2	1069	0.917603714	0.964082229
EWSR1	2	1069	0.917427383	0.964082229
EBF1	9	1062	0.919158781	0.964082229
TBL1XR1	6	1065	0.91948056	0.964082229
KTN1	4	1067	0.925576867	0.965372116
KAT6A	10	1061	0.925864004	0.965372116
TCL1A	2	1069	0.925277727	0.965372116
ARAF	6	1065	0.935588275	0.966053478
CHEK2	13	1058	0.935278978	0.966053478
SFRP4	4	1067	0.934373244	0.966053478
SMC1A	2	1069	0.935627129	0.966053478
FOXO4	5	1066	0.93683122	0.966053478
PAX5	5	1066	0.930083805	0.966053478
MUC16	91	980	0.943099397	0.970736009
ATRX	5	1066	0.944904162	0.970815611
ELN	2	1069	0.952406622	0.973429128
MYB	2	1069	0.951713498	0.973429128
TERT	6	1065	0.952644165	0.973429128
CNTNAP2	18	1053	0.96382897	0.983070565
BRAF	6	1065	0.967183692	0.984705135
TCF7L2	3	1068	0.969886517	0.985671289
TEC	2	1069	0.974395135	0.986684803
POLE	3	1068	0.974340845	0.986684803
POLG	4	1067	0.982627465	0.989671389
PPP2R1A	4	1067	0.982308034	0.989671389

SETD2	9	1062	0.98193043	0.989671389
HSP90AA1	2	1069	0.988260753	0.993564478
COL1A1	8	1063	0.993352191	0.995122872
GATA3	5	1066	0.992146633	0.995122872
IL2	2	1069	0.999626143	0.999626143

Table S6b. Association between CCA of genes undering APOBEC signature and prognosis.

GeneName	CCA>=0.06	CCA<0.06	pvalue	qvalue(fdr)
**CBLB**	**5**	**1066**	**0.00080243**	**0.014644339**
PIK3CA	58	1013	0.699589986	0.867137308
CSF1R	2	1069	0	0
FUBP1	1	1070	1.29E-14	1.41E-12
IDH1	1	1070	1.29E-14	1.41E-12
SMARCE1	1	1070	1.29E-14	1.41E-12
FOXA1	1	1070	3.34E-10	1.83E-08
SRSF3	1	1070	3.34E-10	1.83E-08
ERBB2	1	1070	3.34E-10	1.83E-08
PTPRK	2	1069	3.34E-10	1.83E-08
LCK	1	1070	2.07E-09	9.05E-08
SFRP4	1	1070	2.07E-09	9.05E-08
ATIC	1	1070	1.13E-07	4.11E-06
MALT1	1	1070	1.13E-07	4.11E-06
TCF12	2	1069	1.81E-06	6.10E-05
APOBEC3B	1	1070	3.27E-06	0.000102404
KAT6B	3	1068	1.02E-05	0.000296512
SNX29	4	1067	0.000199075	0.00521121
POU2AF1	2	1069	0.000202262	0.00521121
SPECC1	2	1069	0.000333525	0.006956386
STIL	1	1070	0.000333525	0.006956386
REL	1	1070	0.000333525	0.006956386
ELF3	1	1070	0.000333525	0.006956386
PMS2	1	1070	0.000495919	0.00987329
PDGFRA	1	1070	0.000632926	0.012053118
FBLN2	2	1069	0.001074747	0.018829569
MLLT10	3	1068	0.001149123	0.019358308
MYH11	3	1068	0.002728247	0.044258229
MAP3K13	3	1068	0.003467473	0.05424118
EBF1	3	1068	0.006540162	0.098779004
FEN1	2	1069	0.00705645	0.103024166
BCL9	5	1066	0.008074036	0.114078314
PDE4DIP	15	1056	0.009495407	0.12670248
TLX1	1	1070	0.009835352	0.12670248

ASXL2	1	1070	0.009835352	0.12670248
CHST11	2	1069	0.010343917	0.129446735
H3F3B	2	1069	0.010869078	0.132240444
RNF213	8	1063	0.01331222	0.157587907
FBXO11	3	1068	0.013956701	0.160869346
FCRL4	4	1067	0.017298633	0.193515657
PAX3	2	1069	0.017672663	0.193515657
BRCA1	7	1064	0.01981561	0.206648507
KMT2A	3	1068	0.019502399	0.206648507
VTI1A	1	1070	0.021319051	0.212221466
IKBKB	1	1070	0.021319051	0.212221466
VHL	1	1070	0.022122331	0.215324018
TET2	5	1066	0.028844109	0.274646082
NR4A3	3	1068	0.031190515	0.290669057
CANT1	1	1070	0.035853933	0.302000434
EIF4A2	1	1070	0.035853933	0.302000434
CCDC6	1	1070	0.035853933	0.302000434
KLF6	1	1070	0.035853933	0.302000434
RAD51B	1	1070	0.035853933	0.302000434
BAZ1A	5	1066	0.037419937	0.308189701
SOX21	1	1070	0.037995991	0.308189701
PRDM1	4	1067	0.042232799	0.336326654
SPEN	9	1062	0.045334525	0.354580745
RB1	9	1062	0.047666295	0.366277849
COL2A1	3	1068	0.050573034	0.381913603
BRCA2	9	1062	0.051953142	0.385686038
SETDB1	1	1070	0.055627619	0.406081617
MAP3K1	1	1070	0.05694745	0.408901362
EPHA7	2	1069	0.058281634	0.411731542
FLT3	2	1069	0.065236638	0.439870898
MET	2	1069	0.065731976	0.439870898
NCOA2	5	1066	0.065924327	0.439870898
ZEB1	5	1066	0.066281916	0.439870898
CDH10	10	1061	0.06904318	0.447507037
PTEN	8	1063	0.069475978	0.447507037
PMS1	2	1069	0.072172689	0.458139679
ZMYM2	5	1066	0.076044963	0.459406561
AMER1	2	1069	0.074885219	0.459406561
DDX10	1	1070	0.07656776	0.459406561
GPC5	1	1070	0.07656776	0.459406561
MDM4	2	1069	0.081950214	0.478589249
SMAD3	2	1069	0.081950214	0.478589249
HIST1H3B	4	1067	0.084149567	0.484967239

NUMA1	5	1066	0.097133321	0.49530618
PAX7	2	1069	0.090949475	0.49530618
PWWP2A	1	1070	0.097251898	0.49530618
FLT4	1	1070	0.097251898	0.49530618
GNA11	1	1070	0.097251898	0.49530618
JUN	1	1070	0.097251898	0.49530618
PRDM16	2	1069	0.095269913	0.49530618
TAF15	1	1070	0.097251898	0.49530618
LARP4B	1	1070	0.093904564	0.49530618
ARHGAP26	2	1069	0.093904564	0.49530618
TP53	127	944	0.105717437	0.532232616
TBL1XR1	2	1069	0.111394252	0.554439571
CIC	2	1069	0.120031598	0.590717304
BCL3	1	1070	0.125277811	0.60298551
PTPN11	2	1069	0.125277811	0.60298551
LRP1B	32	1039	0.137218586	0.653279788
PSIP1	1	1070	0.140401482	0.654211161
KDM5C	1	1070	0.140401482	0.654211161
ERC1	2	1069	0.142342905	0.656275711
GRIN2A	3	1068	0.148333878	0.676773319
ABL2	3	1068	0.154227115	0.696406974
EP300	25	1046	0.156010675	0.697272199
KIF5B	3	1068	0.16282608	0.702474822
KDM5A	3	1068	0.161462786	0.702474822
FAT1	30	1041	0.163590027	0.702474822
MSH6	3	1068	0.160169618	0.702474822
RSPO2	1	1070	0.170285157	0.72412523
ACSL3	1	1070	0.407619244	0.733531498
AFF1	1	1070	0.391950155	0.733531498
BCR	8	1063	0.384277445	0.733531498
CNTRL	5	1066	0.319062799	0.733531498
CEP89	2	1069	0.315703817	0.733531498
CLP1	1	1070	0.411623741	0.733531498
DCTN1	1	1070	0.417847639	0.733531498
EML4	1	1070	0.430792583	0.733531498
FGFR1OP	3	1068	0.187320285	0.733531498
FNBP1	4	1067	0.427657489	0.733531498
GOPC	1	1070	0.456719497	0.733531498
GMPS	2	1069	0.430792583	0.733531498
HSP90AA1	2	1069	0.255752327	0.733531498
HOOK3	1	1070	0.417847639	0.733531498
IL2	1	1070	0.385922639	0.733531498
LIFR	1	1070	0.411623741	0.733531498

LSM14A	1	1070	0.433111469	0.733531498
MYO5A	1	1070	0.433111469	0.733531498
NIN	3	1068	0.455901098	0.733531498
NFIB	3	1068	0.364234347	0.733531498
RALGDS	1	1070	0.440780193	0.733531498
SEPT5	2	1069	0.259943991	0.733531498
SRGAP3	1	1070	0.445739552	0.733531498
TRIP11	4	1067	0.254532276	0.733531498
TOP1	2	1069	0.257253975	0.733531498
TCEA1	1	1070	0.430792583	0.733531498
TMPRSS2	1	1070	0.385922639	0.733531498
ZCCHC8	2	1069	0.179278156	0.733531498
NT5C2	2	1069	0.265858623	0.733531498
AFF3	6	1065	0.299100978	0.733531498
A1CF	4	1067	0.36769271	0.733531498
ARAF	2	1069	0.244529837	0.733531498
ACKR3	1	1070	0.456719497	0.733531498
BCL11A	3	1068	0.250930125	0.733531498
BRD3	1	1070	0.385922639	0.733531498
BRD4	1	1070	0.430792583	0.733531498
CACNA1D	4	1067	0.290794736	0.733531498
CTNNA2	3	1068	0.180217055	0.733531498
CTNND2	8	1063	0.232097433	0.733531498
CCR7	1	1070	0.458875868	0.733531498
CDK6	1	1070	0.430792583	0.733531498
DEK	1	1070	0.417847639	0.733531498
DGCR8	1	1070	0.407619244	0.733531498
ETV1	1	1070	0.395300839	0.733531498
ETV5	3	1068	0.373622612	0.733531498
EWSR1	1	1070	0.263918441	0.733531498
FGFR2	1	1070	0.292702882	0.733531498
FOXR1	1	1070	0.263918441	0.733531498
HNRNPA2B1	1	1070	0.373078968	0.733531498
IL7R	2	1069	0.227147276	0.733531498
JAK3	1	1070	0.347435254	0.733531498
KAT6A	3	1068	0.37808569	0.733531498
KAT7	2	1069	0.233197604	0.733531498
MAML2	2	1069	0.342338277	0.733531498
MTOR	6	1065	0.438417231	0.733531498
MITF	1	1070	0.445739552	0.733531498
MN1	1	1070	0.206813106	0.733531498
NUP98	1	1070	0.41162043	0.733531498
PREX2	4	1067	0.328854923	0.733531498

PDGFRB	3	1068	0.424946816	0.733531498
PBX1	1	1070	0.417847639	0.733531498
RAP1GDS1	1	1070	0.433111469	0.733531498
SH3GL1	1	1070	0.411623741	0.733531498
SIX1	1	1070	0.411623741	0.733531498
SKI	2	1069	0.453373144	0.733531498
SALL4	1	1070	0.314749166	0.733531498
SF3B1	3	1068	0.1929183	0.733531498
SND1	1	1070	0.430792583	0.733531498
TCL1A	1	1070	0.400378314	0.733531498
TCF7L2	1	1070	0.395300839	0.733531498
TFE3	1	1070	0.41162043	0.733531498
UBR5	6	1065	0.442411011	0.733531498
MAFB	1	1070	0.375455671	0.733531498
MYCL	1	1070	0.411623741	0.733531498
MYCN	1	1070	0.453302589	0.733531498
ACVR2A	2	1069	0.241529589	0.733531498
ASXL1	4	1067	0.40946466	0.733531498
APC	8	1063	0.348257508	0.733531498
ARID1A	3	1068	0.370808522	0.733531498
ARID1B	3	1068	0.379166136	0.733531498
ATM	6	1065	0.379002838	0.733531498
ATP2B3	1	1070	0.395300839	0.733531498
AXIN1	2	1069	0.396184077	0.733531498
AXIN2	1	1070	0.391950155	0.733531498
BRIP1	4	1067	0.30168207	0.733531498
BUB1B	3	1068	0.225347311	0.733531498
CAMTA1	3	1068	0.303427301	0.733531498
CNTNAP2	6	1065	0.378526352	0.733531498
CDKN1B	1	1070	0.411623741	0.733531498
CDKN2A	7	1064	0.418048562	0.733531498
DNMT3A	1	1070	0.385922639	0.733531498
POLG	1	1070	0.391950155	0.733531498
DNM2	1	1070	0.385922639	0.733531498
MLH1	2	1069	0.243132888	0.733531498
EIF3E	2	1069	0.259714145	0.733531498
ERCC2	1	1070	0.263918441	0.733531498
ERCC5	2	1069	0.282966218	0.733531498
FANCC	1	1070	0.395300839	0.733531498
FANCD2	5	1066	0.43682908	0.733531498
FANCE	1	1070	0.413219358	0.733531498
FANCF	2	1069	0.382974853	0.733531498
FBXW7	16	1055	0.37339786	0.733531498

PTCH1	5	1066	0.458230144	0.733531498
IGF2BP2	2	1069	0.256482613	0.733531498
KEAP1	4	1067	0.286371554	0.733531498
LATS1	5	1066	0.218486274	0.733531498
LRIG3	1	1070	0.433111469	0.733531498
KMT2C	19	1052	0.438347347	0.733531498
MEN1	1	1070	0.187129562	0.733531498
EXT1	1	1070	0.430792583	0.733531498
MSH2	3	1068	0.391773056	0.733531498
MAX	2	1069	0.390995472	0.733531498
NRG1	3	1068	0.420211198	0.733531498
NAB2	1	1070	0.419287106	0.733531498
NBN	2	1069	0.2778505	0.733531498
NDRG1	1	1070	0.407619244	0.733531498
NTHL1	1	1070	0.422021426	0.733531498
NCOA4	1	1070	0.395300839	0.733531498
NCOR2	1	1070	0.395300839	0.733531498
MGMT	1	1070	0.443317461	0.733531498
PHOX2B	2	1069	0.307187883	0.733531498
PRDM2	2	1069	0.324306627	0.733531498
POT1	1	1070	0.391950155	0.733531498
PTPRT	3	1068	0.307687816	0.733531498
ARHGEF12	2	1069	0.411623741	0.733531498
ARHGEF10	4	1067	0.339169427	0.733531498
RFWD3	1	1070	0.427152138	0.733531498
RNF43	2	1069	0.333205089	0.733531498
SMAD4	2	1069	0.244293005	0.733531498
SPOP	2	1069	0.240580143	0.733531498
STAG2	4	1067	0.193659496	0.733531498
SDHB	1	1070	0.430792583	0.733531498
SDHC	1	1070	0.187129562	0.733531498
SUFU	2	1069	0.260246365	0.733531498
SMARCA4	3	1068	0.25734343	0.733531498
SMARCD1	1	1070	0.411623741	0.733531498
TGFBR2	4	1067	0.442831146	0.733531498
TSC1	3	1068	0.253139183	0.733531498
YWHAE	1	1070	0.41162043	0.733531498
WNK2	1	1070	0.395300839	0.733531498
XPC	1	1070	0.437589284	0.733531498
ZBTB16	4	1067	0.215662635	0.733531498
PATZ1	1	1070	0.41162043	0.733531498
BIRC3	1	1070	0.407619244	0.733531498
BMP5	2	1069	0.219505821	0.733531498

BTK	2	1069	0.199233355	0.733531498
CBL	2	1069	0.243935228	0.733531498
CTNND1	1	1070	0.41162043	0.733531498
CD209	1	1070	0.440780193	0.733531498
CNBD1	2	1069	0.206813106	0.733531498
POLQ	1	1070	0.411623741	0.733531498
EZH2	2	1069	0.256652898	0.733531498
EPHA3	4	1067	0.247371553	0.733531498
ERBB4	6	1065	0.241966952	0.733531498
ESR1	1	1070	0.433111469	0.733531498
FAM47C	3	1068	0.251274364	0.733531498
FAT3	17	1054	0.432607741	0.733531498
FES	1	1070	0.400378314	0.733531498
FKBP9	1	1070	0.231603482	0.733531498
FOXO4	2	1069	0.413971812	0.733531498
NBEA	10	1061	0.229286766	0.733531498
NOTCH2	4	1067	0.314568914	0.733531498
NFKB2	1	1070	0.407619244	0.733531498
PABPC1	2	1069	0.262138582	0.733531498
PRPF40B	1	1070	0.430792583	0.733531498
RUNX1T1	7	1064	0.397871612	0.733531498
SUZ12	1	1070	0.458875868	0.733531498
TBX3	3	1068	0.284284491	0.733531498
TCF3	1	1070	0.41162043	0.733531498
WT1	1	1070	0.195844402	0.733531498
ZNF479	1	1070	0.419287106	0.733531498
NFATC2	3	1068	0.465300937	0.734853278
IKZF1	3	1068	0.466413725	0.734853278
PTPRD	6	1065	0.46355254	0.734853278
ANK1	1	1070	0.463485221	0.734853278
VAV1	3	1068	0.468760161	0.735903048
GAS7	1	1070	0.472569172	0.738006687
N4BP2	1	1070	0.473470043	0.738006687
STAT3	4	1067	0.490083777	0.753181384
CDH11	4	1067	0.486811604	0.753181384
DDX3X	1	1070	0.48639064	0.753181384
ERCC3	1	1070	0.489147537	0.753181384
JAK2	4	1067	0.497620021	0.762089403
TRRAP	4	1067	0.502289499	0.766560281
ERBB3	5	1066	0.506458633	0.770239171
AKAP9	6	1065	0.513753518	0.774175533
CXCR4	1	1070	0.512482662	0.774175533
ARID2	4	1067	0.514349498	0.774175533

KDM6A	7	1064	0.517210966	0.775816449
TPR	7	1064	0.526158787	0.786544534
COL3A1	6	1065	0.528260293	0.787000028
PRCC	1	1070	0.534248166	0.79051711
CNOT3	4	1067	0.536035574	0.79051711
LZTR1	1	1070	0.534248166	0.79051711
NUP214	2	1069	0.543024078	0.797116246
CARD11	3	1068	0.544150131	0.797116246
MECOM	1	1070	0.553530825	0.80015347
ARHGAP5	7	1064	0.548320427	0.80015347
CUL3	7	1064	0.553262326	0.80015347
CYP2C8	1	1070	0.551646762	0.80015347
CSMD3	32	1039	0.56543057	0.814666413
RGS7	3	1068	0.568808443	0.816846223
COL1A1	4	1067	0.594451472	0.828172089
MSN	1	1070	0.594507495	0.828172089
NONO	1	1070	0.587849196	0.828172089
NCOA1	3	1068	0.620183665	0.828172089
THRAP3	1	1070	0.617980156	0.828172089
AFF4	3	1068	0.589723823	0.828172089
AR	1	1070	0.617980156	0.828172089
CALR	1	1070	0.617980156	0.828172089
CTNNB1	1	1070	0.594507495	0.828172089
EGFR	3	1068	0.602789115	0.828172089
HIF1A	1	1070	0.609717786	0.828172089
MUC16	29	1042	0.618902393	0.828172089
PIK3CB	4	1067	0.587857747	0.828172089
PLAG1	3	1068	0.620053057	0.828172089
PPM1D	1	1070	0.598005533	0.828172089
TSHR	2	1069	0.589129255	0.828172089
USP6	5	1066	0.582140426	0.828172089
WWTR1	1	1070	0.598005533	0.828172089
FUS	1	1070	0.598005533	0.828172089
PBRM1	6	1065	0.605196429	0.828172089
FLNA	2	1069	0.609246465	0.828172089
JAK1	3	1068	0.610336093	0.828172089
TRIM24	3	1068	0.605655558	0.828172089
KIAA1549	5	1066	0.622887604	0.829254621
PIM1	1	1070	0.631164581	0.835196636
KLF4	1	1070	0.631164581	0.835196636
HSP90AB1	1	1070	0.647651958	0.840686578
SETBP1	1	1070	0.639282373	0.840686578
BARD1	7	1064	0.647853056	0.840686578

CBFB	2	1069	0.648313941	0.840686578
FAS	1	1070	0.639282373	0.840686578
WIF1	1	1070	0.647750187	0.840686578
ZNF429	2	1069	0.648749003	0.840686578
CASP3	1	1070	0.652057651	0.842481567
GNAS	4	1067	0.658578788	0.848404438
ROS1	2	1069	0.668577226	0.85126984
DICER1	3	1068	0.666469269	0.85126984
PPP2R1A	5	1066	0.667621172	0.85126984
RANBP2	5	1066	0.662857038	0.85126984
SETD1B	2	1069	0.672065492	0.853230973
KTN1	5	1066	0.707525788	0.867137308
NSD1	4	1067	0.691281657	0.867137308
GLI1	3	1068	0.707054185	0.867137308
NUTM1	3	1068	0.70099633	0.867137308
BRAF	2	1069	0.708476258	0.867137308
CASP8	4	1067	0.700771628	0.867137308
CLTCL1	3	1068	0.715148856	0.867137308
ERCC4	4	1067	0.716675127	0.867137308
FH	4	1067	0.69995609	0.867137308
PER1	3	1068	0.715361149	0.867137308
ARHGEF10L	2	1069	0.698206282	0.867137308
SETD2	5	1066	0.69840816	0.867137308
CREBBP	5	1066	0.709259165	0.867137308
DCC	8	1063	0.715143672	0.867137308
GATA3	2	1069	0.686012732	0.867137308
ITGAV	3	1068	0.696550287	0.867137308
MACC1	2	1069	0.724084425	0.867983812
NCOR1	5	1066	0.726743433	0.867983812
WRN	4	1067	0.72728324	0.867983812
CDKN1A	2	1069	0.721147992	0.867983812
RGPD3	8	1063	0.724741164	0.867983812
CIITA	3	1068	0.730622189	0.869599235
GRM3	4	1067	0.733982363	0.871231098
TNC	3	1068	0.741131593	0.872622683
ZNF521	5	1066	0.739374544	0.872622683
ETV6	2	1069	0.740892569	0.872622683
CHD4	6	1065	0.750535379	0.881325726
PTPRC	1	1070	0.755222452	0.882099824
STAG1	4	1067	0.754768958	0.882099824
ITK	1	1070	0.759750613	0.882681084
FGFR4	1	1070	0.759750613	0.882681084
CLIP1	3	1068	0.765841101	0.883725548

TFRC	2	1069	0.770737806	0.883725548
ALK	3	1068	0.770686847	0.883725548
MAPK1	3	1068	0.766935832	0.883725548
ZNF331	3	1068	0.762672113	0.883725548
SET	3	1068	0.77616759	0.885316158
ATRX	6	1065	0.775639567	0.885316158
DDX5	3	1068	0.784625693	0.888131247
XPO1	3	1068	0.781759302	0.888131247
CDK12	5	1066	0.784718705	0.888131247
TET1	3	1068	0.78905776	0.890740461
ERG	2	1069	0.797164002	0.897577977
TFG	2	1069	0.803053498	0.901890851
FAM131B	3	1068	0.823909719	0.920974164
EPAS1	3	1068	0.82425085	0.920974164
BIRC6	7	1064	0.8264725	0.921106756
WAS	1	1070	0.82902498	0.92160645
CD274	2	1069	0.835801637	0.926787637
FAM135B	9	1062	0.849217918	0.939286485
ATR	2	1069	0.853716481	0.939517132
EPS15	2	1069	0.853545394	0.939517132
RPN1	2	1069	0.862245028	0.944158305
PRF1	2	1069	0.86037945	0.944158305
BAP1	4	1067	0.86991581	0.950182356
ABL1	2	1069	0.875985297	0.952063424
DROSHA	3	1068	0.873823489	0.952063424
SIRPA	2	1069	0.88620339	0.960784864
CRTC3	2	1069	0.901464238	0.960825189
NACA	2	1069	0.910370899	0.960825189
HOXD13	3	1068	0.902221259	0.960825189
LPP	5	1066	0.909858785	0.960825189
RET	2	1069	0.908542475	0.960825189
AKT3	3	1068	0.903302023	0.960825189
CTCF	2	1069	0.909485437	0.960825189
FAT4	14	1057	0.904597891	0.960825189
EXT2	1	1070	0.896764362	0.960825189
PTPN6	1	1070	0.896764362	0.960825189
BCLAF1	5	1066	0.893504696	0.960825189
MLLT6	2	1069	0.916904891	0.961508198
CRTC1	2	1069	0.917603714	0.961508198
NF1	5	1066	0.914941039	0.961508198
NFE2L2	14	1057	0.923330717	0.965200129
RAF1	2	1069	0.933689893	0.971392335
ZFHX3	10	1061	0.931707928	0.971392335

PIK3R1	2	1069	0.939635019	0.974855649
POLE	3	1068	0.943566893	0.974855649
ISX	2	1069	0.944379902	0.974855649
NOTCH1	27	1044	0.948147275	0.974855649
TP63	4	1067	0.947267806	0.974855649
CHD2	4	1067	0.954855204	0.977911859
MB21D2	2	1069	0.955585105	0.977911859
ABI1	2	1069	0.965211102	0.985305515
MYH9	5	1066	0.970477831	0.985305515
PTPN13	7	1064	0.972074302	0.985305515
ARNT	2	1069	0.970591091	0.985305515
KMT2D	28	1043	0.974057735	0.985305515
MED12	5	1066	0.976425196	0.985424507
NF2	2	1069	0.980456614	0.987218383
LCP1	2	1069	0.988538781	0.988538781
CHEK2	6	1065	0.988003651	0.988538781
PTPRB	5	1066	0.987928139	0.988538781

Table S6c. Association between CCA of genes undering APOBEC signature and prognosis.

GeneName	CCA>=0.06	CCA<0.06	pvalue	qvalue(fdr)
**UBR5**	**5**	**1066**	**0.002703035**	**0.031430636**
**ARHGAP5**	**7**	**1064**	**0.003515709**	**0.039063435**
**BRCA1**	**10**	**1061**	**0.005652824**	**0.056528244**
**SETD2**	**5**	**1066**	**0.007176192**	**0.069001843**
PIK3CA	55	1016	0.375336577	0.777546954
FGFR1	1	1070	1.29E-14	1.29E-12
MDM4	1	1070	1.29E-14	1.29E-12
SYK	1	1070	1.29E-14	1.29E-12
FHIT	1	1070	1.29E-14	1.29E-12
NTRK1	1	1070	1.29E-14	1.29E-12
CSF1R	1	1070	9.65E-14	8.04E-12
PPARG	2	1069	2.88E-10	8.80E-09
HLA-A	1	1070	3.34E-10	8.80E-09
TCEA1	1	1070	3.34E-10	8.80E-09
HNRNPA2B1	1	1070	3.34E-10	8.80E-09
KCNJ5	1	1070	3.34E-10	8.80E-09
TNC	1	1070	3.34E-10	8.80E-09
AKT2	1	1070	3.34E-10	8.80E-09
BRAF	1	1070	3.34E-10	8.80E-09
ETNK1	1	1070	3.34E-10	8.80E-09
FH	1	1070	3.34E-10	8.80E-09
BCORL1	2	1069	3.34E-10	8.80E-09
CDKN1A	1	1070	3.34E-10	8.80E-09

FOXO1	1	1070	3.34E-10	8.80E-09
C15orf65	1	1070	2.07E-09	4.69E-08
ABL2	1	1070	2.07E-09	4.69E-08
MYCL	1	1070	2.07E-09	4.69E-08
DNAJB1	1	1070	1.13E-07	2.08E-06
HIST1H4I	1	1070	1.13E-07	2.08E-06
NPM1	1	1070	1.13E-07	2.08E-06
SIX2	1	1070	1.13E-07	2.08E-06
BMP5	1	1070	1.13E-07	2.08E-06
APOBEC3B	1	1070	3.27E-06	5.84E-05
WWTR1	2	1069	2.31E-05	0.000397925
CNBD1	4	1067	0.000272319	0.004538655
CD79B	1	1070	0.000333525	0.005379441
ESR1	2	1069	0.000485322	0.007583158
TFRC	1	1070	0.000632926	0.009041804
DEK	1	1070	0.000632926	0.009041804
ARHGEF10	1	1070	0.000632926	0.009041804
KAT6B	3	1068	0.000710207	0.00986399
PTPN11	2	1069	0.001412521	0.019088128
SETBP1	2	1069	0.002411403	0.031390097
KDM5A	3	1068	0.002552927	0.031390097
WAS	1	1070	0.002636768	0.031390097
CDX2	1	1070	0.002636768	0.031390097
LEF1	1	1070	0.002636768	0.031390097
MUC4	3	1068	0.0030957	0.035178404
STIL	4	1067	0.003728996	0.04053256
NFATC2	3	1068	0.00402929	0.042864787
ZNF384	2	1069	0.004805303	0.049033702
CAMTA1	3	1068	0.004805303	0.049033702
COL2A1	3	1068	0.006376979	0.062519402
TRAF7	2	1069	0.007485247	0.070615537
PCM1	3	1068	0.008337439	0.077198513
CLP1	1	1070	0.009835352	0.087815646
NCOA2	2	1069	0.009835352	0.087815646
CRTC3	3	1068	0.010236951	0.089797819
NR4A3	3	1068	0.011562046	0.099672813
CHD4	4	1067	0.017754619	0.150462875
RGPD3	3	1068	0.0200494	0.159097398
ZFHX3	12	1059	0.020919001	0.159097398
ETV6	4	1067	0.021189593	0.159097398
SEPT5	1	1070	0.021319051	0.159097398
HOXC13	1	1070	0.021319051	0.159097398
IKBKB	1	1070	0.021319051	0.159097398

SALL4	1	1070	0.021319051	0.159097398
MLH1	1	1070	0.021319051	0.159097398
RBM15	1	1070	0.022122331	0.162664196
NCOR1	7	1064	0.022807614	0.165272562
NFE2L2	23	1048	0.030316826	0.21654876
TOP1	1	1070	0.035853933	0.232817745
PRKACA	1	1070	0.035853933	0.232817745
KLF6	1	1070	0.035853933	0.232817745
ARHGEF12	1	1070	0.035853933	0.232817745
EZH2	1	1070	0.035853933	0.232817745
FOXO4	1	1070	0.035853933	0.232817745
RUNX1	1	1070	0.035853933	0.232817745
BCL11B	3	1068	0.037501531	0.240394431
KDM5C	4	1067	0.045565052	0.288386403
NRG1	2	1069	0.0497558	0.310754201
ZNF331	5	1066	0.050342181	0.310754201
NCOA4	6	1065	0.051915111	0.316555556
CD74	1	1070	0.055627619	0.327221287
BCOR	2	1069	0.055627619	0.327221287
ARHGAP26	1	1070	0.055627619	0.327221287
FLNA	2	1069	0.0588221	0.341988955
ANK1	5	1066	0.060665286	0.348651071
TBX3	2	1069	0.064532729	0.366663232
TRIM33	2	1069	0.065731976	0.369280763
KLF4	2	1069	0.069963881	0.388688228
PTPRD	8	1063	0.079655147	0.437665641
MTOR	4	1067	0.085355171	0.463886798
SEPT6	2	1069	0.095487137	0.513371702
CASP8	5	1066	0.09847164	0.523785318
RABEP1	1	1070	0.100644937	0.529710196
CUL3	5	1066	0.104332288	0.543397332
RANBP2	4	1067	0.107417655	0.552826675
ETV1	4	1067	0.108354028	0.552826675
WRN	4	1067	0.111758767	0.564438217
DICER1	4	1067	0.114936008	0.57468004
THRAP3	2	1069	0.116660693	0.575025557
MUC16	57	1014	0.117305214	0.575025557
NUP214	3	1068	0.121909727	0.59179479
MSN	1	1070	0.125277811	0.596561007
BRD3	1	1070	0.125277811	0.596561007
CLIP1	5	1066	0.132496435	0.619210076
CDH10	7	1064	0.132510956	0.619210076
LZTR1	1	1070	0.140401482	0.650006861

TET2	7	1064	0.142622945	0.654233694
SRGAP3	3	1068	0.147876724	0.672166925
FANCC	4	1067	0.150943018	0.67537766
LIFR	3	1068	0.151284596	0.67537766
ZNF479	3	1068	0.157086825	0.695074448
COL3A1	7	1064	0.168117899	0.725957378
MYO5A	3	1068	0.168396458	0.725957378
NOTCH1	27	1044	0.168422112	0.725957378
SMAD3	1	1070	0.170285157	0.727714345
BCLAF1	13	1058	0.175024033	0.735407828
FOXA1	2	1069	0.175027063	0.735407828
CIC	3	1068	0.184959948	0.75075496
KDM6A	8	1063	0.18689477	0.75075496
ERC1	1	1070	0.187129562	0.75075496
GLI1	1	1070	0.187129562	0.75075496
PRPF40B	1	1070	0.187129562	0.75075496
RALGDS	1	1070	0.191473481	0.75075496
AFF3	1	1070	0.191473481	0.75075496
PRF1	1	1070	0.191473481	0.75075496
EPHA3	3	1068	0.19219327	0.75075496
MAP3K13	4	1067	0.202471021	0.773249634
RAD51B	2	1069	0.202763852	0.773249634
EP300	24	1047	0.204121884	0.773249634
IGF2BP2	1	1070	0.206813106	0.773249634
ATP1A1	1	1070	0.206813106	0.773249634
CTNNA2	5	1066	0.207230902	0.773249634
PLCG1	4	1067	0.211125599	0.777546954
PPM1D	3	1068	0.214294965	0.777546954
MB21D2	8	1063	0.223439331	0.777546954
TPM3	3	1068	0.224348347	0.777546954
BUB1B	3	1068	0.225347311	0.777546954
SET	2	1069	0.226154643	0.777546954
PLAG1	4	1067	0.228193572	0.777546954
PRCC	1	1070	0.231603482	0.777546954
ATIC	2	1069	0.232033344	0.777546954
HOOK3	2	1069	0.234981788	0.777546954
KEAP1	2	1069	0.235221462	0.777546954
STAG1	6	1065	0.236030855	0.777546954
DDR2	2	1069	0.236861429	0.777546954
RAP1GDS1	3	1068	0.239955748	0.777546954
SPEN	20	1051	0.240540403	0.777546954
MSH6	2	1069	0.240662693	0.777546954
RNF213	7	1064	0.241563528	0.777546954

BCR	2	1069	0.243132888	0.777546954
CIITA	2	1069	0.243145205	0.777546954
CBL	2	1069	0.243935228	0.777546954
LPP	5	1066	0.246517861	0.777546954
ZBTB16	2	1069	0.249032412	0.777546954
MLF1	2	1069	0.25033191	0.777546954
AKAP9	16	1055	0.250417719	0.777546954
KAT7	2	1069	0.25380449	0.777546954
POT1	5	1066	0.257389146	0.777546954
ERCC5	5	1066	0.261566884	0.777546954
PAX3	1	1070	0.263918441	0.777546954
DDX10	2	1069	0.264114469	0.777546954
POLG	2	1069	0.264637283	0.777546954
PPP2R1A	6	1065	0.276455072	0.777546954
NAB2	2	1069	0.276583967	0.777546954
MLLT6	5	1066	0.278853428	0.777546954
NT5C2	2	1069	0.282939433	0.777546954
EXT2	2	1069	0.287371988	0.777546954
EBF1	2	1069	0.292157559	0.777546954
XPO1	4	1067	0.295935709	0.777546954
CARD11	5	1066	0.297410092	0.777546954
CLTC	4	1067	0.298800824	0.777546954
NBEA	10	1061	0.309095002	0.777546954
SF3B1	6	1065	0.309702908	0.777546954
RAD21	3	1068	0.317046259	0.777546954
CANT1	1	1070	0.317258756	0.777546954
ZRSR2	2	1069	0.324202734	0.777546954
KTN1	10	1061	0.33260545	0.777546954
TET1	4	1067	0.333557036	0.777546954
PIM1	2	1069	0.33657118	0.777546954
PAFAH1B2	1	1070	0.337248505	0.777546954
PIK3R1	1	1070	0.339789994	0.777546954
CALR	2	1069	0.342338277	0.777546954
TLX3	1	1070	0.344881115	0.777546954
PCBP1	1	1070	0.344881115	0.777546954
KAT6A	2	1069	0.349439211	0.777546954
LATS1	7	1064	0.350761105	0.777546954
PIK3CB	2	1069	0.350947326	0.777546954
CDK12	8	1063	0.354544839	0.777546954
PTPRT	5	1066	0.355073964	0.777546954
BCL9	6	1065	0.360513629	0.777546954
TRRAP	9	1062	0.360732307	0.777546954
ZCCHC8	1	1070	0.370271862	0.777546954

JAK1	5	1066	0.370945555	0.777546954
KDR	4	1067	0.371514605	0.777546954
AFF4	4	1067	0.372196744	0.777546954
LSM14A	1	1070	0.373078968	0.777546954
DDIT3	1	1070	0.373078968	0.777546954
B2M	1	1070	0.373078968	0.777546954
TMEM127	1	1070	0.373078968	0.777546954
ARNT	1	1070	0.373078968	0.777546954
EIF1AX	1	1070	0.373078968	0.777546954
TPR	7	1064	0.377071208	0.777546954
PMS2	1	1070	0.380667335	0.777546954
BCL7A	1	1070	0.385922639	0.777546954
TMPRSS2	1	1070	0.385922639	0.777546954
USP8	1	1070	0.385922639	0.777546954
WNK2	1	1070	0.385922639	0.777546954
DAXX	1	1070	0.385922639	0.777546954
PAX5	1	1070	0.385922639	0.777546954
CACNA1D	4	1067	0.38942686	0.777546954
KLK2	1	1070	0.391950155	0.777546954
FLT3	2	1069	0.391950155	0.777546954
RBM10	1	1070	0.391950155	0.777546954
SUFU	1	1070	0.391950155	0.777546954
PICALM	1	1070	0.395300839	0.777546954
NTRK3	1	1070	0.395300839	0.777546954
ASXL2	1	1070	0.395300839	0.777546954
DNM2	1	1070	0.395300839	0.777546954
TNFRSF14	1	1070	0.395300839	0.777546954
MAP3K1	1	1070	0.395300839	0.777546954
PRKAR1A	1	1070	0.395300839	0.777546954
ACSL3	1	1070	0.402894233	0.777546954
PAX8	1	1070	0.407619244	0.777546954
HOXA13	1	1070	0.407619244	0.777546954
HOXC11	1	1070	0.407619244	0.777546954
MACC1	5	1066	0.410496703	0.777546954
NOTCH2	11	1060	0.410799163	0.777546954
FGFR4	1	1070	0.41162043	0.777546954
JUN	1	1070	0.41162043	0.777546954
ABI1	1	1070	0.41162043	0.777546954
CEBPA	1	1070	0.41162043	0.777546954
SDHC	1	1070	0.41162043	0.777546954
CTNND1	3	1068	0.41162043	0.777546954
DDB2	1	1070	0.41162043	0.777546954
ZMYM2	1	1070	0.411623741	0.777546954

SRSF2	1	1070	0.411623741	0.777546954
KIT	1	1070	0.411623741	0.777546954
FUS	1	1070	0.411623741	0.777546954
DCAF12L2	1	1070	0.411623741	0.777546954
TP63	1	1070	0.411623741	0.777546954
CTNNB1	1	1070	0.413219358	0.777546954
H3F3A	1	1070	0.413219358	0.777546954
FANCE	1	1070	0.413219358	0.777546954
DCTN1	1	1070	0.417847639	0.777546954
IL21R	1	1070	0.417847639	0.777546954
CCNE1	1	1070	0.417847639	0.777546954
MAP2K2	1	1070	0.417847639	0.777546954
PBX1	1	1070	0.417847639	0.777546954
SMARCE1	1	1070	0.417847639	0.777546954
NKX2-1	1	1070	0.417847639	0.777546954
CDKN2C	1	1070	0.419287106	0.777546954
ELL	1	1070	0.419287106	0.777546954
GRIN2A	1	1070	0.422021426	0.777546954
NONO	1	1070	0.424618121	0.777546954
LCP1	2	1069	0.424828555	0.777546954
TAL2	1	1070	0.427152138	0.777546954
DROSHA	4	1067	0.427298834	0.777546954
ARID2	5	1066	0.427832338	0.777546954
HIF1A	1	1070	0.430792583	0.777546954
TFEB	1	1070	0.430792583	0.777546954
ERG	1	1070	0.430792583	0.777546954
ARHGEF10L	2	1069	0.430792583	0.777546954
TNFAIP3	1	1070	0.430792583	0.777546954
NBN	4	1067	0.431567538	0.777546954
ELK4	1	1070	0.433111469	0.777546954
WIF1	1	1070	0.433111469	0.777546954
GATA3	1	1070	0.433111469	0.777546954
FBXO11	4	1067	0.434432789	0.777546954
NCOR2	4	1067	0.437479491	0.777546954
CSF3R	1	1070	0.437589284	0.777546954
MDM2	1	1070	0.43794341	0.777546954
REL	1	1070	0.43794341	0.777546954
PDGFRA	5	1066	0.438421227	0.777546954
KIF5B	2	1069	0.439748565	0.777546954
CRTC1	1	1070	0.439748565	0.777546954
MSI2	1	1070	0.439748565	0.777546954
ZEB1	4	1067	0.439828262	0.777546954
EIF3E	1	1070	0.440780193	0.777546954

MGMT	1	1070	0.440780193	0.777546954
PDGFRB	1	1070	0.445739552	0.777546954
TCL1A	1	1070	0.445739552	0.777546954
RPL5	1	1070	0.445739552	0.777546954
FAM47C	2	1069	0.445739552	0.777546954
EXT1	4	1067	0.446311952	0.777546954
MYH9	9	1062	0.448297725	0.778294662
ZMYM3	4	1067	0.451630592	0.77889791
PRRX1	1	1070	0.453302589	0.77889791
ZNF429	7	1064	0.453318584	0.77889791
GOPC	1	1070	0.456719497	0.779594576
FANCA	4	1067	0.456842422	0.779594576
PRKCB	2	1069	0.462520303	0.786599156
CREB1	1	1070	0.468047515	0.790946075
HIST1H3B	4	1067	0.470947908	0.790946075
EIF4A2	4	1067	0.471676579	0.790946075
PDCD1LG2	1	1070	0.473470043	0.790946075
N4BP2	1	1070	0.473470043	0.790946075
BLM	7	1064	0.475118978	0.790946075
ERBB4	9	1062	0.476847163	0.790946075
ATRX	11	1060	0.478208618	0.790946075
PMS1	7	1064	0.48379437	0.790946075
MLLT10	6	1065	0.485826589	0.790946075
ELF3	1	1070	0.48639064	0.790946075
TCF3	2	1069	0.48639064	0.790946075
AFF1	4	1067	0.488145897	0.790946075
BMPR1A	1	1070	0.489147537	0.790946075
PSIP1	1	1070	0.489725063	0.790946075
ALK	6	1065	0.490386566	0.790946075
MED12	4	1067	0.49812781	0.800848568
EGFR	2	1069	0.499985763	0.801259235
SMO	1	1070	0.503021437	0.803548622
ERCC4	3	1068	0.505079379	0.804266527
CDH11	4	1067	0.506968052	0.804711193
FAT1	32	1039	0.511279094	0.805159296
SNX29	1	1070	0.511570598	0.805159296
HERPUD1	1	1070	0.512482662	0.805159296
STRN	4	1067	0.513691631	0.805159296
SPOP	6	1065	0.515882261	0.806066033
KRAS	2	1069	0.518153844	0.807093215
PRDM2	6	1065	0.519939447	0.807359389
CNTNAP2	7	1064	0.533119812	0.823732574
HOXD13	1	1070	0.533778708	0.823732574

TAF15	2	1069	0.536566663	0.825487173
SUZ12	3	1068	0.54365663	0.828819664
RB1	6	1065	0.54562938	0.828819664
FBXW7	24	1047	0.545710023	0.828819664
ROS1	4	1067	0.545785961	0.828819664
DDX3X	3	1068	0.547512112	0.828819664
PTK6	1	1070	0.548678618	0.828819664
HNF1A	1	1070	0.551646762	0.828854388
RHOA	1	1070	0.553530825	0.828854388
CTCF	3	1068	0.553674731	0.828854388
BAZ1A	3	1068	0.558409233	0.832497703
SETD1B	5	1066	0.559438456	0.832497703
SFRP4	3	1068	0.563247194	0.833299565
MYH11	4	1067	0.563310506	0.833299565
TRIP11	9	1062	0.569231895	0.836732482
MECOM	6	1065	0.571176776	0.836732482
MAPK1	1	1070	0.572325018	0.836732482
MAP2K1	1	1070	0.572325018	0.836732482
NIN	6	1065	0.585167128	0.842406111
ARID1A	5	1066	0.587850485	0.842406111
BIRC3	3	1068	0.588975558	0.842406111
CNTRL	4	1067	0.590340364	0.842406111
FCRL4	3	1068	0.591292756	0.842406111
CSMD3	47	1024	0.594211308	0.842406111
PATZ1	1	1070	0.594507495	0.842406111
CYLD	3	1068	0.597763442	0.842406111
HSP90AB1	1	1070	0.598005533	0.842406111
SKI	1	1070	0.598005533	0.842406111
CEP89	6	1065	0.600581072	0.842406111
NUTM1	2	1069	0.602181948	0.842406111
TSC1	8	1063	0.604854415	0.842406111
XPC	1	1070	0.612147297	0.842406111
CTNND2	9	1062	0.614660914	0.842406111
TGFBR2	3	1068	0.616264642	0.842406111
TCF12	2	1069	0.617884855	0.842406111
AR	1	1070	0.617980156	0.842406111
BCL11A	1	1070	0.617980156	0.842406111
FLT4	2	1069	0.617980156	0.842406111
MN1	1	1070	0.617980156	0.842406111
CPEB3	1	1070	0.617980156	0.842406111
SLC34A2	1	1070	0.617980156	0.842406111
STAT5B	1	1070	0.617980156	0.842406111
ELN	1	1070	0.619573817	0.842406111

GRM3	7	1064	0.620010898	0.842406111
ISX	3	1068	0.627678567	0.84591178
SLC45A3	1	1070	0.631164581	0.84591178
ARAF	1	1070	0.631164581	0.84591178
CD209	1	1070	0.631164581	0.84591178
FES	1	1070	0.631164581	0.84591178
HSP90AA1	4	1067	0.632742012	0.84591178
BRD4	2	1069	0.639105527	0.850109539
BTK	1	1070	0.639282373	0.850109539
PREX2	9	1062	0.641477595	0.850766041
PTPRB	8	1063	0.644522616	0.851549431
BARD1	7	1064	0.646119285	0.851549431
ATM	12	1059	0.647177568	0.851549431
MAML2	3	1068	0.649889712	0.852873638
MALT1	1	1070	0.656479784	0.854791386
MAFB	1	1070	0.656479784	0.854791386
CREB3L1	1	1070	0.656479784	0.854791386
NF1	9	1062	0.665524629	0.86090316
CHD2	9	1062	0.666400439	0.86090316
TFE3	3	1068	0.66748641	0.86090316
TSHR	2	1069	0.671642111	0.86090316
EPS15	2	1069	0.675383192	0.86090316
ROBO2	1	1070	0.676064717	0.86090316
FUBP1	5	1066	0.680184012	0.86090316
ECT2L	2	1069	0.680520593	0.86090316
PTPN13	11	1060	0.680794865	0.86090316
GAS7	1	1070	0.681240418	0.86090316
LRP1B	35	1036	0.681932392	0.86090316
NFIB	1	1070	0.683259216	0.86090316
CHEK2	4	1067	0.683557109	0.86090316
LCK	1	1070	0.685648459	0.861367411
FGFR2	6	1065	0.693047987	0.865627577
POLE	4	1067	0.693517773	0.865627577
AXIN2	1	1070	0.694233317	0.865627577
ITGAV	3	1068	0.708051554	0.877466643
MYB	2	1069	0.710285762	0.877466643
GPHN	2	1069	0.71043695	0.877466643
CDH1	3	1068	0.712529485	0.877466643
CNOT3	2	1069	0.714978819	0.877466643
PER1	3	1068	0.715361149	0.877466643
SMC1A	1	1070	0.717767714	0.877466643
TERT	1	1070	0.717767714	0.877466643
KMT2A	9	1062	0.720137661	0.878216659

IKZF1	3	1068	0.724549309	0.87982226
BRIP1	7	1064	0.726277985	0.87982226
DNMT3A	1	1070	0.727984809	0.87982226
PTCH1	4	1067	0.730167574	0.87982226
TP53	85	986	0.730252476	0.87982226
SEPT9	2	1069	0.735077481	0.883033292
CBLB	3	1068	0.741107078	0.883033292
PABPC1	7	1064	0.741889824	0.883033292
RUNX1T1	5	1066	0.743319846	0.883033292
STAT3	3	1068	0.743505507	0.883033292
BRCA2	15	1056	0.743514032	0.883033292
BIRC6	19	1052	0.75408148	0.891253372
FGFR3	1	1070	0.755222452	0.891253372
VAV1	3	1068	0.759218031	0.891253372
DGCR8	4	1067	0.760051681	0.891253372
FAM131B	3	1068	0.760377067	0.891253372
TBL1XR1	3	1068	0.762353385	0.891253372
PML	2	1069	0.762912887	0.891253372
FGFR1OP	2	1069	0.764816805	0.891394878
FANCD2	5	1066	0.770045963	0.892495037
SMAD2	3	1068	0.771046196	0.892495037
PRDM16	4	1067	0.771115712	0.892495037
NCOA1	3	1068	0.772994081	0.892602865
NUTM2D	2	1069	0.775935317	0.893934697
TSC2	2	1069	0.78168764	0.89849154
COL1A1	2	1069	0.789549645	0.904697563
PBRM1	9	1062	0.791613208	0.904697563
LRIG3	2	1069	0.796047627	0.904697563
PTEN	5	1066	0.798945323	0.904697563
FOXP1	2	1069	0.800016498	0.904697563
ARID1B	6	1065	0.800945013	0.904697563
RNF43	4	1067	0.802829558	0.904697563
EML4	5	1066	0.80538992	0.904697563
CLTCL1	2	1069	0.805446425	0.904697563
MSH2	3	1068	0.806930156	0.904697563
CBFA2T3	2	1069	0.806990226	0.904697563
DDX5	3	1068	0.80908736	0.905019418
NTHL1	2	1069	0.820953852	0.916243138
PTPRK	6	1065	0.829467973	0.923683711
BAP1	4	1067	0.845781551	0.932746174
SMARCD1	2	1069	0.847103414	0.932746174
GMPS	2	1069	0.849385008	0.932746174
RET	2	1069	0.84974784	0.932746174

AKT3	6	1065	0.851805425	0.932746174
SOX21	2	1069	0.851907956	0.932746174
IL7R	2	1069	0.853716481	0.932746174
ATR	11	1060	0.854092797	0.932746174
ERBB2	4	1067	0.854395495	0.932746174
ASXL1	2	1069	0.86037945	0.935309117
RSPO3	2	1069	0.862924164	0.935309117
NF2	2	1069	0.863870489	0.935309117
KIAA1549	7	1064	0.864225624	0.935309117
NSD1	7	1064	0.869295051	0.938763554
CREBBP	10	1061	0.873605873	0.939444534
PRDM1	7	1064	0.873683417	0.939444534
KMT2D	29	1042	0.877733365	0.941773997
PDE4DIP	11	1060	0.882284873	0.943180375
MNX1	2	1069	0.884599945	0.943180375
FAT4	14	1057	0.886227252	0.943180375
GNAS	7	1064	0.886589552	0.943180375
SBDS	2	1069	0.890161778	0.944970041
PALB2	3	1068	0.894953389	0.947922022
ELF4	2	1069	0.896734233	0.947922022
FAM135B	9	1062	0.900209293	0.949587862
CCNC	2	1069	0.903876302	0.951448738
APC	2	1069	0.905884949	0.95155982
PTPRC	6	1065	0.911458801	0.954022714
CDKN2A	9	1062	0.912045714	0.954022714
ABL1	2	1069	0.91569438	0.955839645
NCKIPSD	2	1069	0.923214431	0.961681699
POLQ	5	1066	0.931862685	0.966129818
KMT2C	31	1040	0.931924195	0.966129818
NUMA1	4	1067	0.933281404	0.966129818
CARS	2	1069	0.936797098	0.966181418
FNBP1	4	1067	0.939109356	0.966181418
FLCN	3	1068	0.939128338	0.966181418
USP6	6	1065	0.944750007	0.968670516
FAT3	17	1054	0.945422424	0.968670516
NDRG1	2	1069	0.947866162	0.969188305
STAG2	3	1068	0.954042058	0.973512304
JAK2	8	1063	0.965316957	0.980012796
ERCC2	3	1068	0.967212001	0.980012796
ERBB3	2	1069	0.967896771	0.980012796
ZNF521	5	1066	0.968252643	0.980012796
RGS7	4	1067	0.974626399	0.982884437
DCC	9	1062	0.975021362	0.982884437

RAD17	2	1069	0.983616736	0.98955406
EPHA7	9	1062	0.990283722	0.993652357
OMD	4	1067	0.991665053	0.993652357
ITK	3	1068	0.99682699	0.99682699

Table S6d. Association between CCA of genes undering New signature and prognosis.

GeneName	CCA>=0.06	CCA<0.06	pvalue	qvalue(fdr)
**SNX29**	**6**	**1065**	**0.000786359**	**0.010770738**
**CHD2**	**5**	**1066**	**0.005287229**	**0.058288481**
**TET2**	**7**	**1064**	**0.006987285**	**0.073447741**
MYH11	1	1070	1.29E-14	1.16E-12
CCND1	1	1070	1.29E-14	1.16E-12
PRKACA	1	1070	1.29E-14	1.16E-12
SFPQ	1	1070	1.29E-14	1.16E-12
SMARCE1	1	1070	1.29E-14	1.16E-12
FGFR1OP	1	1070	3.34E-10	1.68E-08
STRN	1	1070	3.34E-10	1.68E-08
TCF12	1	1070	3.34E-10	1.68E-08
BRAF	1	1070	3.34E-10	1.68E-08
C15orf65	1	1070	2.07E-09	7.78E-08
SEPT6	1	1070	2.07E-09	7.78E-08
ZCCHC8	1	1070	2.07E-09	7.78E-08
ATIC	1	1070	1.13E-07	2.99E-06
SH3GL1	1	1070	1.13E-07	2.99E-06
TAL1	1	1070	1.13E-07	2.99E-06
CBLB	2	1069	1.13E-07	2.99E-06
FOXO1	1	1070	1.13E-07	2.99E-06
STK11	2	1069	3.81E-06	9.56E-05
NUP214	1	1070	4.54E-06	9.77E-05
EIF1AX	1	1070	4.54E-06	9.77E-05
PCBP1	1	1070	4.54E-06	9.77E-05
EZR	1	1070	0.000333525	0.004863015
KIF5B	1	1070	0.000333525	0.004863015
SPECC1	1	1070	0.000333525	0.004863015
ZNF384	2	1069	0.000333525	0.004863015
CD79B	1	1070	0.000333525	0.004863015
POU2AF1	1	1070	0.000333525	0.004863015
PPM1D	1	1070	0.000333525	0.004863015
KRAS	2	1069	0.000333525	0.004863015
ROS1	1	1070	0.000333525	0.004863015
ELF3	1	1070	0.000333525	0.004863015
PRDM1	3	1068	0.000436678	0.006168081
HNF1A	2	1069	0.000869004	0.011552644
NUTM2D	1	1070	0.002636768	0.02979548

NUTM2B	1	1070	0.002636768	0.02979548
PAX3	1	1070	0.002636768	0.02979548
WAS	1	1070	0.002636768	0.02979548
CDH1	1	1070	0.002636768	0.02979548
PPARG	1	1070	0.002636768	0.02979548
ACSL6	2	1069	0.005950193	0.064035408
TRAF7	2	1069	0.007485247	0.0768939
H3F3B	2	1069	0.010869078	0.109173846
LEF1	2	1069	0.013429288	0.131957356
HOXD13	1	1070	0.021319051	0.20502577
STAT3	4	1067	0.026207573	0.246787977
RNF213	7	1064	0.031370251	0.28358707
KAT6B	2	1069	0.031174637	0.28358707
CASP8	4	1067	0.033933451	0.295810416
POLQ	3	1068	0.034031287	0.295810416
FANCC	1	1070	0.035853933	0.300110696
ARHGEF12	3	1068	0.035853933	0.300110696
NIN	3	1068	0.040413158	0.326191917
CAMTA1	5	1066	0.040246197	0.326191917
AKAP9	8	1063	0.046434361	0.357863773
CANT1	2	1069	0.047504041	0.357863773
REL	2	1069	0.045289874	0.357863773
ARID2	6	1065	0.046901746	0.357863773
KDM6A	7	1064	0.048893219	0.362290739
IDH1	2	1069	0.05229001	0.371648574
MUC16	59	1012	0.052334544	0.371648574
POLE	3	1068	0.052622807	0.371648574
CREBBP	9	1062	0.054797955	0.375278861
GATA1	1	1070	0.055627619	0.375278861
STAT5B	1	1070	0.055627619	0.375278861
MLLT10	2	1069	0.058281634	0.387401448
ATR	5	1066	0.06001969	0.393172462
MN1	2	1069	0.062715459	0.402514213
PIK3CA	11	1060	0.064117308	0.402514213
IKZF1	3	1068	0.063237388	0.402514213
KIAA1549	3	1068	0.07656776	0.441864814
MNX1	1	1070	0.07656776	0.441864814
DDX5	1	1070	0.07656776	0.441864814
FLT4	1	1070	0.07656776	0.441864814
OLIG2	1	1070	0.078091237	0.441864814
SALL4	1	1070	0.07656776	0.441864814
BCORL1	2	1069	0.078206162	0.441864814
BTK	1	1070	0.07656776	0.441864814

PTPRT	5	1066	0.081606631	0.455385149
HIST1H3B	4	1067	0.084149567	0.46384883
COL2A1	5	1066	0.095697881	0.521149906
NUMA1	5	1066	0.099090176	0.533199517
GRIN2A	4	1067	0.100719466	0.535590572
TFG	2	1069	0.105303911	0.547096182
KMT2A	4	1067	0.104300696	0.547096182
EGFR	6	1065	0.110258314	0.562723917
MGMT	4	1067	0.110801833	0.562723917
KMT2C	15	1056	0.112455883	0.564778434
JAK2	5	1066	0.115090374	0.571657679
CNTNAP2	5	1066	0.118148879	0.580470578
ARID1B	4	1067	0.121330337	0.589691529
BCL3	1	1070	0.125277811	0.602399689
FNBP1	2	1069	0.141503372	0.657849425
PDE4DIP	17	1054	0.141229468	0.657849425
H3F3A	3	1068	0.147278056	0.657849425
JAK3	2	1069	0.149286715	0.657849425
NTRK3	3	1068	0.149908165	0.657849425
TRRAP	5	1066	0.141657557	0.657849425
AMER1	2	1069	0.146647612	0.657849425
DNMT3A	3	1068	0.148724135	0.657849425
FBXW7	22	1049	0.149530995	0.657849425
TET1	3	1068	0.154893292	0.673190078
AFF1	1	1070	0.417847639	0.704002286
ALDH2	1	1070	0.337248505	0.704002286
ASPSCR1	1	1070	0.370271862	0.704002286
CLIP1	2	1069	0.313238488	0.704002286
COL1A1	5	1066	0.386825913	0.704002286
CRTC3	1	1070	0.411623741	0.704002286
ERC1	1	1070	0.433111469	0.704002286
GMPS	2	1069	0.228796622	0.704002286
KTN1	2	1069	0.194826302	0.704002286
LIFR	2	1069	0.245154168	0.704002286
LSM14A	1	1070	0.433111469	0.704002286
NACA	1	1070	0.411623741	0.704002286
NSD1	2	1069	0.271986492	0.704002286
PAX7	1	1070	0.439748565	0.704002286
PRCC	2	1069	0.221234376	0.704002286
PAFAH1B2	1	1070	0.337248505	0.704002286
RBM15	1	1070	0.433111469	0.704002286
SEPT5	2	1069	0.259943991	0.704002286
SRGAP3	1	1070	0.391950155	0.704002286

SLC45A3	1	1070	0.391950155	0.704002286
TOP1	2	1069	0.257253975	0.704002286
TPR	7	1064	0.429094484	0.704002286
VAV1	1	1070	0.411623741	0.704002286
VTI1A	2	1069	0.320318855	0.704002286
ZMYM2	1	1070	0.440780193	0.704002286
NT5C2	3	1068	0.286867472	0.704002286
ACVR1	1	1070	0.411623741	0.704002286
AFF3	5	1066	0.406090572	0.704002286
AFF4	1	1070	0.430792583	0.704002286
ALK	5	1066	0.35651495	0.704002286
BCL11A	4	1067	0.274685812	0.704002286
BCL2L12	1	1070	0.411623741	0.704002286
BRD3	1	1070	0.385922639	0.704002286
ABL2	4	1067	0.438066617	0.704002286
CARD11	1	1070	0.411623741	0.704002286
CTNNB1	2	1069	0.299766649	0.704002286
CD28	1	1070	0.440780193	0.704002286
CD79A	1	1070	0.440780193	0.704002286
CHD4	5	1066	0.219949866	0.704002286
DGCR8	1	1070	0.407619244	0.704002286
DDR2	2	1069	0.33657118	0.704002286
ERBB3	1	1070	0.430792583	0.704002286
FCRL4	3	1068	0.186793859	0.704002286
FOXA1	1	1070	0.411623741	0.704002286
FOXR1	1	1070	0.263918441	0.704002286
GATA2	1	1070	0.430792583	0.704002286
GNAQ	1	1070	0.430792583	0.704002286
HLF	1	1070	0.417847639	0.704002286
HNRNPA2B1	1	1070	0.373078968	0.704002286
HOXC13	1	1070	0.411623741	0.704002286
HIF1A	3	1068	0.434958744	0.704002286
IL7R	2	1069	0.216162919	0.704002286
JUN	1	1070	0.440780193	0.704002286
LPP	4	1067	0.382468803	0.704002286
KAT7	1	1070	0.391950155	0.704002286
MAML2	2	1069	0.36761093	0.704002286
MDM4	1	1070	0.170285157	0.704002286
MTOR	5	1066	0.41369727	0.704002286
MET	3	1068	0.176387689	0.704002286
MAP2K2	1	1070	0.417847639	0.704002286
MALT1	1	1070	0.437589284	0.704002286
NFATC2	1	1070	0.424618121	0.704002286

NCOA2	2	1069	0.287800974	0.704002286
NPM1	1	1070	0.417847639	0.704002286
PLAG1	3	1068	0.268044052	0.704002286
PRDM16	3	1068	0.210775245	0.704002286
RAC1	1	1070	0.268705878	0.704002286
SRSF2	1	1070	0.411623741	0.704002286
SGK1	1	1070	0.411623741	0.704002286
SETDB1	1	1070	0.391950155	0.704002286
SIX1	1	1070	0.411623741	0.704002286
SIX2	1	1070	0.438674524	0.704002286
SMO	3	1068	0.329281044	0.704002286
SND1	1	1070	0.430792583	0.704002286
SSX1	1	1070	0.430792583	0.704002286
TAF15	2	1069	0.323854722	0.704002286
TCL1A	1	1070	0.400378314	0.704002286
ABL1	3	1068	0.188947353	0.704002286
ERBB2	1	1070	0.41162043	0.704002286
ERG	2	1069	0.249032412	0.704002286
MAF	1	1070	0.413219358	0.704002286
MYB	2	1069	0.413219358	0.704002286
MYCL	1	1070	0.411623741	0.704002286
ACVR2A	2	1069	0.241529589	0.704002286
ASXL1	5	1066	0.297602403	0.704002286
ASXL2	1	1070	0.373078968	0.704002286
ATM	3	1068	0.40003411	0.704002286
ATP2B3	2	1069	0.25033191	0.704002286
AXIN1	4	1067	0.43306719	0.704002286
BAX	2	1069	0.385922639	0.704002286
B2M	1	1070	0.373078968	0.704002286
CTCF	1	1070	0.206813106	0.704002286
CNOT3	1	1070	0.407619244	0.704002286
CD274	1	1070	0.395300839	0.704002286
CIITA	1	1070	0.411623741	0.704002286
CCDC6	1	1070	0.419287106	0.704002286
CBFB	1	1070	0.430792583	0.704002286
CUL3	4	1067	0.283038118	0.704002286
CDK12	8	1063	0.372106951	0.704002286
CDKN1B	1	1070	0.411623741	0.704002286
CDKN2C	1	1070	0.419287106	0.704002286
CARS	1	1070	0.419287106	0.704002286
DICER1	4	1067	0.425231485	0.704002286
EBF1	5	1066	0.310338131	0.704002286
ERCC2	2	1069	0.334664011	0.704002286

ERCC5	2	1069	0.280824065	0.704002286
CYLD	4	1067	0.300317302	0.704002286
FANCA	1	1070	0.422021426	0.704002286
FANCF	1	1070	0.440780193	0.704002286
KEAP1	6	1065	0.439061579	0.704002286
LATS1	2	1069	0.39728041	0.704002286
LRIG3	2	1069	0.270670967	0.704002286
LZTR1	3	1068	0.20216345	0.704002286
MEN1	1	1070	0.187129562	0.704002286
MSH6	3	1068	0.24564132	0.704002286
MYH9	4	1067	0.356824514	0.704002286
NRG1	1	1070	0.191473481	0.704002286
NF2	3	1068	0.352108567	0.704002286
NAB2	2	1069	0.254352404	0.704002286
NCOA4	2	1069	0.311021725	0.704002286
PHOX2B	2	1069	0.307187883	0.704002286
PRF1	3	1068	0.34078336	0.704002286
PER1	1	1070	0.231603482	0.704002286
PIK3R1	1	1070	0.407619244	0.704002286
PRDM2	1	1070	0.400378314	0.704002286
PPP2R1A	5	1066	0.401245792	0.704002286
PTPRB	5	1066	0.288282081	0.704002286
RB1	9	1062	0.246173764	0.704002286
ARHGEF10	2	1069	0.380809587	0.704002286
RPL5	1	1070	0.385922639	0.704002286
RNF43	2	1069	0.343169563	0.704002286
RBM10	2	1069	0.233197604	0.704002286
ROBO2	1	1070	0.395300839	0.704002286
SETD1B	6	1065	0.414867248	0.704002286
SETD2	5	1066	0.38322907	0.704002286
SH2B3	1	1070	0.391950155	0.704002286
SMAD3	1	1070	0.268705878	0.704002286
SMC1A	1	1070	0.407619244	0.704002286
SDHA	1	1070	0.430792583	0.704002286
SUFU	1	1070	0.422021426	0.704002286
SMARCD1	1	1070	0.419287106	0.704002286
TRIM33	2	1069	0.439748565	0.704002286
TSC1	1	1070	0.411623741	0.704002286
USP44	1	1070	0.437589284	0.704002286
WRN	3	1068	0.25015524	0.704002286
WNK2	2	1069	0.238827651	0.704002286
ZNRF3	1	1070	0.170285157	0.704002286
ZMYM3	2	1069	0.25872592	0.704002286

PATZ1	1	1070	0.41162043	0.704002286
ANK1	2	1069	0.261330953	0.704002286
ARNT	1	1070	0.339789994	0.704002286
ATP1A1	1	1070	0.395300839	0.704002286
BIRC3	1	1070	0.440780193	0.704002286
BCLAF1	9	1062	0.381887311	0.704002286
CBL	1	1070	0.407619244	0.704002286
CD209	2	1069	0.257544622	0.704002286
CNBD1	2	1069	0.28047518	0.704002286
CDKN1A	1	1070	0.440780193	0.704002286
DAXX	2	1069	0.257910577	0.704002286
EPAS1	6	1065	0.407522128	0.704002286
FAM47C	4	1067	0.418637031	0.704002286
FES	1	1070	0.400378314	0.704002286
FKBP9	1	1070	0.231603482	0.704002286
GATA3	1	1070	0.422021426	0.704002286
ISX	1	1070	0.427152138	0.704002286
MAP3K1	1	1070	0.422021426	0.704002286
MAP3K13	1	1070	0.391950155	0.704002286
NKX2-1	1	1070	0.422021426	0.704002286
NFKB2	1	1070	0.407619244	0.704002286
PMS1	1	1070	0.391950155	0.704002286
PRKCB	2	1069	0.302943739	0.704002286
RUNX1T1	5	1066	0.323824983	0.704002286
TBX3	4	1067	0.341168102	0.704002286
TERT	1	1070	0.439748565	0.704002286
TCF3	2	1069	0.247918536	0.704002286
TP53	265	806	0.383187569	0.704002286
TP63	3	1068	0.216212138	0.704002286
WT1	1	1070	0.370271862	0.704002286
ZNF429	1	1070	0.407619244	0.704002286
BCR	5	1066	0.459694213	0.704345032
EML4	1	1070	0.458875868	0.704345032
GPHN	2	1069	0.453302589	0.704345032
GOLGA5	1	1070	0.453302589	0.704345032
ACKR3	1	1070	0.456719497	0.704345032
KDM5A	1	1070	0.453302589	0.704345032
MACC1	1	1070	0.453302589	0.704345032
NR4A3	2	1069	0.458561783	0.704345032
APOBEC3B	1	1070	0.456719497	0.704345032
BMP5	1	1070	0.453302589	0.704345032
EZH2	1	1070	0.453302589	0.704345032
JAK1	1	1070	0.445739552	0.704345032

AR	2	1069	0.464286921	0.705950855
MUC4	2	1069	0.463485221	0.705950855
SPEN	11	1060	0.465427776	0.705950855
NTRK1	1	1070	0.468047515	0.707550089
PML	1	1070	0.472569172	0.712004219
PPFIBP1	2	1069	0.476550498	0.715617358
CDH11	2	1069	0.483425555	0.723537585
TGFBR2	6	1065	0.497326382	0.74188622
USP6	7	1064	0.501149984	0.745130898
FAM131B	1	1070	0.511570598	0.749139223
CTNND2	9	1062	0.508136577	0.749139223
CXCR4	1	1070	0.512482662	0.749139223
TNC	3	1068	0.508978623	0.749139223
KDM5C	3	1068	0.513790175	0.749139223
DCAF12L2	1	1070	0.511570598	0.749139223
ACSL3	1	1070	0.521696503	0.754205952
BRCA2	3	1068	0.523939533	0.754205952
MED12	3	1068	0.523477311	0.754205952
TBL1XR1	3	1068	0.51993447	0.754205952
DNAJB1	3	1068	0.527435099	0.754432483
SMAD4	3	1068	0.526157823	0.754432483
ZNF479	1	1070	0.533778708	0.761097716
MLH1	3	1068	0.542025223	0.770425789
PALB2	8	1063	0.548676349	0.777434827
FANCD2	6	1065	0.550758811	0.777946821
PTPRD	11	1060	0.555175323	0.781742199
BIRC6	3	1068	0.563205261	0.790586267
BLM	4	1067	0.566752725	0.793102884
TMPRSS2	1	1070	0.572325018	0.798428728
DCC	7	1064	0.578117524	0.804028064
HSP90AB1	1	1070	0.598005533	0.806861197
MSN	1	1070	0.594507495	0.806861197
NONO	1	1070	0.587849196	0.806861197
RPN1	3	1068	0.584979266	0.806861197
GRM3	4	1067	0.59020303	0.806861197
PREX2	5	1066	0.588417109	0.806861197
WWTR1	1	1070	0.598005533	0.806861197
FUS	1	1070	0.598005533	0.806861197
CBLC	2	1069	0.594507495	0.806861197
ELF4	1	1070	0.594507495	0.806861197
PCM1	2	1069	0.607001528	0.808188972
XPO1	1	1070	0.609717786	0.808188972
LARP4B	2	1069	0.602316251	0.808188972

SLC34A2	1	1070	0.609717786	0.808188972
RGS7	7	1064	0.602795732	0.808188972
TRIM24	3	1068	0.605655558	0.808188972
ELN	1	1070	0.619573817	0.81014949
THRAP3	1	1070	0.617980156	0.81014949
RET	1	1070	0.619573817	0.81014949
FH	1	1070	0.617980156	0.81014949
LRP1B	39	1032	0.621949814	0.81014949
PTPRK	3	1068	0.621951046	0.81014949
ZFHX3	12	1059	0.6244297	0.811040874
CRTC1	1	1070	0.631164581	0.815103974
KLF4	1	1070	0.631164581	0.815103974
NCOR1	8	1063	0.635950185	0.818944397
S100A7	1	1070	0.648840211	0.830809562
SS18L1	1	1070	0.648840211	0.830809562
CASP3	1	1070	0.652057651	0.832570786
MAFB	1	1070	0.656479784	0.835855951
NCOR2	4	1067	0.659972463	0.837942565
FLT3	2	1069	0.66672389	0.844143412
MAX	2	1069	0.674743508	0.84997828
FLNA	5	1066	0.675093368	0.84997828
RABEP1	1	1070	0.681240418	0.850634697
PTPN11	2	1069	0.683142467	0.850634697
PTCH1	6	1065	0.679646564	0.850634697
FAM135B	22	1049	0.682513996	0.850634697
CEP89	2	1069	0.693595474	0.851751012
RALGDS	2	1069	0.695618819	0.851751012
GLI1	3	1068	0.707054185	0.851751012
PDGFRB	2	1069	0.691274519	0.851751012
KDR	3	1068	0.699460031	0.851751012
CLTCL1	3	1068	0.708115101	0.851751012
PTEN	8	1063	0.706962823	0.851751012
PTPN13	5	1066	0.694750988	0.851751012
PTPRC	2	1069	0.698206282	0.851751012
SFRP4	4	1067	0.70853624	0.851751012
ZBTB16	3	1068	0.703383612	0.851751012
ERBB4	9	1062	0.698489886	0.851751012
ESR1	1	1070	0.701909	0.851751012
FBLN2	3	1068	0.727878229	0.87268159
FAT1	23	1048	0.731932867	0.875221312
CNTRL	3	1068	0.737526656	0.877268548
ATRX	5	1066	0.736029116	0.877268548
BRD4	2	1069	0.750570818	0.89044097

FGFR4	1	1070	0.759750613	0.89897193
MAPK1	3	1068	0.766935832	0.905104428
TFRC	2	1069	0.770737806	0.905761868
PAX5	2	1069	0.771500706	0.905761868
ITGAV	2	1069	0.776398907	0.909151052
TLX1	2	1069	0.784937837	0.916027142
ETV6	3	1068	0.786324184	0.916027142
ARAF	5	1066	0.795364863	0.916186866
ZEB1	3	1068	0.798656789	0.916186866
EP300	17	1054	0.795385873	0.916186866
ARID1A	7	1064	0.793457023	0.916186866
GPC5	3	1068	0.798860504	0.916186866
NBN	3	1068	0.789391081	0.916186866
RANBP2	5	1066	0.802961368	0.916186866
EPHA3	6	1065	0.804150222	0.916186866
KMT2D	31	1040	0.804703951	0.916186866
KAT6A	4	1067	0.816910477	0.919475437
NUTM1	2	1069	0.827922304	0.919475437
USP8	2	1069	0.818361989	0.919475437
CHEK2	4	1067	0.816403182	0.919475437
CDKN2A	27	1044	0.834037454	0.919475437
DDX10	2	1069	0.833465249	0.919475437
ERCC4	3	1068	0.826807738	0.919475437
FAT4	13	1058	0.827184079	0.919475437
ARHGEF10L	2	1069	0.815228737	0.919475437
NBEA	11	1060	0.833211803	0.919475437
NOTCH1	51	1020	0.828004738	0.919475437
NOTCH2	14	1057	0.809836124	0.919475437
PABPC1	2	1069	0.830467487	0.919475437
AKT3	2	1069	0.851193376	0.935104313
XPC	2	1069	0.852351719	0.935104313
COL3A1	6	1065	0.859988346	0.941197899
NFE2L2	10	1061	0.874049932	0.954276738
GAS7	5	1066	0.889969822	0.962359712
SEPT9	2	1069	0.888618238	0.962359712
PIK3CB	6	1065	0.887416718	0.962359712
ARHGAP5	5	1066	0.885274468	0.962359712
SF3B1	1	1070	0.896764362	0.964246297
PTPN6	1	1070	0.896764362	0.964246297
RAD21	2	1069	0.898114361	0.964246297
MLLT6	3	1068	0.908069462	0.968327104
EWSR1	2	1069	0.917427383	0.968327104
ZNF521	6	1065	0.915685132	0.968327104

ABI1	2	1069	0.913640495	0.968327104
CDH10	12	1059	0.904536057	0.968327104
N4BP2	4	1067	0.911140086	0.968327104
PBRM1	5	1066	0.919053823	0.968327104
FAT3	18	1053	0.917279179	0.968327104
A1CF	4	1067	0.93829061	0.986296176
IGF2BP2	3	1068	0.943261401	0.989220773
GNAS	6	1065	0.951693341	0.989686596
BAP1	5	1066	0.95246387	0.989686596
BRCA1	3	1068	0.947841484	0.989686596
RSPO2	2	1069	0.951704723	0.989686596
TRIP11	4	1067	0.957121864	0.990117922
STAG1	4	1067	0.957260027	0.990117922
SPOP	2	1069	0.959618628	0.990291369
CTNNA2	7	1064	0.972102078	0.990675125
MECOM	2	1069	0.975072316	0.990675125
UBR5	11	1060	0.970777388	0.990675125
APC	5	1066	0.97752457	0.990675125
BARD1	4	1067	0.962886215	0.990675125
BAZ1A	4	1067	0.975678732	0.990675125
EPHA7	3	1068	0.975678732	0.990675125
FOXO4	2	1069	0.96595224	0.990675125
RGPD3	4	1067	0.980393729	0.991360102
PICALM	2	1069	0.990905111	0.995309134
SET	2	1069	0.988210821	0.995309134
TNFRSF17	2	1069	0.990147493	0.995309134
SETBP1	2	1069	0.995315754	0.995315754
CSMD3	47	1024	0.993947071	0.995315754

Table S6e. Association between CCA of genes undering SBS3* signature and prognosis.

**GeneName**	**CCA>=0.06**	**CCA<0.06**	**pvalue**	**qvalue(fdr)**
**CDH10**	**9**	**1062**	**9.25E-06**	**0.000200013**
TCEA1	1	1070	1.29E-14	8.35E-13
FGFR1	1	1070	1.29E-14	8.35E-13
IDH1	1	1070	1.29E-14	8.35E-13
LCK	1	1070	1.29E-14	8.35E-13
CDH1	1	1070	1.29E-14	8.35E-13
FANCG	1	1070	1.29E-14	8.35E-13
FHIT	1	1070	1.29E-14	8.35E-13
AKT3	1	1070	9.65E-14	5.48E-12
CIITA	1	1070	3.34E-10	1.38E-08
CBFB	1	1070	3.34E-10	1.38E-08
STAG2	2	1069	3.34E-10	1.38E-08
C15orf65	1	1070	2.07E-09	7.21E-08

MYCL	1	1070	2.07E-09	7.21E-08
FOXO1	1	1070	1.13E-07	3.65E-06
ACSL6	2	1069	8.30E-07	2.51E-05
TSHR	1	1070	3.27E-06	8.74E-05
ATP2B3	1	1070	3.27E-06	8.74E-05
CLP1	2	1069	3.96E-06	9.99E-05
SNX29	1	1070	6.20E-06	0.000140637
PTPN11	2	1069	6.20E-06	0.000140637
LARP4B	1	1070	0.000333525	0.006583501
TRAF7	1	1070	0.000333525	0.006583501
CUL3	3	1068	0.000538469	0.010186031
STK11	1	1070	0.000711264	0.012916559
DGCR8	2	1069	0.000904485	0.015793697
RGS7	4	1067	0.002382054	0.040053801
MEN1	1	1070	0.002636768	0.042753312
MUC4	1	1070	0.002852382	0.044654538
KAT6A	4	1067	0.003287093	0.049744675
ARID1B	2	1069	0.006288799	0.092100474
H3F3B	3	1068	0.008054573	0.11427425
MSH2	1	1070	0.009835352	0.131330881
ECT2L	1	1070	0.009835352	0.131330881
TFG	1	1070	0.010869078	0.140987464
ZCCHC8	2	1069	0.012294021	0.155041263
TBX3	5	1066	0.016892211	0.207271995
RBM15	1	1070	0.022122331	0.244964344
SEPT5	1	1070	0.021319051	0.244964344
SEPT9	1	1070	0.021319051	0.244964344
VHL	1	1070	0.022122331	0.244964344
SLC34A2	2	1069	0.023374411	0.252666251
POU2AF1	1	1070	0.035853933	0.339118447
BRIP1	4	1067	0.035566622	0.339118447
FANCE	2	1069	0.034415831	0.339118447
POLE	1	1070	0.035853933	0.339118447
ARHGEF12	1	1070	0.035853933	0.339118447
ESR1	1	1070	0.035853933	0.339118447
AFF3	6	1065	0.03983195	0.369055206
NUTM1	2	1069	0.042923977	0.389749708
NFE2L2	16	1055	0.043851506	0.390364389
BIRC6	9	1062	0.046538252	0.406314737
TPR	3	1068	0.048688089	0.417064011
HSP90AA1	2	1069	0.055717761	0.438238594
CD74	1	1070	0.055627619	0.438238594
ETV5	1	1070	0.055627619	0.438238594

SPEN	10	1061	0.055986428	0.438238594
GATA1	1	1070	0.055627619	0.438238594
PAX7	3	1068	0.058788232	0.452370461
CSMD3	61	1010	0.061743718	0.467194133
ATM	6	1065	0.06314668	0.469976928
ALK	4	1067	0.072196365	0.520272216
PIK3CA	7	1064	0.071491212	0.520272216
SS18	2	1069	0.074111832	0.52573081
OLIG2	1	1070	0.078091237	0.539608217
EXT2	1	1070	0.079633812	0.539608217
TNFAIP3	1	1070	0.079633812	0.539608217
CASP8	7	1064	0.089558601	0.593591676
KAT6B	6	1065	0.090215475	0.593591676
NRAS	1	1070	0.093904564	0.600460172
SRC	1	1070	0.093904564	0.600460172
EPS15	1	1070	0.105818107	0.637009749
FAT4	14	1057	0.10495003	0.637009749
FEN1	1	1070	0.105818107	0.637009749
RANBP2	4	1067	0.106590668	0.637009749
KLF4	3	1068	0.106635993	0.637009749
CTNNA2	6	1065	0.111382336	0.656721827
BRD4	3	1068	0.117582114	0.684388202
BCL3	1	1070	0.125277811	0.685254535
BRD3	1	1070	0.125277811	0.685254535
IL6ST	1	1070	0.125277811	0.685254535
AMER1	1	1070	0.125277811	0.685254535
ATP1A1	1	1070	0.125277811	0.685254535
CHD4	3	1068	0.129363683	0.699179904
PCM1	3	1068	0.13477864	0.7198765
FH	4	1067	0.136656791	0.721420733
IL2	1	1070	0.140401482	0.724344009
ERCC5	2	1069	0.140401482	0.724344009
MACC1	4	1067	0.147526017	0.736008918
GRIN2A	3	1068	0.146122666	0.736008918
TP53	208	863	0.147329354	0.736008918
COL3A1	10	1061	0.174887815	0.743918987
DNAJB1	2	1069	0.223500184	0.743918987
EML4	2	1069	0.209128832	0.743918987
ELN	2	1069	0.285319133	0.743918987
EIF4A2	2	1069	0.234900111	0.743918987
GOLGA5	1	1070	0.417847639	0.743918987
HERPUD1	1	1070	0.440780193	0.743918987
HOOK3	1	1070	0.395300839	0.743918987

IL21R	1	1070	0.417847639	0.743918987
JAZF1	1	1070	0.385922639	0.743918987
KLK2	1	1070	0.391950155	0.743918987
KIAA1549	6	1065	0.181239552	0.743918987
HLA-A	1	1070	0.417847639	0.743918987
MNX1	1	1070	0.391950155	0.743918987
MLLT3	1	1070	0.339789994	0.743918987
MLLT6	4	1067	0.303032196	0.743918987
MYO5A	4	1067	0.156706384	0.743918987
NCKIPSD	2	1069	0.430792583	0.743918987
NIN	2	1069	0.247529909	0.743918987
NUMA1	2	1069	0.306828922	0.743918987
NCOA1	1	1070	0.402894233	0.743918987
OMD	1	1070	0.287800974	0.743918987
PAX8	1	1070	0.437589284	0.743918987
PRCC	1	1070	0.231603482	0.743918987
PAFAH1B2	1	1070	0.337248505	0.743918987
PWWP2A	1	1070	0.337248505	0.743918987
RALGDS	1	1070	0.391950155	0.743918987
RNF213	6	1065	0.287645172	0.743918987
SLC45A3	1	1070	0.411623741	0.743918987
TRIP11	5	1066	0.390313926	0.743918987
TOP1	1	1070	0.416309934	0.743918987
TMPRSS2	2	1069	0.315454632	0.743918987
TPM4	1	1070	0.395300839	0.743918987
ZMYM2	1	1070	0.422021426	0.743918987
AR	5	1066	0.202934262	0.743918987
BCL9	4	1067	0.38815773	0.743918987
CARD11	2	1069	0.245154168	0.743918987
CTNNB1	2	1069	0.327642253	0.743918987
CTNND2	11	1060	0.309247835	0.743918987
CCR4	1	1070	0.385922639	0.743918987
CD28	1	1070	0.440780193	0.743918987
CSF1R	3	1068	0.420004431	0.743918987
XPO1	4	1067	0.368569102	0.743918987
FCGR2B	1	1070	0.314749166	0.743918987
FGFR2	1	1070	0.385922639	0.743918987
FGFR4	2	1069	0.175571893	0.743918987
FOXA1	1	1070	0.411623741	0.743918987
GATA2	2	1069	0.264806643	0.743918987
GRM3	8	1063	0.37064607	0.743918987
GNAQ	1	1070	0.430792583	0.743918987
H3F3A	2	1069	0.217749627	0.743918987

HNRNPA2B1	2	1069	0.235074203	0.743918987
HOXA13	1	1070	0.407619244	0.743918987
HOXC13	1	1070	0.411623741	0.743918987
HIF1A	2	1069	0.344989579	0.743918987
JAK2	4	1067	0.427550864	0.743918987
JUN	1	1070	0.440780193	0.743918987
KDM5A	2	1069	0.251329443	0.743918987
MDM4	1	1070	0.268705878	0.743918987
MECOM	1	1070	0.385922639	0.743918987
MAP2K2	1	1070	0.417847639	0.743918987
MALT1	2	1069	0.394058235	0.743918987
MLLT10	2	1069	0.260298756	0.743918987
NTRK3	2	1069	0.272521984	0.743918987
PDGFRB	1	1070	0.433111469	0.743918987
PLAG1	2	1069	0.206813106	0.743918987
PRDM16	1	1070	0.391950155	0.743918987
RARA	1	1070	0.402894233	0.743918987
RSPO3	1	1070	0.395300839	0.743918987
SRSF2	1	1070	0.411623741	0.743918987
SGK1	1	1070	0.411623741	0.743918987
SET	1	1070	0.170285157	0.743918987
STAT3	3	1068	0.343216276	0.743918987
SF3B1	3	1068	0.383286988	0.743918987
SSX1	1	1070	0.430792583	0.743918987
TLX3	1	1070	0.391950155	0.743918987
TFEB	3	1068	0.155476796	0.743918987
TNFRSF17	1	1070	0.373078968	0.743918987
USP6	3	1068	0.204352826	0.743918987
ABL1	3	1068	0.204515773	0.743918987
ERG	1	1070	0.411623741	0.743918987
KRAS	1	1070	0.337248505	0.743918987
KIT	2	1069	0.272671285	0.743918987
REL	1	1070	0.411623741	0.743918987
ROS1	3	1068	0.265073228	0.743918987
ZEB1	3	1068	0.280609326	0.743918987
ASXL1	5	1066	0.259939691	0.743918987
ATRX	1	1070	0.407619244	0.743918987
ARID1A	5	1066	0.328419436	0.743918987
BLM	2	1069	0.274159386	0.743918987
BUB1B	2	1069	0.407619244	0.743918987
CREB3L1	2	1069	0.417847639	0.743918987
CEBPA	1	1070	0.41162043	0.743918987
CCDC6	1	1070	0.268705878	0.743918987

CNTNAP2	10	1061	0.214878122	0.743918987
CDK12	6	1065	0.304208418	0.743918987
DNMT3A	1	1070	0.411623741	0.743918987
DNM2	1	1070	0.385922639	0.743918987
MLH1	1	1070	0.411623741	0.743918987
ELL	1	1070	0.419287106	0.743918987
ETV6	2	1069	0.430547421	0.743918987
ERCC2	1	1070	0.413219358	0.743918987
ERCC4	3	1068	0.181410321	0.743918987
BRCA1	3	1068	0.347806428	0.743918987
BRCA2	8	1063	0.329096765	0.743918987
FANCC	2	1069	0.292723845	0.743918987
FANCD2	4	1067	0.311077763	0.743918987
FAT1	19	1052	0.38570386	0.743918987
FBXO11	1	1070	0.385922639	0.743918987
GPC5	3	1068	0.184273045	0.743918987
IKZF1	3	1068	0.380692078	0.743918987
IGF2BP2	2	1069	0.348229847	0.743918987
KEAP1	7	1064	0.387018721	0.743918987
LRP1B	34	1037	0.152786669	0.743918987
LZTR1	1	1070	0.233915093	0.743918987
EXT1	2	1069	0.244293005	0.743918987
MSH6	3	1068	0.152562976	0.743918987
NRG1	2	1069	0.235141718	0.743918987
NF1	6	1065	0.263068786	0.743918987
NAB2	1	1070	0.419287106	0.743918987
NBN	3	1068	0.338018666	0.743918987
NDRG1	1	1070	0.430792583	0.743918987
NCOA4	1	1070	0.395300839	0.743918987
NCOR1	6	1065	0.374071982	0.743918987
PHOX2B	4	1067	0.182193707	0.743918987
PPARG	1	1070	0.395300839	0.743918987
PTEN	6	1065	0.236597882	0.743918987
PIK3R1	1	1070	0.373078968	0.743918987
PBRM1	3	1068	0.210047255	0.743918987
PRDM1	3	1068	0.411546419	0.743918987
PML	2	1069	0.259903199	0.743918987
PPP2R1A	2	1069	0.440780193	0.743918987
PTPN13	2	1069	0.225478423	0.743918987
RNF43	1	1070	0.337248505	0.743918987
ROBO2	1	1070	0.437589284	0.743918987
SFRP4	2	1069	0.306763163	0.743918987
SETD1B	5	1066	0.302988072	0.743918987

SBDS	1	1070	0.417847639	0.743918987
SMAD2	1	1070	0.385922639	0.743918987
SFPQ	1	1070	0.407619244	0.743918987
STAG1	4	1067	0.310558635	0.743918987
SMC1A	1	1070	0.407619244	0.743918987
SDHB	1	1070	0.373078968	0.743918987
SMARCA4	3	1068	0.25734343	0.743918987
SMARCE1	1	1070	0.233915093	0.743918987
TGFBR2	6	1065	0.235141827	0.743918987
TRIM33	3	1068	0.209028188	0.743918987
TSC1	3	1068	0.391950155	0.743918987
USP44	1	1070	0.417847639	0.743918987
WNK2	1	1070	0.391950155	0.743918987
ZNRF3	1	1070	0.391950155	0.743918987
ZRSR2	1	1070	0.395300839	0.743918987
ZNF331	4	1067	0.286675423	0.743918987
ANK1	2	1069	0.385922639	0.743918987
BCLAF1	3	1068	0.204810974	0.743918987
BMP5	2	1069	0.25588785	0.743918987
CIC	5	1066	0.385166104	0.743918987
CTNND1	2	1069	0.206813106	0.743918987
CREBBP	7	1064	0.307718842	0.743918987
CNBD1	3	1068	0.167512175	0.743918987
CYP2C8	1	1070	0.41162043	0.743918987
DCAF12L2	3	1068	0.235461176	0.743918987
EZH2	1	1070	0.395300839	0.743918987
EPHA7	5	1066	0.438901164	0.743918987
ERBB4	13	1058	0.249993023	0.743918987
GATA3	2	1069	0.271205242	0.743918987
JAK1	1	1070	0.419287106	0.743918987
LEF1	1	1070	0.437589284	0.743918987
KDM6A	7	1064	0.337783079	0.743918987
MAP3K13	5	1066	0.173300002	0.743918987
NTRK1	1	1070	0.437589284	0.743918987
NKX2-1	1	1070	0.417847639	0.743918987
NOTCH2	15	1056	0.313805285	0.743918987
PTK6	2	1069	0.230071418	0.743918987
SUZ12	3	1068	0.397966594	0.743918987
TERT	2	1069	0.274757473	0.743918987
TCF3	1	1070	0.41162043	0.743918987
TBL1XR1	2	1069	0.223500184	0.743918987
TAF15	1	1070	0.445739552	0.743991753
TCL1A	1	1070	0.445739552	0.743991753

RPL10	1	1070	0.445739552	0.743991753
GPHN	2	1069	0.453302589	0.751092611
RAD51B	1	1070	0.453302589	0.751092611
FANCA	3	1068	0.455248295	0.751573549
KDM5C	4	1067	0.460826507	0.758026211
GNAS	2	1069	0.463485221	0.759647258
CREB1	1	1070	0.468047515	0.761625705
PTPRC	4	1067	0.467982495	0.761625705
GAS7	1	1070	0.472569172	0.762393575
ZNF521	10	1061	0.473372777	0.762393575
CHD2	7	1064	0.474156966	0.762393575
PTPRB	7	1064	0.475236524	0.762393575
CHEK2	8	1063	0.480141133	0.767549558
ACSL3	3	1068	0.492961889	0.769088308
DDR2	3	1068	0.491225962	0.769088308
LPP	7	1064	0.491618775	0.769088308
TSC2	5	1066	0.492210021	0.769088308
BMPR1A	1	1070	0.489147537	0.769088308
PCBP1	1	1070	0.48639064	0.769088308
ZNF429	8	1063	0.48969111	0.769088308
BIRC3	1	1070	0.494686932	0.769136531
SMO	2	1069	0.503021437	0.779425708
CDK4	1	1070	0.522790815	0.788205092
FCRL4	2	1069	0.524312639	0.788205092
SETBP1	3	1068	0.522027946	0.788205092
BRAF	3	1068	0.519187277	0.788205092
ARID2	5	1066	0.514349498	0.788205092
BAP1	4	1067	0.517356248	0.788205092
KMT2C	17	1054	0.518204936	0.788205092
SETD2	4	1067	0.516391875	0.788205092
TET1	3	1068	0.511194562	0.788205092
LCP1	1	1070	0.532846927	0.791946188
ASXL2	4	1067	0.531059779	0.791946188
DDX10	1	1070	0.532846927	0.791946188
N4BP2	2	1069	0.533778708	0.791946188
HSP90AB1	1	1070	0.542212034	0.801837992
COL2A1	4	1067	0.54543067	0.803978975
SMAD4	1	1070	0.548678618	0.806149167
COL1A1	4	1067	0.550928525	0.80684371
PTPRK	3	1068	0.554542558	0.80952515
NUP214	2	1069	0.562141069	0.813511791
TET2	4	1067	0.561941954	0.813511791
KMT2D	18	1053	0.562649124	0.813511791

WRN	3	1068	0.564775871	0.81399443
FLT3	1	1070	0.572325018	0.81967053
MAP2K1	1	1070	0.572325018	0.81967053
AXIN2	3	1068	0.57684273	0.823542765
CNTRL	3	1068	0.583659971	0.827990511
CEP89	1	1070	0.594507495	0.827990511
MSN	1	1070	0.594507495	0.827990511
PPFIBP1	1	1070	0.594507495	0.827990511
CXCR4	2	1069	0.594507495	0.827990511
SKI	1	1070	0.594548253	0.827990511
CLTC	2	1069	0.594507495	0.827990511
CYLD	3	1068	0.583794574	0.827990511
CLIP1	4	1067	0.608583327	0.830068021
FGFR1OP	1	1070	0.609717786	0.830068021
MAML2	1	1070	0.617980156	0.830068021
MN1	1	1070	0.609717786	0.830068021
PLCG1	1	1070	0.617980156	0.830068021
KDR	4	1067	0.615575177	0.830068021
SDHC	1	1070	0.617980156	0.830068021
SUFU	3	1068	0.600377098	0.830068021
XPC	1	1070	0.612147297	0.830068021
EPAS1	4	1067	0.613403488	0.830068021
FES	1	1070	0.612147297	0.830068021
ZNF479	7	1064	0.59841857	0.830068021
LIFR	3	1068	0.62083305	0.831440131
ETV1	3	1068	0.625027867	0.834596034
NOTCH1	42	1029	0.631347786	0.840562741
FAM131B	1	1070	0.639282373	0.843704062
CCNE1	1	1070	0.639282373	0.843704062
NCOR2	3	1068	0.638391578	0.843704062
BCL11A	2	1069	0.662143907	0.856575806
STIL	3	1068	0.664129259	0.856575806
TAL1	2	1069	0.658492407	0.856575806
APC	6	1065	0.656537969	0.856575806
EBF1	2	1069	0.655862677	0.856575806
LRIG3	3	1068	0.663857502	0.856575806
ZFHX3	16	1055	0.655170583	0.856575806
RGPD3	6	1065	0.65968254	0.856575806
CACNA1D	1	1070	0.666106344	0.856692012
MTOR	6	1065	0.67343155	0.861000139
BARD1	3	1068	0.675145483	0.861000139
RUNX1T1	5	1066	0.673414106	0.861000139
ARHGAP26	3	1068	0.678192298	0.862463035

KTN1	4	1067	0.687606021	0.871183399
RSPO2	3	1068	0.688887313	0.871183399
CBLB	4	1067	0.69348052	0.872133397
DICER1	4	1067	0.693310591	0.872133397
FUBP1	2	1069	0.699754145	0.875588317
PALB2	7	1064	0.700084932	0.875588317
STRN	2	1069	0.708476258	0.881228004
LATS1	2	1069	0.707915225	0.881228004
RET	1	1070	0.717767714	0.888824276
FAM135B	17	1054	0.71849892	0.888824276
ERCC3	1	1070	0.727984809	0.893257036
NF2	3	1068	0.725634762	0.893257036
FLNA	2	1069	0.726243988	0.893257036
AKAP9	9	1062	0.732411807	0.89626674
EGFR	3	1068	0.737071981	0.897098629
PRDM2	7	1064	0.739019575	0.897098629
NBEA	10	1061	0.738276851	0.897098629
FAS	2	1069	0.748441701	0.903703543
BCL11B	2	1069	0.746989094	0.903703543
ATR	5	1066	0.75525468	0.909510941
STAT6	3	1068	0.759808973	0.910433772
PABPC1	4	1067	0.760031717	0.910433772
PICALM	1	1070	0.764573816	0.913464507
PIK3CB	2	1069	0.769184229	0.916560734
NSD1	3	1068	0.772651697	0.916568027
SND1	3	1068	0.773439704	0.916568027
UBR5	5	1066	0.775246966	0.916568027
MLLT1	1	1070	0.782201891	0.922388723
PTPRT	2	1069	0.789549645	0.928641293
POLQ	9	1062	0.79211211	0.929247798
CTCF	2	1069	0.798497405	0.934324284
FNBP1	2	1069	0.805058849	0.936827541
PDE4DIP	16	1055	0.806827243	0.936827541
CLTCL1	2	1069	0.805446425	0.936827541
NCOA2	5	1066	0.814174194	0.940827906
TPM3	3	1068	0.814417108	0.940827906
CRTC3	2	1069	0.829400088	0.941206396
AFF4	4	1067	0.830875824	0.941206396
FLT4	2	1069	0.839673197	0.941206396
SIX2	2	1069	0.834173999	0.941206396
TNC	2	1069	0.822491413	0.941206396
USP8	2	1069	0.818361989	0.941206396
BAZ1A	6	1065	0.838390814	0.941206396

CAMTA1	3	1068	0.82183667	0.941206396
FBXW7	14	1057	0.838554065	0.941206396
RB1	11	1060	0.841695588	0.941206396
SMARCB1	2	1069	0.831939554	0.941206396
DCC	9	1062	0.824598352	0.941206396
PMS1	3	1068	0.839126536	0.941206396
EIF1AX	2	1069	0.848327782	0.946291924
VAV1	2	1069	0.858886426	0.951059604
RPL5	2	1069	0.858755466	0.951059604
PAX5	3	1068	0.856121793	0.951059604
CDKN2A	12	1059	0.868656451	0.959537783
IL7R	2	1069	0.871418321	0.960252228
ZMYM3	3	1068	0.874137328	0.960916095
ERBB2	2	1069	0.878076249	0.962914534
ATIC	2	1069	0.900411337	0.964119686
BCR	2	1069	0.89395553	0.964119686
DDX5	2	1069	0.899967359	0.964119686
NUP98	4	1067	0.896970886	0.964119686
ARHGAP5	4	1067	0.89317282	0.964119686
CDH11	5	1066	0.898223388	0.964119686
PTPN6	1	1070	0.896764362	0.964119686
EPHA3	2	1069	0.898509508	0.964119686
FAT3	23	1048	0.885598008	0.964119686
PRKCB	1	1070	0.896764362	0.964119686
ERBB3	4	1067	0.920275703	0.969385543
TRRAP	9	1062	0.915461706	0.969385543
EP300	21	1050	0.916144907	0.969385543
MYH9	8	1063	0.918736036	0.969385543
IRF4	2	1069	0.918759883	0.969385543
PRPF40B	2	1069	0.92027465	0.969385543
TP63	2	1069	0.91993751	0.969385543
MED12	4	1067	0.924684755	0.971775182
RAC1	2	1069	0.928543613	0.972558136
PTPRD	6	1065	0.931856364	0.972558136
MAP3K1	2	1069	0.931583416	0.972558136
MUC16	64	1007	0.94004854	0.978857884
GMPS	2	1069	0.952406622	0.982685305
KMT2A	5	1066	0.954110995	0.982685305
WWTR1	2	1069	0.954546739	0.982685305
SPOP	2	1069	0.952406622	0.982685305
ELF4	2	1069	0.95167803	0.982685305
A1CF	2	1069	0.961639704	0.985517891
MET	2	1069	0.961524504	0.985517891

MYH11	5	1066	0.976825491	0.989092082
CD79A	2	1069	0.967853133	0.989092082
JAK3	2	1069	0.986913465	0.989092082
PREX2	7	1064	0.983847468	0.989092082
SETDB1	2	1069	0.985375108	0.989092082
DROSHA	4	1067	0.980467801	0.989092082
PTCH1	5	1066	0.979256927	0.989092082
ARHGEF10	2	1069	0.985895961	0.989092082
BCORL1	3	1068	0.977244702	0.989092082
FAM47C	2	1069	0.979153987	0.989092082
PDGFRA	3	1068	0.996259836	0.996259836

Table S6f. Association between CCA of genes undering SBS16* signature and prognosis.

GeneName	CCA>=0.06	CCA<0.06	pvalue	qvalue(fdr)
**CDKN2A**	**5**	**1066**	**1.30E-06**	**5.54E-05**
**SPEN**	**11**	**1060**	**0.001500997**	**0.030476769**
**NFE2L2**	**32**	**1039**	**0.003239965**	**0.047283234**
BCR	2	1069	3.34E-10	2.60E-08
FAM131B	1	1070	3.34E-10	2.60E-08
TFRC	1	1070	3.34E-10	2.60E-08
SRSF3	1	1070	3.34E-10	2.60E-08
RNF43	1	1070	3.34E-10	2.60E-08
FOXO1	1	1070	3.34E-10	2.60E-08
C15orf65	1	1070	2.07E-09	1.07E-07
SEPT6	1	1070	2.07E-09	1.07E-07
ZCCHC8	1	1070	2.07E-09	1.07E-07
GATA3	1	1070	1.13E-07	5.25E-06
EIF1AX	1	1070	4.54E-06	0.000176683
NUP214	2	1069	2.16E-05	0.00076987
NUTM1	2	1069	2.31E-05	0.00076987
FANCD2	2	1069	3.36E-05	0.001044937
STAT3	2	1069	8.13E-05	0.002371947
ZNF384	1	1070	0.000333525	0.008197703
CD79B	1	1070	0.000333525	0.008197703
N4BP2	1	1070	0.000333525	0.008197703
CASP8	4	1067	0.000414854	0.009686848
ACSL6	1	1070	0.000711264	0.015817162
RAP1GDS1	2	1069	0.001321929	0.02806095
NUTM2D	1	1070	0.002636768	0.04104569
NUTM2B	1	1070	0.002636768	0.04104569
CTNNB1	1	1070	0.002636768	0.04104569
CDX2	1	1070	0.002636768	0.04104569
KAT6B	2	1069	0.002439339	0.04104569
STK11	1	1070	0.002636768	0.04104569

MAP3K1	2	1069	0.002240267	0.04104569
MUC4	3	1068	0.0030957	0.046635215
CCDC6	3	1068	0.005385853	0.076217982
CSF3R	1	1070	0.009835352	0.127586376
USP8	1	1070	0.009835352	0.127586376
MYB	1	1070	0.009835352	0.127586376
AFF3	3	1068	0.011055722	0.135868999
TAF15	1	1070	0.010869078	0.135868999
RHOA	2	1069	0.01200519	0.143754459
EP300	26	1045	0.014167891	0.165410126
AKAP9	12	1059	0.015880648	0.180884452
SEPT5	1	1070	0.021319051	0.226272659
VTI1A	1	1070	0.021319051	0.226272659
PIK3CA	17	1054	0.021282861	0.226272659
COL3A1	7	1064	0.022533551	0.23384818
CTNNA2	11	1060	0.025523676	0.259120796
AFF4	6	1065	0.030917731	0.307203842
KAT6A	6	1065	0.03303648	0.321417422
PCM1	3	1068	0.035246484	0.321995896
POU2AF1	1	1070	0.035853933	0.321995896
CCNC	1	1070	0.035853933	0.321995896
DDB2	1	1070	0.035853933	0.321995896
CTCF	1	1070	0.036916996	0.325287497
ERBB2	3	1068	0.039829622	0.344452472
SETD2	7	1064	0.044143676	0.374819941
CDK12	8	1063	0.045484055	0.37930453
CANT1	2	1069	0.047504041	0.389199771
KDM6A	6	1065	0.048969649	0.392412464
RGS7	5	1066	0.049576735	0.392412464
FGFR2	1	1070	0.055627619	0.419001581
KRAS	2	1069	0.055627619	0.419001581
BCOR	1	1070	0.055627619	0.419001581
DDX5	2	1069	0.057675269	0.42084923
RBM10	1	1070	0.05694745	0.42084923
SALL4	2	1069	0.063237388	0.444137481
KEAP1	6	1065	0.062895843	0.444137481
RB1	11	1060	0.063719938	0.444137481
CRTC3	2	1069	0.080430458	0.509385504
BAP1	2	1069	0.074667976	0.509385504
HNF1A	2	1069	0.080716333	0.509385504
EXT2	1	1070	0.079633812	0.509385504
TNFAIP3	1	1070	0.079633812	0.509385504
CIC	3	1068	0.080506356	0.509385504

CTNND1	3	1068	0.075457266	0.509385504
HSP90AA1	1	1070	0.093904564	0.537795768
PWWP2A	1	1070	0.097251898	0.537795768
SRGAP3	4	1067	0.099037336	0.537795768
TCEA1	3	1068	0.094246572	0.537795768
GNA11	1	1070	0.097251898	0.537795768
H3F3B	1	1070	0.093904564	0.537795768
JUN	1	1070	0.097251898	0.537795768
LPP	4	1067	0.094307673	0.537795768
SIX2	1	1070	0.093904564	0.537795768
MSH6	6	1065	0.098413119	0.537795768
PTPRD	10	1061	0.094437136	0.537795768
SIRPA	1	1070	0.097251898	0.537795768
AR	2	1069	0.113879392	0.611283633
MACC1	4	1067	0.120920758	0.641704475
BRD3	1	1070	0.125277811	0.642909208
TRIM33	2	1069	0.125277811	0.642909208
ATP1A1	1	1070	0.125277811	0.642909208
MLLT6	4	1067	0.131245602	0.666214089
PTCH1	6	1065	0.142408588	0.70005064
LATS1	5	1066	0.14146696	0.70005064
RANBP2	7	1064	0.142381063	0.70005064
CARD11	3	1068	0.146896152	0.711163033
PRDM16	3	1068	0.147714806	0.711163033
COL2A1	3	1068	0.153490268	0.728242208
NCOA2	4	1067	0.157646773	0.728242208
DROSHA	3	1068	0.159059326	0.728242208
FBXW7	17	1054	0.15836774	0.728242208
FAM47C	3	1068	0.155180378	0.728242208
AFF1	1	1070	0.170285157	0.736325632
HIST1H3B	4	1067	0.168490964	0.736325632
ZNF521	10	1061	0.168401128	0.736325632
BLM	1	1070	0.170285157	0.736325632
POT1	3	1068	0.163429949	0.736325632
ZNRF3	1	1070	0.170285157	0.736325632
ASXL1	6	1065	0.179712612	0.763439426
CNBD1	3	1068	0.179825132	0.763439426
PALB2	4	1067	0.184355605	0.77562223
SDHC	1	1070	0.187129562	0.780263442
SF3B1	6	1065	0.190533204	0.780517599
TBX3	1	1070	0.1892981	0.780517599
CLIP1	5	1066	0.373250388	0.782678137
EML4	1	1070	0.290250051	0.782678137

EIF4A2	1	1070	0.411623741	0.782678137
FGFR1OP	2	1069	0.365888558	0.782678137
FIP1L1	1	1070	0.419287106	0.782678137
GOPC	1	1070	0.395300839	0.782678137
HERPUD1	1	1070	0.440780193	0.782678137
ITK	1	1070	0.391950155	0.782678137
JAZF1	1	1070	0.385922639	0.782678137
KLK2	1	1070	0.391950155	0.782678137
LIFR	2	1069	0.23945622	0.782678137
LSM14A	1	1070	0.373078968	0.782678137
MYO5A	5	1066	0.392464192	0.782678137
NACA	1	1070	0.41162043	0.782678137
NSD1	1	1070	0.370271862	0.782678137
NCOA1	3	1068	0.391597355	0.782678137
PRCC	1	1070	0.231603482	0.782678137
PAFAH1B2	1	1070	0.337248505	0.782678137
RBM15	3	1068	0.225401296	0.782678137
SEPT9	2	1069	0.37388124	0.782678137
SPECC1	3	1068	0.395300839	0.782678137
TMPRSS2	1	1070	0.378058023	0.782678137
TPM4	1	1070	0.395300839	0.782678137
VAV1	1	1070	0.422021426	0.782678137
ZMYM2	1	1070	0.440780193	0.782678137
NT5C2	2	1069	0.243145205	0.782678137
ARAF	1	1070	0.370271862	0.782678137
BIRC6	16	1055	0.366070688	0.782678137
CACNA1D	2	1069	0.236861429	0.782678137
CTNND2	9	1062	0.41480696	0.782678137
CCR4	1	1070	0.385922639	0.782678137
CD28	1	1070	0.440780193	0.782678137
CD79A	1	1070	0.414260415	0.782678137
CHD4	2	1069	0.207263567	0.782678137
CXCR4	2	1069	0.367921612	0.782678137
CCNE1	1	1070	0.417847639	0.782678137
CDK6	1	1070	0.430792583	0.782678137
DEK	1	1070	0.417847639	0.782678137
EGFR	5	1066	0.240763656	0.782678137
ERBB3	1	1070	0.266309994	0.782678137
ETV5	4	1067	0.196231503	0.782678137
FUBP1	1	1070	0.385922639	0.782678137
FCGR2B	1	1070	0.314749166	0.782678137
FGFR1	1	1070	0.419287106	0.782678137
GLI1	1	1070	0.263918441	0.782678137

GRM3	3	1068	0.433111469	0.782678137
HOXC11	1	1070	0.407619244	0.782678137
HIF1A	2	1069	0.344989579	0.782678137
IKBKB	1	1070	0.41690198	0.782678137
IL7R	2	1069	0.216162919	0.782678137
MAML2	2	1069	0.237638668	0.782678137
MAP2K2	1	1070	0.417847639	0.782678137
MUC16	63	1008	0.333989066	0.782678137
NFATC2	1	1070	0.407619244	0.782678137
PREX2	9	1062	0.394708089	0.782678137
PPM1D	1	1070	0.375455671	0.782678137
PTPN11	1	1070	0.266309994	0.782678137
SRSF2	1	1070	0.411623741	0.782678137
SETDB1	3	1068	0.297849431	0.782678137
SH3GL1	1	1070	0.411623741	0.782678137
TCF7L2	1	1070	0.395300839	0.782678137
AKT3	2	1069	0.301174678	0.782678137
RAF1	1	1070	0.290250051	0.782678137
REL	4	1067	0.256481505	0.782678137
AMER1	4	1067	0.318573801	0.782678137
ARID1B	1	1070	0.391950155	0.782678137
ARID2	2	1069	0.440780193	0.782678137
ATR	11	1060	0.241271967	0.782678137
B2M	1	1070	0.373078968	0.782678137
BARD1	3	1068	0.393933187	0.782678137
CDH1	1	1070	0.407619244	0.782678137
CDH11	5	1066	0.227397263	0.782678137
CNOT3	2	1069	0.399173255	0.782678137
CIITA	1	1070	0.411623741	0.782678137
CLTC	3	1068	0.397192571	0.782678137
CLTCL1	3	1068	0.293827982	0.782678137
CUL3	12	1059	0.227992096	0.782678137
CCNB1IP1	2	1069	0.407619244	0.782678137
CDKN1B	1	1070	0.411623741	0.782678137
CDKN2C	1	1070	0.419287106	0.782678137
DDX10	2	1069	0.264114469	0.782678137
DDX3X	1	1070	0.290250051	0.782678137
MLH1	1	1070	0.411623741	0.782678137
ETV6	2	1069	0.430547421	0.782678137
EIF3E	1	1070	0.411623741	0.782678137
ERCC2	1	1070	0.395300839	0.782678137
ERCC4	4	1067	0.394310647	0.782678137
ERCC5	2	1069	0.270670967	0.782678137

BRCA2	9	1062	0.306418113	0.782678137
FANCE	4	1067	0.438028383	0.782678137
FH	1	1070	0.385922639	0.782678137
IGF2BP2	1	1070	0.206813106	0.782678137
LRP1B	31	1040	0.312489202	0.782678137
LZTR1	1	1070	0.233915093	0.782678137
MED12	2	1069	0.254935397	0.782678137
EXT1	2	1069	0.233197604	0.782678137
MSH2	2	1069	0.257283624	0.782678137
MYH9	7	1064	0.350086659	0.782678137
NF1	7	1064	0.256536494	0.782678137
NAB2	1	1070	0.422021426	0.782678137
NCOR2	3	1068	0.236861429	0.782678137
PHOX2B	1	1070	0.391950155	0.782678137
PRF1	3	1068	0.413612676	0.782678137
PPARG	1	1070	0.395300839	0.782678137
PIK3R1	1	1070	0.373078968	0.782678137
PBRM1	5	1066	0.433427986	0.782678137
PRDM1	5	1066	0.250962255	0.782678137
PTPRK	5	1066	0.350277131	0.782678137
ARHGEF10L	2	1069	0.385922639	0.782678137
SH2B3	1	1070	0.391950155	0.782678137
SBDS	2	1069	0.249122406	0.782678137
SMAD3	1	1070	0.268705878	0.782678137
STAG1	4	1067	0.339578894	0.782678137
SDHB	1	1070	0.430792583	0.782678137
SMARCA4	2	1069	0.402894233	0.782678137
SMARCB1	1	1070	0.233915093	0.782678137
TET2	9	1062	0.286616485	0.782678137
TSC1	4	1067	0.435879314	0.782678137
TRAF7	1	1070	0.206813106	0.782678137
YWHAE	1	1070	0.41162043	0.782678137
USP44	1	1070	0.437589284	0.782678137
WRN	1	1070	0.385922639	0.782678137
WNK2	1	1070	0.395300839	0.782678137
ZFHX3	8	1063	0.242978808	0.782678137
ZMYM3	1	1070	0.337248505	0.782678137
CBL	1	1070	0.407619244	0.782678137
CRNKL1	2	1069	0.370271862	0.782678137
DCAF12L2	1	1070	0.433111469	0.782678137
ECT2L	1	1070	0.427152138	0.782678137
FLNA	3	1068	0.195672301	0.782678137
IRF4	1	1070	0.287800974	0.782678137

ISX	1	1070	0.427152138	0.782678137
JAK1	1	1070	0.419287106	0.782678137
LEF1	1	1070	0.231603482	0.782678137
KMT2D	17	1054	0.403889247	0.782678137
MAP2K4	1	1070	0.395300839	0.782678137
MAP3K13	2	1069	0.289892665	0.782678137
NTRK1	1	1070	0.437589284	0.782678137
NKX2-1	2	1069	0.246253721	0.782678137
NOTCH2	17	1054	0.200057036	0.782678137
PABPC1	10	1061	0.206739177	0.782678137
PCBP1	3	1068	0.345557431	0.782678137
RUNX1T1	9	1062	0.433568504	0.782678137
TET1	2	1069	0.407619244	0.782678137
TBL1XR1	2	1069	0.417847639	0.782678137
TRIM24	2	1069	0.236861429	0.782678137
TP63	3	1068	0.212776816	0.782678137
WT1	1	1070	0.195844402	0.782678137
ZNF429	3	1068	0.419163833	0.782678137
GMPS	3	1068	0.446819055	0.784079824
NUMA1	8	1063	0.471721226	0.784079824
PRRX1	1	1070	0.453302589	0.784079824
CREB1	1	1070	0.468047515	0.784079824
CCR7	1	1070	0.458875868	0.784079824
MTOR	5	1066	0.462667726	0.784079824
MITF	1	1070	0.445739552	0.784079824
ARHGAP5	9	1062	0.455047021	0.784079824
MYCN	1	1070	0.453302589	0.784079824
CHD2	6	1065	0.468174496	0.784079824
EBF1	1	1070	0.468047515	0.784079824
GPC5	3	1068	0.451906943	0.784079824
ID3	1	1070	0.453302589	0.784079824
NF2	2	1069	0.473470043	0.784079824
PML	2	1069	0.472569172	0.784079824
ROBO2	4	1067	0.469234708	0.784079824
APOBEC3B	1	1070	0.456719497	0.784079824
DAXX	1	1070	0.468047515	0.784079824
NOTCH1	43	1028	0.455395631	0.784079824
A1CF	1	1070	0.489725063	0.792786687
CRTC1	1	1070	0.483425555	0.792786687
RSPO3	3	1068	0.498494041	0.792786687
ABL1	1	1070	0.489725063	0.792786687
APC	5	1066	0.499099114	0.792786687
CD274	1	1070	0.494686932	0.792786687

CNTNAP2	9	1062	0.494633168	0.792786687
DICER1	4	1067	0.494043115	0.792786687
ELF3	1	1070	0.483425555	0.792786687
BIRC3	2	1069	0.494686932	0.792786687
BMPR1A	1	1070	0.489147537	0.792786687
PMS1	2	1069	0.496937394	0.792786687
BRCA1	4	1067	0.502430291	0.795372697
ERC1	3	1068	0.507297006	0.797669029
UBR5	8	1063	0.505977014	0.797669029
SETBP1	3	1068	0.512911195	0.800652005
ROS1	5	1066	0.511862987	0.800652005
PTPRC	4	1067	0.514337477	0.800652005
KDM5A	6	1065	0.518049337	0.802615661
EPHA7	6	1065	0.519036252	0.802615661
CDK4	1	1070	0.522790815	0.8057535
PTPRT	4	1067	0.528510216	0.811889048
PTPN13	4	1067	0.530655534	0.812511916
BRAF	3	1068	0.53360272	0.814354478
ACSL3	1	1070	0.537145804	0.814438606
FAM135B	12	1059	0.535835788	0.814438606
PTK6	1	1070	0.548678618	0.829232733
LCP1	1	1070	0.554615282	0.829902922
SS18	3	1068	0.556230438	0.829902922
CYP2C8	1	1070	0.551646762	0.829902922
TERT	3	1068	0.553489657	0.829902922
SUZ12	2	1069	0.562141069	0.836050571
FOXP1	1	1070	0.573777874	0.850648467
PAX7	1	1070	0.577668975	0.853706997
MYH11	3	1068	0.58151469	0.856679369
CYLD	3	1068	0.583794574	0.857333541
PPFIBP1	1	1070	0.594507495	0.859610012
NUP98	2	1069	0.587849196	0.859610012
SKI	1	1070	0.594548253	0.859610012
SPOP	3	1068	0.593204811	0.859610012
CBLC	1	1070	0.594507495	0.859610012
NIN	5	1066	0.612672321	0.866038732
BCL9	5	1066	0.61383045	0.866038732
XPO1	3	1068	0.609717786	0.866038732
FCRL4	1	1070	0.609717786	0.866038732
MET	4	1067	0.612467258	0.866038732
ATM	11	1060	0.609940741	0.866038732
STAG2	1	1070	0.609717786	0.866038732
BCLAF1	10	1061	0.606853311	0.866038732

RET	1	1070	0.619573817	0.868891809
PRDM2	5	1066	0.619378653	0.868891809
RAD21	3	1068	0.621997995	0.869679831
CHST11	1	1070	0.631164581	0.879862267
HSP90AB1	1	1070	0.647651958	0.883406351
KIF5B	1	1070	0.646658489	0.883406351
S100A7	1	1070	0.648840211	0.883406351
TFPT	1	1070	0.647750187	0.883406351
CYSLTR2	1	1070	0.646658489	0.883406351
HNRNPA2B1	2	1069	0.64106609	0.883406351
PLCG1	1	1070	0.647750187	0.883406351
SND1	2	1069	0.648313941	0.883406351
BRD4	1	1070	0.656479784	0.886057975
CREB3L1	1	1070	0.656479784	0.886057975
IKZF1	5	1066	0.65564683	0.886057975
USP6	6	1065	0.662997413	0.890262297
NBEA	9	1062	0.663407451	0.890262297
FANCA	3	1068	0.672132676	0.896817027
BMP5	3	1068	0.670970864	0.896817027
RABEP1	1	1070	0.681240418	0.903804759
KDM5C	2	1069	0.679981148	0.903804759
HLA-A	2	1069	0.688862885	0.908754145
TPM3	2	1069	0.687005857	0.908754145
RAD51B	1	1070	0.694233317	0.91325904
TPR	7	1064	0.697906637	0.915512357
CAMTA1	4	1067	0.702827292	0.916816607
NCOR1	9	1062	0.702004587	0.916816607
CNTRL	3	1068	0.707305268	0.918774497
DGCR8	3	1068	0.708262996	0.918774497
FGFR3	1	1070	0.725272733	0.925559021
MALT1	1	1070	0.725272733	0.925559021
MYC	1	1070	0.729348437	0.925559021
BRIP1	8	1063	0.726977309	0.925559021
BAZ1A	6	1065	0.718406516	0.925559021
DNMT3A	1	1070	0.727984809	0.925559021
RPL5	3	1068	0.726555426	0.925559021
BCL11B	1	1070	0.727984809	0.925559021
FAT1	27	1044	0.734780788	0.929925821
ATRX	5	1066	0.737263751	0.93054641
CDH10	10	1061	0.741099273	0.9328662
GPHN	2	1069	0.749106651	0.934540494
ABL2	3	1068	0.749257654	0.934540494
KMT2C	23	1048	0.750434016	0.934540494

EPHA3	6	1065	0.750251331	0.934540494
TGFBR2	5	1066	0.75264404	0.934799912
ITGAV	3	1068	0.755588474	0.935967686
SLC34A2	2	1069	0.762512405	0.939560141
TP53	225	846	0.762351007	0.939560141
ELF4	3	1068	0.766349748	0.941803507
TRIP11	3	1068	0.77693875	0.942416614
SET	3	1068	0.77616759	0.942416614
TSHR	3	1068	0.773283679	0.942416614
LARP4B	2	1069	0.772082745	0.942416614
PAX5	2	1069	0.771500706	0.942416614
ALK	4	1067	0.780126989	0.943823555
RARA	2	1069	0.782140719	0.943823555
MECOM	4	1067	0.786843389	0.944910056
MLLT10	3	1068	0.795126497	0.944910056
KDR	4	1067	0.793149331	0.944910056
ARID1A	9	1062	0.795076365	0.944910056
ERCC3	3	1068	0.793837942	0.944910056
DCC	8	1063	0.795181267	0.944910056
FBXO11	3	1068	0.804155929	0.952685067
PTEN	5	1066	0.805804286	0.952685067
RNF213	12	1059	0.808680065	0.953670683
FLT4	3	1068	0.821982071	0.960446314
STIL	2	1069	0.824708719	0.960446314
ZEB1	7	1064	0.824621671	0.960446314
FAT4	17	1054	0.8170522	0.960446314
TSC2	2	1069	0.822998228	0.960446314
STRN	3	1068	0.837790078	0.969093191
ETV1	2	1069	0.837159385	0.969093191
GNAS	2	1069	0.838358992	0.969093191
SMAD4	2	1069	0.842656278	0.971655511
CEP89	4	1067	0.84851671	0.97600321
PDE4DIP	10	1061	0.856176852	0.98239457
CREBBP	6	1065	0.860889949	0.985381387
TFG	2	1069	0.865746341	0.986106199
PRKCB	2	1069	0.865699617	0.986106199
MDM4	1	1070	0.869396332	0.987854226
COL1A1	2	1069	0.875740955	0.990607394
FNBP1	2	1069	0.950397075	0.990607394
GAS7	4	1067	0.930689357	0.990607394
KIAA1549	5	1066	0.950987463	0.990607394
KTN1	8	1063	0.895196925	0.990607394
NCKIPSD	3	1068	0.923214431	0.990607394

BCL11A	4	1067	0.894166973	0.990607394
CSF1R	2	1069	0.905170273	0.990607394
JAK2	5	1066	0.954008801	0.990607394
KMT2A	12	1059	0.945458732	0.990607394
RAC1	2	1069	0.928543613	0.990607394
TLX3	2	1069	0.90318993	0.990607394
TNC	3	1068	0.880004589	0.990607394
TRRAP	5	1066	0.912397087	0.990607394
WWTR1	2	1069	0.954546739	0.990607394
ASXL2	2	1069	0.916791148	0.990607394
AXIN2	2	1069	0.93620753	0.990607394
CBLB	2	1069	0.945553901	0.990607394
POLG	2	1069	0.901464238	0.990607394
DNM2	2	1069	0.915625704	0.990607394
EPS15	2	1069	0.879137449	0.990607394
FANCC	2	1069	0.91993751	0.990607394
FLCN	3	1068	0.939128338	0.990607394
LRIG3	2	1069	0.939594285	0.990607394
NBN	2	1069	0.898114361	0.990607394
NDRG1	2	1069	0.947866162	0.990607394
MGMT	2	1069	0.897116032	0.990607394
PMS2	2	1069	0.885309648	0.990607394
POLE	4	1067	0.894578206	0.990607394
ARHGAP26	3	1068	0.945553901	0.990607394
ARHGEF12	2	1069	0.91993751	0.990607394
SETD1B	7	1064	0.926664883	0.990607394
ZBTB16	2	1069	0.928017425	0.990607394
ZNF331	2	1069	0.941168894	0.990607394
ANK1	5	1066	0.921062965	0.990607394
EPAS1	3	1068	0.946422588	0.990607394
ERBB4	8	1063	0.948535875	0.990607394
FAT3	19	1052	0.900094594	0.990607394
RGPD3	4	1067	0.901326049	0.990607394
OMD	2	1069	0.976527959	0.992115817
PIK3CB	3	1068	0.972410169	0.992115817
PLAG1	3	1068	0.961343032	0.992115817
ABI1	2	1069	0.965211102	0.992115817
CHEK2	8	1063	0.961662648	0.992115817
GRIN2A	2	1069	0.974395135	0.992115817
NRG1	2	1069	0.968566718	0.992115817
SUFU	2	1069	0.971921772	0.992115817
BCORL1	2	1069	0.977244702	0.992115817
EZH2	2	1069	0.967245223	0.992115817

RALGDS	2	1069	0.981776591	0.993564571
STAT6	2	1069	0.987181929	0.993564571
NCOA4	2	1069	0.986932031	0.993564571
PTPRB	9	1062	0.985712039	0.993564571
TNFRSF17	2	1069	0.990147493	0.994406192
CSMD3	54	1017	0.994542518	0.996676729
POLQ	9	1062	0.999791353	0.999791353

Table S6g. Association between CCA of genes undering SBS18* signature and prognosis.

**GeneName**	**CCA>=0.06**	**CCA<0.06**	**pvalue**	**qvalue(fdr)**
**PRDM1**	**5**	**1066**	**3.06E-05**	**0.000851193**
**CASP8**	**7**	**1064**	**0.004171976**	**0.063211753**
**SETD2**	**5**	**1066**	**0.004537629**	**0.065844928**
LCK	2	1069	0	0
FLT3	1	1070	1.29E-14	1.29E-12
FANCE	1	1070	1.29E-14	1.29E-12
FANCG	1	1070	1.29E-14	1.29E-12
FHIT	1	1070	1.29E-14	1.29E-12
FAM131B	1	1070	3.34E-10	1.67E-08
STRN	1	1070	3.34E-10	1.67E-08
CIITA	1	1070	3.34E-10	1.67E-08
CBFB	1	1070	3.34E-10	1.67E-08
STAG2	2	1069	3.34E-10	1.67E-08
C15orf65	1	1070	2.07E-09	7.94E-08
SEPT6	1	1070	2.07E-09	7.94E-08
ABL2	1	1070	2.07E-09	7.94E-08
STK11	3	1068	1.00E-08	3.58E-07
FOXO1	1	1070	1.13E-07	3.75E-06
EIF1AX	1	1070	4.54E-06	0.000141876
PTPN11	2	1069	6.20E-06	0.000182219
ACSL6	3	1068	9.15E-05	0.002409101
EZR	1	1070	0.000333525	0.007250552
ZNF384	2	1069	0.000333525	0.007250552
CD79B	1	1070	0.000333525	0.007250552
ARHGEF10L	2	1069	0.000333525	0.007250552
PMS2	1	1070	0.000495919	0.01033164
DEK	1	1070	0.000632926	0.012658526
NUTM2D	1	1070	0.002636768	0.047085145
CDX2	1	1070	0.002636768	0.047085145
MB21D2	1	1070	0.002636768	0.047085145
DGCR8	2	1069	0.003128118	0.053933068
PLAG1	3	1068	0.003626797	0.059869404
BRCA1	4	1067	0.003831642	0.059869404

SPOP	4	1067	0.003732755	0.059869404
ETV5	2	1069	0.004609145	0.065844928
FEN1	2	1069	0.00705645	0.098006246
TRAF7	2	1069	0.007485247	0.101151986
MEN1	2	1069	0.008677632	0.114179372
EWSR1	1	1070	0.009835352	0.117087528
TLX1	1	1070	0.009835352	0.117087528
TFEB	1	1070	0.009835352	0.117087528
ECT2L	2	1069	0.009835352	0.117087528
ZCCHC8	2	1069	0.012294021	0.142953731
BIRC6	10	1061	0.01374754	0.156222042
MSH2	2	1069	0.017632189	0.19591321
RGS7	5	1066	0.01854626	0.201589778
RNF213	9	1062	0.019486208	0.204990878
SEPT9	1	1070	0.021319051	0.204990878
NFATC2	1	1070	0.021319051	0.204990878
WAS	2	1069	0.019853894	0.204990878
BCORL1	3	1068	0.021009921	0.204990878
SUZ12	2	1069	0.020449795	0.204990878
VHL	1	1070	0.022122331	0.208701233
AKAP9	12	1059	0.022606058	0.209315349
PTEN	8	1063	0.023529109	0.213900987
POU2AF1	2	1069	0.035853933	0.309085627
WWTR1	1	1070	0.035853933	0.309085627
DDB2	1	1070	0.035853933	0.309085627
SNX29	4	1067	0.037261943	0.315779177
KTN1	6	1065	0.039804478	0.331703981
NUTM1	2	1069	0.042923977	0.349993613
CCDC6	2	1069	0.043399208	0.349993613
BRIP1	3	1068	0.051163538	0.406059829
GATA1	1	1070	0.055627619	0.434590772
BCL11B	2	1069	0.059121476	0.454780584
PCM1	4	1067	0.072638175	0.532880234
RPN1	1	1070	0.07656776	0.532880234
FGFR2	3	1068	0.071374086	0.532880234
H3F3B	2	1069	0.076734754	0.532880234
SALL4	1	1070	0.07656776	0.532880234
TNFRSF17	1	1070	0.07656776	0.532880234
PBRM1	3	1068	0.076120473	0.532880234
OLIG2	1	1070	0.078091237	0.534871488
AMER1	5	1066	0.082423052	0.542256921
HNF1A	2	1069	0.080716333	0.542256921
PTCH1	11	1060	0.082262176	0.542256921

LEF1	4	1067	0.089383516	0.580412442
PAX7	2	1069	0.090949475	0.583009455
MYH11	5	1066	0.094732256	0.592076601
NRAS	1	1070	0.093904564	0.592076601
CDK12	10	1061	0.098243177	0.606439362
MSH6	5	1066	0.10272743	0.626386767
PIK3CB	5	1066	0.105145686	0.633407749
AXIN1	2	1069	0.107620796	0.640599979
NFE2L2	20	1051	0.109415403	0.643620015
ALK	8	1063	0.114442102	0.647873587
BAP1	6	1065	0.113180147	0.647873587
CDKN2A	20	1051	0.116189781	0.647873587
PTPRC	3	1068	0.11248162	0.647873587
MAP3K13	4	1067	0.116617246	0.647873587
IL6ST	1	1070	0.125277811	0.680857671
ATP1A1	1	1070	0.125277811	0.680857671
NIN	3	1068	0.145192754	0.72596377
FOXA1	4	1067	0.142473232	0.72596377
SETDB1	1	1070	0.140401482	0.72596377
MYC	2	1069	0.142845981	0.72596377
ASXL2	3	1068	0.142198974	0.72596377
DNMT3A	4	1067	0.143935845	0.72596377
KAT6B	5	1066	0.137960874	0.72596377
TSC1	5	1066	0.143712429	0.72596377
ERCC4	4	1067	0.149678949	0.733720338
DCAF12L2	4	1067	0.148609756	0.733720338
MET	4	1067	0.15393619	0.747263057
KIAA1549	5	1066	0.158810092	0.763510056
FANCA	3	1068	0.16119339	0.767587573
ZEB1	6	1065	0.168304709	0.77402344
CLTCL1	2	1069	0.167547314	0.77402344
BRCA2	9	1062	0.170136048	0.77402344
RSPO2	1	1070	0.170285157	0.77402344
JAK1	3	1068	0.168962789	0.77402344
FGFR4	2	1069	0.175571893	0.790864384
ATIC	3	1068	0.25458493	0.800015629
BCL7A	1	1070	0.385922639	0.800015629
BCR	1	1070	0.385922639	0.800015629
CANT1	1	1070	0.317258756	0.800015629
CNTRL	3	1068	0.232022727	0.800015629
COL3A1	17	1054	0.428281023	0.800015629
COL1A1	2	1069	0.248306771	0.800015629
ELN	2	1069	0.285319133	0.800015629

FIP1L1	1	1070	0.419287106	0.800015629
GOPC	1	1070	0.395300839	0.800015629
GOLGA5	2	1069	0.269260647	0.800015629
HOOK3	1	1070	0.395300839	0.800015629
ITK	1	1070	0.391950155	0.800015629
JAZF1	1	1070	0.385922639	0.800015629
KLK2	1	1070	0.391950155	0.800015629
MLLT3	1	1070	0.339789994	0.800015629
MLLT6	7	1064	0.424164516	0.800015629
NFIB	2	1069	0.417847639	0.800015629
PAX8	1	1070	0.407619244	0.800015629
PRCC	2	1069	0.221234376	0.800015629
PDE4DIP	26	1045	0.369526048	0.800015629
PWWP2A	1	1070	0.337248505	0.800015629
SRGAP3	2	1069	0.225478423	0.800015629
SPECC1	3	1068	0.395300839	0.800015629
TPM4	1	1070	0.395300839	0.800015629
ZMYM2	2	1069	0.27156427	0.800015629
AFF4	6	1065	0.254895873	0.800015629
ARAF	2	1069	0.347002397	0.800015629
BCL11A	2	1069	0.231817974	0.800015629
BCL2L12	1	1070	0.411623741	0.800015629
CDH17	1	1070	0.433111469	0.800015629
CACNA1D	2	1069	0.240580143	0.800015629
CARD11	5	1066	0.280107964	0.800015629
CTNNB1	2	1069	0.299766649	0.800015629
CTNND2	15	1056	0.278702112	0.800015629
CD28	1	1070	0.440780193	0.800015629
CSF1R	1	1070	0.314749166	0.800015629
DDR2	2	1069	0.236861429	0.800015629
ELK4	1	1070	0.433111469	0.800015629
EGFR	6	1065	0.303008497	0.800015629
FCRL4	3	1068	0.186793859	0.800015629
FGFR1	1	1070	0.419287106	0.800015629
GATA2	2	1069	0.264806643	0.800015629
GNAS	3	1068	0.190587551	0.800015629
GNAQ	1	1070	0.430792583	0.800015629
H3F3A	2	1069	0.237638668	0.800015629
HNRNPA2B1	1	1070	0.373078968	0.800015629
HOXD13	1	1070	0.385922639	0.800015629
JUN	1	1070	0.440780193	0.800015629
KMT2A	6	1065	0.183592863	0.800015629
MAML2	5	1066	0.407505037	0.800015629

MITF	1	1070	0.445739552	0.800015629
MACC1	4	1067	0.250837416	0.800015629
MAP2K2	1	1070	0.417847639	0.800015629
MSI2	1	1070	0.439748565	0.800015629
NPM1	1	1070	0.417847639	0.800015629
PREX2	10	1061	0.230486108	0.800015629
PDGFRA	4	1067	0.436934903	0.800015629
PRDM16	1	1070	0.391950155	0.800015629
RAC1	1	1070	0.268705878	0.800015629
RSPO3	1	1070	0.407619244	0.800015629
SRSF2	1	1070	0.411623741	0.800015629
SGK1	1	1070	0.411623741	0.800015629
SETBP1	4	1067	0.420461464	0.800015629
STAT3	3	1068	0.343216276	0.800015629
SF3B1	7	1064	0.414162939	0.800015629
SSX1	1	1070	0.430792583	0.800015629
TAF15	2	1069	0.340088027	0.800015629
TAL2	1	1070	0.427152138	0.800015629
TCL1A	2	1069	0.255953846	0.800015629
TLX3	1	1070	0.391950155	0.800015629
TFE3	1	1070	0.373078968	0.800015629
USP8	1	1070	0.407619244	0.800015629
ABL1	3	1068	0.204515773	0.800015629
KIT	2	1069	0.272671285	0.800015629
MAFB	2	1069	0.365758967	0.800015629
MYCL	1	1070	0.411623741	0.800015629
BRAF	2	1069	0.249032412	0.800015629
EP300	25	1046	0.343839488	0.800015629
ABI1	1	1070	0.41162043	0.800015629
ACVR2A	1	1070	0.402894233	0.800015629
ARID1A	8	1063	0.226002037	0.800015629
B2M	1	1070	0.373078968	0.800015629
BAZ1A	6	1065	0.357246861	0.800015629
CDH1	1	1070	0.337248505	0.800015629
CTCF	3	1068	0.345843726	0.800015629
CD274	1	1070	0.395300839	0.800015629
CHD2	8	1063	0.362900792	0.800015629
CSMD3	80	991	0.430699218	0.800015629
CUL3	6	1065	0.19927549	0.800015629
CDKN2C	1	1070	0.419287106	0.800015629
DNM2	1	1070	0.385922639	0.800015629
ELL	1	1070	0.419287106	0.800015629
ERCC5	5	1066	0.316494097	0.800015629

FAT4	17	1054	0.362138309	0.800015629
FBXW7	29	1042	0.436687192	0.800015629
FBXO11	2	1069	0.385922639	0.800015629
FBLN2	1	1070	0.445739552	0.800015629
FH	3	1068	0.227120888	0.800015629
GRIN2A	7	1064	0.342627157	0.800015629
LRIG3	5	1066	0.265931641	0.800015629
KMT2C	38	1033	0.449542472	0.800015629
EXT1	2	1069	0.244293005	0.800015629
MAX	2	1069	0.390995472	0.800015629
MYH9	6	1065	0.367999395	0.800015629
N4BP2	2	1069	0.302675747	0.800015629
NAB2	2	1069	0.254352404	0.800015629
NCOA4	2	1069	0.282577042	0.800015629
NCOR2	5	1066	0.391349936	0.800015629
MGMT	2	1069	0.260576179	0.800015629
PHOX2B	4	1067	0.182193707	0.800015629
PER1	1	1070	0.231603482	0.800015629
PPARG	1	1070	0.395300839	0.800015629
PML	1	1070	0.385922639	0.800015629
POT1	1	1070	0.402894233	0.800015629
PTPRD	10	1061	0.25598312	0.800015629
PTPRT	5	1066	0.307063735	0.800015629
PTPRB	11	1060	0.409303796	0.800015629
RANBP2	4	1067	0.313052018	0.800015629
RPL10	1	1070	0.445739552	0.800015629
RFWD3	1	1070	0.427152138	0.800015629
RBM10	1	1070	0.391950155	0.800015629
SFRP4	7	1064	0.409225507	0.800015629
SH2B3	1	1070	0.391950155	0.800015629
SMAD2	1	1070	0.385922639	0.800015629
SMAD4	1	1070	0.43794341	0.800015629
SPEN	11	1060	0.199775765	0.800015629
SMC1A	1	1070	0.407619244	0.800015629
SUFU	4	1067	0.38480206	0.800015629
SMARCA4	2	1069	0.402894233	0.800015629
SMARCB1	1	1070	0.233915093	0.800015629
SMARCE1	1	1070	0.233915093	0.800015629
TMEM127	1	1070	0.373078968	0.800015629
TPM3	2	1069	0.261330953	0.800015629
TSC2	2	1069	0.27156427	0.800015629
TNFRSF14	1	1070	0.395300839	0.800015629
YWHAE	1	1070	0.41162043	0.800015629

USP44	1	1070	0.437589284	0.800015629
WRN	2	1069	0.230887678	0.800015629
WNK2	1	1070	0.391950155	0.800015629
ZRSR2	2	1069	0.324202734	0.800015629
ZBTB16	1	1070	0.411623741	0.800015629
ZFHX3	18	1053	0.449608784	0.800015629
ZMYM3	2	1069	0.396184077	0.800015629
ZNF331	4	1067	0.191856146	0.800015629
ANK1	5	1066	0.392517621	0.800015629
ARNT	1	1070	0.422021426	0.800015629
CIC	5	1066	0.385166104	0.800015629
CD209	1	1070	0.407619244	0.800015629
CREBBP	9	1062	0.371653915	0.800015629
DAXX	2	1069	0.257910577	0.800015629
EZH2	1	1070	0.395300839	0.800015629
ERBB4	14	1057	0.41654016	0.800015629
FAM135B	23	1048	0.44851236	0.800015629
FAT3	29	1042	0.213716696	0.800015629
ISX	1	1070	0.427152138	0.800015629
KLF4	2	1069	0.194722041	0.800015629
KDM6A	14	1057	0.179748947	0.800015629
MAP2K4	1	1070	0.395300839	0.800015629
MAP3K1	1	1070	0.41162043	0.800015629
NKX2-1	2	1069	0.246253721	0.800015629
NOTCH1	83	988	0.205660456	0.800015629
NOTCH2	18	1053	0.283270595	0.800015629
PMS1	2	1069	0.274183796	0.800015629
PABPC1	7	1064	0.285757962	0.800015629
PRPF40B	1	1070	0.430792583	0.800015629
PTK6	2	1069	0.230071418	0.800015629
RHOA	1	1070	0.20904711	0.800015629
RUNX1T1	9	1062	0.409604674	0.800015629
TCF3	1	1070	0.41162043	0.800015629
EML4	3	1068	0.453515039	0.801263319
KDM5A	1	1070	0.453302589	0.801263319
CREB1	1	1070	0.468047515	0.804205352
CXCR4	2	1069	0.468047515	0.804205352
MUC4	1	1070	0.463485221	0.804205352
NTRK3	4	1067	0.465199958	0.804205352
NR4A3	2	1069	0.458561783	0.804205352
TSHR	1	1070	0.468047515	0.804205352
ASXL1	4	1067	0.46755347	0.804205352
ROBO2	4	1067	0.466687124	0.804205352

MTOR	6	1065	0.470555297	0.805745371
NUMA1	7	1064	0.476066746	0.805961423
NUP214	1	1070	0.473470043	0.805961423
MN1	2	1069	0.475168677	0.805961423
TNC	3	1068	0.478757905	0.805961423
CNOT3	2	1069	0.480353008	0.805961423
NCOR1	9	1062	0.480112268	0.805961423
EPAS1	4	1067	0.48496317	0.810975201
POLQ	9	1062	0.488784567	0.814640945
GPC5	4	1067	0.495889691	0.821009423
BIRC3	1	1070	0.494686932	0.821009423
PLCG1	2	1069	0.498569629	0.822722159
LIFR	5	1066	0.503896654	0.828777392
DCC	16	1055	0.50908466	0.834565017
HERPUD1	1	1070	0.512482662	0.837389971
LATS1	4	1067	0.516151774	0.840638068
NT5C2	1	1070	0.521696503	0.843210991
CDK4	1	1070	0.522790815	0.843210991
MLH1	3	1068	0.520626418	0.843210991
CLIP1	5	1066	0.530418212	0.845788773
LCP1	1	1070	0.532846927	0.845788773
IDH1	1	1070	0.531351014	0.845788773
KDR	7	1064	0.529891516	0.845788773
STAG1	3	1068	0.529759656	0.845788773
CEP89	3	1068	0.537155058	0.84798323
DDX10	4	1067	0.540570464	0.84798323
POLG	3	1068	0.5410077	0.84798323
PRKCB	2	1069	0.541013301	0.84798323
AFF3	7	1064	0.544078531	0.850122705
KEAP1	6	1065	0.546412828	0.851110324
VAV1	1	1070	0.548678618	0.851985431
BMP5	2	1069	0.559624065	0.866291122
FOXP1	1	1070	0.573777874	0.874661393
ARID1B	5	1066	0.567752004	0.874661393
CDH11	5	1066	0.573576642	0.874661393
DICER1	3	1068	0.571970908	0.874661393
STAT5B	1	1070	0.572325018	0.874661393
AXIN2	3	1068	0.57684273	0.876660683
HSP90AB1	1	1070	0.598005533	0.876840958
MSN	1	1070	0.594507495	0.876840958
NONO	1	1070	0.587849196	0.876840958
PPFIBP1	1	1070	0.594507495	0.876840958
TCF12	1	1070	0.598005533	0.876840958

FUBP1	1	1070	0.587849196	0.876840958
SKI	1	1070	0.594548253	0.876840958
KRAS	5	1066	0.59701908	0.876840958
CLTC	2	1069	0.594507495	0.876840958
NRG1	3	1068	0.59456898	0.876840958
CBLC	2	1069	0.594507495	0.876840958
TBX3	4	1067	0.58558286	0.876840958
LARP4B	2	1069	0.602316251	0.880579315
HIF1A	1	1070	0.609717786	0.888801437
LRP1B	47	1024	0.619761101	0.895608527
SDHC	1	1070	0.617980156	0.895608527
NTRK1	2	1069	0.617884855	0.895608527
CHST11	1	1070	0.631164581	0.896540598
JAK3	3	1068	0.625363445	0.896540598
PIM1	1	1070	0.631164581	0.896540598
FANCD2	6	1065	0.629980398	0.896540598
FLCN	1	1070	0.631164581	0.896540598
FAM47C	3	1068	0.629717807	0.896540598
NCOA1	3	1068	0.635903763	0.900713545
FNBP1	3	1068	0.649815541	0.901046372
S100A7	1	1070	0.648840211	0.901046372
TFPT	1	1070	0.647750187	0.901046372
AR	3	1068	0.643399463	0.901046372
CYSLTR2	1	1070	0.646658489	0.901046372
MALT1	2	1069	0.638006349	0.901046372
APC	5	1066	0.643591432	0.901046372
TRIM33	4	1067	0.65055548	0.901046372
MYO5A	4	1067	0.652989867	0.901919706
CREB3L1	1	1070	0.656479784	0.904242127
ZNF429	6	1065	0.658870022	0.905041239
BCLAF1	9	1062	0.662340836	0.907316214
CLP1	4	1067	0.676813674	0.909831209
DNAJB1	3	1068	0.683568335	0.909831209
RABEP1	1	1070	0.681240418	0.909831209
PIK3CA	7	1064	0.683796973	0.909831209
AKT3	3	1068	0.672497628	0.909831209
ATR	10	1061	0.671253481	0.909831209
BLM	2	1069	0.671441116	0.909831209
CHEK2	11	1060	0.678557023	0.909831209
TGFBR2	5	1066	0.685280134	0.909831209
FOXO4	1	1070	0.685648459	0.909831209
GATA3	2	1069	0.686012732	0.909831209
RGPD3	6	1065	0.670960411	0.909831209

HLA-A	2	1069	0.688862885	0.911194293
KIF5B	4	1067	0.703774494	0.912433026
TRIP11	3	1068	0.703262018	0.912433026
GRM3	8	1063	0.700146334	0.912433026
MLLT10	5	1066	0.704398296	0.912433026
ARHGAP5	4	1067	0.697937119	0.912433026
TRRAP	6	1065	0.701454559	0.912433026
CBLB	4	1067	0.69348052	0.912433026
TP53	345	726	0.692219821	0.912433026
GMPS	3	1068	0.708775353	0.914089139
EPHA7	5	1066	0.709333172	0.914089139
FGFR3	1	1070	0.725272733	0.917179417
RET	1	1070	0.717767714	0.917179417
ARID2	8	1063	0.728240457	0.917179417
ERCC3	1	1070	0.727984809	0.917179417
NF2	4	1067	0.725634762	0.917179417
PALB2	11	1060	0.720037115	0.917179417
RB1	10	1061	0.716824595	0.917179417
TET2	8	1063	0.72584689	0.917179417
ELF4	3	1068	0.724131593	0.917179417
LZTR1	3	1068	0.730139277	0.917260398
THRAP3	2	1069	0.739050282	0.926128173
GPHN	3	1068	0.749106651	0.927112192
XPO1	3	1068	0.748464392	0.927112192
FAS	2	1069	0.748441701	0.927112192
NBEA	15	1056	0.748906687	0.927112192
TERT	3	1068	0.746978948	0.927112192
PICALM	1	1070	0.764573816	0.927385736
RBM15	2	1069	0.770961028	0.927385736
ERBB3	4	1067	0.768858412	0.927385736
MUC16	107	964	0.770926207	0.927385736
NUP98	3	1068	0.773271437	0.927385736
STAT6	3	1068	0.759808973	0.927385736
SND1	3	1068	0.773439704	0.927385736
ERBB2	3	1068	0.760973211	0.927385736
PRDM2	8	1063	0.756320463	0.927385736
ARHGAP26	1	1070	0.764573816	0.927385736
SLC34A2	2	1069	0.762512405	0.927385736
PAX5	2	1069	0.771500706	0.927385736
ZNF479	6	1065	0.762375243	0.927385736
TMPRSS2	3	1068	0.77649618	0.928823182
MLLT1	1	1070	0.782201891	0.928980868
NSD1	4	1067	0.780957107	0.928980868

MECOM	2	1069	0.781897676	0.928980868
ACSL3	5	1066	0.788106076	0.929371869
ATRX	6	1065	0.791824833	0.929371869
KDM5C	5	1066	0.789703829	0.929371869
SETD1B	8	1063	0.785685637	0.929371869
ESR1	2	1069	0.790870493	0.929371869
RAD21	4	1067	0.794208966	0.92998708
RNF43	3	1068	0.800017337	0.934599693
USP6	10	1061	0.803413082	0.936378884
POLE	3	1068	0.811815399	0.943971394
IL7R	2	1069	0.815228737	0.945740995
PPP2R1A	7	1064	0.821258276	0.950530412
TPR	5	1066	0.824915543	0.952558364
CRTC3	2	1069	0.829400088	0.953333434
FCGR2B	1	1070	0.82902498	0.953333434
CSF3R	2	1069	0.831947541	0.954068281
SEPT5	2	1069	0.840631832	0.954365178
ETV1	4	1067	0.845896806	0.954365178
SIX2	2	1069	0.834173999	0.954365178
ERG	2	1069	0.849385008	0.954365178
DROSHA	3	1068	0.843939954	0.954365178
FAT1	39	1032	0.841573434	0.954365178
CTNND1	3	1068	0.847250018	0.954365178
EPHA3	8	1063	0.847937532	0.954365178
KMT2D	37	1034	0.845576476	0.954365178
XPC	2	1069	0.852351719	0.955551255
TRIM24	4	1067	0.856166966	0.957681171
FLT4	4	1067	0.859206679	0.958936025
ATM	7	1064	0.864516268	0.962712993
ETV6	1	1070	0.869396332	0.965995924
TP63	4	1067	0.875251575	0.970345427
EPS15	2	1069	0.879137449	0.972497178
EIF4A2	2	1069	0.89808997	0.973990648
CHD4	4	1067	0.883286591	0.973990648
DDX5	2	1069	0.899967359	0.973990648
LPP	7	1064	0.898906291	0.973990648
TAL1	1	1070	0.896764362	0.973990648
REL	2	1069	0.89669414	0.973990648
NF1	9	1062	0.896234843	0.973990648
NBN	2	1069	0.898114361	0.973990648
PTPN6	1	1070	0.896764362	0.973990648
PTPRK	6	1065	0.886211106	0.973990648
KAT6A	5	1066	0.904447569	0.976717424

UBR5	8	1063	0.908234895	0.976717424
CAMTA1	9	1062	0.910300639	0.976717424
PTPN13	4	1067	0.910248946	0.976717424
PDGFRB	2	1069	0.912976114	0.977490486
FANCC	2	1069	0.91993751	0.978656925
IRF4	2	1069	0.918759883	0.978656925
TBL1XR1	4	1067	0.91832655	0.978656925
NCKIPSD	3	1068	0.923214431	0.980057783
GAS7	6	1065	0.938464549	0.992034407
SS18	2	1069	0.937268744	0.992034407
A1CF	3	1068	0.948274158	0.993596462
BCL9	8	1063	0.948279936	0.993596462
EBF1	6	1065	0.949878217	0.993596462
IKZF1	5	1066	0.942743817	0.993596462
NDRG1	2	1069	0.947866162	0.993596462
NCOA2	7	1064	0.952716439	0.9944848
BRD4	4	1067	0.970493454	0.997641787
CTNNA2	8	1063	0.9717031	0.997641787
CD79A	2	1069	0.967853133	0.997641787
STIL	5	1066	0.95999333	0.997641787
ZNF521	10	1061	0.971165137	0.997641787
CYLD	7	1064	0.95838477	0.997641787
IGF2BP2	3	1068	0.971103054	0.997641787
CNBD1	5	1066	0.970697996	0.997641787
COL2A1	5	1066	0.987821646	0.999775072
HSP90AA1	2	1069	0.988260753	0.999775072
BRD3	2	1069	0.990905111	0.999775072
JAK2	4	1067	0.993513772	0.999775072
BARD1	2	1069	0.983616736	0.999775072
CDH10	18	1053	0.982977629	0.999775072
CNTNAP2	13	1058	0.999775072	0.999775072
ELF3	2	1069	0.993804114	0.999775072
MED12	6	1065	0.997967912	0.999775072
PRF1	2	1069	0.992315055	0.999775072
ARHGEF10	2	1069	0.985895961	0.999775072
ZNRF3	2	1069	0.996485759	0.999775072
TET1	5	1066	0.988235832	0.999775072

Table S6h. Association between CCA of genes undering SBS33* signature and prognosis.

GeneName	CCA>=0.06	CCA<0.06	pvalue	qvalue(fdr)
**PRDM1**	**5**	**1066**	**6.60E-06**	**0.000196573**
**RNF213**	**6**	**1065**	**0.001223397**	**0.026975898**
**ALK**	**5**	**1066**	**0.002051862**	**0.041130511**

**BIRC6**	**5**	**1066**	**0.00650643**	**0.09564452**
LCK	2	1069	0	0
FLT3	1	1070	1.29E-14	1.14E-12
PRKACA	1	1070	1.29E-14	1.14E-12
FANCG	1	1070	1.29E-14	1.14E-12
FHIT	1	1070	1.29E-14	1.14E-12
NUTM2B	2	1069	7.84E-09	5.76E-07
SH3GL1	1	1070	1.13E-07	6.20E-06
FOXO1	1	1070	1.13E-07	6.20E-06
ACSL6	2	1069	8.30E-07	4.07E-05
TFEB	2	1069	1.79E-06	7.90E-05
CLP1	1	1070	3.27E-06	0.000111036
FGFR1	1	1070	3.27E-06	0.000111036
ATP2B3	1	1070	3.27E-06	0.000111036
STK11	2	1069	6.69E-06	0.000196573
EPS15	3	1068	0.000185032	0.005099937
IL7R	1	1070	0.000333525	0.008652041
PRPF40B	1	1070	0.000382315	0.009366707
CARD11	1	1070	0.000632926	0.014690552
MSH2	2	1069	0.001875035	0.039375735
NUTM2D	1	1070	0.002636768	0.043067213
BARD1	1	1070	0.002636768	0.043067213
CDH1	1	1070	0.002636768	0.043067213
MEN1	1	1070	0.002636768	0.043067213
PPARG	1	1070	0.002636768	0.043067213
FGFR3	3	1068	0.005724765	0.090165047
CASP8	4	1067	0.006346315	0.09564452
COL1A1	4	1067	0.006881134	0.097889678
PCM1	1	1070	0.009835352	0.123925439
RALGDS	2	1069	0.009513856	0.123925439
OLIG2	2	1069	0.009413082	0.123925439
TET1	2	1069	0.009835352	0.123925439
MAX	1	1070	0.021319051	0.247413201
TPM3	1	1070	0.021319051	0.247413201
SUZ12	2	1069	0.020449795	0.247413201
PTPRC	5	1066	0.023422972	0.264859758
ZNF331	3	1068	0.028783665	0.317339907
MALT1	2	1069	0.031162446	0.333177804
CNTNAP2	10	1061	0.031731219	0.333177804
MUC4	1	1070	0.035853933	0.351368541
CCNC	1	1070	0.035853933	0.351368541
ARHGEF12	1	1070	0.035853933	0.351368541
ERBB4	9	1062	0.03729396	0.357535574

IGF2BP2	3	1068	0.047410439	0.44485114
HSP90AA1	2	1069	0.055717761	0.501459852
FGFR2	1	1070	0.055627619	0.501459852
PDGFRA	3	1068	0.059058607	0.52089691
MNX1	1	1070	0.07656776	0.602971111
SS18	2	1069	0.074111832	0.602971111
GNA11	1	1070	0.07656776	0.602971111
SALL4	1	1070	0.07656776	0.602971111
HNF1A	1	1070	0.07656776	0.602971111
KLF4	1	1070	0.07656776	0.602971111
ARAF	2	1069	0.080506356	0.612125913
TNFAIP3	1	1070	0.079633812	0.612125913
BCR	4	1067	0.0931402	0.627453226
H3F3A	4	1067	0.092482994	0.627453226
NRAS	1	1070	0.093904564	0.627453226
SRC	1	1070	0.093904564	0.627453226
CBFA2T3	1	1070	0.093904564	0.627453226
NBN	1	1070	0.093904564	0.627453226
PABPC1	7	1064	0.08990345	0.627453226
PTK6	2	1069	0.090981005	0.627453226
AFF1	1	1070	0.097251898	0.639864181
TNC	2	1069	0.100114804	0.639864181
PRDM2	4	1067	0.098887419	0.639864181
BRCA2	5	1066	0.101719034	0.640829915
CEP89	2	1069	0.108005996	0.661536725
TAL1	3	1068	0.107436083	0.661536725
PTPRT	4	1067	0.111508668	0.673634556
MACC1	2	1069	0.115285858	0.687041399
FGFR1OP	1	1070	0.140401482	0.711730105
IL2	1	1070	0.140401482	0.711730105
AR	4	1067	0.141704249	0.711730105
IL6ST	1	1070	0.125277811	0.711730105
SETDB1	1	1070	0.140401482	0.711730105
MYC	2	1069	0.142845981	0.711730105
APC	3	1068	0.140401482	0.711730105
CDH11	6	1065	0.132539479	0.711730105
CDKN2A	16	1055	0.125374719	0.711730105
FAT4	16	1055	0.140996449	0.711730105
LRIG3	3	1068	0.138134319	0.711730105
PALB2	3	1068	0.143637141	0.711730105
SLC34A2	3	1068	0.129712885	0.711730105
EZH2	2	1069	0.125277811	0.711730105
FAM135B	17	1054	0.12864089	0.711730105

POLQ	4	1067	0.145459435	0.712751231
STIL	3	1068	0.152988921	0.731224913
PTCH1	6	1065	0.154203893	0.731224913
RGS7	6	1065	0.153346378	0.731224913
ATIC	1	1070	0.337248505	0.751527976
AKAP9	8	1063	0.339261766	0.751527976
ACSL3	3	1068	0.177649449	0.751527976
ASPSCR1	1	1070	0.370271862	0.751527976
COL3A1	7	1064	0.23298788	0.751527976
COL2A1	2	1069	0.280582544	0.751527976
ELN	2	1069	0.285319133	0.751527976
ERC1	1	1070	0.433111469	0.751527976
EIF4A2	1	1070	0.41162043	0.751527976
FAM131B	2	1069	0.234504988	0.751527976
GPHN	1	1070	0.453302589	0.751527976
GAS7	1	1070	0.419287106	0.751527976
GMPS	1	1070	0.395300839	0.751527976
HERPUD1	1	1070	0.440780193	0.751527976
JAZF1	1	1070	0.385922639	0.751527976
LIFR	1	1070	0.411623741	0.751527976
LSM14A	2	1069	0.264227822	0.751527976
HLA-A	1	1070	0.417847639	0.751527976
MLLT3	1	1070	0.339789994	0.751527976
MYO5A	2	1069	0.395300839	0.751527976
NUP214	2	1069	0.265807737	0.751527976
PAX8	2	1069	0.255992587	0.751527976
SEPT5	1	1070	0.439748565	0.751527976
SLC45A3	2	1069	0.251274364	0.751527976
SPECC1	1	1070	0.395300839	0.751527976
TRIP11	3	1068	0.218187534	0.751527976
ZMYM2	1	1070	0.453302589	0.751527976
NT5C2	3	1068	0.238161697	0.751527976
ACVR1	1	1070	0.411623741	0.751527976
A1CF	4	1067	0.380081415	0.751527976
ACKR3	1	1070	0.395300839	0.751527976
BRD4	1	1070	0.391950155	0.751527976
ABL2	2	1069	0.248332745	0.751527976
CCR4	1	1070	0.385922639	0.751527976
CD79A	1	1070	0.391950155	0.751527976
CSF1R	1	1070	0.314749166	0.751527976
DDX5	1	1070	0.295163638	0.751527976
DEK	1	1070	0.395300839	0.751527976
DDR2	4	1067	0.428413172	0.751527976

EGFR	4	1067	0.39604625	0.751527976
FLT4	1	1070	0.437589284	0.751527976
FOXA1	2	1069	0.288516467	0.751527976
FLI1	1	1070	0.385922639	0.751527976
GRM3	5	1066	0.30032561	0.751527976
GNAS	3	1068	0.208493972	0.751527976
H3F3B	1	1070	0.337248505	0.751527976
HNRNPA2B1	2	1069	0.235074203	0.751527976
HOXA13	1	1070	0.407619244	0.751527976
HOXC13	1	1070	0.411623741	0.751527976
HOXD13	1	1070	0.385922639	0.751527976
JAK2	2	1069	0.29334266	0.751527976
JUN	1	1070	0.440780193	0.751527976
MAML2	2	1069	0.36761093	0.751527976
MDM2	1	1070	0.402894233	0.751527976
MECOM	3	1068	0.428461534	0.751527976
MTOR	6	1065	0.31821553	0.751527976
NCOA2	6	1065	0.233723336	0.751527976
PAX3	2	1069	0.231603482	0.751527976
PLAG1	1	1070	0.385922639	0.751527976
POU2AF1	2	1069	0.427152138	0.751527976
PRDM16	4	1067	0.368383221	0.751527976
RARA	1	1070	0.402894233	0.751527976
ARHGAP5	3	1068	0.274782492	0.751527976
STAT6	2	1069	0.268468337	0.751527976
SIX2	1	1070	0.438674524	0.751527976
SMO	2	1069	0.329281044	0.751527976
SF3B1	3	1068	0.29394171	0.751527976
TAF15	1	1070	0.445739552	0.751527976
TAL2	1	1070	0.427152138	0.751527976
TCL1A	1	1070	0.445739552	0.751527976
TEC	1	1070	0.20904711	0.751527976
TRRAP	7	1064	0.229146436	0.751527976
USP8	1	1070	0.407619244	0.751527976
AKT3	2	1069	0.234981788	0.751527976
ERBB2	2	1069	0.344409135	0.751527976
ERG	1	1070	0.419287106	0.751527976
KIT	2	1069	0.307187883	0.751527976
MAF	1	1070	0.413219358	0.751527976
MYB	1	1070	0.413219358	0.751527976
WAS	1	1070	0.385922639	0.751527976
ZNF521	5	1066	0.21280408	0.751527976
EP300	12	1059	0.305055521	0.751527976

ACVR2A	1	1070	0.413219358	0.751527976
AMER1	2	1069	0.318573801	0.751527976
ARID1B	4	1067	0.420539358	0.751527976
ATM	5	1066	0.293217273	0.751527976
AXIN1	1	1070	0.427152138	0.751527976
BAX	1	1070	0.385922639	0.751527976
BLM	1	1070	0.411623741	0.751527976
BUB1B	2	1069	0.407619244	0.751527976
CD274	1	1070	0.395300839	0.751527976
CDC73	1	1070	0.231603482	0.751527976
CHD2	2	1069	0.265532186	0.751527976
CCDC6	1	1070	0.419287106	0.751527976
CSMD3	38	1033	0.233020086	0.751527976
CCNB1IP1	1	1070	0.445739552	0.751527976
CARS	1	1070	0.419287106	0.751527976
DNMT3A	2	1069	0.419287106	0.751527976
EBF1	4	1067	0.245532398	0.751527976
ELL	1	1070	0.419287106	0.751527976
ETV6	1	1070	0.440780193	0.751527976
ERCC2	3	1068	0.198082068	0.751527976
FAT1	20	1051	0.440297338	0.751527976
FBXW7	16	1055	0.407932529	0.751527976
FBXO11	2	1069	0.241037462	0.751527976
FBLN2	1	1070	0.445739552	0.751527976
FH	1	1070	0.453302589	0.751527976
FUS	1	1070	0.213533295	0.751527976
GRIN2A	6	1065	0.282724834	0.751527976
KEAP1	8	1063	0.265946332	0.751527976
LRP1B	19	1052	0.423392786	0.751527976
KDM5C	2	1069	0.254756196	0.751527976
MSH6	4	1067	0.166704621	0.751527976
N4BP2	3	1068	0.302675747	0.751527976
NF1	6	1065	0.358888107	0.751527976
MGMT	3	1068	0.163376188	0.751527976
PER1	2	1069	0.27156427	0.751527976
PTEN	6	1065	0.380858487	0.751527976
POLE	2	1069	0.236861429	0.751527976
PML	2	1069	0.259903199	0.751527976
PTPRK	3	1068	0.171162604	0.751527976
RAD51B	1	1070	0.453302589	0.751527976
RANBP2	3	1068	0.24562879	0.751527976
ARHGAP26	2	1069	0.379745605	0.751527976
ARHGEF10	1	1070	0.41162043	0.751527976

ARHGEF10L	2	1069	0.222522916	0.751527976
RPL10	1	1070	0.445739552	0.751527976
RFWD3	1	1070	0.427152138	0.751527976
RNF43	1	1070	0.419287106	0.751527976
RBM10	3	1068	0.162938243	0.751527976
RSPO2	3	1068	0.168706247	0.751527976
SFRP4	6	1065	0.237730654	0.751527976
SETD1B	5	1066	0.219018388	0.751527976
SETD2	4	1067	0.405374783	0.751527976
SPEN	11	1060	0.170821142	0.751527976
SUFU	2	1069	0.255627265	0.751527976
SMARCA4	4	1067	0.259943991	0.751527976
SMARCD1	1	1070	0.419287106	0.751527976
TRIM33	4	1067	0.307387018	0.751527976
TNFRSF14	1	1070	0.395300839	0.751527976
USP44	1	1070	0.417847639	0.751527976
WRN	2	1069	0.254785398	0.751527976
WNK2	1	1070	0.419287106	0.751527976
WIF1	1	1070	0.314749166	0.751527976
ZRSR2	1	1070	0.395300839	0.751527976
ZFHX3	9	1062	0.223650985	0.751527976
ATP1A1	1	1070	0.395300839	0.751527976
BIRC3	1	1070	0.440780193	0.751527976
BCORL1	1	1070	0.373078968	0.751527976
BMP5	2	1069	0.293936789	0.751527976
CBLC	1	1070	0.395300839	0.751527976
CD209	1	1070	0.407619244	0.751527976
CYP2C8	1	1070	0.41162043	0.751527976
DCAF12L2	6	1065	0.381349789	0.751527976
EPHA3	4	1067	0.173905519	0.751527976
EPHA7	2	1069	0.223500184	0.751527976
ECT2L	1	1070	0.43794341	0.751527976
ESR1	1	1070	0.433111469	0.751527976
FAM47C	2	1069	0.294488545	0.751527976
FAT3	21	1050	0.440992573	0.751527976
GATA3	3	1068	0.228029158	0.751527976
JAK1	2	1069	0.419287106	0.751527976
LEF1	1	1070	0.437589284	0.751527976
MB21D2	1	1070	0.453302589	0.751527976
MAP3K13	3	1068	0.244176796	0.751527976
NTRK1	3	1068	0.177301004	0.751527976
PMS1	1	1070	0.433111469	0.751527976
RHOA	1	1070	0.20904711	0.751527976

TBX3	2	1069	0.374848729	0.751527976
TP63	3	1068	0.434008688	0.751527976
WT1	1	1070	0.370271862	0.751527976
ZNF429	11	1060	0.253380779	0.751527976
ZNF479	2	1069	0.284469426	0.751527976
CRTC3	1	1070	0.456719497	0.754356922
MLLT6	4	1067	0.476721649	0.757020479
AFF3	4	1067	0.485853015	0.757020479
CDH17	1	1070	0.469858578	0.757020479
FGFR4	2	1069	0.47396853	0.757020479
NTRK3	4	1067	0.47793739	0.757020479
ABL1	1	1070	0.473470043	0.757020479
KRAS	2	1069	0.473470043	0.757020479
CAMTA1	3	1068	0.485420139	0.757020479
CBLB	1	1070	0.463485221	0.757020479
CLTCL1	3	1068	0.490947522	0.757020479
ELF3	1	1070	0.48639064	0.757020479
FANCE	1	1070	0.462165043	0.757020479
IKZF1	3	1068	0.482258388	0.757020479
RHOH	1	1070	0.489725063	0.757020479
STAG1	1	1070	0.468047515	0.757020479
SMARCB1	1	1070	0.483425555	0.757020479
ANK1	4	1067	0.475701058	0.757020479
BCLAF1	5	1066	0.488866059	0.757020479
PCBP1	1	1070	0.48639064	0.757020479
HSP90AB1	1	1070	0.542212034	0.772822496
KTN1	4	1067	0.529366584	0.772822496
LCP1	1	1070	0.532846927	0.772822496
MYH11	3	1068	0.506209743	0.772822496
PAX7	2	1069	0.543096937	0.772822496
RPN1	1	1070	0.508651841	0.772822496
TPR	5	1066	0.524231927	0.772822496
CCND2	1	1070	0.544087487	0.772822496
ETV1	4	1067	0.510373793	0.772822496
FCRL4	2	1069	0.524312639	0.772822496
IDH1	1	1070	0.531351014	0.772822496
KAT6A	3	1068	0.521351795	0.772822496
LPP	6	1065	0.524617403	0.772822496
KMT2A	3	1068	0.531346785	0.772822496
MITF	2	1069	0.544087487	0.772822496
MN1	4	1067	0.519717598	0.772822496
PIK3CB	1	1070	0.544087487	0.772822496
PLCG1	1	1070	0.544087487	0.772822496

ARID1A	4	1067	0.521981616	0.772822496
ARID2	3	1068	0.513441089	0.772822496
CHEK2	6	1065	0.545006341	0.772822496
CDK12	3	1068	0.536148283	0.772822496
DDX10	1	1070	0.532846927	0.772822496
KAT6B	4	1067	0.504909856	0.772822496
NCOA4	1	1070	0.511570598	0.772822496
VAV1	3	1068	0.56143765	0.791035155
MAP3K1	3	1068	0.561065767	0.791035155
TCF12	1	1070	0.598005533	0.798567543
TMPRSS2	1	1070	0.572325018	0.798567543
CTNNB1	4	1067	0.599045231	0.798567543
CHD4	4	1067	0.584878291	0.798567543
ERBB3	3	1068	0.575798616	0.798567543
FCGR2B	3	1068	0.59449481	0.798567543
FOXP1	1	1070	0.573777874	0.798567543
NUTM1	2	1069	0.592611009	0.798567543
SETBP1	3	1068	0.589410983	0.798567543
REL	3	1068	0.59937836	0.798567543
CDH10	10	1061	0.573229023	0.798567543
GPC5	4	1067	0.582630646	0.798567543
PPP2R1A	3	1068	0.596543744	0.798567543
STAG2	2	1069	0.594507495	0.798567543
ELF4	1	1070	0.594507495	0.798567543
NBEA	8	1063	0.577060042	0.798567543
RGPD3	8	1063	0.584736154	0.798567543
TBL1XR1	1	1070	0.598005533	0.798567543
PICALM	1	1070	0.617980156	0.805704073
PDE4DIP	14	1057	0.621177743	0.805704073
CD74	1	1070	0.617980156	0.805704073
JAK3	3	1068	0.608664242	0.805704073
BAZ1A	3	1068	0.607145118	0.805704073
CNOT3	1	1070	0.617980156	0.805704073
PBRM1	1	1070	0.617980156	0.805704073
FES	1	1070	0.612147297	0.805704073
RAD21	3	1068	0.619836885	0.805704073
NUP98	4	1067	0.631396805	0.816557158
PPFIBP1	1	1070	0.636889808	0.817169642
CTNNA2	1	1070	0.639282373	0.817169642
CCNE1	1	1070	0.639282373	0.817169642
RB1	11	1060	0.63519075	0.817169642
RPL5	3	1068	0.641543096	0.817689322
NSD1	2	1069	0.644004745	0.818461361

KMT2D	16	1055	0.647030317	0.819943591
MAFB	1	1070	0.656479784	0.829534627
BCL11A	2	1069	0.662143907	0.831924396
PTPN13	3	1068	0.660408531	0.831924396
DGCR8	3	1068	0.671946231	0.841841727
CIC	4	1067	0.676888106	0.843241962
CREBBP	7	1064	0.676575531	0.843241962
MYH9	5	1066	0.685438476	0.846718117
ROBO2	2	1069	0.684812171	0.846718117
CNBD1	3	1068	0.685045979	0.846718117
EWSR1	2	1069	0.689249407	0.849047454
ZEB1	2	1069	0.699754145	0.857427256
ASXL1	3	1068	0.699940617	0.857427256
CACNA1D	4	1067	0.709502032	0.866732399
SFPQ	2	1069	0.712529485	0.868026251
TET2	2	1069	0.719189424	0.873725995
BCL9	4	1067	0.728153397	0.877365159
BCL11B	1	1070	0.727984809	0.877365159
FLNA	2	1069	0.726243988	0.877365159
SND1	2	1069	0.736117255	0.884544168
RUNX1T1	5	1066	0.743349205	0.890807064
CNTRL	2	1069	0.764816805	0.897012849
RABEP1	2	1069	0.769604921	0.897012849
RBM15	2	1069	0.770961028	0.897012849
ROS1	3	1068	0.752306024	0.897012849
ATRX	4	1067	0.761610146	0.897012849
BAP1	5	1066	0.765973562	0.897012849
CREB3L1	3	1068	0.762353385	0.897012849
ERCC4	6	1065	0.772936242	0.897012849
FANCD2	4	1067	0.768173428	0.897012849
PTPRD	7	1064	0.755302641	0.897012849
TSC2	3	1068	0.758499171	0.897012849
NOTCH1	43	1028	0.767069931	0.897012849
UBR5	5	1066	0.778763292	0.897748641
KMT2C	21	1050	0.779677391	0.897748641
PAX5	4	1067	0.776807469	0.897748641
MLLT1	1	1070	0.782201891	0.898309984
TLX1	2	1069	0.784937837	0.899110614
DICER1	5	1066	0.786977704	0.899111832
FANCC	2	1069	0.790870493	0.901224516
FANCA	2	1069	0.800016498	0.909152817
LATS2	2	1069	0.801951125	0.909152817
KIF5B	3	1068	0.823078687	0.911288132

NCOA1	2	1069	0.821761104	0.911288132
PRCC	2	1069	0.830698025	0.911288132
MUC16	60	1011	0.823632098	0.911288132
PREX2	5	1066	0.822973458	0.911288132
PIK3CA	14	1057	0.828891736	0.911288132
USP6	7	1064	0.813689541	0.911288132
NF2	3	1068	0.827627504	0.911288132
NCOR2	4	1067	0.82387791	0.911288132
PMS2	2	1069	0.818769735	0.911288132
SPOP	2	1069	0.807988839	0.911288132
NFE2L2	16	1055	0.827728096	0.911288132
TP53	275	796	0.815062886	0.911288132
BRIP1	4	1067	0.835663459	0.91446051
SMAD4	2	1069	0.842656278	0.919830245
DROSHA	3	1068	0.850315851	0.921381671
XPC	2	1069	0.852351719	0.921381671
ZMYM3	2	1069	0.852574437	0.921381671
APOBEC3B	2	1069	0.854524044	0.921381671
CTNND1	2	1069	0.850298949	0.921381671
CDKN1A	2	1069	0.864646094	0.930021774
MED12	2	1069	0.86991581	0.933413314
NOTCH2	10	1061	0.87276531	0.93419782
DNAJB1	2	1069	0.888632665	0.944572154
SNX29	3	1068	0.893083616	0.944572154
SEPT9	2	1069	0.903876302	0.944572154
SRGAP3	2	1069	0.889025378	0.944572154
AFF4	2	1069	0.903424176	0.944572154
CTCF	2	1069	0.898114361	0.944572154
LATS1	1	1070	0.896764362	0.944572154
NCOR1	6	1065	0.896345462	0.944572154
PRKCB	1	1070	0.896764362	0.944572154
TRIM24	2	1069	0.903760786	0.944572154
ERCC3	2	1069	0.910370899	0.945732247
LZTR1	2	1069	0.910370899	0.945732247
TERT	4	1067	0.911419966	0.945732247
AXIN2	3	1068	0.920329582	0.948283518
TGFBR2	7	1064	0.920315358	0.948283518
ZBTB16	2	1069	0.917353232	0.948283518
CTNND2	9	1062	0.934944865	0.961097169
KDM5A	5	1066	0.945186626	0.96525294
ATR	6	1065	0.943590945	0.96525294
PRF1	2	1069	0.945553901	0.96525294
KDR	4	1067	0.950196656	0.965522408

EPAS1	5	1066	0.949383429	0.965522408
NUMA1	4	1067	0.954451303	0.967616148
CUL3	2	1069	0.965211102	0.972231562
DCC	8	1063	0.965617742	0.972231562
KDM6A	7	1064	0.963457533	0.972231562
KIAA1549	5	1066	0.977185832	0.979406709
ASXL2	3	1068	0.975342402	0.979406709
PTPRB	7	1064	0.987257049	0.987257049

ITable S6i. Association between CCA of genes undering SBS5* signature and prognosis.

**GeneName**	**CCA>=0.06**	**CCA<0.06**	**pvalue**	**qvalue(fdr)**
**PRDM1**	**7**	**1064**	**0.00023899**	**0.008252196**
**RNF213**	**19**	**1052**	**0.001486106**	**0.037928007**
TCEA1	1	1070	1.29E-14	1.08E-12
CCND1	1	1070	1.29E-14	1.08E-12
FGFR1	1	1070	1.29E-14	1.08E-12
MAP2K2	1	1070	1.29E-14	1.08E-12
PRKACA	1	1070	1.29E-14	1.08E-12
FANCG	1	1070	1.29E-14	1.08E-12
FHIT	1	1070	1.29E-14	1.08E-12
FGFR1OP	1	1070	3.34E-10	2.45E-08
NUTM2B	2	1069	7.84E-09	4.60E-07
CDH1	3	1068	7.84E-09	4.60E-07
SH3GL1	1	1070	1.13E-07	5.50E-06
FOXO1	1	1070	1.13E-07	5.50E-06
ACSL6	2	1069	8.30E-07	3.75E-05
TFEB	2	1069	1.79E-06	7.51E-05
STK11	2	1069	6.69E-06	0.000261652
LCK	3	1068	6.53E-05	0.002394498
EZR	1	1070	0.000333525	0.00978897
CD79B	1	1070	0.000333525	0.00978897
TRAF7	1	1070	0.000333525	0.00978897
PPM1D	3	1068	0.0004515	0.012620498
SIX2	2	1069	0.000477342	0.012736348
NUTM2D	1	1070	0.002636768	0.057325292
IDH2	1	1070	0.002636768	0.057325292
CDX2	1	1070	0.002636768	0.057325292
MSH2	3	1068	0.002560151	0.057325292
HIST1H3B	2	1069	0.003131954	0.065659182
SMARCE1	2	1069	0.004315683	0.087355383
ZNF384	3	1068	0.004805303	0.094023758
PRPF40B	2	1069	0.006070642	0.114950548
FEN1	2	1069	0.00705645	0.129441749
H3F3B	4	1067	0.008054573	0.139059828

NFE2L2	42	1029	0.008036628	0.139059828
KAT6B	10	1061	0.008460727	0.141493615
MEN1	2	1069	0.008677632	0.141493615
OLIG2	2	1069	0.009413082	0.149337269
TLX1	1	1070	0.009835352	0.151930311
RHOA	2	1069	0.01200519	0.180693506
PAX3	2	1069	0.013429288	0.197074808
CSF1R	2	1069	0.013828012	0.197976659
GNA11	2	1069	0.015348536	0.214514067
BIRC6	16	1055	0.018852029	0.257352122
CARS	1	1070	0.021319051	0.284415526
VHL	1	1070	0.022122331	0.288573513
SRC	2	1069	0.023439912	0.299113665
SNX29	7	1064	0.024152824	0.301653358
SETD2	9	1062	0.027878063	0.340925481
SPOP	2	1069	0.029200871	0.349814512
CCNC	1	1070	0.035853933	0.397099218
FANCE	3	1068	0.034415831	0.397099218
DDB2	1	1070	0.035853933	0.397099218
RUNX1	1	1070	0.035853933	0.397099218
PTK6	3	1068	0.038596689	0.419560301
UBR5	16	1055	0.039853701	0.425347685
CIC	7	1064	0.041039521	0.430182117
CCDC6	2	1069	0.043399208	0.446935704
MSH6	9	1062	0.045288566	0.458351526
CSMD3	90	981	0.046077281	0.458429899
SDHC	3	1068	0.050346403	0.492555643
IDH1	2	1069	0.05229001	0.503184194
COX6C	1	1070	0.055627619	0.504144345
KAT6A	8	1063	0.055825183	0.504144345
CASP8	5	1066	0.055392138	0.504144345
GATA1	1	1070	0.055627619	0.504144345
ETV5	5	1066	0.058582687	0.51232141
WNK2	6	1065	0.058152453	0.51232141
KDM6A	21	1050	0.059348988	0.51232141
SALL4	2	1069	0.063237388	0.537976038
PCM1	7	1064	0.066897493	0.560983263
NIN	6	1065	0.071626477	0.592179462
PWWP2A	2	1069	0.078023764	0.599295481
SS18	2	1069	0.074111832	0.599295481
TCF12	2	1069	0.077855202	0.599295481
MET	5	1066	0.079039261	0.599295481
EXT2	1	1070	0.079633812	0.599295481

TNFAIP3	1	1070	0.079633812	0.599295481
BTK	1	1070	0.07656776	0.599295481
SFRP4	8	1063	0.082626283	0.613944658
ASXL1	8	1063	0.086063736	0.631492663
AFF1	1	1070	0.097251898	0.647056622
GMPS	4	1067	0.099712027	0.647056622
PDE4DIP	35	1036	0.101132592	0.647056622
ERBB3	7	1064	0.100017219	0.647056622
H3F3A	4	1067	0.092482994	0.647056622
NRAS	1	1070	0.093904564	0.647056622
TAL1	2	1069	0.098935024	0.647056622
HNF1A	3	1068	0.092862778	0.647056622
ZNRF3	1	1070	0.102358298	0.647056622
EPHA7	11	1060	0.091320164	0.647056622
LEF1	4	1067	0.089383516	0.647056622
NKX2-1	4	1067	0.101945536	0.647056622
PABPC1	9	1062	0.102514933	0.647056622
EPS15	1	1070	0.105818107	0.656826576
STAG1	4	1067	0.106300723	0.656826576
FOXA1	4	1067	0.117175901	0.7090954
MAP3K13	5	1066	0.116617246	0.7090954
BCR	10	1061	0.122060252	0.731116
BCL3	1	1070	0.125277811	0.735380753
IL6ST	1	1070	0.125277811	0.735380753
NUMA1	7	1064	0.129092106	0.742912415
NUP214	4	1067	0.128638939	0.742912415
ABL1	4	1067	0.134130912	0.749855671
BRCA2	12	1059	0.133791322	0.749855671
PRDM2	14	1057	0.133759423	0.749855671
FH	4	1067	0.136656791	0.75676921
CHST11	3	1068	0.142109303	0.762278097
EGFR	9	1062	0.142806156	0.762278097
FGFR4	5	1066	0.14019561	0.762278097
MYC	2	1069	0.142845981	0.762278097
FLT3	5	1066	0.145894765	0.768590611
AMER1	2	1069	0.146647612	0.768590611
SLC45A3	3	1068	0.157852818	0.772163366
CTNND2	23	1048	0.154762339	0.772163366
HNRNPA2B1	3	1068	0.157810102	0.772163366
STIL	9	1062	0.155046827	0.772163366
KIT	4	1067	0.155324115	0.772163366
ITGAV	3	1068	0.155449734	0.772163366
STAT5B	2	1069	0.151503752	0.772163366

TRIM24	6	1065	0.153942195	0.772163366
KDM5A	3	1068	0.160968696	0.774496923
SMARCA4	5	1066	0.160105546	0.774496923
ERBB4	20	1051	0.162740443	0.776655612
SEPT5	3	1068	0.171497894	0.7965255
ZMYM2	3	1068	0.172029892	0.7965255
LRIG3	6	1065	0.172331752	0.7965255
JAK1	3	1068	0.168962789	0.7965255
NCOA2	12	1059	0.174305274	0.799353094
NT5C2	4	1067	0.179194386	0.809131573
TCF3	3	1068	0.179153449	0.809131573
SUFU	6	1065	0.181483303	0.813211443
AKT3	3	1068	0.18859962	0.81532579
CDK12	15	1056	0.188900013	0.81532579
FBXW7	36	1035	0.185722706	0.81532579
LARP4B	3	1068	0.188871371	0.81532579
NCOA4	3	1068	0.185617752	0.81532579
IKZF1	6	1065	0.190417408	0.815876049
RET	3	1068	0.195420798	0.825266247
KLF4	2	1069	0.194722041	0.825266247
ERCC2	3	1068	0.198082068	0.830529815
AKAP9	16	1055	0.276504474	0.834556263
ACSL3	7	1064	0.403313739	0.834556263
ALDH2	1	1070	0.337248505	0.834556263
ASPSCR1	1	1070	0.370271862	0.834556263
BCL7A	1	1070	0.385922639	0.834556263
CLIP1	6	1065	0.373115329	0.834556263
COL2A1	6	1065	0.219521313	0.834556263
FNBP1	4	1067	0.228750466	0.834556263
GOLGA5	1	1070	0.453302589	0.834556263
HOOK3	1	1070	0.395300839	0.834556263
IL21R	1	1070	0.417847639	0.834556263
JAZF1	1	1070	0.385922639	0.834556263
KTN1	11	1060	0.318110814	0.834556263
LSM14A	1	1070	0.395300839	0.834556263
HLA-A	1	1070	0.417847639	0.834556263
MLLT3	1	1070	0.339789994	0.834556263
MLLT6	9	1062	0.433829703	0.834556263
MYO5A	4	1067	0.359348128	0.834556263
MYH11	13	1058	0.244859241	0.834556263
NFIB	2	1069	0.417847639	0.834556263
NCOA1	4	1067	0.426873477	0.834556263
PAX8	2	1069	0.255992587	0.834556263

RBM15	3	1068	0.225401296	0.834556263
SRGAP3	2	1069	0.272521984	0.834556263
TOP1	2	1069	0.257253975	0.834556263
TMPRSS2	2	1069	0.315454632	0.834556263
TPM4	1	1070	0.395300839	0.834556263
ZCCHC8	1	1070	0.314749166	0.834556263
ACVR1	1	1070	0.411623741	0.834556263
AFF3	15	1056	0.260825698	0.834556263
ARAF	4	1067	0.44402072	0.834556263
BCL2L12	1	1070	0.411623741	0.834556263
BRD4	6	1065	0.44907226	0.834556263
CDH17	2	1069	0.285998539	0.834556263
CREB3L2	1	1070	0.440780193	0.834556263
CTNNA2	12	1059	0.266999646	0.834556263
CCR4	1	1070	0.385922639	0.834556263
CCR7	2	1069	0.413530834	0.834556263
CD28	1	1070	0.440780193	0.834556263
CD74	2	1069	0.380809587	0.834556263
CSF3R	1	1070	0.402894233	0.834556263
CDK4	2	1069	0.394938992	0.834556263
DDR2	5	1066	0.22456323	0.834556263
ETV1	9	1062	0.219731767	0.834556263
EWSR1	3	1068	0.328787707	0.834556263
XPO1	5	1066	0.368569102	0.834556263
FUBP1	3	1068	0.232033344	0.834556263
FOXP1	3	1068	0.269837564	0.834556263
FOXR1	1	1070	0.263918441	0.834556263
FLI1	1	1070	0.385922639	0.834556263
GATA2	2	1069	0.264806643	0.834556263
GRM3	14	1057	0.39485013	0.834556263
GNAQ	2	1069	0.258061381	0.834556263
HOXA13	1	1070	0.407619244	0.834556263
HOXC13	1	1070	0.411623741	0.834556263
JAK2	5	1066	0.299360919	0.834556263
JAK3	4	1067	0.369617192	0.834556263
KMT2A	15	1056	0.401976283	0.834556263
MTCP1	1	1070	0.453302589	0.834556263
MDM2	1	1070	0.402894233	0.834556263
MITF	4	1067	0.204532415	0.834556263
MUC16	116	955	0.276737494	0.834556263
MUC4	5	1066	0.206929763	0.834556263
MALT1	2	1069	0.394058235	0.834556263
MSI2	1	1070	0.439748565	0.834556263

PIK3CB	8	1063	0.20343512	0.834556263
PLCG1	3	1068	0.363100809	0.834556263
PDGFRA	4	1067	0.424607495	0.834556263
PTPN11	4	1067	0.326146042	0.834556263
SGK1	1	1070	0.411623741	0.834556263
SETDB1	3	1068	0.351025341	0.834556263
SET	2	1069	0.266178119	0.834556263
SIX1	1	1070	0.411623741	0.834556263
SMO	6	1065	0.332855886	0.834556263
SF3B1	9	1062	0.322256226	0.834556263
SSX1	1	1070	0.430792583	0.834556263
TAL2	1	1070	0.427152138	0.834556263
TCL1A	2	1069	0.255953846	0.834556263
TLX3	1	1070	0.391950155	0.834556263
TFE3	1	1070	0.373078968	0.834556263
USP8	2	1069	0.237468848	0.834556263
MAF	1	1070	0.413219358	0.834556263
MAFB	2	1069	0.365758967	0.834556263
MYCL	1	1070	0.411623741	0.834556263
MYCN	1	1070	0.453302589	0.834556263
RAF1	1	1070	0.453302589	0.834556263
ABI1	1	1070	0.314749166	0.834556263
ACVR2A	1	1070	0.402894233	0.834556263
ASXL2	5	1066	0.346266972	0.834556263
AXIN1	6	1065	0.421951464	0.834556263
BAX	2	1069	0.385922639	0.834556263
BARD1	4	1067	0.400392162	0.834556263
BUB1B	3	1068	0.241108389	0.834556263
CDH11	12	1059	0.20533816	0.834556263
CAMTA1	10	1061	0.399084679	0.834556263
CEBPA	1	1070	0.41162043	0.834556263
CHD2	11	1060	0.338740203	0.834556263
CLTC	3	1068	0.397192571	0.834556263
CLTCL1	6	1065	0.434925546	0.834556263
CBFB	1	1070	0.430792583	0.834556263
CCNB1IP1	2	1069	0.259943991	0.834556263
CDKN2A	36	1035	0.377909577	0.834556263
DDX3X	1	1070	0.290250051	0.834556263
DNMT3A	3	1068	0.232033344	0.834556263
POLG	4	1067	0.368524407	0.834556263
ELL	1	1070	0.419287106	0.834556263
ETV6	3	1068	0.252564174	0.834556263
EIF3E	1	1070	0.411623741	0.834556263

ERCC4	8	1063	0.319801383	0.834556263
BRCA1	5	1066	0.386747134	0.834556263
CYLD	6	1065	0.363112299	0.834556263
FANCA	5	1066	0.217541241	0.834556263
FANCD2	10	1061	0.24179258	0.834556263
FAT4	34	1037	0.315958637	0.834556263
FBLN2	5	1066	0.368557264	0.834556263
FUS	2	1069	0.247578126	0.834556263
PTCH1	15	1056	0.285522933	0.834556263
ID3	2	1069	0.27156427	0.834556263
IGF2BP2	3	1068	0.25962508	0.834556263
KEAP1	20	1051	0.383692617	0.834556263
EXT1	2	1069	0.244293005	0.834556263
NAB2	2	1069	0.254352404	0.834556263
NBN	5	1066	0.346840935	0.834556263
NDRG1	1	1070	0.407619244	0.834556263
NCOR1	13	1058	0.357407704	0.834556263
MGMT	5	1066	0.247783212	0.834556263
PHOX2B	3	1068	0.216162919	0.834556263
PIK3R1	2	1069	0.223500184	0.834556263
PML	2	1069	0.259903199	0.834556263
PPP2R1A	6	1065	0.214154806	0.834556263
PTPRT	13	1058	0.345586535	0.834556263
RAD51B	1	1070	0.453302589	0.834556263
RANBP2	9	1062	0.422565203	0.834556263
ARHGAP26	4	1067	0.409232095	0.834556263
RPL10	1	1070	0.445739552	0.834556263
RFWD3	1	1070	0.427152138	0.834556263
RBM10	4	1067	0.443098685	0.834556263
SBDS	1	1070	0.417847639	0.834556263
SMAD2	1	1070	0.385922639	0.834556263
SMAD3	1	1070	0.385922639	0.834556263
SMAD4	4	1067	0.451938023	0.834556263
SOX21	1	1070	0.411623741	0.834556263
SMC1A	1	1070	0.407619244	0.834556263
SDHB	1	1070	0.373078968	0.834556263
TET2	9	1062	0.453532279	0.834556263
TMEM127	1	1070	0.373078968	0.834556263
TRIM33	7	1064	0.311111097	0.834556263
TSC1	6	1065	0.34888555	0.834556263
TNFRSF14	1	1070	0.395300839	0.834556263
YWHAE	1	1070	0.41162043	0.834556263
USP44	1	1070	0.417847639	0.834556263

WRN	7	1064	0.279979318	0.834556263
WIF1	1	1070	0.433111469	0.834556263
ZRSR2	2	1069	0.324202734	0.834556263
ZFHX3	25	1046	0.303966119	0.834556263
PATZ1	1	1070	0.41162043	0.834556263
ZNF331	6	1065	0.287958622	0.834556263
ATP1A1	1	1070	0.395300839	0.834556263
BCORL1	6	1065	0.437146787	0.834556263
BMP5	4	1067	0.451966909	0.834556263
CBLC	1	1070	0.395300839	0.834556263
CTNND1	3	1068	0.413651652	0.834556263
CD209	2	1069	0.257544622	0.834556263
CRNKL1	1	1070	0.370271862	0.834556263
CDKN1A	2	1069	0.257544622	0.834556263
CYP2C8	2	1069	0.321053112	0.834556263
DCAF12L2	9	1062	0.307937665	0.834556263
DAXX	1	1070	0.385922639	0.834556263
POLQ	11	1060	0.313675575	0.834556263
EPAS1	7	1064	0.425805201	0.834556263
EPHA3	12	1059	0.414060205	0.834556263
FKBP9	1	1070	0.231603482	0.834556263
GATA3	4	1067	0.447658076	0.834556263
NTRK1	5	1066	0.241631224	0.834556263
NFKB2	1	1070	0.407619244	0.834556263
PCBP1	3	1068	0.42241834	0.834556263
RGS7	15	1056	0.28562674	0.834556263
TBX3	7	1064	0.276526748	0.834556263
ZNF479	6	1065	0.271783735	0.834556263
ELF4	4	1067	0.457910106	0.838079488
TET1	8	1063	0.458302412	0.838079488
NSD1	9	1062	0.472794159	0.838511518
BCL11A	9	1062	0.46065431	0.838511518
CACNA1D	4	1067	0.464634434	0.838511518
CREB1	1	1070	0.468047515	0.838511518
GNAS	9	1062	0.468289006	0.838511518
NUTM1	5	1066	0.472500876	0.838511518
SND1	5	1066	0.46879954	0.838511518
BRAF	7	1064	0.469034719	0.838511518
ZEB1	9	1062	0.46396076	0.838511518
PAX5	6	1065	0.474232274	0.838511518
RGPD3	9	1062	0.474251829	0.838511518
PPFIBP1	2	1069	0.476550498	0.838565968
FANCC	4	1067	0.47713975	0.838565968

RSPO3	3	1068	0.480214247	0.841450038
PRF1	3	1068	0.484147129	0.845816561
WWTR1	3	1068	0.487568695	0.846753918
ELF3	1	1070	0.48639064	0.846753918
BMPR1A	1	1070	0.489147537	0.846989983
KMT2D	45	1026	0.492025529	0.849467604
ARID1B	11	1060	0.494135743	0.850609036
DROSHA	8	1063	0.497079091	0.853173761
JUN	3	1068	0.507848522	0.869116857
CRTC3	3	1068	0.512628373	0.871341092
HERPUD1	1	1070	0.512482662	0.871341092
FBXO11	3	1068	0.513601393	0.871341092
NTRK3	6	1065	0.516731336	0.874124766
EP300	54	1017	0.524217895	0.879268643
ARID2	11	1060	0.524265801	0.879268643
RAD21	5	1066	0.521790445	0.879268643
LCP1	1	1070	0.532846927	0.890984748
AFF4	9	1062	0.537323	0.890984748
BLM	4	1067	0.537138227	0.890984748
PMS1	4	1067	0.536453534	0.890984748
CHD4	11	1060	0.545413027	0.901851964
TFG	1	1070	0.548678618	0.904703226
BAZ1A	9	1062	0.552207653	0.905435454
MB21D2	4	1067	0.550858425	0.905435454
HSP90AA1	3	1068	0.576720022	0.912961911
MSN	1	1070	0.594507495	0.912961911
NCKIPSD	2	1069	0.594507495	0.912961911
NONO	1	1070	0.587849196	0.912961911
RPN1	3	1068	0.584979266	0.912961911
A1CF	5	1066	0.574901995	0.912961911
CD79A	3	1068	0.561681471	0.912961911
CRTC1	3	1068	0.589416747	0.912961911
FCGR2B	3	1068	0.59449481	0.912961911
FLT4	5	1066	0.56790286	0.912961911
LPP	14	1057	0.559498447	0.912961911
MAP2K1	1	1070	0.572325018	0.912961911
NR4A3	3	1068	0.580084908	0.912961911
PREX2	13	1058	0.597346938	0.912961911
PRDM16	6	1065	0.594657736	0.912961911
SKI	1	1070	0.594548253	0.912961911
TRRAP	16	1055	0.587786153	0.912961911
CUL3	12	1059	0.598791032	0.912961911
DICER1	8	1063	0.57938753	0.912961911

POLE	6	1065	0.598148837	0.912961911
PTPRK	8	1063	0.587067683	0.912961911
ARHGEF10	6	1065	0.597586254	0.912961911
SLC34A2	4	1067	0.569504471	0.912961911
ANK1	8	1063	0.593867478	0.912961911
ARNT	3	1068	0.587070765	0.912961911
FAM135B	33	1038	0.565478209	0.912961911
WT1	3	1068	0.56814597	0.912961911
EML4	3	1068	0.60363107	0.91691934
ABL2	4	1067	0.604510706	0.91691934
TPR	12	1059	0.608381142	0.920411677
MTOR	10	1061	0.61118577	0.920864894
TPM3	4	1067	0.611818243	0.920864894
S100A7	1	1070	0.617980156	0.923940283
CNOT3	1	1070	0.617980156	0.923940283
SUZ12	3	1068	0.618583528	0.923940283
CARD11	9	1062	0.623522757	0.926602173
TP63	8	1063	0.62236012	0.926602173
DGCR8	8	1063	0.62511107	0.926616662
PIM1	1	1070	0.631164581	0.933233273
SS18L1	1	1070	0.648840211	0.938101487
TFPT	1	1070	0.647750187	0.938101487
CCNE1	1	1070	0.639282373	0.938101487
CYSLTR2	1	1070	0.646658489	0.938101487
FCRL4	4	1067	0.642871836	0.938101487
PIK3CA	31	1040	0.637909634	0.938101487
ARHGAP5	13	1058	0.648466217	0.938101487
ATR	13	1058	0.63907066	0.938101487
ESR1	3	1068	0.648409779	0.938101487
CASP3	1	1070	0.652057651	0.940436956
NRG1	3	1068	0.655061005	0.942452965
EIF1AX	3	1068	0.660389721	0.947796494
STAT3	4	1067	0.664402911	0.94891608
ARHGEF10L	3	1068	0.663053464	0.94891608
PER1	3	1068	0.672679833	0.958405491
CLP1	4	1067	0.676813674	0.961960355
CNTRL	7	1064	0.67882187	0.96248415
FANCF	1	1070	0.683259216	0.966441349
COL3A1	20	1051	0.715010529	0.968026327
EIF4A2	3	1068	0.741432628	0.968026327
HSP90AB1	3	1068	0.749793807	0.968026327
RALGDS	5	1066	0.731755613	0.968026327
STRN	2	1069	0.708476258	0.968026327

THRAP3	2	1069	0.739050282	0.968026327
AR	6	1065	0.704185201	0.968026327
CXCR4	2	1069	0.697990334	0.968026327
DEK	3	1068	0.713251106	0.968026327
FGFR3	1	1070	0.725272733	0.968026327
MECOM	5	1066	0.710399639	0.968026327
MLLT10	4	1067	0.704398296	0.968026327
NFATC2	3	1068	0.707161354	0.968026327
NPM1	3	1068	0.699040524	0.968026327
POU2AF1	3	1068	0.709025491	0.968026327
TAF15	2	1069	0.713057301	0.968026327
KRAS	5	1066	0.747959328	0.968026327
ZNF521	17	1054	0.728005667	0.968026327
CDH10	20	1051	0.750344087	0.968026327
CIITA	6	1065	0.708561987	0.968026327
MLH1	5	1066	0.698035118	0.968026327
EBF1	9	1062	0.733498636	0.968026327
FAS	2	1069	0.748441701	0.968026327
LATS1	6	1065	0.699378662	0.968026327
KMT2C	39	1032	0.728951286	0.968026327
MED12	7	1064	0.733218052	0.968026327
NF1	11	1060	0.745213032	0.968026327
PBRM1	8	1063	0.717179454	0.968026327
RB1	27	1044	0.740429541	0.968026327
RNF43	2	1069	0.693595474	0.968026327
ROBO2	3	1068	0.742284396	0.968026327
SFPQ	2	1069	0.712529485	0.968026327
SMARCB1	4	1067	0.717616775	0.968026327
BCL11B	2	1069	0.746989094	0.968026327
BCLAF1	19	1052	0.724539303	0.968026327
DCC	22	1049	0.748267283	0.968026327
FAT3	43	1028	0.708572869	0.968026327
NOTCH1	108	963	0.699861746	0.968026327
PRKAR1A	1	1070	0.727984809	0.968026327
RUNX1T1	11	1060	0.734771501	0.968026327
MYH9	14	1057	0.754268126	0.970700801
N4BP2	3	1068	0.755724474	0.970700801
PICALM	1	1070	0.764573816	0.973546269
STAT6	3	1068	0.759808973	0.973546269
ATP2B3	4	1067	0.76337768	0.973546269
CREB3L1	3	1068	0.762353385	0.973546269
MLLT1	1	1070	0.782201891	0.974296737
CTNNB1	6	1065	0.769302312	0.974296737

MAML2	3	1068	0.779927015	0.974296737
MN1	6	1065	0.782426151	0.974296737
RARA	2	1069	0.782140719	0.974296737
TSHR	3	1068	0.773283679	0.974296737
ATRX	7	1064	0.784094557	0.974296737
CTCF	4	1067	0.788400255	0.974296737
LRP1B	66	1005	0.786309321	0.974296737
LZTR1	5	1066	0.772800575	0.974296737
SDHA	2	1069	0.774518029	0.974296737
TGFBR2	10	1061	0.77226549	0.974296737
FES	2	1069	0.787161576	0.974296737
ZNF429	11	1060	0.776838846	0.974296737
PLAG1	4	1067	0.792571715	0.975345066
PRKCB	3	1068	0.791337005	0.975345066
GPC5	5	1066	0.795104562	0.976415016
FLCN	2	1069	0.79732377	0.977096144
ELN	3	1068	0.800046941	0.978390738
LATS2	2	1069	0.801951125	0.978680479
ATIC	3	1068	0.864142003	0.98016517
CEP89	5	1066	0.847143451	0.98016517
GAS7	5	1066	0.815408119	0.98016517
KIF5B	3	1068	0.823078687	0.98016517
LIFR	6	1065	0.887770504	0.98016517
PAX7	4	1067	0.858291297	0.98016517
PRCC	2	1069	0.830698025	0.98016517
SPECC1	4	1067	0.848687662	0.98016517
TRIP11	4	1067	0.817918955	0.98016517
VAV1	4	1067	0.851132288	0.98016517
ACKR3	2	1069	0.854524044	0.98016517
BCL9	6	1065	0.84684946	0.98016517
DDX5	3	1068	0.903354952	0.98016517
GLI1	4	1067	0.878033875	0.98016517
HOXD13	3	1068	0.902221259	0.98016517
HIF1A	4	1067	0.872516075	0.98016517
IL7R	4	1067	0.813133047	0.98016517
MDM4	2	1069	0.869396332	0.98016517
MACC1	7	1064	0.892600818	0.98016517
NUP98	5	1066	0.896970886	0.98016517
TNC	5	1066	0.846560952	0.98016517
TNFRSF17	2	1069	0.866473972	0.98016517
USP6	17	1054	0.820950167	0.98016517
KDR	10	1061	0.874386636	0.98016517
ERBB2	3	1068	0.871149	0.98016517

ERG	2	1069	0.849385008	0.98016517
ROS1	8	1063	0.889660564	0.98016517
WAS	3	1068	0.899232203	0.98016517
ATM	11	1060	0.89245837	0.98016517
AXIN2	7	1064	0.856985984	0.98016517
BAP1	6	1065	0.873432848	0.98016517
CBFA2T3	2	1069	0.863416411	0.98016517
DDX10	3	1068	0.878881216	0.98016517
ERCC3	4	1067	0.847161405	0.98016517
ERCC5	6	1065	0.884077576	0.98016517
GRIN2A	9	1062	0.887300974	0.98016517
KDM5C	6	1065	0.837611365	0.98016517
MAX	3	1068	0.885573957	0.98016517
PALB2	12	1059	0.870898974	0.98016517
PPARG	2	1069	0.828767425	0.98016517
PMS2	2	1069	0.818769735	0.98016517
PTPN13	7	1064	0.895148383	0.98016517
PTPRD	17	1054	0.815406543	0.98016517
ARHGEF12	4	1067	0.833396849	0.98016517
RSPO2	4	1067	0.859824085	0.98016517
SETD1B	11	1060	0.896932041	0.98016517
SPEN	25	1046	0.815010332	0.98016517
SMARCD1	2	1069	0.847103414	0.98016517
TSC2	7	1064	0.838652655	0.98016517
XPC	2	1069	0.852351719	0.98016517
ZBTB16	4	1067	0.885058501	0.98016517
ZMYM3	3	1068	0.874137328	0.98016517
APOBEC3B	2	1069	0.854524044	0.98016517
CNBD1	5	1066	0.84871877	0.98016517
EZH2	6	1065	0.870438273	0.98016517
ECT2L	3	1068	0.806861579	0.98016517
FAM47C	7	1064	0.880911651	0.98016517
NBEA	18	1053	0.885956158	0.98016517
TERT	6	1065	0.902778386	0.98016517
TP53	559	512	0.843989425	0.98016517
NACA	2	1069	0.910370899	0.985955198
OMD	2	1069	0.920208139	0.98617576
ALK	7	1064	0.923187519	0.98617576
APC	7	1064	0.924014767	0.98617576
NF2	3	1068	0.921597423	0.98617576
SH2B3	2	1069	0.923452734	0.98617576
BIRC3	3	1068	0.922903061	0.98617576
IRF4	2	1069	0.918759883	0.98617576

TBL1XR1	6	1065	0.91948056	0.98617576
ERC1	6	1065	0.932271662	0.98654668
MNX1	2	1069	0.941168894	0.98654668
RAC1	2	1069	0.928543613	0.98654668
SETBP1	6	1065	0.93843466	0.98654668
ARID1A	9	1062	0.934755781	0.98654668
DNM2	2	1069	0.940761033	0.98654668
PTEN	16	1055	0.927715796	0.98654668
PTPRC	6	1065	0.937314485	0.98654668
FOXO4	5	1066	0.93683122	0.98654668
MAP3K1	4	1067	0.936704252	0.98654668
FAM131B	2	1069	0.956562171	0.988141902
GPHN	3	1068	0.948576648	0.988141902
SEPT9	6	1065	0.959747902	0.988141902
FGFR2	5	1066	0.958707344	0.988141902
MAPK1	4	1067	0.950856258	0.988141902
PDGFRB	4	1067	0.957277372	0.988141902
MYB	2	1069	0.951713498	0.988141902
CHEK2	15	1056	0.961188942	0.988141902
FAT1	50	1021	0.961207881	0.988141902
FLNA	10	1061	0.947841856	0.988141902
NOTCH2	34	1037	0.957905602	0.988141902
PTPRB	16	1055	0.964613819	0.989909636
COL1A1	8	1063	0.979455729	0.990604626
DNAJB1	4	1067	0.980479196	0.990604626
KIAA1549	13	1058	0.967212002	0.990604626
RABEP1	4	1067	0.978698135	0.990604626
TEC	2	1069	0.974395135	0.990604626
TCF7L2	2	1069	0.969886517	0.990604626
BRIP1	6	1065	0.977511048	0.990604626
CBLB	5	1066	0.977280069	0.990604626
CREBBP	20	1051	0.976653341	0.990604626
BRD3	2	1069	0.990905111	0.995995377
REL	5	1066	0.99071689	0.995995377
RPL5	2	1069	0.990905111	0.995995377
CNTNAP2	21	1050	0.99352688	0.996923553
IL2	2	1069	0.999626143	0.999626143
NCOR2	11	1060	0.998215408	0.999626143

Table S6j. Association between CCA of genes undering SBS17b* signature and prognosis.

GeneName	CCA>=0.06	CCA<0.06	pvalue	qvalue(fdr)
NUTM2B	1	1070	1.29E-14	1.50E-12
TCEA1	1	1070	1.29E-14	1.50E-12

MDM4	1	1070	1.29E-14	1.50E-12
ABL2	1	1070	2.07E-09	1.80E-07
TFEB	1	1070	1.13E-07	7.85E-06
LCP1	1	1070	3.07E-07	1.79E-05
ATP2B3	1	1070	3.27E-06	0.000142792
CDKN2A	1	1070	3.27E-06	0.000142792
SETDB1	1	1070	0.000333525	0.01058185
PPARG	1	1070	0.000333525	0.01058185
STK11	1	1070	0.000333525	0.01058185
MYH9	1	1070	0.000632926	0.018407606
SETBP1	3	1068	0.001534429	0.041193528
BARD1	1	1070	0.002636768	0.065730862
MUC4	1	1070	0.002852382	0.06636543
NCOA1	1	1070	0.009835352	0.201913997
CTNND2	1	1070	0.009835352	0.201913997
TFG	1	1070	0.010869078	0.210739338
FANCD2	5	1066	0.011859666	0.217843338
TSHR	2	1069	0.015059893	0.262795127
KAT6A	2	1069	0.017597637	0.292455964
TRIP11	1	1070	0.021319051	0.310014538
NFATC2	1	1070	0.021319051	0.310014538
POLG	1	1070	0.021319051	0.310014538
NFE2L2	13	1058	0.024285924	0.339031505
CEP89	1	1070	0.035853933	0.379182501
RABEP1	3	1068	0.030027374	0.379182501
ETV5	1	1070	0.035853933	0.379182501
BAP1	1	1070	0.035853933	0.379182501
ARHGEF12	1	1070	0.035853933	0.379182501
EZH2	1	1070	0.035853933	0.379182501
FOXO4	1	1070	0.035853933	0.379182501
LEF1	1	1070	0.035853933	0.379182501
AKAP9	4	1067	0.037587771	0.385827415
NBN	2	1069	0.040649747	0.394076715
MB21D2	2	1069	0.039718489	0.394076715
ETV1	2	1069	0.048176354	0.454420206
PIK3CA	8	1063	0.052479461	0.481982415
MTOR	1	1070	0.07656776	0.661719055
EXT2	1	1070	0.079633812	0.661719055
SMAD4	1	1070	0.07656776	0.661719055
TNFAIP3	1	1070	0.079633812	0.661719055
NRAS	1	1070	0.093904564	0.700107623
NCOA2	2	1069	0.093904564	0.700107623
SRC	1	1070	0.093904564	0.700107623

RANBP2	2	1069	0.088719682	0.700107623
POLQ	4	1067	0.094283835	0.700107623
ACSL6	1	1070	0.187129562	0.716880663
AFF1	2	1069	0.324202734	0.716880663
ASPSCR1	1	1070	0.370271862	0.716880663
CNTRL	5	1066	0.348944165	0.716880663
CLP1	1	1070	0.411623741	0.716880663
COL3A1	5	1066	0.325908192	0.716880663
DNAJB1	1	1070	0.373078968	0.716880663
ERC1	1	1070	0.385922639	0.716880663
FIP1L1	1	1070	0.419287106	0.716880663
GOPC	1	1070	0.395300839	0.716880663
GAS7	2	1069	0.342338277	0.716880663
HSP90AA1	1	1070	0.266309994	0.716880663
HERPUD1	1	1070	0.440780193	0.716880663
IL2	1	1070	0.140401482	0.716880663
KIF5B	2	1069	0.368832432	0.716880663
LIFR	1	1070	0.400378314	0.716880663
LSM14A	1	1070	0.395300839	0.716880663
MSN	1	1070	0.125277811	0.716880663
MYO5A	2	1069	0.395300839	0.716880663
NIN	2	1069	0.351845493	0.716880663
NONO	1	1070	0.424618121	0.716880663
NUMA1	1	1070	0.385922639	0.716880663
NSD1	2	1069	0.311405117	0.716880663
NUP214	2	1069	0.265807737	0.716880663
NUTM2D	1	1070	0.413219358	0.716880663
PCM1	3	1068	0.202763786	0.716880663
PDE4DIP	7	1064	0.427448307	0.716880663
RNF213	2	1069	0.440780193	0.716880663
SNX29	1	1070	0.417847639	0.716880663
SLC45A3	1	1070	0.411623741	0.716880663
THRAP3	1	1070	0.419287106	0.716880663
TMPRSS2	1	1070	0.422021426	0.716880663
AR	1	1070	0.263918441	0.716880663
A1CF	2	1069	0.417847639	0.716880663
BIRC6	3	1068	0.296477055	0.716880663
BCL9	2	1069	0.380138011	0.716880663
CACNA1D	2	1069	0.240580143	0.716880663
CTNNB1	1	1070	0.411623741	0.716880663
CCR4	1	1070	0.385922639	0.716880663
CSF1R	1	1070	0.385922639	0.716880663
CSF3R	1	1070	0.402894233	0.716880663

DGCR8	4	1067	0.369615205	0.716880663
ELK4	1	1070	0.433111469	0.716880663
EGFR	1	1070	0.373078968	0.716880663
GATA2	1	1070	0.430792583	0.716880663
GNAS	1	1070	0.140401482	0.716880663
H3F3A	1	1070	0.427152138	0.716880663
HIF1A	2	1069	0.262919105	0.716880663
IKBKB	1	1070	0.41690198	0.716880663
JAK2	2	1069	0.326396151	0.716880663
KAT7	1	1070	0.433111469	0.716880663
MAML2	1	1070	0.413219358	0.716880663
MECOM	2	1069	0.222522916	0.716880663
MUC16	39	1032	0.258182296	0.716880663
MLLT10	1	1070	0.407619244	0.716880663
NUTM1	1	1070	0.445739552	0.716880663
PREX2	3	1068	0.425387765	0.716880663
PIK3CB	2	1069	0.279190422	0.716880663
PIM1	2	1069	0.33657118	0.716880663
PLAG1	2	1069	0.417847639	0.716880663
PRDM16	1	1070	0.417847639	0.716880663
RAC1	1	1070	0.385922639	0.716880663
USP6	4	1067	0.327545685	0.716880663
AKT3	2	1069	0.385922639	0.716880663
KDR	4	1067	0.171110408	0.716880663
ERBB2	1	1070	0.445739552	0.716880663
MYB	2	1069	0.445739552	0.716880663
WWTR1	1	1070	0.373078968	0.716880663
ZEB1	4	1067	0.416177428	0.716880663
EP300	10	1061	0.444177829	0.716880663
ASXL1	2	1069	0.23403774	0.716880663
APC	3	1068	0.29149094	0.716880663
ARID1A	1	1070	0.411623741	0.716880663
ARID1B	2	1069	0.244293005	0.716880663
ARID2	5	1066	0.389703109	0.716880663
CDH1	1	1070	0.337248505	0.716880663
CDH10	5	1066	0.189157831	0.716880663
CAMTA1	3	1068	0.256652898	0.716880663
CASP8	3	1068	0.288771803	0.716880663
CDC73	1	1070	0.231603482	0.716880663
CIITA	2	1069	0.424618121	0.716880663
CNTNAP2	4	1067	0.299920767	0.716880663
CUL3	4	1067	0.142583864	0.716880663
CCNB1IP1	2	1069	0.259943991	0.716880663

DICER1	1	1070	0.391950155	0.716880663
DNMT3A	1	1070	0.445739552	0.716880663
ETV6	1	1070	0.440780193	0.716880663
ERCC2	3	1068	0.255627265	0.716880663
ERCC4	1	1070	0.445739552	0.716880663
BRCA1	1	1070	0.419287106	0.716880663
BRCA2	5	1066	0.344995826	0.716880663
CYLD	4	1067	0.248306771	0.716880663
FANCA	1	1070	0.385922639	0.716880663
FAS	1	1070	0.385922639	0.716880663
FAT1	7	1064	0.366739563	0.716880663
FAT4	8	1063	0.239511731	0.716880663
FH	1	1070	0.385922639	0.716880663
FUS	1	1070	0.411623741	0.716880663
GPC5	1	1070	0.373078968	0.716880663
IKZF1	3	1068	0.407470976	0.716880663
KAT6B	1	1070	0.391950155	0.716880663
LRP1B	19	1052	0.431463862	0.716880663
LRIG3	1	1070	0.385922639	0.716880663
MED12	1	1070	0.417847639	0.716880663
EXT1	1	1070	0.411623741	0.716880663
MSH2	2	1069	0.164260904	0.716880663
MSH6	1	1070	0.395300839	0.716880663
MLF1	1	1070	0.395300839	0.716880663
NF1	4	1067	0.11081272	0.716880663
NCOR2	2	1069	0.411623741	0.716880663
PALB2	1	1070	0.337248505	0.716880663
PIK3R1	1	1070	0.373078968	0.716880663
PMS2	1	1070	0.391950155	0.716880663
POLE	1	1070	0.395300839	0.716880663
PML	3	1068	0.15656234	0.716880663
PTPRT	1	1070	0.411623741	0.716880663
PTPRC	5	1066	0.416673127	0.716880663
ARHGAP26	1	1070	0.411623741	0.716880663
ARHGEF10L	1	1070	0.385922639	0.716880663
RPL5	1	1070	0.438674524	0.716880663
RBM10	1	1070	0.391950155	0.716880663
RSPO2	1	1070	0.424618121	0.716880663
SETD1B	6	1065	0.400249315	0.716880663
SETD2	2	1069	0.122415058	0.716880663
SMAD2	2	1069	0.234907297	0.716880663
SFPQ	1	1070	0.407619244	0.716880663
SDHC	1	1070	0.41162043	0.716880663

SMARCA4	1	1070	0.407619244	0.716880663
SMARCE1	1	1070	0.417847639	0.716880663
TRIM33	1	1070	0.337248505	0.716880663
TPM3	2	1069	0.261330953	0.716880663
WRN	1	1070	0.411623741	0.716880663
WNK2	1	1070	0.385922639	0.716880663
ZNRF3	1	1070	0.170285157	0.716880663
ZFHX3	6	1065	0.309756868	0.716880663
ZMYM3	1	1070	0.439748565	0.716880663
ZNF331	1	1070	0.391950155	0.716880663
ARNT	2	1069	0.225347311	0.716880663
ATP1A1	1	1070	0.206813106	0.716880663
BCL11B	1	1070	0.187129562	0.716880663
BCLAF1	4	1067	0.325737322	0.716880663
BCORL1	1	1070	0.373078968	0.716880663
CIC	2	1069	0.270694386	0.716880663
CTNND1	1	1070	0.206813106	0.716880663
CD209	1	1070	0.407619244	0.716880663
DCC	1	1070	0.41162043	0.716880663
DCAF12L2	1	1070	0.413219358	0.716880663
ELF4	2	1069	0.287607934	0.716880663
EPHA3	3	1068	0.412939603	0.716880663
ERBB4	4	1067	0.240662693	0.716880663
FAM47C	1	1070	0.41162043	0.716880663
FAT3	10	1061	0.415717229	0.716880663
FLNA	2	1069	0.117163425	0.716880663
GATA1	1	1070	0.417847639	0.716880663
GATA3	1	1070	0.440780193	0.716880663
GPC3	1	1070	0.422021426	0.716880663
IRF4	1	1070	0.385922639	0.716880663
MAP2K4	1	1070	0.395300839	0.716880663
MAP3K1	1	1070	0.41162043	0.716880663
NTRK1	1	1070	0.407619244	0.716880663
PMS1	3	1068	0.30962529	0.716880663
PRPF40B	1	1070	0.430792583	0.716880663
RAD21	1	1070	0.445739552	0.716880663
RGPD3	1	1070	0.391950155	0.716880663
RUNX1T1	1	1070	0.215785307	0.716880663
TERT	1	1070	0.206813106	0.716880663
TET1	2	1069	0.252666494	0.716880663
TBL1XR1	1	1070	0.407619244	0.716880663
TP53	85	986	0.335945862	0.716880663
ZNF429	6	1065	0.292754099	0.716880663

GOLGA5	1	1070	0.453302589	0.721576616
MYH11	4	1067	0.453282965	0.721576616
OMD	1	1070	0.458875868	0.721576616
SEPT9	2	1069	0.468435221	0.721576616
PDCD1LG2	1	1070	0.473470043	0.721576616
FANCE	1	1070	0.462165043	0.721576616
KDM5C	1	1070	0.472569172	0.721576616
APOBEC3B	1	1070	0.456719497	0.721576616
BIRC3	1	1070	0.473470043	0.721576616
BMP5	1	1070	0.463485221	0.721576616
DAXX	1	1070	0.468047515	0.721576616
ITGAV	2	1069	0.473470043	0.721576616
CLIP1	4	1067	0.488561066	0.723175563
GMPS	5	1066	0.480321766	0.723175563
PSIP1	1	1070	0.489725063	0.723175563
RSPO3	3	1068	0.491459658	0.723175563
KRAS	3	1068	0.482481799	0.723175563
ELF3	1	1070	0.483425555	0.723175563
PTCH1	3	1068	0.492318144	0.723175563
PTEN	3	1068	0.493168436	0.723175563
SMARCB1	1	1070	0.483425555	0.723175563
SPEN	4	1067	0.498132375	0.727398322
PTPRB	2	1069	0.506850867	0.737045636
CHD2	7	1064	0.516907761	0.748551073
ACSL3	1	1070	0.521696503	0.752363965
KMT2A	3	1068	0.524796077	0.753719468
KMT2D	5	1066	0.530383326	0.758622052
DDX10	1	1070	0.532846927	0.75903501
HSP90AB1	1	1070	0.542212034	0.759546131
ALK	3	1068	0.543096937	0.759546131
FCGR2B	2	1069	0.537028372	0.759546131
MITF	1	1070	0.544087487	0.759546131
KDM6A	6	1065	0.538338544	0.759546131
PBRM1	7	1064	0.555616421	0.769484647
RHOA	1	1070	0.553530825	0.769484647
ATRX	6	1065	0.559550224	0.771869677
ERCC3	2	1069	0.567847887	0.78023194
LATS1	2	1069	0.570986099	0.781467249
PAX7	1	1070	0.577668975	0.787130014
NBEA	6	1065	0.579634423	0.787130014
EIF4A2	3	1068	0.597425559	0.787562004
CXCR4	2	1069	0.594507495	0.787562004
SET	1	1070	0.598005533	0.787562004

SKI	1	1070	0.594548253	0.787562004
TFE3	1	1070	0.586514313	0.787562004
UBR5	1	1070	0.587849196	0.787562004
PATZ1	1	1070	0.594507495	0.787562004
CBLC	2	1069	0.594507495	0.787562004
FOXA1	1	1070	0.608697349	0.791046496
MN1	1	1070	0.609717786	0.791046496
SF3B1	1	1070	0.609717786	0.791046496
ATM	5	1066	0.606716921	0.791046496
AXIN2	1	1070	0.617980156	0.798796573
KMT2C	13	1058	0.627879332	0.808597369
CCNE1	1	1070	0.639282373	0.814268424
STAT6	1	1070	0.639282373	0.814268424
EPAS1	1	1070	0.639282373	0.814268424
BLM	3	1068	0.642120831	0.814909709
S100A7	1	1070	0.648840211	0.819373772
LPP	3	1068	0.65033391	0.819373772
AFF3	3	1068	0.655467236	0.822870738
MLLT6	3	1068	0.671225197	0.833705212
CHD4	3	1068	0.675369357	0.833705212
PDGFRA	3	1068	0.674658702	0.833705212
STIL	3	1068	0.680819443	0.833705212
PTPRD	2	1069	0.675405523	0.833705212
RB1	6	1065	0.678631425	0.833705212
NOTCH2	4	1067	0.680309882	0.833705212
CNBD1	4	1067	0.689288159	0.841124361
ERBB3	2	1069	0.704830696	0.851384145
FBXW7	7	1064	0.705014378	0.851384145
SFRP4	2	1069	0.703787627	0.851384145
SMC1A	1	1070	0.717767714	0.863796317
MALT1	1	1070	0.725272733	0.866611465
EPS15	2	1069	0.723110929	0.866611465
TGFBR2	4	1067	0.72755633	0.866611465
ATR	5	1066	0.730820878	0.86753907
ROBO2	2	1069	0.739050282	0.874334062
ZNF479	4	1067	0.744218057	0.877473317
TSC1	4	1067	0.757659965	0.889677071
TRIM24	2	1069	0.759666955	0.889677071
ZNF521	8	1063	0.764891981	0.89280034
KTN1	7	1064	0.772388657	0.896847739
FAM135B	10	1061	0.773499053	0.896847739
CDK12	3	1068	0.777530277	0.897943917
TET2	4	1067	0.779590277	0.897943917

PLCG1	2	1069	0.783230791	0.899169559
NTRK3	3	1068	0.803027898	0.915871688
EPHA7	2	1069	0.801147632	0.915871688
CARD11	2	1069	0.811021461	0.91799129
IL7R	2	1069	0.815228737	0.91799129
ROS1	3	1068	0.813867564	0.91799129
KEAP1	2	1069	0.815407737	0.91799129
AFF4	2	1069	0.821761104	0.920259195
CSMD3	27	1044	0.822695899	0.920259195
NOTCH1	21	1050	0.82742475	0.922591814
FAM131B	2	1069	0.83744765	0.924902626
DROSHA	4	1067	0.835756132	0.924902626
RGS7	2	1069	0.834330541	0.924902626
BRIP1	3	1068	0.861443678	0.948403292
ATIC	2	1069	0.900411337	0.963881482
KIAA1549	3	1068	0.89808997	0.963881482
CTNNA2	8	1063	0.905210112	0.963881482
CTCF	2	1069	0.888618238	0.963881482
CHEK2	6	1065	0.906559636	0.963881482
CCNC	2	1069	0.903876302	0.963881482
NCOR1	2	1069	0.899967359	0.963881482
PRDM2	2	1069	0.903424176	0.963881482
PTPN13	2	1069	0.889158699	0.963881482
PTPRK	2	1069	0.890350205	0.963881482
SBDS	2	1069	0.899297546	0.963881482
ANK1	3	1068	0.908644721	0.963881482
TPR	2	1069	0.918485505	0.965516389
ARHGAP5	4	1067	0.917383676	0.965516389
SPOP	2	1069	0.917353232	0.965516389
GRM3	5	1066	0.927929556	0.972514759
BAZ1A	4	1067	0.940056462	0.982274567
EML4	2	1069	0.950001998	0.986758028
GPHN	2	1069	0.948576648	0.986758028
FGFR1OP	2	1069	0.976990357	0.989285997
RALGDS	2	1069	0.981776591	0.989285997
SS18	2	1069	0.967245223	0.989285997
DDX5	2	1069	0.963440981	0.989285997
KDM5A	2	1069	0.981817741	0.989285997
TRRAP	4	1067	0.973746023	0.989285997
LZTR1	4	1067	0.97145255	0.989285997
PPP2R1A	2	1069	0.972200649	0.989285997
RAD17	2	1069	0.983616736	0.989285997
CREBBP	5	1066	0.973968902	0.989285997

PABPC1	6	1065	0.9714217	0.989285997
FUBP1	4	1067	0.999280648	0.999672597
GRIN2A	2	1069	0.999672597	0.999672597

Table S6k. Association between CCA of genes undering SBS22* signature and prognosis.

GeneName	CCA>=0.06	CCA<0.06	pvalue	qvalue(fdr)
NOTCH1	12	1059	0.004194428	0.057556878
CCND1	1	1070	1.29E-14	1.06E-12
PTPN13	1	1070	1.29E-14	1.06E-12
PTPRT	1	1070	1.29E-14	1.06E-12
LCK	1	1070	2.07E-09	1.28E-07
MALT1	1	1070	1.13E-07	5.56E-06
SFRP4	2	1069	8.13E-05	0.003345444
COL3A1	3	1068	0.000259167	0.005492051
FNBP1	1	1070	0.000333525	0.005492051
KAT6A	3	1068	0.000178365	0.005492051
MTOR	2	1069	0.000302308	0.005492051
PPM1D	1	1070	0.000333525	0.005492051
RARA	1	1070	0.000333525	0.005492051
KRAS	1	1070	0.000333525	0.005492051
ABI1	1	1070	0.000333525	0.005492051
KAT6B	3	1068	0.00021212	0.005492051
TCF12	1	1070	0.000632926	0.0097708
IGF2BP2	1	1070	0.00331936	0.048228352
CTNND2	1	1070	0.009835352	0.121466601
EWSR1	1	1070	0.009835352	0.121466601
TFG	1	1070	0.010869078	0.122030099
POLQ	4	1067	0.010563593	0.122030099
CUL3	2	1069	0.013628562	0.146358905
AR	3	1068	0.015475297	0.1592666
VAV1	1	1070	0.021319051	0.181579506
MN1	1	1070	0.021319051	0.181579506
MYC	1	1070	0.021319051	0.181579506
NBN	1	1070	0.021319051	0.181579506
SPOP	1	1070	0.021319051	0.181579506
EP300	10	1061	0.024574495	0.20233001
CHD4	3	1068	0.036870154	0.260197942
RET	1	1070	0.035853933	0.260197942
FANCC	1	1070	0.035853933	0.260197942
ARHGEF12	1	1070	0.035853933	0.260197942
DCC	1	1070	0.035853933	0.260197942
WIF1	2	1069	0.039885148	0.273656435
COX6C	1	1070	0.055627619	0.361579522

FGFR2	1	1070	0.055627619	0.361579522
AXIN1	1	1070	0.058281634	0.369117014
SF3B1	2	1069	0.061369895	0.378959102
CASP8	2	1069	0.078091237	0.438375809
IKZF1	1	1070	0.07656776	0.438375809
NCOR1	1	1070	0.07656776	0.438375809
NFE2L2	8	1063	0.074628567	0.438375809
H3F3B	1	1070	0.093904564	0.493498455
SIX2	1	1070	0.093904564	0.493498455
ELF3	2	1069	0.091062948	0.493498455
RGS7	2	1069	0.098155691	0.505092827
AKAP9	1	1070	0.125277811	0.606737636
ROBO2	1	1070	0.125277811	0.606737636
ATP1A1	1	1070	0.125277811	0.606737636
ZNF429	7	1064	0.128810824	0.611851412
AFF1	1	1070	0.417847639	0.651392756
CLIP1	1	1070	0.391950155	0.651392756
ERC1	1	1070	0.411623741	0.651392756
GOLGA5	1	1070	0.453302589	0.651392756
HERPUD1	1	1070	0.440780193	0.651392756
HOOK3	1	1070	0.395300839	0.651392756
JAZF1	1	1070	0.385922639	0.651392756
KTN1	1	1070	0.440780193	0.651392756
LMNA	1	1070	0.146311412	0.651392756
LIFR	1	1070	0.411623741	0.651392756
MLLT3	1	1070	0.411623741	0.651392756
NCOA1	1	1070	0.373078968	0.651392756
OMD	1	1070	0.458875868	0.651392756
PDE4DIP	3	1068	0.287353908	0.651392756
RALGDS	1	1070	0.391950155	0.651392756
RNF213	1	1070	0.187129562	0.651392756
THRAP3	1	1070	0.419287106	0.651392756
TPR	3	1068	0.202266816	0.651392756
TMPRSS2	1	1070	0.378058023	0.651392756
AFF3	1	1070	0.395300839	0.651392756
BIRC6	3	1068	0.19667146	0.651392756
BCL2L12	1	1070	0.411623741	0.651392756
ABL2	3	1068	0.177507079	0.651392756
CTNNB1	1	1070	0.402894233	0.651392756
CTNNA2	1	1070	0.170285157	0.651392756
CCR4	1	1070	0.385922639	0.651392756
CD79A	1	1070	0.414260415	0.651392756
CSF1R	1	1070	0.437589284	0.651392756

DDX5	1	1070	0.295163638	0.651392756
XPO1	1	1070	0.391950155	0.651392756
FCGR2B	1	1070	0.314749166	0.651392756
FLT3	3	1068	0.214909726	0.651392756
FSTL3	1	1070	0.417847639	0.651392756
GLI1	1	1070	0.263918441	0.651392756
GNAQ	1	1070	0.417847639	0.651392756
HLF	1	1070	0.417847639	0.651392756
IL7R	1	1070	0.391950155	0.651392756
IDH1	1	1070	0.395300839	0.651392756
JAK2	1	1070	0.422021426	0.651392756
KDM5A	1	1070	0.411623741	0.651392756
KMT2A	2	1069	0.246377937	0.651392756
MAML2	1	1070	0.453302589	0.651392756
MDM2	1	1070	0.402894233	0.651392756
MET	2	1069	0.254478742	0.651392756
MUC16	18	1053	0.405313087	0.651392756
MLLT10	1	1070	0.424618121	0.651392756
NTRK3	2	1069	0.264806643	0.651392756
NFATC2	1	1070	0.424618121	0.651392756
NUP98	1	1070	0.437589284	0.651392756
PREX2	1	1070	0.195844402	0.651392756
PIK3CA	5	1066	0.197396633	0.651392756
PLAG1	1	1070	0.427152138	0.651392756
ARHGAP5	1	1070	0.391950155	0.651392756
SGK1	1	1070	0.411623741	0.651392756
SETBP1	1	1070	0.437589284	0.651392756
TAF15	2	1069	0.267796034	0.651392756
AKT3	1	1070	0.385922639	0.651392756
ERBB2	1	1070	0.445739552	0.651392756
KIT	1	1070	0.453302589	0.651392756
REL	2	1069	0.263918441	0.651392756
ROS1	1	1070	0.411623741	0.651392756
WWTR1	1	1070	0.373078968	0.651392756
ASXL1	1	1070	0.427152138	0.651392756
ASXL2	1	1070	0.427152138	0.651392756
APC	3	1068	0.27156427	0.651392756
ARID1A	1	1070	0.411623741	0.651392756
ARID1B	1	1070	0.456719497	0.651392756
ATM	3	1068	0.448946522	0.651392756
AXIN2	1	1070	0.391950155	0.651392756
BAP1	2	1069	0.411623741	0.651392756
BARD1	4	1067	0.164407721	0.651392756

BAZ1A	2	1069	0.362039524	0.651392756
CIITA	1	1070	0.411623741	0.651392756
CCDC6	1	1070	0.268705878	0.651392756
CNTNAP2	1	1070	0.458875868	0.651392756
DICER1	1	1070	0.391950155	0.651392756
DNMT3A	1	1070	0.411623741	0.651392756
POLG	1	1070	0.370271862	0.651392756
EBF1	1	1070	0.411623741	0.651392756
FANCA	2	1069	0.234900111	0.651392756
FAT1	8	1063	0.449280342	0.651392756
FBLN2	1	1070	0.195844402	0.651392756
FH	1	1070	0.422021426	0.651392756
GRIN2A	3	1068	0.395584105	0.651392756
ID3	2	1069	0.27156427	0.651392756
LARP4B	1	1070	0.375455671	0.651392756
LZTR1	1	1070	0.453302589	0.651392756
KMT2C	4	1067	0.411468507	0.651392756
MYH9	1	1070	0.439748565	0.651392756
N4BP2	3	1068	0.410112764	0.651392756
NRG1	2	1069	0.235141718	0.651392756
PHOX2B	1	1070	0.391950155	0.651392756
PBRM1	1	1070	0.292702882	0.651392756
PTPRD	5	1066	0.175435477	0.651392756
PTPRC	1	1070	0.453302589	0.651392756
ARHGAP26	1	1070	0.395300839	0.651392756
ARHGEF10L	1	1070	0.385922639	0.651392756
SLC34A2	1	1070	0.427152138	0.651392756
SPEN	2	1069	0.263574998	0.651392756
SMARCA4	1	1070	0.445739552	0.651392756
TET2	2	1069	0.150640218	0.651392756
TGFBR2	2	1069	0.235141718	0.651392756
TRIM33	1	1070	0.439748565	0.651392756
TNFRSF14	1	1070	0.395300839	0.651392756
WRN	1	1070	0.391950155	0.651392756
ZFHX3	1	1070	0.385922639	0.651392756
CBLC	1	1070	0.395300839	0.651392756
CTNND1	1	1070	0.206813106	0.651392756
CREBBP	2	1069	0.411926957	0.651392756
CRNKL1	1	1070	0.370271862	0.651392756
EPAS1	2	1069	0.171853672	0.651392756
EPHA7	1	1070	0.437589284	0.651392756
ERBB4	2	1069	0.373078968	0.651392756
FAT3	6	1065	0.34615679	0.651392756

GATA3	1	1070	0.440780193	0.651392756
MB21D2	1	1070	0.370271862	0.651392756
MAP3K13	1	1070	0.445739552	0.651392756
NTRK1	1	1070	0.395300839	0.651392756
NOTCH2	8	1063	0.39871481	0.651392756
PRKCB	1	1070	0.41162043	0.651392756
RAD21	1	1070	0.445739552	0.651392756
TBL1XR1	1	1070	0.407619244	0.651392756
RAD51B	1	1070	0.468047515	0.656862137
STAG1	1	1070	0.468047515	0.656862137
PALB2	3	1068	0.471241756	0.657608552
PPP2R1A	3	1068	0.4883049	0.675373295
RB1	7	1064	0.492174871	0.675373295
FLNA	2	1069	0.490059446	0.675373295
CSMD3	18	1053	0.496442484	0.67746571
ITGAV	1	1070	0.503021437	0.681868113
PABPC1	3	1068	0.505189736	0.681868113
ATRX	2	1069	0.527072148	0.707537069
NUMA1	1	1070	0.534248166	0.713293497
CDK12	1	1070	0.544087487	0.722524781
FBXW7	4	1067	0.548539262	0.724541164
PCM1	3	1068	0.553002739	0.72655147
CAMTA1	3	1068	0.55643949	0.727198699
NCOA2	3	1068	0.577948919	0.751333595
PPFIBP1	1	1070	0.594507495	0.760846379
CBLB	1	1070	0.594507495	0.760846379
ZNF479	4	1067	0.591427339	0.760846379
FGFR1OP	1	1070	0.609717786	0.774827912
VTI1A	1	1070	0.617980156	0.774827912
FANCD2	1	1070	0.617980156	0.774827912
FES	1	1070	0.612147297	0.774827912
CHST11	1	1070	0.631164581	0.783405284
FLCN	1	1070	0.631164581	0.783405284
FAM131B	1	1070	0.639282373	0.785585802
ZNF521	1	1070	0.639282373	0.785585802
PLCG1	1	1070	0.647750187	0.788149242
MAX	1	1070	0.647750187	0.788149242
CHEK2	3	1068	0.652794492	0.790393331
BRD4	1	1070	0.656479784	0.790978082
MECOM	1	1070	0.694233317	0.825152878
GPC5	2	1069	0.697176199	0.825152878
RANBP2	3	1068	0.693062213	0.825152878
ZNF331	2	1069	0.698206282	0.825152878

ESR1	1	1070	0.701909	0.825578681
SEPT9	1	1070	0.725272733	0.849015948
BCL11A	1	1070	0.755222452	0.852413706
NF1	2	1069	0.748556427	0.852413706
PTPRK	2	1069	0.755724474	0.852413706
SETD1B	2	1069	0.757866147	0.852413706
SDHA	1	1070	0.734171059	0.852413706
FAM135B	7	1064	0.75923488	0.852413706
NBEA	6	1065	0.751645702	0.852413706
RGPD3	3	1068	0.744016974	0.852413706
TET1	2	1069	0.748556427	0.852413706
PTPRB	3	1068	0.769602415	0.860143875
TRRAP	2	1069	0.782140719	0.870219629
PRDM2	2	1069	0.797164002	0.882957437
BRAF	2	1069	0.807099081	0.889970862
CDH10	3	1068	0.813928257	0.893512353
PIK3CB	2	1069	0.829400088	0.900607976
TSHR	2	1069	0.831330439	0.900607976
ATR	4	1067	0.826483605	0.900607976
BCORL1	2	1069	0.838358992	0.904256205
ZEB1	2	1069	0.848587283	0.911308952
UBR5	2	1069	0.866364606	0.925607302
CDKN2A	2	1069	0.869396332	0.925607302
TP53	89	982	0.91923338	0.974466287
FAM47C	2	1069	0.928103425	0.979664727
KEAP1	2	1069	0.932325367	0.979933471
PAX7	2	1069	0.941141511	0.980314484
LRP1B	10	1061	0.944594524	0.980314484
SUFU	2	1069	0.936823228	0.980314484
SS18	2	1069	0.967245223	0.990190667
CARD11	2	1069	0.989566269	0.990190667
ETV1	2	1069	0.978333791	0.990190667
STAT6	2	1069	0.987181929	0.990190667
BRIP1	3	1068	0.96983202	0.990190667
FAT4	4	1067	0.98048076	0.990190667
PTCH1	2	1069	0.964826224	0.990190667
CNBD1	5	1066	0.990190667	0.990190667
KMT2D	8	1063	0.974507545	0.990190667

Table S6l. Association between CCA of genes undering SBS15* signature and prognosis.

GeneName	CCA>=0.06	CCA<0.06	pvalue	qvalue(fdr)
**PRDM1**	**5**	**1066**	**0.000159079**	**0.005567771**
ABL2	2	1069	3.55E-15	1.26E-12

MAP2K2	1	1070	1.29E-14	1.26E-12
PRKACA	1	1070	1.29E-14	1.26E-12
AKT2	1	1070	1.29E-14	1.26E-12
SDHC	1	1070	1.29E-14	1.26E-12
ACSL3	4	1067	3.70E-10	3.02E-08
SH3GL1	1	1070	1.13E-07	6.13E-06
CHD2	2	1069	1.13E-07	6.13E-06
FOXO1	1	1070	1.13E-07	6.13E-06
TNFRSF14	1	1070	3.07E-07	1.51E-05
TFEB	2	1069	1.79E-06	7.98E-05
TAL1	2	1069	1.93E-05	0.000789588
MLLT10	2	1069	6.05E-05	0.002280069
DCTN1	3	1068	0.000276383	0.00742852
FNBP1	1	1070	0.000333525	0.00742852
SNX29	1	1070	0.000333525	0.00742852
FGFR3	2	1069	0.000259167	0.00742852
IL7R	1	1070	0.000333525	0.00742852
TLX3	1	1070	0.000333525	0.00742852
CASP8	2	1069	0.000259167	0.00742852
STK11	1	1070	0.000333525	0.00742852
PMS2	1	1070	0.000495919	0.010565224
FAM131B	2	1069	0.001115801	0.022780941
NUTM2B	1	1070	0.002636768	0.046143442
IDH2	1	1070	0.002636768	0.046143442
MEN1	1	1070	0.002636768	0.046143442
PPARG	2	1069	0.002578419	0.046143442
NCOR2	2	1069	0.002981625	0.050379179
ACSL6	4	1067	0.003603726	0.058860863
ETV5	3	1068	0.005385853	0.082470876
HNF1A	3	1068	0.00534152	0.082470876
JAZF1	1	1070	0.009835352	0.130251963
CCR4	1	1070	0.009835352	0.130251963
MPL	1	1070	0.009835352	0.130251963
OLIG2	2	1069	0.009413082	0.130251963
ECT2L	1	1070	0.009835352	0.130251963
TAF15	1	1070	0.010869078	0.140153896
RHOA	2	1069	0.01200519	0.150834443
PTK6	2	1069	0.013710533	0.167954025
MYC	1	1070	0.021319051	0.242938027
FEN1	1	1070	0.021319051	0.242938027
NBN	1	1070	0.021319051	0.242938027
FANCD2	5	1066	0.022504448	0.250617716
SRC	2	1069	0.023439912	0.255234602

ERBB3	2	1069	0.02531998	0.269712827
CARD11	3	1068	0.027721248	0.289008755
DNAJB1	2	1069	0.029253345	0.298627899
CDH1	2	1069	0.030052525	0.300525253
BIRC6	4	1067	0.032838588	0.321818167
EML4	1	1070	0.035853933	0.322017987
ROS1	1	1070	0.035853933	0.322017987
CBFA2T3	2	1069	0.036144876	0.322017987
CCNC	1	1070	0.035853933	0.322017987
DDB2	1	1070	0.035853933	0.322017987
COL1A1	3	1068	0.03694702	0.323286424
IGF2BP2	3	1068	0.047410439	0.407563423
ARHGEF10	3	1068	0.051045482	0.431246315
CCDC6	1	1070	0.055627619	0.446844807
CYP2C8	1	1070	0.055627619	0.446844807
STAT5B	1	1070	0.055627619	0.446844807
BARD1	2	1069	0.05694745	0.448849372
NFE2L2	20	1051	0.057709205	0.448849372
MAML2	5	1066	0.062789908	0.47621268
RANBP2	5	1066	0.06317107	0.47621268
FLT3	2	1069	0.065236638	0.484332617
BCR	5	1066	0.07615553	0.521086146
MNX1	1	1070	0.07656776	0.521086146
GNA11	1	1070	0.07656776	0.521086146
MAP2K1	1	1070	0.07656776	0.521086146
CIITA	6	1065	0.074614543	0.521086146
MSH2	2	1069	0.072415613	0.521086146
NIN	3	1068	0.078673382	0.528081604
FOXP1	2	1069	0.080506356	0.533082627
NUMA1	5	1066	0.082651268	0.539988283
PAX3	2	1069	0.110687206	0.670494163
PDGFRB	4	1067	0.109337223	0.670494163
EPS15	1	1070	0.105818107	0.670494163
PTCH1	8	1063	0.11148604	0.670494163
PRDM2	5	1066	0.112205146	0.670494163
BCLAF1	15	1056	0.109696178	0.670494163
RAD21	2	1069	0.104157968	0.670494163
RET	3	1068	0.113905871	0.672456348
SS18	1	1070	0.125277811	0.730787233
ATIC	1	1070	0.337248505	0.739665392
AFF1	3	1068	0.408981776	0.739665392
ALDH2	1	1070	0.337248505	0.739665392
ASPSCR1	1	1070	0.370271862	0.739665392

COL3A1	12	1059	0.403764517	0.739665392
ELN	1	1070	0.395300839	0.739665392
EIF4A2	2	1069	0.249264305	0.739665392
FGFR1OP	1	1070	0.140401482	0.739665392
GOLGA5	3	1068	0.168220415	0.739665392
GAS7	2	1069	0.254352404	0.739665392
GMPS	3	1068	0.147927546	0.739665392
HOOK3	1	1070	0.395300839	0.739665392
ITK	1	1070	0.391950155	0.739665392
IL2	1	1070	0.140401482	0.739665392
LMNA	2	1069	0.299920249	0.739665392
LCP1	2	1069	0.389360932	0.739665392
MLLT3	1	1070	0.339789994	0.739665392
NFIB	2	1069	0.239884432	0.739665392
NCOA1	2	1069	0.225347311	0.739665392
NUP214	3	1068	0.153621397	0.739665392
PRCC	1	1070	0.402894233	0.739665392
RABEP1	2	1069	0.244358421	0.739665392
RNF213	8	1063	0.255348392	0.739665392
SEPT5	1	1070	0.439748565	0.739665392
SRGAP3	1	1070	0.140401482	0.739665392
SLC45A3	3	1068	0.157852818	0.739665392
SPECC1	3	1068	0.282577042	0.739665392
TOP1	1	1070	0.416309934	0.739665392
TPR	10	1061	0.196815492	0.739665392
TPM4	1	1070	0.395300839	0.739665392
VAV1	2	1069	0.395976269	0.739665392
ZMYM2	2	1069	0.27156427	0.739665392
NT5C2	3	1068	0.238161697	0.739665392
ACVR1	1	1070	0.411623741	0.739665392
ACKR3	1	1070	0.395300839	0.739665392
BCL11A	5	1066	0.181743725	0.739665392
BCL2L12	1	1070	0.407619244	0.739665392
CACNA1D	4	1067	0.353414488	0.739665392
CREB3L2	1	1070	0.440780193	0.739665392
CTNNA2	9	1062	0.269669719	0.739665392
CSF1R	1	1070	0.314749166	0.739665392
CSF3R	1	1070	0.402894233	0.739665392
DEK	1	1070	0.395300839	0.739665392
DGCR8	2	1069	0.25040706	0.739665392
DDR2	4	1067	0.189057045	0.739665392
ELK4	1	1070	0.433111469	0.739665392
EGFR	3	1068	0.158301747	0.739665392

FCRL4	1	1070	0.170285157	0.739665392
FGFR1	1	1070	0.419287106	0.739665392
FGFR2	1	1070	0.391950155	0.739665392
FGFR4	3	1068	0.16118189	0.739665392
FOXA1	2	1069	0.27156427	0.739665392
FLI1	1	1070	0.385922639	0.739665392
GATA2	1	1070	0.430792583	0.739665392
GRM3	11	1060	0.143604947	0.739665392
GNAS	10	1061	0.25879928	0.739665392
GNAQ	1	1070	0.430792583	0.739665392
H3F3A	1	1070	0.373078968	0.739665392
HNRNPA2B1	2	1069	0.273764179	0.739665392
HOXC13	1	1070	0.411623741	0.739665392
HOXD13	1	1070	0.385922639	0.739665392
HIF1A	1	1070	0.370271862	0.739665392
IL6ST	1	1070	0.419287106	0.739665392
JUN	1	1070	0.440780193	0.739665392
LCK	1	1070	0.411623741	0.739665392
KDM5A	3	1068	0.230370201	0.739665392
KMT2A	8	1063	0.171219254	0.739665392
MDM2	1	1070	0.402894233	0.739665392
MITF	2	1069	0.417847639	0.739665392
MN1	5	1066	0.287590364	0.739665392
MUC16	62	1009	0.32928365	0.739665392
MUC4	3	1068	0.243853859	0.739665392
NCOA2	7	1064	0.336209112	0.739665392
NPM1	2	1069	0.417847639	0.739665392
PREX2	6	1065	0.147855835	0.739665392
PLCG1	4	1067	0.16706924	0.739665392
PLAG1	3	1068	0.385922639	0.739665392
POU2AF1	2	1069	0.427152138	0.739665392
PPM1D	1	1070	0.375455671	0.739665392
PTPN11	1	1070	0.427152138	0.739665392
RAC1	1	1070	0.417847639	0.739665392
RARA	1	1070	0.402894233	0.739665392
RSPO3	1	1070	0.395300839	0.739665392
SRSF3	1	1070	0.206813106	0.739665392
SGK1	1	1070	0.411623741	0.739665392
SETBP1	6	1065	0.223657067	0.739665392
SKI	2	1069	0.249850969	0.739665392
SF3B1	3	1068	0.299531954	0.739665392
TCL1A	1	1070	0.337248505	0.739665392
TEC	1	1070	0.391950155	0.739665392

TNC	2	1069	0.419287106	0.739665392
TSHR	1	1070	0.314749166	0.739665392
TCF7L2	2	1069	0.231603482	0.739665392
TRRAP	7	1064	0.183693859	0.739665392
TNFRSF17	1	1070	0.337248505	0.739665392
USP8	2	1069	0.237468848	0.739665392
ABL1	2	1069	0.275425765	0.739665392
AKT3	1	1070	0.417847639	0.739665392
KDR	7	1064	0.14267076	0.739665392
ERG	1	1070	0.419287106	0.739665392
KIT	3	1068	0.417241756	0.739665392
MAF	1	1070	0.413219358	0.739665392
WAS	1	1070	0.385922639	0.739665392
WWTR1	1	1070	0.440780193	0.739665392
ACVR2A	1	1070	0.413219358	0.739665392
ASXL1	1	1070	0.427152138	0.739665392
ASXL2	3	1068	0.26751754	0.739665392
ATM	4	1067	0.346270437	0.739665392
ATP2B3	4	1067	0.173221414	0.739665392
ATR	8	1063	0.24189756	0.739665392
AXIN2	4	1067	0.234547484	0.739665392
BAX	1	1070	0.385922639	0.739665392
BCOR	1	1070	0.414260415	0.739665392
BUB1B	1	1070	0.407619244	0.739665392
CAMTA1	9	1062	0.300225121	0.739665392
CBLB	2	1069	0.227147276	0.739665392
CTCF	3	1068	0.183390036	0.739665392
CD274	1	1070	0.395300839	0.739665392
CDC73	1	1070	0.231603482	0.739665392
CLTCL1	2	1069	0.243132888	0.739665392
CSMD3	39	1032	0.39424095	0.739665392
CDKN2A	18	1053	0.169320359	0.739665392
CARS	1	1070	0.419287106	0.739665392
CPEB3	2	1069	0.419287106	0.739665392
DDX10	3	1068	0.205196834	0.739665392
DDX3X	1	1070	0.290250051	0.739665392
DNMT3A	4	1067	0.166529103	0.739665392
EBF1	7	1064	0.281115247	0.739665392
ETV6	1	1070	0.440780193	0.739665392
ERCC2	2	1069	0.246770885	0.739665392
ERCC5	5	1066	0.3260679	0.739665392
BRCA2	5	1066	0.149553368	0.739665392
CYLD	4	1067	0.416148546	0.739665392

FANCC	2	1069	0.292723845	0.739665392
FAS	1	1070	0.385922639	0.739665392
FAT1	28	1043	0.298422074	0.739665392
FBXW7	15	1056	0.291117132	0.739665392
FBLN2	4	1067	0.150625367	0.739665392
FLCN	1	1070	0.411623741	0.739665392
FUS	1	1070	0.213533295	0.739665392
GPC5	3	1068	0.190925302	0.739665392
IKZF1	7	1064	0.276806656	0.739665392
LATS1	2	1069	0.389838572	0.739665392
LATS2	2	1069	0.260246365	0.739665392
LRIG3	2	1069	0.257043568	0.739665392
EXT1	2	1069	0.258816028	0.739665392
MAX	2	1069	0.264110947	0.739665392
MYH9	5	1066	0.292457347	0.739665392
MGMT	2	1069	0.259988302	0.739665392
PHOX2B	2	1069	0.413219358	0.739665392
PRF1	4	1067	0.237339846	0.739665392
PER1	3	1068	0.163047372	0.739665392
PPP2R1A	3	1068	0.276007059	0.739665392
PTPRD	14	1057	0.42761373	0.739665392
PTPRT	7	1064	0.277410423	0.739665392
PTPRC	7	1064	0.131195653	0.739665392
PTPRK	5	1066	0.412972318	0.739665392
ARHGEF12	4	1067	0.294997259	0.739665392
ARHGEF10L	1	1070	0.391950155	0.739665392
RPL5	2	1069	0.244675584	0.739665392
RFWD3	1	1070	0.427152138	0.739665392
RNF43	2	1069	0.203895306	0.739665392
ROBO2	2	1069	0.346377166	0.739665392
SFRP4	5	1066	0.136300968	0.739665392
SETD1B	4	1067	0.249018865	0.739665392
SETD2	3	1068	0.261426668	0.739665392
SBDS	1	1070	0.417847639	0.739665392
SMAD3	1	1070	0.385922639	0.739665392
SMAD4	5	1066	0.319433235	0.739665392
SPOP	3	1068	0.345560719	0.739665392
STAG1	1	1070	0.413219358	0.739665392
SMARCA4	3	1068	0.179523887	0.739665392
SMARCD1	2	1069	0.249032412	0.739665392
TET2	4	1067	0.13789338	0.739665392
TRIM33	1	1070	0.439748565	0.739665392
TPM3	1	1070	0.395300839	0.739665392

TSC1	3	1068	0.216162919	0.739665392
TSC2	4	1067	0.149494809	0.739665392
USP44	3	1068	0.172736002	0.739665392
WNK2	6	1065	0.228991608	0.739665392
XPC	1	1070	0.437589284	0.739665392
ZNRF3	2	1069	0.385210042	0.739665392
ZBTB16	3	1068	0.256949345	0.739665392
APOBEC3B	1	1070	0.395300839	0.739665392
ARNT	1	1070	0.411623741	0.739665392
BIRC3	1	1070	0.440780193	0.739665392
BCORL1	2	1069	0.216676703	0.739665392
CBLC	1	1070	0.395300839	0.739665392
CTNND1	2	1069	0.27378048	0.739665392
CD209	1	1070	0.407619244	0.739665392
ELF4	2	1069	0.249314563	0.739665392
EPHA7	2	1069	0.223500184	0.739665392
ERBB4	10	1061	0.147600871	0.739665392
ESR1	1	1070	0.433111469	0.739665392
EIF1AX	1	1070	0.395300839	0.739665392
FAM135B	22	1049	0.368450053	0.739665392
FOXO3	1	1070	0.417847639	0.739665392
LEF1	1	1070	0.437589284	0.739665392
KMT2D	20	1051	0.286134592	0.739665392
MB21D2	4	1067	0.378681064	0.739665392
MAP3K13	3	1068	0.276154316	0.739665392
PMS1	1	1070	0.433111469	0.739665392
PCBP1	1	1070	0.437589284	0.739665392
RUNX1	1	1070	0.395300839	0.739665392
SUZ12	1	1070	0.378058023	0.739665392
TCF3	1	1070	0.41690198	0.739665392
TBL1XR1	2	1069	0.257043568	0.739665392
TRIM24	4	1067	0.301716012	0.739665392
MACC1	1	1070	0.443317461	0.740380951
ARID2	6	1065	0.445168652	0.740380951
CCNB1IP1	1	1070	0.445739552	0.740380951
TGFBR2	7	1064	0.447351023	0.740547301
LIFR	3	1068	0.453820551	0.741240233
MTCP1	1	1070	0.453302589	0.741240233
RAD51B	1	1070	0.453302589	0.741240233
ZNF479	1	1070	0.453302589	0.741240233
ARID1A	7	1064	0.457175304	0.744238868
LARP4B	1	1070	0.463485221	0.752012445
HRAS	1	1070	0.473470043	0.763158952

PML	1	1070	0.472569172	0.763158952
KDM5C	4	1067	0.477048043	0.766405053
JAK2	4	1067	0.484599397	0.774484057
EP300	17	1054	0.48997971	0.774484057
ABI1	1	1070	0.489725063	0.774484057
CIC	7	1064	0.489103131	0.774484057
TERT	6	1065	0.4856348	0.774484057
NF2	2	1069	0.491916838	0.775045822
S100A7	2	1069	0.49922056	0.777028617
NUTM1	1	1070	0.497463654	0.777028617
AKT1	1	1070	0.496937394	0.777028617
FAT4	21	1050	0.499518397	0.777028617
RPN1	1	1070	0.508651841	0.786244171
ZMYM3	1	1070	0.508651841	0.786244171
NCOA4	1	1070	0.511570598	0.786954453
FAM47C	6	1065	0.512323409	0.786954453
BRIP1	3	1068	0.517444004	0.792336131
KIAA1549	5	1066	0.519611128	0.793175865
ALK	5	1066	0.522877825	0.795683647
TMPRSS2	3	1068	0.526475012	0.796212209
TP63	6	1065	0.524993185	0.796212209
GRIN2A	12	1059	0.529688297	0.79860697
N4BP2	2	1069	0.533778708	0.80230542
PIK3CB	3	1068	0.547452455	0.815354721
CNTNAP2	14	1057	0.544677949	0.815354721
ZFHX3	11	1060	0.546314633	0.815354721
GPHN	2	1069	0.553530825	0.81716271
AR	3	1068	0.553669428	0.81716271
STAG2	1	1070	0.553530825	0.81716271
A1CF	4	1067	0.562575062	0.827813155
SEPT9	1	1070	0.572325018	0.834640651
CDK4	1	1070	0.572325018	0.834640651
RGS7	8	1063	0.569410325	0.834640651
MET	3	1068	0.581489013	0.840516921
RB1	12	1059	0.578890621	0.840516921
NOTCH2	12	1059	0.581500482	0.840516921
AFF3	8	1063	0.585613552	0.843972473
MECOM	5	1066	0.591250159	0.849597002
FCGR2B	3	1068	0.59449481	0.851628733
NBEA	9	1062	0.596140113	0.851628733
AKAP9	7	1064	0.623414077	0.865326969
CEP89	1	1070	0.631164581	0.865326969
MYH11	9	1062	0.637617683	0.865326969

PICALM	1	1070	0.617980156	0.865326969
PPFIBP1	1	1070	0.636889808	0.865326969
CHST11	2	1069	0.631164581	0.865326969
CD74	1	1070	0.617980156	0.865326969
MTOR	6	1065	0.625537619	0.865326969
PIM1	1	1070	0.631164581	0.865326969
STAT6	1	1070	0.639282373	0.865326969
APC	9	1062	0.621068314	0.865326969
BAZ1A	4	1067	0.608676304	0.865326969
CNOT3	1	1070	0.617980156	0.865326969
KAT6B	5	1066	0.633394607	0.865326969
LRP1B	26	1045	0.625205012	0.865326969
SUFU	3	1068	0.63923679	0.865326969
ZNF331	3	1068	0.628687315	0.865326969
ATP1A1	3	1068	0.615651094	0.865326969
KDM6A	9	1062	0.633673114	0.865326969
CYSLTR2	1	1070	0.646658489	0.870501812
POLQ	4	1067	0.645927961	0.870501812
SS18L1	1	1070	0.648840211	0.871045762
PAX7	3	1068	0.651608462	0.872371985
CDH11	8	1063	0.65679236	0.87691623
DCAF12L2	9	1062	0.664232181	0.884439589
TFE3	1	1070	0.666106344	0.88453146
NOTCH1	53	1018	0.668247664	0.884976636
ARID1B	5	1066	0.681139397	0.899618072
CLIP1	3	1068	0.7126379	0.90020336
CLP1	2	1069	0.728459017	0.90020336
RALGDS	3	1068	0.704875966	0.90020336
TRIP11	3	1068	0.717297026	0.90020336
BCL9	3	1068	0.728153397	0.90020336
CTNNB1	3	1068	0.696354815	0.90020336
CTNND2	10	1061	0.72767929	0.90020336
CCR7	1	1070	0.729348437	0.90020336
EWSR1	2	1069	0.689249407	0.90020336
KAT6A	7	1064	0.719046951	0.90020336
SND1	4	1067	0.705139791	0.90020336
KRAS	2	1069	0.708811741	0.90020336
ZNF521	9	1062	0.721467246	0.90020336
AXIN1	3	1068	0.686979909	0.90020336
BLM	2	1069	0.699754145	0.90020336
CLTC	2	1069	0.713469749	0.90020336
CDK12	3	1068	0.690245904	0.90020336
KEAP1	12	1059	0.706572011	0.90020336

NCOR1	4	1067	0.709457019	0.90020336
SFPQ	2	1069	0.712529485	0.90020336
SMARCB1	5	1066	0.717616775	0.90020336
EPAS1	4	1067	0.70099633	0.90020336
NKX2-1	3	1068	0.687079201	0.90020336
PRPF40B	2	1069	0.699531719	0.90020336
PRKAR1A	1	1070	0.727984809	0.90020336
TET1	3	1068	0.715409316	0.90020336
MLLT6	3	1068	0.732048106	0.901265256
TBX3	3	1068	0.736040281	0.903909116
ERCC4	4	1067	0.745695273	0.912307343
EZH2	3	1068	0.746989094	0.912307343
GATA3	2	1069	0.748464392	0.912307343
RBM15	2	1069	0.75235345	0.914772185
KTN1	3	1068	0.766615456	0.916006825
FUBP1	2	1069	0.773065921	0.916006825
PIK3CA	8	1063	0.763737045	0.916006825
PDGFRA	2	1069	0.773035979	0.916006825
STIL	5	1066	0.775801699	0.916006825
USP6	8	1063	0.767320444	0.916006825
ATRX	4	1067	0.761610146	0.916006825
CREB3L1	2	1069	0.762353385	0.916006825
KMT2C	21	1050	0.771286329	0.916006825
PTPRB	10	1061	0.761912626	0.916006825
RSPO2	3	1068	0.772784702	0.916006825
SDHA	2	1069	0.774518029	0.916006825
EZR	2	1069	0.782140719	0.916935231
MLLT1	1	1070	0.782201891	0.916935231
ARAF	2	1069	0.781870014	0.916935231
TLX1	2	1069	0.784937837	0.917946397
NCKIPSD	2	1069	0.788409876	0.919811522
ERC1	4	1067	0.792123864	0.921949391
AFF4	4	1067	0.803003111	0.929992137
ETV1	3	1068	0.80472789	0.929992137
DNM2	2	1069	0.803105249	0.929992137
PTPN13	4	1067	0.81217855	0.936394092
PDE4DIP	17	1054	0.822715466	0.940021287
BMP5	2	1069	0.822998228	0.940021287
EPHA3	6	1065	0.822423104	0.940021287
RGPD3	2	1069	0.822998228	0.940021287
FANCA	2	1069	0.825374694	0.940543256
CNBD1	5	1066	0.827655749	0.940954332
PBRM1	5	1066	0.832799605	0.944610663

PCM1	3	1068	0.836529221	0.946649696
MYO5A	5	1066	0.845311023	0.952622102
DCC	9	1062	0.845695131	0.952622102
CHD4	5	1066	0.85242154	0.957996685
ERCC3	2	1069	0.857505859	0.959790985
ARHGAP26	2	1069	0.859894372	0.959790985
SPEN	9	1062	0.859313062	0.959790985
CD79A	2	1069	0.868620762	0.961634769
MDM4	2	1069	0.869396332	0.961634769
REL	2	1069	0.866364606	0.961634769
CDKN1A	2	1069	0.864646094	0.961634769
UBR5	10	1061	0.878494168	0.96950933
KIF5B	4	1067	0.884294219	0.970279152
SMO	4	1067	0.885132206	0.970279152
RBM10	2	1069	0.882634026	0.970279152
FBXO11	2	1069	0.88888575	0.972218789
PALB2	5	1066	0.892281583	0.973759411
AMER1	2	1069	0.896764362	0.97647675
CXCR4	3	1068	0.903424176	0.979375767
LPP	7	1064	0.901665635	0.979375767
NTRK3	4	1067	0.911248313	0.985676983
MED12	2	1069	0.923076328	0.992306666
PTEN	9	1062	0.921393006	0.992306666
SH2B3	2	1069	0.923452734	0.992306666
PAX5	5	1066	0.926355656	0.993247859
ZEB1	6	1065	0.93149162	0.994402819
CREBBP	18	1053	0.930851586	0.994402819
CDH10	15	1056	0.935649617	0.99450827
SMC1A	2	1069	0.935627129	0.99450827
NSD1	5	1066	0.948391798	0.999504843
MYB	3	1068	0.951713498	0.999504843
DICER1	8	1063	0.958328255	0.999504843
DROSHA	4	1067	0.94380644	0.999504843
NF1	8	1063	0.955204553	0.999504843
FAT3	27	1044	0.958708727	0.999504843
FLNA	6	1065	0.956962272	0.999504843
RUNX1T1	7	1064	0.955694535	0.999504843
ZNF429	7	1064	0.94757365	0.999504843
CNTRL	2	1069	0.975583268	0.99954859
COL2A1	2	1069	0.992800056	0.99954859
BRD4	3	1068	0.990391182	0.99954859
FLT4	4	1067	0.974080672	0.99954859
JAK3	2	1069	0.991570864	0.99954859

PRDM16	2	1069	0.977869256	0.99954859
ARHGAP5	7	1064	0.985801449	0.99954859
BAP1	3	1068	0.997837021	0.99954859
CHEK2	8	1063	0.981957918	0.99954859
CUL3	7	1064	0.981762583	0.99954859
LZTR1	4	1067	0.99954859	0.99954859
POLE	2	1069	0.995315754	0.99954859
WRN	2	1069	0.996588653	0.99954859
ANK1	5	1066	0.965378393	0.99954859
FOXO4	2	1069	0.995315754	0.99954859
MAP3K1	4	1067	0.978829071	0.99954859
NTRK1	2	1069	0.978288532	0.99954859
PABPC1	11	1060	0.970620126	0.99954859
TP53	322	749	0.99562473	0.99954859
WT1	4	1067	0.979738043	0.99954859
