# Supplementary material for: A practical framework RNMF for exploring the association between mutational signatures and genes using gene cumulative contribution abundance
Source: Cancer Med. 2022 May 16;11(21):4053–69. doi: 10.1002/cam4.4717 (PMC9636515; doi:10.1002/cam4.4717)
Supplement: Supplementary file 16 — Table S7 [file CAM4-11-4053-s002.pdf]

Table S7a. The number of individuals representing each signature category.

Category	New	SBS1	SBS2	SBS3	SBS5	SBS13	SBS15	SBS16	SBS17b	SBS18	SBS22	SBS33
G1	90	160	149	91	150	153	142	84	8	146	95	120
G2	161	305	297	185	300	289	277	183	181	283	205	216
G3	15	31	33	19	30	28	26	15	19	31	27	23
G4	189	372	363	213	353	348	346	243	210	337	251	267

Table S7b. Training accuracy of different models on SBS New signature between G2 and G4.

**Models**	**Resnet50**	**Resnet101**	**Densenet121**	**Densenet161**	**MoblieNetV2**	**MoblieNetV3**	**InceptionV4**
**SBS (New) Best Accuracy(%)**	**68.571**	62.857	**71.429**	65.714	**68.571**	65.714	**68.571**

**Training**	Resnet50	**Densenet121**	MoblieNetV2	InceptionV4
**1**	65.714	65.714	57.143	62.857
**2**	68.571	68.571	60.000	62.857
**3**	60.000	71.429	68.571	65.714
**4**	57.143	62.857	57.143	62.857
**5**	62.857	68.571	62.857	65.714
**6**	62.857	62.857	54.286	60.000
**7**	65.714	65.714	54.286	60.000
**8**	57.143	68.571	62.857	62.857
**9**	65.714	65.714	65.714	68.571
**10**	60.000	71.429	65.714	62.857
**Mean Accuracy(%)**	**62.5713**	**67.1427**	**60.8571**	**63.4284**
**Median Accuracy(%)**	**62.857**	**67.1425**	**61.4285**	**62.857**

Note: for each result, we randomly select 90% of the samples as training, and the remaining 10% as test data set for analysis.

Table S7c. Training accuracy of densenet121 model on all SBSs.

**SBSs**	**New**	**SBS1**	**SBS2**	**SBS3**	**SBS5**	**SBS13**	**SBS15**	**SBS16**	**SBS17b**	**SBS18**	**SBS22**	**SBS33**
G2-VS-G4 Best Accuracy(%)	**71.4**	64.7	62.1	**72.5**	65.2	64.1	65.1	65.1	**77.5**	67.7	67.4	69.2
G2-VS-G4 Mean Accuracy(%)	**66.3**	59.9	57	**67.5**	60.3	58.8	61.1	60.2	**69**	63.2	61.7	61.6
G2G3-VS-G4 Best Accuracy(%)	**73**	66.2	66.7	**76.2**	62.3	67.2	69.2	71.1	**70.7**	65.2	69.4	68.6
G2G3-VS-G4 Mean Accuracy(%)	**64.9**	59.2	59.1	**66.2**	59.3	59.7	59.2	66.2	**64.6**	59.5	61.4	63.5

Note: for each result, we randomly select 90% of the samples as training, and the remaining 10% as test data set for analysis.
